# Systematic comparison between methods for the detection of influential spreaders in complex networks

**DOI:** 10.1038/s41598-019-51209-6

**Published:** 2019-10-22

**Authors:** Şirag Erkol, Claudio Castellano, Filippo Radicchi

**Affiliations:** 1 Center for Complex Networks and Systems Research, School of Informatics, Computing, and Engineering, Indiana University, Bloomington, Indiana 47408 USA; 2 Istituto dei Sistemi Complessi (ISC-CNR), Via dei Taurini 19, I-00185 Roma, Italy

**Keywords:** Complex networks, Phase transitions and critical phenomena

## Abstract

Influence maximization is the problem of finding the set of nodes of a network that maximizes the size of the outbreak of a spreading process occurring on the network. Solutions to this problem are important for strategic decisions in marketing and political campaigns. The typical setting consists in the identification of small sets of initial spreaders in very large networks. This setting makes the optimization problem computationally infeasible for standard greedy optimization algorithms that account simultaneously for information about network topology and spreading dynamics, leaving space only to heuristic methods based on the drastic approximation of relying on the geometry of the network alone. The literature on the subject is plenty of purely topological methods for the identification of influential spreaders in networks. However, it is unclear how far these methods are from being optimal. Here, we perform a systematic test of the performance of a multitude of heuristic methods for the identification of influential spreaders. We quantify the performance of the various methods on a corpus of 100 real-world networks; the corpus consists of networks small enough for the application of greedy optimization so that results from this algorithm are used as the baseline needed for the analysis of the performance of the other methods on the same corpus of networks. We find that relatively simple network metrics, such as adaptive degree or closeness centralities, are able to achieve performances very close to the baseline value, thus providing good support for the use of these metrics in large-scale problem settings. Also, we show that a further 2–5% improvement towards the baseline performance is achievable by hybrid algorithms that combine two or more topological metrics together. This final result is validated on a small collection of large graphs where greedy optimization is not applicable.

## Introduction

Every day, we witness the dissemination of new pieces of information in social networks^1–5^. Few of them become widespread; the vast majority, however, diffuse only over a vanishing portion of the network. Are there *a priori* identifiable features that allow for the early prediction of the outcome of a spreading process in a network? Many studies have pointed out that the “quality” or “attractiveness” of the information might have an effect on how far it may spread^1,6^. In mathematical models of information spreading, the notion of quality is typically quantified in terms of the probability of spreading events along individual edges in the social network. However, the spreading probability of individual edges is not the only key factor that determines the fate of a piece of information spreading in a network. The nodes that act as seeds for the spreading process may play a role that is more important than the actual probability to spread information along social contacts. Intuitively, if the diffusion process is seeded by central nodes, then the piece of information may reach large popularity; on the other hand, a piece of information originated from peripheral nodes is much less likely to become widespread.

The problem of selecting the best set of seed nodes for a spreading process in a network has been traditionally named as the problem of influence maximization. The problem is generally considered under the strong assumption of having full and exact knowledge of both the network topology and the spreading dynamics. We will adopt this line here too, although we remark that such an assumption is at least optimistic and may potentially lead, if not satisfied, to significant mistakes in the identification of the true influential spreaders^7^. The function that is optimized in influence maximization is the average value of the outbreak size. The optimization problem is solved for a given size of the seed set, generally much smaller than the network size. The problem was first formulated by Domingos and Richardson^8^, and later generalized by Kempe *et al*.^9^. In particular, Kempe *et al*. showed that influence maximization is a NP-hard problem, exactly solvable for very small networks only. Also, Kempe *et al*. demonstrated that for specific models of opinion spreading, such as the independent cascade and the linear threshold models, the average outbreak size is a submodular function, and thus greedy optimization algorithms allow to find, in polynomial time, approximate solutions that are less than a factor $(1-1/e)$ away from the true optimum^10^. The greedy algorithm actively uses information about the topology of the network and the dynamical rules of the spreading model. After the seminal work by Kempe *et al*., other similar greedy techniques for approximating solutions to the influence maximization problem have been proposed^11–14^. As all these algorithms require knowledge of the model at the basis of the spreading process, often obtained through numerical simulations, they all suffer from the limitation of being applicable to small-medium sized networks only. We remark that some attempts of greedy-like algorithms applicable to large networks have been made^15,16^. Those attempts, however, rely on approximate estimations of the outcome of numerical simulations, thus leading to solutions to the influence maximization problem that are generally inferior to the solutions obtained with straight greedy optimization.

On large networks, like those of interest in practical applications, solutions to the influence maximization problem are generally obtained via heuristic methods. The literature is full of examples^17–23^. Heuristic methods use complete information about the network structure, but they completely neglect information about the dynamical model of spreading. They are generally much faster than greedy algorithms, but clearly less effective. Their main limitations are two-fold. On the one hand, heuristic methods are characterized by the inability to account for the combined effect that seeds may have in a complex spreading process, as the set of influential nodes is built combining the best individual spreaders and their influence sets may be strongly overlapping. On the other hand, being based on purely topological properties, heuristic methods lack sensitivity to the features of the spreading dynamics and the variation of the associated parameters. Given the wealth of heuristic methods that have been proposed to identify influential nodes in networks, how different these methods are in terms of performance? Even more important, how far is the performance of the best heuristic methods from optimality, at least the achievable optimality provided by greedy algorithms? We realized that no clear answer to these fundamental questions can be found in current literature, and we decided to fill this gap of knowledge here.

The present paper reports on a systematic test of 16 heuristic methods that have been proposed to approximate solutions to the influence maximization problem. Our analysis is based on a corpus of 100 real-world networks, and performance of the various heuristic methods is quantified for SIR-like spreading processes. Despite the various methods rely on rather different centrality metrics, we find that many of them are able to achieve comparable performances. When used to select the top 5% initial seeds of spreading in real-networks, the best performing methods show levels of performance that are within 90% from those achievable by greedy optimization, so that the room for potential improvement appears small. We show that one way to achieve better performances is relying on hybrid methods that combine two or more centrality metrics together. We validate this final result on a small set of large-scale networks.

## Methods

### Networks

In this study, we focus most of our attention on a corpus of 100, undirected and unweighted, real-world networks. Sizes of these networks range from 100 to 30,000 nodes, and their density varies between 0.0001 and 0.25. The corpus is composed of networks of small to medium size on purpose, as these allow for the application of greedy optimization in the solution of the influence maximization problem. We consider networks from different domains. Specifically, our corpus of networks include 63 social, 16 technological, 10 information, 8 biological, and 3 transportation networks. Details about the analyzed networks can be found in the SM1. In the final part of the paper, we validate some of our findings on 9 large real-world social and information networks with sizes ranging from 50,000 to slightly more than 1,000,000. Details are provided in Table 3.

### Spreading dynamics

We concentrate our attention on the Independent Cascade Model (ICM)^9^. This is a very popular model in studies focusing on the influence maximization problem. The ICM is a simplified version of the Susceptible-Infected-Recovered (SIR) model^24^. Nodes can be in either one of the three states S, I, or R. At the beginning of the dynamics, all nodes start in the S state except for those who are selected to be the initial spreaders, which are assigned to the I state. At each step of the model, all nodes in state I try to infect their neighbors in state S with probability *p*; then, they recover immediately, by changing their states from I to R. Nodes in state R never change their state and no longer participate to the spreading dynamics. The dynamics continue until there are no nodes left in state I. The size of the outbreak is calculated by counting the number of nodes that ended up in state R at the end of the spreading dynamics. As the spreading from one node to another happens with probability *p*, the model has a stochastic nature. To properly account for the stochastic nature of the model, all our results are obtained as average values over 50 independent numerical simulations for every given initial condition.

### Methods for the selection of influential spreaders

In total, we consider 18 methods for the identification of influential spreaders in networks (see Table 1). Each method outputs a list of nodes in a specific order from the most influential node to the least influential node. We use this rank to construct, in a sequential manner, the set of the top spreaders according to a particular method. The various methods take as input different type/amount of information, and make use of rather different types of rankings. As a consequence, the computational complexity of the various methods may be significantly different. For illustrative purposes, we decided to group the 18 methods for the selection of influential spreaders into four main groups.

**Table 1:** Methods for the selection of influential spreaders.

Group	Method	Abbrev.	Ref.	Complexity
Baseline	Greedy	G	^12^	cubic
	Random	R	—	constant
Local	Degree	D	—	linear
	Adaptive Degree	AD	^12^	linear
Global	Betweenness	B	^25^	quadratic
	Closeness	C	^26^	quadratic
	Eigenvector	E	^27^	linear
	Katz	K	^28^	linear
	PageRank	PR	^31^	linear
	Non-backtracking	NB	^29^	linear
	Adaptive NB	ANB	^32^	quadratic
Intermediate	k-shell	KS	^33^	linear
	LocalRank	LR	^34^	linear
	h-index	H	^35^	linear
	CoreHD	CD	^37^	linear
	Collective Influence, $\ell =1$	CI1	^36^	linear
	Collective Influence, $\ell =2$	CI2	^36^	linear
	Expl. Immunization	EI	^38^	linear

We list basic details of all the methods for the detection of influential spreaders in complex networks that we consider in this study. Each row of the table refers to a specific method. From left to right, we report the full name of the method, the abbreviation of the method name, the reference of the paper where the method was introduced, and the computational complexity of the method. Computational complexities reported in the table are obtained under the realistic assumption that methods are applied to sparse networks where the number of edges scales linearly with the network size. Methods are further grouped into different categories, i.e., baseline, local, global, and intermediate, depending on their properties.

The group of baseline methods is formed by the methods greedy and random. The greedy algorithm is the best performing method available on the market, thus providing an upper bound for the performance of all other methods. The greedy algorithm uses all available information about network topology and spreading dynamics. For instance, the algorithm provides different solutions depending on the value of the spreading probability *p*. For the greedy method applied to the ICM, we rely on the Chen *et al*.’s^12^ algorithm, which makes use of the mapping between ICM and bond percolation to obtain faster results regarding the simulations of the spreading process. The random method instead represents a lower bound for the performances of other methods. The method just outputs nodes of the network in random order, *de facto* neglecting any prior information regarding system topology and dynamics.

The remaining 16 of the 18 methods are purely topological methods in the sense that they rely on heuristics that are calculated using full knowledge of the network structure, but no information at all about spreading dynamics. According to these methods the influence of a node is proportional to a network centrality metric. Depending on the nature of the centrality metric used, we classify the topological methods into three groups.

First, methods that use local topological information, in the sense that values of the centrality metric associated to every node are computed using information about their nearest neighbors only. For example, degree centrality, which consists of counting the number of neighbors of a node, belongs to this category. A variant of the degree method, called adaptive degree method, which was proposed by Chen *et al*.^12^ is classified as a local method too.

Second, methods that are based on global centrality metrics whose computation, at the level of the individual nodes, requires complete knowledge about the whole network structure. This group consists of methods relying on betweenness^25^, closeness^26^, eigenvector^27^, Katz^28^, non-backtracking^29,30^, and pagerank^31^ centralities. As a part of this group we also considered the method based on an adaptive variant of the non-backtracking centrality^32^.

Finally, we consider several methods that rely on intermediate topological information (e.g., nearest neighbors, next-nearest neighbors) for the computation of node centrality metrics. This group consists of the methods that rely on the metrics k-shell^33^, localrank^34^, and h-index^35^. We classify in the intermediate group also methods that are based on collective influence^36^, coreHD^37^, and explosive immunization score^38^. These are methods introduced with the goal of approximating solutions to the optimal percolation problem^36^, an optimization problem that has similarities with, but is different from the one considered in influence maximization^39^. We stress that we consider two variations of the *CI* method. Specifically, we consider *CI*1 and *CI*2, where the numerical value indicates the value of the parameter that defines the centrality metric^36^.

### Evaluating the performance of methods for the selection of influential spreaders

Potentially all selection methods described above are subjected to statistical fluctuations in the sense that they may generate a different ranking for the nodes at each run. This is due to the presence of ties in the ranking of nodes, and the fact that we break ties by randomly selecting nodes with the same rank position. To account for statistical fluctuations, we apply every method *R* = 10 independent times to generate *R* rankings for the nodes. We consider each of these rankings to sequentially construct sets of top spreaders. Specifically, we indicate as ${{\mathscr{S}}}_{m}^{(t,r)}$ the set of top *t* *N* spreaders identified by method *m* in instance *r* of the method and for a given network with *N* nodes. For every set ${{\mathscr{S}}}_{m}^{(t,r)}$, we run 50 different times the ICM model, and measure the average value of the outbreak size $O[{{\mathscr{S}}}_{m}^{(t,r)}]$. We then repeat the operation for every instance *r* of the method, and take the average over the *R* potentially different sets, namely

1 $${V}_{m}^{(t)}=\frac{1}{R}\,\mathop{\sum }\limits_{r=1}^{R}\,O[{{\mathscr{S}}}_{m}^{(t,r)}].$$

**Figure 1:**
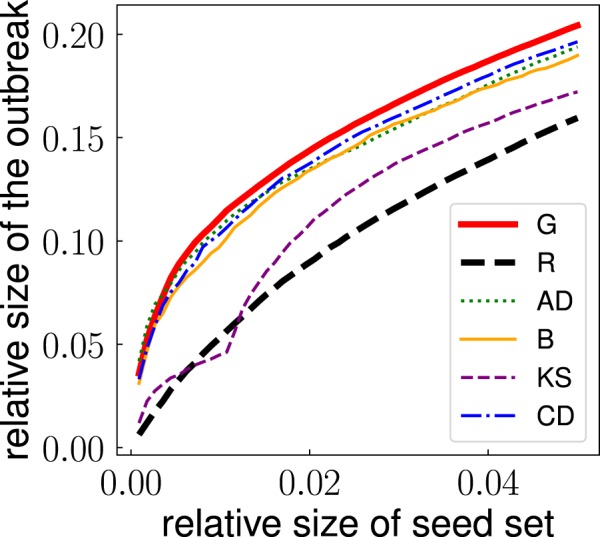
Relative size of the outbreak as a function of the relative size of the seed set for the email communication network of ref.^43^. To obtain relative values, we divide outbreak size and seed set size by the total number of nodes in the network. Relative measures allow for an immediate comparison across networks with different sizes. We compare the performance of different methods for the selection of influential nodes. Outbreak size is calculated for ICM dynamics at critical threshold ${p}_{c}=0.056$. To avoid overcrowding, we display results only for a subset of the methods considered in the paper.

Figure 1 displays how the relative size of the outbreak ${V}_{m}^{(t)}/N$ grows as function of the relative seed set size *t* for some of the methods for the identification of top spreaders considered in this paper. Given the amount of simulations performed, the standard error associated with the average value of the outbreak size of Eq. (1) is always very small. We therefore neglect it in all the considerations and analyses below. Figure 1 clearly shows that the greedy and random algorithms are good baselines for the performances of the other methods. For instance, the greedy algorithm outperforms all other methods. This result is confirmed across the entire corpus of networks we analyzed in this paper (see SM1 and SM2). In a few networks, some heuristic methods are able to slightly outperform the greedy algorithm. This seems to happen only in the case of relatively small networks, composed of hundreds or less nodes. Similarly, all methods perform better than the random selection method, although there are quite a few cases where randomly selecting seeds perform as well as selecting seeds according to some topological heuristic.

As a measure for the performance of method *m* in the identification of the top *T* *N* influential spreaders of a given network, we evaluate the area under the curves of Fig. 1 up to a pre-imposed *T* value

2 $${q}_{m}^{(T)}=\frac{1}{N}\,{\int }_{0}^{T}\,dt\,{V}_{m}^{(t)}.$$

As the size of set of top spreaders are linearly dependent from the size of the network *N*, we can easily aggregate results obtained over the entire corpus of real-world networks at our disposal. Specifically, results in the main paper are obtained for $T=0.05$. We report results for $T=0.1$ in the SM2. No significant differences between the two cases are apparent. As some of the methods considered in the paper are characterized by large computational complexity (see Table 1), we couldn’t consider $T > 0.1$. We note, however, that studying the performance of methods for the identification of influential spreaders has a meaning only for small *T* values, given that in practical applications the seeding is generally performed on a vanishing portion of the system. Also, we test the validity of all results using ${V}_{m}^{(T)}$ as a main metric of performance, instead of its integral of Eq. (2). Results are reported in the SM2. No significant changes with respect to the results presented here in the main paper are apparent.

**Figure 2:**
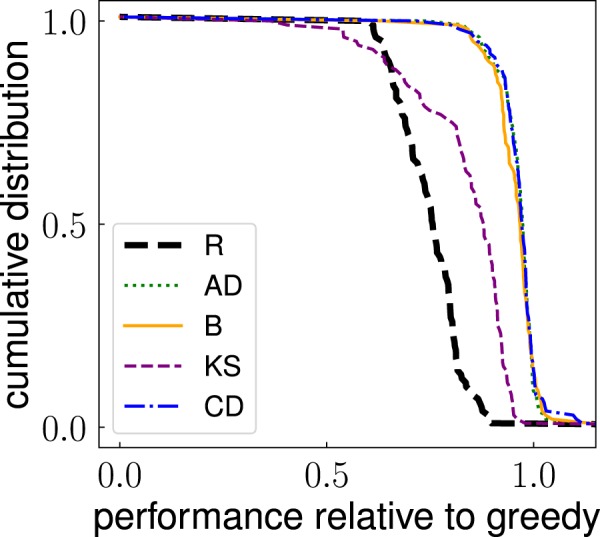
Cumulative distribution of the relative performance ${g}_{m}^{(T)}$ (for $T=0.05$) obtained by using a method for the identification of influential spreaders different from the greedy algorithm. The metric of relative performance is defined in Eq. 3. The distribution is obtained considering all networks in our dataset. For every network, the outbreak size is calculated for ICM dynamics at critical threshold *p*_*c*_. See details in the SM1. To avoid overcrowding, we display results only for the same subset of the methods as already considered in Fig. 1.

As the greedy algorithm provides an upper bound for the performance of the other methods, we use it as a term of comparison for all other methods in our systematic analysis. We consider two main metrics of performance. The first measure is based on a comparison between the outbreak size obtainable by a method compared to the one obtained using the greedy identification method. Specifically, given a network, we first compute

3 $${g}_{m}^{(T)}=\frac{{q}_{m}^{(T)}}{{q}_{G}^{(T)}},$$

where we used the abbreviation ${q}_{G}^{(T)}$ to indicate the expression of Eq. (2) for the greedy algorithm, i.e., $m=G$. Then, we evaluate the performance relative to greedy for all networks in our dataset, and summarize the results in Fig. 2 where we display the cumulative distribution of this quantity for some of the methods. To obtain a single number for the performance of the method over the entire corpus of networks, we define the overall performance $\langle {g}_{m}^{(T)}\rangle$ given by the average value of the metric defined in Eq. (3) over all real networks in the dataset. We remark that statistical errors associated to the metrics of Eqs (1), (2) and (3) are negligible given the large number of independent numerical simulations used to determine their average values. A similar statement, however, doesn’t hold for the overall performance $\langle {g}_{m}^{(T)}\rangle$ due to the relatively small size of the corpus of networks analyzed. In the following, we associate the standard error of the mean to any estimate of the average value $\langle {g}_{m}^{(T)}\rangle$ obtained on samples of real-world networks.

The second metric of performance instead neglects the size of the outbreak, and focuses only on the identity of the nodes identified by the method *m*. For the actual solution of the problem of influence maximization, this second metric is clearly much less important than the one previously considered. However, the metric can tell us something more about the topological properties of the set of top spreaders in networks. Given a network, we evaluate the frequency ${f}_{m}^{(T,i)}$ of every node *i* to be in the set of top *T* *N* spreaders according to method *m* over $R=10$ runs of the algorithm. We then compute the precision of the method relative to the greedy algorithm as

4 $${r}_{m}^{(T)}=\frac{1}{T\,N}\,\mathop{\sum }\limits_{i=1}^{N}\,{f}_{m}^{(T,i)}\,{f}_{G}^{(T,i)}.$$

**Figure 3:**
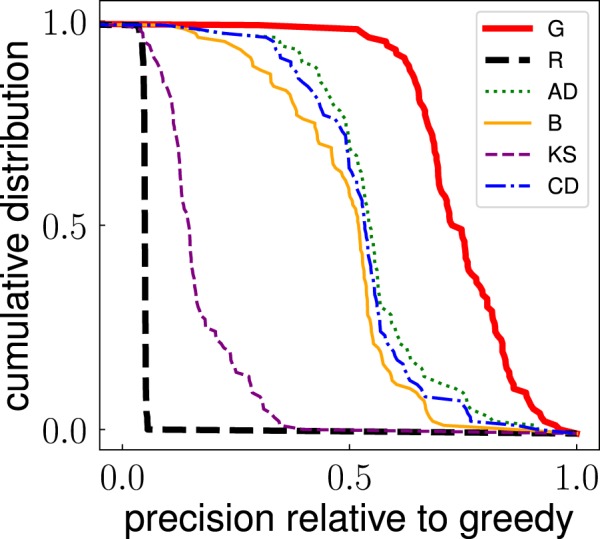
Cumulative distribution of the precision metric ${r}_{m}^{(T)}$ defined in Eq. (4) for $T=0.05$. The distribution is obtained considering all networks in our dataset. Results for the greedy algorithm used in the comparison are those obtained for ICM dynamics at critical threshold *p*_*c*_. See details in the SM1. To avoid overcrowding, we display results only for the same subset of the methods as already considered in Fig. 1.

We note that Eq. (4) can be used to measure the self-consistency of the greedy method by setting $m=G$. The cumulative distribution of the precision metric defined in Eq. (4) across the entire network dataset is displayed in Fig. 3. The plot shows high level of precision between some methods and the greedy algorithm. The random selection method generates a distribution well peaked around the value *T*. We characterize the generic method *m* with a metric of overall precision $\langle {r}_{m}^{(T)}\rangle$ as the average value of the precision defined in Eq. (4) over the entire corpus of real networks. Statistical errors associated to measure of $\langle {r}_{m}^{(T)}\rangle$ are quantified in terms of standard error of the mean. The value of $\langle {r}_{m}^{(T)}\rangle$ tells us how much the method *m* is similar to the baseline provided by the greedy algorithm in the identification of the top spreaders across the entire corpus of networks at our disposal.

## Results

### Individual methods

Armed with the metrics defined in the section above, we test the various methods for the identification of influential spreaders for ICM dynamics over the entire corpus of real networks at our disposal. We remark that both the identity and performance of the true set of influential spreaders may be dependent on the actual value of the spreading probability *p* in the ICM model, so that the performance of the various seed selection methods needs to be evaluated at different values of the spreading probability *p*. For instance, for the extreme cases $p=0$ and $p=1$, predictions are trivial in the sense that all methods have exactly the same performance in terms of outbreak size. The prediction of methods performance is instead non trivial when the uncertainty of the spreading outcome is maximal. For this reason, we focus our attention on ICM dynamics around the critical threshold $p={p}_{c}$. To perform the analysis, we first evaluate the critical threshold values *p*_*c*_ for every network in the database. Specifically, we rely on mapping between bond percolation and the ICM, and we apply the Newman-Ziff algorithm to evaluate *p*_*c*_^40,41^. *p*_*c*_ values for the various networks are reported in the SM1. We then consider ICM dynamics for three distinct values of *p*: (i) subcritical regime at $p={p}_{c}/2$; (ii) critical regime at $p={p}_{c}$; (iii) supercritical regime at $p=2{p}_{c}$.

**Figure 4:**
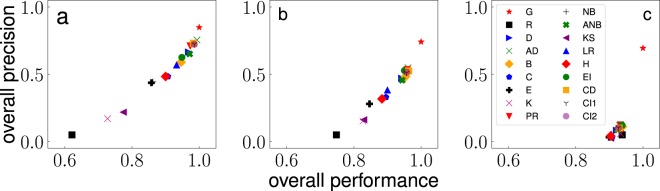
Performance and precision of methods for the identification of influential spreaders in real networks. Results are based on the systematic analysis of 100 real-world networks. For each network, we first evaluate the critical value of the spreading probability *p*_*c*_ for ICM dynamics. Then, we consider the analysis for three distinct phases of spreading: (**a**) $p={p}_{c}/2$, (**b**) $p={p}_{c}$, (**c**) $p=2{p}_{c}$. Each point in the various panels corresponds to one method. Every method is used to identify the top *T* *N*, with $T=0.05$, spreaders in the networks. For clarity of the figure, methods are identified by the same abbreviations as those defined in Table 1. Methods are characterized by the metrics of performance defined in the paper. Both these metrics relate the performance of a generic method *m* to the one of the greedy algorithm. Overall performance $\langle {g}_{m}\rangle$ is a metric of performance that relies on the size of the outbreak associated with the set of influential spreaders identified by the method compared to the typical outbreak obtained with the greedy algorithm. Overall precision $\langle {r}_{m}\rangle$ instead quantifies the overlap between the sets of spreaders identified by a method and those identified by the greedy algorithm. Error bars (not shown) quantifying the standard errors of the mean associated with the numerical estimates of $\langle {g}_{m}\rangle$ and $\langle {r}_{m}\rangle$ are of the same size as of the symbols used in the visualization.

Results of our analysis are summarized in Fig. 4. Every method is used to identify the set of top *T* *N* nodes in the networks, with $T=0.05$. In the figure, we represent results for each method *m* in the plane $(\langle {g}_{m}\rangle ,\langle {r}_{m}\rangle )$. Numerical values of $\langle {g}_{m}\rangle$ and $\langle {r}_{m}\rangle$, as well as their associated statistical errors, are reported in SM2. Please note that we dropped the suffix *T* to simplify the notation. We remark that the performance of every method *m* is measured in relation to the performance of the greedy method, i.e., $m=G$. By definition, we have $\langle {g}_{G}\rangle =1$; we find instead that the self-consistency score is $\langle {r}_{G}\rangle  < 1$ meaning that optimal sets identified by the greedy algorithm have some degree of variability. Such a variability seems due to the existence of (quasi)degenerate solutions to the influence maximization problem, i.e., different seed sets corresponding to similar outbreak sizes. The presence of statistical fluctuations in the numerical estimates of the outbreak size may be an additional confounding factor that exacerbates the degeneracy of greedy solutions. An interesting finding is the absence of a strong dependence of $\langle {r}_{G}\rangle$ from the dynamical regimes of the ICM. The other important reference point in the plane is given by the random method ($m=R$). By definition, we have that $\langle {r}_{R}\rangle \simeq T=0.05$. $\langle {g}_{R}\rangle$ values instead strongly depend on the dynamical regime.

In the subcritical regime (see Fig. 4a), the two metrics $\langle {g}_{m}\rangle$ and $\langle {r}_{m}\rangle$ are tightly related one to the other. Adaptive degree ($m=AD$) outperforms all other methods in both metrics. Other methods that perform very well are those based on algorithms relying on the Degree ($m=D$), Adaptive Non-Backtracking ($m=ANB$) and PageRank ($m=PR$) centralities, as well as those based on the CoreHD ($m=CD$) and Collective Influence ($m=CI$) algorithms. Similar considerations apply to the critical regime (Fig. 4b). The most significant change with respect to the subcritical regime is a slight decrease of range of values for the performance metric of the algorithms. In the supercritical regime (Fig. 4c), there is no longer a proper distinction between the various methods in terms of performance.

A remarkable feature emerging from Fig. 4 is that the overall performance is rather high. For most of the methods values are above 0.9 for all values of *p*, and even random selection provides a performance always larger than 0.6. This observation somehow helps to properly weigh the importance of greedy algorithms for influence maximization: while their solutions are guaranteed to be not too far from the true optimum, their performance can be almost achieved by simple and much more easily implemented purely topological methods.

**Figure 5:**
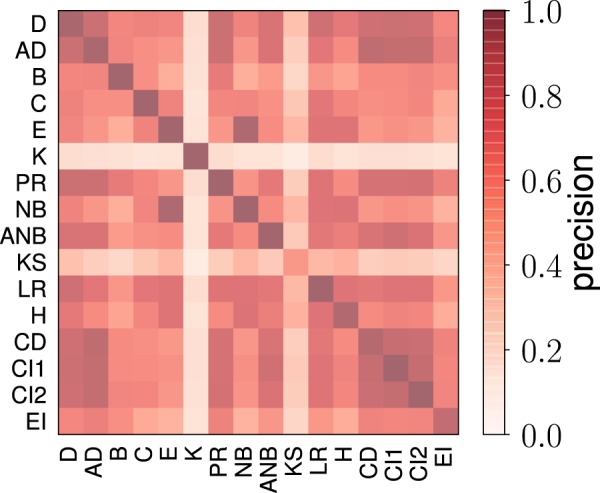
Pairwise comparison among methods for the identification of influential spreaders. For every pair of methods *m*_1_ and *m*_2_, we evaluated the overlap ${r}_{{m}_{1},{m}_{2}}^{(T)}$ among the two sets of top *T* *N* influential spreaders found by the methods in the network using a precision metric similar to the one of Eq. (4), i.e., ${r}_{{m}_{1},{m}_{2}}^{(T)}=\frac{1}{TN}\,{\sum }_{i=1}^{N}\,{f}_{{m}_{1}}^{(T,i)}\,{f}_{{m}_{2}}^{(T,i)}$. We then estimated the average value of the precision over the entire corpus of real networks at our disposal. In the figure, dark colors corresponds to high values of precision; low precision values are represented with light colors. Acronyms of the methods are defined in Table 1. Methods are listed in the table according to the same order as they appear in Table 1.

The similarity in the performance between the various methods can be deduced by a straight pair-wise comparison between the sets of top influential nodes identified by the various methods across the entire corpus of real networks at our disposal. The results of this analysis are summarized in Fig. 5. Top-performing methods provide sets of influential nodes very similar to each other; methods with low performance instead generally identify influential nodes that are rarely selected by any other method.

In the SM2, we repeat the same exercise by computing the performance scores restricted to different subsets of the whole corpus of networks. The subsets correspond to networks from the same domain (e.g., social, technological, transportation); we do not find any significant change in the main outcome of the analysis.

We further consider artificial networks created with the Barabasi-Albert (BA) model^42^. Results are very similar to those obtained on real-world networks (see SM2). In summary, it seems that the main results of the paper are unchanged by the nature/type of the network substrate where spreading is occurring.

### Hybrid methods

In this section, we report on the performance of hybrid methods for the identification of top spreaders in the network obtained from linear combinations of the individual methods considered so far. Specifically, we first select a certain number of individual methods to form a hybrid method ${\mathcal H} =\{{m}_{1},{m}_{2},\ldots ,{m}_{| {\mathcal H} |}\}$. We associate to every node *i* in a given network a score ${s}_{i}^{( {\mathcal H} )}$ that is a linear combination of the scores associated with individual methods, namely

5 $${s}_{ {\mathcal H} }^{(i)}=\sum _{m\in  {\mathcal H} }\,{c}_{m}\,{s}_{m}^{(i)}.$$

In Eq. (5), ${s}_{m}^{(i)}$ is the normalized score of node *i* in the network according to the topological metric used by method *m*. The normalization (L^2^-norm) has the purpose of making scores of comparable magnitude across methods. The best estimates of the linear coefficients *c*_*m*_ are then obtained using information from the greedy algorithm. We use linear regression to find the best linear fit between ${s}_{ {\mathcal H} }^{(i)}$ and ${f}_{G}^{(T,i)}$, i.e., the probability that node *i* is identified by the greedy algorithm in the set of top *T* *N* influential nodes in the network. Best estimates of the coefficients are obtained relying on a training set composed of 80% of networks randomly chosen out of the corpus of real networks at our disposal. We then test the hybrid method ${\mathcal H}$ on the remaining 20% of the corpus, where we measure overall performance and overall precision. We replicate the entire procedure 1,000 times to quantify uncertainty associated with both the best estimates of the linear coefficients as well as the measured values of the performance metrics.

We consider several hybrid methods consisting in the combination of two and three individual centrality metrics. In general, we combine together centrality methods that differ on the basis of their classification in local, global and intermediate methods (see Table 1). Results for some hybrid methods are reported in Table 2. Several remarks are in order. First, with respect to the case of individual methods, there is an increase in the measured values of the overall precision $\langle {r}_{m}\rangle$. This tells us that the coefficients learned from the training set can be meaningfully used on other networks to mimic greedy optimization in terms of topological features only. The overall performance $\langle {g}_{m}\rangle$ of hybrid methods increases too; improvements beat even by 2–5% the best individual methods. Second, when similar individual methods are combined together into an hybrid method, one of the two gets the biggest part of the weight compared to the other. For example, the hybrid method ${\mathcal H} =\{AD,B\}$ learned from data is almost a pure AD method in both the subcritical and critical regimes. Third, the coefficients of the linear combination of Eq. (5) can also be negative. For example, for the hybrid method ${\mathcal H} =\{AD,PR,LR\}$ in the critical regime, ${c}_{LR} < 0$. Thanks to this fact, the method outperforms in both the critical and subcritical regimes all other methods considered in this paper. We stress that the finding ${c}_{LR} < 0$ doesn’t mean that LR centrality is anticorrelated with node influence. ${c}_{LR} < 0$, in fact, is observed only when LR is used in combination with other metrics. Indeed, LR centrality is positively correlated with node influence when LR is used as the only method for the identification of spreaders, as Fig. 4 clearly shows.

**Table 2:** Hybrid methods for the identification of influential spreaders in networks.

Method	Features	Subcrit.	Critical	Supercrit.
AD	${c}_{AD}$	1.000	1.000	1.000
	$\langle {g}_{m}\rangle$	0.993	0.961	0.931
	$\langle {r}_{m}\rangle$	0.755	0.548	0.119
CD	${c}_{CD}$	1.000	1.000	1.000
	$\langle {g}_{m}\rangle$	0.983	0.963	0.929
	$\langle {r}_{m}\rangle$	0.730	0.525	0.100
B	${c}_{B}$	1.000	1.000	1.000
	$\langle {g}_{m}\rangle$	0.946	0.954	0.938
	$\langle {r}_{m}\rangle$	0.590	0.483	0.110
AD,B	${c}_{AD}$	0.718	0.590	0.023
	${c}_{B}$	−0.027	0.046	0.069
	$\langle {g}_{m}\rangle$	0.987	0.964	0.936
	$\langle {r}_{m}\rangle$	0.755	0.551	0.116
AD,PR,LR	${c}_{AD}$	1.189	1.044	0.115
	${c}_{PR}$	−0.266	0.145	0.772
	${c}_{LR}$	−0.336	−0.632	−0.771
	$\langle {g}_{m}\rangle$	0.991	0.980	0.971
	$\langle {r}_{m}\rangle$	0.806	0.616	0.300
PR,LR,CD	${c}_{PR}$	0.006	0.386	0.803
	${c}_{LR}$	−0.419	−0.702	−0.771
	${c}_{CD}$	1.028	0.898	0.088
	$\langle {g}_{m}\rangle$	0.985	0.979	0.971
	$\langle {r}_{m}\rangle$	0.784	0.597	0.293
AD,B,LR	${c}_{AD}$	1.096	1.047	0.343
	${c}_{B}$	−0.010	0.067	0.083
	${c}_{LR}$	−0.466	−0.565	−0.395
	$\langle {g}_{m}\rangle$	0.993	0.976	0.952
	$\langle {r}_{m}\rangle$	0.810	0.625	0.220
PR,LR,EI	${c}_{PR}$	0.304	0.583	0.740
	${c}_{LR}$	0.101	−0.251	−0.733
	${c}_{EI}$	0.235	0.277	0.121
	$\langle {g}_{m}\rangle$	0.973	0.964	0.970
	$\langle {r}_{m}\rangle$	0.698	0.589	0.304

The table is organized in various blocks, each corresponding to a specific method. For every method *m*, either individual or hybrid, we report performance values for the three different dynamical regimes in terms of overall performance $\langle {g}_{m}\rangle$ and overall precision $\langle {r}_{m}\rangle$. The top three blocks correspond to the best individual methods in the three regimes according to overall performance metric. The remaining blocks are for hybrid methods. In each block, the first rows report values of the coefficient *c*_*m*_ of the individual method *m* in the definition of the hybrid method. We report the averages for the coefficient values over 1,000 iterations of the learning algorithm. The bottom two rows in each block correspond instead to the values of the performance metrics. Errors associated with all these measures are always smaller than 0.001, and they are omitted from the table for clarity.

To validate the use of hybrid methods for the identification of influential spreaders, we apply the top-performing hybrid method ${\mathcal H} =\{AD,PR,LR\}$ to large social and information networks. Results are reported in Table 3. These networks are too big for the application of greedy optimization, thus the performance of the hybrid method is compared to the one of the method AD by taking the ratio $\langle {g}_{ {\mathcal H} }\rangle /\langle {g}_{AD}\rangle$. Please note that AD is one of the best individual methods for the identification of influential spreaders according to our analysis on the corpus of small/medium networks. When applying the hybrid method to large networks, we use the same values of the linear coefficients learned from small/medium networks and listed in Table 2. Overall, we see that the hybrid method generates improvements in the detection of influential spreaders compared to the simple AD method. Improvements are almost negligible in the subcritical regime. They are instead significant in both the critical and supercritical dynamical regimes, although in the latter case there are wide variations, with striking performance decrease for some networks. On average, we register improvements of 2–5%. These values are in line to those that can be measured in the corpus of small/medium networks, thus providing additional support to the robustness and generality of our finding. It should be stressed that the hybrid method uses a slightly larger amount of information than the one at disposal of the individual AD method. This might be at the root of the observed performance increase. As a matter of fact, linear coefficients change their value depending on the dynamical regime, so the ranking of the nodes. On the other hand, the improvement in effectiveness doesn’t cause drawbacks in efficiency. Linear coefficients of the various dynamical regimes are given. Also, the computational complexity of estimating numerically the critical threshold *p*_*c*_ scales linearly with system size. *De facto*, the computational complexity of the overall hybrid method is the same as the one of the individual methods, making it applicable to very large networks.

**Table 3:** Identification of influential spreaders in large networks.

Network	*N*	*E*	*p*_*c*_	Ref.	url	$\langle {{\boldsymbol{g}}}_{{\boldsymbol{ {\mathcal H} }}}\rangle /\langle {{\boldsymbol{g}}}_{{\boldsymbol{AD}}}\rangle$		
						Subcrit.	Critical	Supercrit.
Slashdot	51,083	116,573	0.0262	^44, 45^	url	1.003	1.017	1.062
Gnutella, Aug. 31, 2002	62,561	147,878	0.0956	^46, 47^	url	1.009	1.040	1.039
Epinions	75,877	405,739	0.0062	^45, 48^	url	1.012	1.057	1.130
Flickr	105,722	2,316,668	0.0142	^45, 49^	url	1.007	1.082	1.242
Gowalla	196,591	950,327	0.0073	^45, 50^	url	1.011	1.024	1.066
EU email	224,832	339,925	0.0119	^45, 47^	url	1.002	1.009	0.923
Web Stanford	255,265	1,941,926	0.0598	^51^	url	1.009	1.031	1.035
Amazon, Mar. 2, 2003	262,111	899,792	0.0940	^52^	url	1.008	1.025	0.994
YouTube friend. net.	1,134,890	2,987,624	0.0063	^45, 53^	url	1.004	1.013	0.952
*Average on large networks*						1.007 ± 0.001	1.033 ± 0.007	1.050 ± 0.030
*Average on the corpus of 100 networks*						1.001 ± 0.002	1.021 ± 0.003	1.043 ± 0.005

We compare the performance of the hybrid method ${\mathcal H} =\{{\rm{AD}},{\rm{PR}},{\rm{LR}}\}$ with the individual method AD. For the hybrid method, we use the values of the coefficients reported in Table 2. From left to right, we report the name of the network, number of nodes in the giant component, number of edges in the giant component, critical value *p*_*c*_ of the spreading probability, references to studies where the network was first analyzed, url where network data were downloaded, value of the ratio $\langle {g}_{ {\mathcal H} }\rangle /\langle {g}_{AD}\rangle$ between the performance metric of the hybrid method ${\mathcal H} =\{{\rm{AD}},{\rm{PR}},{\rm{LR}}\}$ and the one of the individual method AD for the subcritical, critical and supercritical regimes. The bottom two lines in the table report, for each dynamical regime, average values and standard errors of the mean for the ratios $\langle {g}_{ {\mathcal H} }\rangle /\langle {g}_{AD}\rangle$ over the set of large networks and over the corpus of 100 networks considered in the rest of the paper.

## Conclusions

The goal of this paper was to comparatively analyze the performances of heuristic methods aimed at the identification of influential spreaders in networks. We focused our attention on the spreading dynamics modeled by the independent cascade model, and studied a total of 16 methods for the identification of the influential spreaders that are being used widely in influence maximization studies. We performed a systematic comparison between the various methods by means of extensive numerical experiments on a large corpus of 100 real-world networks. We further drew upper- and lower-bounds for the performance values achievable in the problem by using respectively results from greedy optimization and random selection. We found that the performance of many simple heuristic methods is not far from that of the more computationally costly greedy algorithm. In this framework, the simplest and most effective strategy among those already on the market that can be used to identify top spreaders in large networks is the adaptive degree centrality. The method based on adaptive degree centrality displays an overall performance score that is 96% of the upper-baseline value in the critical regime of spreading, if used to select a set of top spreaders with size equal to 5% of the entire network. Several other methods have comparable performances to adaptive degree centrality. The overlap between influential spreaders selected by heuristic methods and by the greedy algorithm is considerably lower, but this is not surprising given the NP-complete nature of the optimization problem. We finally found that a potential way to get closer to optimality consists in combining different centrality metrics to create hybrid methods. We found that some combinations of three metrics are able to achieve 98% of the upper-baseline value in the critical regime of spreading.

## Supplementary information

SM1

SM2

## Data Availability

The datasets used in this article are all publicly available from the cited sources.
