# Supplementary material for: Systematic comparison between methods for the detection of influential spreaders in complex networks
Source: Sci Rep. 2019 Oct 22;9:15095. doi: 10.1038/s41598-019-51209-6 (PMC6805897; doi:10.1038/s41598-019-51209-6)
Supplement: Supplementary file 1 — SM1 [file 41598_2019_51209_MOESM1_ESM.pdf]

1 Table of Networks in the Corpus

**Network**	**Type**	**N**	**E**	$\mathbf{p_c}$	**Ref.**	**url**
Political books	information	105	441	0.100	[1]	url
College football	social	115	613	0.134	[2]	url
S208	technological	122	189	0.466	[3]	url
High school, 2011	social	126	1709	0.038	[4]	url
Bay Dry	biological	128	2106	0.030	[5, 6]	url
Bay Wet	biological	128	2075	0.031	[6]	url
Radoslaw Email	social	167	3250	0.020	[7, 6]	url
High school, 2012	social	180	2220	0.044	[4]	url
Little Rock Lake	biological	183	2434	0.030	[8, 6]	url
Jazz	social	198	2742	0.031	[9]	url
S420	technological	252	399	0.451	[3]	url
C. Elegans, neural	biological	297	2148	0.045	[10]	url
Network Science	social	379	914	0.398	[11]	url
Dublin	social	410	2765	0.078	[12, 6]	url
US Air Trasportation	transportation	500	2980	0.026	[13]	url
S838	technological	512	819	0.349	[3]	url
Yeast, transcription	biological	662	1062	0.246	[14]	url
Caltech	social	762	16651	0.016	[15, 16, 17]	url
Reed	social	962	18812	0.015	[15, 16, 17]	url
Mouse retina	biological	1076	90811	0.004	[18, 19]	url
URV email	social	1133	5451	0.056	[20]	url
Political blogs	information	1222	16714	0.015	[1]	url
Air traffic	transportation	1226	2408	0.163	[6]	url
Haverford	social	1446	59589	0.009	[15, 16, 17]	url
Simmons	social	1510	32984	0.016	[15, 16, 17]	url
Swarthmore	social	1657	61049	0.009	[15, 16, 17]	url
Petster, hamster	social	1788	12476	0.025	[6]	url
UC Irvine	social	1893	13835	0.023	[21]	url
Yeast, protein	biological	2224	6609	0.071	[22]	url
Amherst	social	2235	90954	0.008	[15, 16, 17]	url
Bowdoin	social	2250	84386	0.009	[15, 16, 17]	url
Hamilton	social	2312	96393	0.008	[15, 16, 17]	url
Adolescent health	social	2539	10455	0.117	[23, 19]	url
Trinity	social	2613	111996	0.008	[15, 16, 17]	url

Table 1: Information of the networks analyzed in the study. From left to right we report the name of the network, the type of the network, number of nodes in the giant component, number of edges in the giant component, percolation threshold of the network, references to studies where the network is presented and analyzed, and url where the network can be found.

**Network**	**Type**	**N**	**E**	$\mathbf{p_c}$	**Ref.**	**url**
USFCA	social	2672	65244	0.011	[15, 16, 17]	url
Japanese	information	2698	7995	0.030	[3]	url
Williams	social	2788	112985	0.008	[15, 16, 17]	url
Open flights	transportation	2905	15645	0.020	[24, 6]	url
Oberlin	social	2920	89912	0.010	[15, 16, 17]	url
Smith	social	2970	97133	0.010	[15, 16, 17]	url
Wellesley	social	2970	94899	0.010	[15, 16, 17]	url
Vassar	social	3068	119161	0.009	[15, 16, 17]	url
Middlebury	social	3069	124607	0.008	[15, 16, 17]	url
Pepperdine	social	3440	152003	0.007	[15, 16, 17]	url
Colgate	social	3482	155043	0.008	[15, 16, 17]	url
Santa	social	3578	151747	0.007	[15, 16, 17]	url
Wesleyan	social	3591	138034	0.009	[15, 16, 17]	url
Mich	social	3745	81901	0.011	[15, 16, 17]	url
Bitcoin Alpha	social	3775	14120	0.027	[25, 26, 27]	url
Bucknell	social	3824	158863	0.008	[15, 16, 17]	url
Brandeis	social	3887	137561	0.008	[15, 16, 17]	url
Howard	social	4047	204850	0.006	[15, 16, 17]	url
Rice	social	4083	184826	0.007	[15, 16, 17]	url
GR-QC, 1993-2003	social	4158	13422	0.091	[28, 27]	url
Tennis	social	4338	81865	0.007	[29]	None
Rochester	social	4561	161403	0.009	[15, 16, 17]	url
US Power grid	technological	4941	6594	0.437	[10]	url
Lehigh	social	5073	198346	0.008	[15, 16, 17]	url
Johns Hopkins	social	5157	186572	0.007	[15, 16, 17]	url
HT09	social	5352	18481	0.025	[12]	url
Wake	social	5366	279186	0.006	[15, 16, 17]	url
Hep-Th, 1995-1999	social	5835	13815	0.108	[30]	url
Bitcoin OTC	social	5875	21489	0.023	[25, 26, 27]	url
Reactome	biological	5973	145778	0.011	[31, 6]	url
Jung	technological	6120	50290	0.009	[32, 6]	url
Gnutella, Aug. 8, 2002	technological	6299	20776	0.046	[33, 28, 27]	url
American	social	6370	217654	0.008	[15, 16, 17]	url
MIT	social	6402	251230	0.006	[15, 16, 17]	url
JDK	technological	6434	53658	0.009	[6]	url
William	social	6472	266378	0.007	[15, 16, 17]	url
AS Oregon	technological	6474	12572	0.036	[34, 27]	url
UChicago	social	6561	208088	0.008	[15, 16, 17]	url
Princeton	social	6575	293307	0.007	[15, 16, 17]	url
Carnegie	social	6621	249959	0.007	[15, 16, 17]	url
Tufts	social	6672	249722	0.008	[15, 16, 17]	url
UC	social	6810	155320	0.010	[15, 16, 17]	url

Table 2: Continuation of Table 1

**Network**	**Type**	**N**	**E**	$\mathbf{p_c}$	**Ref.**	**url**
Wikipedia elections	social	7066	100736	0.008	[35, 36, 27]	url
English	information	7377	44205	0.011	[3]	url
Gnutella, Aug. 9, 2002	technological	8104	26008	0.045	[33, 28, 27]	url
French	information	8308	23832	0.022	[3]	url
Hep-Th, 1993-2003	social	8638	24806	0.072	[28, 27]	url
Gnutella, Aug. 6, 2002	technological	8717	31525	0.065	[33, 28, 27]	url
Gnutella, Aug. 5, 2002	technological	8842	31837	0.056	[33, 28, 27]	url
PGP	social	10680	24316	0.064	[37]	url
Gnutella, Aug. 4, 2002	technological	10876	39994	0.076	[33, 28, 27]	url
Hep-Ph, 1993-2003	social	11204	117619	0.005	[28, 27]	url
Spanish 1	information	11558	43050	0.012	[3]	url
DBLP, citations	information	12495	49563	0.032	[38, 6]	url
Spanish 2	information	12643	55019	0.012	[6]	url
Cond-Mat, 1995-1999	social	13861	44619	0.064	[30, 27]	url
Astrophysics	social	14845	119652	0.018	[30]	url
AstroPhys, 1993-2003	social	17903	196972	0.013	[28, 27]	url
Marvel	social	19365	96616	0.019	[39, 19]	url
Cond-Mat, 1993-2003	social	21363	91286	0.037	[28, 27]	url
Gnutella, Aug. 25, 2002	technological	22663	54693	0.115	[33, 28, 27]	url
Internet	technological	22963	48436	0.019	None	url
Thesaurus	information	23132	297094	0.011	[40, 6]	url
Cora	information	23166	89157	0.045	[41, 6]	url
AS Caida	technological	26475	53381	0.021	[34, 27]	url
Gnutella, Aug. 24, 2002	technological	26498	65359	0.106	[33, 28, 27]	url

Table 3: Continuation of Table 1

2 T=0.05

2.1 Relative size of seed set vs relative size of the outbreak

2.1.1 Subcritical Regime

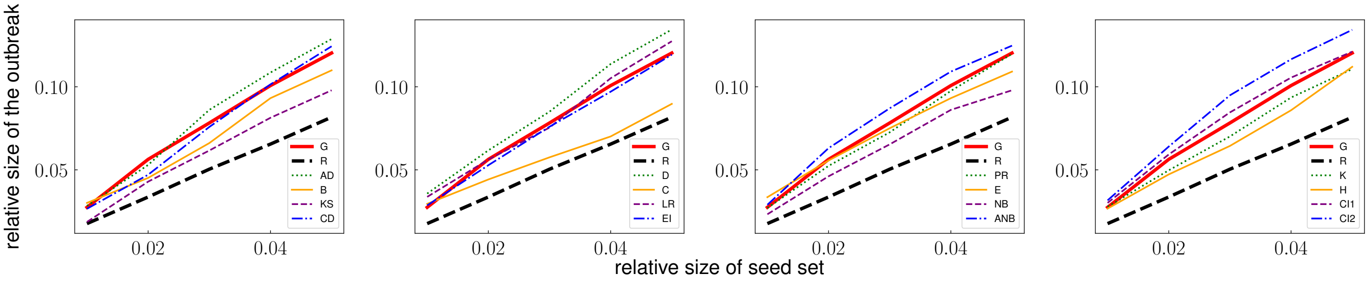

Figure 1: Political books - $p=0.5p_c$

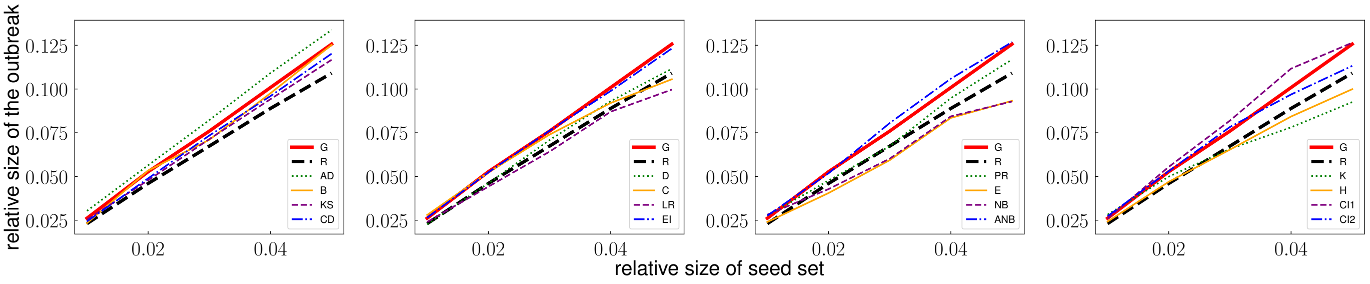

Figure 2: College football - $p=0.5p_c$

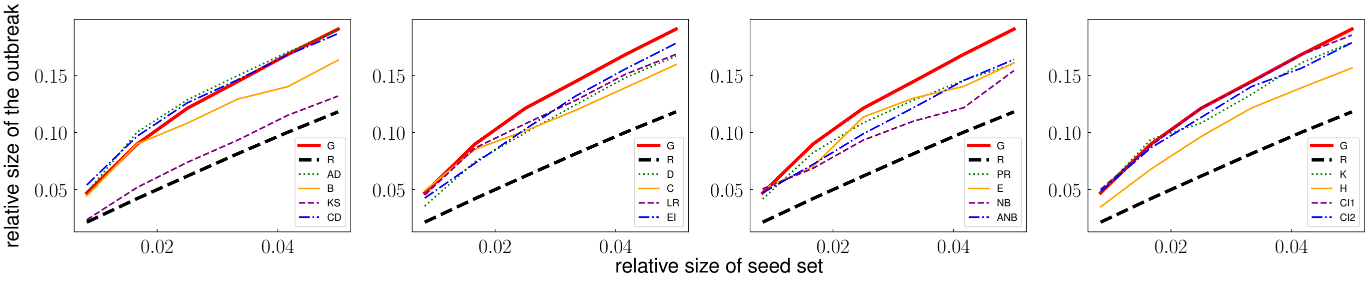

Figure 3: S208 - $p=0.5p_c$

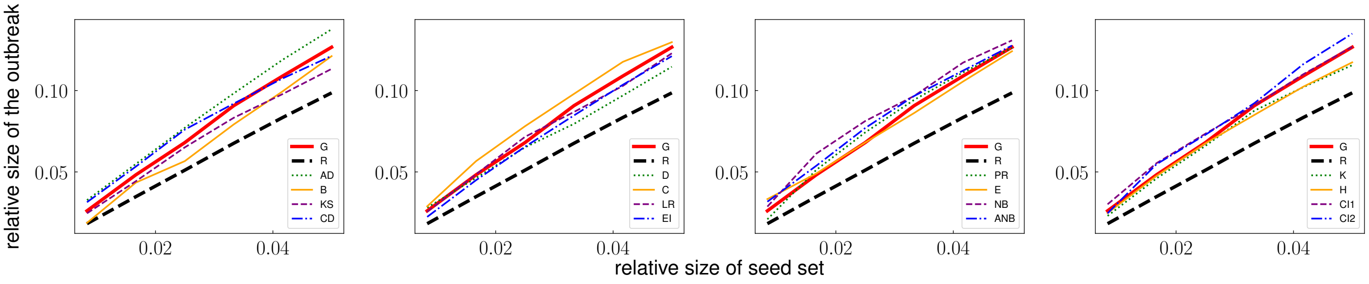

Figure 4: High school, 2011 - p=0.5p_c

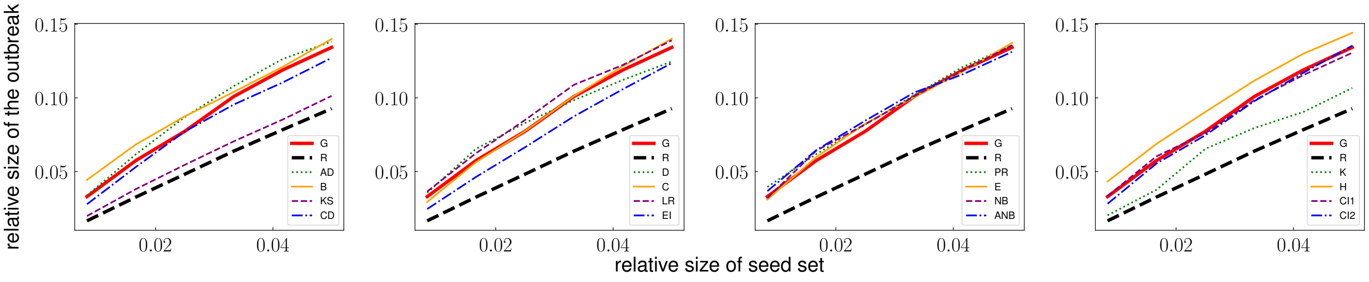

Figure 5: Bay Dry - p=0.5p_c

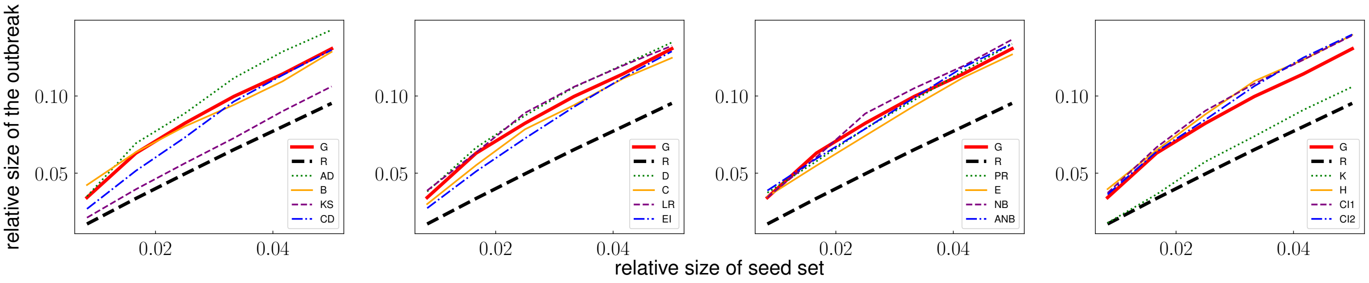

Figure 6: Bay Wet - p=0.5p_c

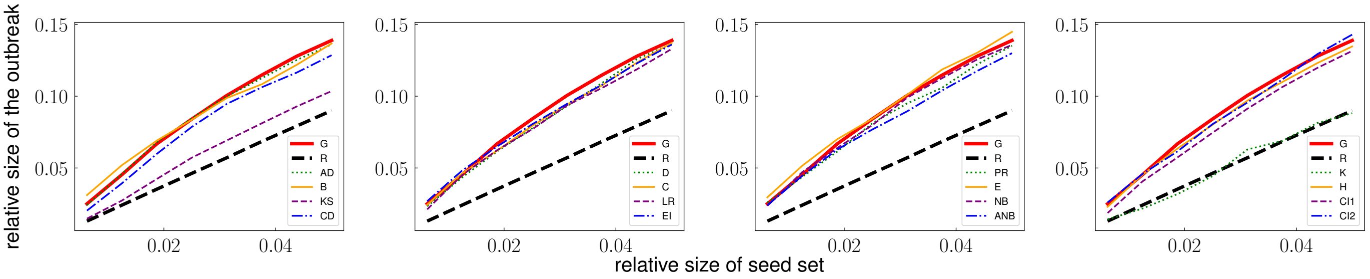

Figure 7: Radoslaw Email - p=0.5p_c

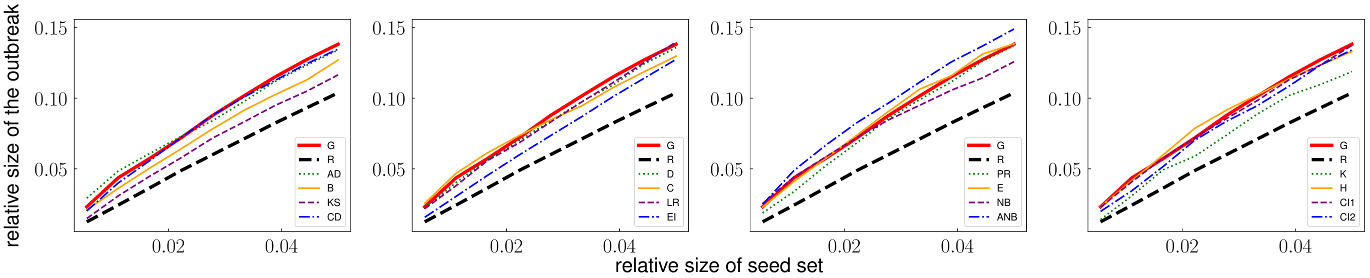

Figure 8: High school, 2012 - p=0.5p_c

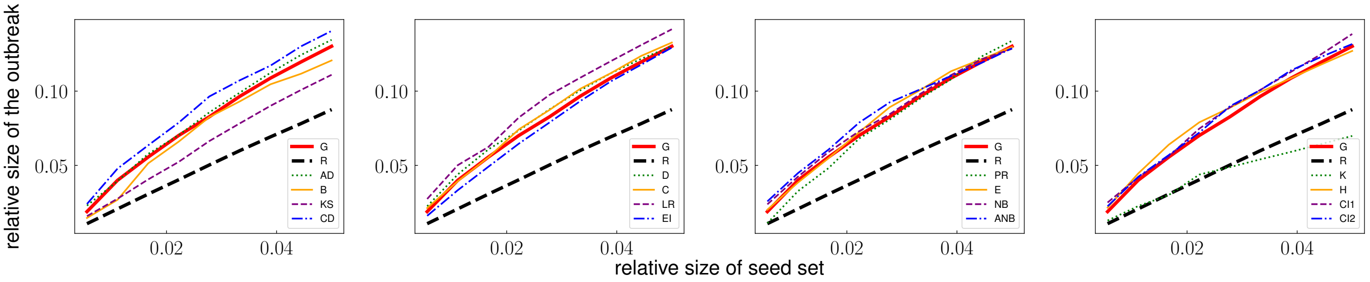

Figure 9: Little Rock Lake - p=0.5p_c

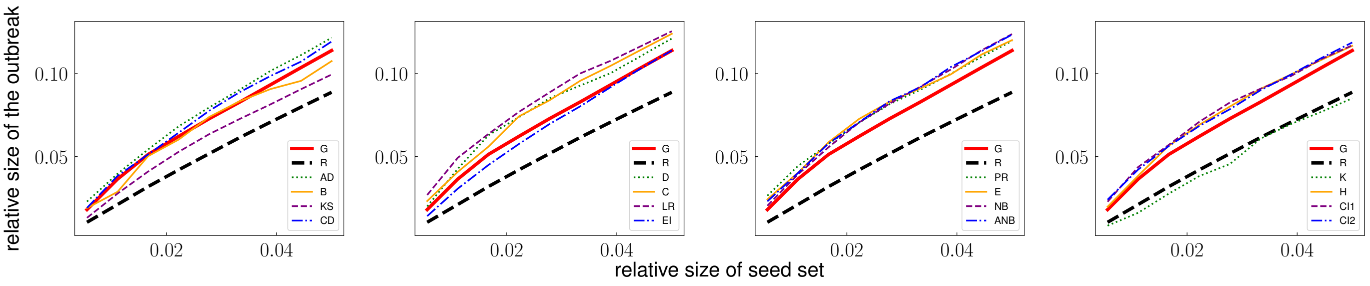

Figure 10: Jazz - p=0.5p_c

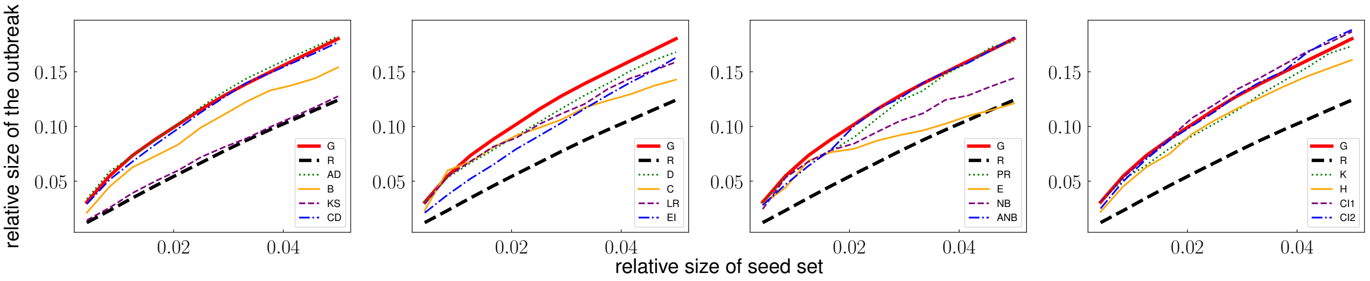

Figure 11: S420 - p=0.5p_c

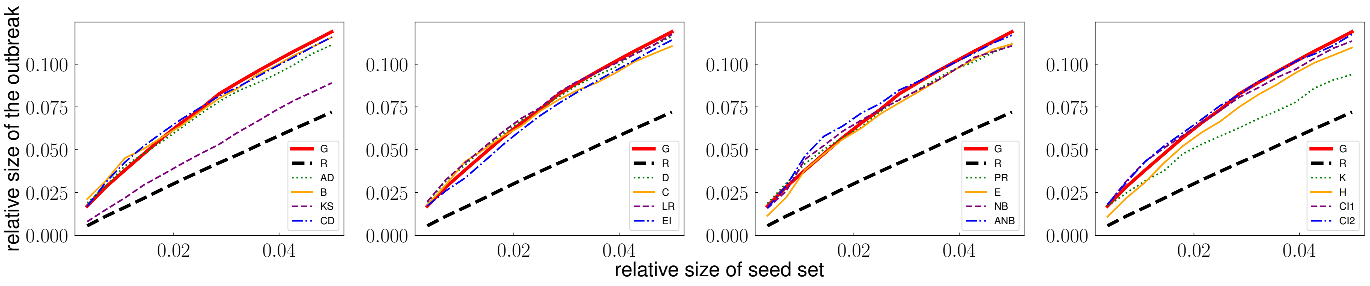

Figure 12: C. Elegans, neural - p=0.5p_c

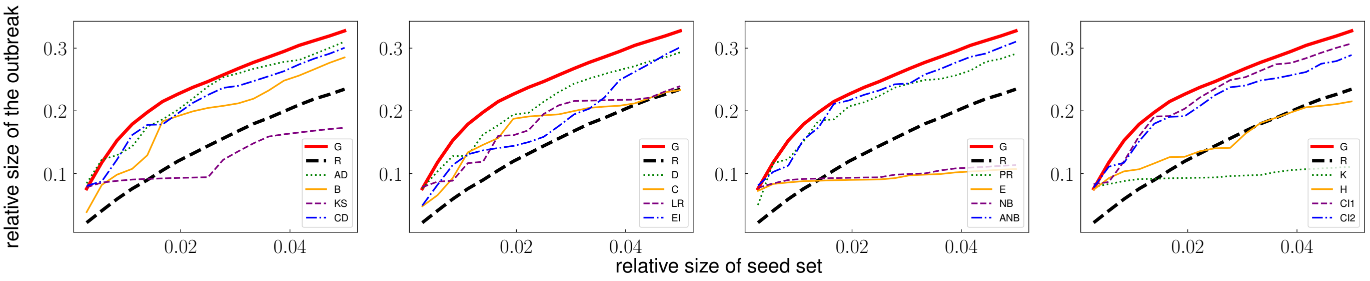

Figure 13: Network Science - p=0.5p_c

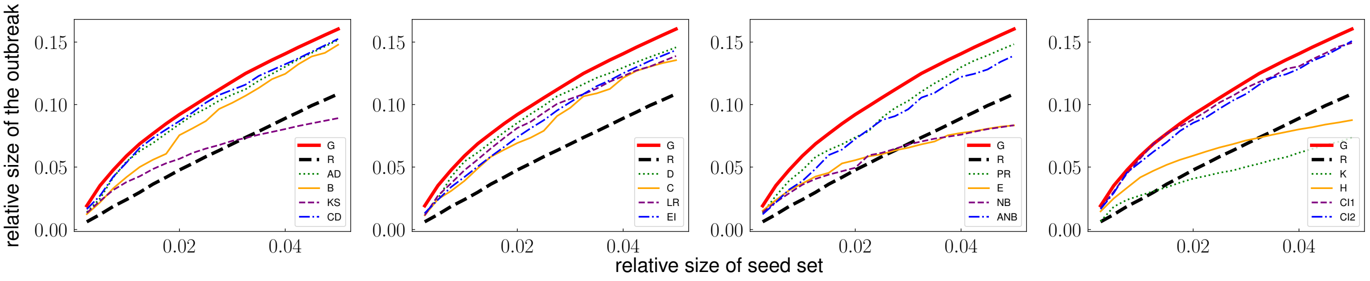

Figure 14: Dublin - p=0.5p_c

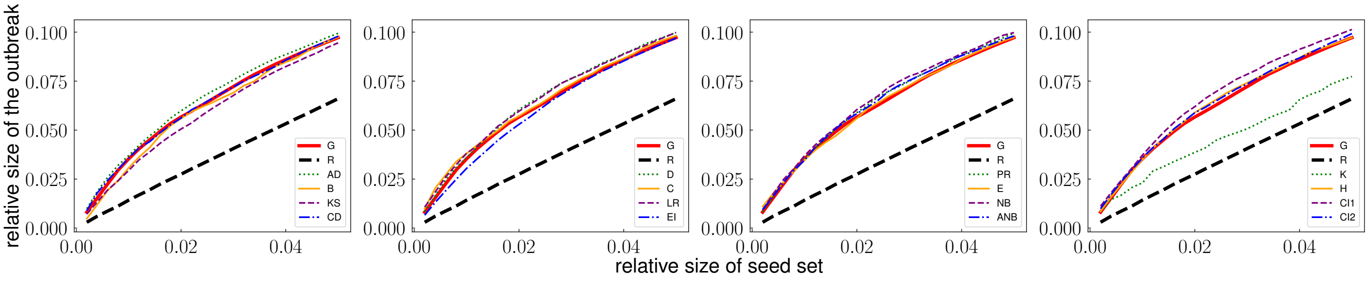

Figure 15: US Air Trasportation - p=0.5p_c

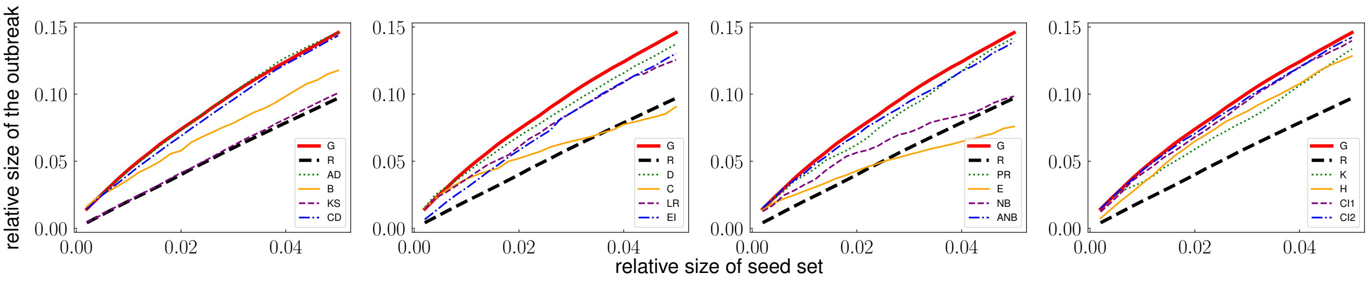

Figure 16: S838 - p=0.5p_c

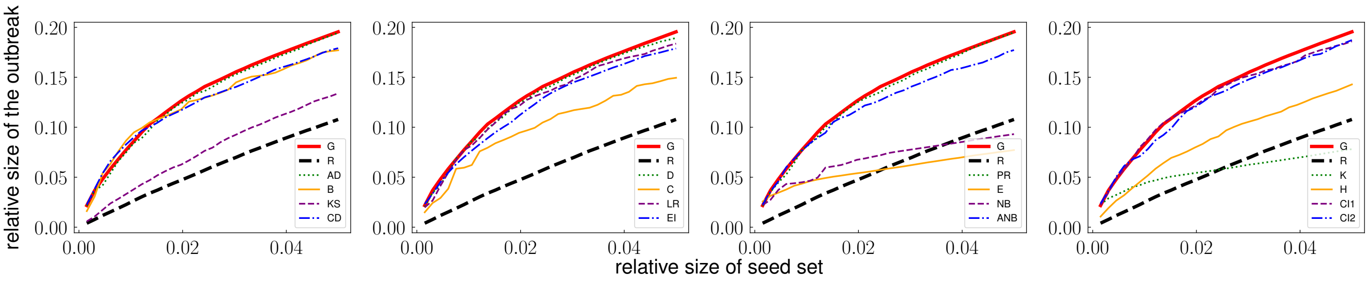

Figure 17: Yeast, transcription - p=0.5p_c

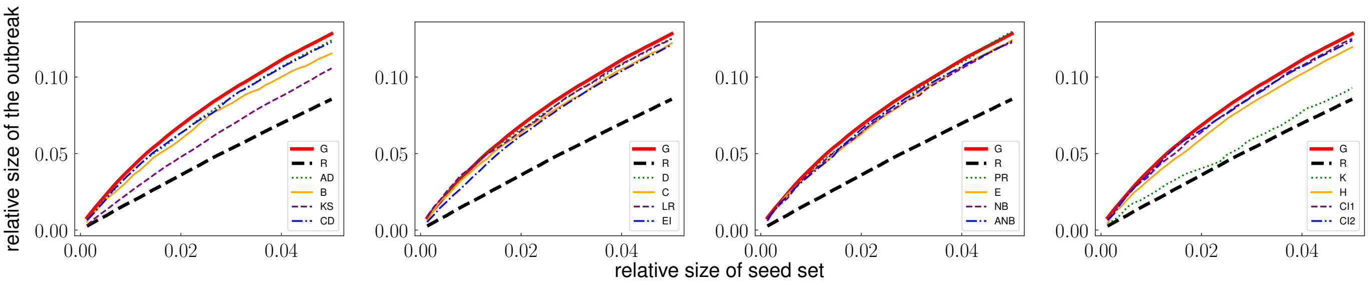

Figure 18: Caltech - p=0.5p_c

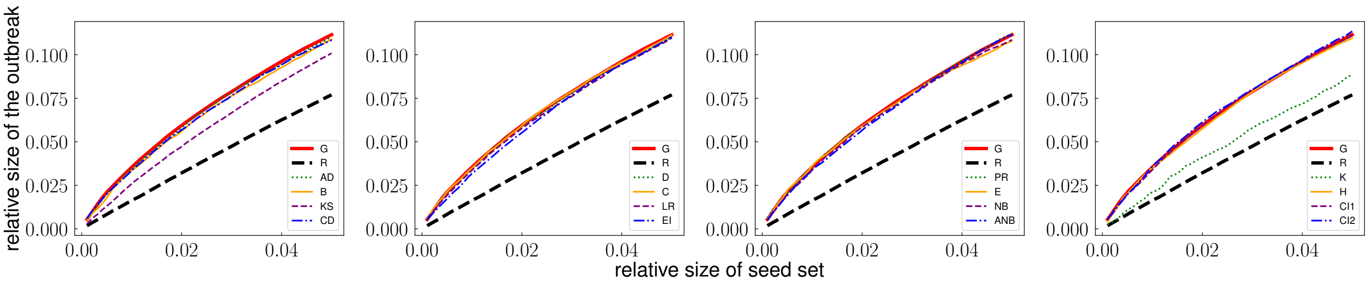

Figure 19: Reed - $p=0.5p_c$

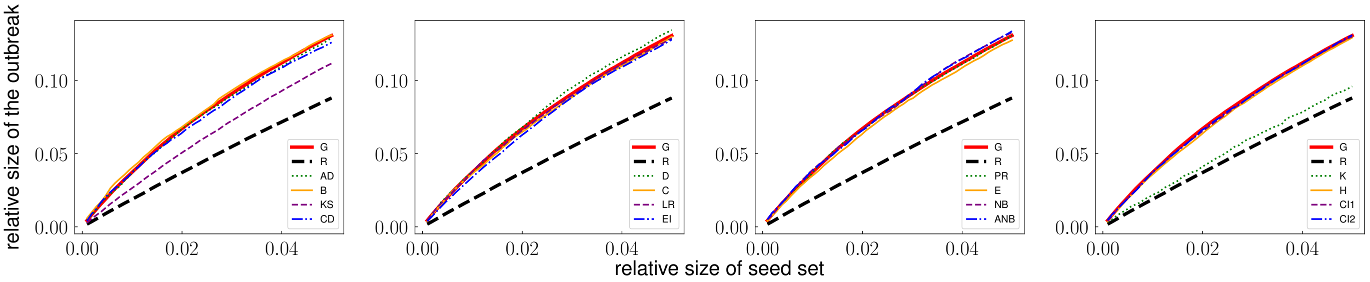

Figure 20: Mouse retina - $p=0.5p_c$

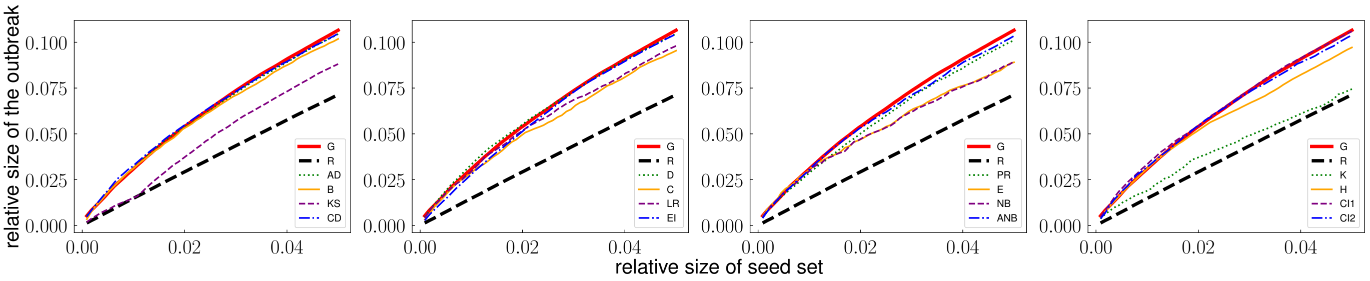

Figure 21: URV email - $p=0.5p_c$

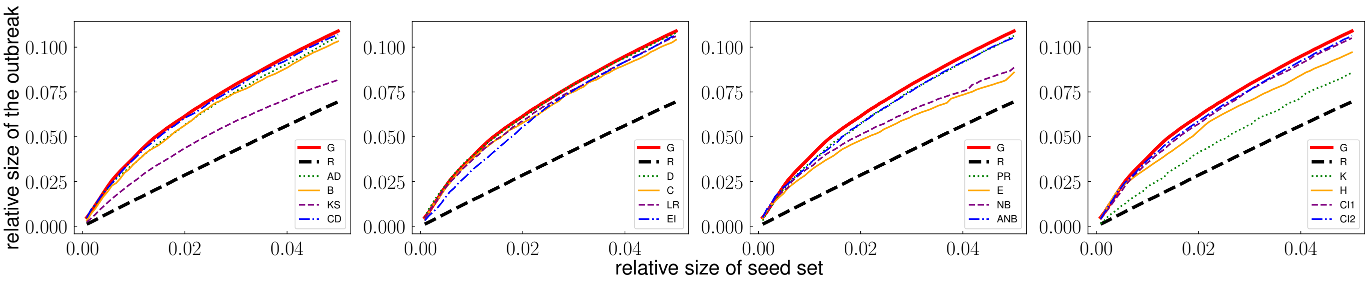

Figure 22: Political blogs - p=0.5p_c

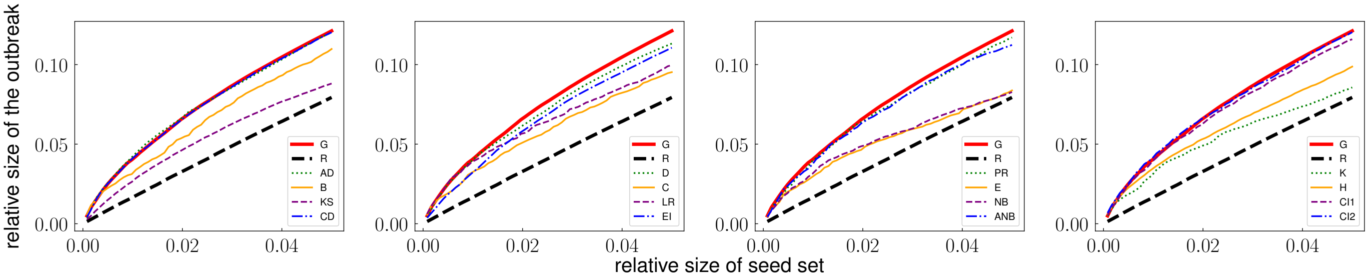

Figure 23: Air traffic - p=0.5p_c

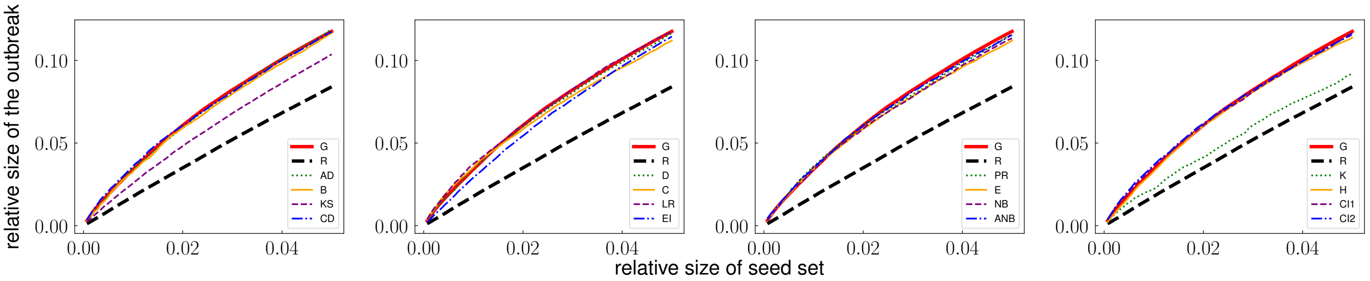

Figure 24: Haverford - p=0.5p_c

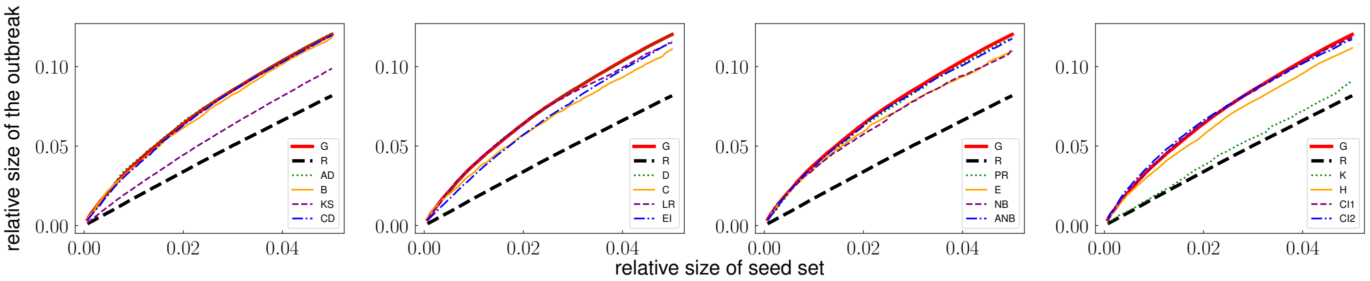

Figure 25: Simmons - $p=0.5p_c$

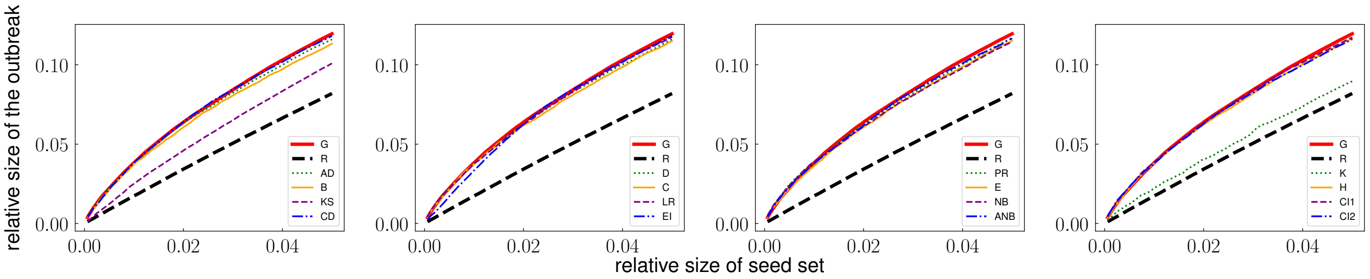

Figure 26: Swarthmore - $p=0.5p_c$

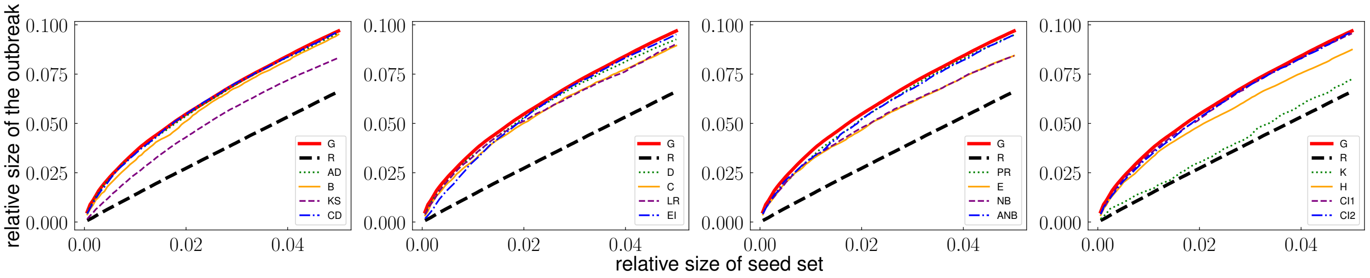

Figure 27: Petster, hamster - $p=0.5p_c$

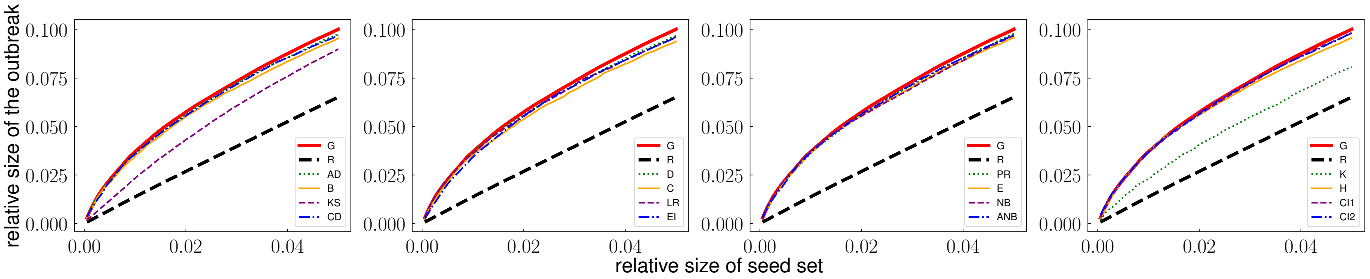

Figure 28: UC Irvine - p=0.5p_c

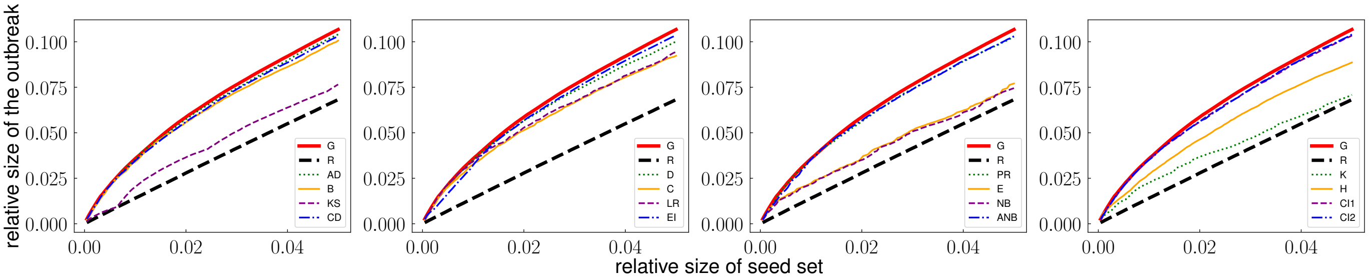

Figure 29: Yeast, protein - p=0.5p_c

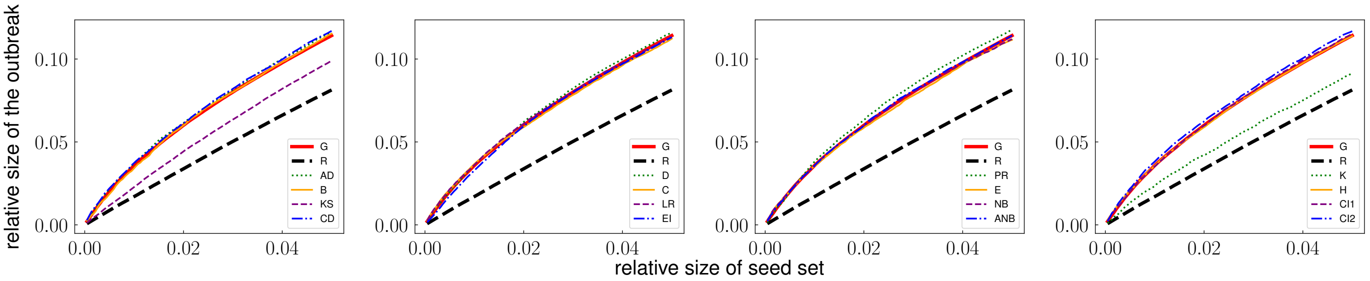

Figure 30: Amherst - p=0.5p_c

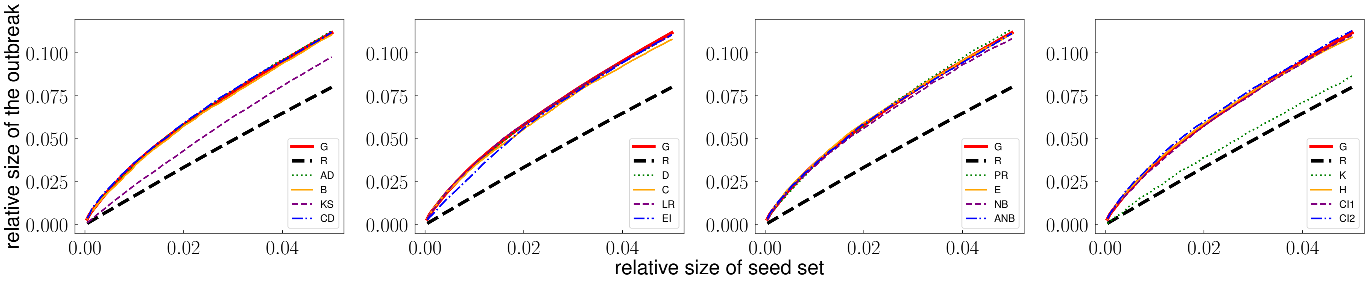

Figure 31: Bowdoin - p=0.5p_c

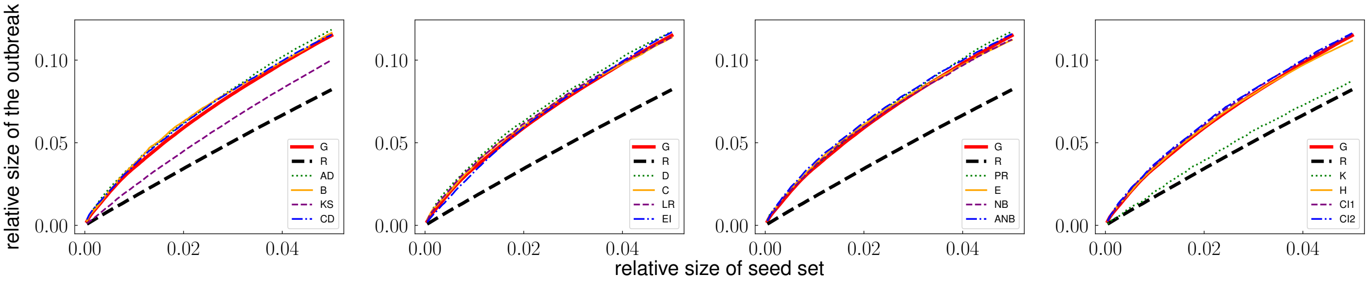

Figure 32: Hamilton - p=0.5p_c

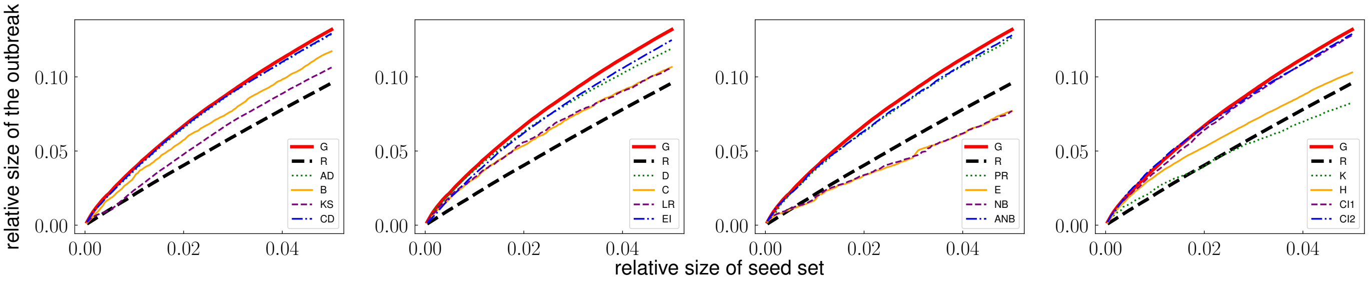

Figure 33: Adolescent health - p=0.5p_c

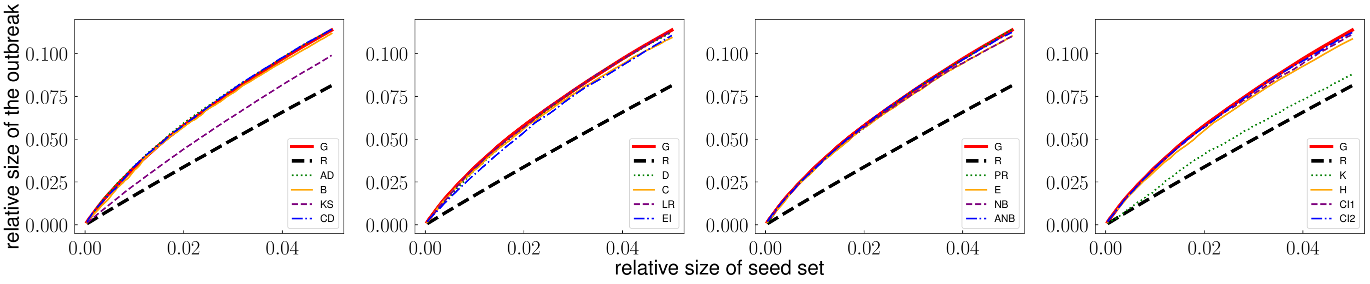

Figure 34: Trinity - $p=0.5p_c$

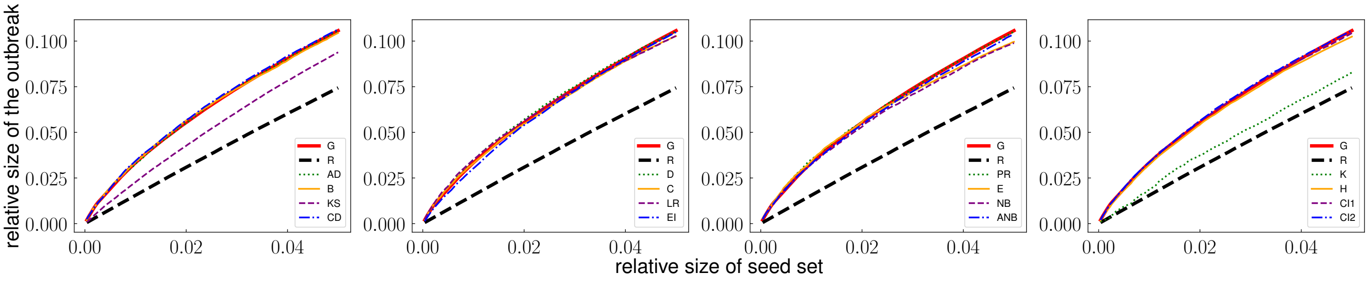

Figure 35: USFCA - $p=0.5p_c$

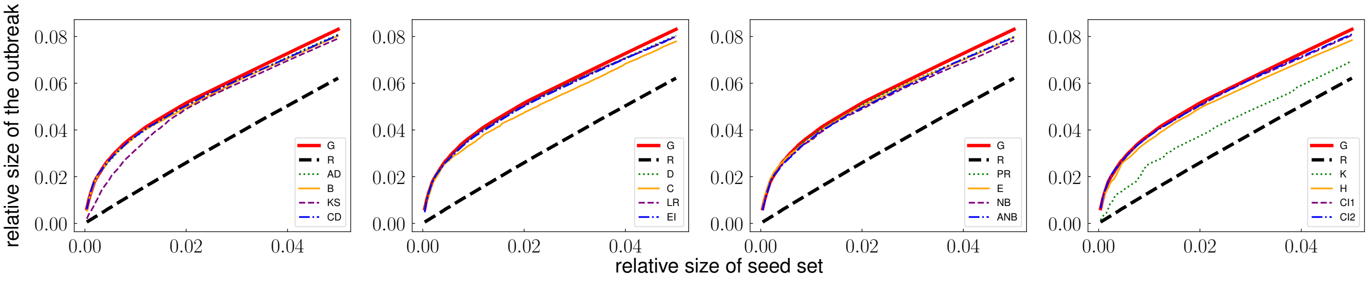

Figure 36: Japanese - $p=0.5p_c$

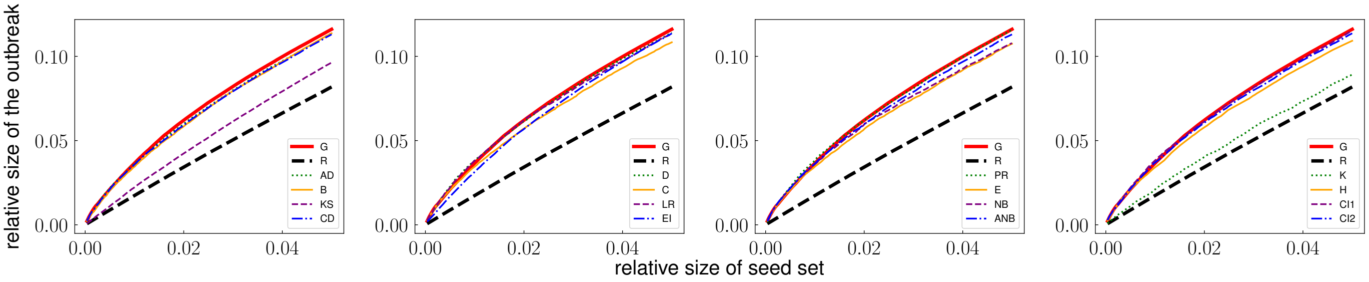

Figure 37: Williams - $p=0.5p_c$

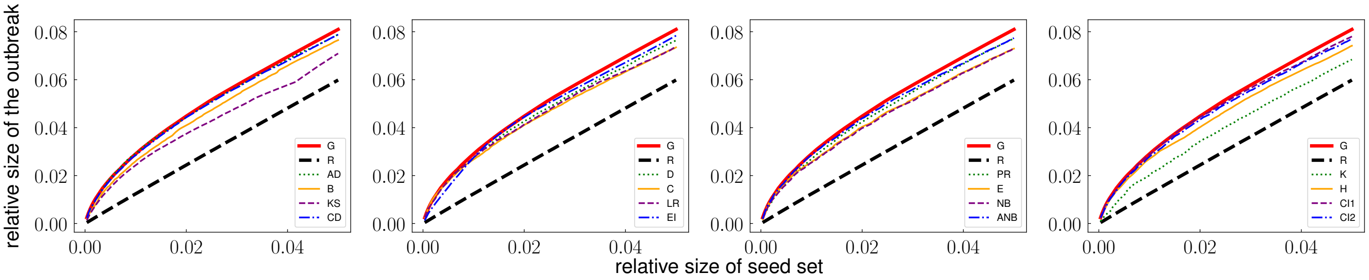

Figure 38: Open flights - $p=0.5p_c$

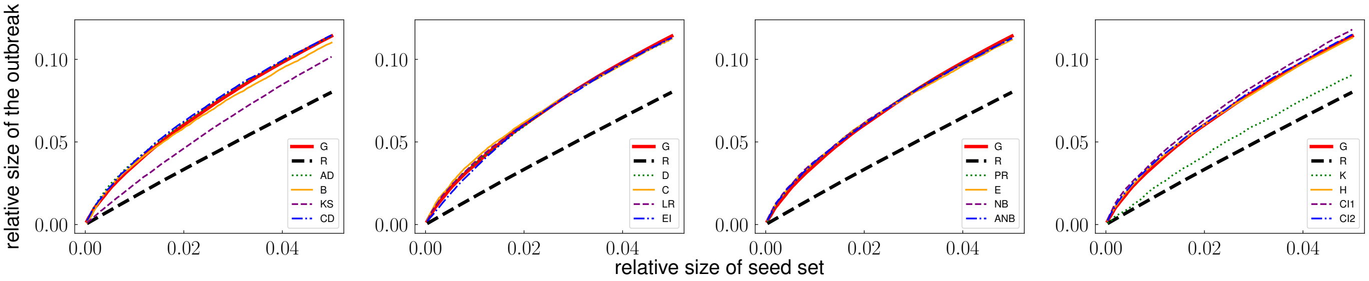

Figure 39: Oberlin - $p=0.5p_c$

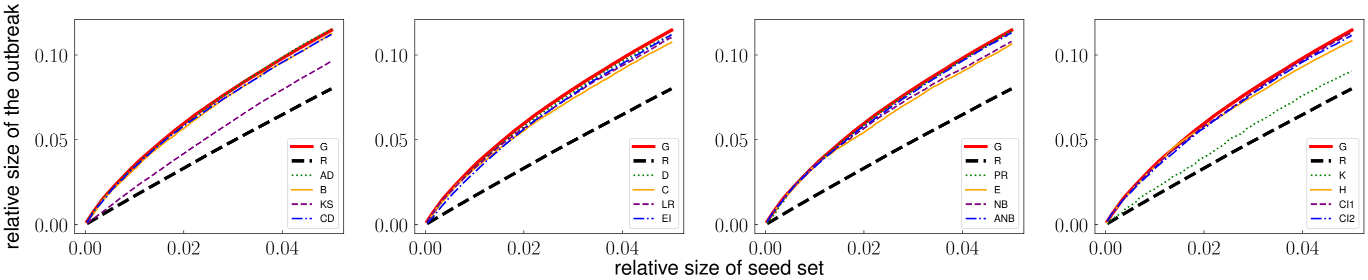

Figure 40: Smith - p=0.5p_c

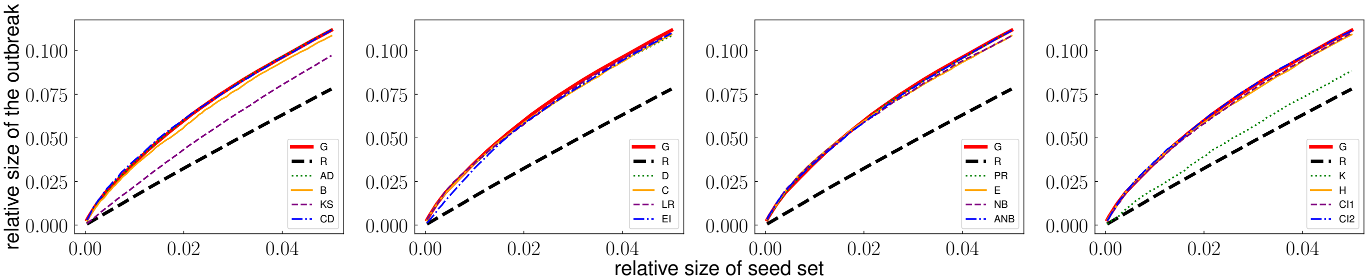

Figure 41: Wellesley - p=0.5p_c

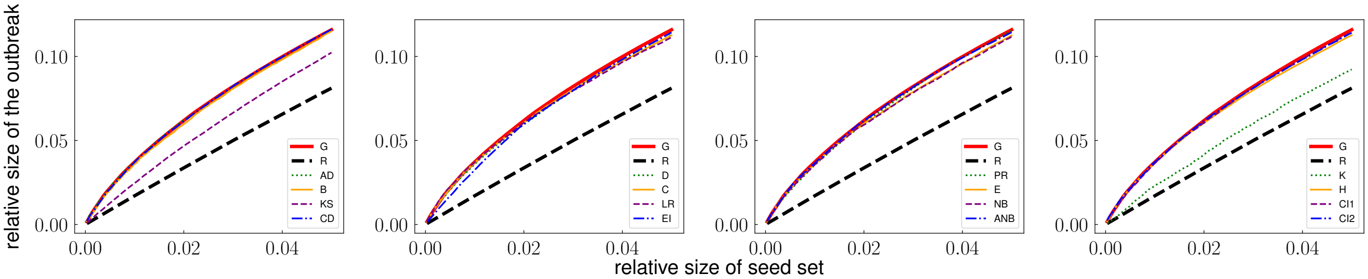

Figure 42: Vassar - p=0.5p_c

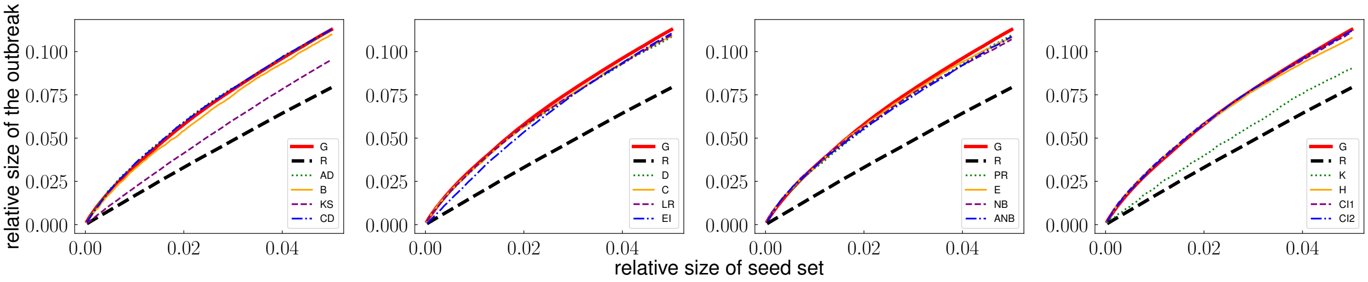

Figure 43: Middlebury - p=0.5p_c

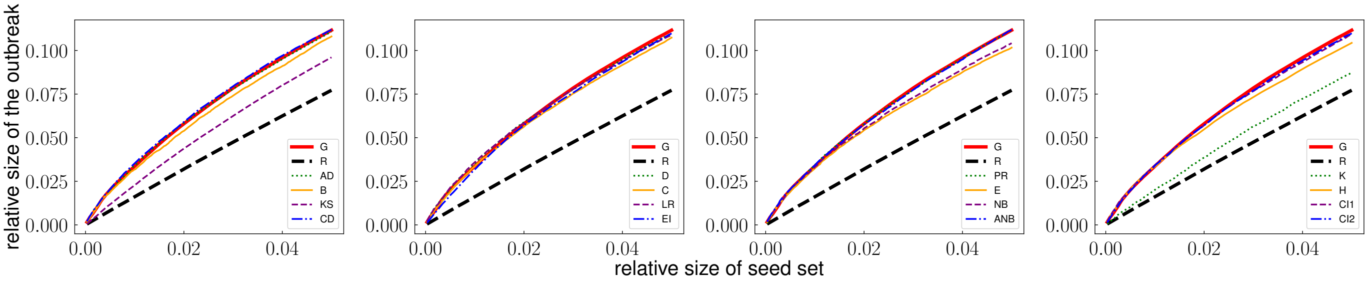

Figure 44: Pepperdine - p=0.5p_c

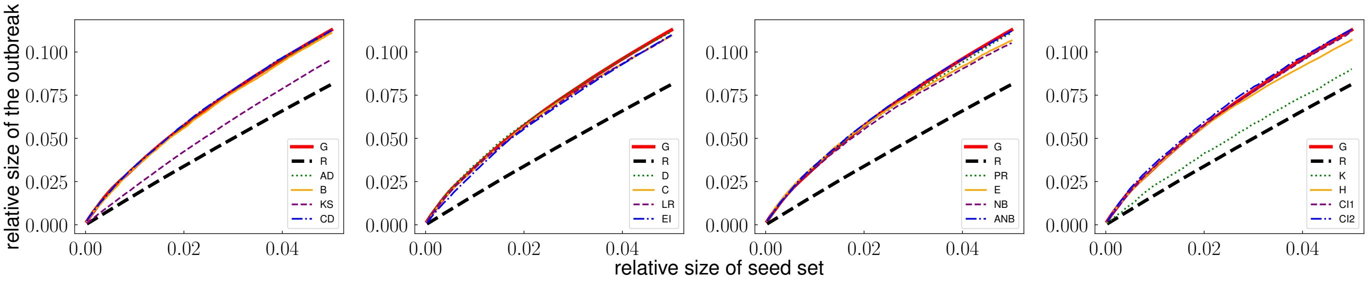

Figure 45: Colgate - p=0.5p_c

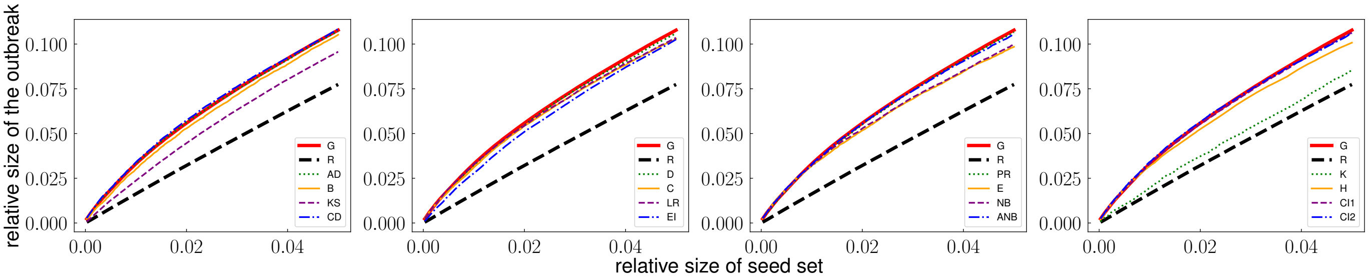

Figure 46: Santa - p=0.5p_c

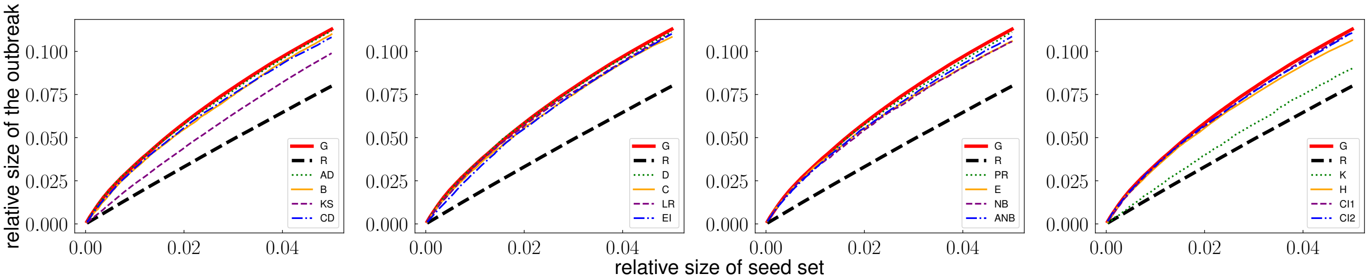

Figure 47: Wesleyan - p=0.5p_c

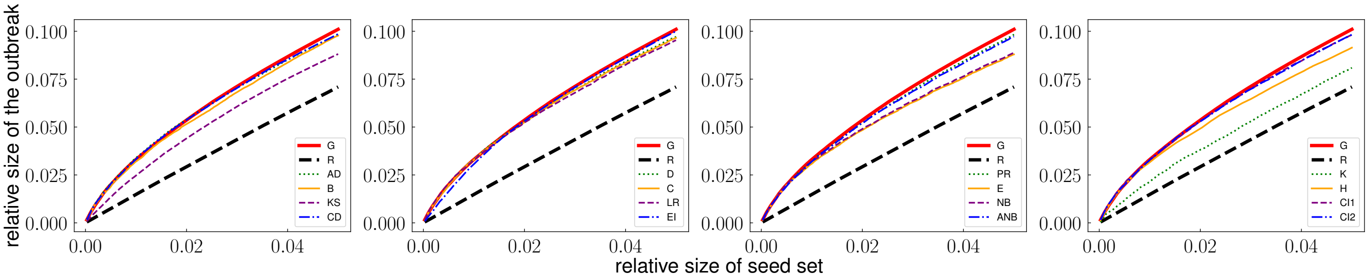

Figure 48: Mich - p=0.5p_c

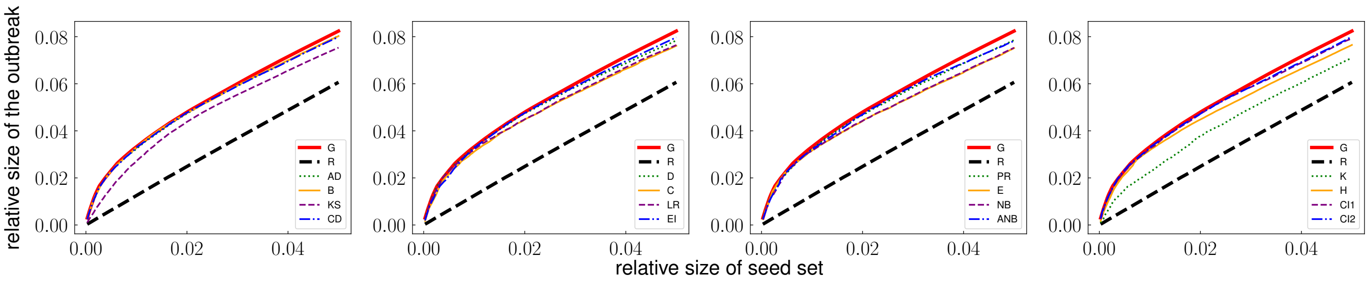

Figure 49: Bitcoin Alpha - p=0.5p_c

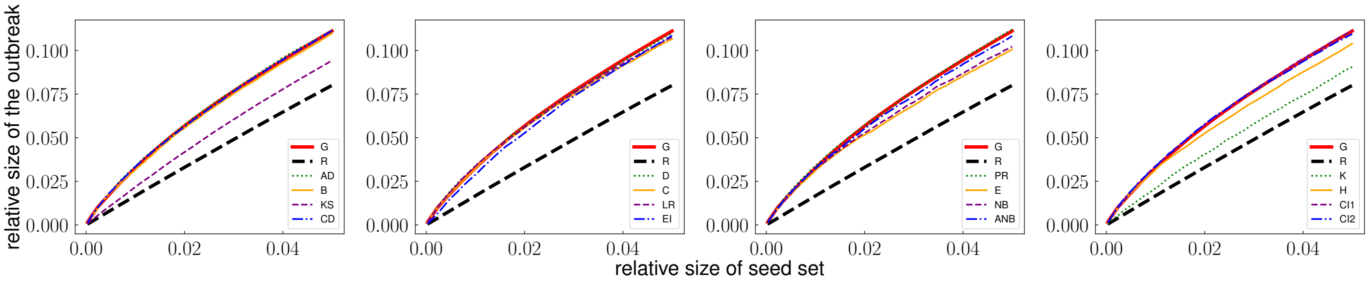

Figure 50: Bucknell - p=0.5p_c

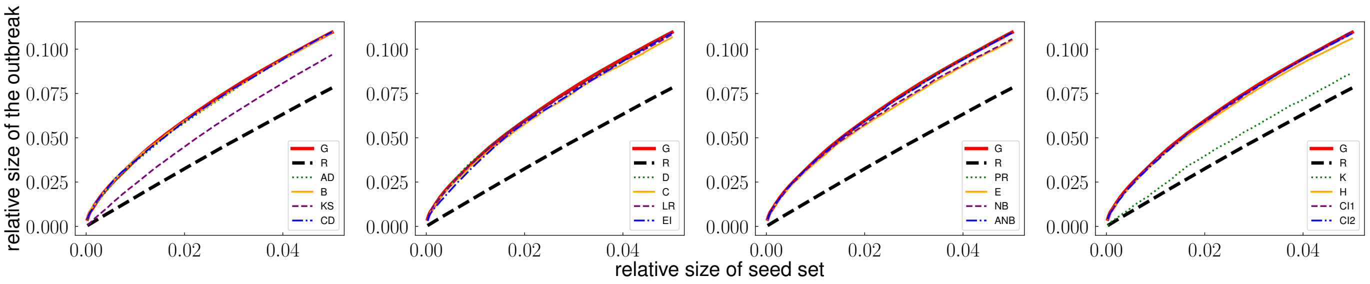

Figure 51: Brandeis - p=0.5p_c

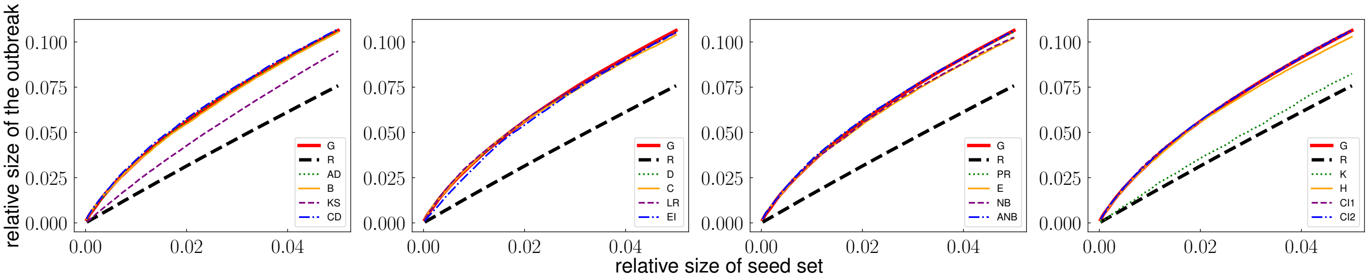

Figure 52: Howard - p=0.5p_c

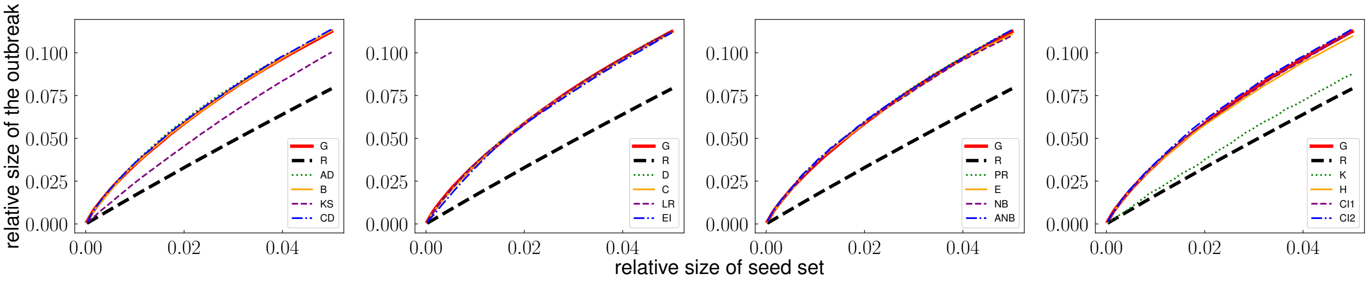

Figure 53: Rice - p=0.5p_c

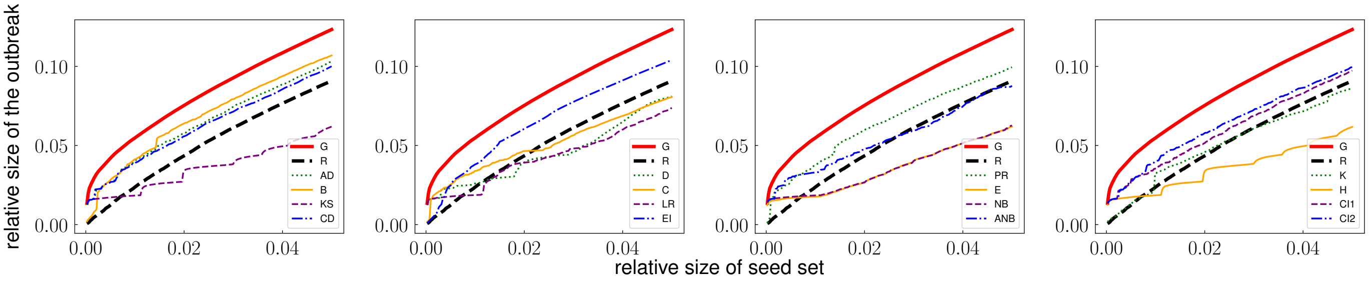

Figure 54: GR-QC, 1993-2003 - p=0.5p_c

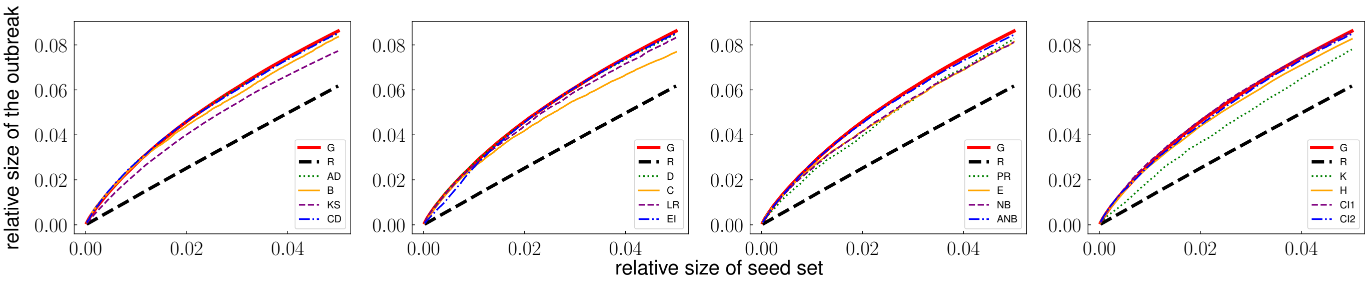

Figure 55: Tennis - p=0.5p_c

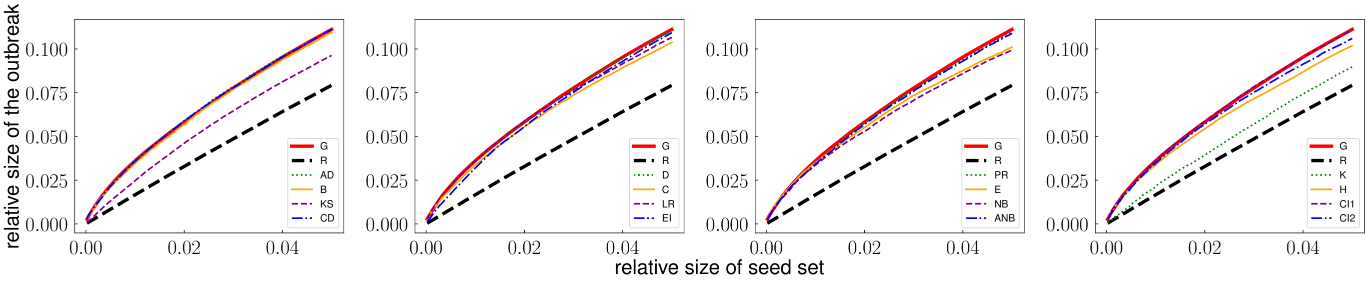

Figure 56: Rochester - p=0.5p_c

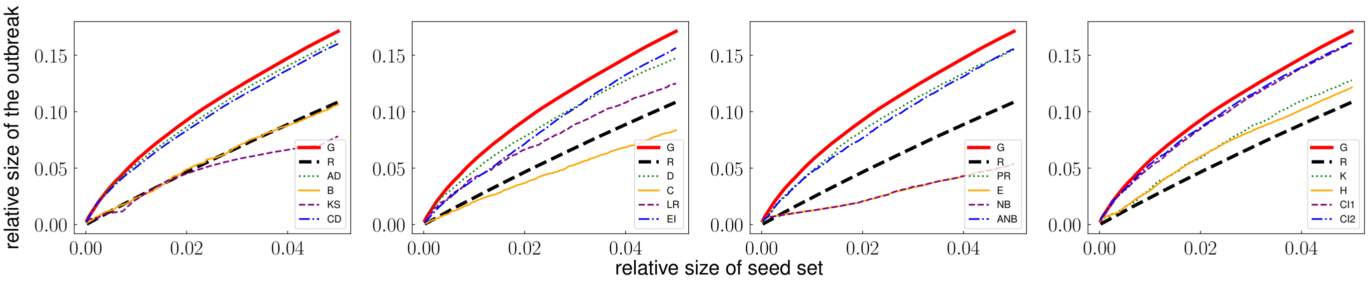

Figure 57: US Power grid - p=0.5p_c

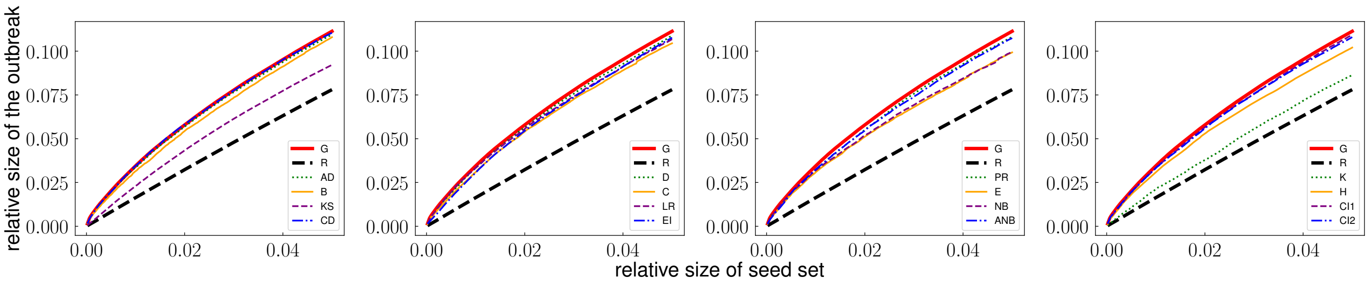

Figure 58: Lehigh - p=0.5p_c

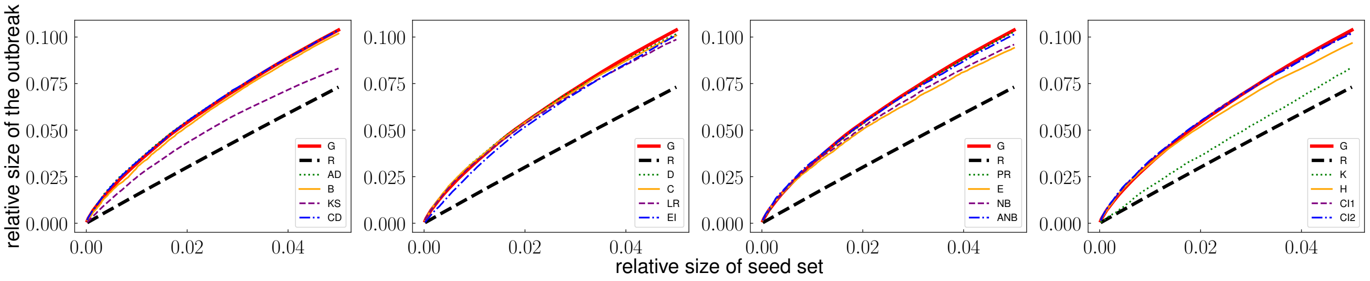

Figure 59: Johns Hopkins - p=0.5p_c

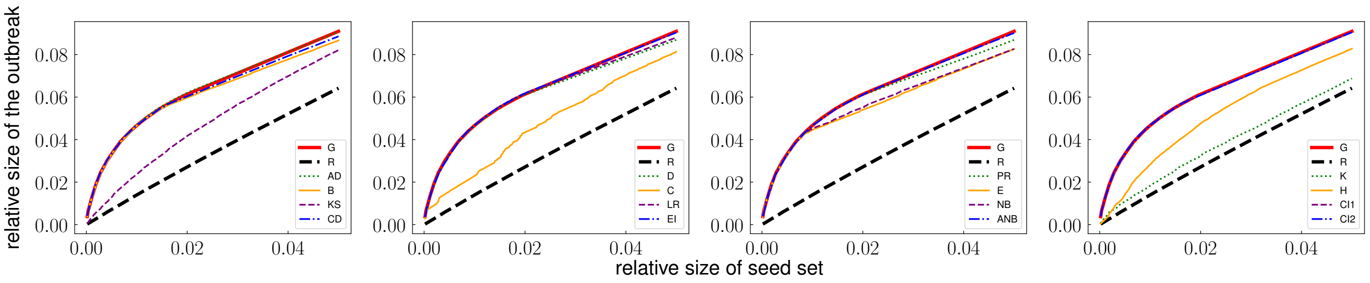

Figure 60: HT09 - p=0.5p_c

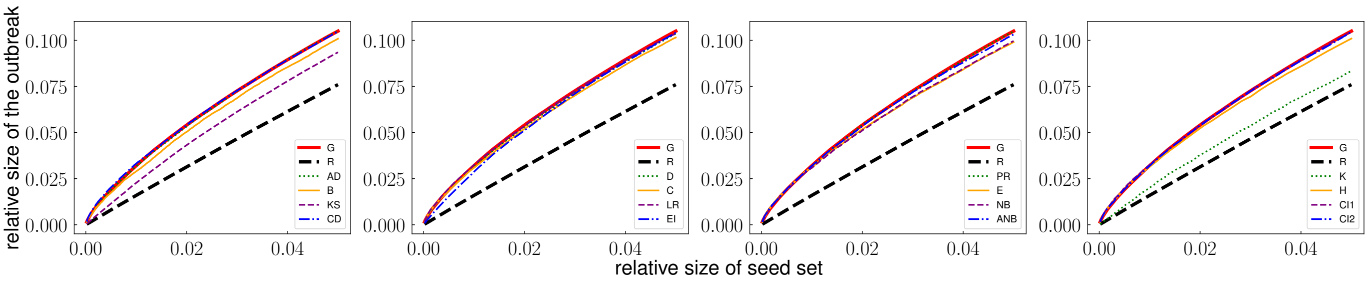

Figure 61: Wake - p=0.5p_c

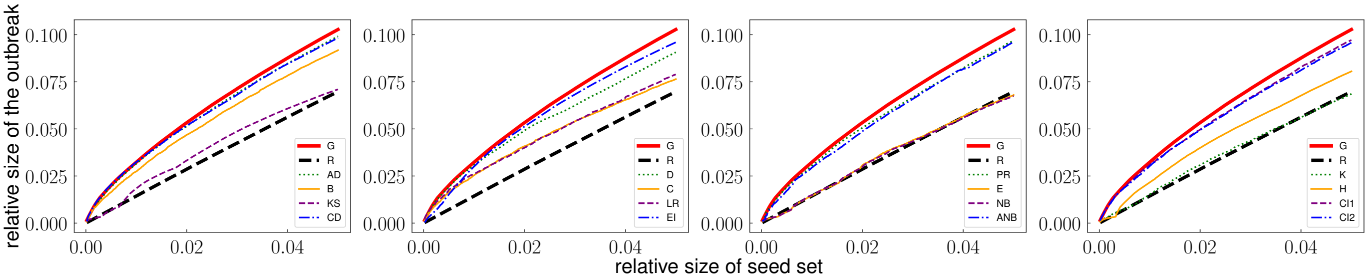

Figure 62: Hep-Th, 1995-1999 - p=0.5p_c

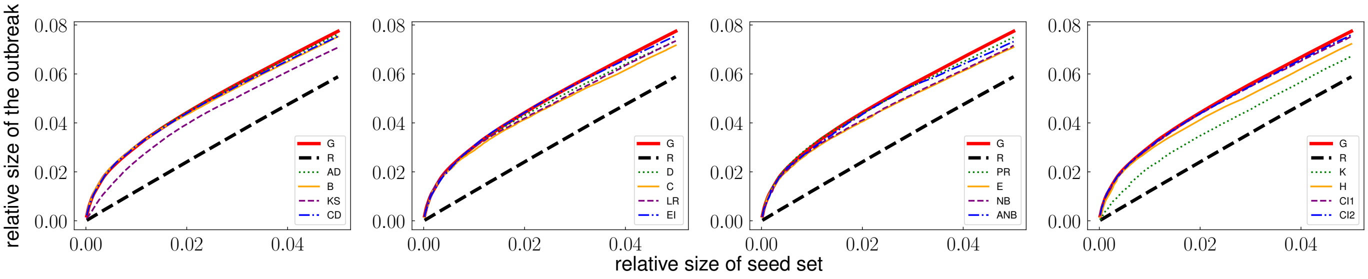

Figure 63: Bitcoin OTC - p=0.5p_c

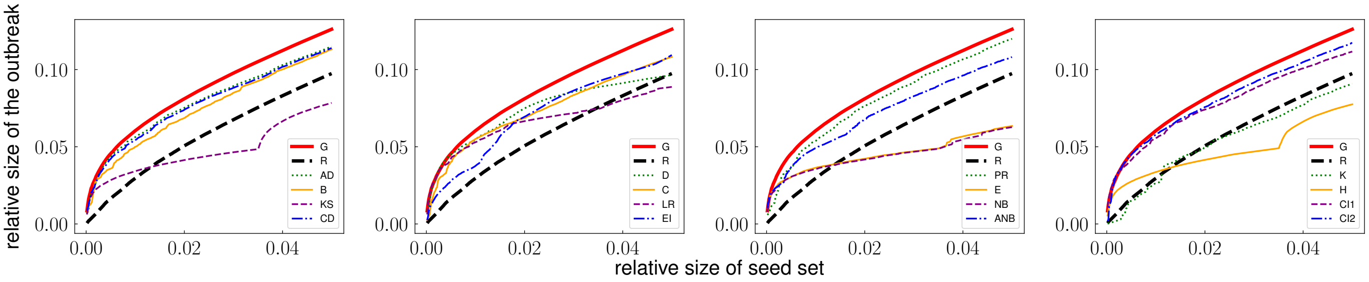

Figure 64: Reactome - p=0.5p$_c$

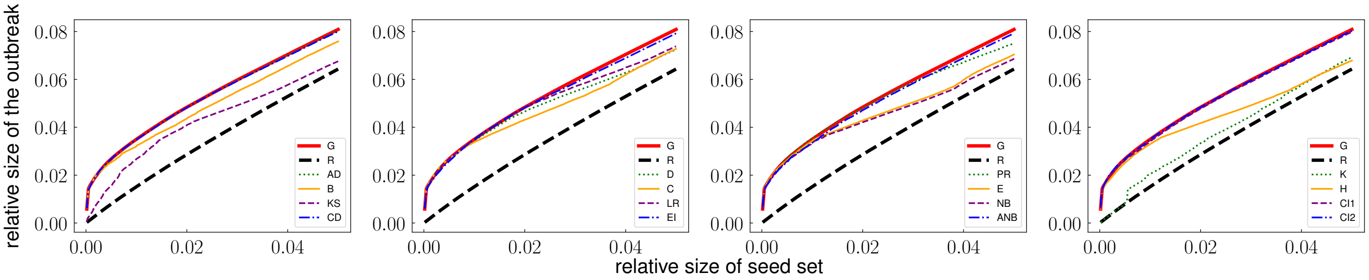

Figure 65: Jung - p=0.5p$_c$

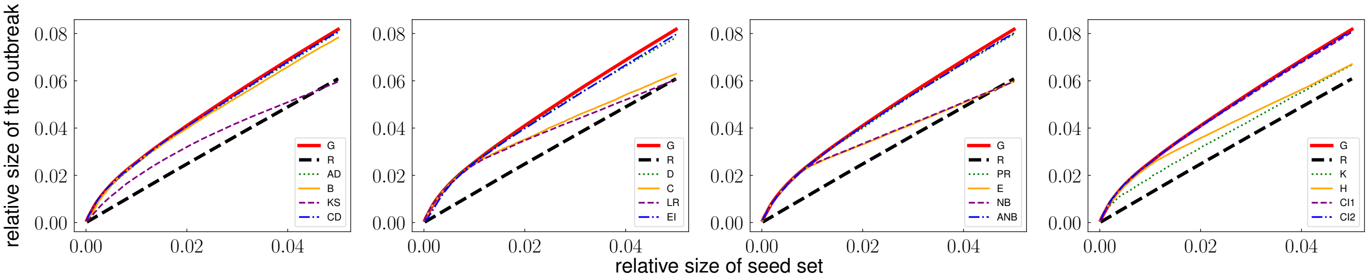

Figure 66: Gnutella, Aug. 8, 2002 - p=0.5p$_c$

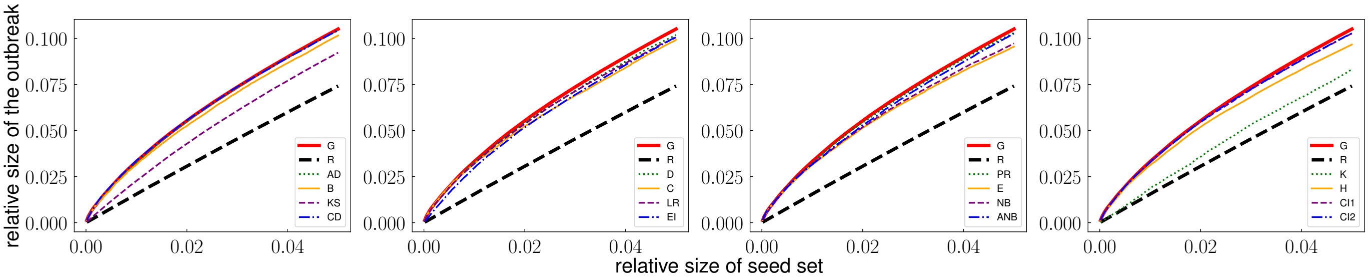

Figure 67: American - $p=0.5p_c$

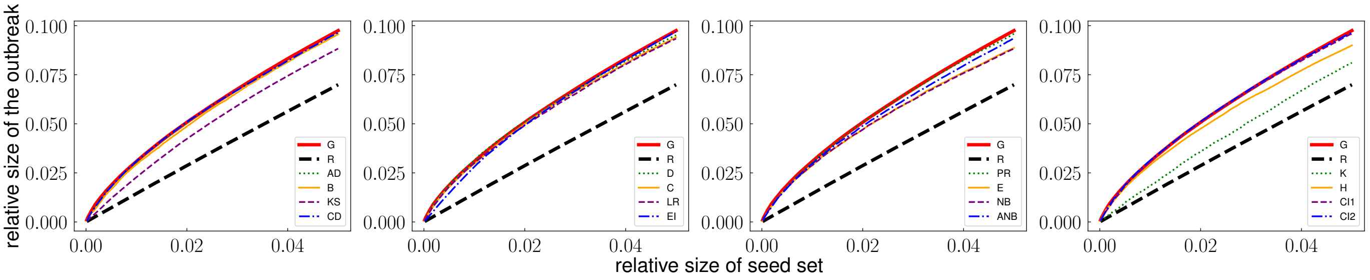

Figure 68: MIT - $p=0.5p_c$

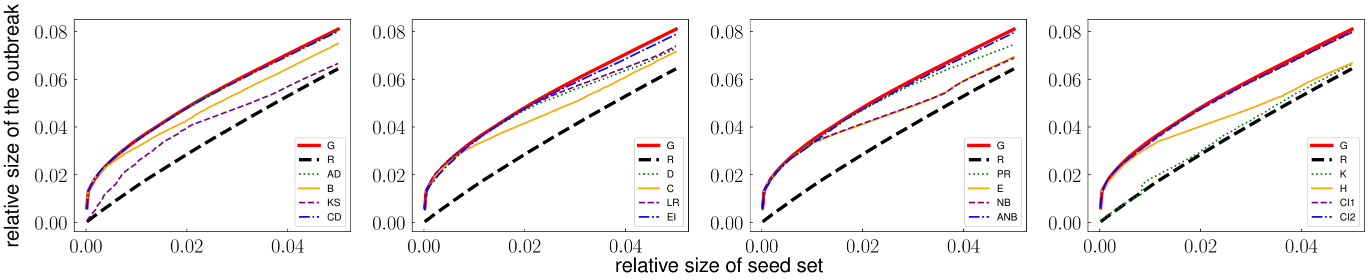

Figure 69: JDK - $p=0.5p_c$

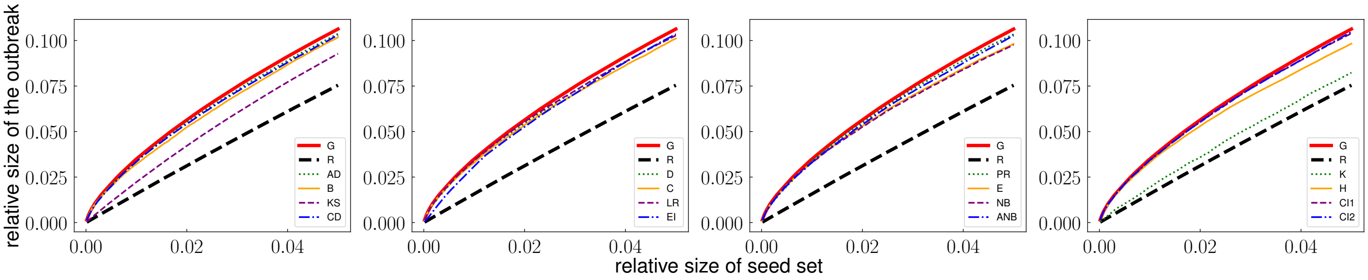

Figure 70: William - p=0.5p_c

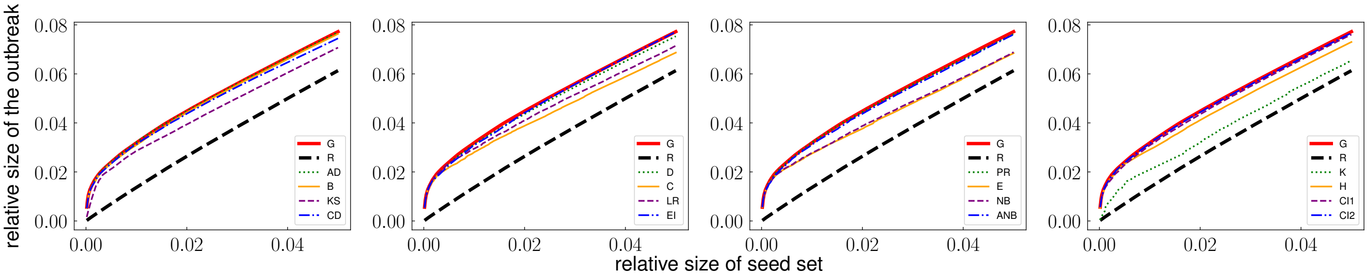

Figure 71: AS Oregon - p=0.5p_c

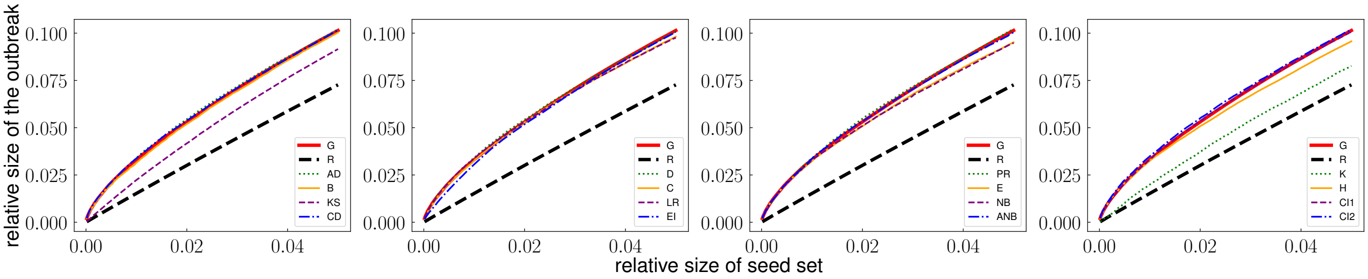

Figure 72: UChicago - p=0.5p_c

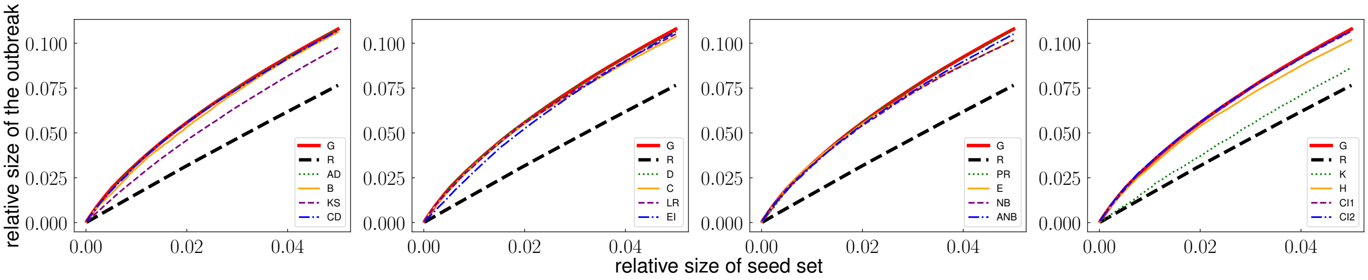

Figure 73: Princeton - p=0.5p_c

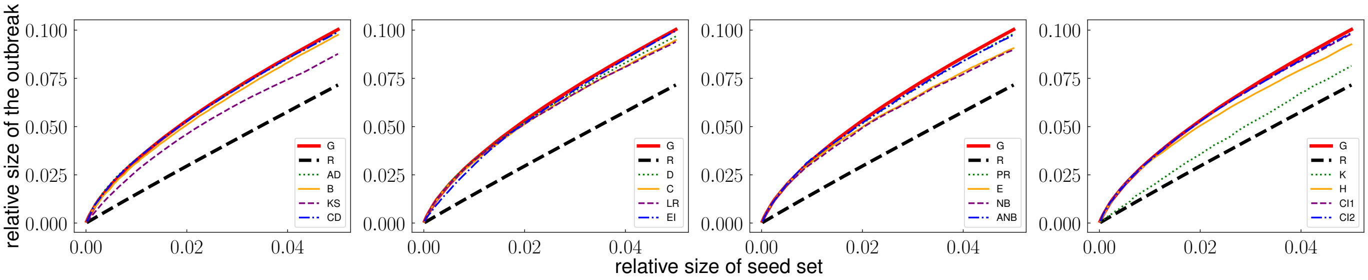

Figure 74: Carnegie - p=0.5p_c

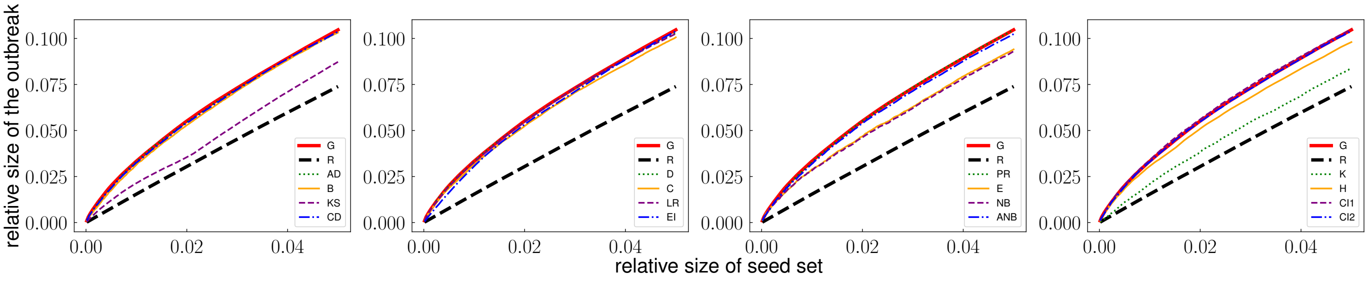

Figure 75: Tufts - p=0.5p_c

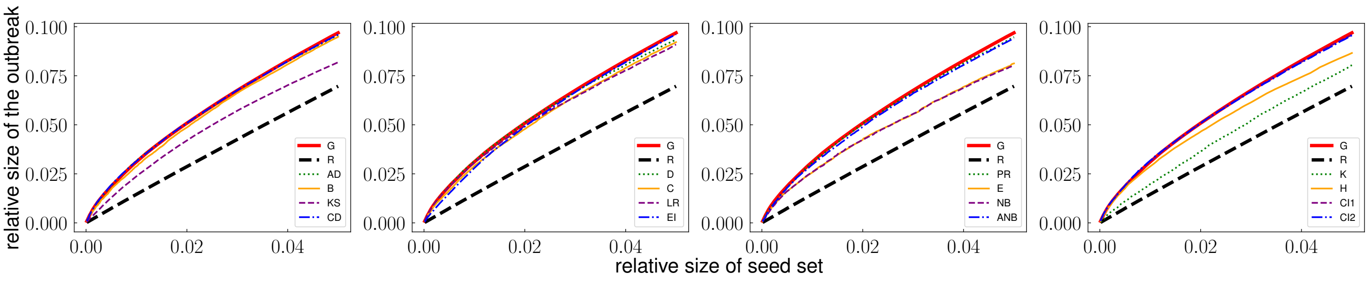

Figure 76: UC - p=0.5p_c

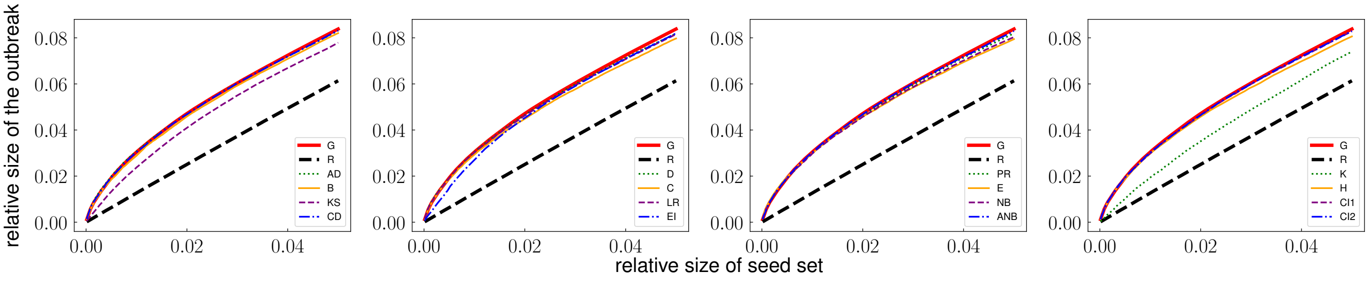

Figure 77: Wikipedia elections - p=0.5p_c

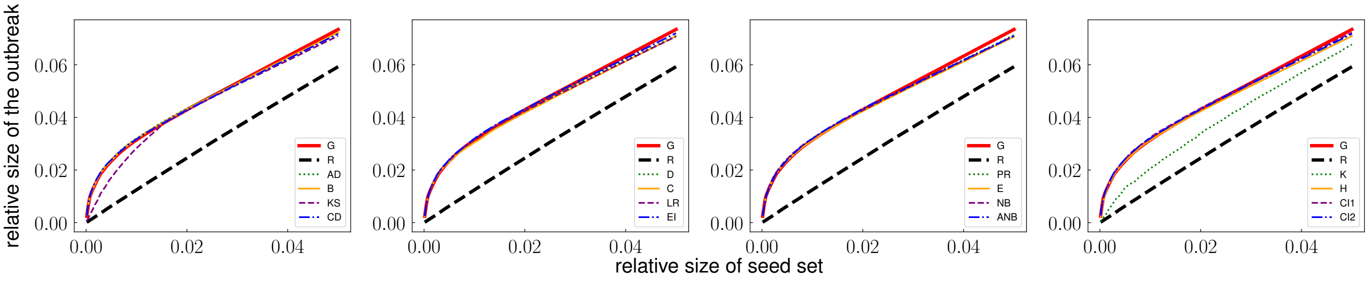

Figure 78: English - p=0.5p_c

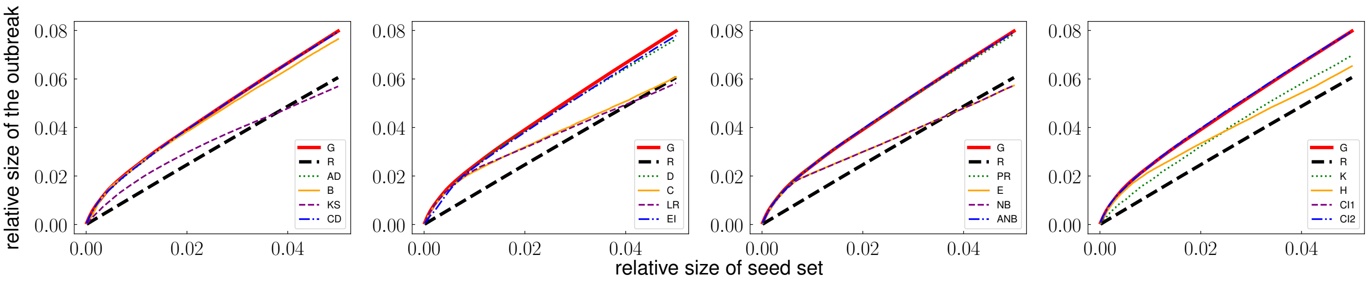

Figure 79: Gnutella, Aug. 9, 2002 - p=0.5p_c

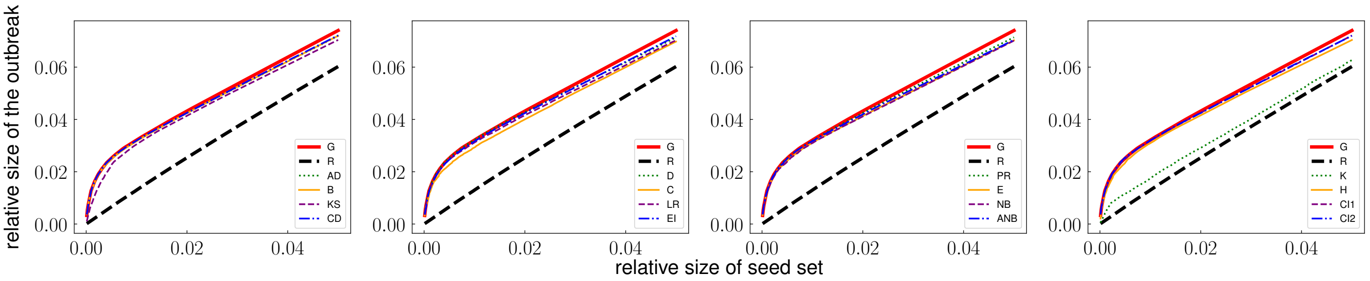

Figure 80: French - p=0.5p_c

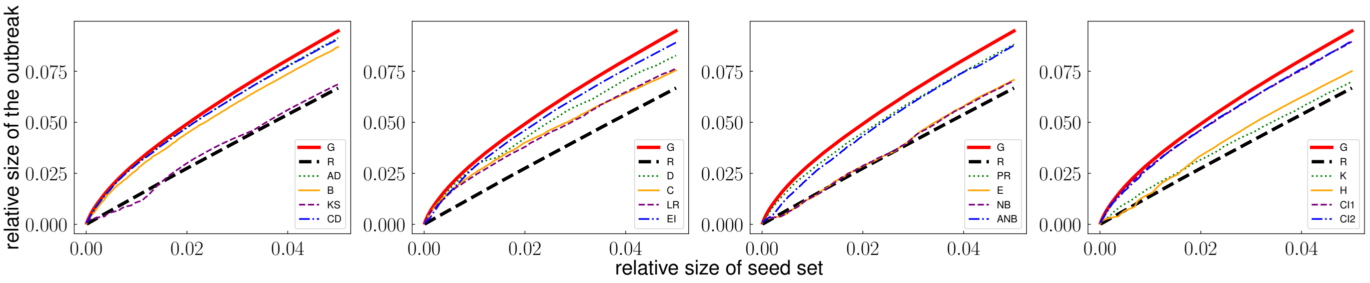

Figure 81: Hep-Th, 1993-2003 - p=0.5p_c

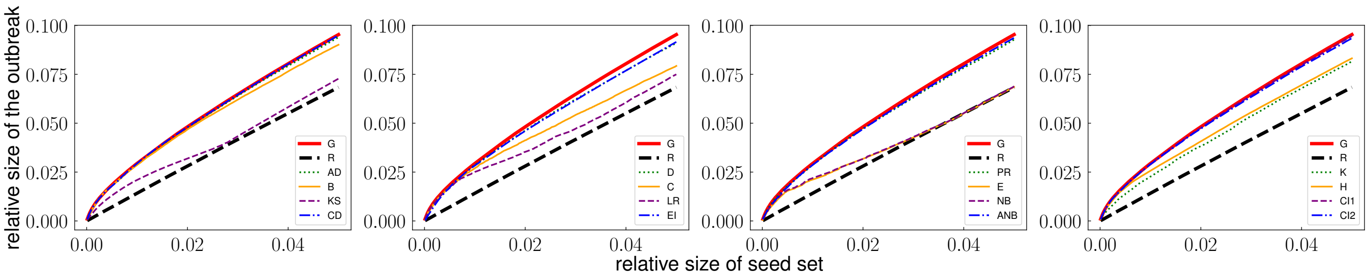

Figure 82: Gnutella, Aug. 6, 2002 - p=0.5p_c

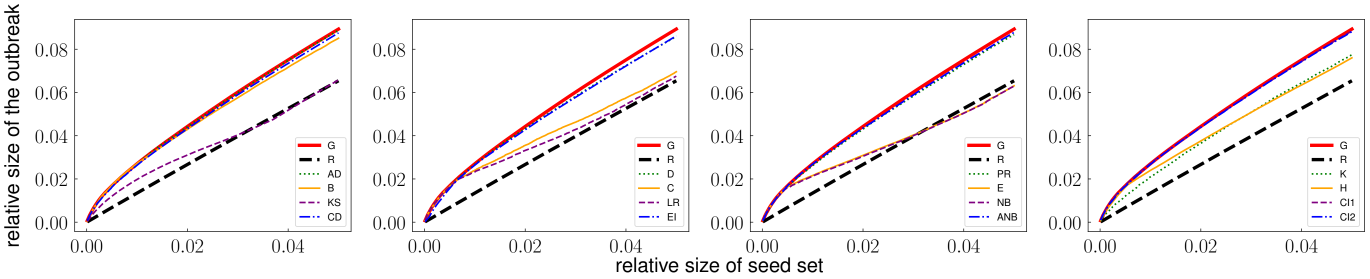

Figure 83: Gnutella, Aug. 5, 2002 - p=0.5p_c

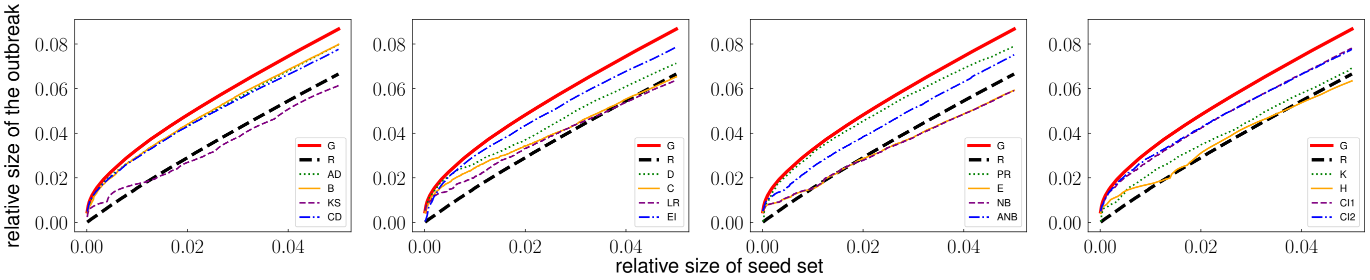

Figure 84: PGP - p=0.5p_c

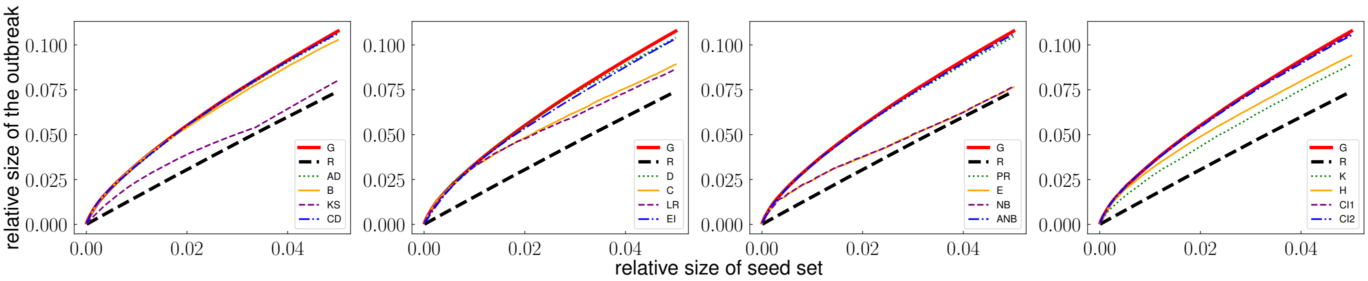

Figure 85: Gnutella, Aug. 4, 2002 - p=0.5p_c

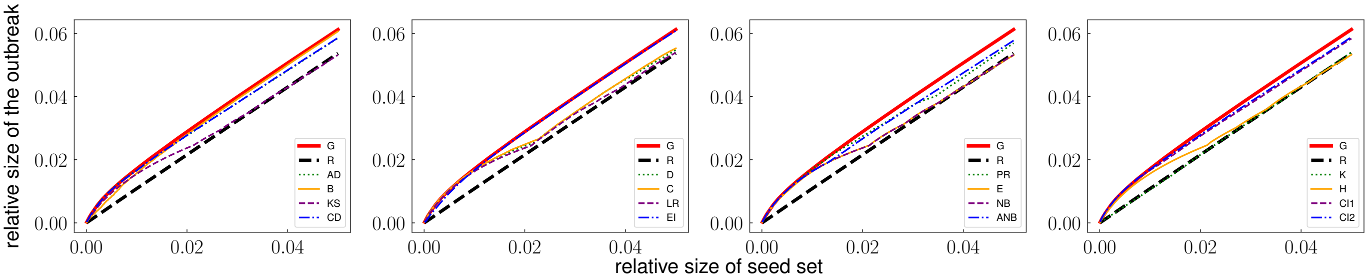

Figure 86: Hep-Ph, 1993-2003 - p=0.5p_c

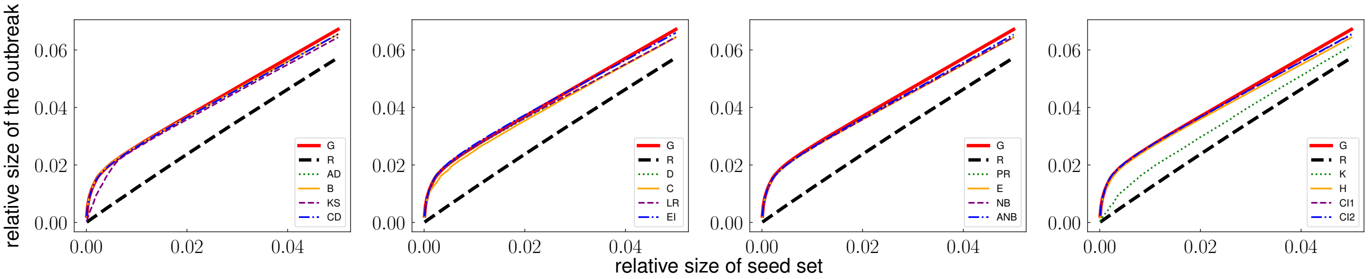

Figure 87: Spanish 1 - p=0.5p_c

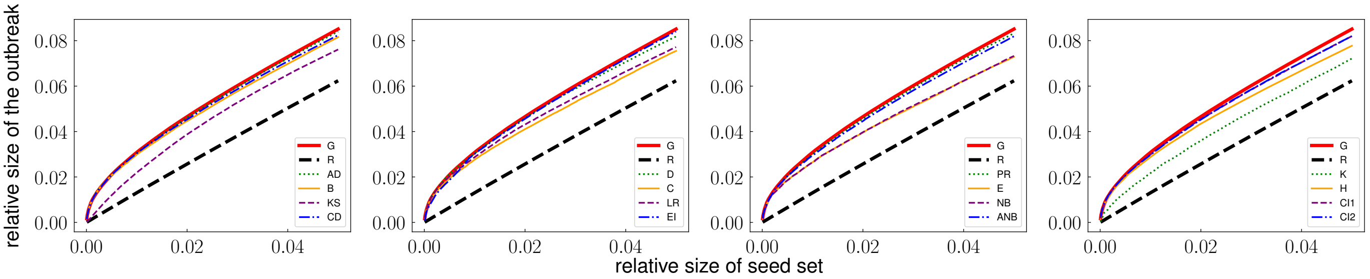

Figure 88: DBLP, citations - p=0.5p$_c$

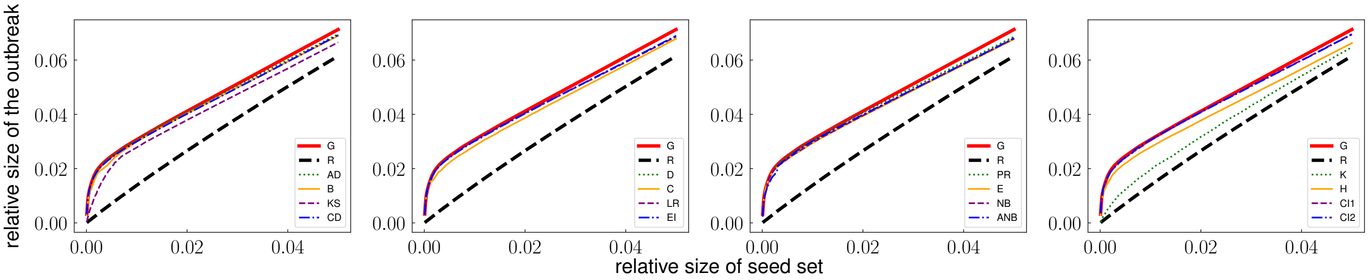

Figure 89: Spanish 2 - p=0.5p$_c$

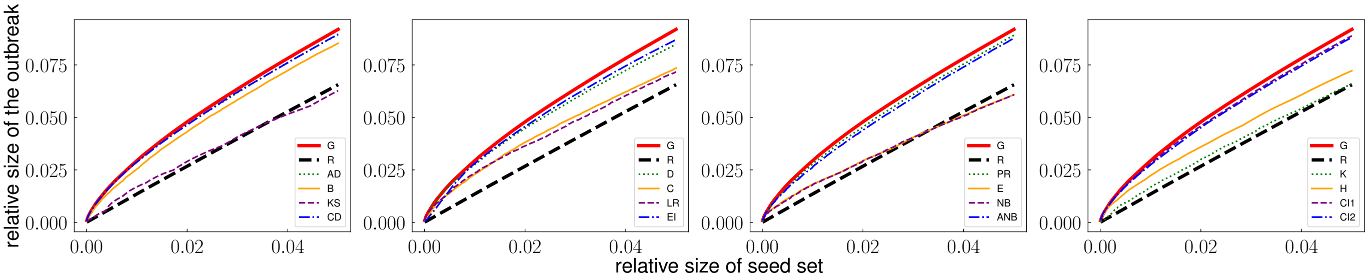

Figure 90: Cond-Mat, 1995-1999 - p=0.5p$_c$

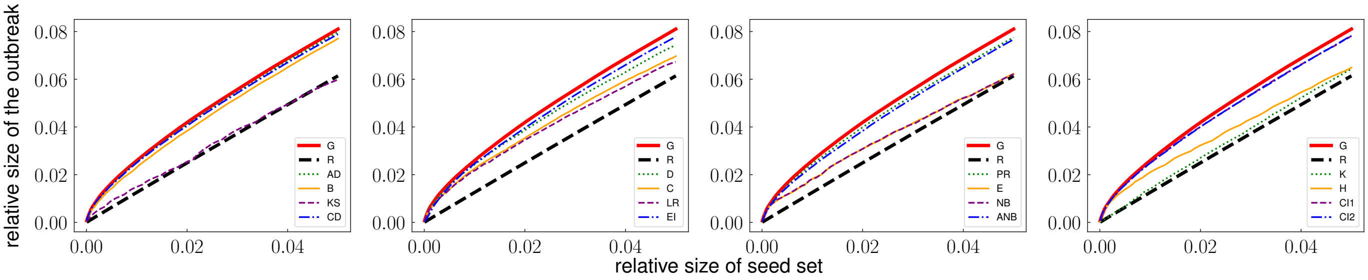

Figure 91: Astrophysics - p=0.5p_c

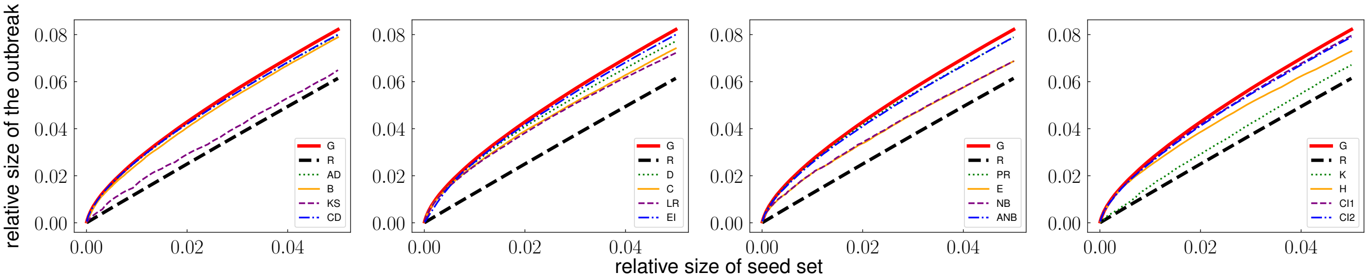

Figure 92: AstroPhys, 1993-2003 - p=0.5p_c

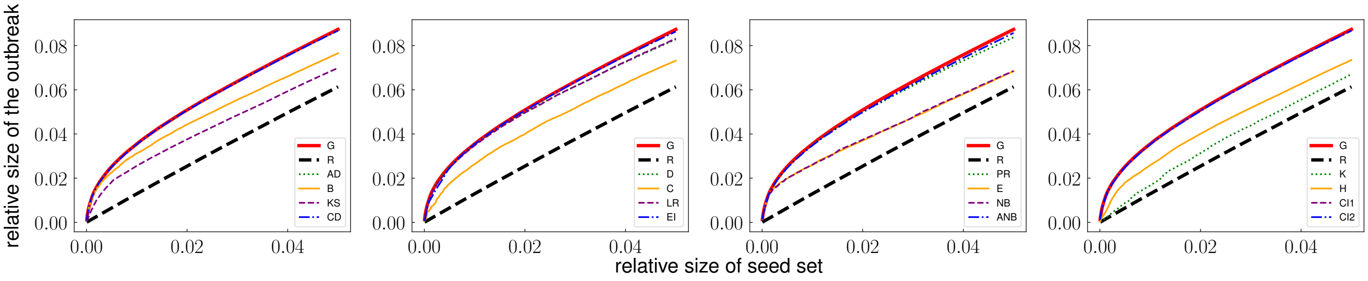

Figure 93: Marvel - p=0.5p_c

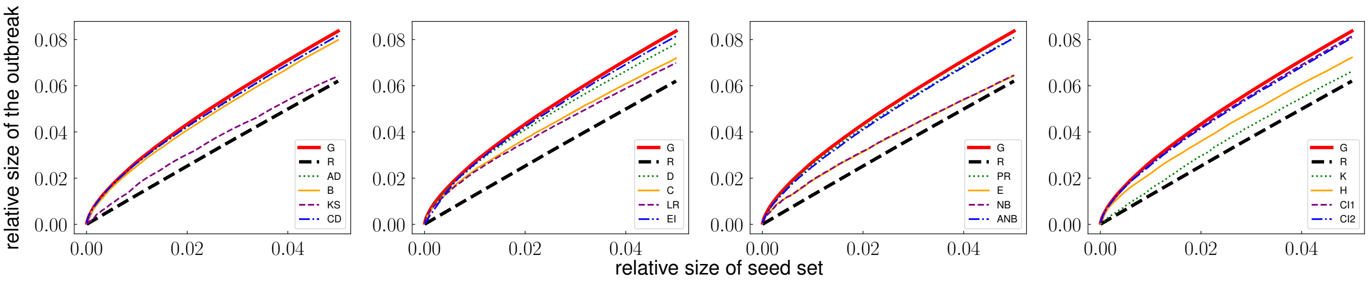

Figure 94: Cond-Mat, 1993-2003 - p=0.5p_c

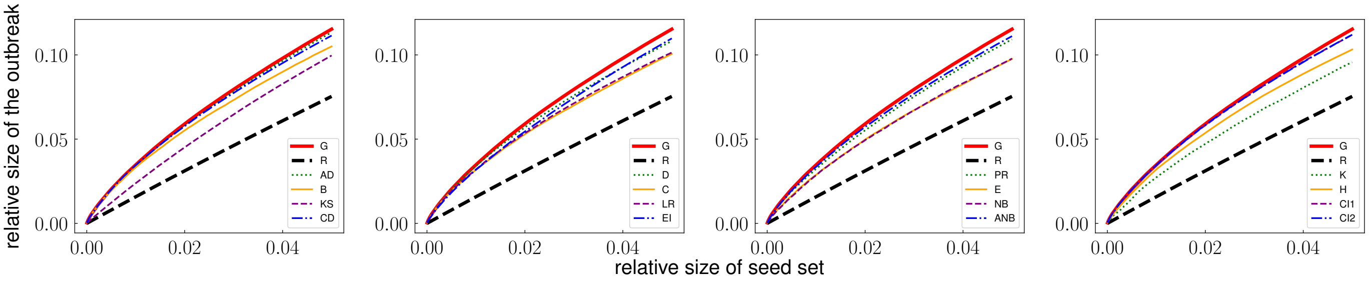

Figure 95: Gnutella, Aug. 25, 2002 - p=0.5p_c

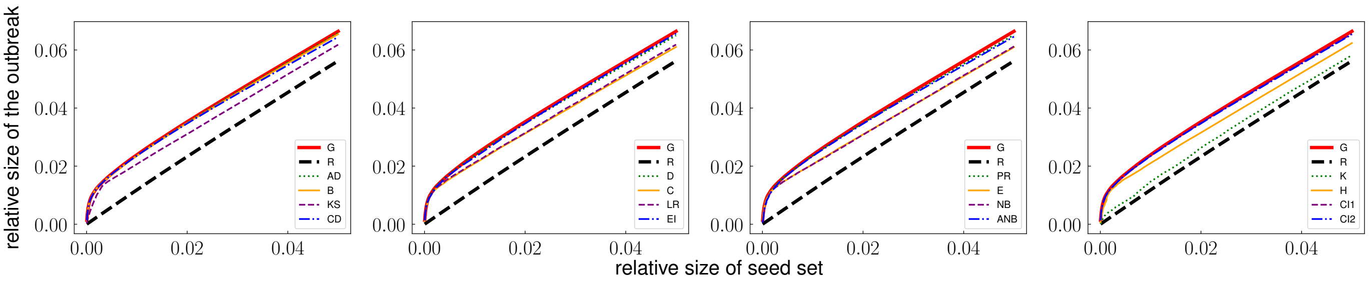

Figure 96: Internet - p=0.5p_c

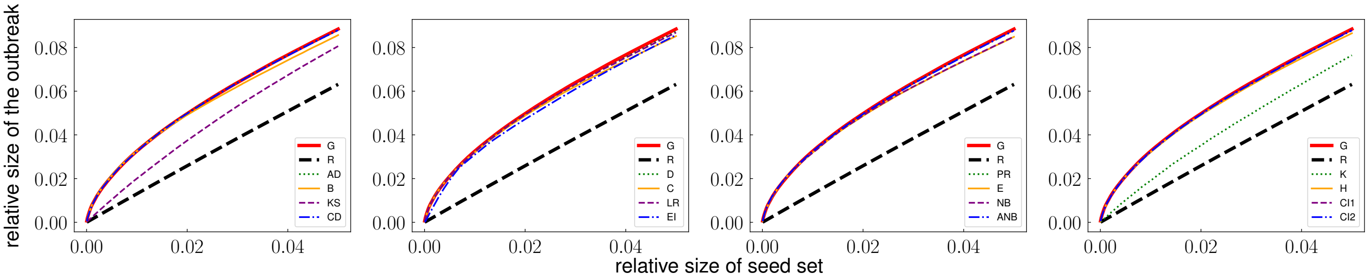

Figure 97: Thesaurus - p=0.5p_c

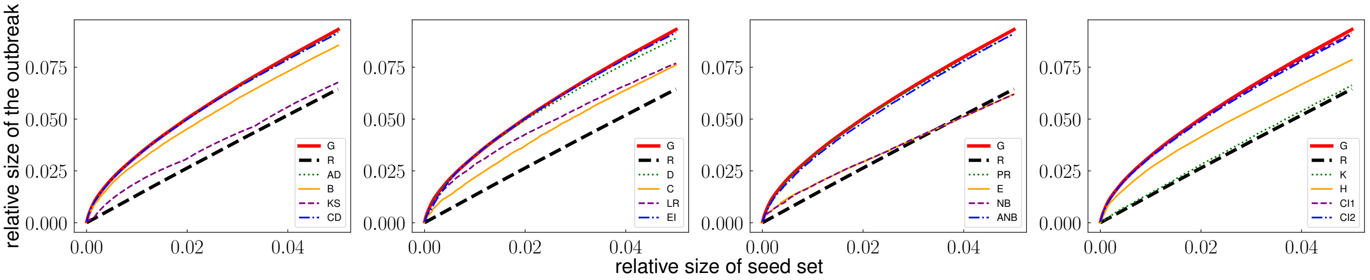

Figure 98: Cora - p=0.5p_c

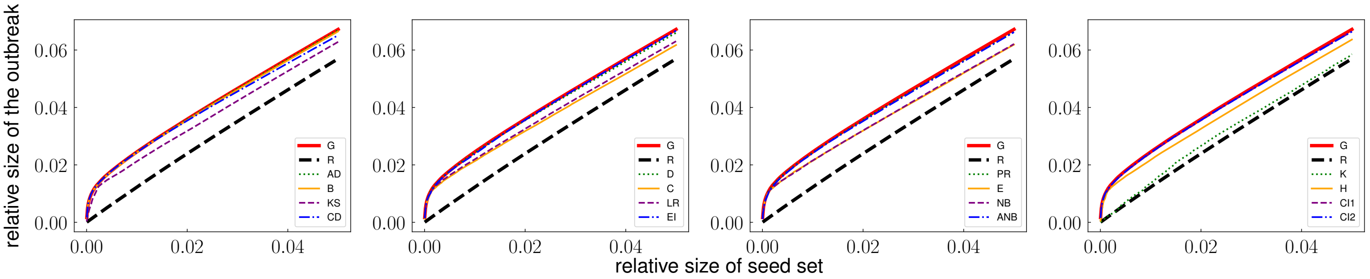

Figure 99: AS Caida - p=0.5p_c

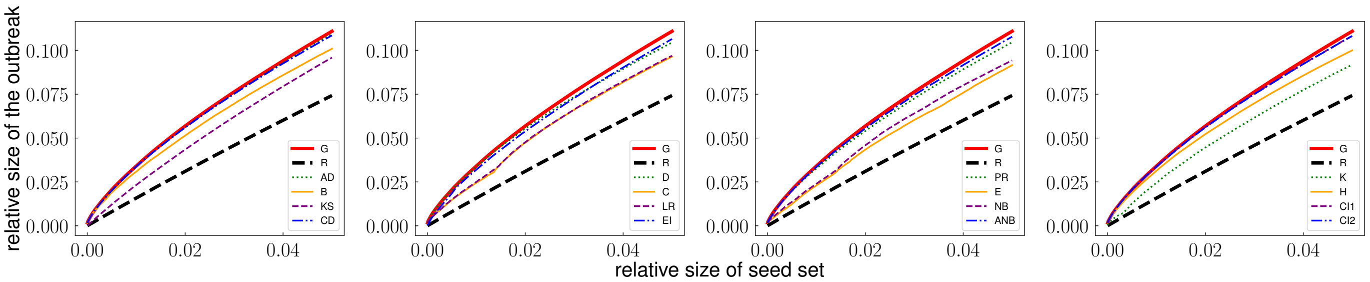

Figure 100: Gnutella, Aug. 24, 2002 - p=0.5p_c

2.1.2 Critical Regime

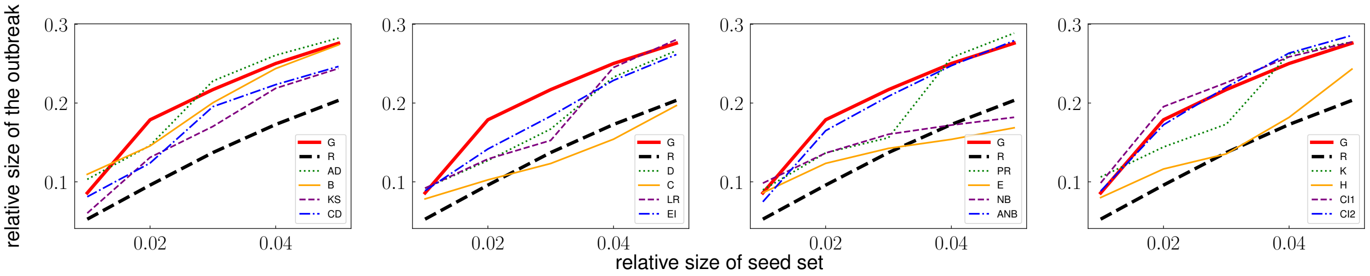

Figure 101: Political books - p=1.0p_c

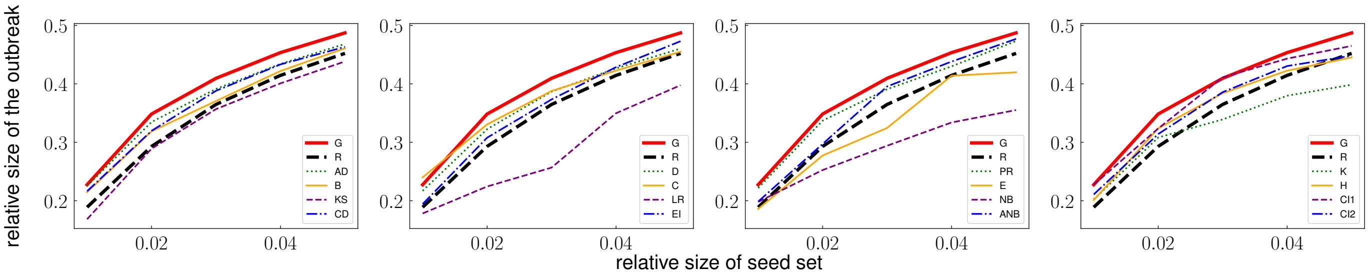

Figure 102: College football - p=1.0p_c

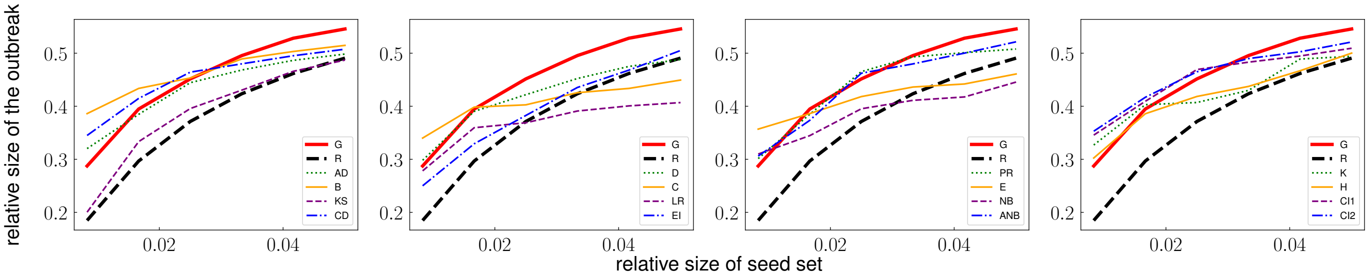

Figure 103: S208 - p=1.0p_c

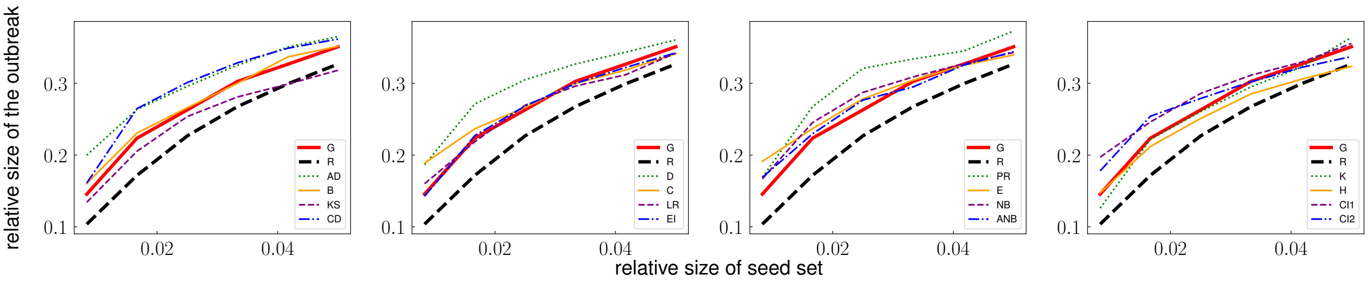

Figure 104: High school, 2011 - p=1.0p_c

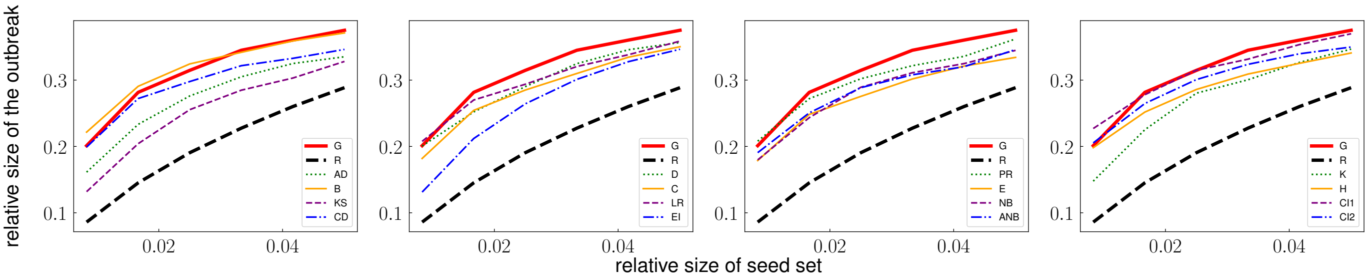

Figure 105: Bay Dry - p=1.0p_c

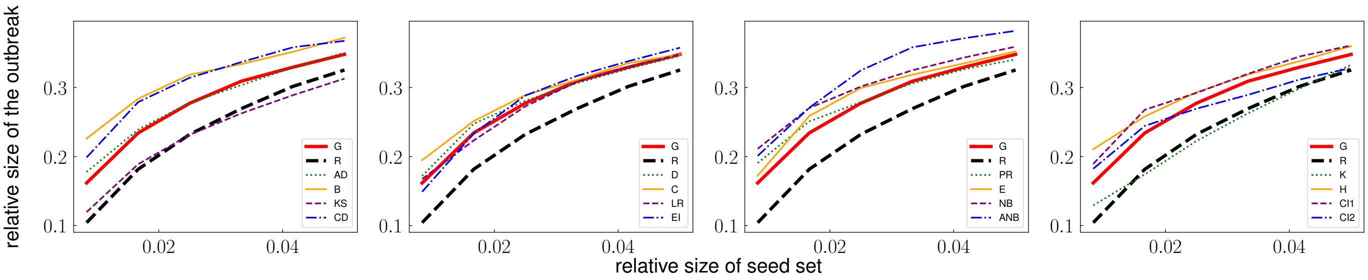

Figure 106: Bay Wet - p=1.0p_c

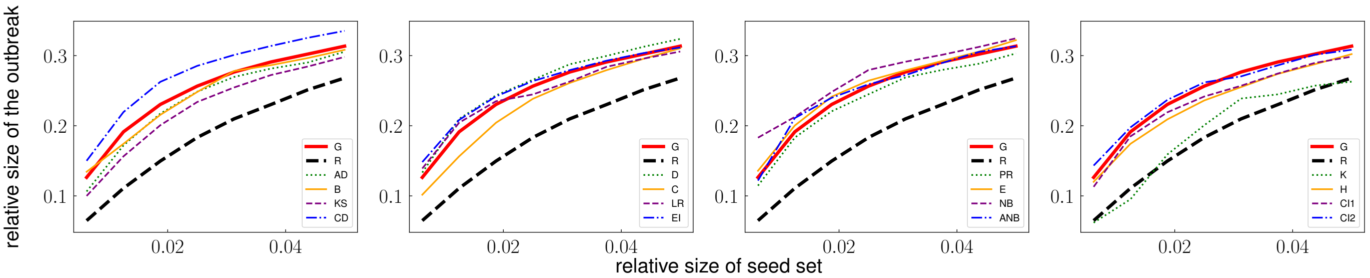

Figure 107: Radoslaw Email - p=1.0p_c

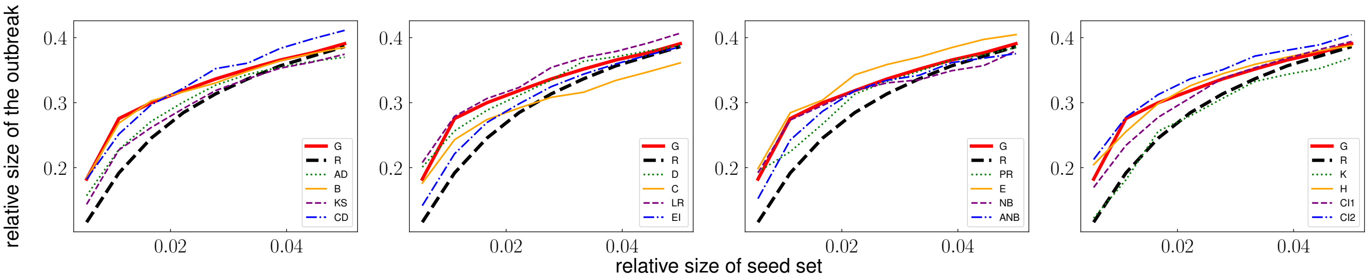

Figure 108: High school, 2012 - p=1.0p_c

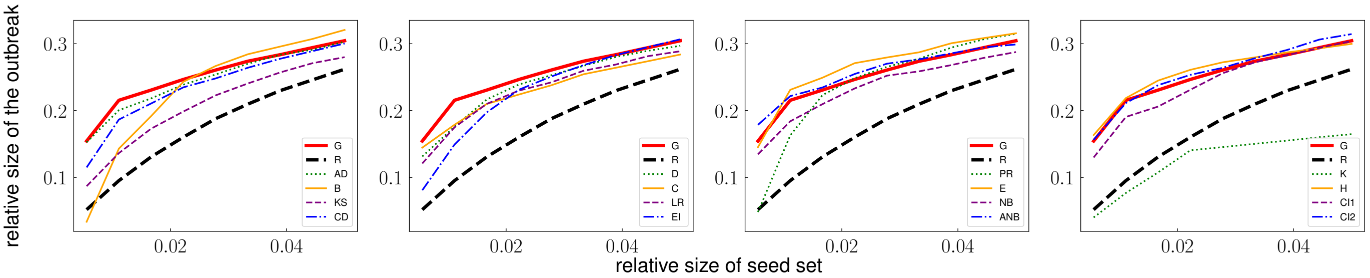

Figure 109: Little Rock Lake - p=1.0p_c

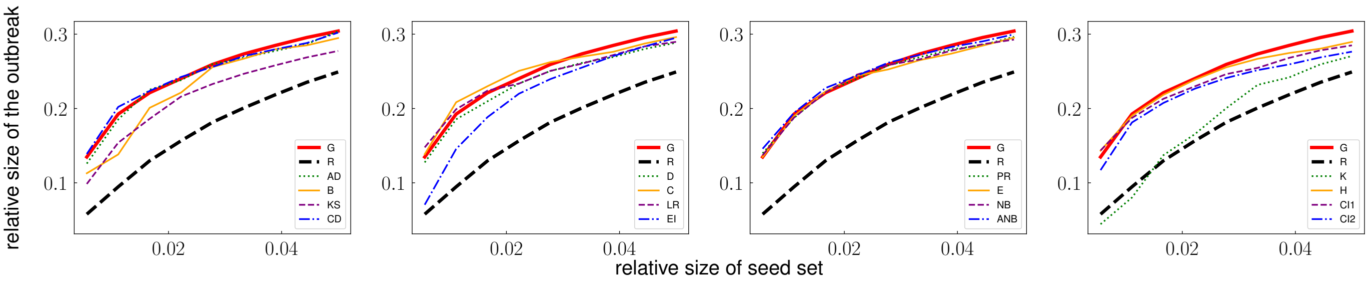

Figure 110: Jazz - p=1.0p_c

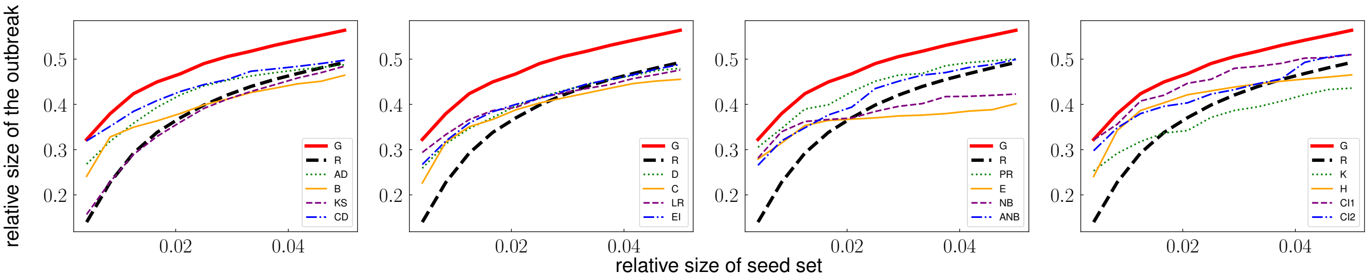

Figure 111: S420 - p=1.0p_c

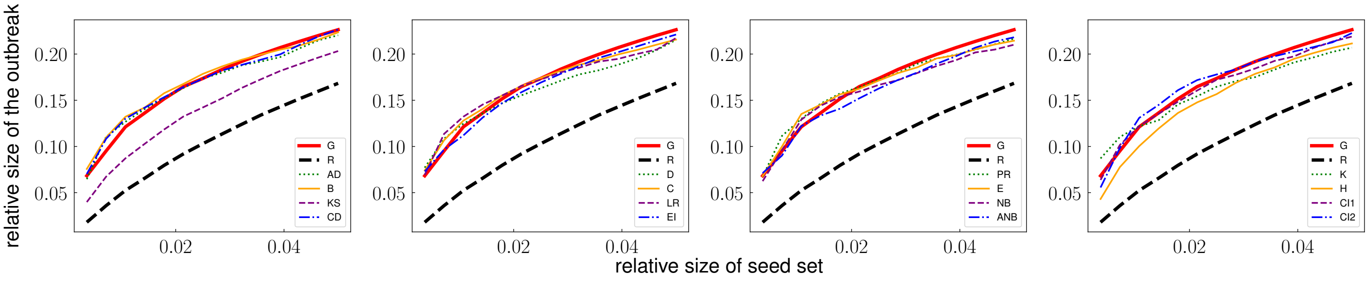

Figure 112: C. Elegans, neural - p=1.0p_c

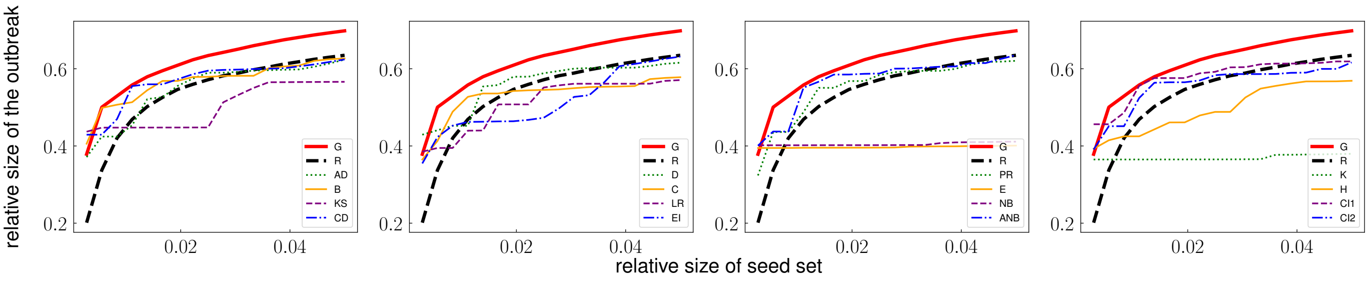

Figure 113: Network Science - p=1.0p_c

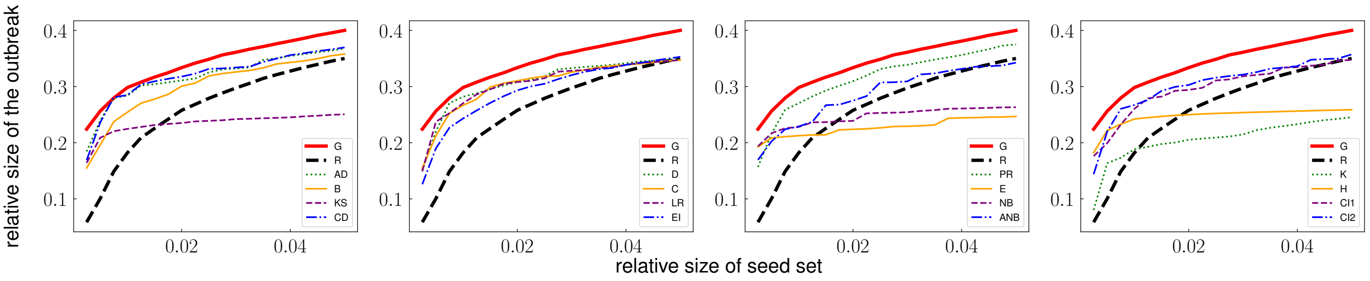

Figure 114: Dublin - p=1.0p_c

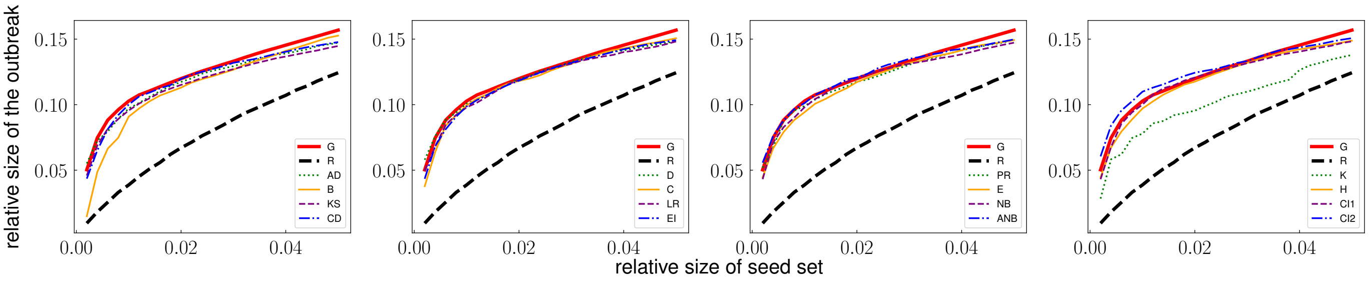

Figure 115: US Air Trasportation - p=1.0p_c

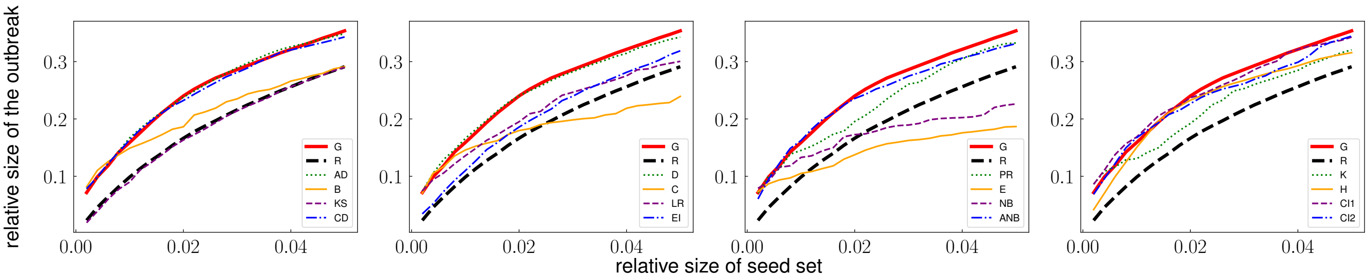

Figure 116: S838 - p=1.0p_c

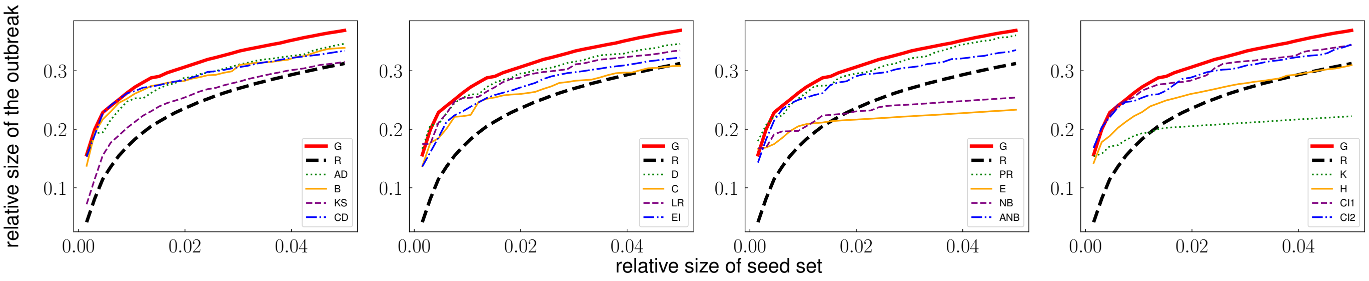

Figure 117: Yeast, transcription - p=1.0p_c

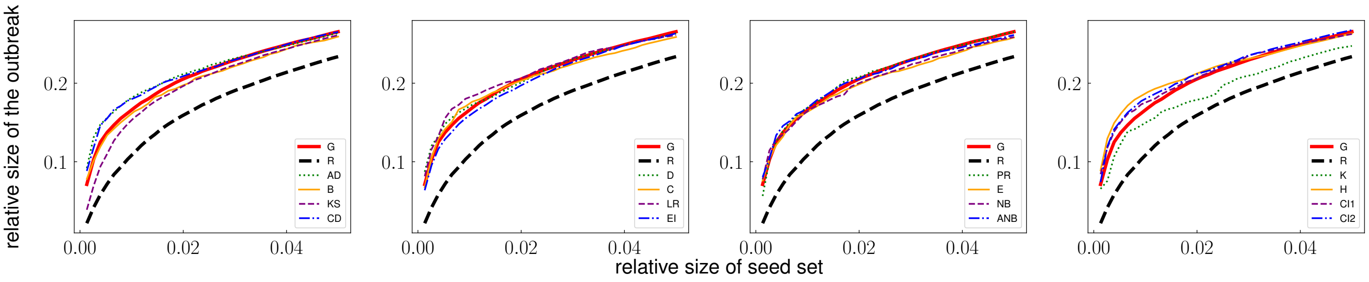

Figure 118: Caltech - p=1.0p_c

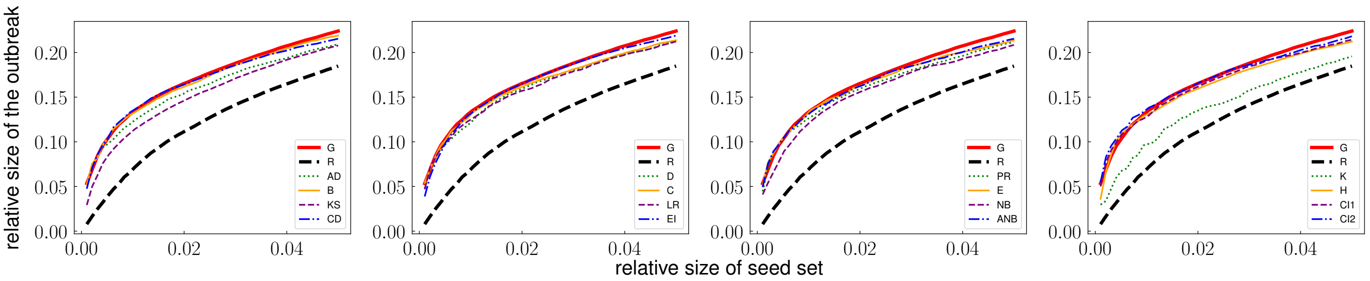

Figure 119: Reed - p=1.0p_c

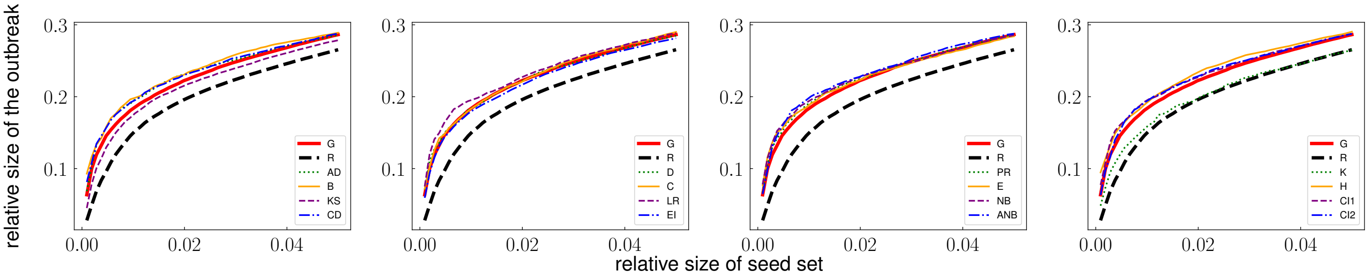

Figure 120: Mouse retina - p=1.0p_c

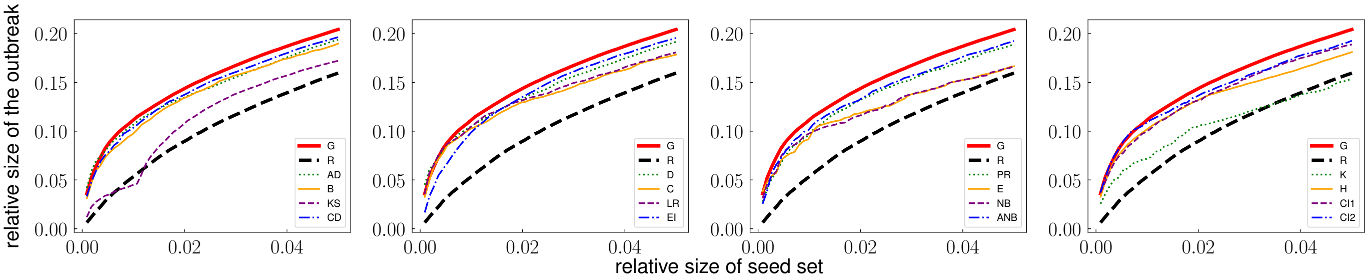

Figure 121: URV email - p=1.0p_c

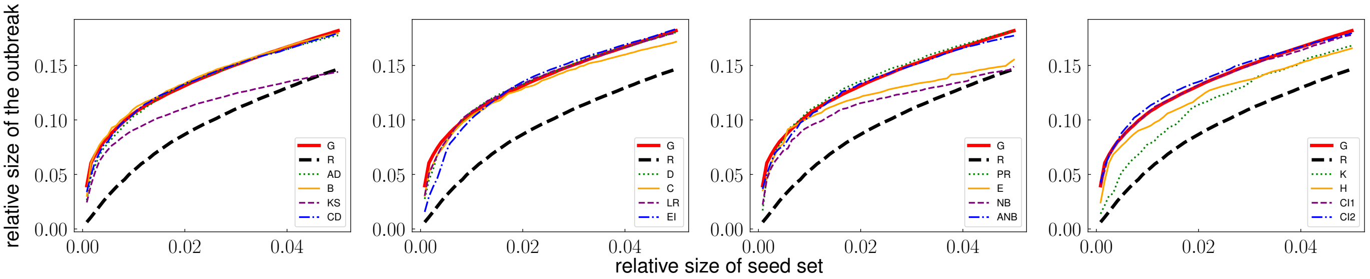

Figure 122: Political blogs - p=1.0p_c

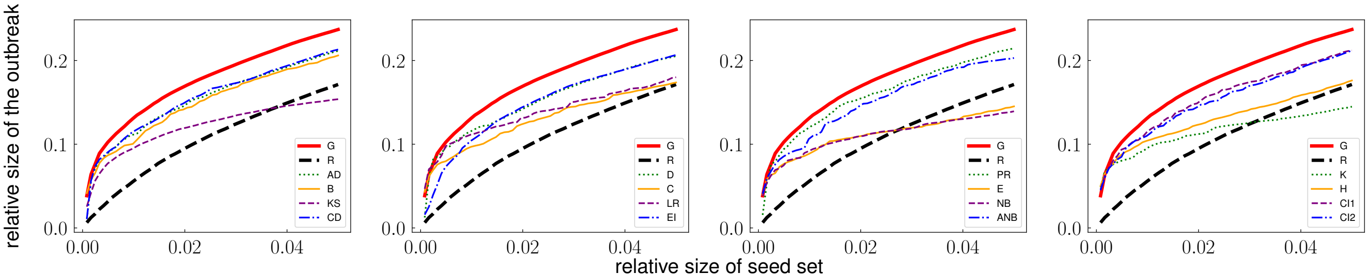

Figure 123: Air traffic - p=1.0p_c

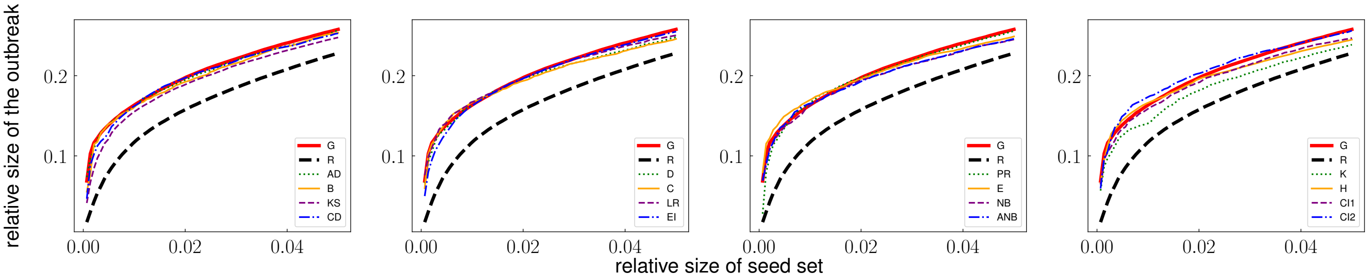

Figure 124: Haverford - p=1.0p_c

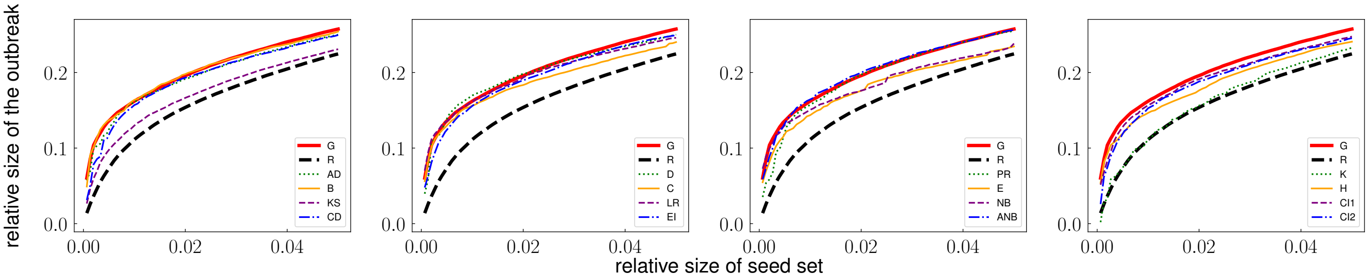

Figure 125: Simmons - p=1.0p_c

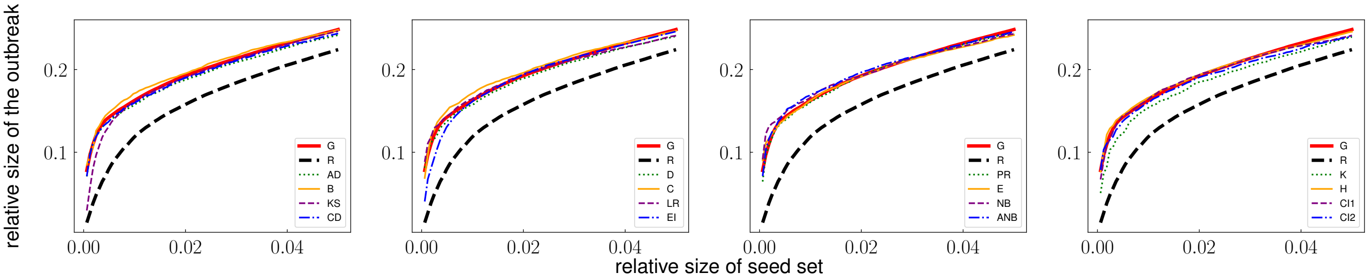

Figure 126: Swarthmore - p=1.0p_c

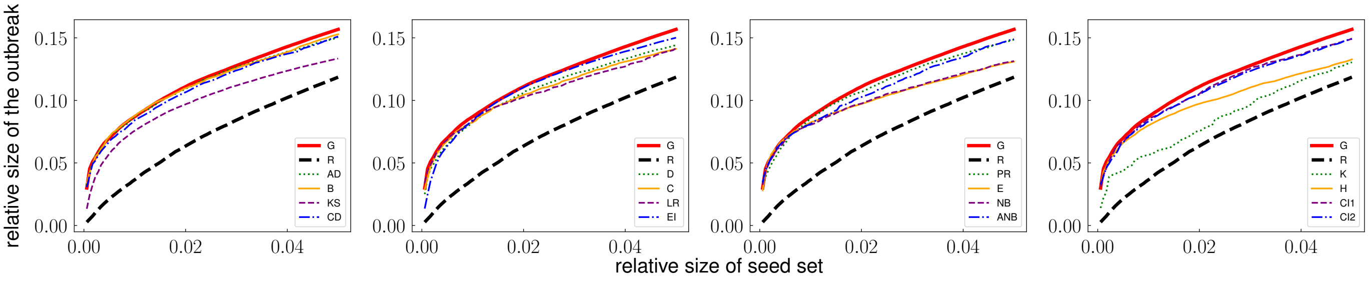

Figure 127: Petster, hamster - p=1.0p_c

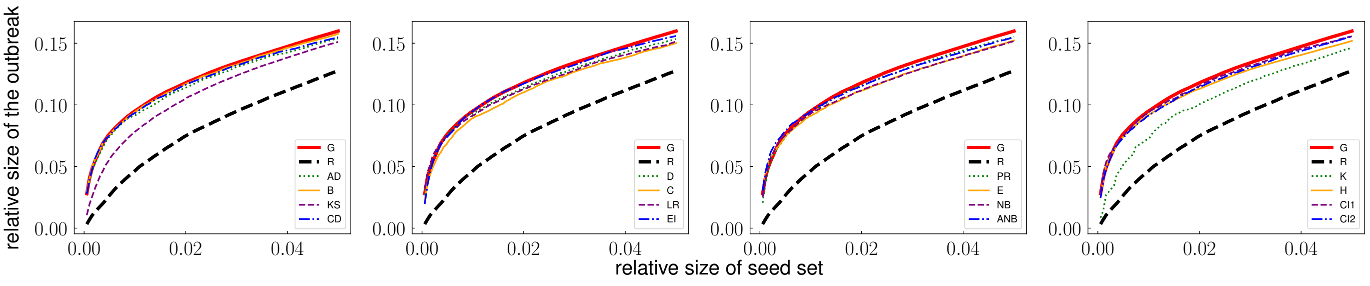

Figure 128: UC Irvine - p=1.0p_c

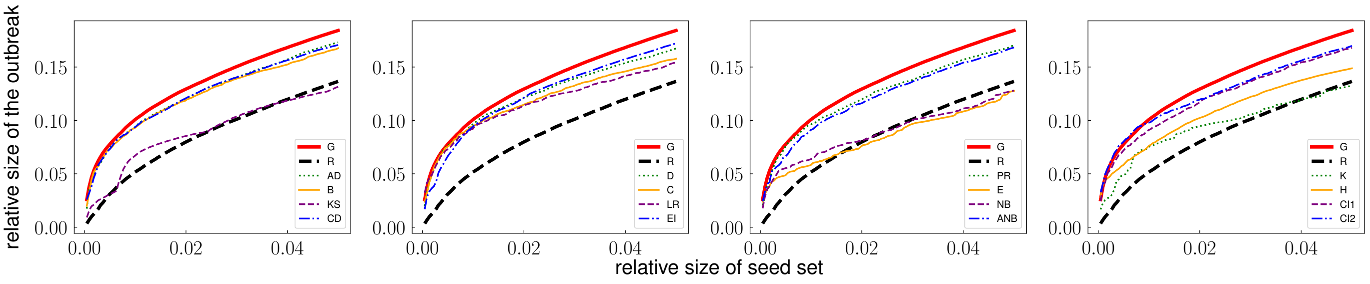

Figure 129: Yeast, protein - p=1.0p_c

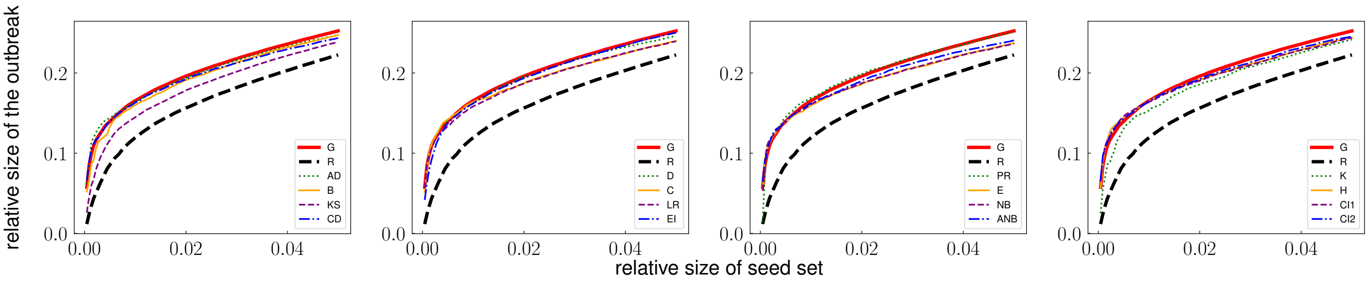

Figure 130: Amherst - p=1.0p_c

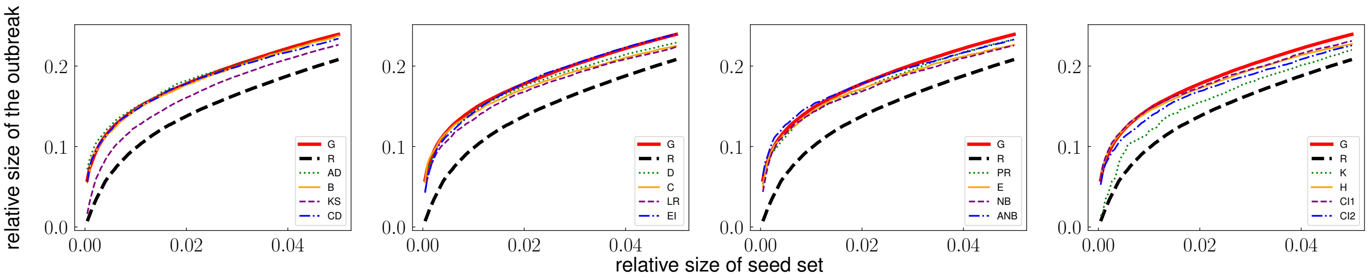

Figure 131: Bowdoin - p=1.0p_c

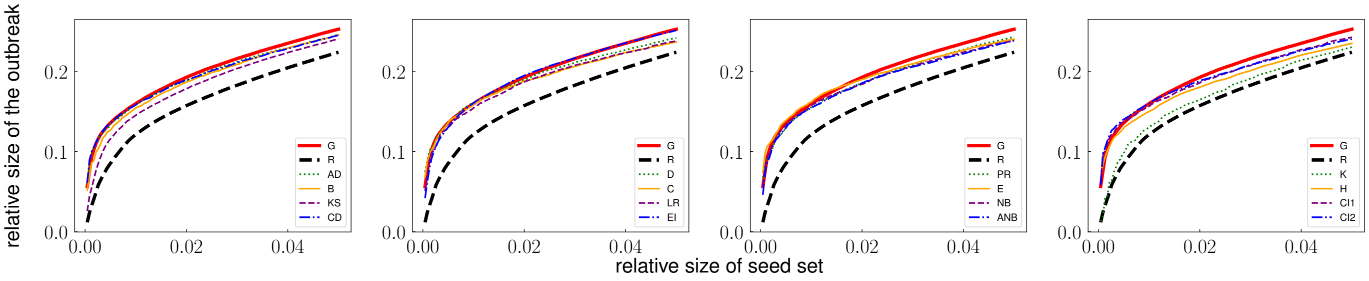

Figure 132: Hamilton - p=1.0p_c

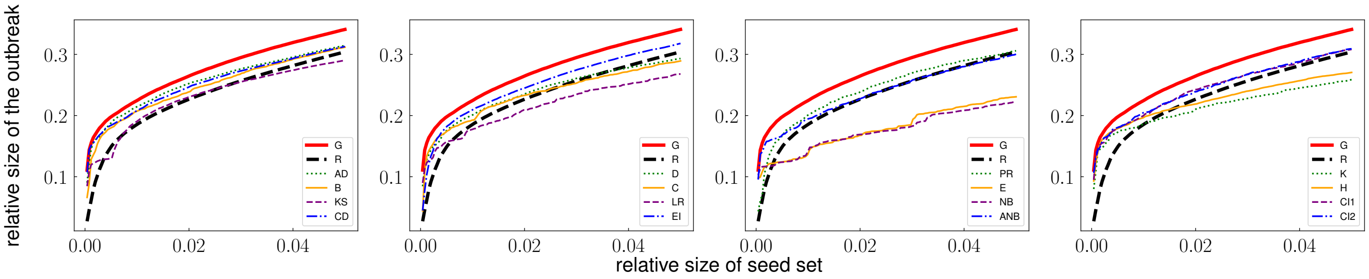

Figure 133: Adolescent health - p=1.0p_c

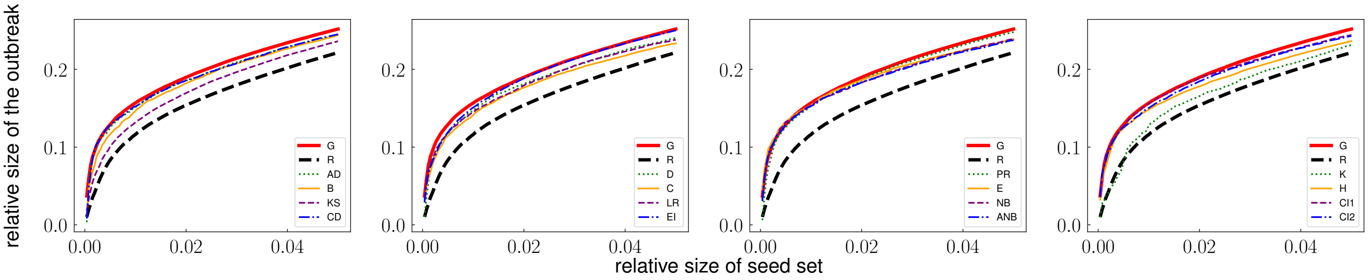

Figure 134: Trinity - p=1.0p_c

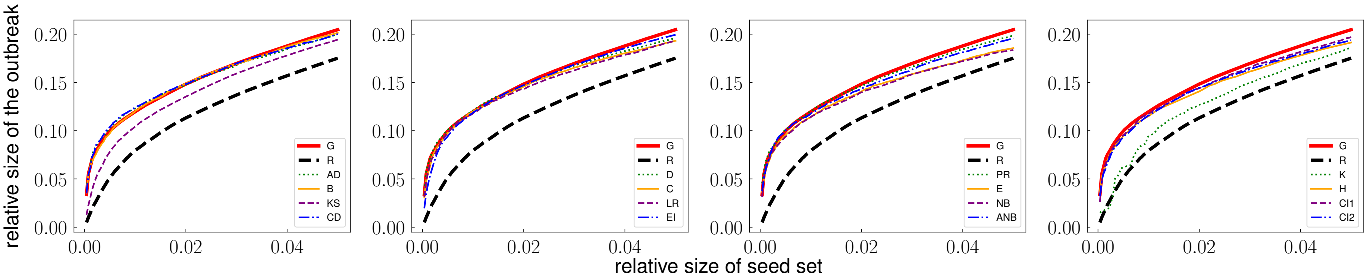

Figure 135: USFCA - p=1.0p_c

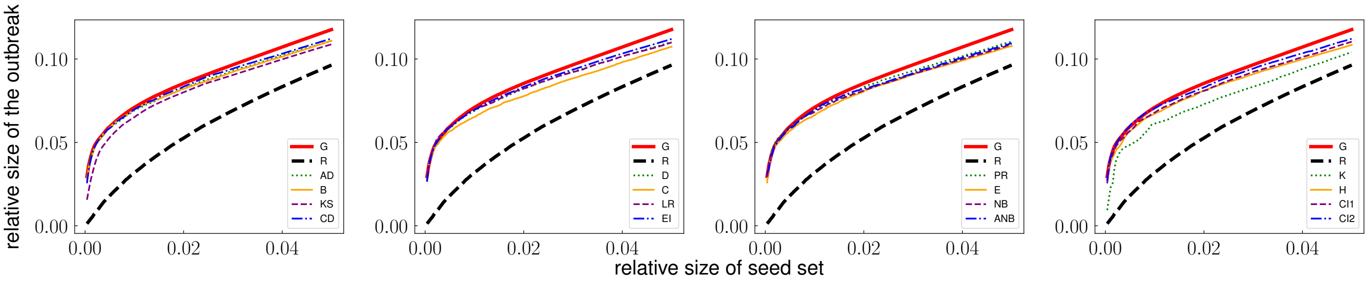

Figure 136: Japanese - p=1.0p_c

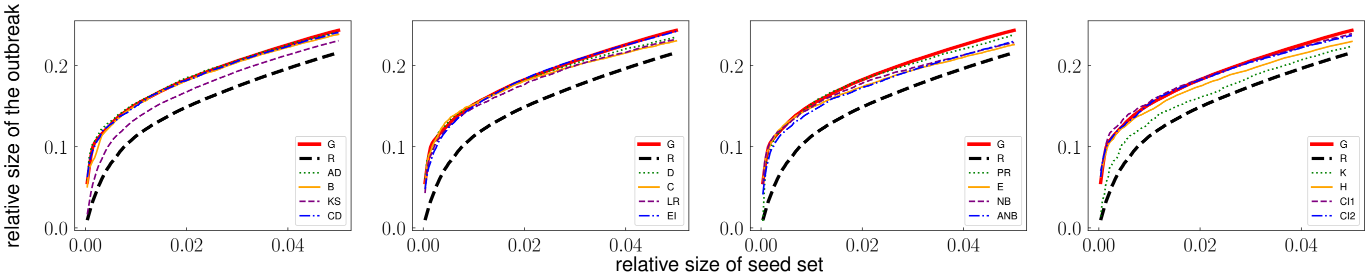

Figure 137: Williams - p=1.0p_c

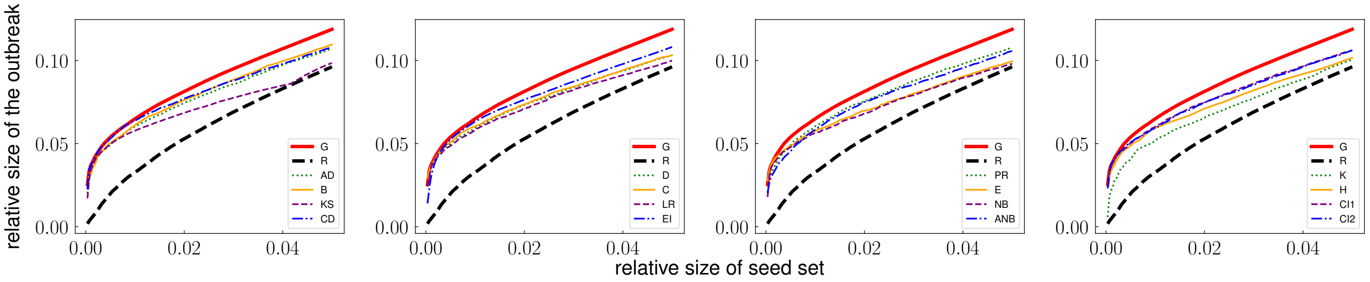

Figure 138: Open flights - p=1.0p_c

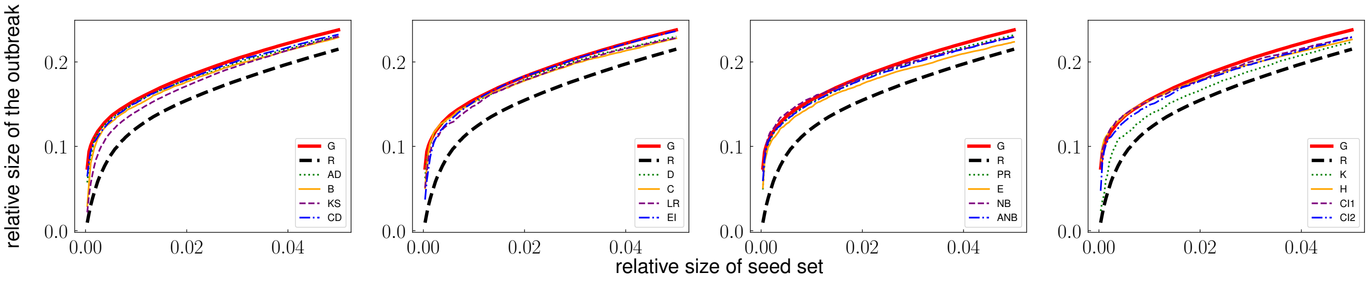

Figure 139: Oberlin - p=1.0p_c

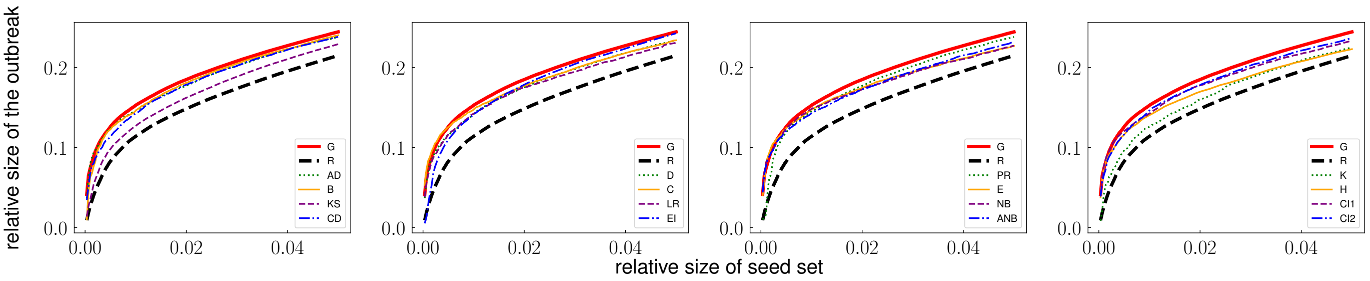

Figure 140: Smith - p=1.0p_c

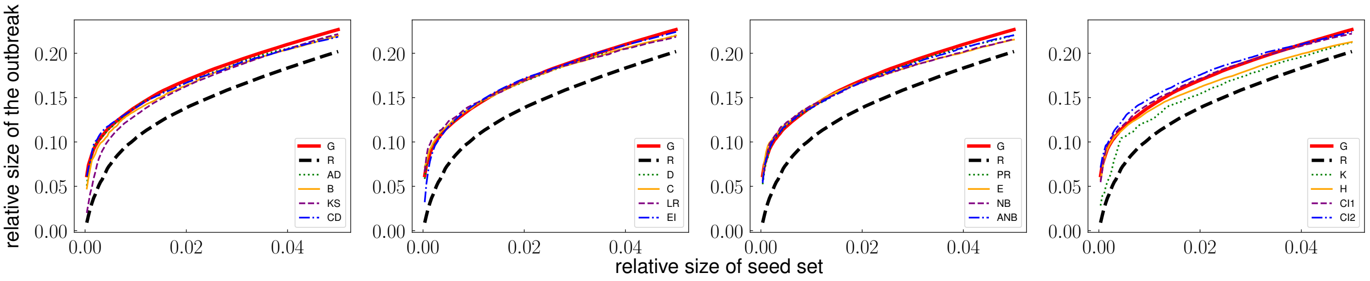

Figure 141: Wellesley - p=1.0p_c

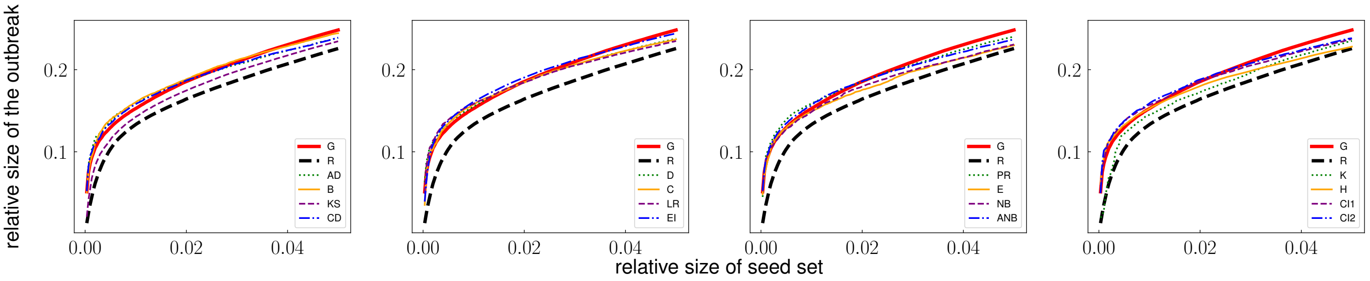

Figure 142: Vassar - p=1.0p_c

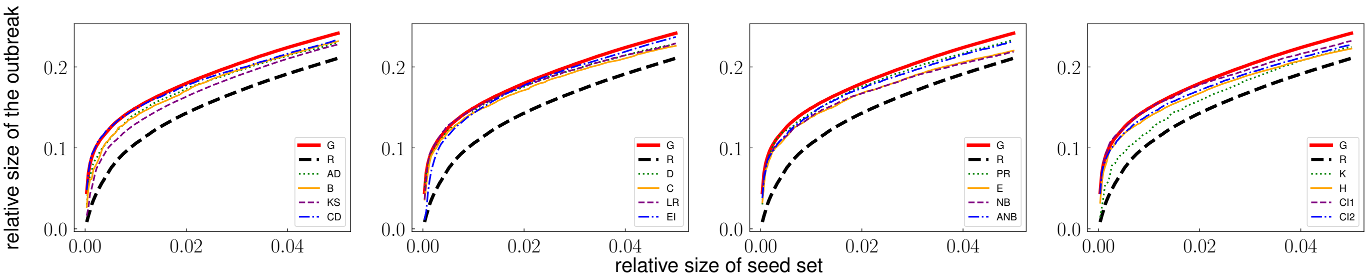

Figure 143: Middlebury - p=1.0p_c

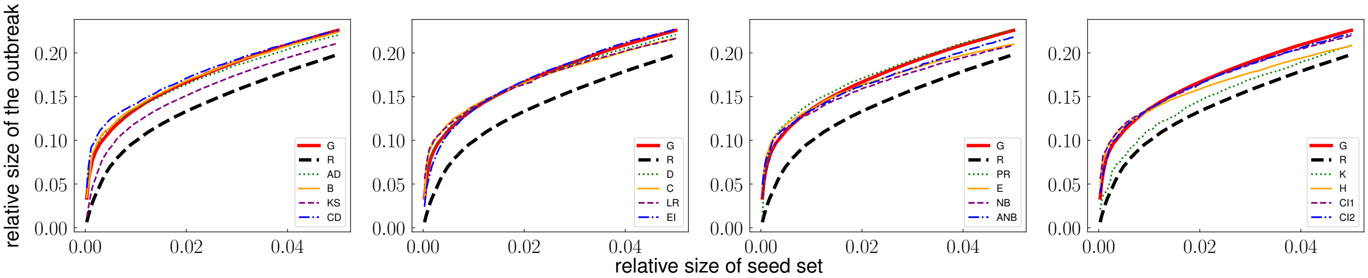

Figure 144: Pepperdine - p=1.0p_c

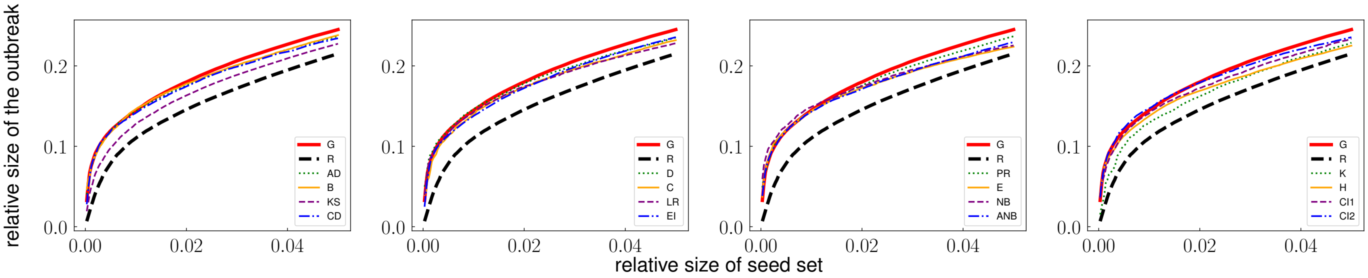

Figure 145: Colgate - p=1.0p_c

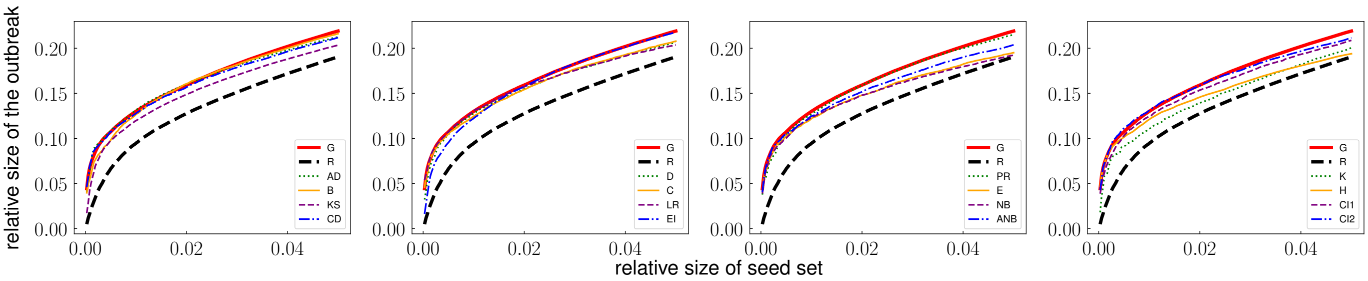

Figure 146: Santa - p=1.0p_c

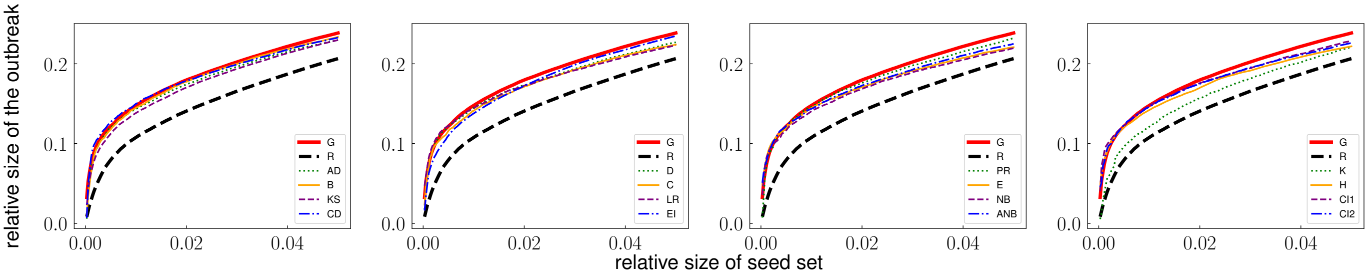

Figure 147: Wesleyan - p=1.0p_c

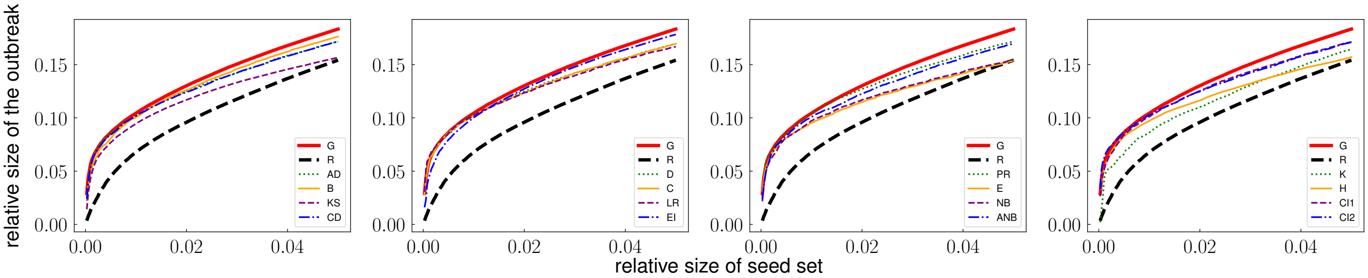

Figure 148: Mich - p=1.0p_c

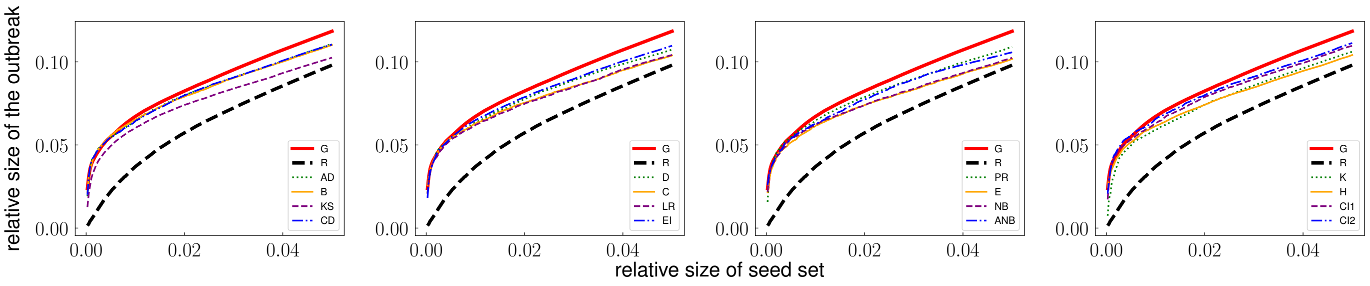

Figure 149: Bitcoin Alpha - p=1.0p$_c$

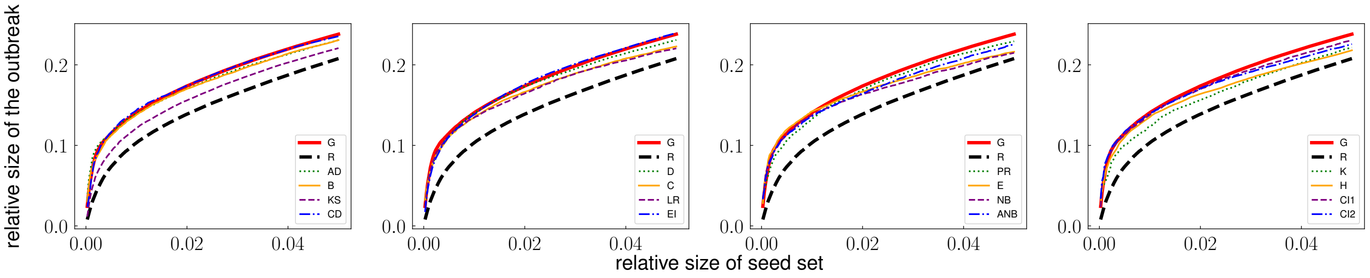

Figure 150: Bucknell - p=1.0p$_c$

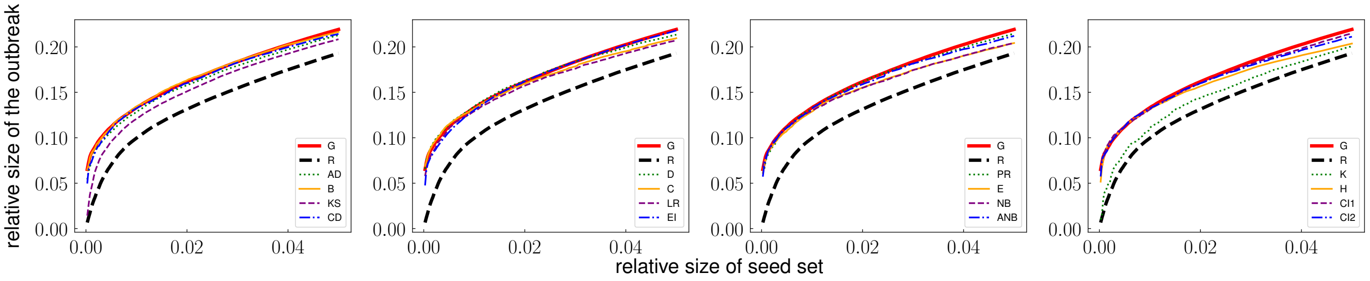

Figure 151: Brandeis - p=1.0p$_c$

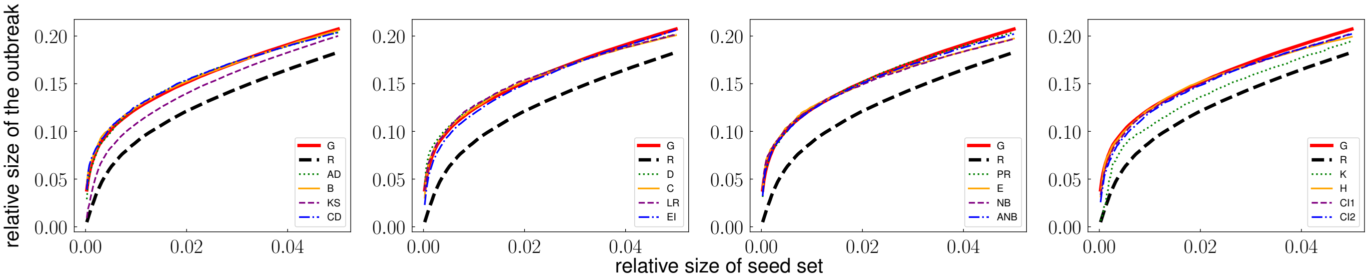

Figure 152: Howard - p=1.0p$_c$

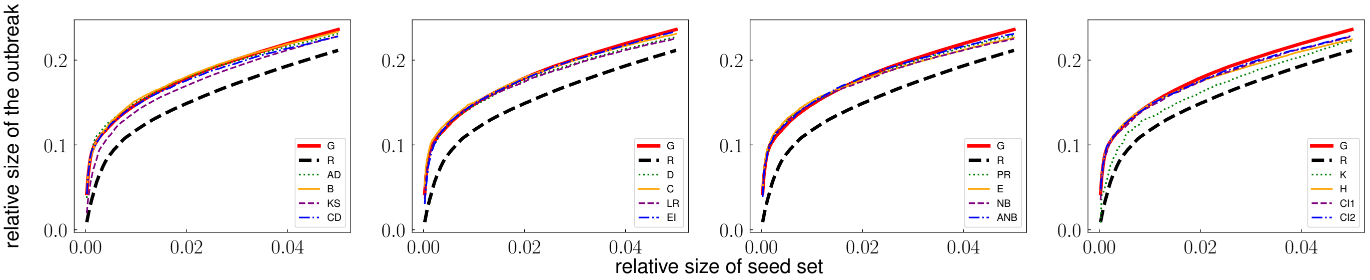

Figure 153: Rice - p=1.0p$_c$

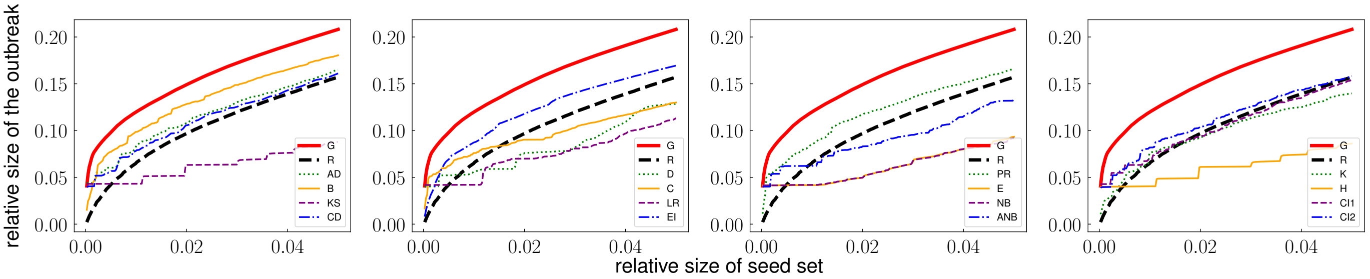

Figure 154: GR-QC, 1993-2003 - p=1.0p$_c$

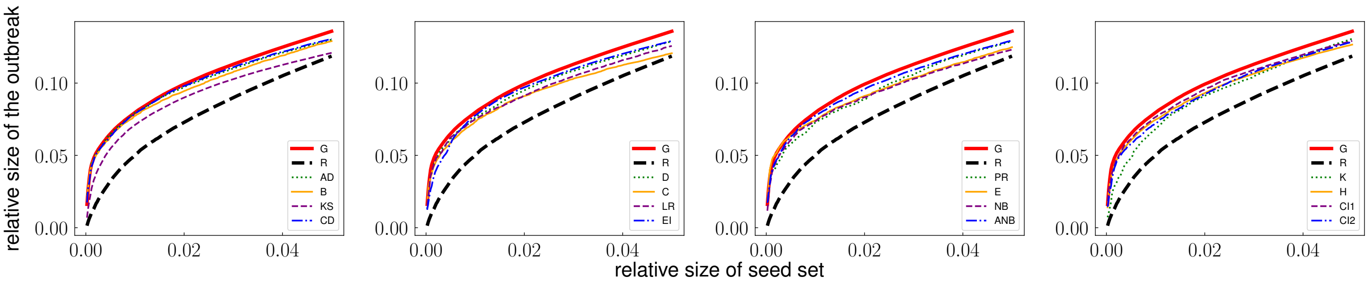

Figure 155: Tennis - $p=1.0p_c$

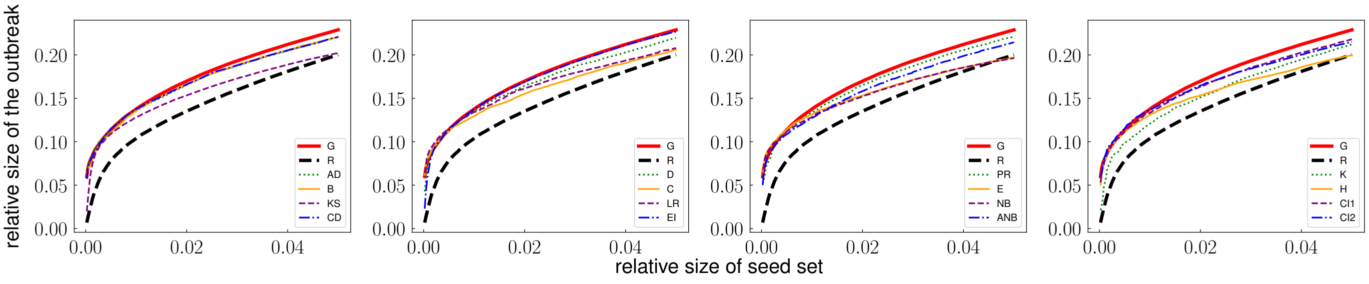

Figure 156: Rochester - $p=1.0p_c$

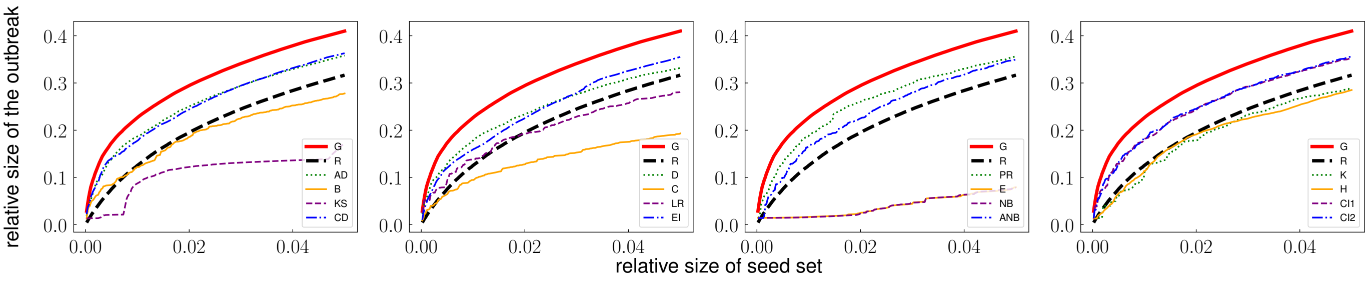

Figure 157: US Power grid - $p=1.0p_c$

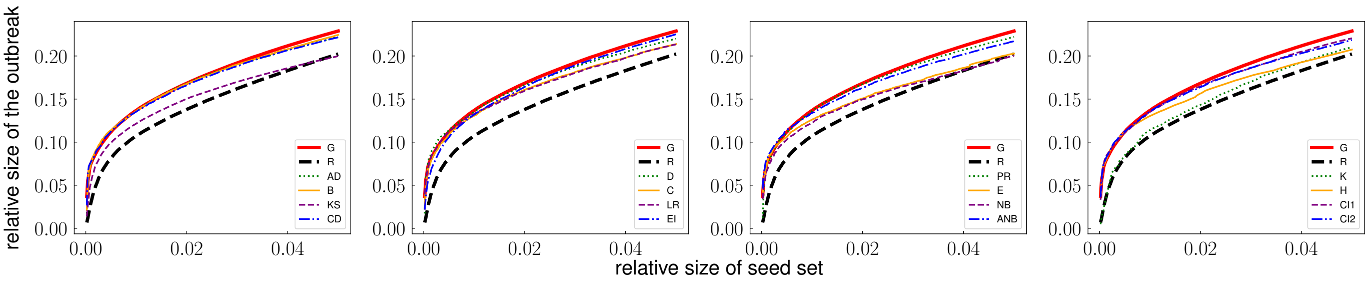

Figure 158: Lehigh - p=1.0p_c

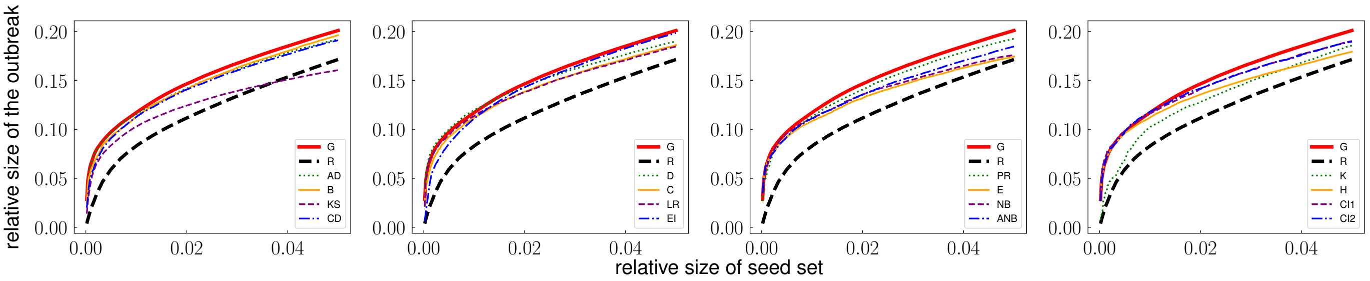

Figure 159: Johns Hopkins - p=1.0p_c

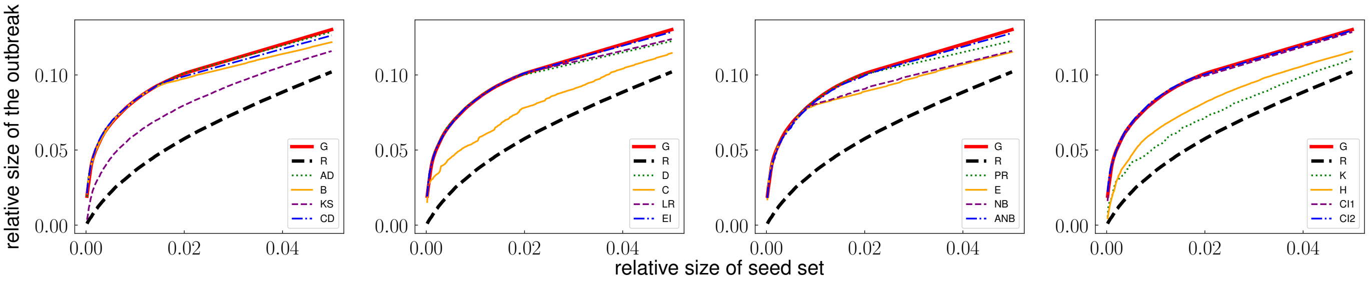

Figure 160: HT09 - p=1.0p_c

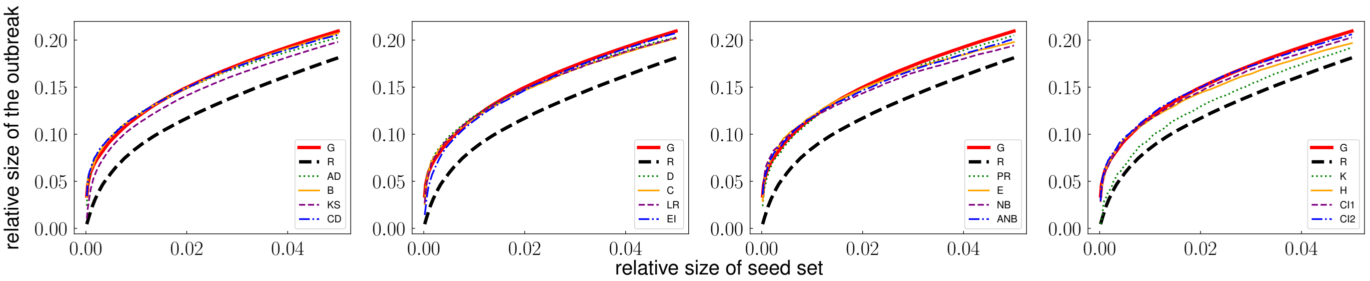

Figure 161: Wake - p=1.0p_c

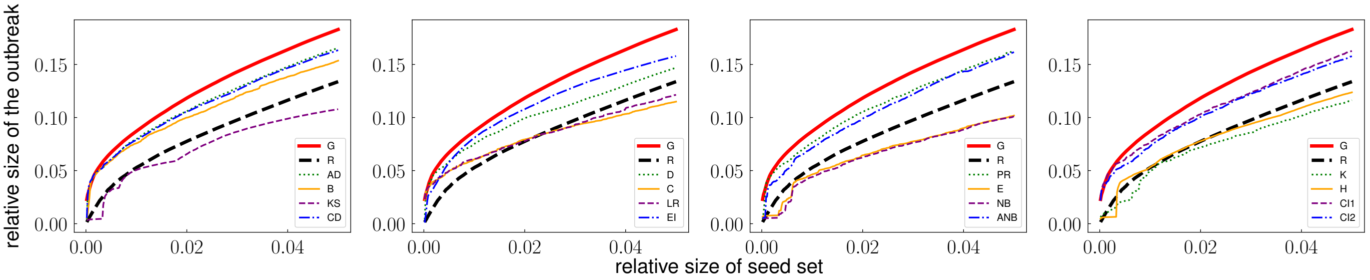

Figure 162: Hep-Th, 1995-1999 - p=1.0p_c

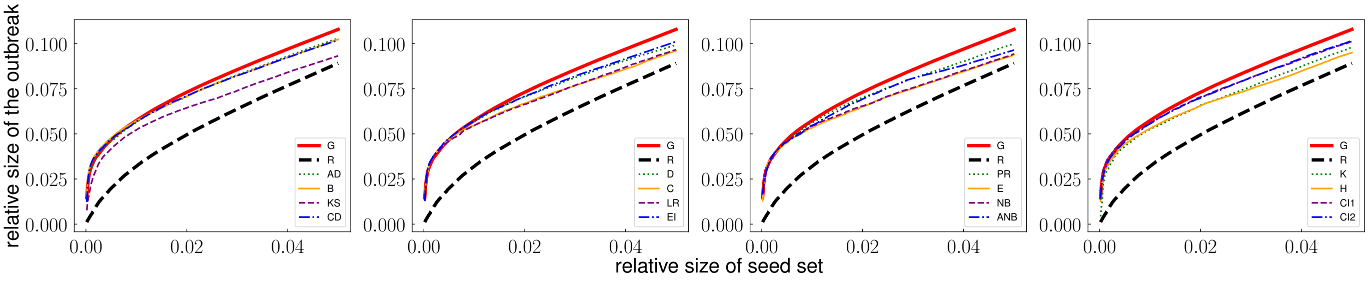

Figure 163: Bitcoin OTC - p=1.0p_c

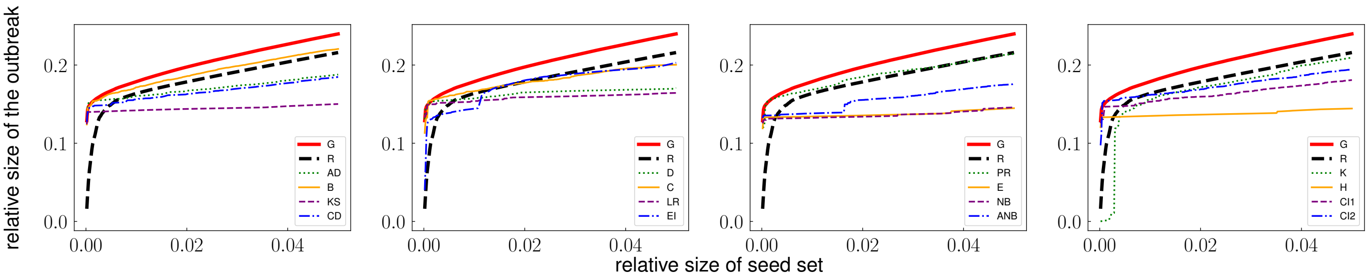

Figure 164: Reactome - p=1.0p_c

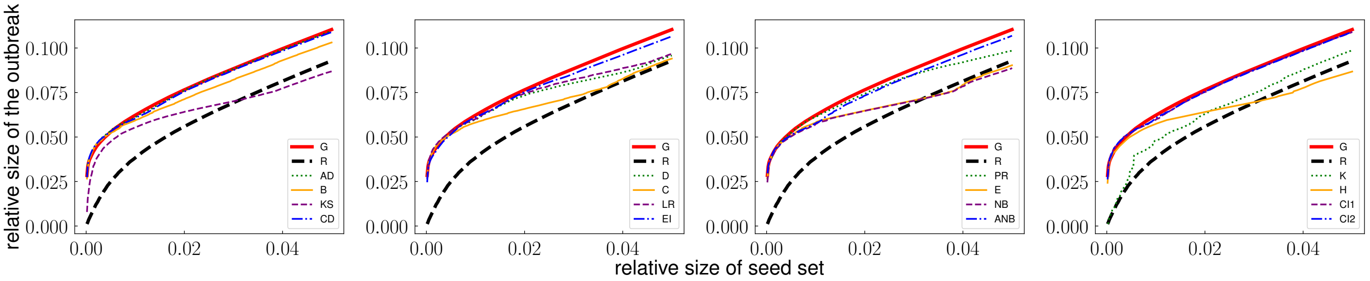

Figure 165: Jung - p=1.0p_c

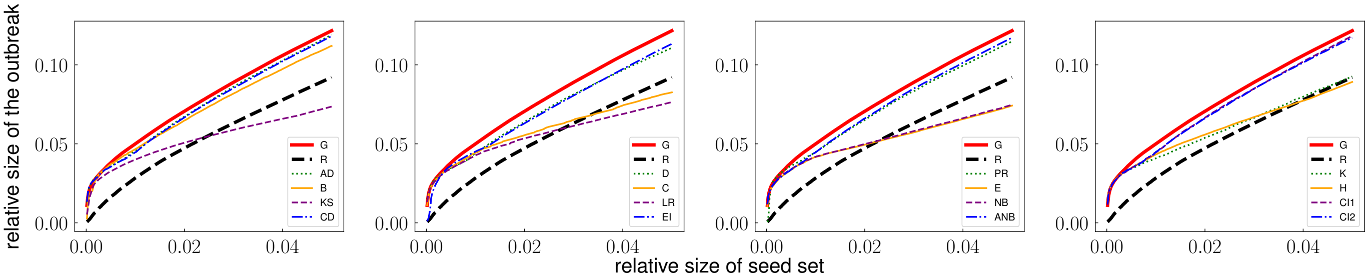

Figure 166: Gnutella, Aug. 8, 2002 - p=1.0p_c

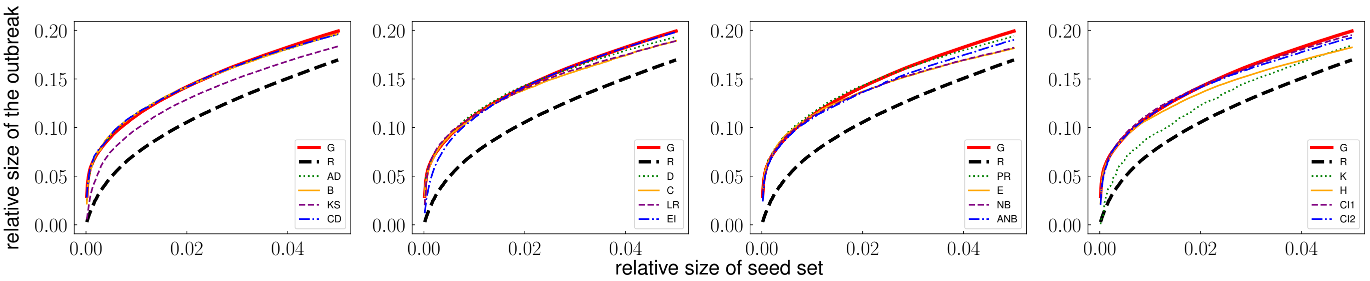

Figure 167: American - p=1.0p_c

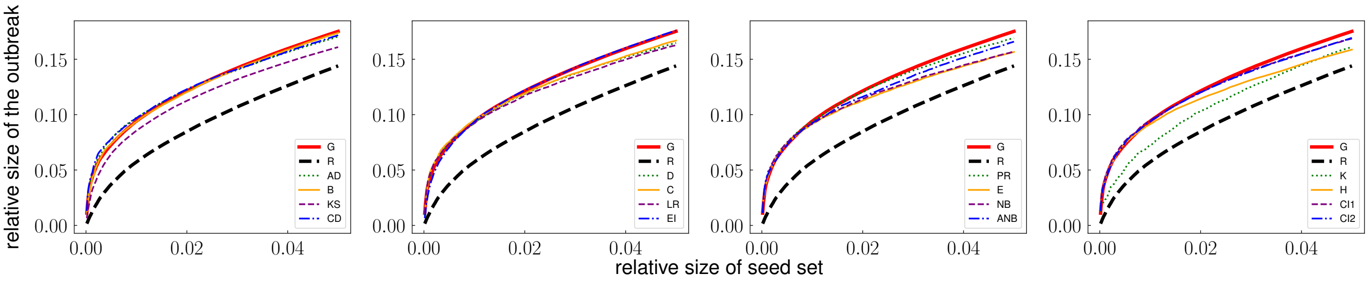

Figure 168: MIT - p=1.0p_c

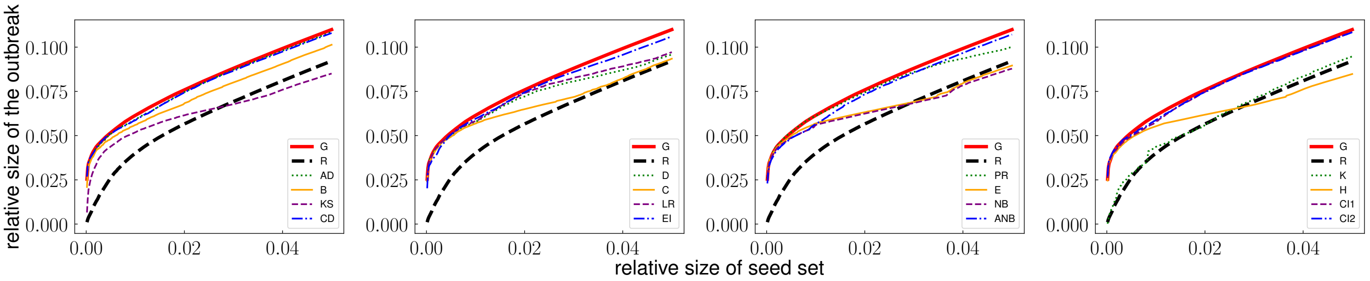

Figure 169: JDK - p=1.0p_c

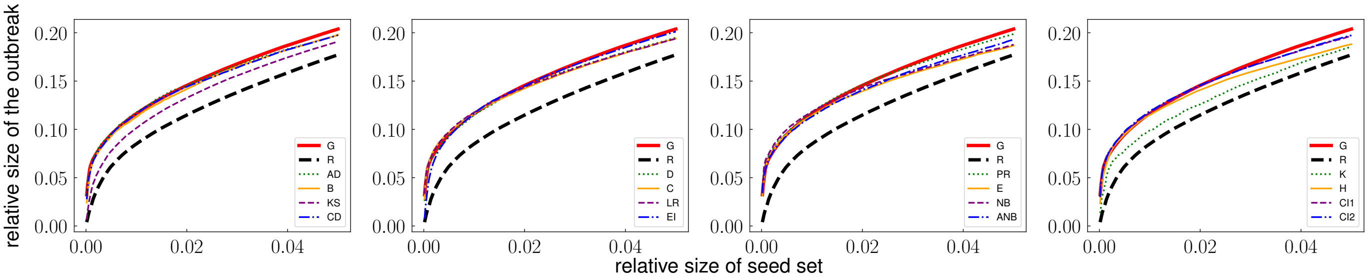

Figure 170: William - p=1.0p_c

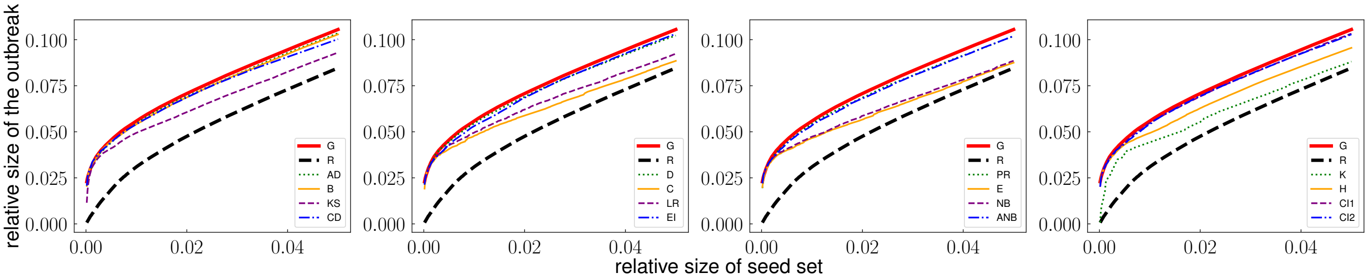

Figure 171: AS Oregon - p=1.0p_c

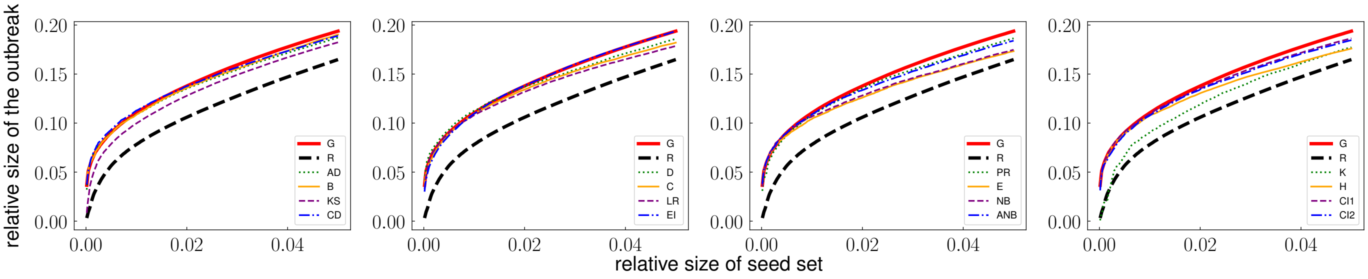

Figure 172: UChicago - p=1.0p_c

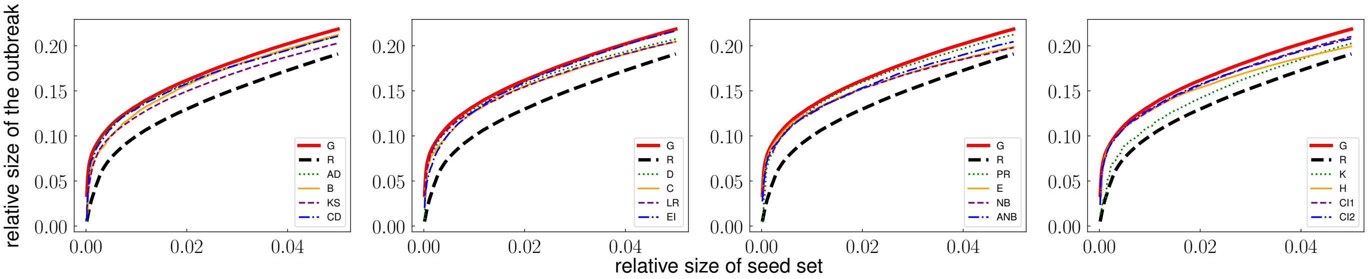

Figure 173: Princeton - p=1.0p_c

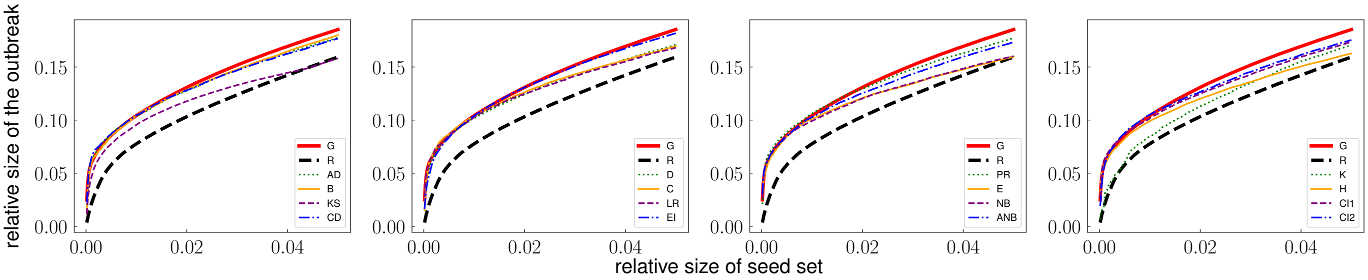

Figure 174: Carnegie - p=1.0p_c

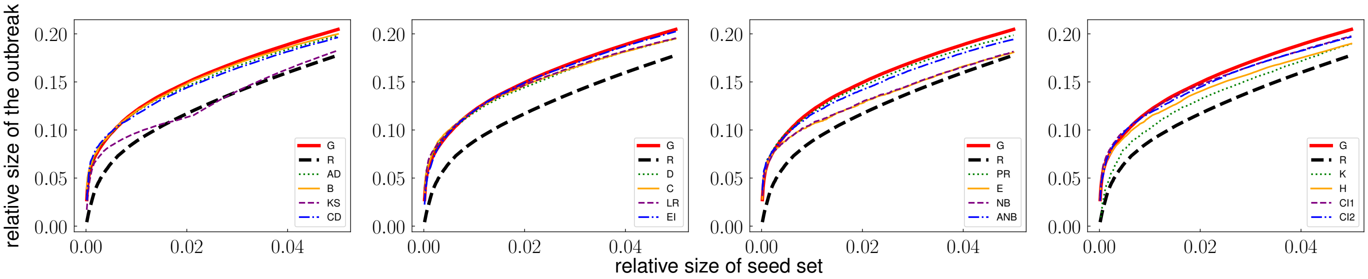

Figure 175: Tufts - p=1.0p_c

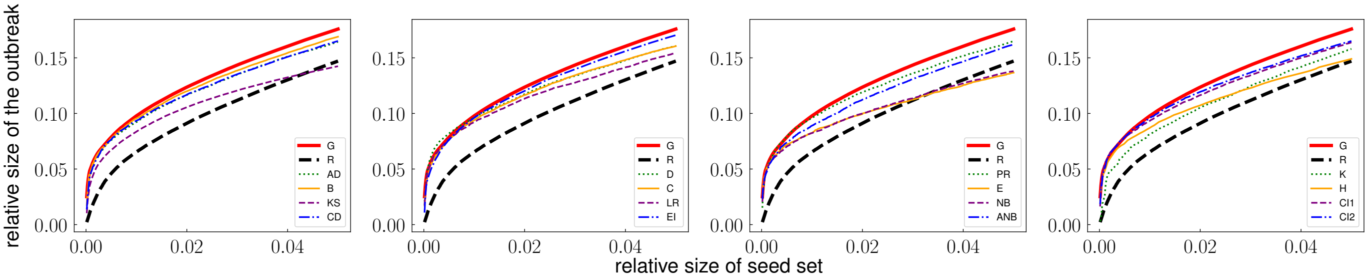

Figure 176: UC - p=1.0p_c

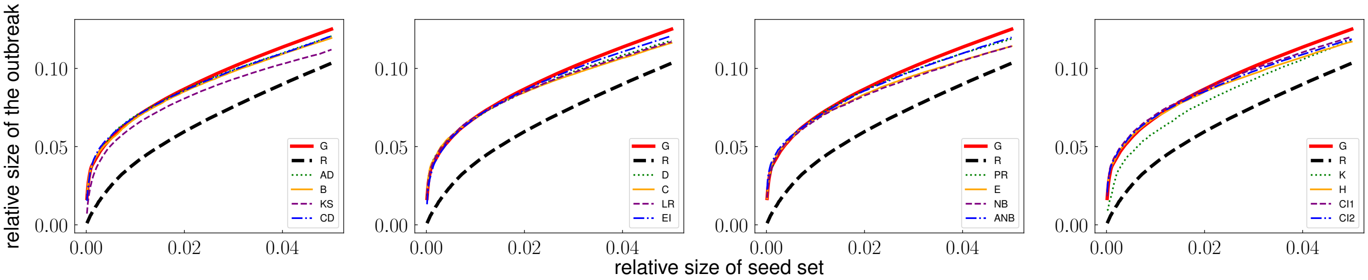

Figure 177: Wikipedia elections - p=1.0p_c

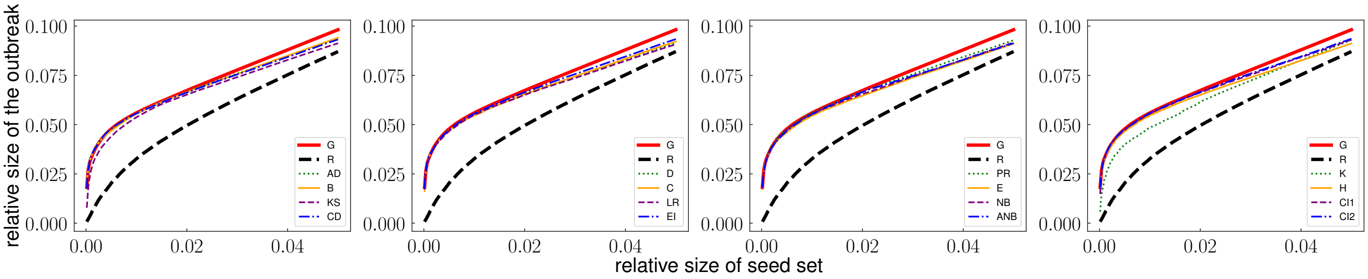

Figure 178: English - p=1.0p_c

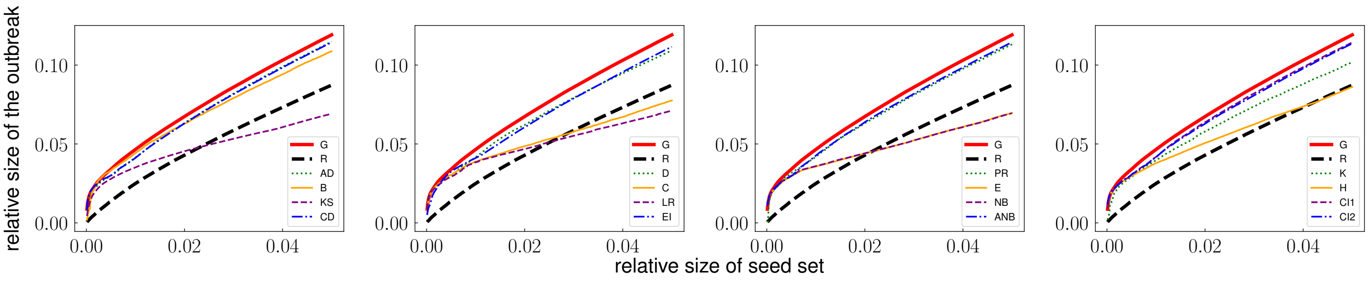

Figure 179: Gnutella, Aug. 9, 2002 - p=1.0p_c

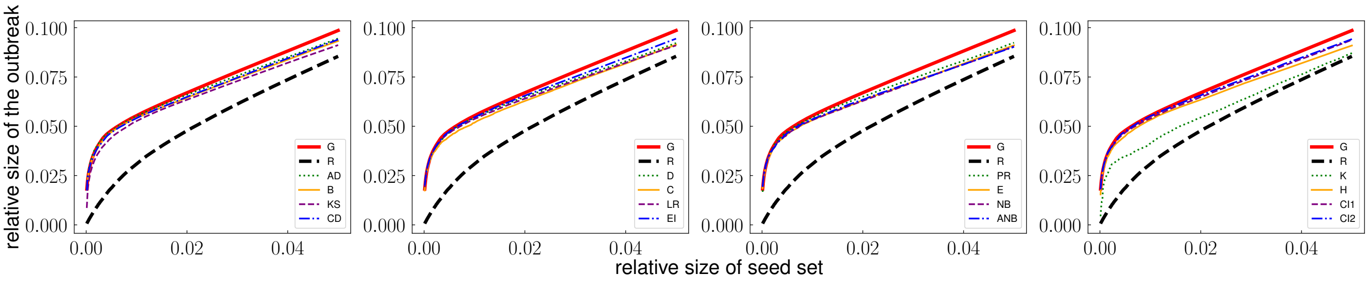

Figure 180: French - p=1.0p_c

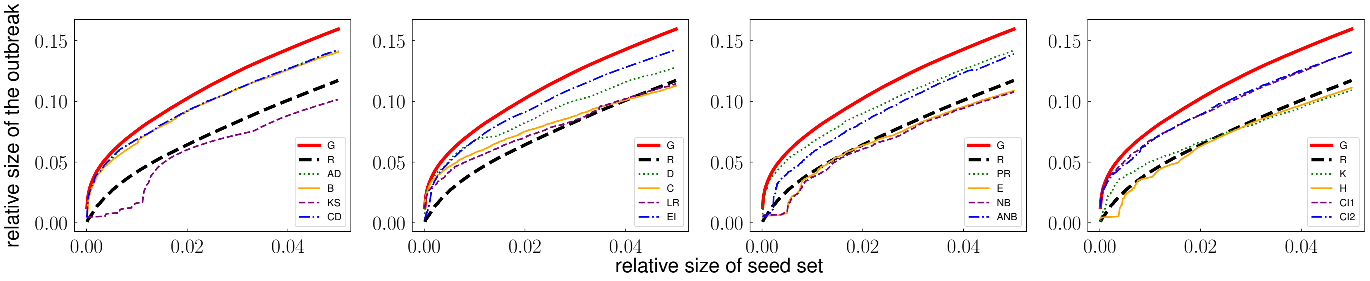

Figure 181: Hep-Th, 1993-2003 - p=1.0p_c

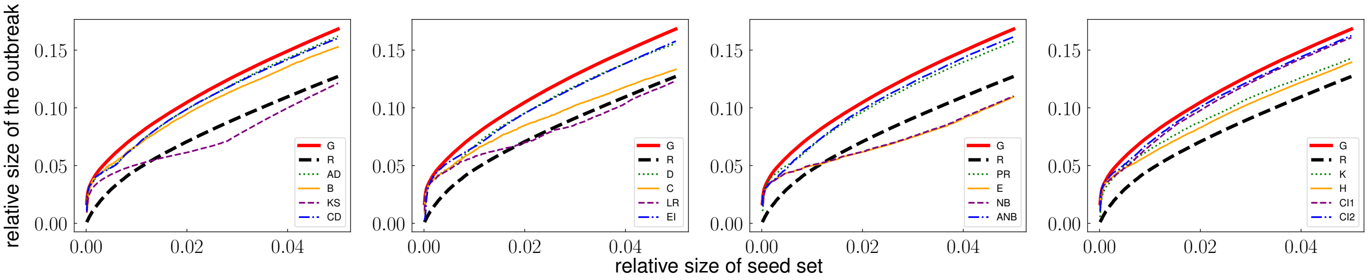

Figure 182: Gnutella, Aug. 6, 2002 - p=1.0p$_c$

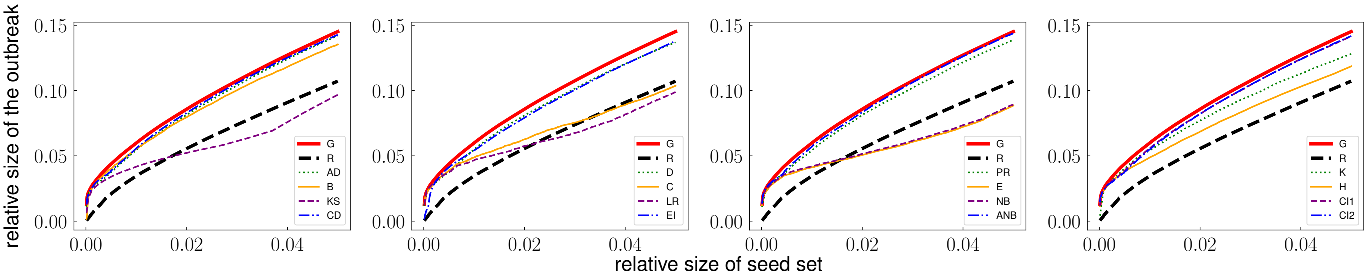

Figure 183: Gnutella, Aug. 5, 2002 - p=1.0p$_c$

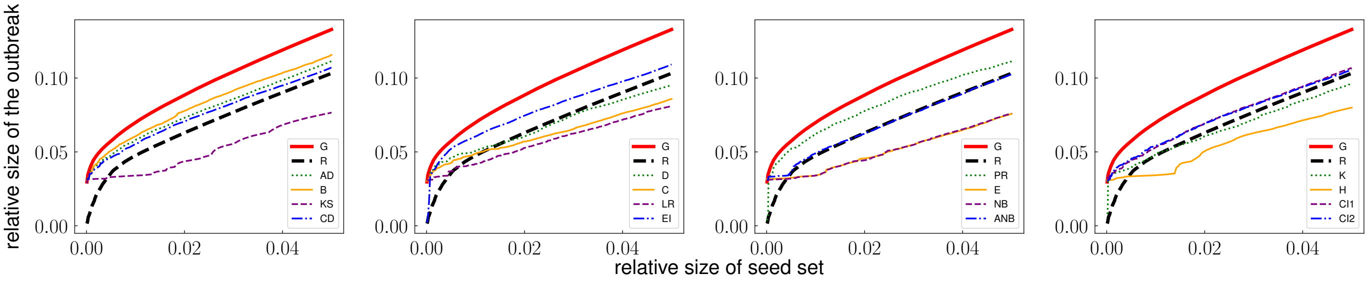

Figure 184: PGP - p=1.0p$_c$

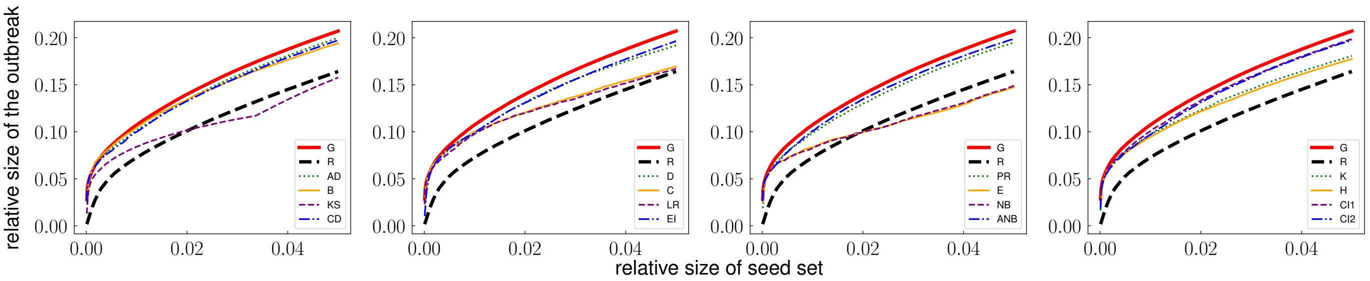

Figure 185: Gnutella, Aug. 4, 2002 - p=1.0p_c

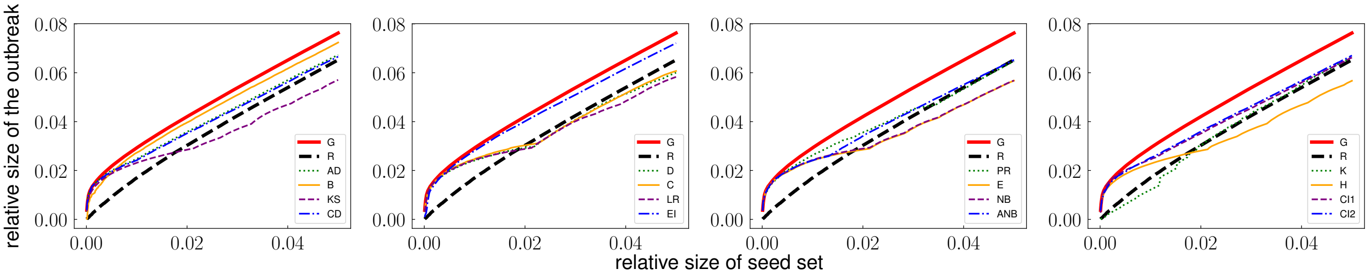

Figure 186: Hep-Ph, 1993-2003 - p=1.0p_c

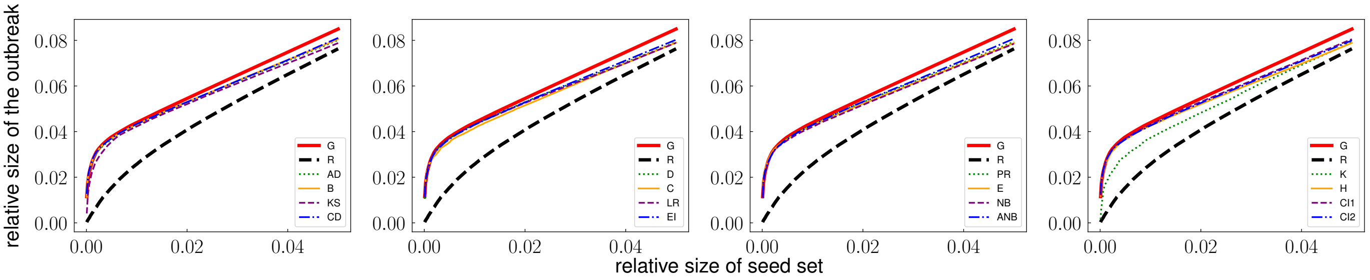

Figure 187: Spanish 1 - p=1.0p_c

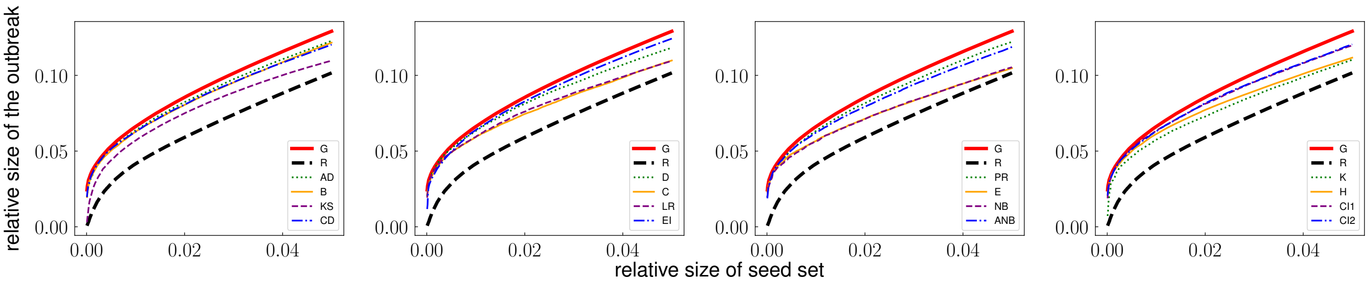

Figure 188: DBLP, citations - p=1.0p_c

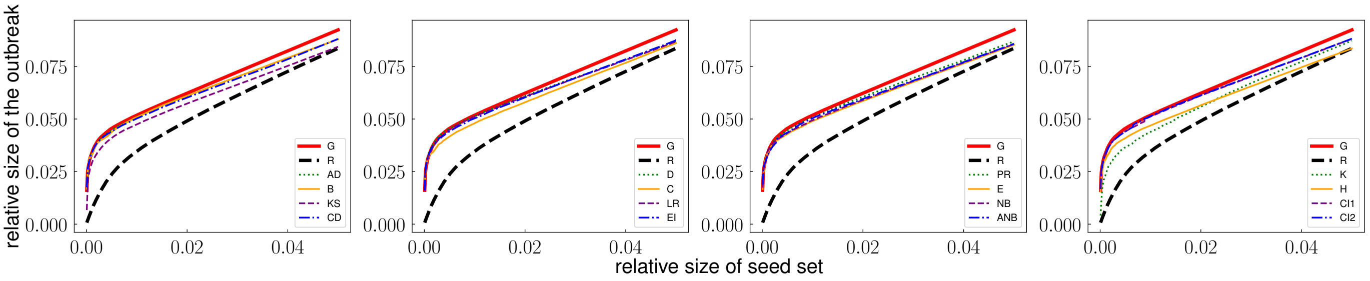

Figure 189: Spanish 2 - p=1.0p_c

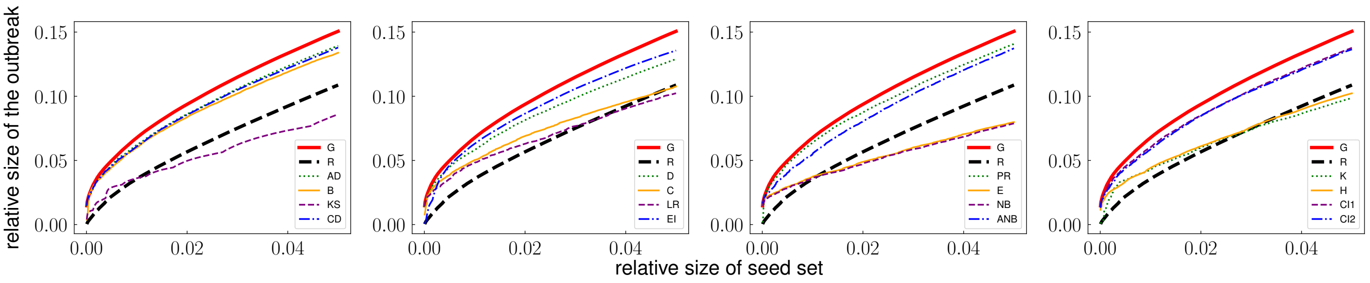

Figure 190: Cond-Mat, 1995-1999 - p=1.0p_c

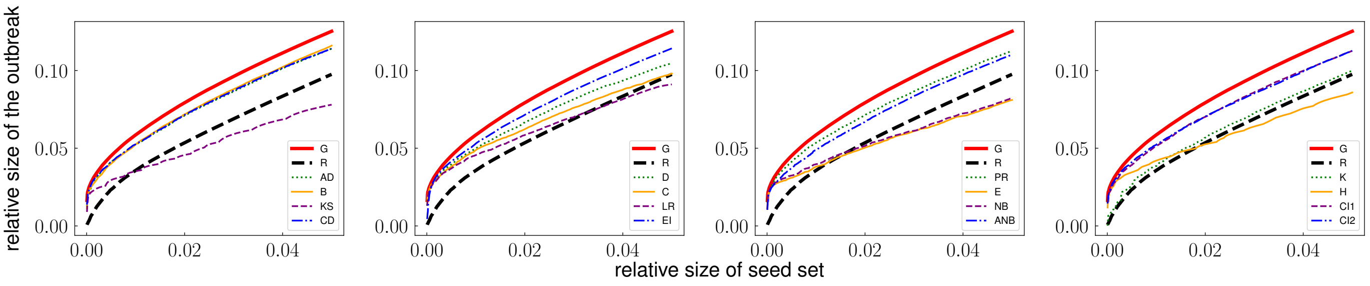

Figure 191: Astrophysics - p=1.0p_c

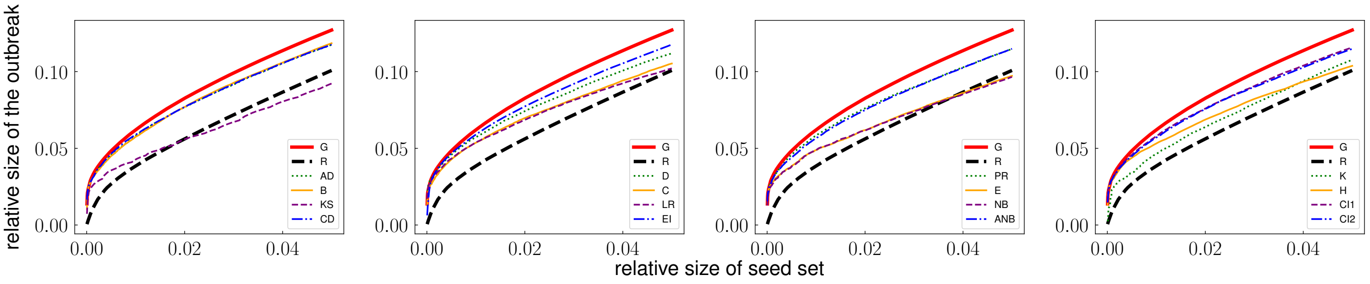

Figure 192: AstroPhys, 1993-2003 - p=1.0p_c

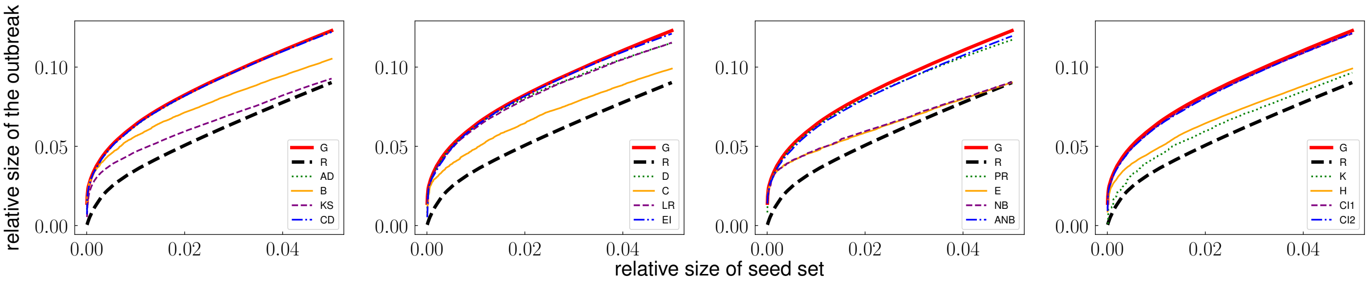

Figure 193: Marvel - p=1.0p_c

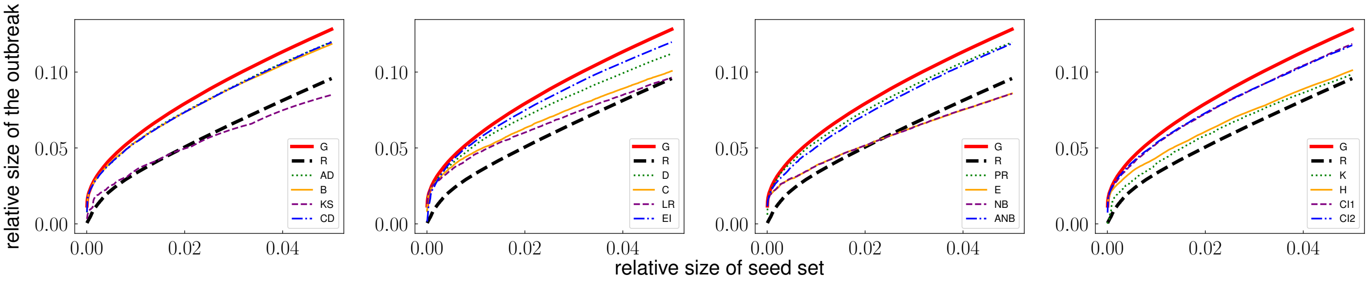

Figure 194: Cond-Mat, 1993-2003 - p=1.0p_c

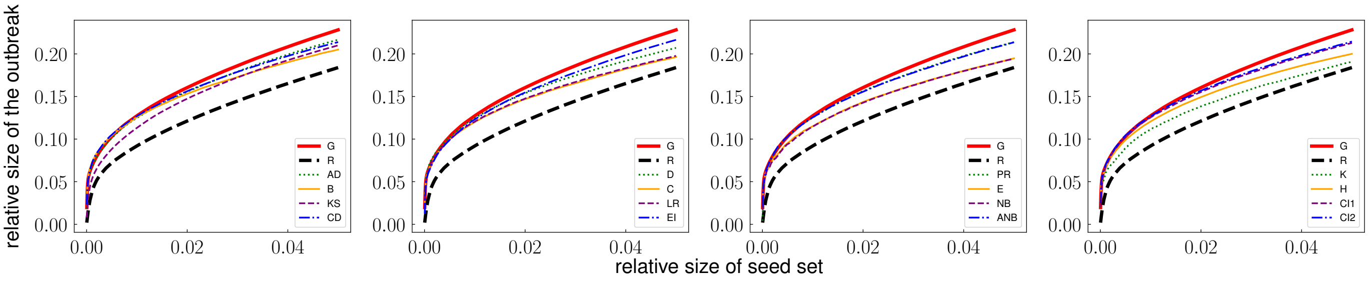

Figure 195: Gnutella, Aug. 25, 2002 - p=1.0p_c

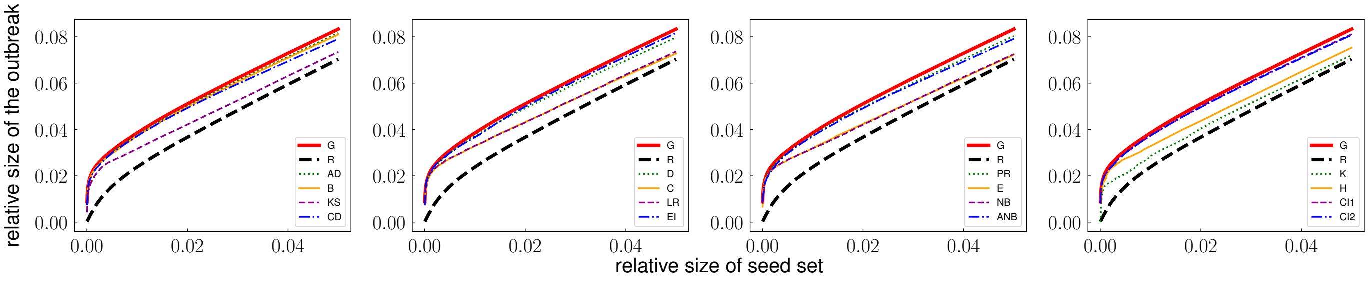

Figure 196: Internet - p=1.0p_c

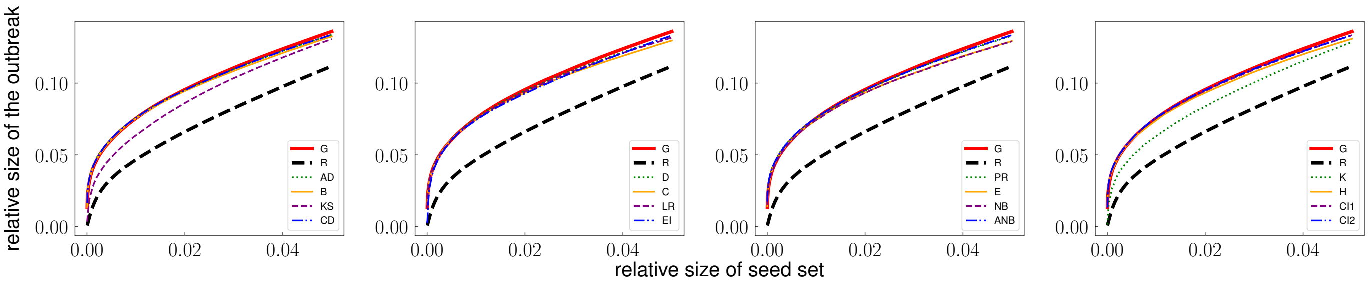

Figure 197: Thesaurus - p=1.0p_c

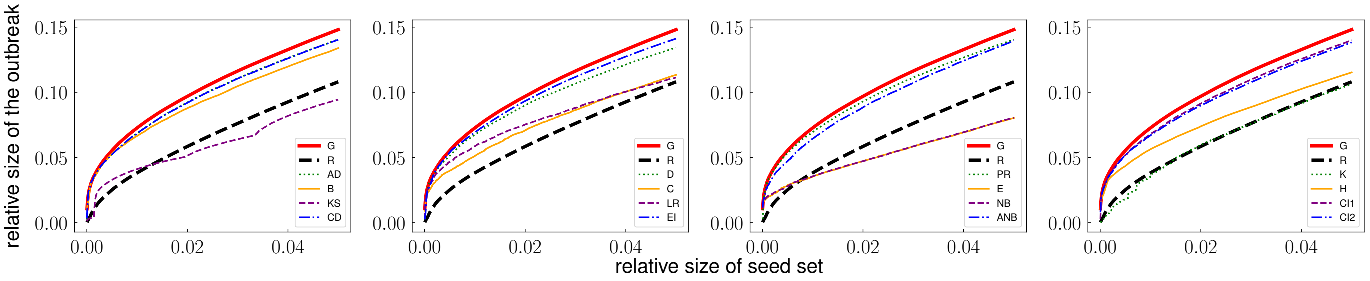

Figure 198: Cora - p=1.0p_c

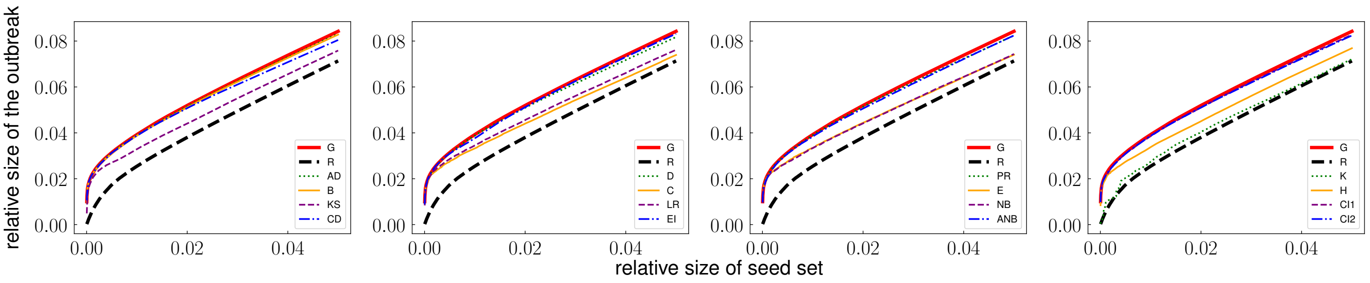

Figure 199: AS Caida - p=1.0p_c

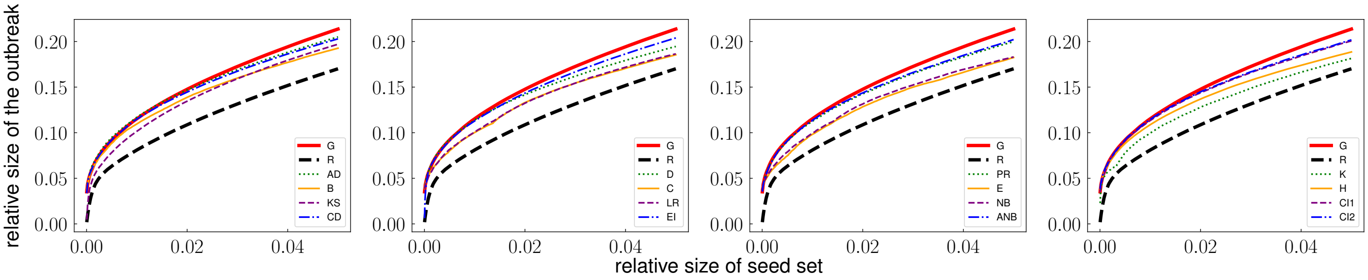

Figure 200: Gnutella, Aug. 24, 2002 - p=1.0p_c

2.1.3 Supercritical Regime

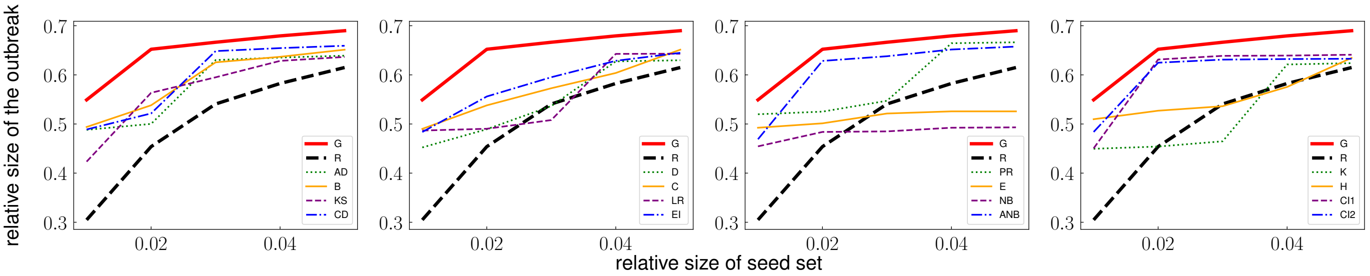

Figure 201: Political books - p=2.0p$_c$

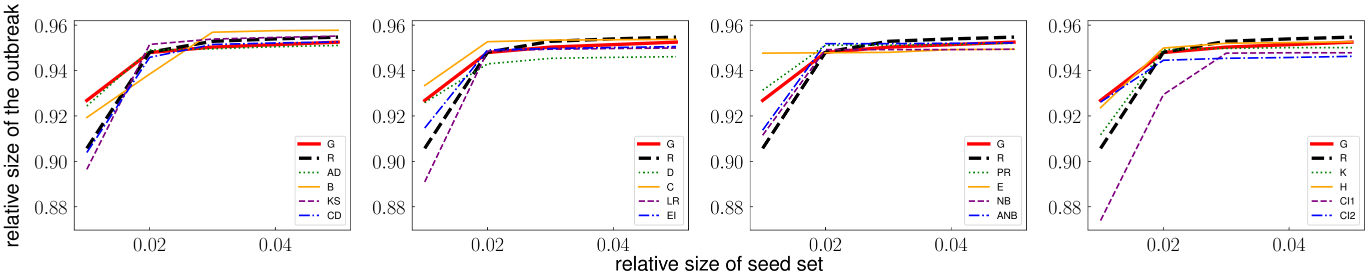

Figure 202: College football - p=2.0p$_c$

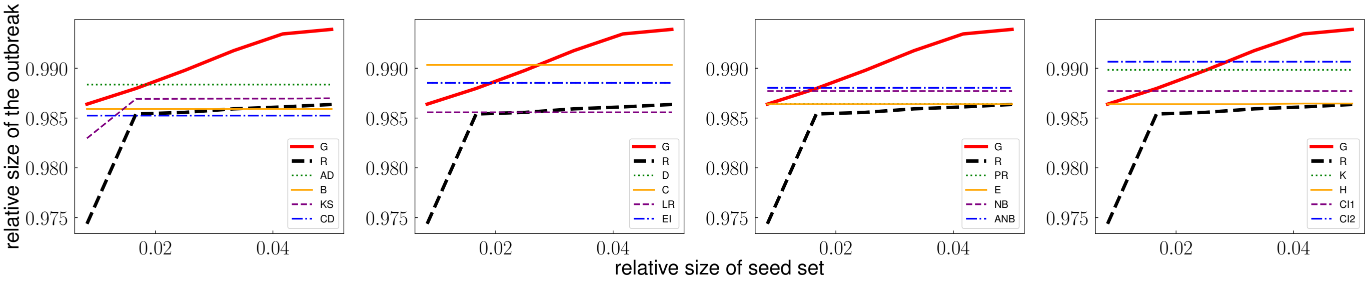

Figure 203: S208 - p=2.0p$_c$

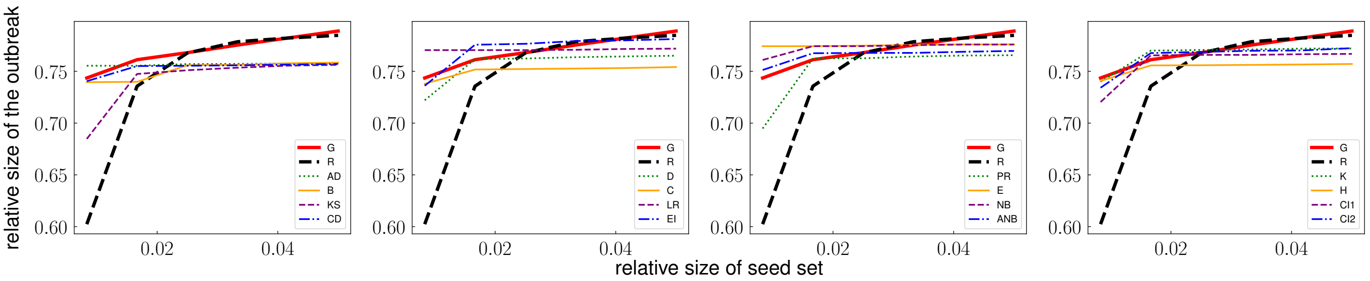

Figure 204: High school, 2011 - p=2.0p_c

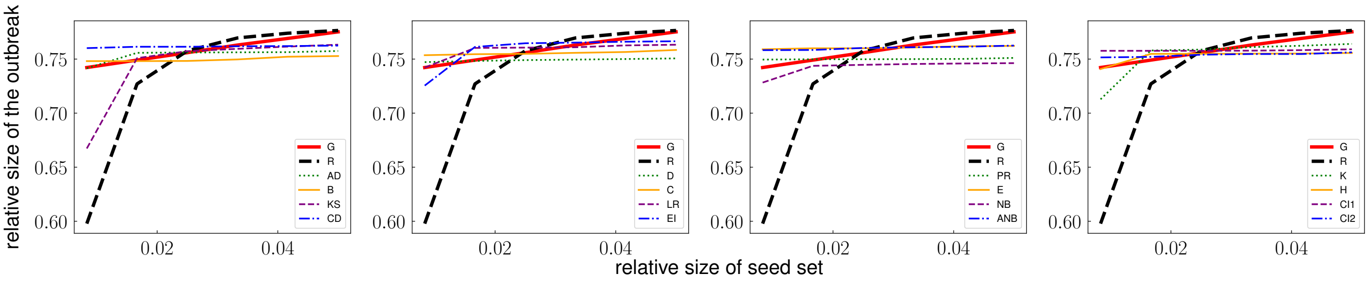

Figure 205: Bay Dry - p=2.0p_c

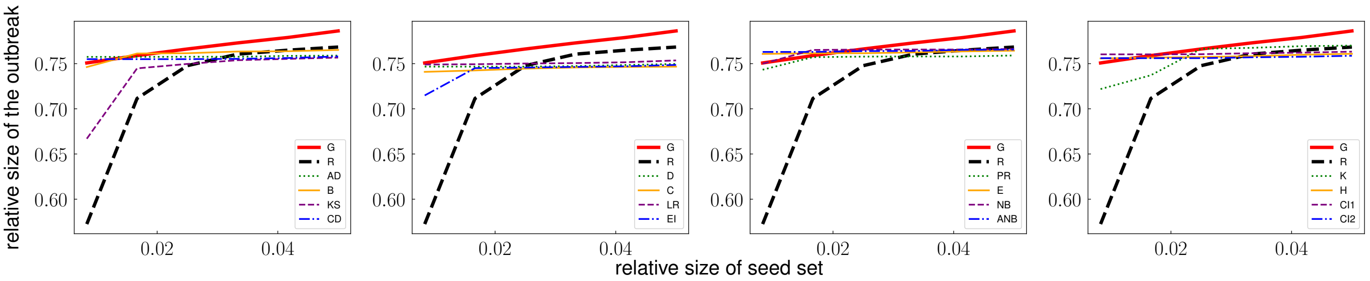

Figure 206: Bay Wet - p=2.0p_c

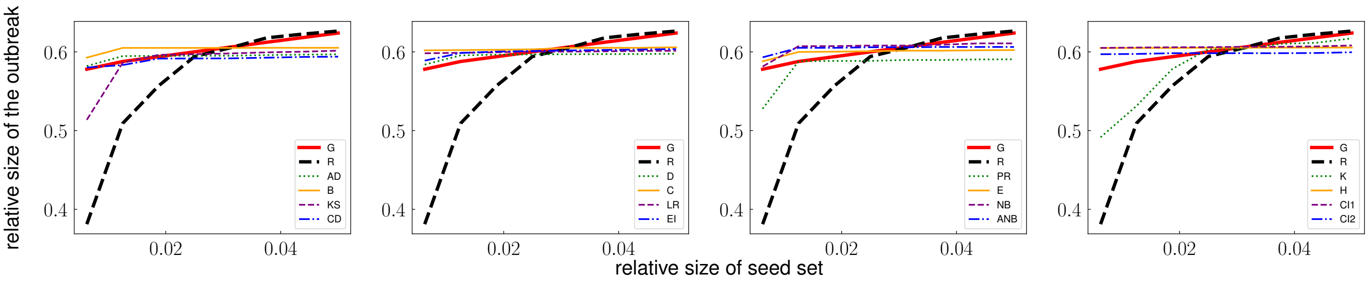

Figure 207: Radoslaw Email - p=2.0p_c

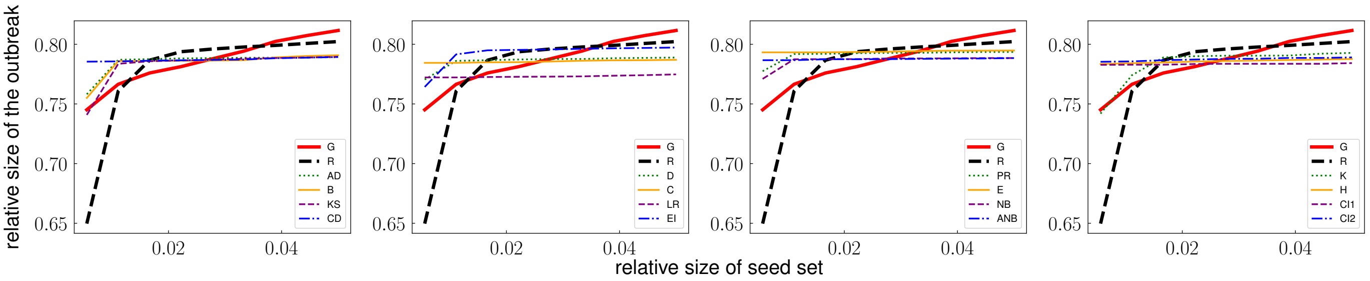

Figure 208: High school, 2012 - p=2.0p_c

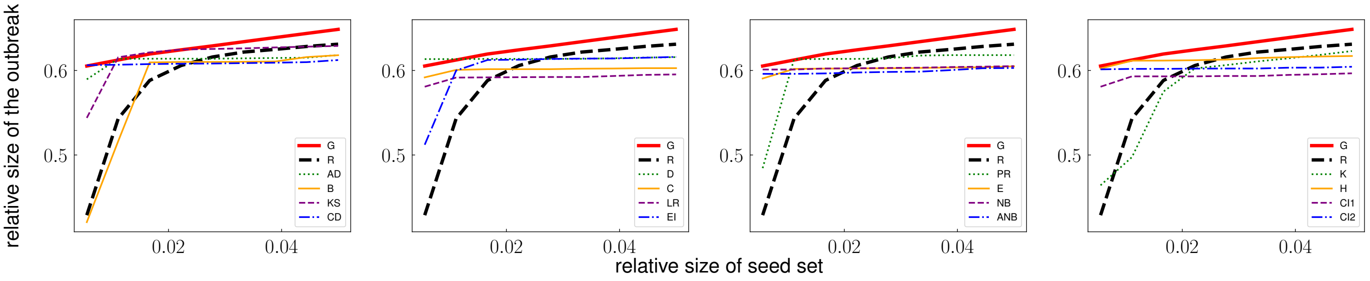

Figure 209: Little Rock Lake - p=2.0p_c

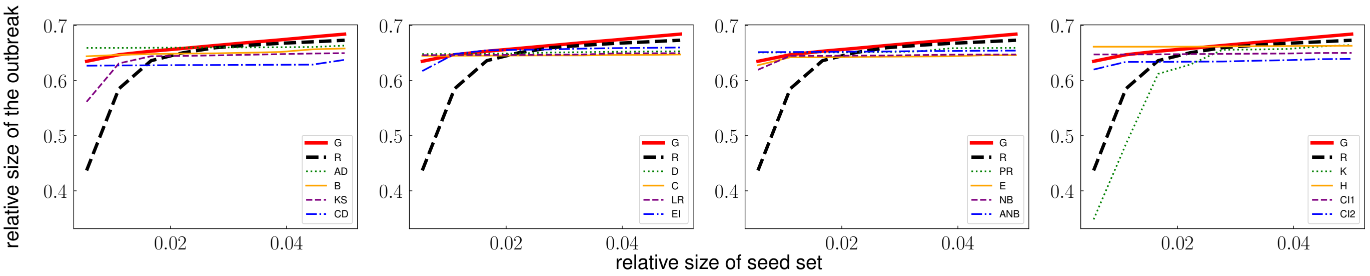

Figure 210: Jazz - p=2.0p_c

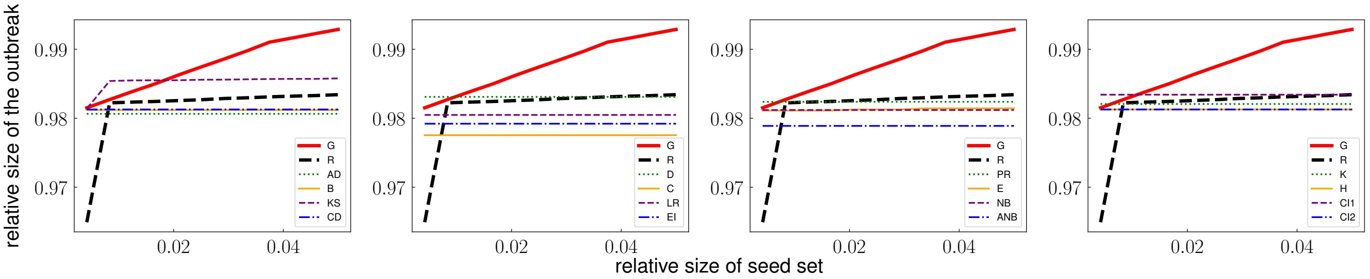

Figure 211: S420 - p=2.0p_c

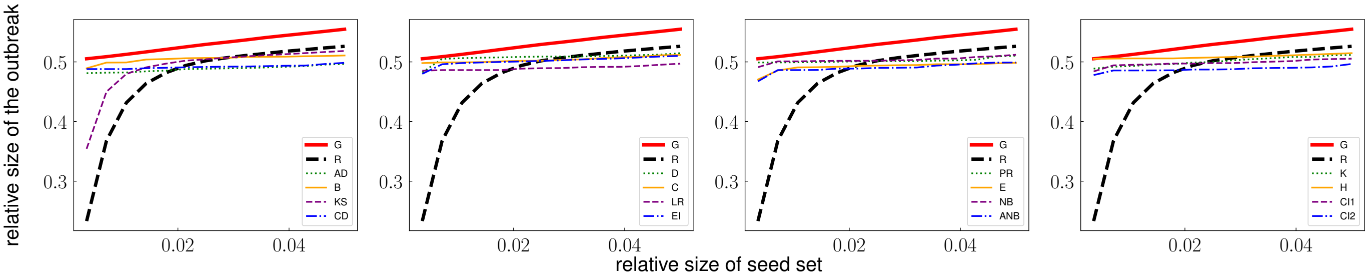

Figure 212: C. Elegans, neural - p=2.0p_c

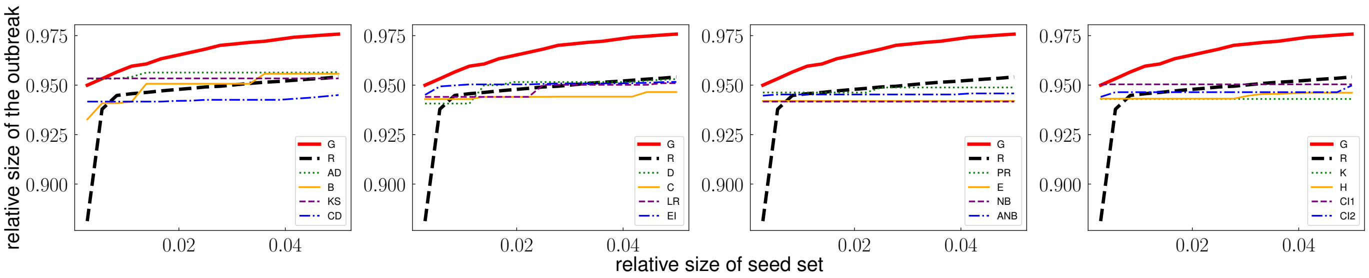

Figure 213: Network Science - p=2.0p_c

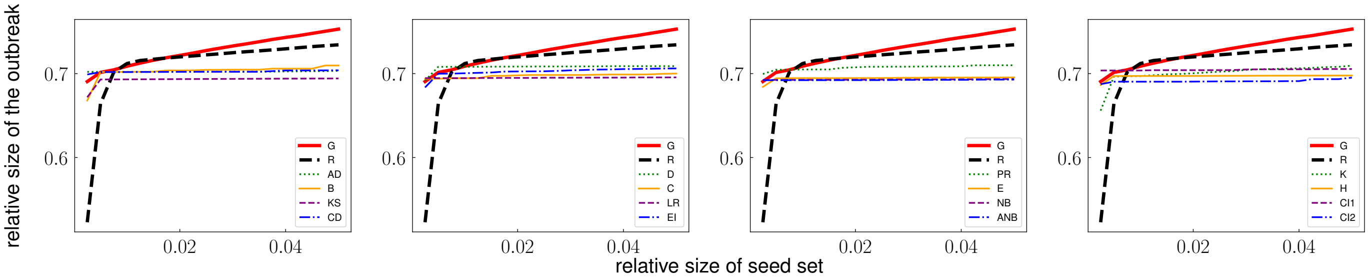

Figure 214: Dublin - p=2.0p_c

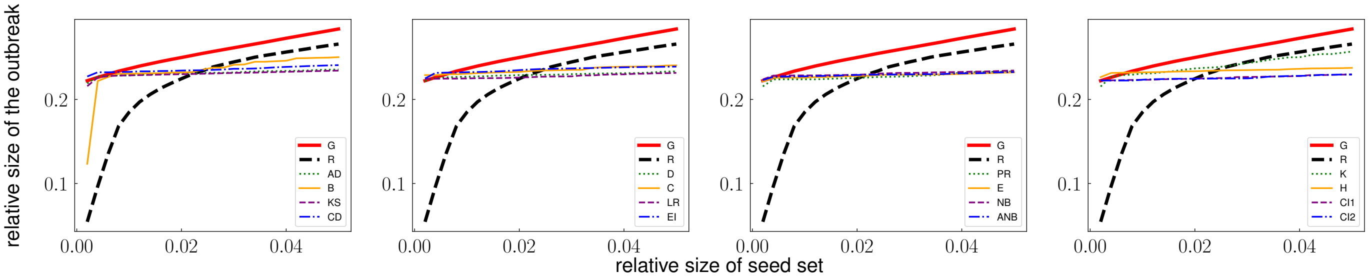

Figure 215: US Air Trasportation - p=2.0p_c

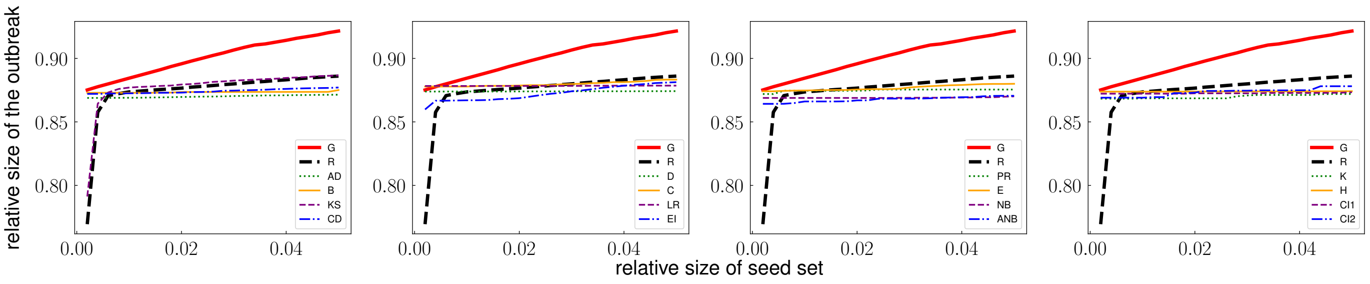

Figure 216: S838 - p=2.0p$_c$

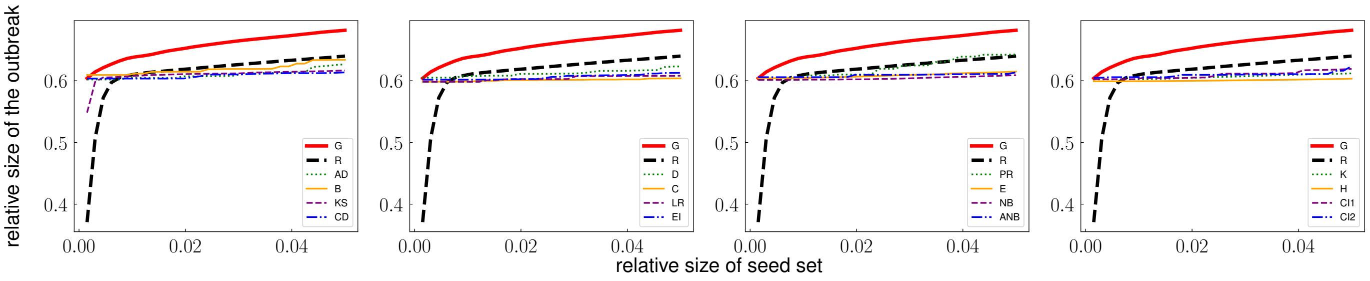

Figure 217: Yeast, transcription - p=2.0p$_c$

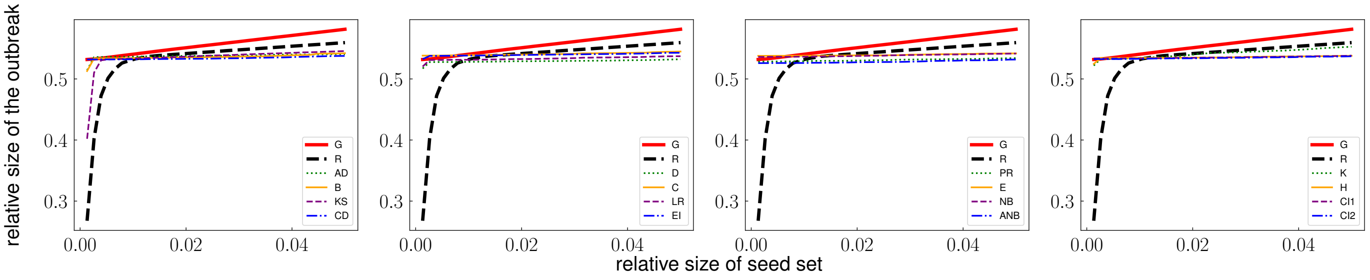

Figure 218: Caltech - p=2.0p$_c$

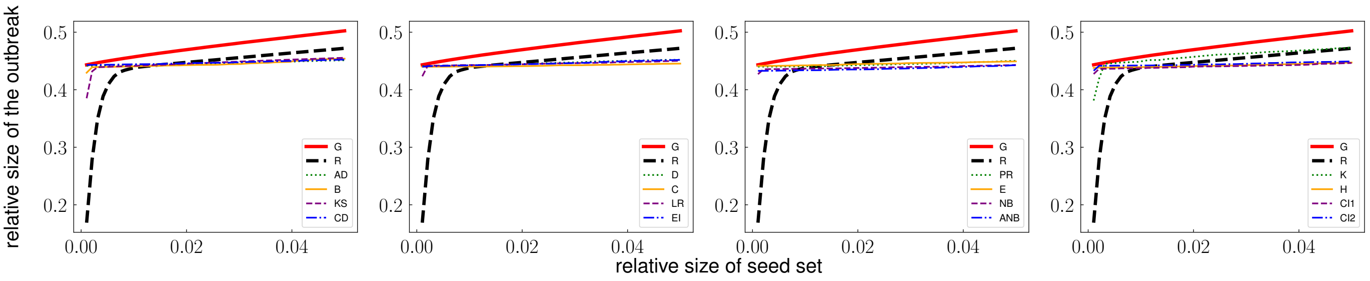

Figure 219: Reed - p=2.0p_c

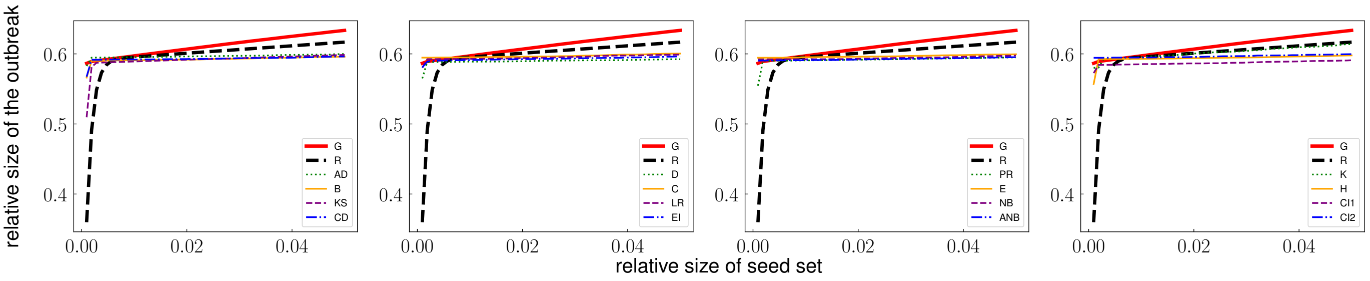

Figure 220: Mouse retina - p=2.0p_c

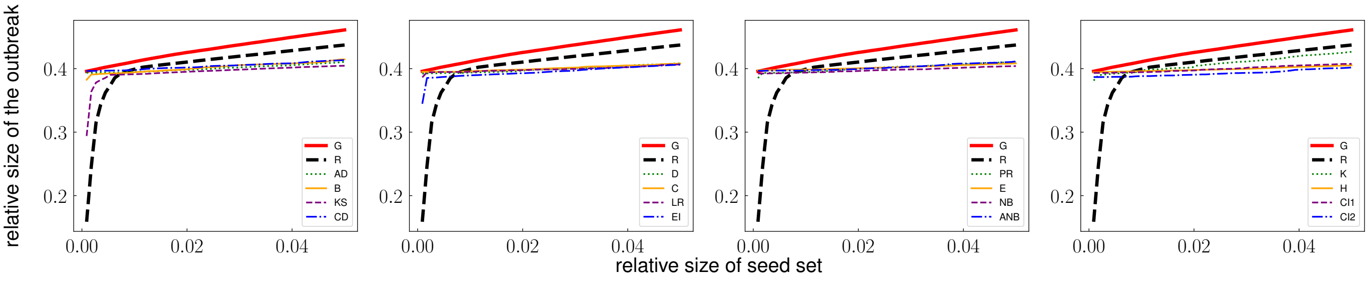

Figure 221: URV email - p=2.0p_c

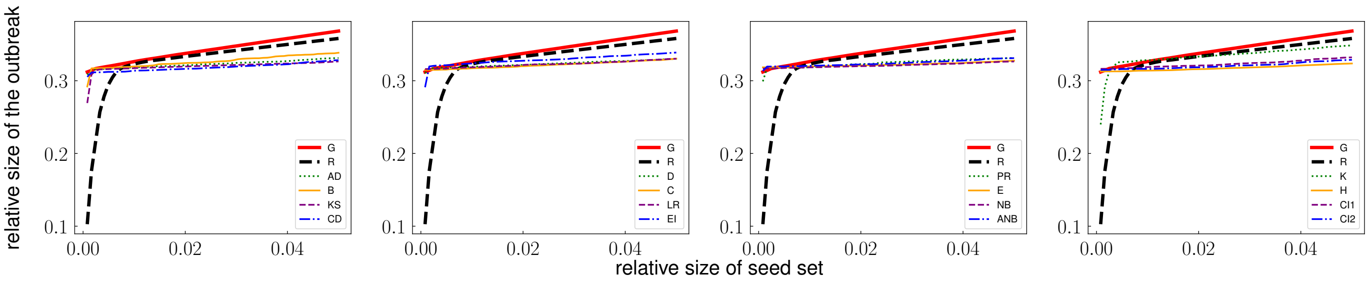

Figure 222: Political blogs - p=2.0p$_c$

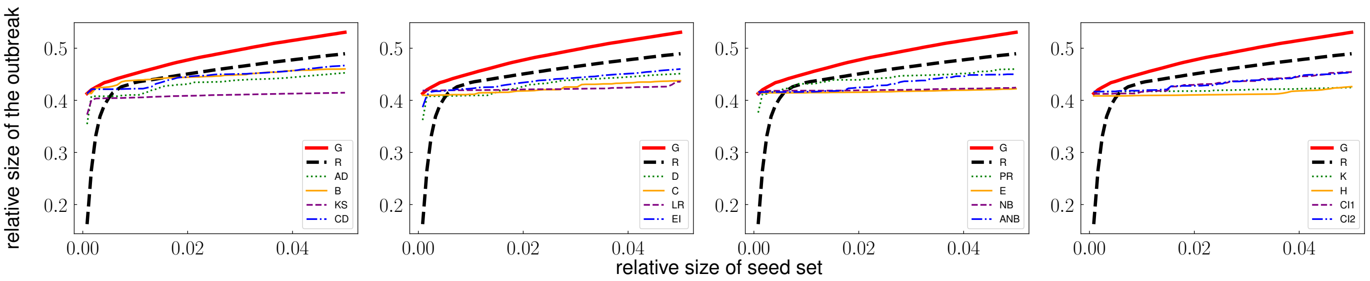

Figure 223: Air traffic - p=2.0p$_c$

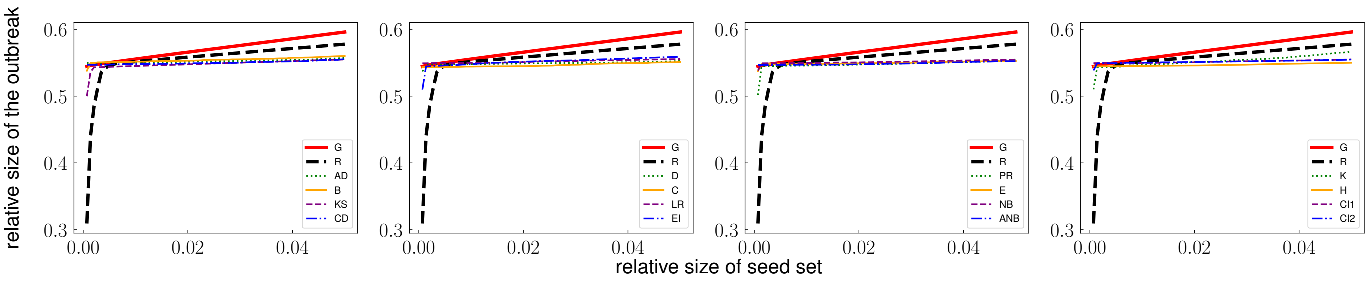

Figure 224: Haverford - p=2.0p$_c$

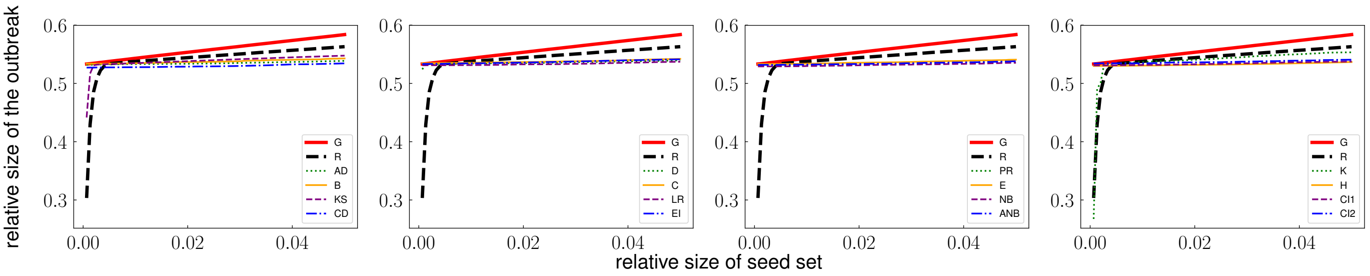

Figure 225: Simmons - p=2.0p_c

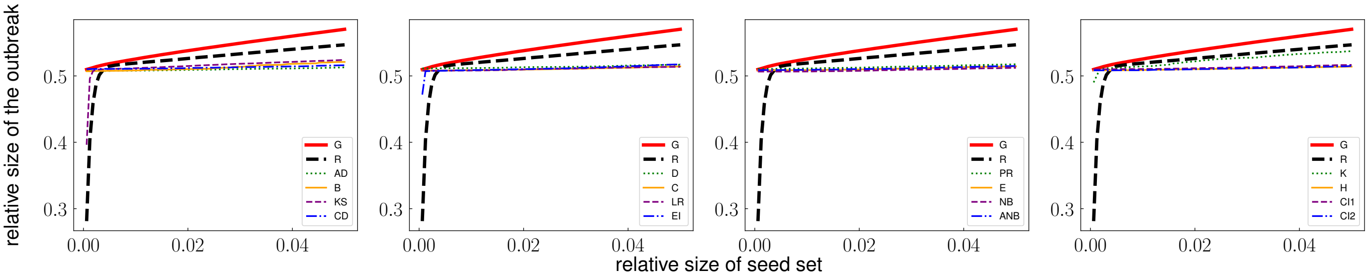

Figure 226: Swarthmore - p=2.0p_c

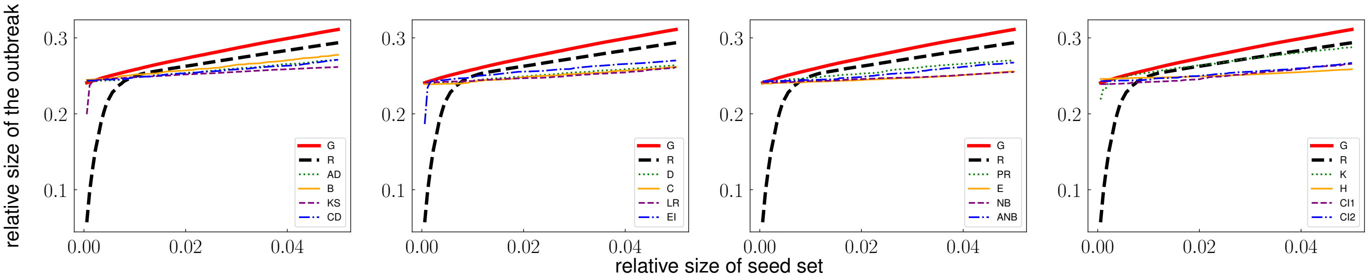

Figure 227: Petster, hamster - p=2.0p_c

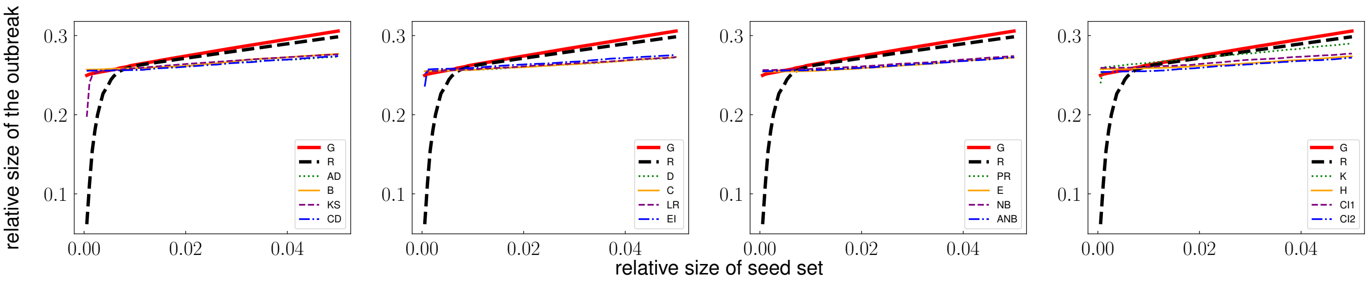

Figure 228: UC Irvine - p=2.0p_c

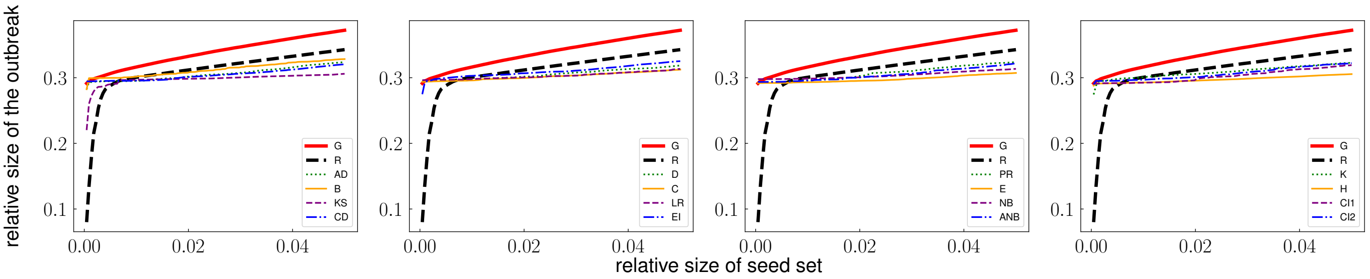

Figure 229: Yeast, protein - p=2.0p_c

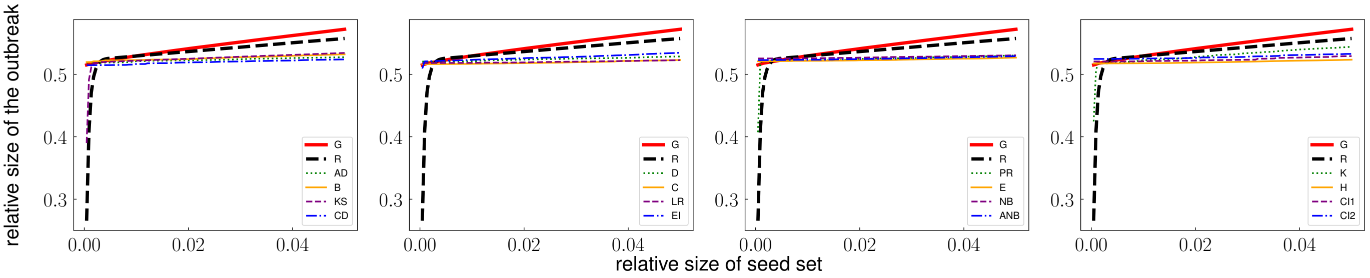

Figure 230: Amherst - p=2.0p_c

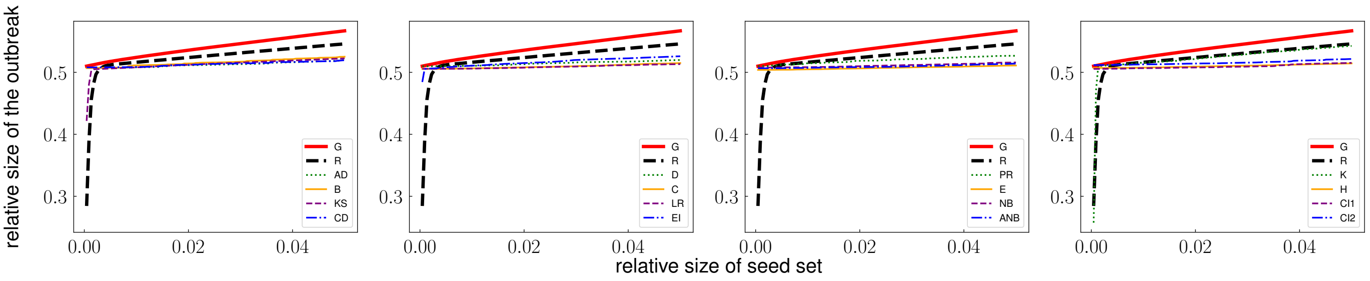

Figure 231: Bowdoin - p=2.0p_c

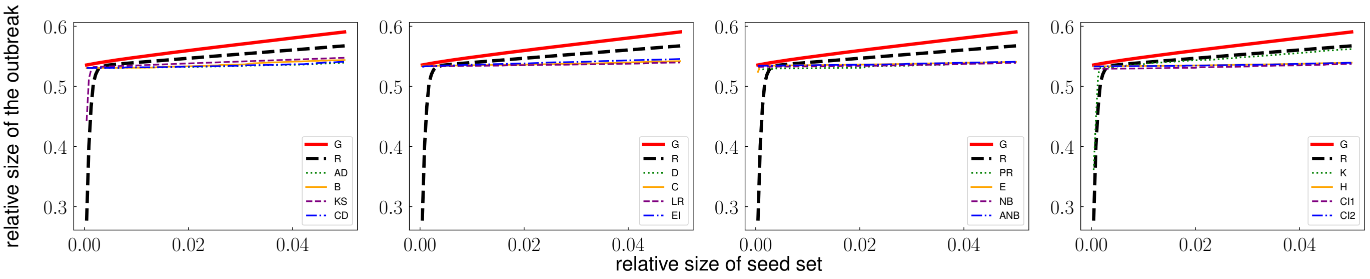

Figure 232: Hamilton - p=2.0p_c

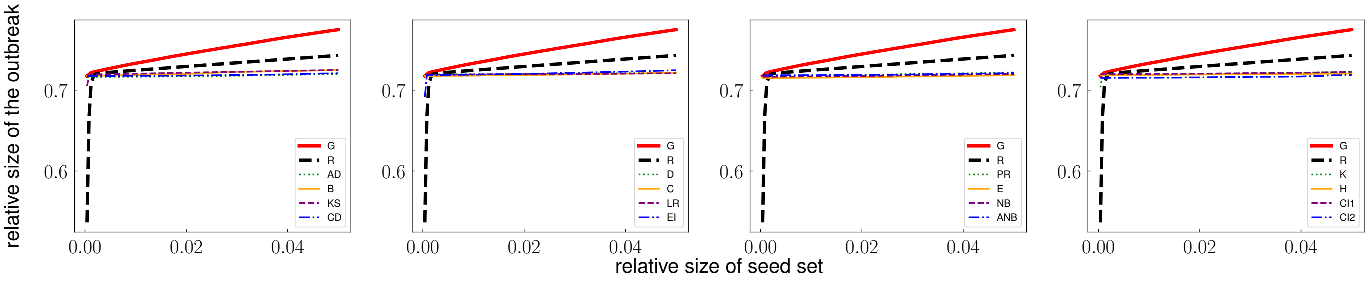

Figure 233: Adolescent health - p=2.0p_c

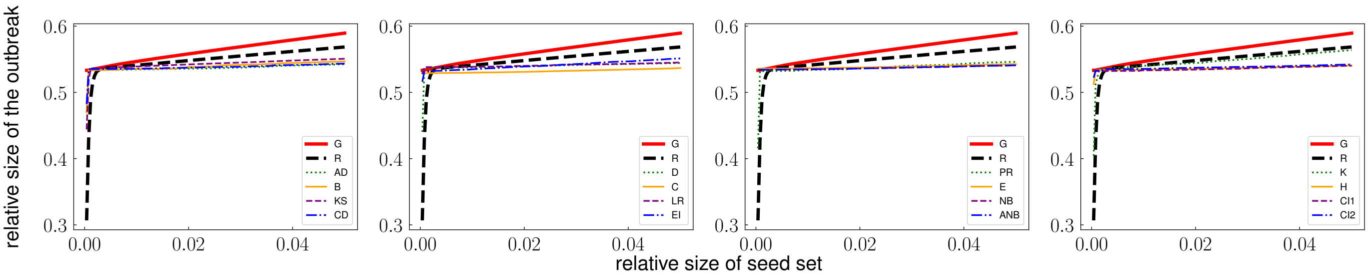

Figure 234: Trinity - p=2.0p_c

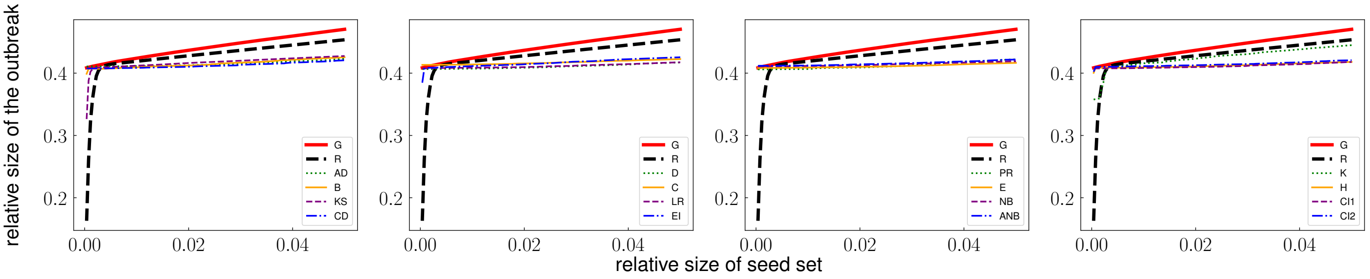

Figure 235: USFCA - p=2.0p_c

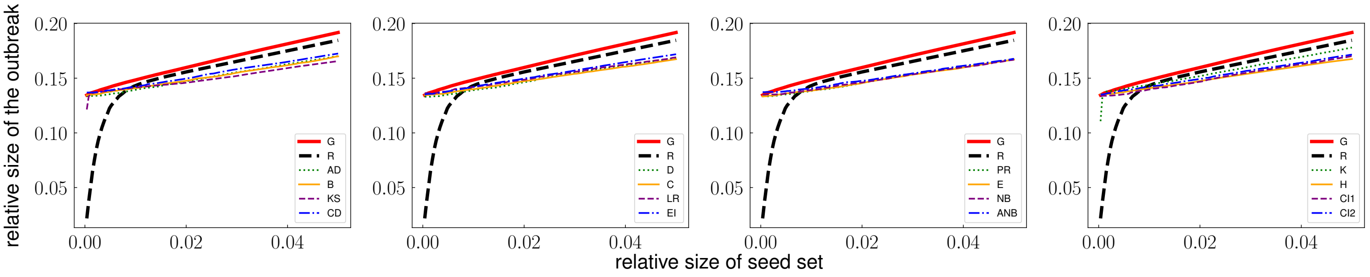

Figure 236: Japanese - p=2.0p_c

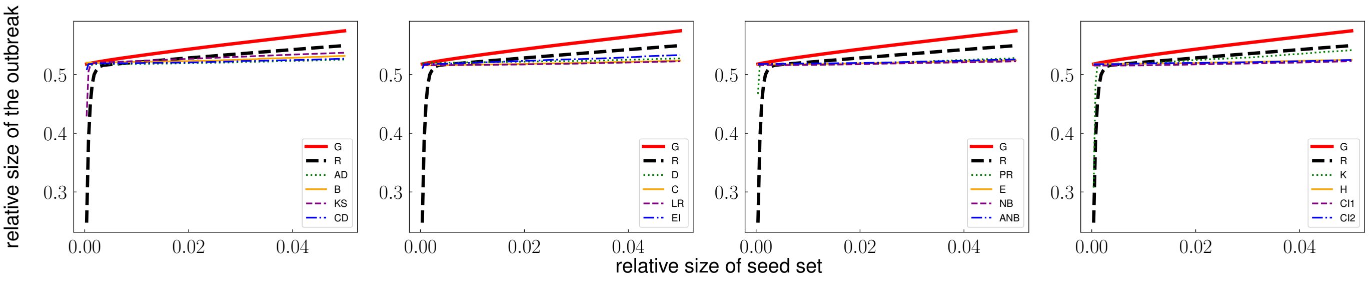

Figure 237: Williams - p=2.0p_c

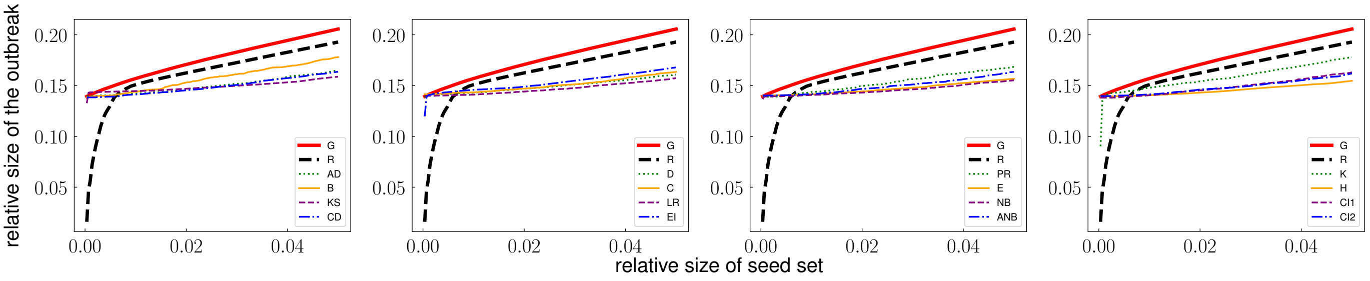

Figure 238: Open flights - p=2.0p_c

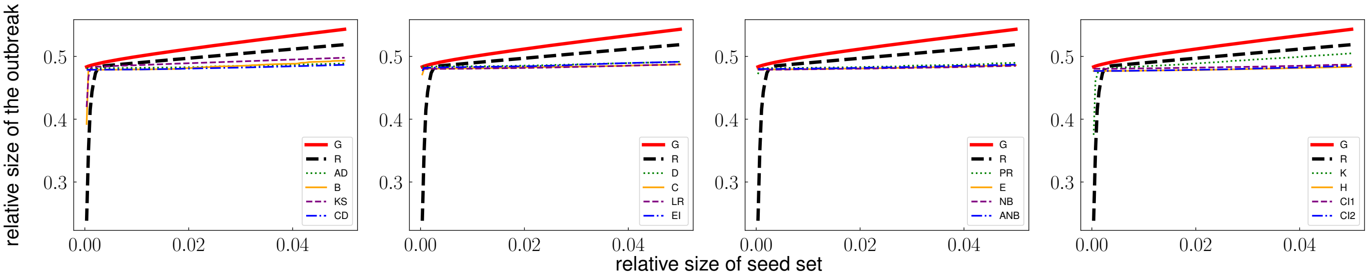

Figure 239: Oberlin - p=2.0p_c

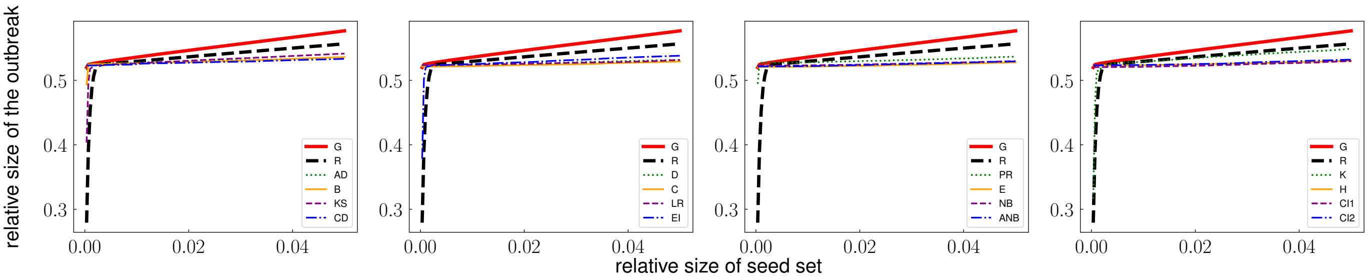

Figure 240: Smith - p=2.0p_c

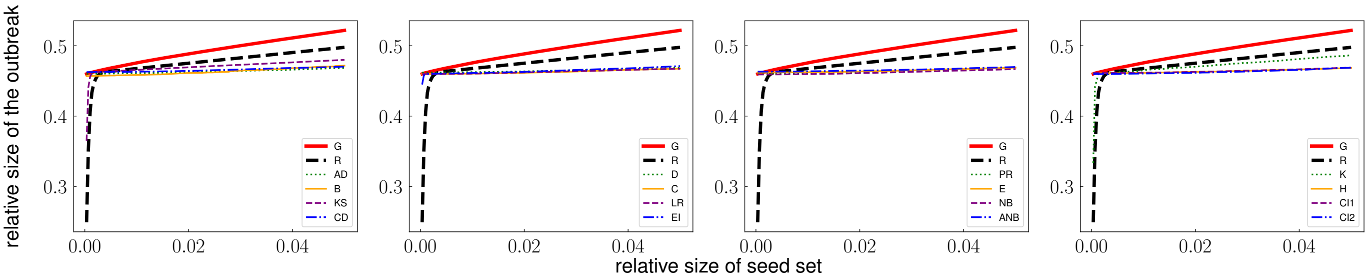

Figure 241: Wellesley - p=2.0p_c

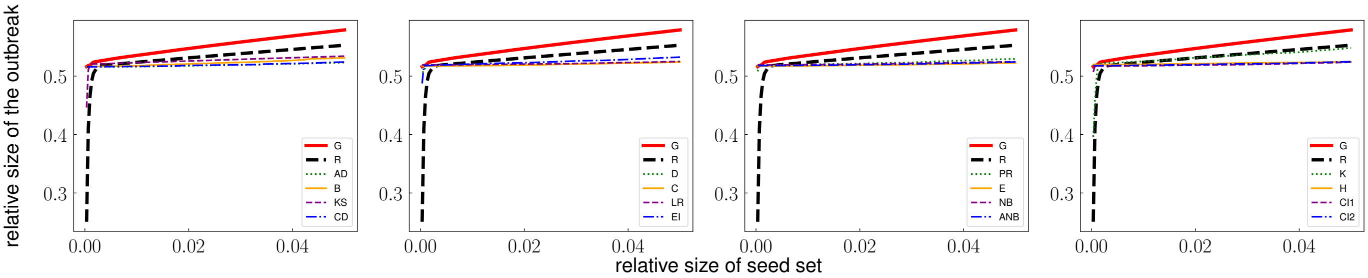

Figure 242: Vassar - p=2.0p_c

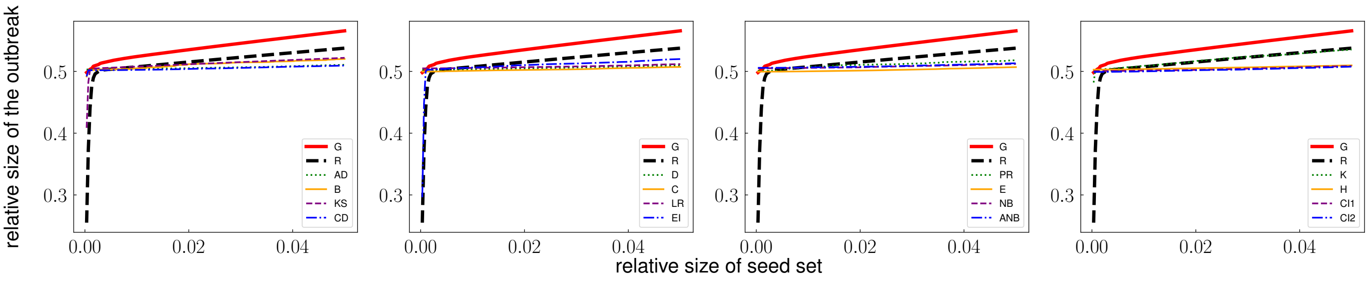

Figure 243: Middlebury - p=2.0p_c

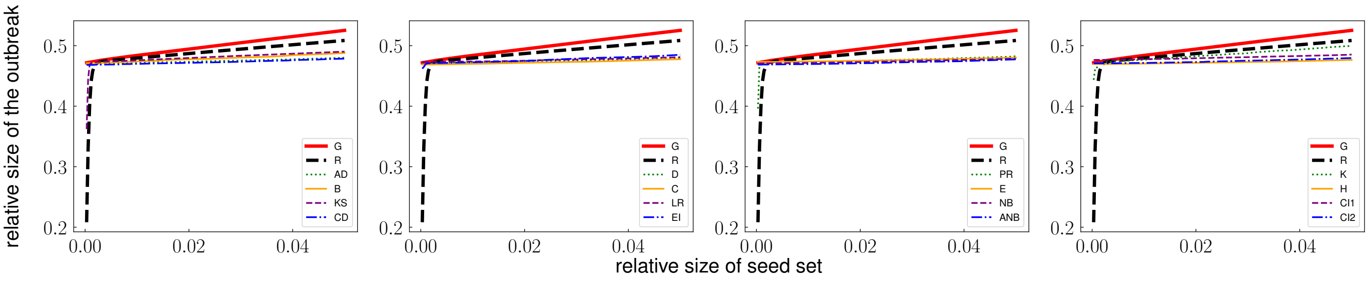

Figure 244: Pepperdine - p=2.0p_c

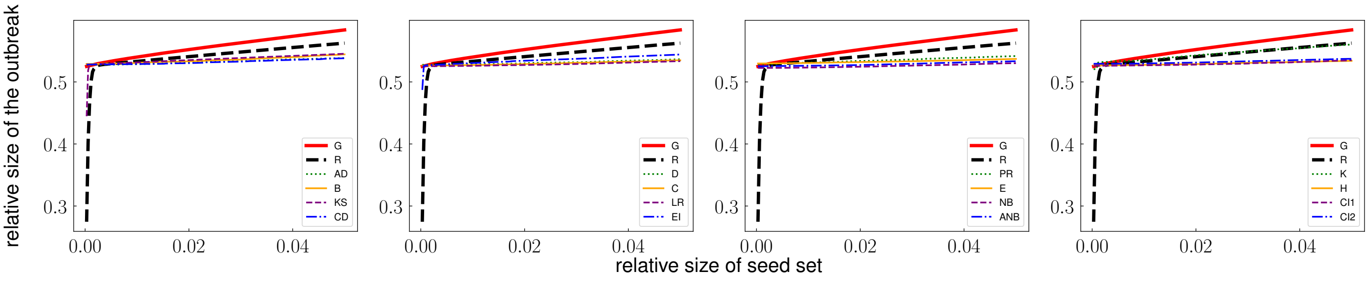

Figure 245: Colgate - p=2.0p_c

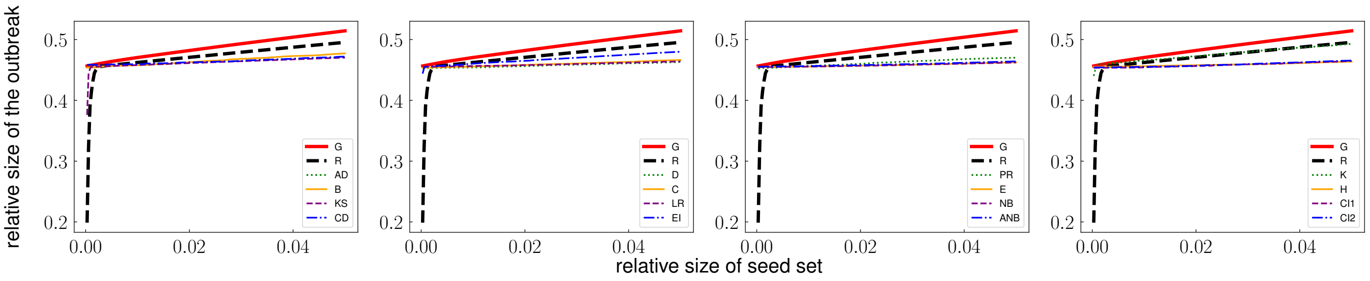

Figure 246: Santa - p=2.0p_c

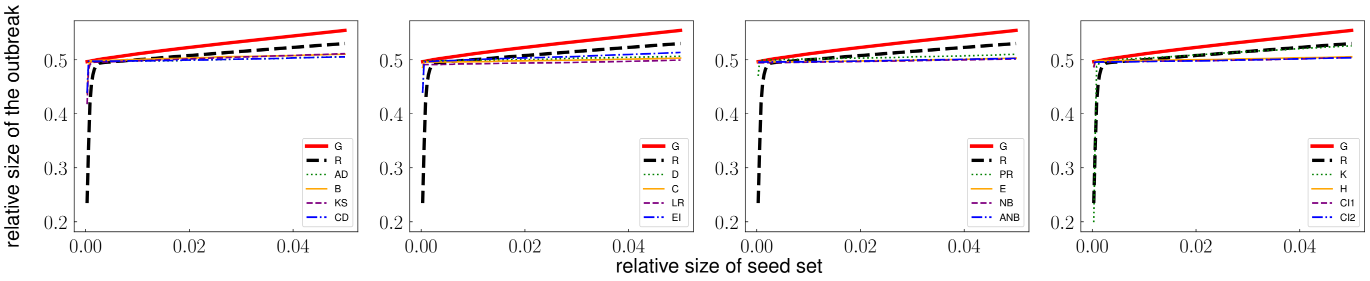

Figure 247: Wesleyan - p=2.0p_c

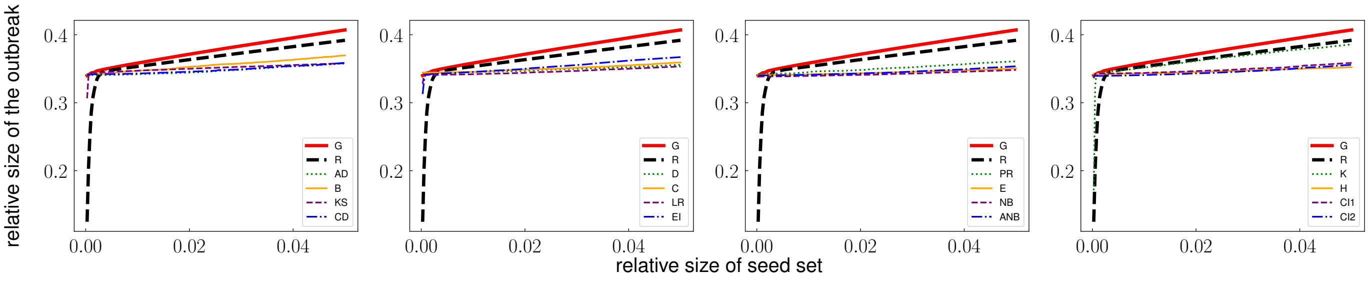

Figure 248: Mich - p=2.0p_c

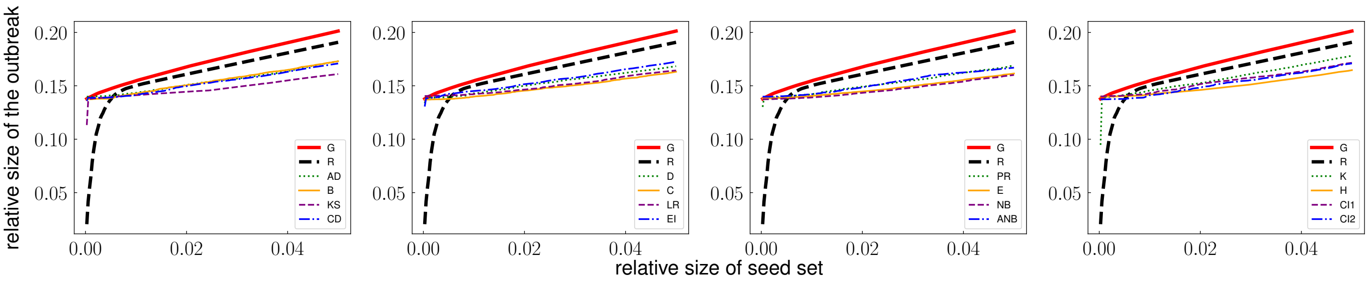

Figure 249: Bitcoin Alpha - p=2.0p$_c$

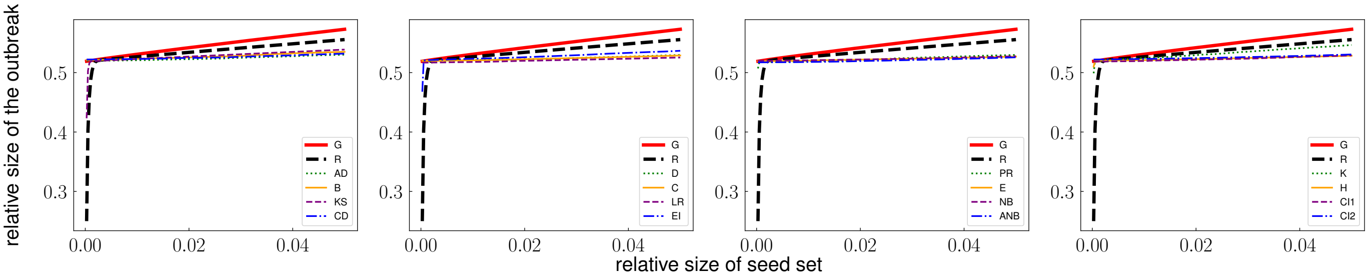

Figure 250: Bucknell - p=2.0p$_c$

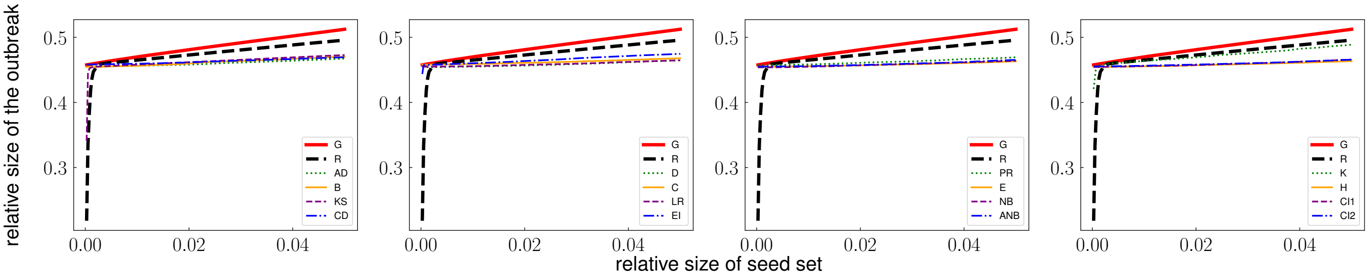

Figure 251: Brandeis - p=2.0p$_c$

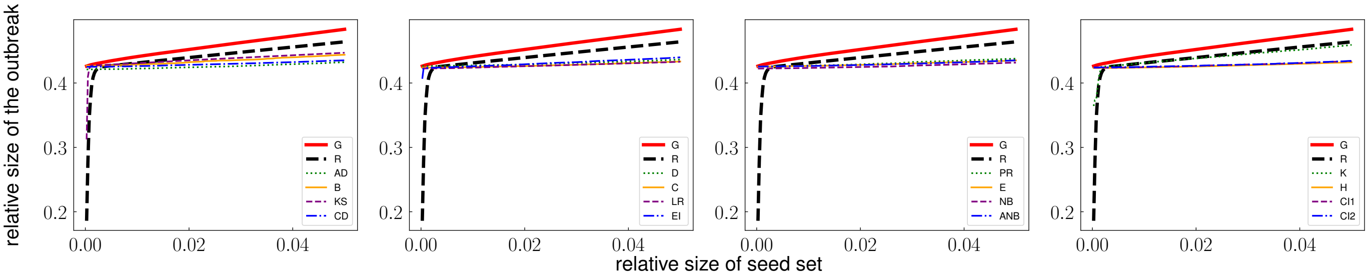

Figure 252: Howard - p=2.0p_c

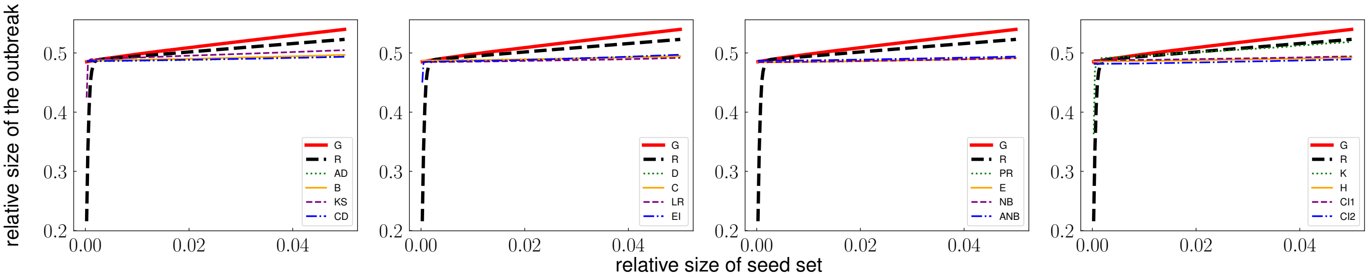

Figure 253: Rice - p=2.0p_c

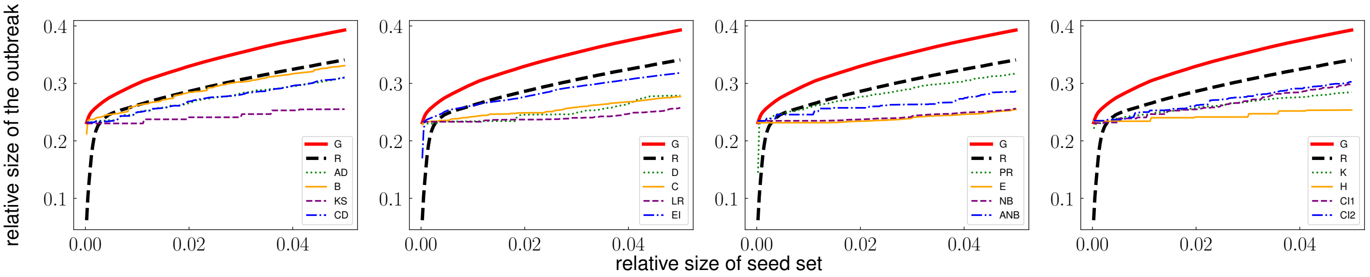

Figure 254: GR-QC, 1993-2003 - p=2.0p_c

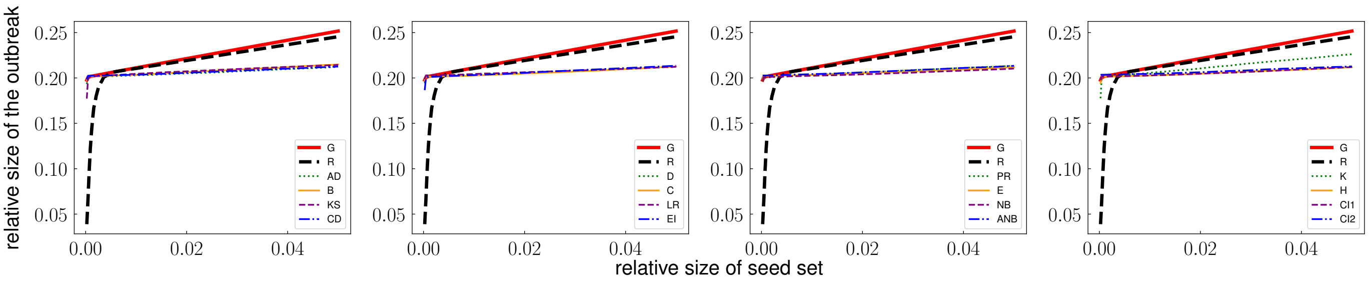

Figure 255: Tennis - p=2.0p_c

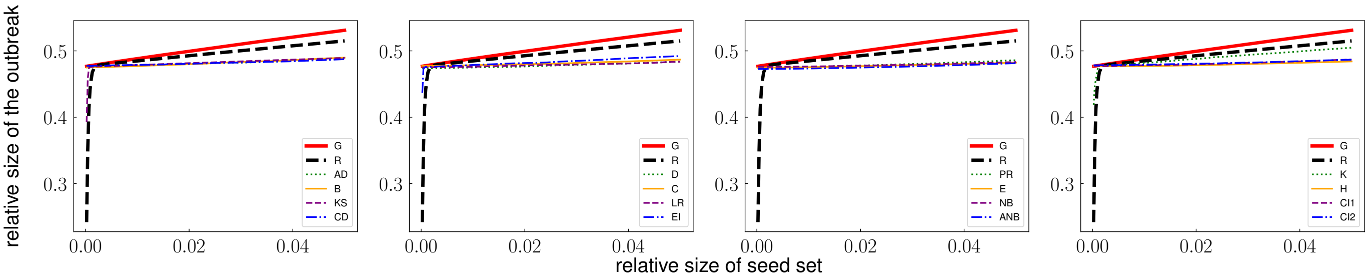

Figure 256: Rochester - p=2.0p_c

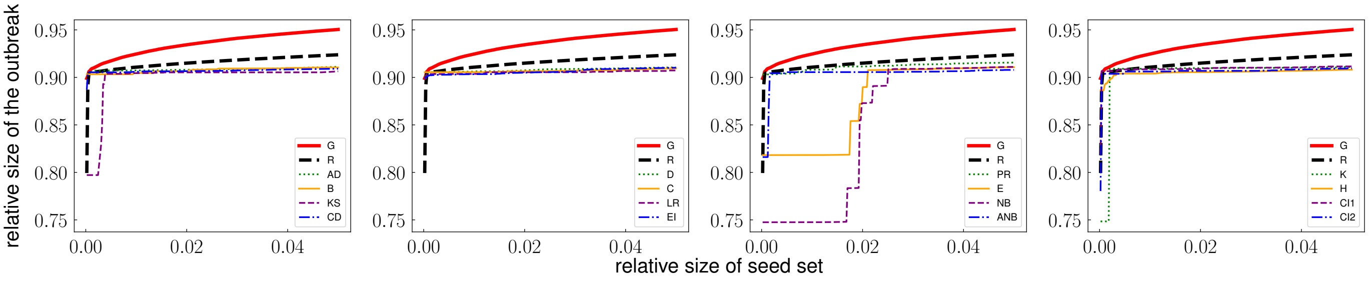

Figure 257: US Power grid - p=2.0p_c

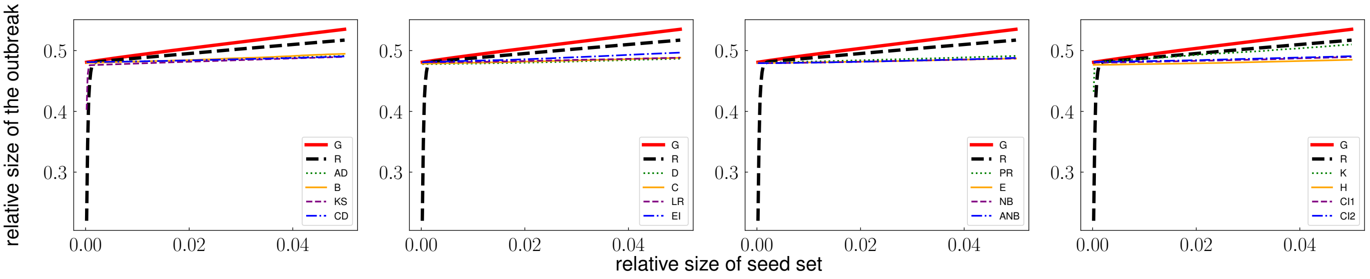

Figure 258: Lehigh - p=2.0p_c

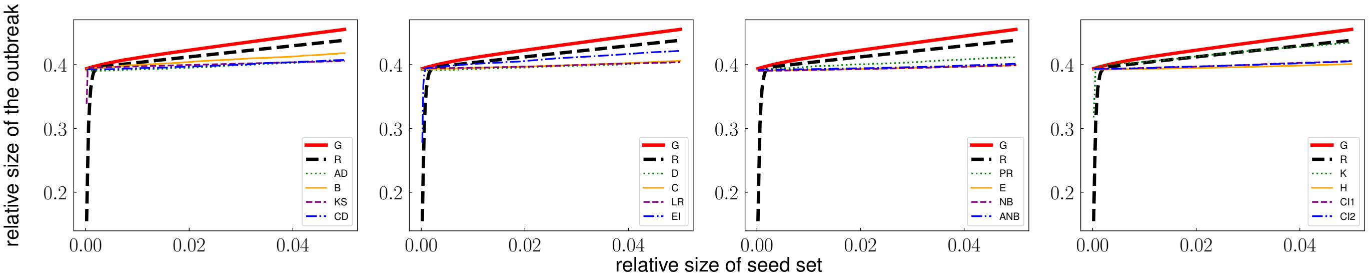

Figure 259: Johns Hopkins - p=2.0p_c

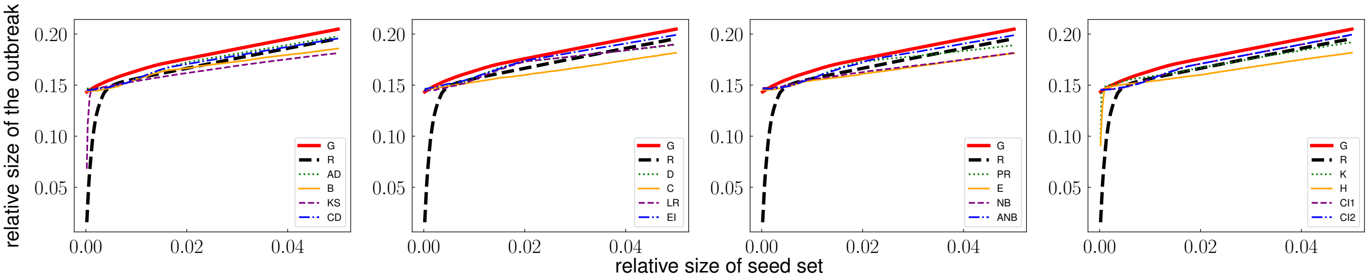

Figure 260: HT09 - p=2.0p_c

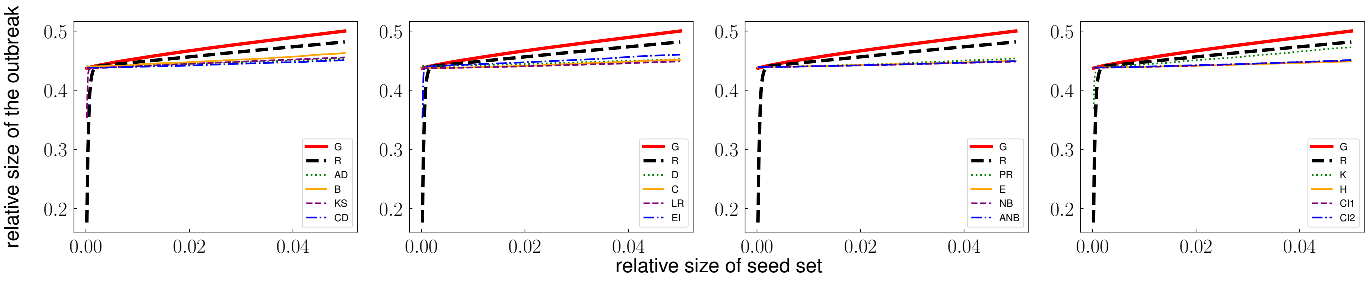

Figure 261: Wake - p=2.0p_c

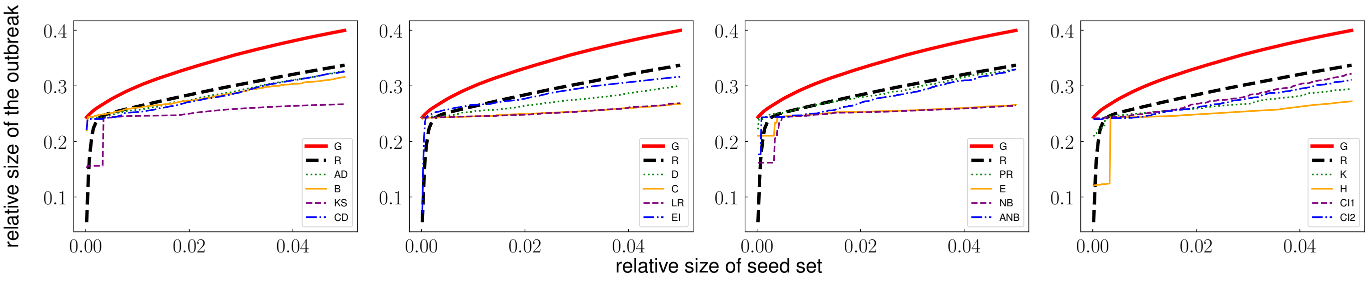

Figure 262: Hep-Th, 1995-1999 - p=2.0p_c

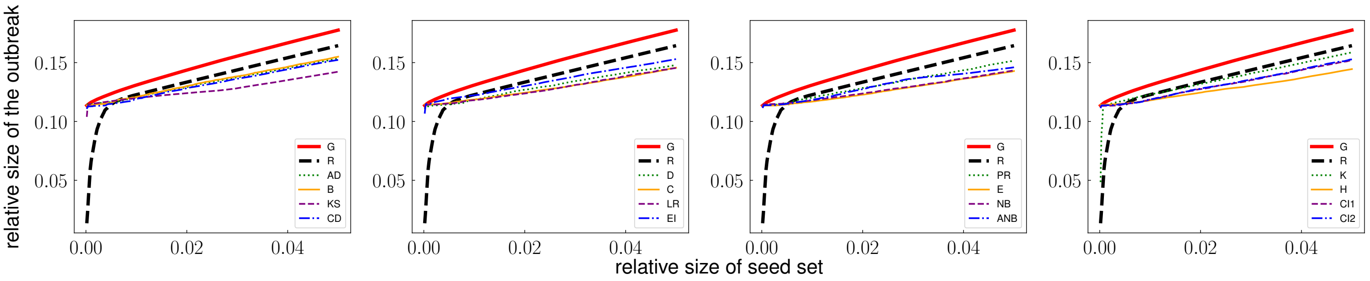

Figure 263: Bitcoin OTC - p=2.0p_c

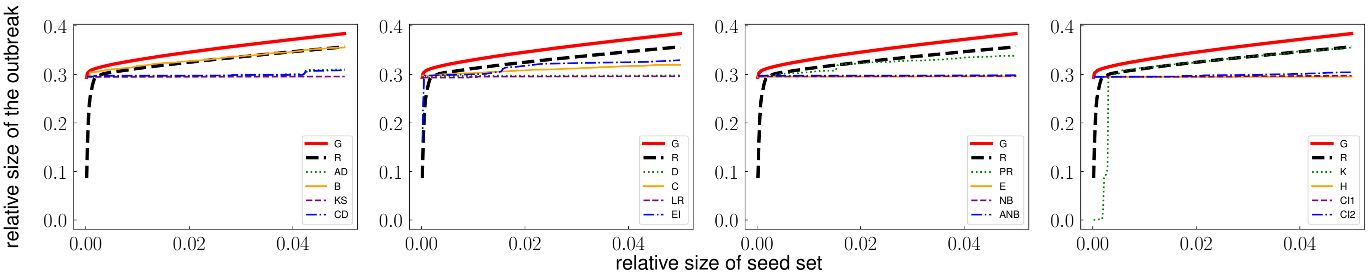

Figure 264: Reactome - p=2.0p_c

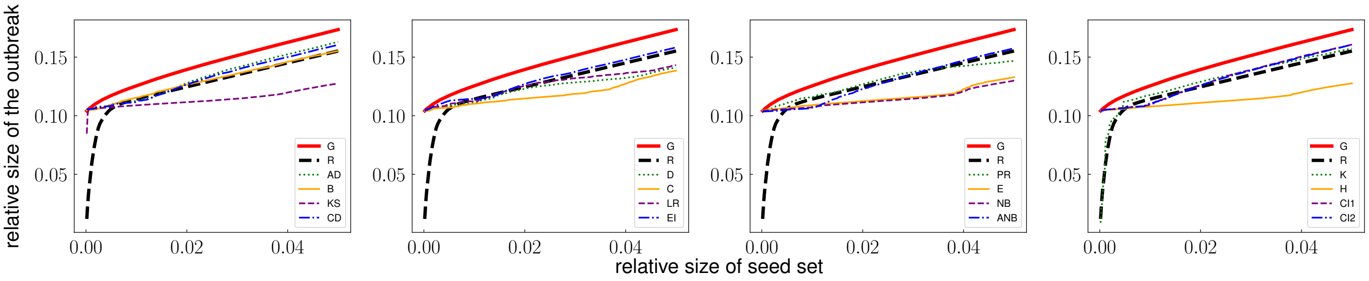

Figure 265: Jung - p=2.0p_c

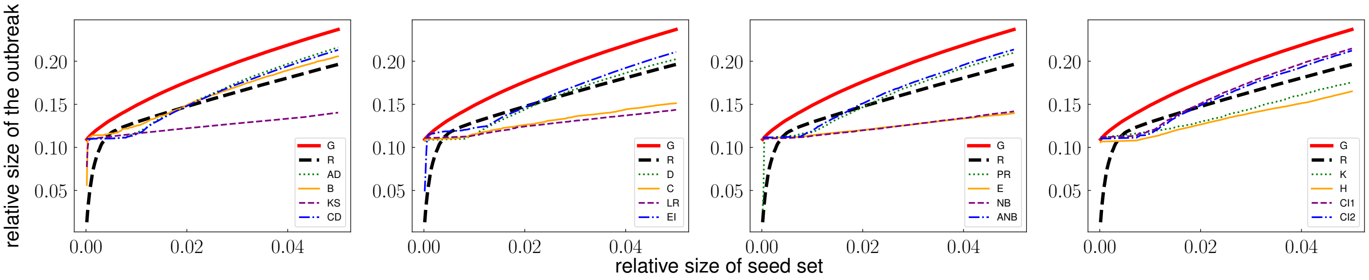

Figure 266: Gnutella, Aug. 8, 2002 - p=2.0p_c

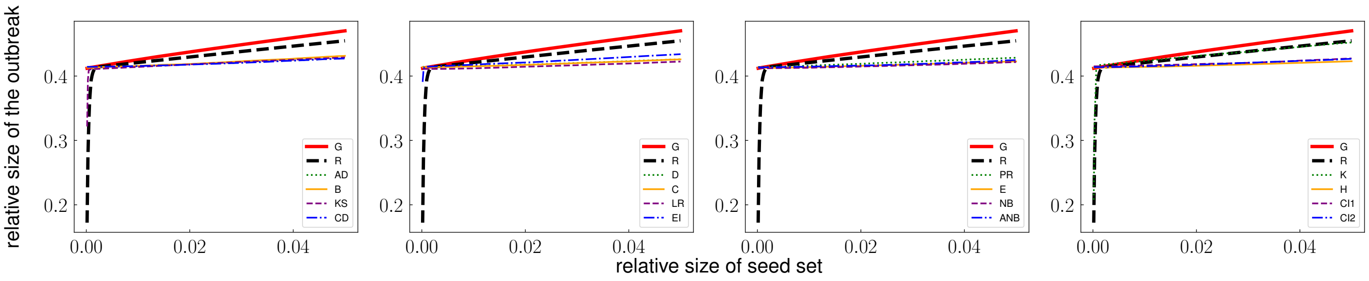

Figure 267: American - p=2.0p_c

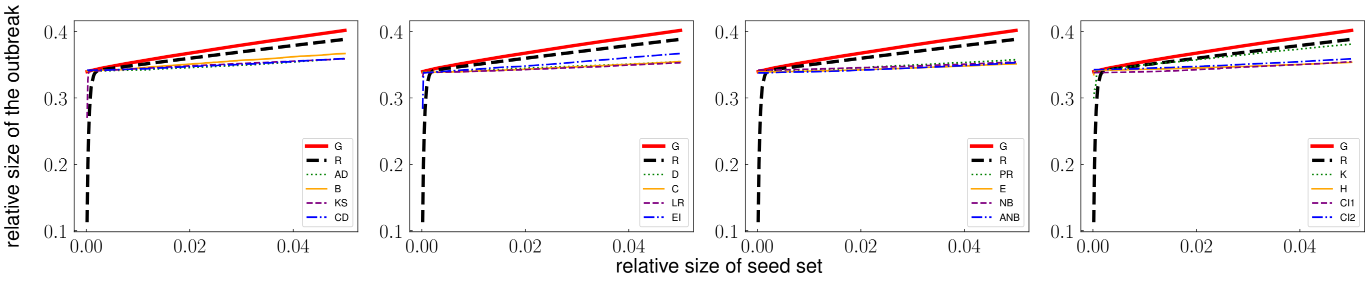

Figure 268: MIT - p=2.0p_c

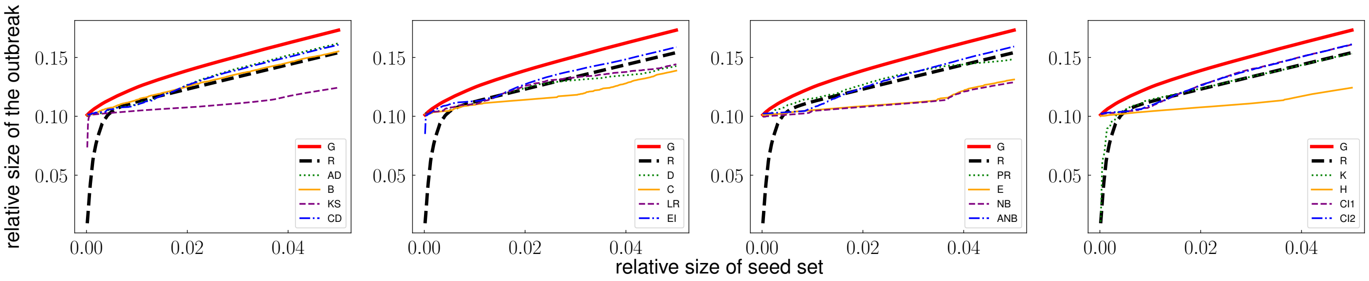

Figure 269: JDK - p=2.0p_c

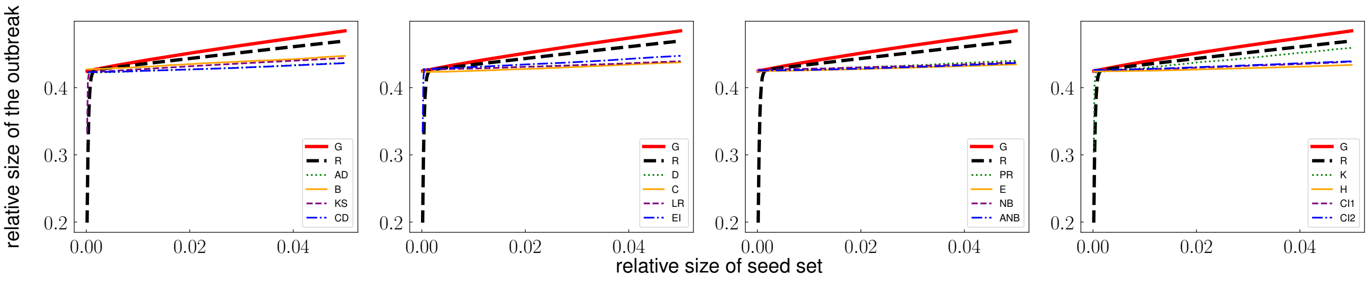

Figure 270: William - p=2.0p_c

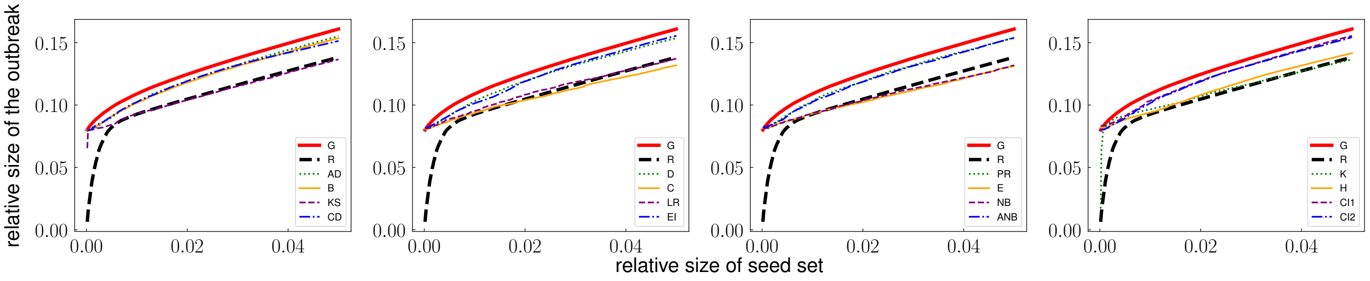

Figure 271: AS Oregon - p=2.0p_c

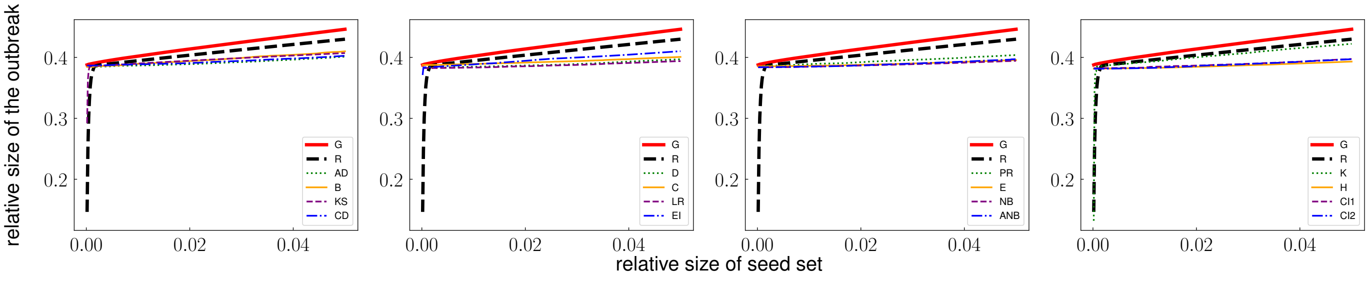

Figure 272: UChicago - p=2.0p_c

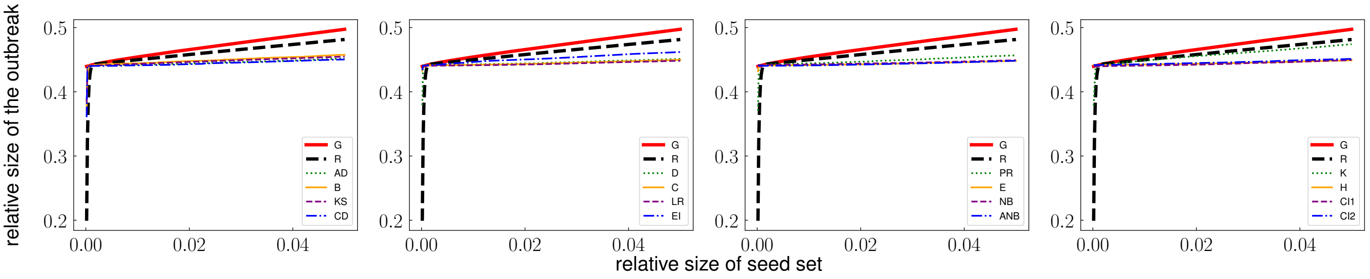

Figure 273: Princeton - p=2.0p_c

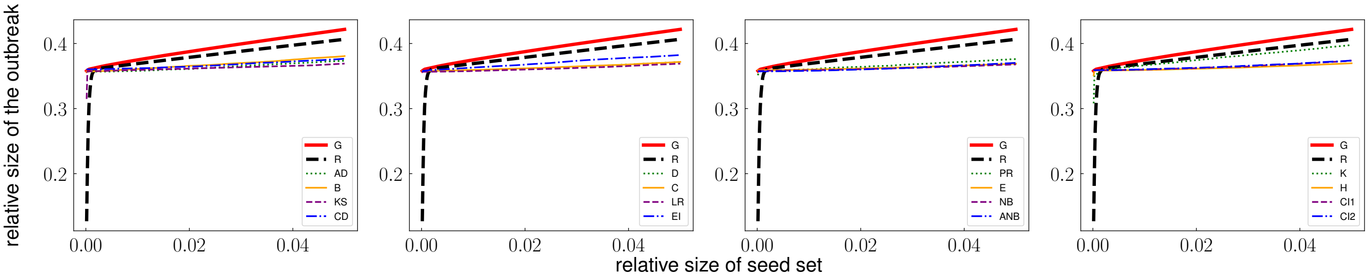

Figure 274: Carnegie - p=2.0p_c

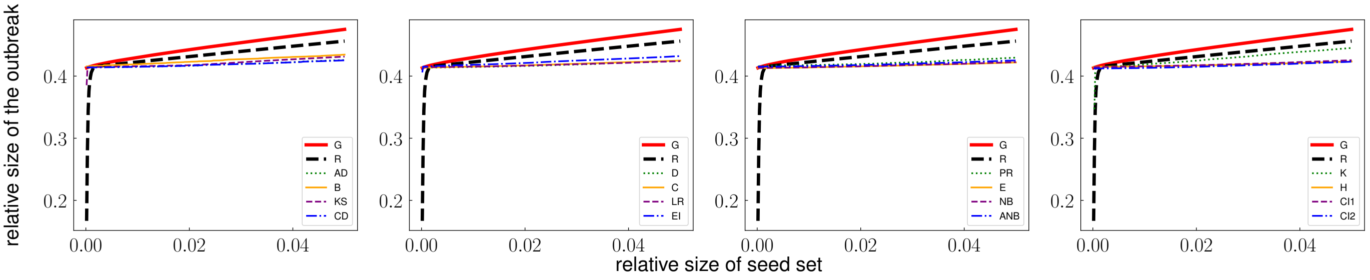

Figure 275: Tufts - p=2.0p_c

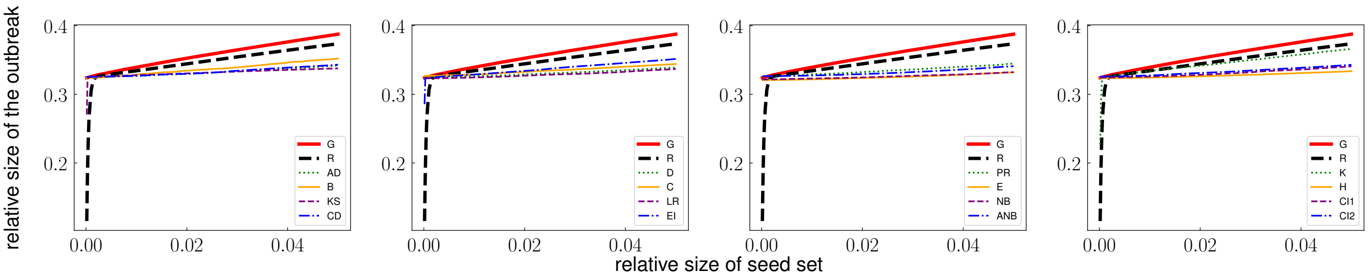

Figure 276: UC - p=2.0p_c

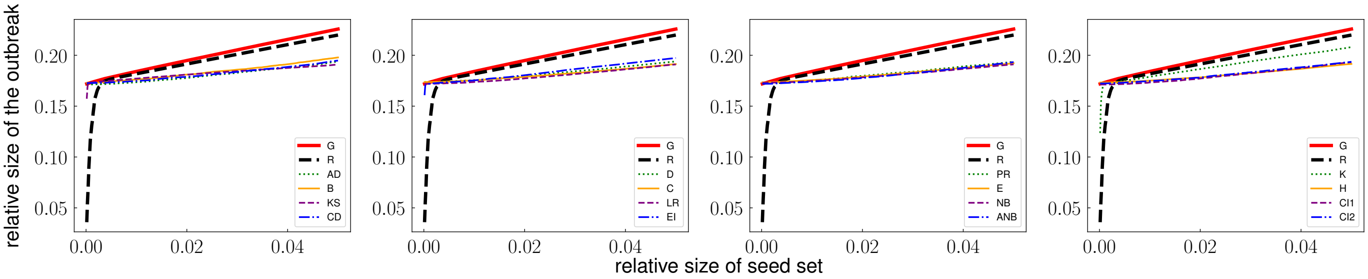

Figure 277: Wikipedia elections - p=2.0p_c

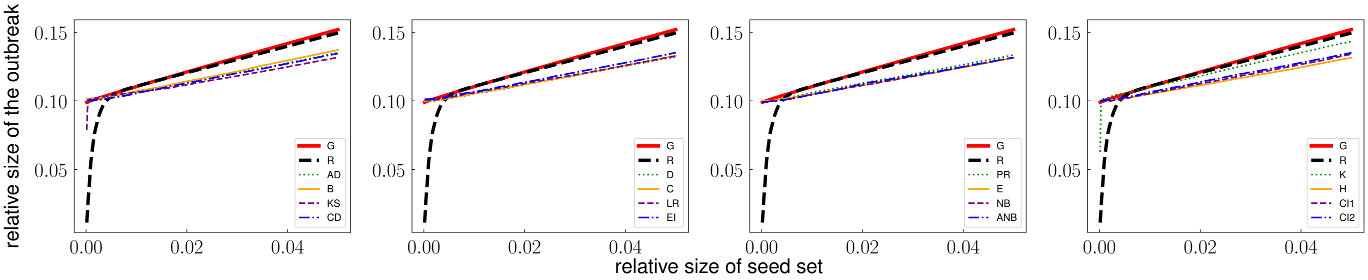

Figure 278: English - p=2.0p_c

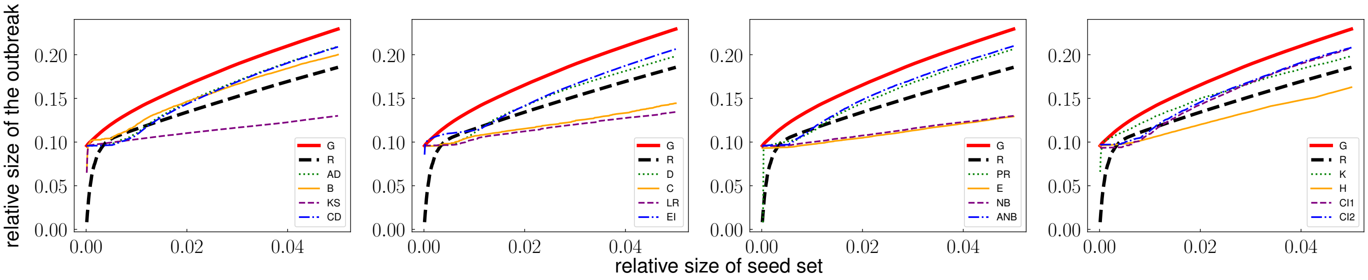

Figure 279: Gnutella, Aug. 9, 2002 - p=2.0p_c

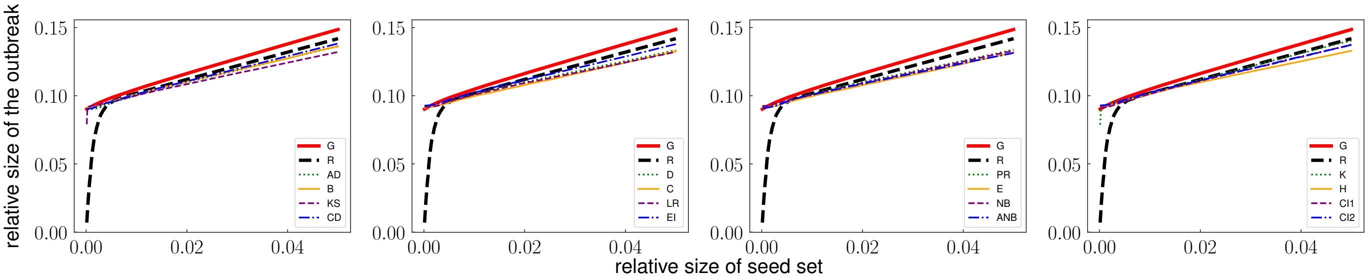

Figure 280: French - p=2.0p_c

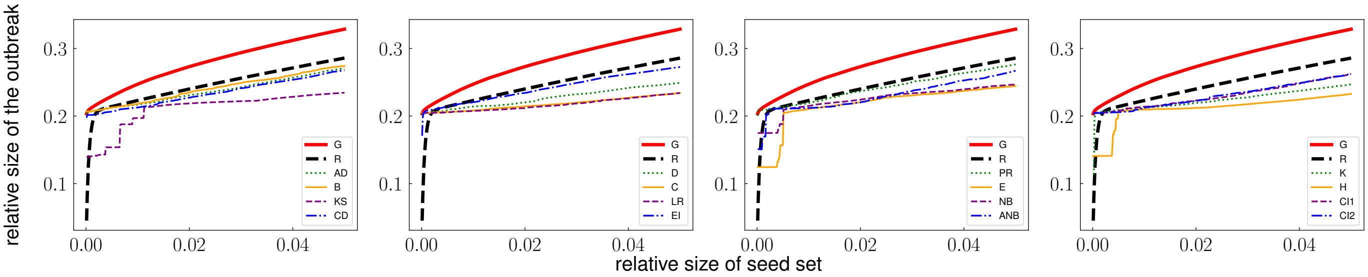

Figure 281: Hep-Th, 1993-2003 - p=2.0p_c

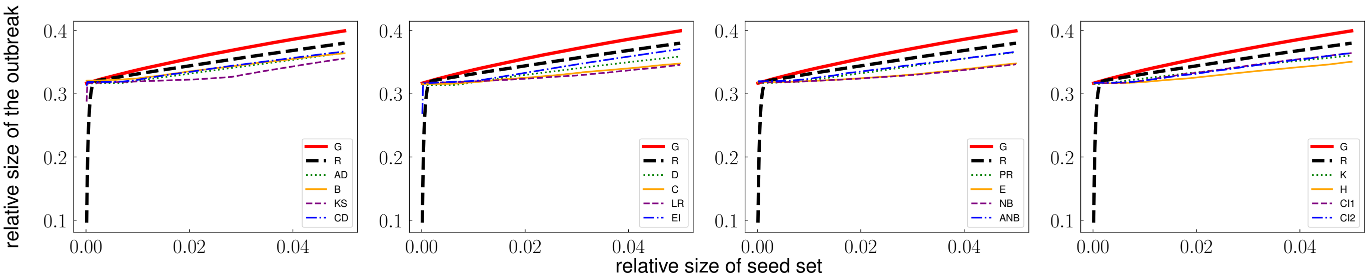

Figure 282: Gnutella, Aug. 6, 2002 - p=2.0p_c

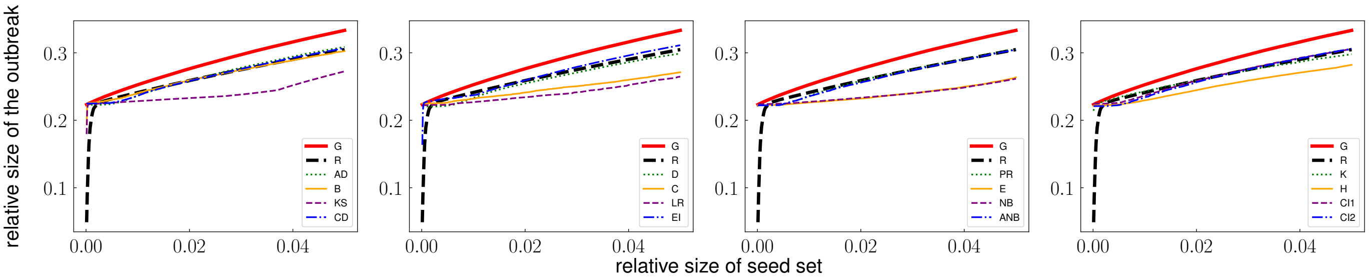

Figure 283: Gnutella, Aug. 5, 2002 - p=2.0p_c

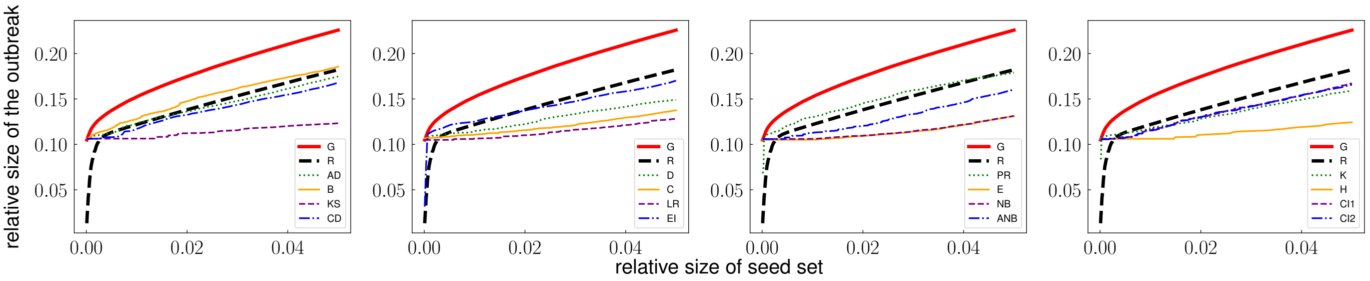

Figure 284: PGP - p=2.0p_c

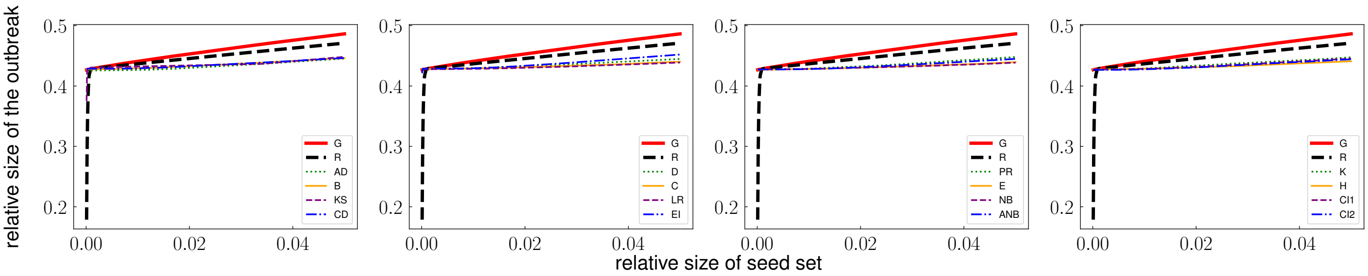

Figure 285: Gnutella, Aug. 4, 2002 - $p=2.0p_c$

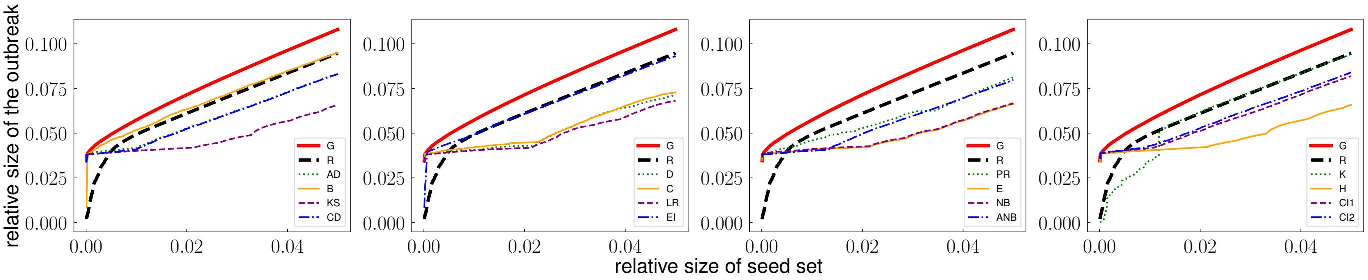

Figure 286: Hep-Ph, 1993-2003 - $p=2.0p_c$

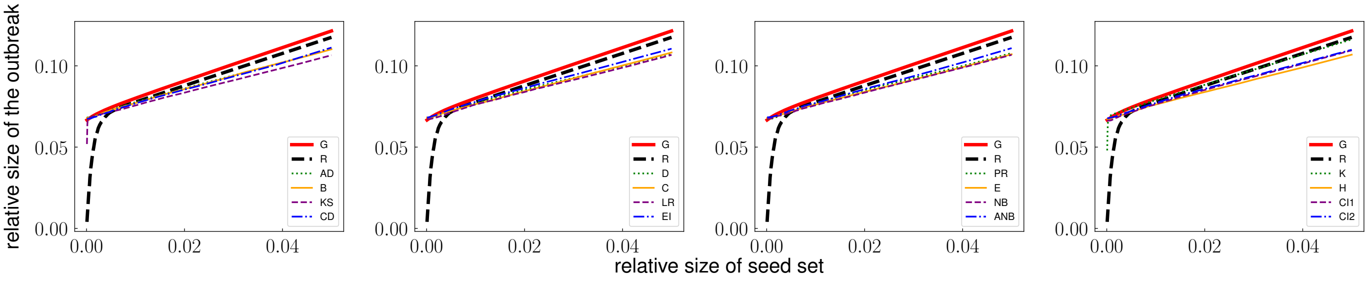

Figure 287: Spanish 1 - $p=2.0p_c$

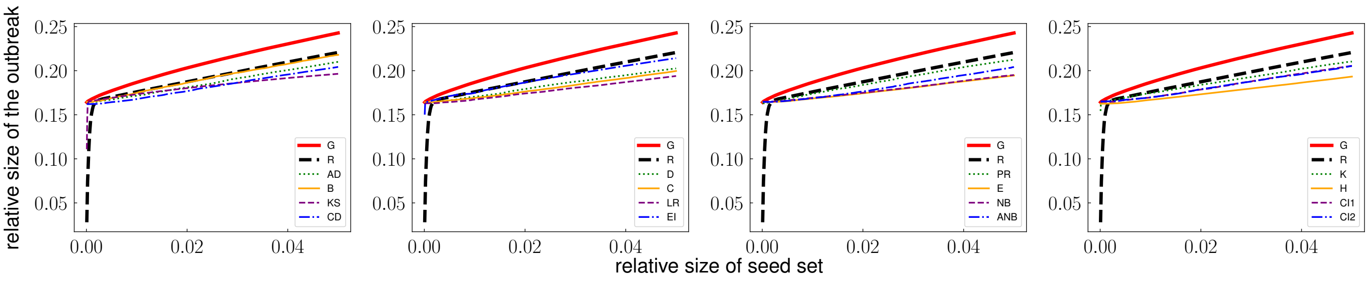

Figure 288: DBLP, citations - p=2.0p_c

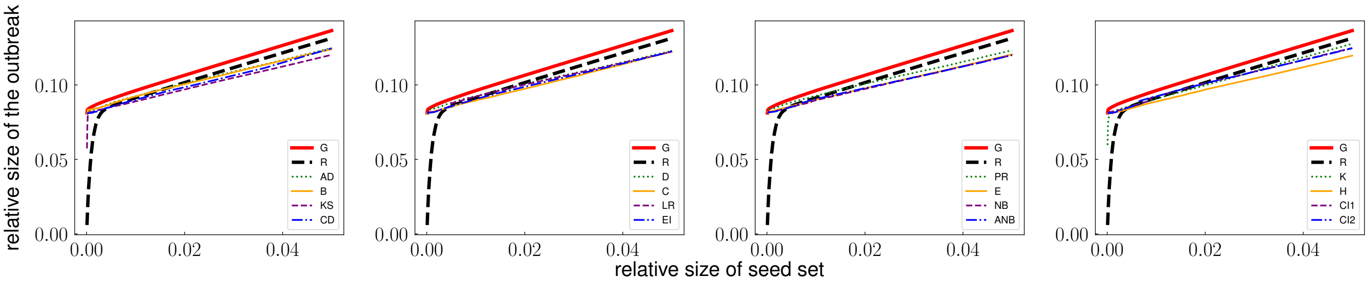

Figure 289: Spanish 2 - p=2.0p_c

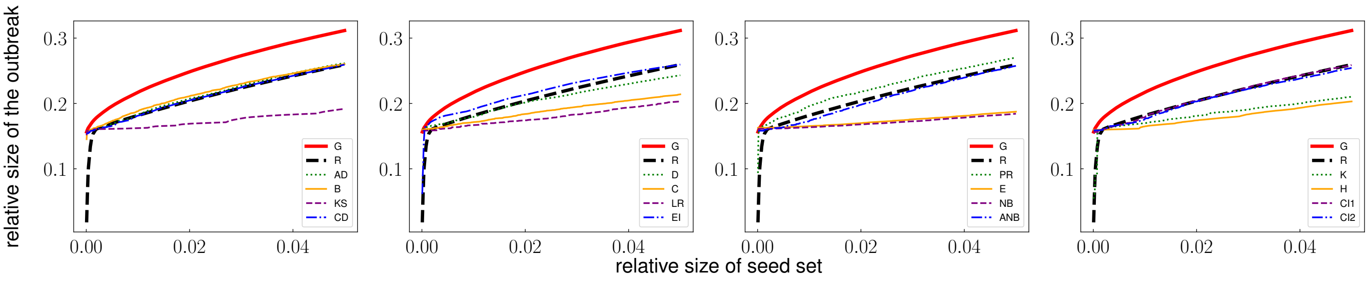

Figure 290: Cond-Mat, 1995-1999 - p=2.0p_c

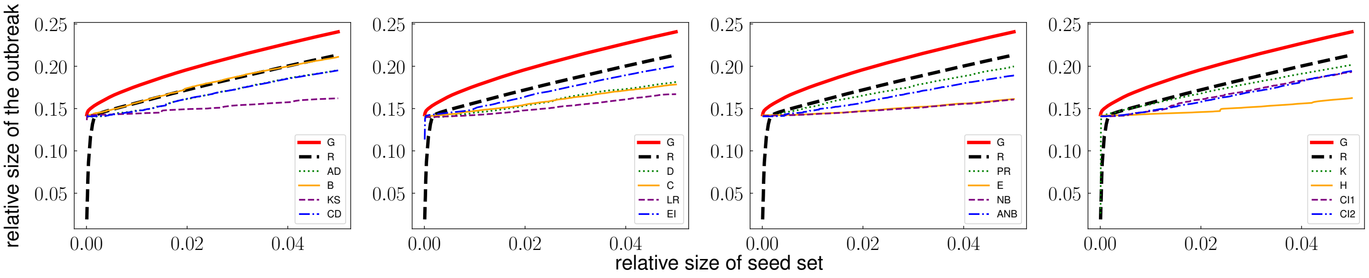

Figure 291: Astrophysics - $p=2.0p_c$

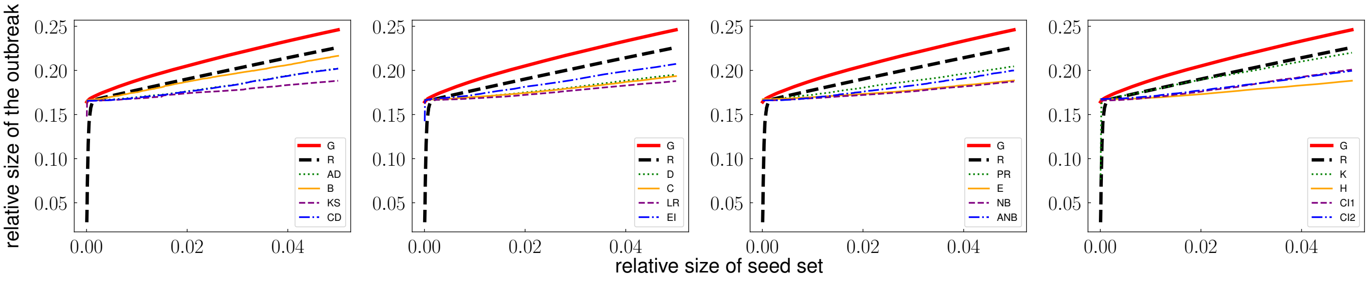

Figure 292: AstroPhys, 1993-2003 - $p=2.0p_c$

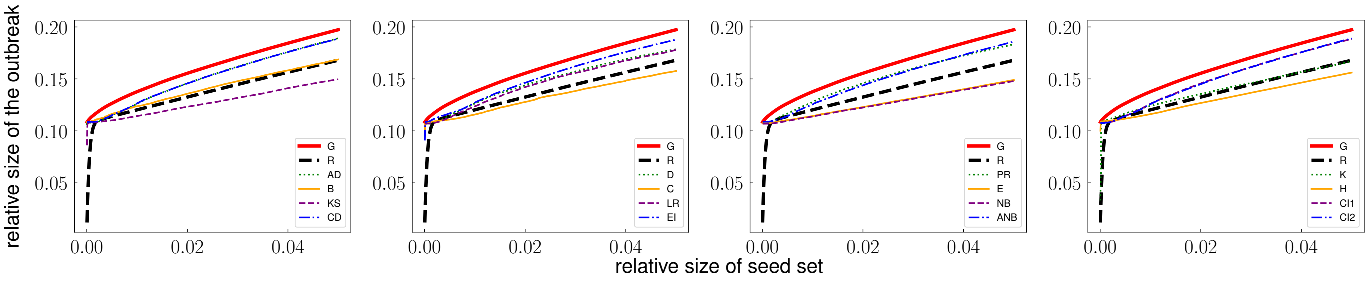

Figure 293: Marvel - $p=2.0p_c$

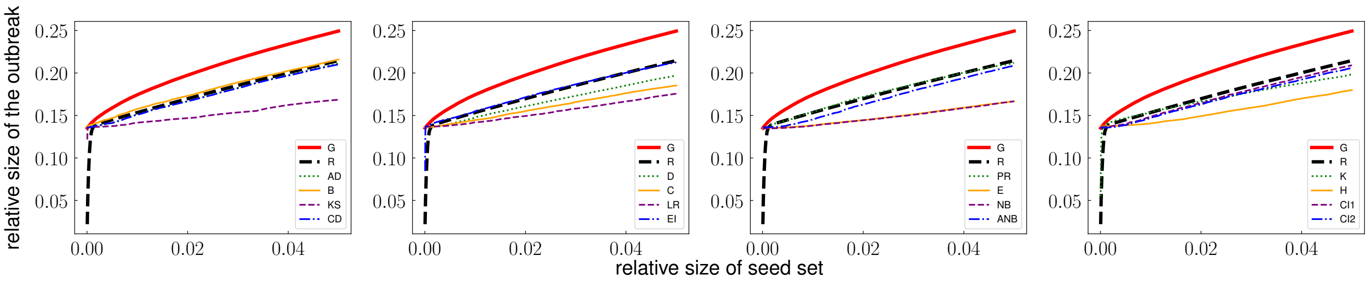

Figure 294: Cond-Mat, 1993-2003 - p=2.0p_c

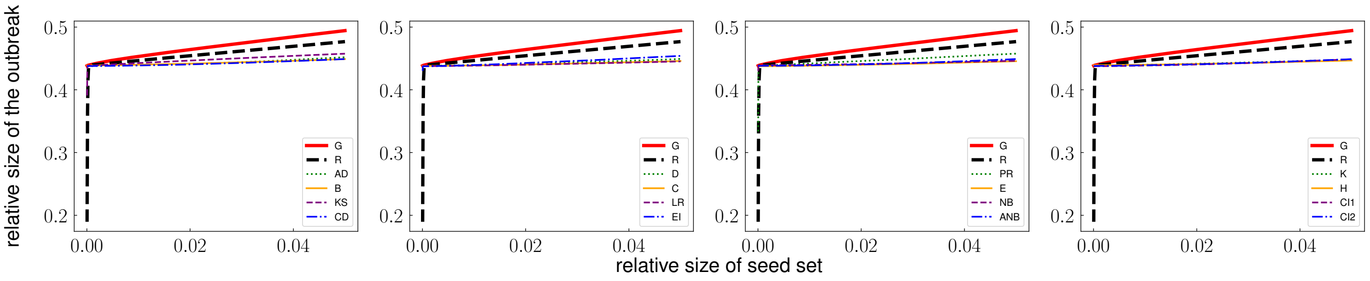

Figure 295: Gnutella, Aug. 25, 2002 - p=2.0p_c

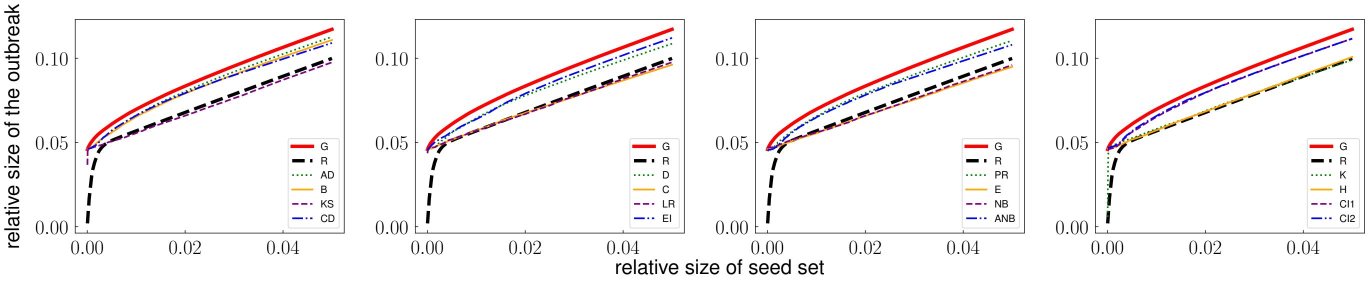

Figure 296: Internet - p=2.0p_c

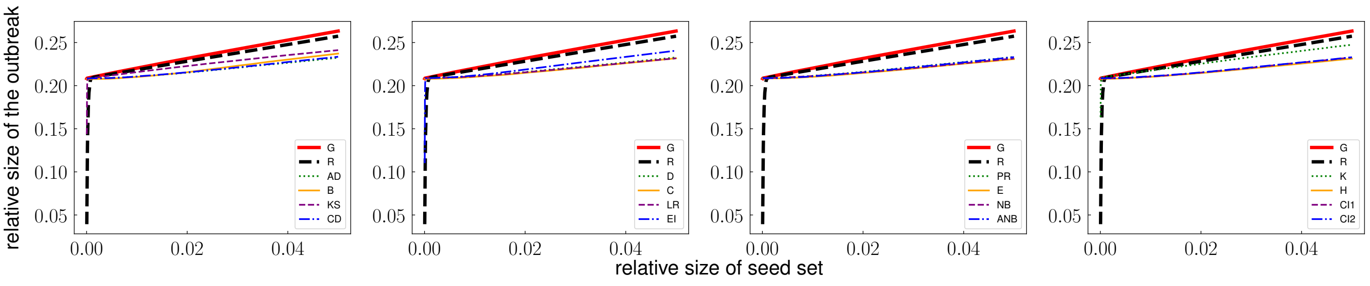

Figure 297: Thesaurus - p=2.0p_c

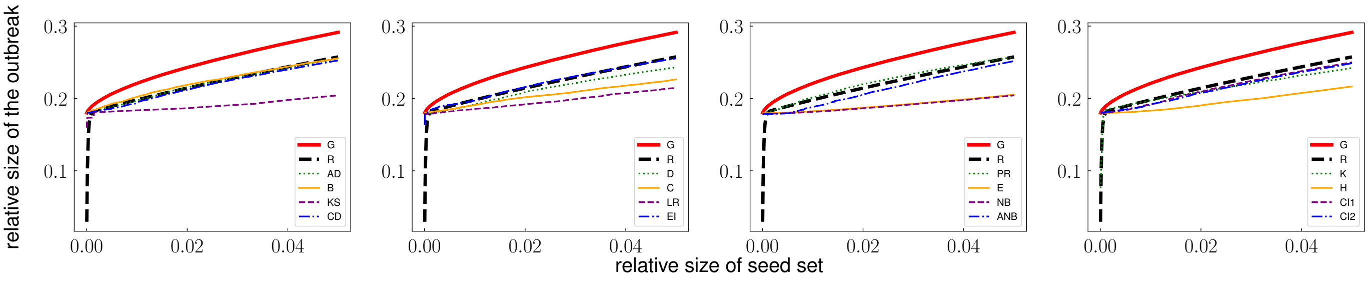

Figure 298: Cora - p=2.0p_c

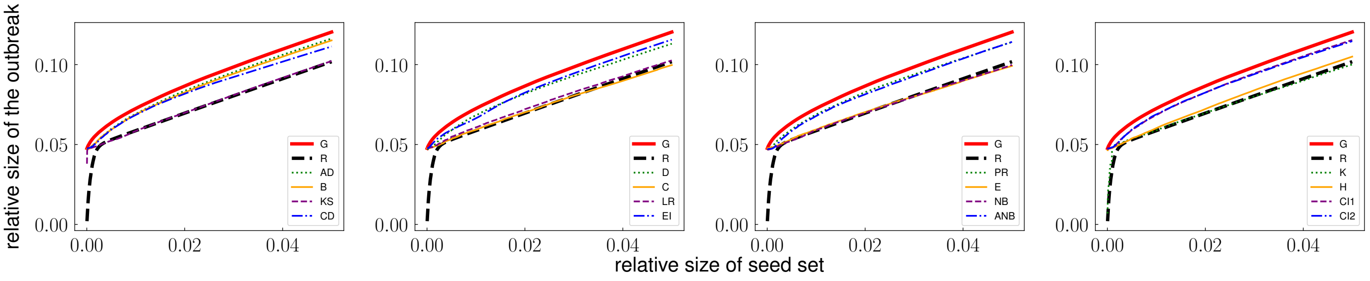

Figure 299: AS Caida - p=2.0p_c

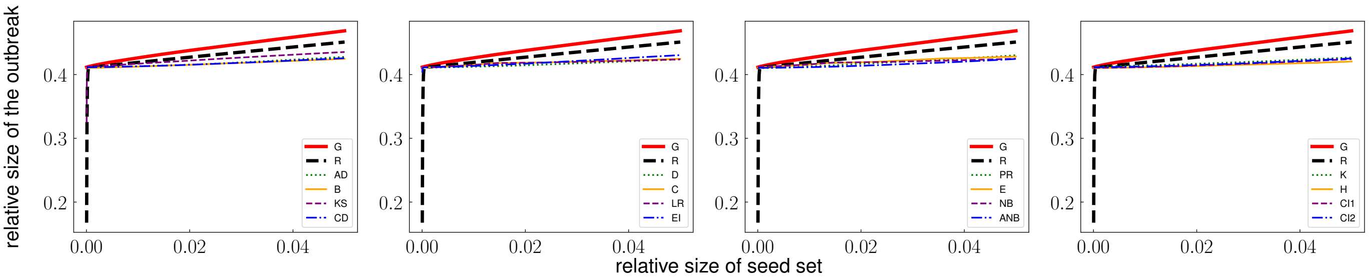

Figure 300: Gnutella, Aug. 24, 2002 - p=2.0p_c

3 T=0.10

3.1 Relative size of seed set vs relative size of the outbreak

3.1.1 Subcritical Regime

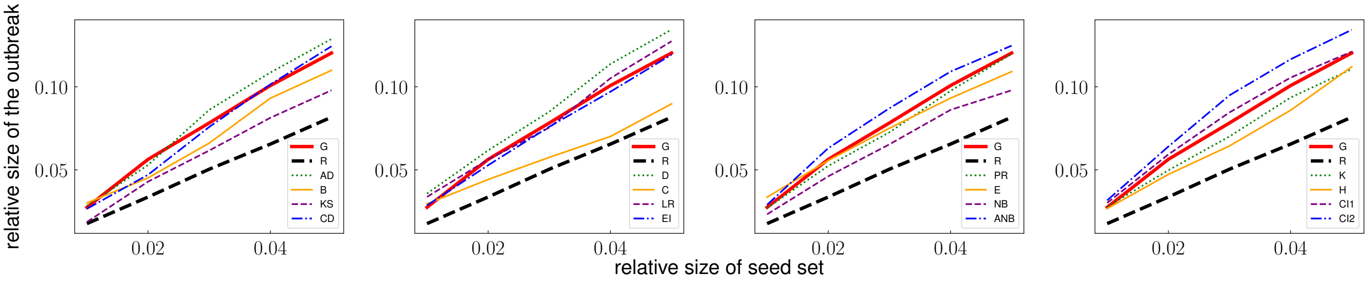

Figure 301: Political books - p=0.5p_c

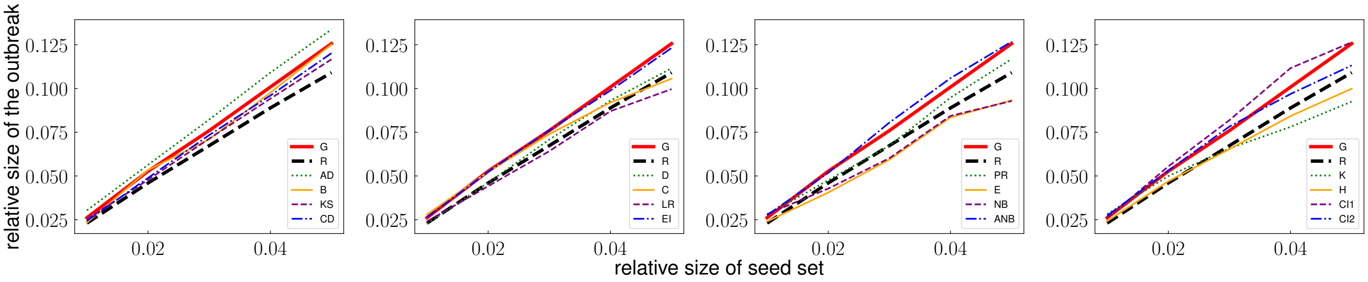

Figure 302: College football - p=0.5p_c

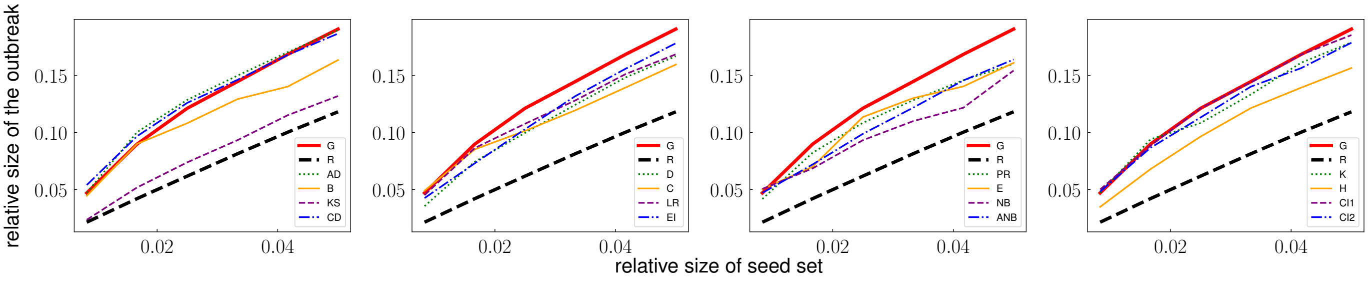

Figure 303: S208 - p=0.5p_c

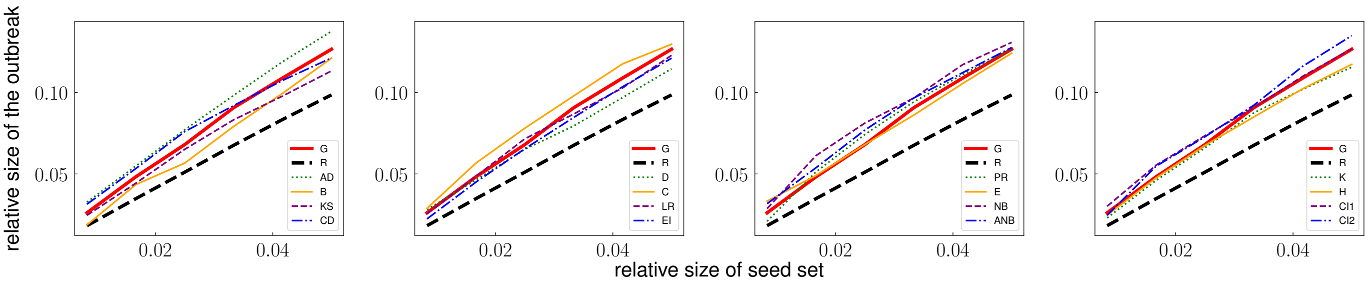

Figure 304: High school, 2011 - $p=0.5p_c$

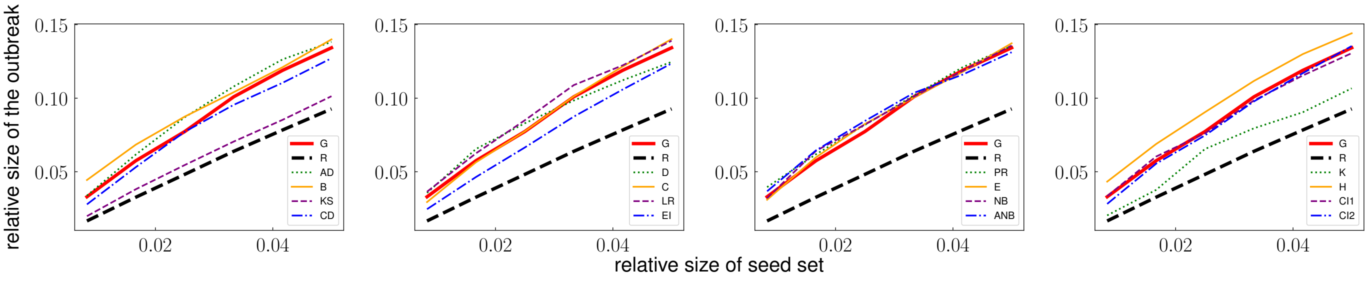

Figure 305: Bay Dry - $p=0.5p_c$

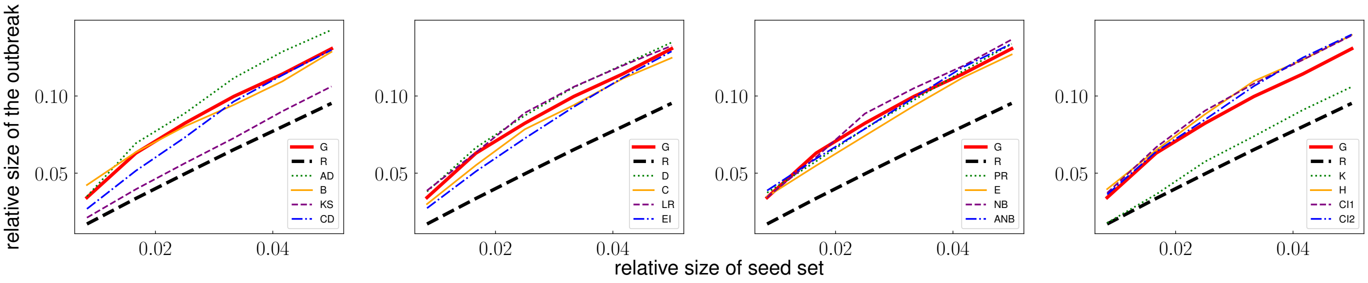

Figure 306: Bay Wet - $p=0.5p_c$

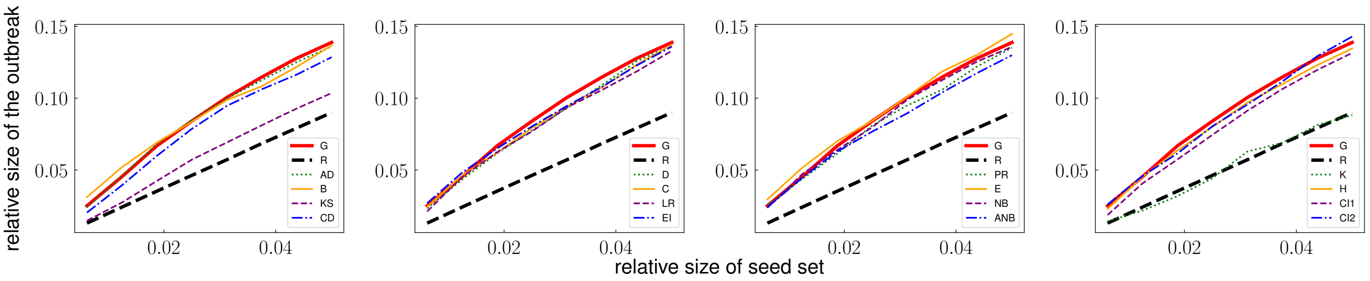

Figure 307: Radoslaw Email - p=0.5p_c

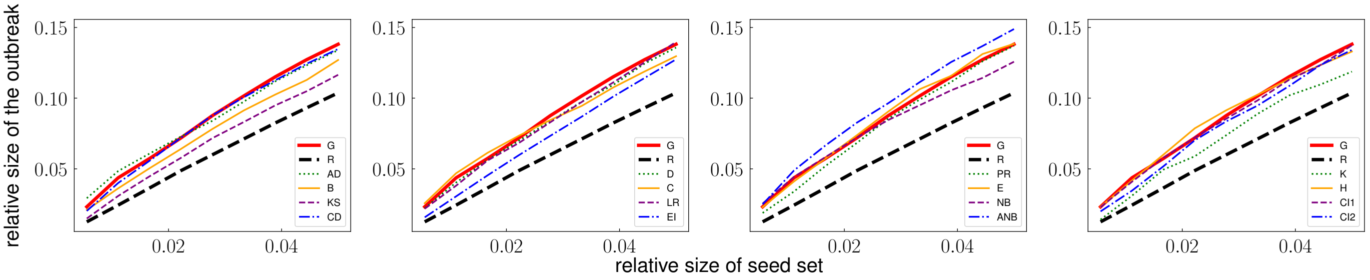

Figure 308: High school, 2012 - p=0.5p_c

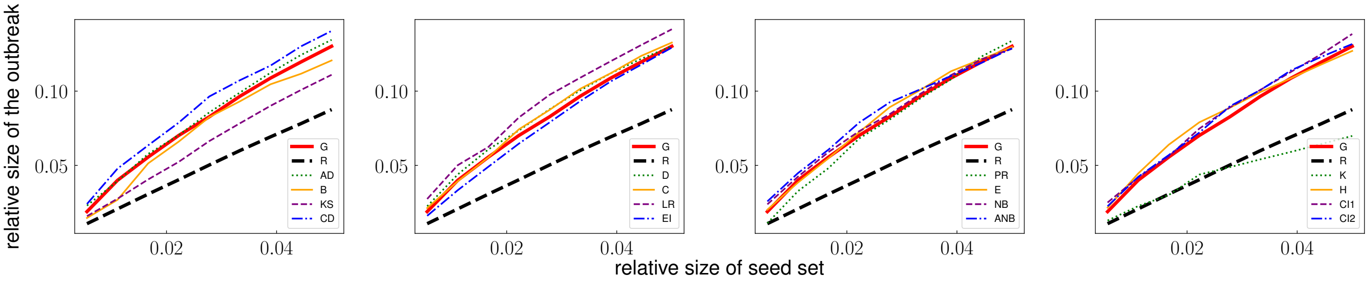

Figure 309: Little Rock Lake - p=0.5p_c

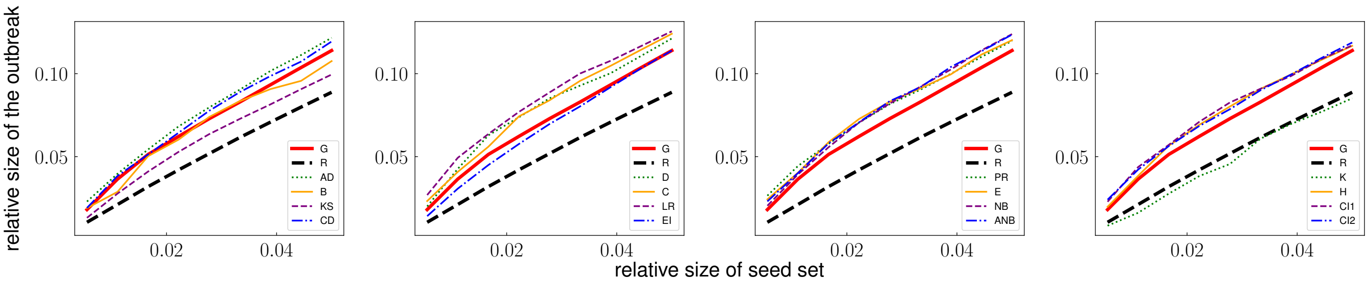

Figure 310: Jazz - p=0.5p_c

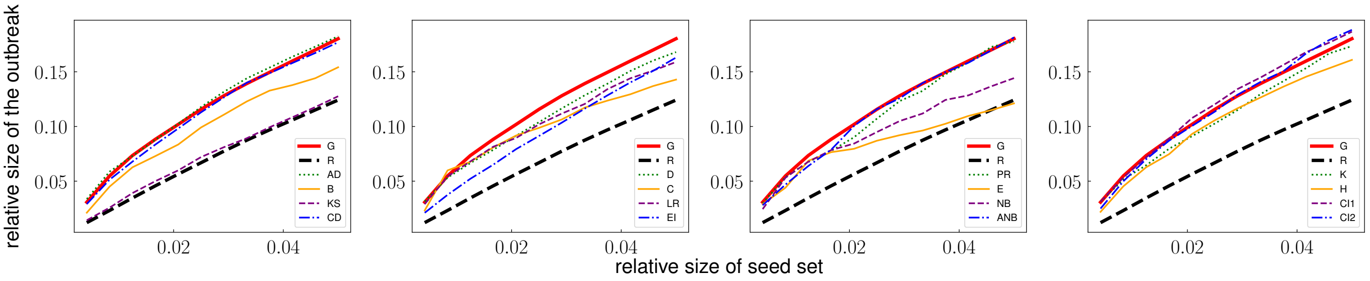

Figure 311: S420 - p=0.5p_c

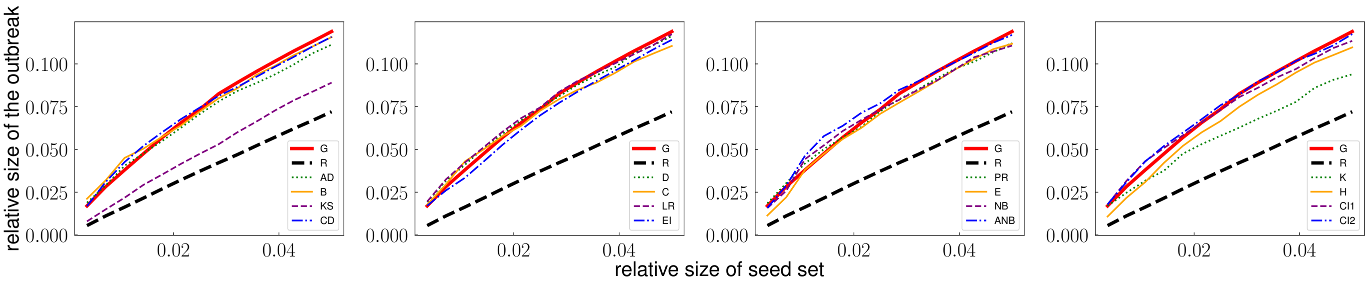

Figure 312: C. Elegans, neural - p=0.5p_c

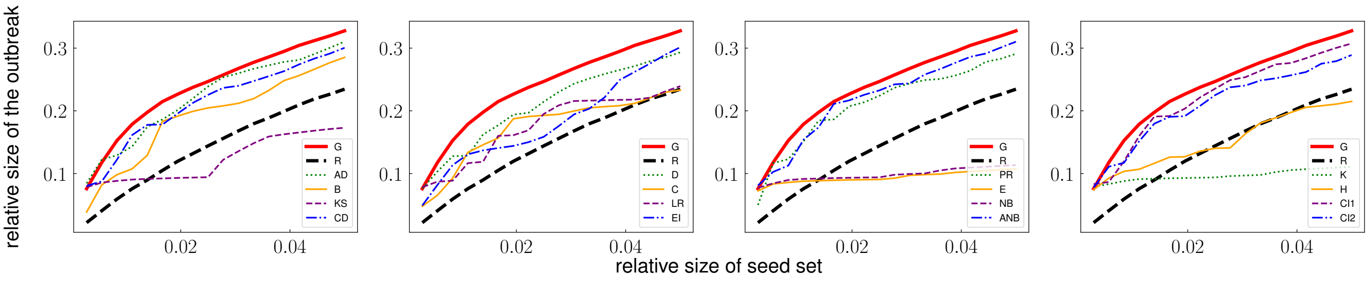

Figure 313: Network Science - $p{=}0.5p_c$

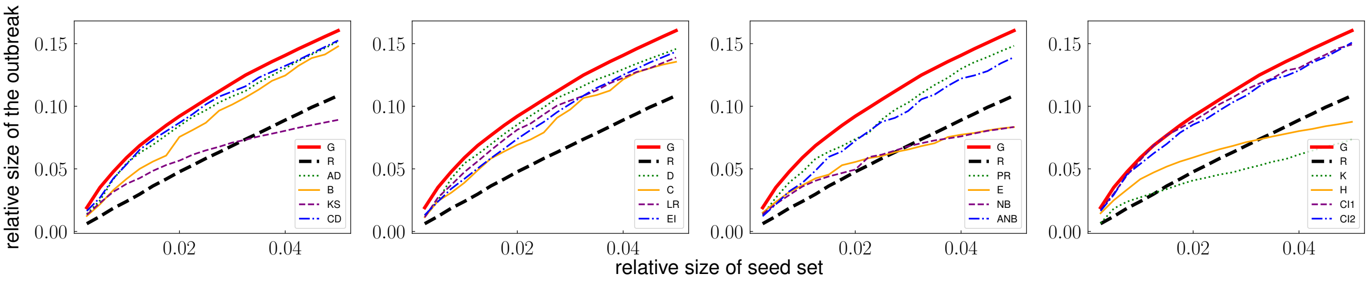

Figure 314: Dublin - $p{=}0.5p_c$

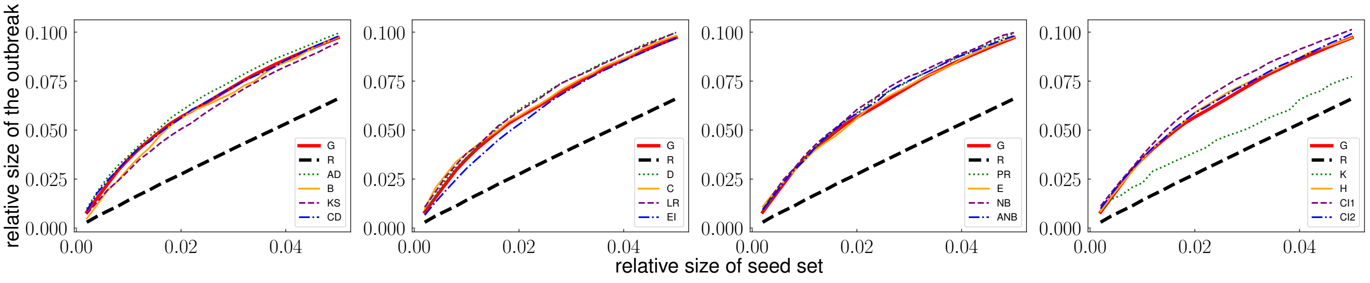

Figure 315: US Air Trasportation - $p{=}0.5p_c$

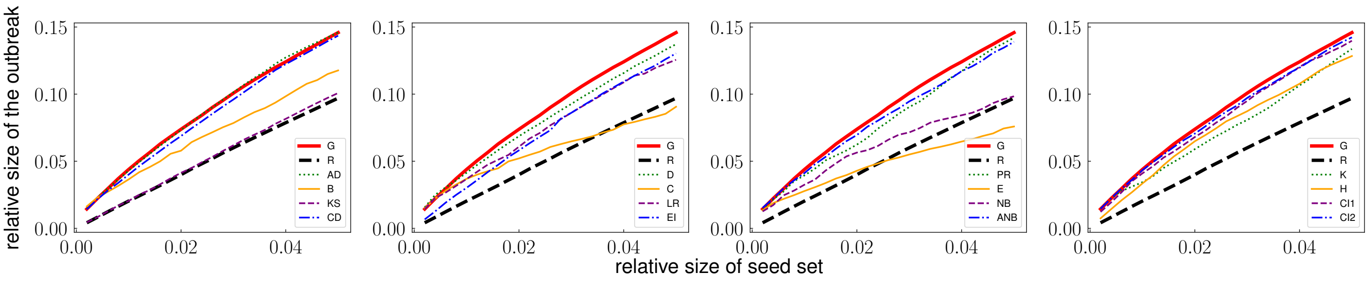

Figure 316: S838 - p=0.5p_c

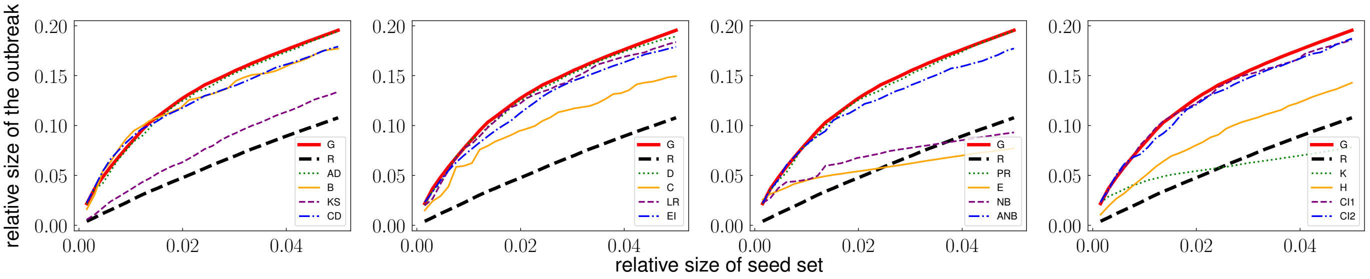

Figure 317: Yeast, transcription - p=0.5p_c

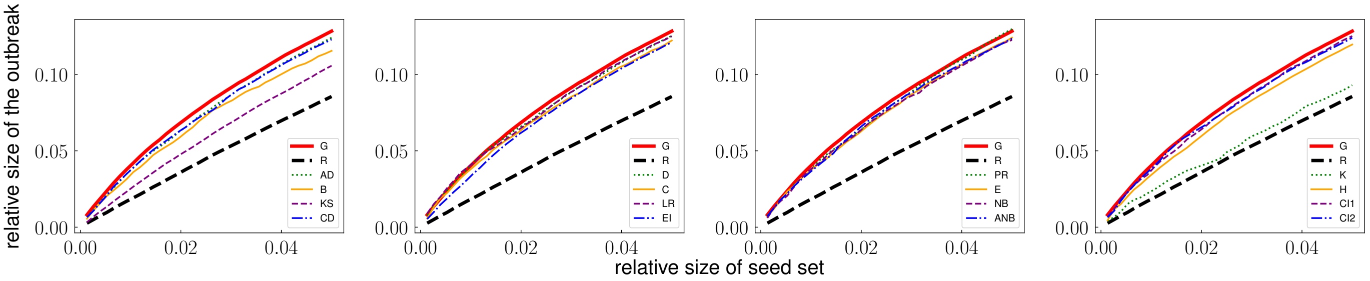

Figure 318: Caltech - p=0.5p_c

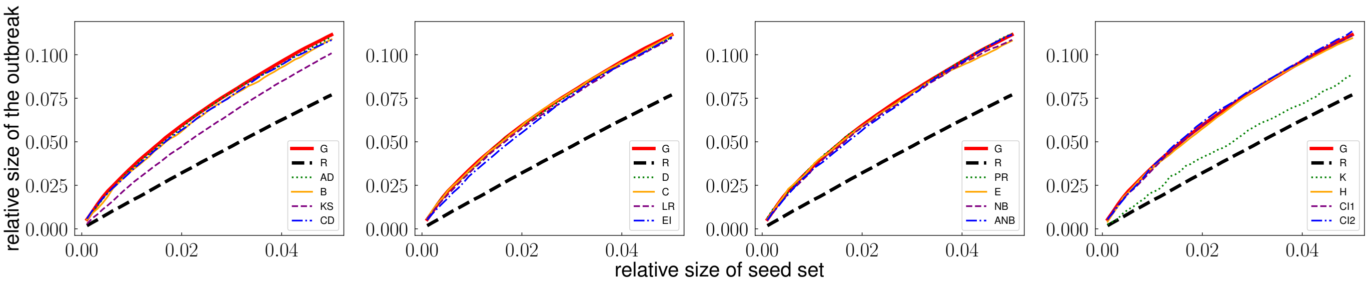

Figure 319: Reed - p=0.5p_c

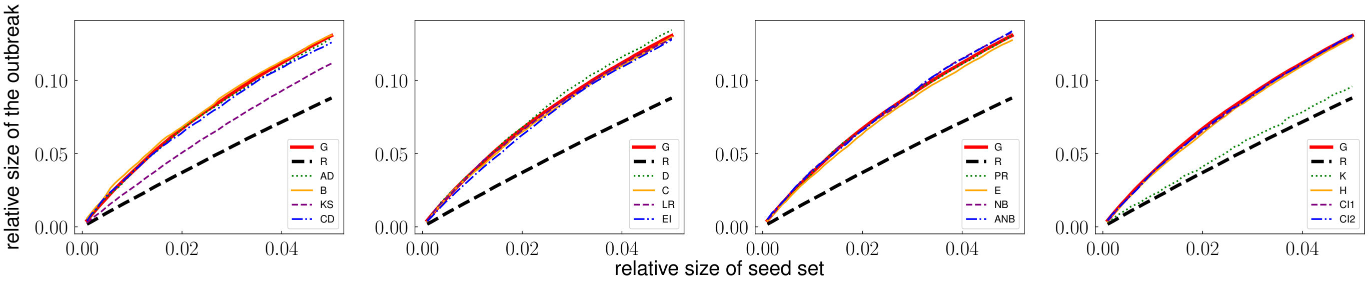

Figure 320: Mouse retina - p=0.5p_c

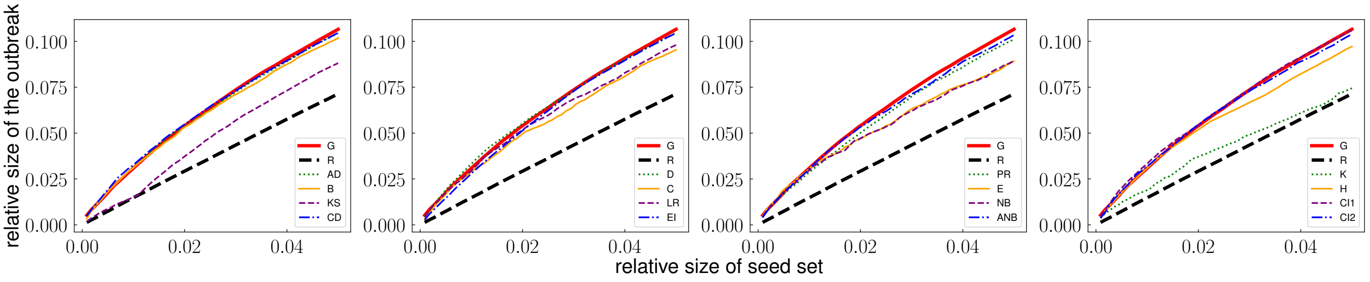

Figure 321: URV email - p=0.5p_c

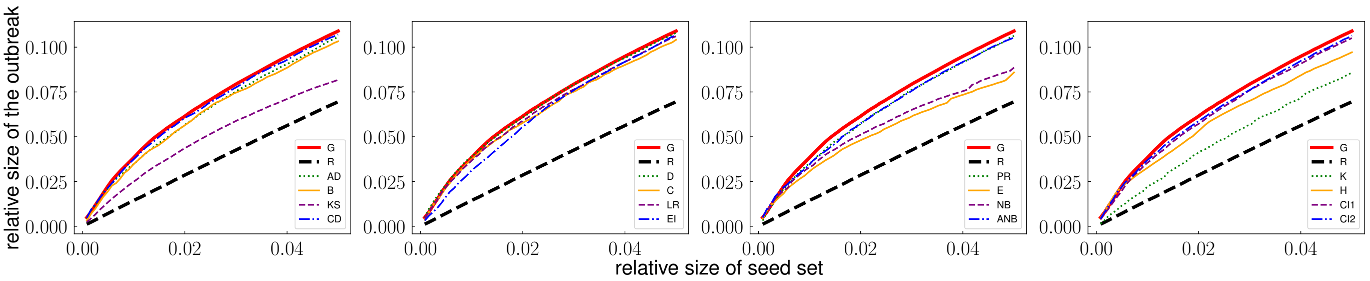

Figure 322: Political blogs - p=0.5p_c

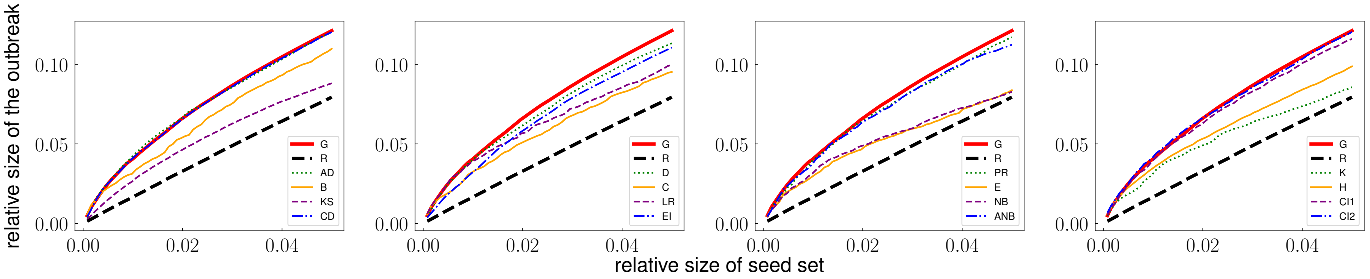

Figure 323: Air traffic - p=0.5p_c

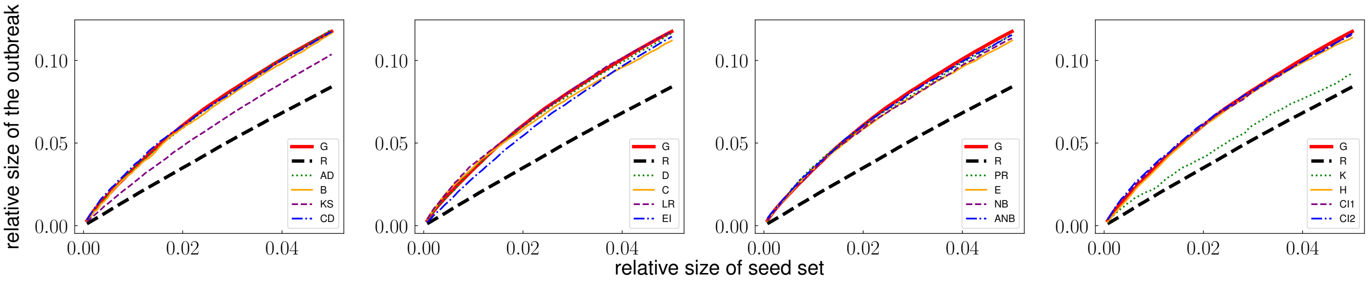

Figure 324: Haverford - p=0.5p_c

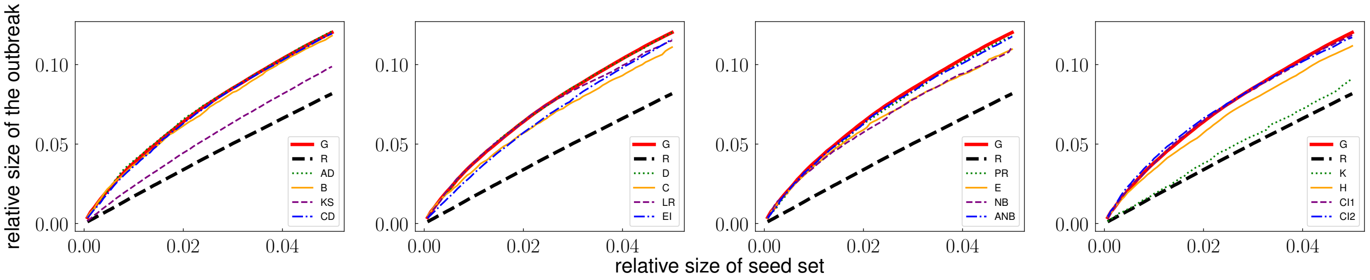

Figure 325: Simmons - p=0.5p_c

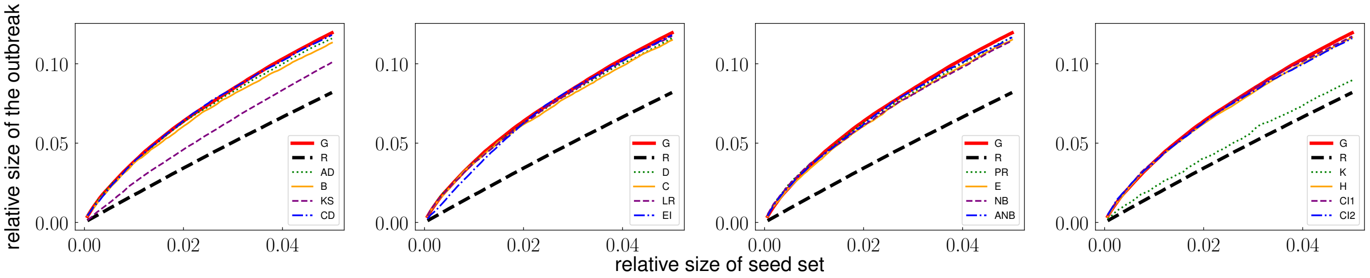

Figure 326: Swarthmore - p=0.5p_c

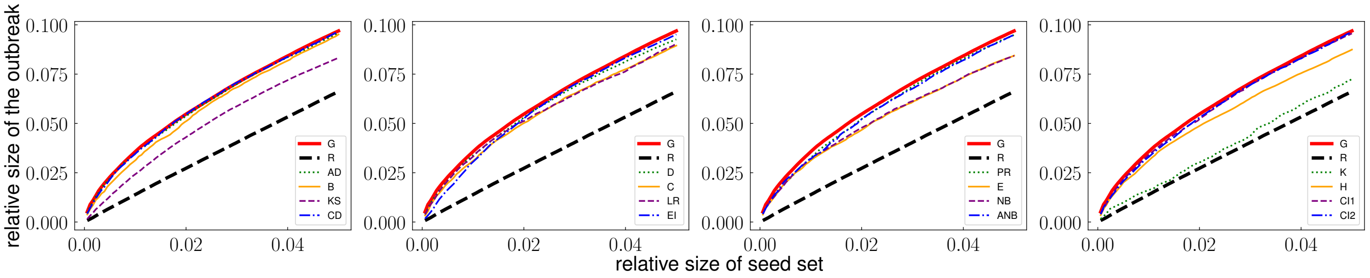

Figure 327: Petster, hamster - p=0.5p_c

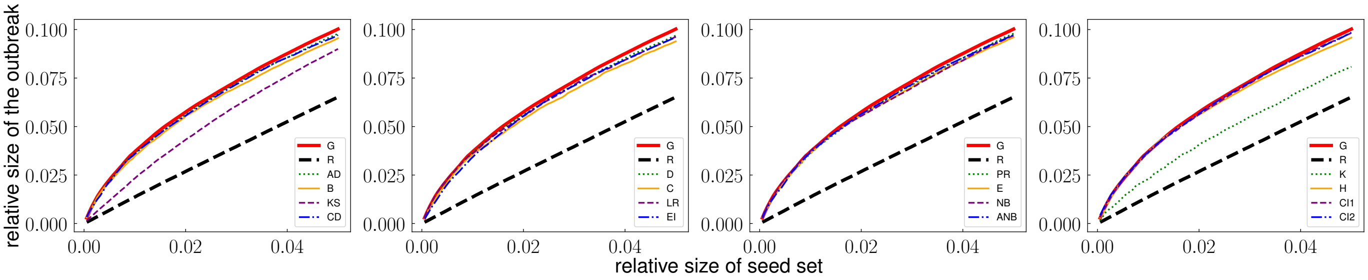

Figure 328: UC Irvine - p=0.5p_c

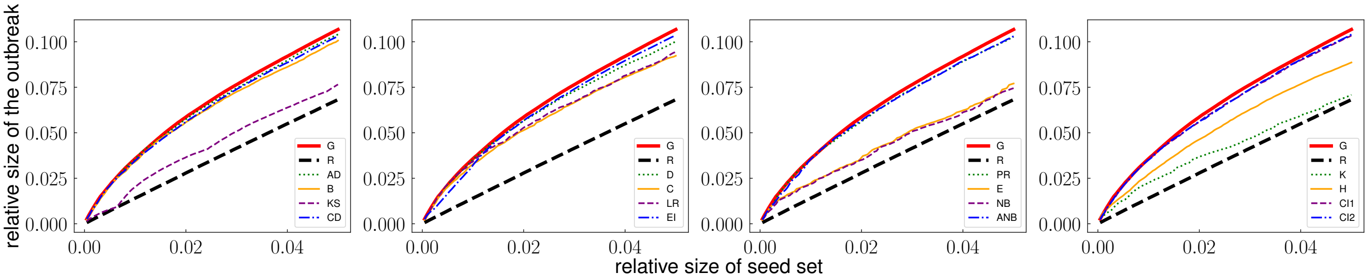

Figure 329: Yeast, protein - p=0.5p_c

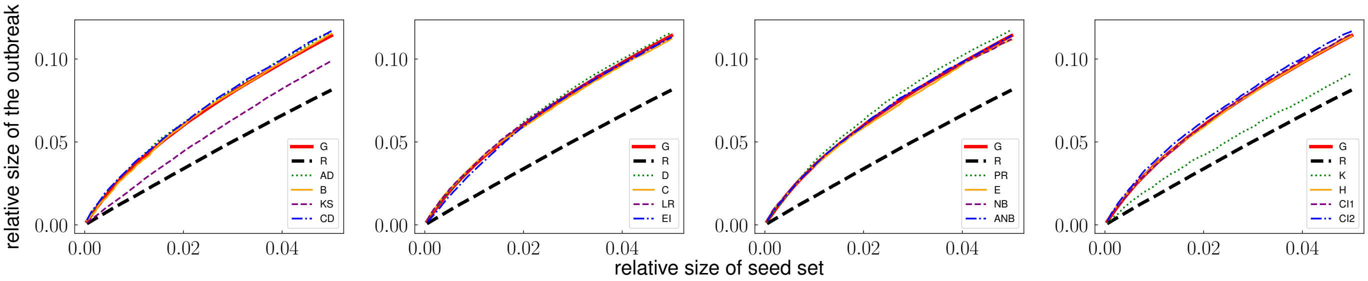

Figure 330: Amherst - p=0.5p_c

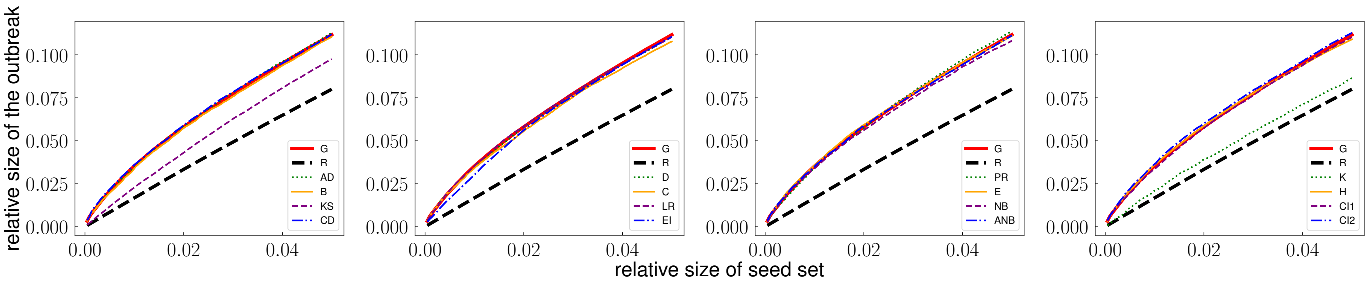

Figure 331: Bowdoin - p=0.5p_c

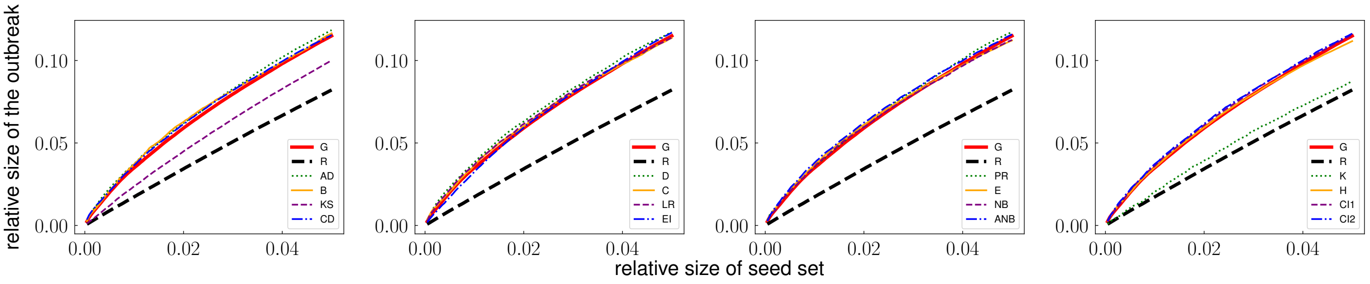

Figure 332: Hamilton - p=0.5p_c

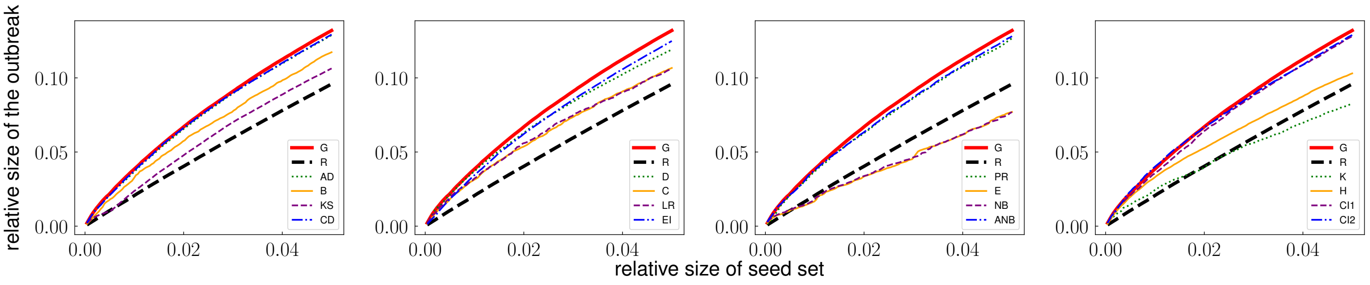

Figure 333: Adolescent health - p=0.5p_c

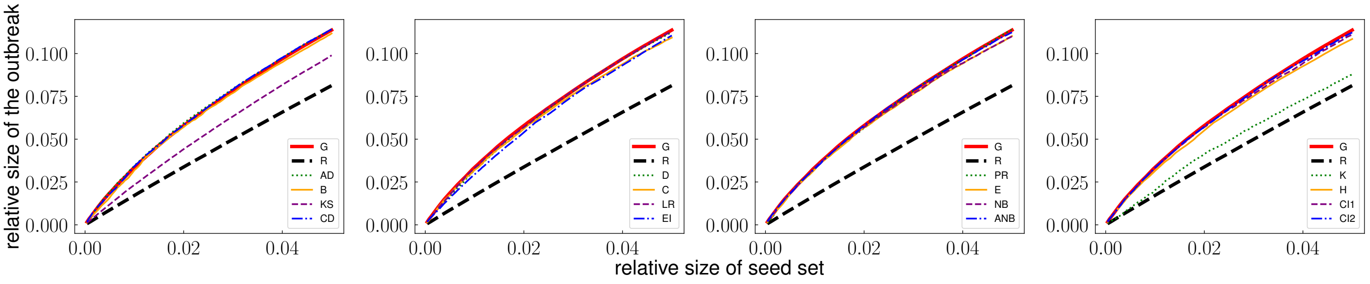

Figure 334: Trinity - p=0.5p_c

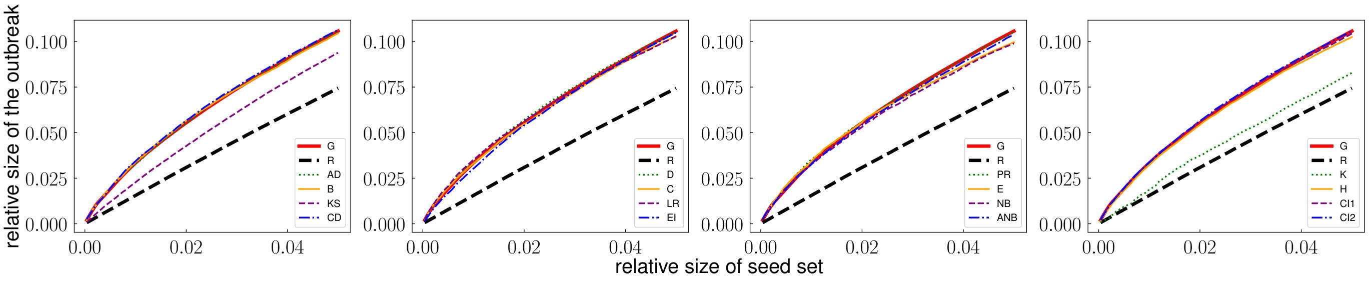

Figure 335: USFCA - p=0.5p_c

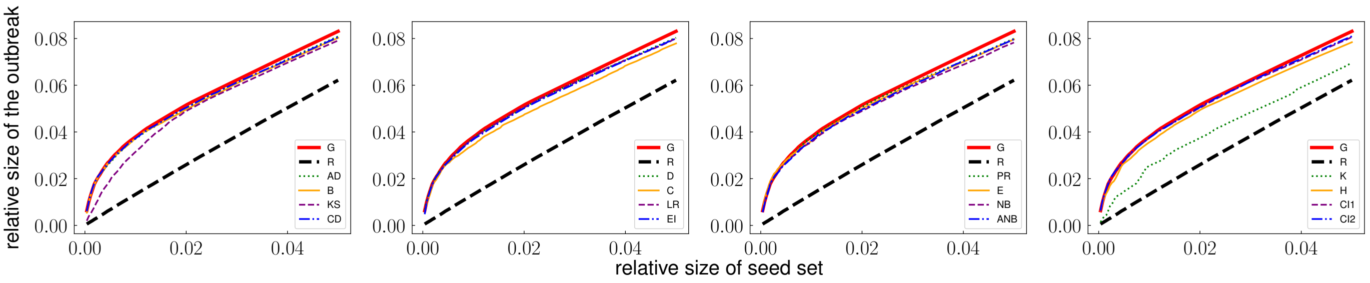

Figure 336: Japanese - p=0.5p_c

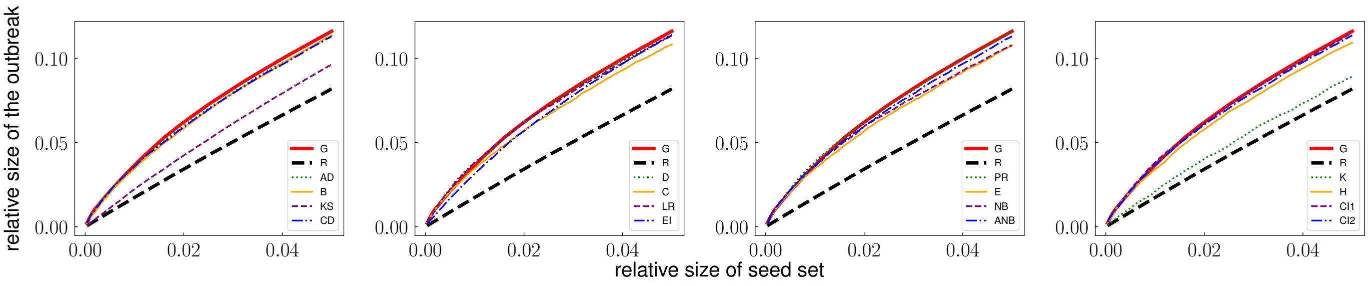

Figure 337: Williams - p=0.5p_c

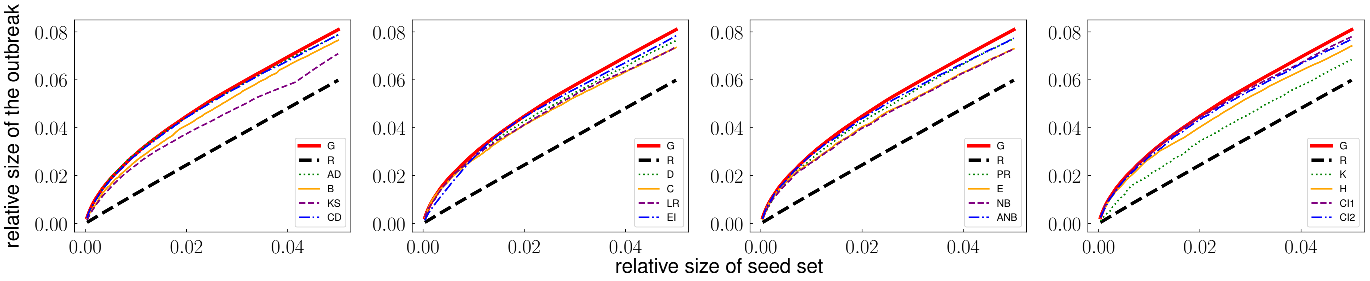

Figure 338: Open flights - p=0.5p_c

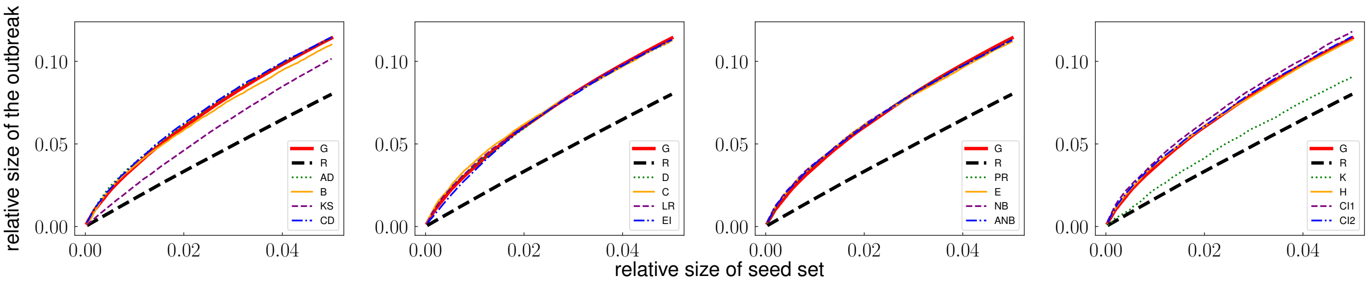

Figure 339: Oberlin - p=0.5p_c

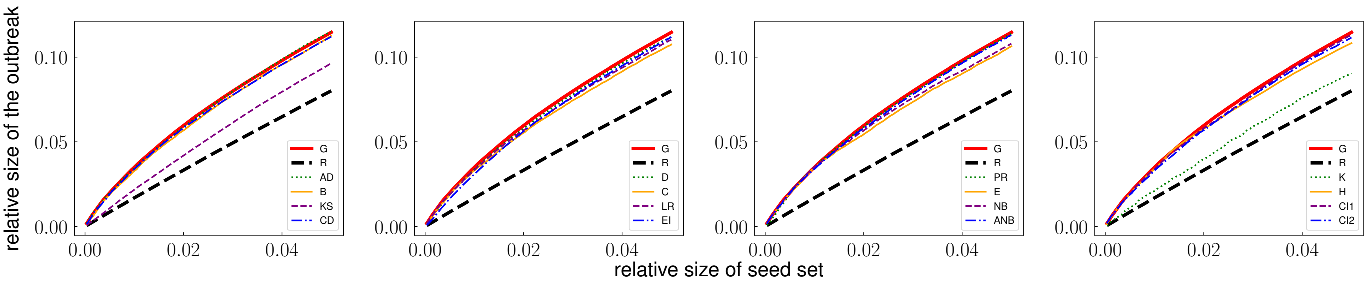

Figure 340: Smith - p=0.5p_c

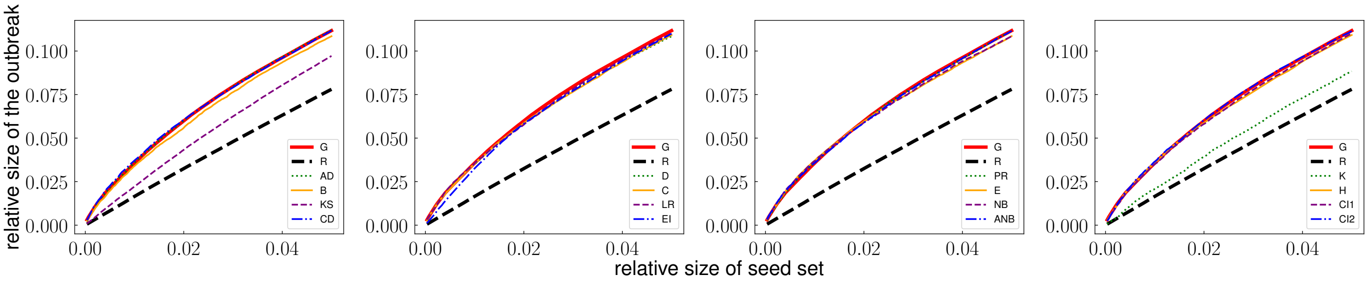

Figure 341: Wellesley - p=0.5p_c

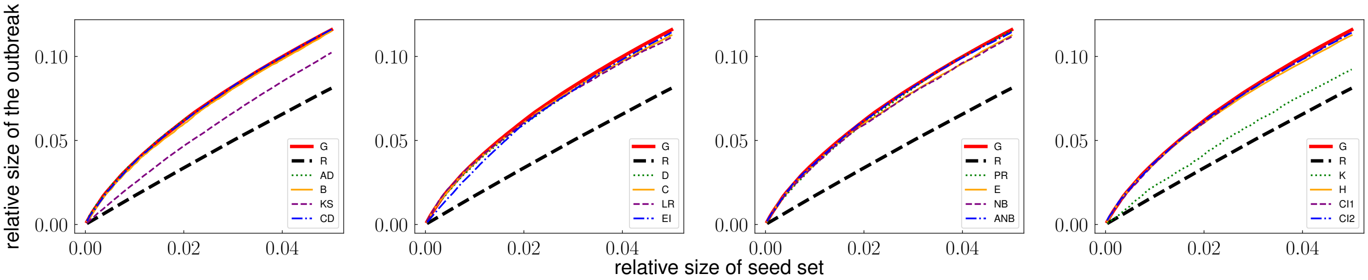

Figure 342: Vassar - p=0.5p_c

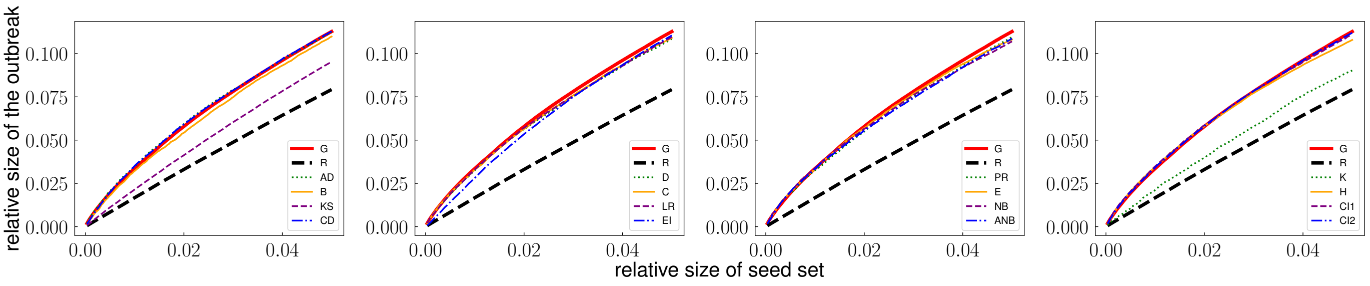

Figure 343: Middlebury - p=0.5p_c

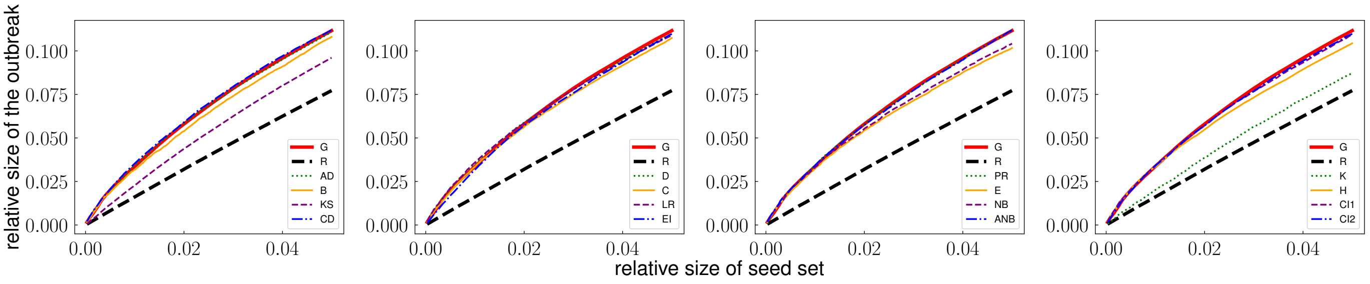

Figure 344: Pepperdine - p=0.5p_c

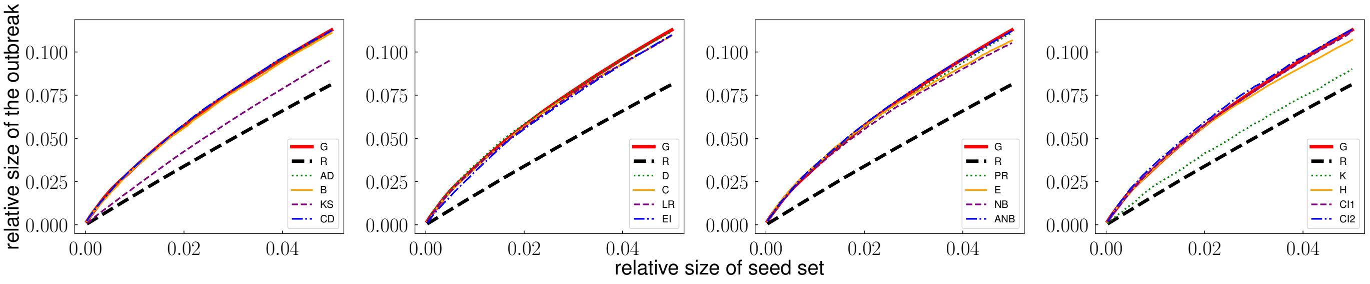

Figure 345: Colgate - p=0.5p_c

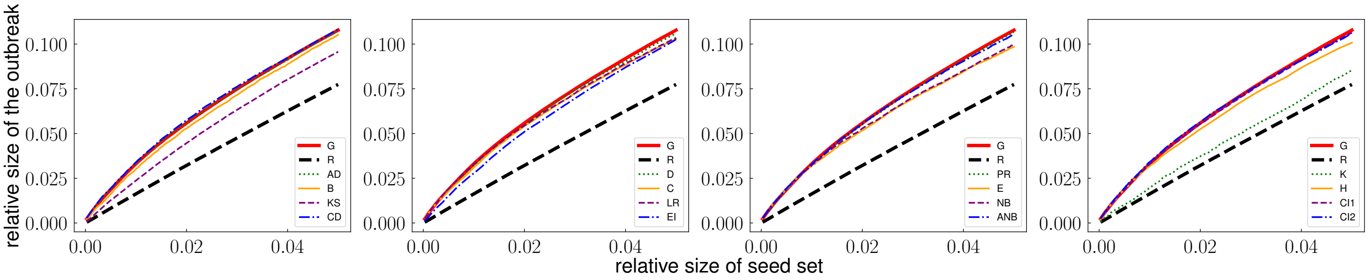

Figure 346: Santa - p=0.5p_c

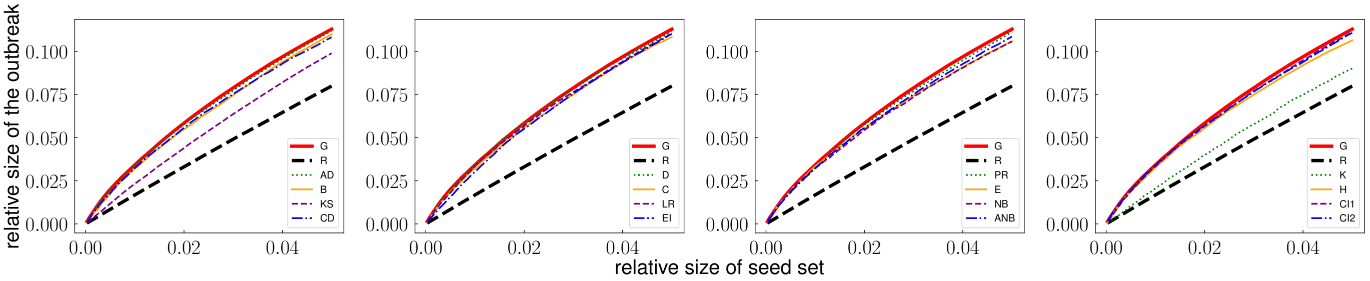

Figure 347: Wesleyan - p=0.5p_c

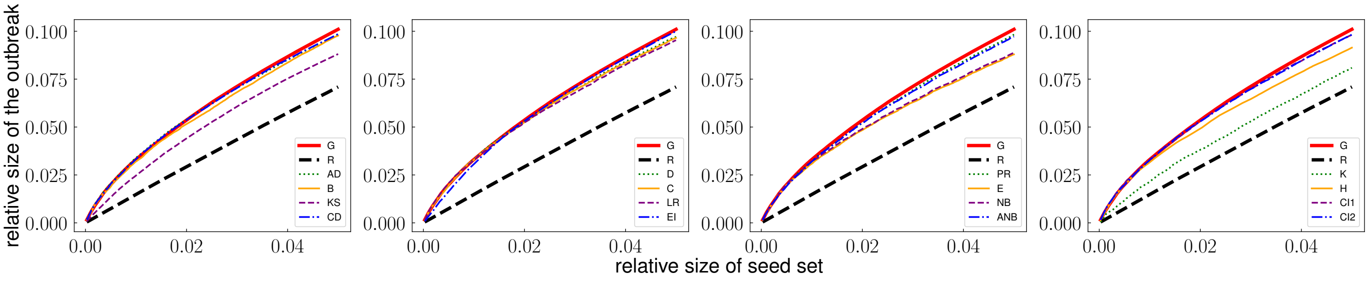

Figure 348: Mich - p=0.5p_c

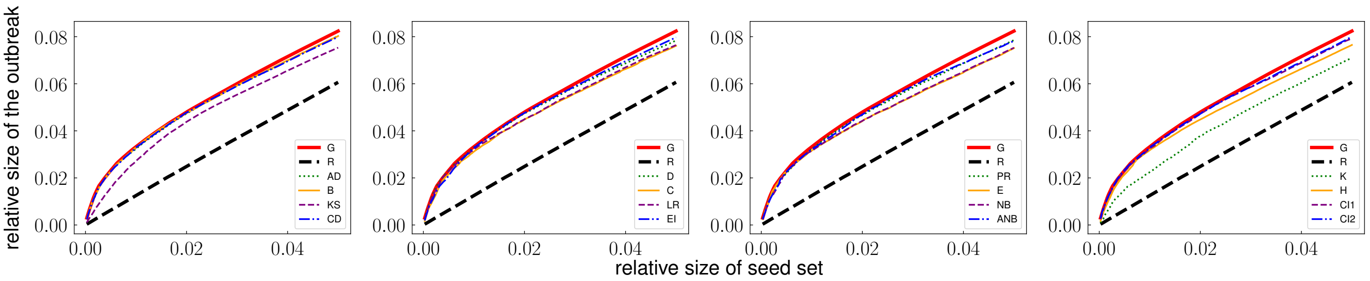

Figure 349: Bitcoin Alpha - $p=0.5p_c$

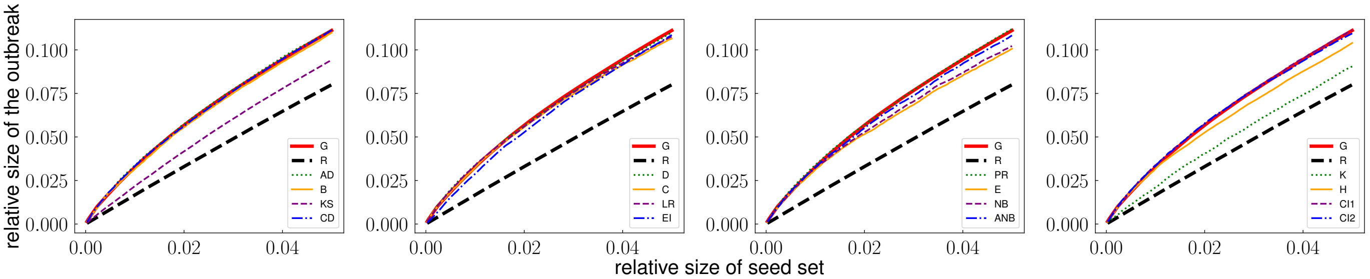

Figure 350: Bucknell - $p=0.5p_c$

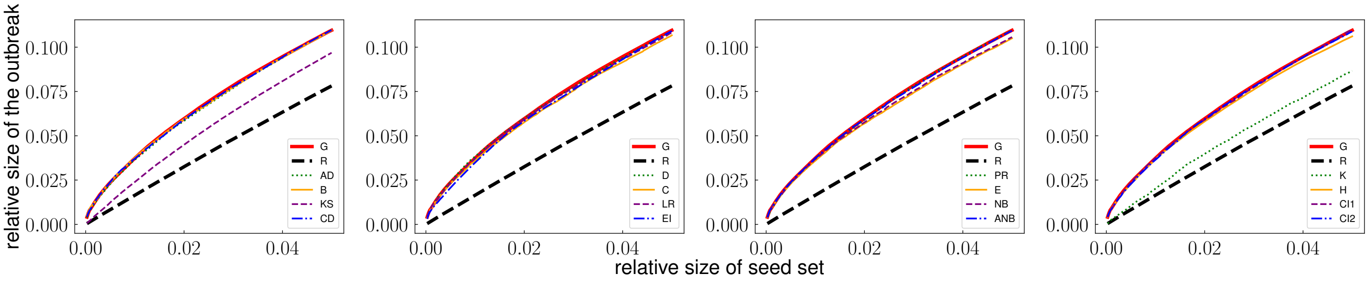

Figure 351: Brandeis - $p=0.5p_c$

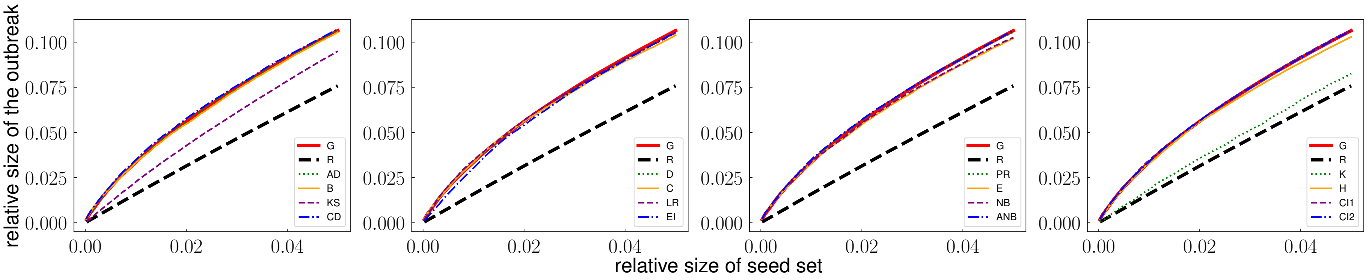

Figure 352: Howard - p=0.5p$_c$

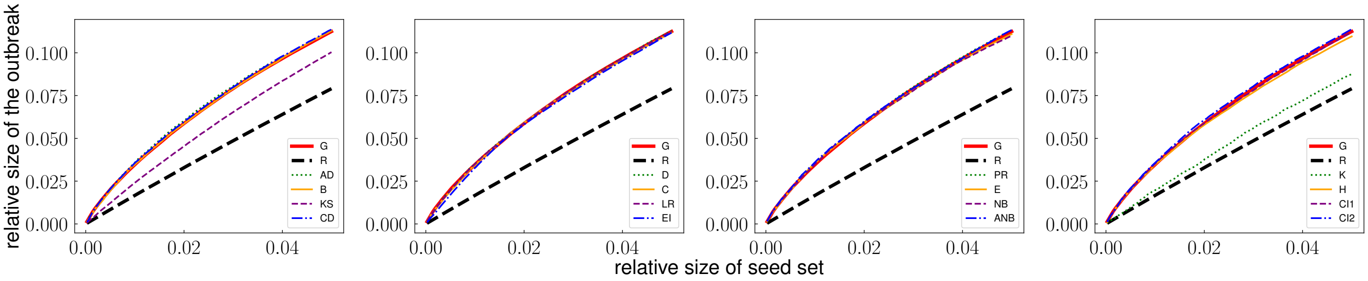

Figure 353: Rice - p=0.5p$_c$

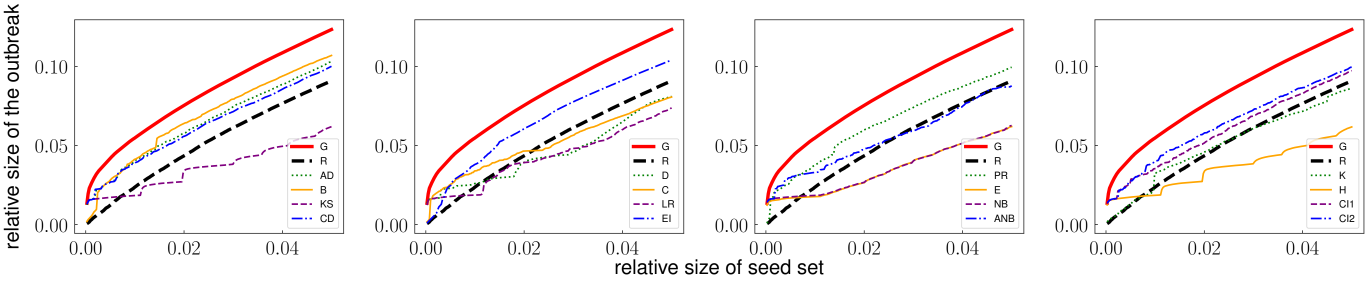

Figure 354: GR-QC, 1993-2003 - p=0.5p$_c$

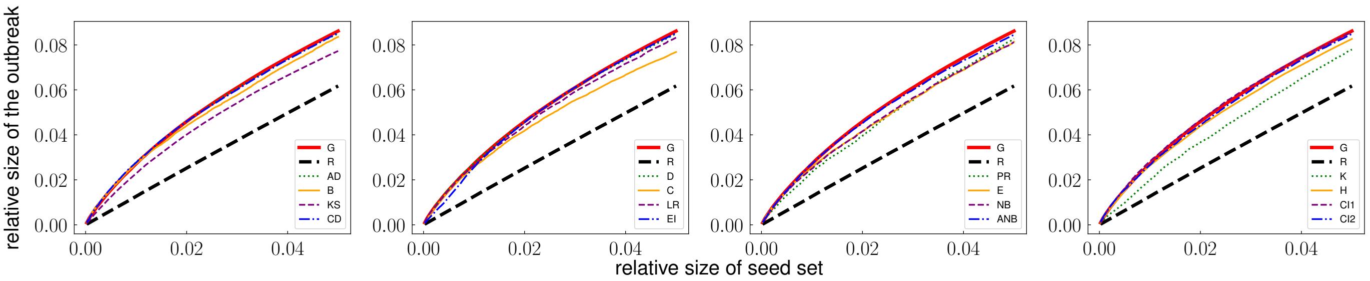

Figure 355: Tennis - p=0.5p_c

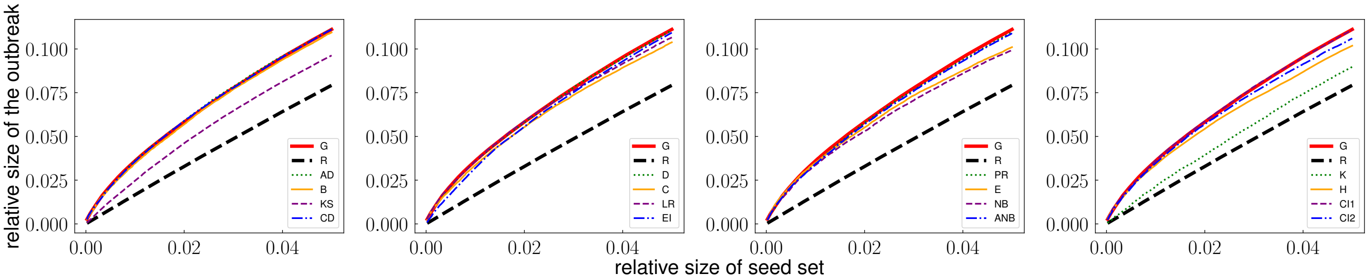

Figure 356: Rochester - p=0.5p_c

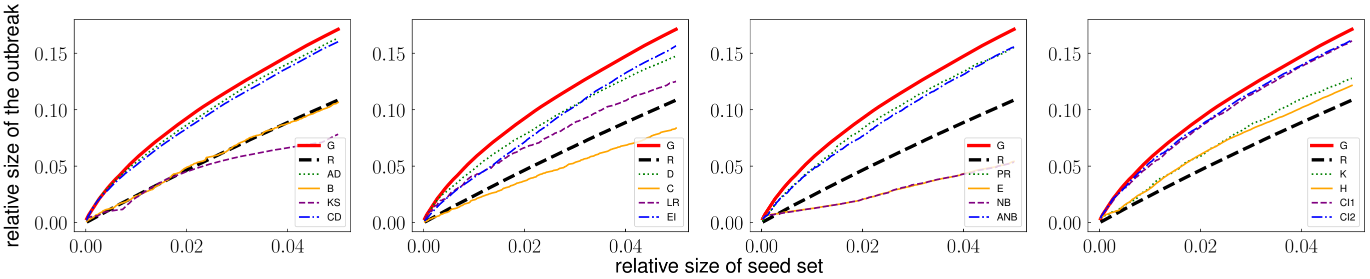

Figure 357: US Power grid - p=0.5p_c

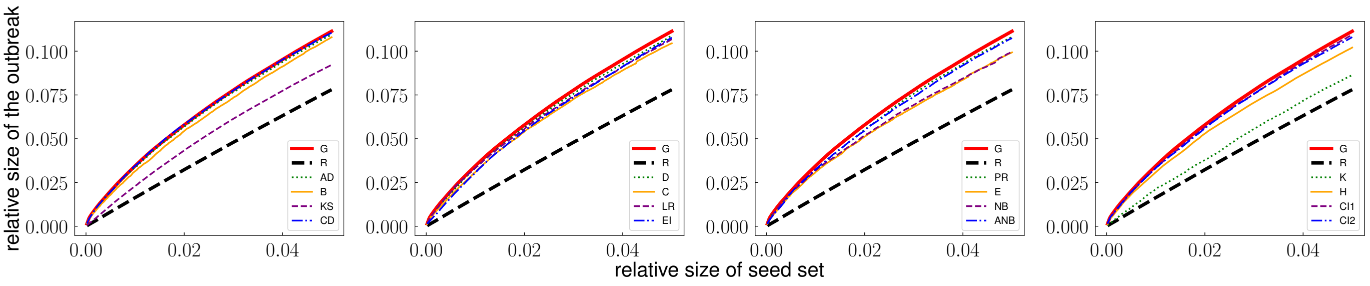

Figure 358: Lehigh - p=0.5p_c

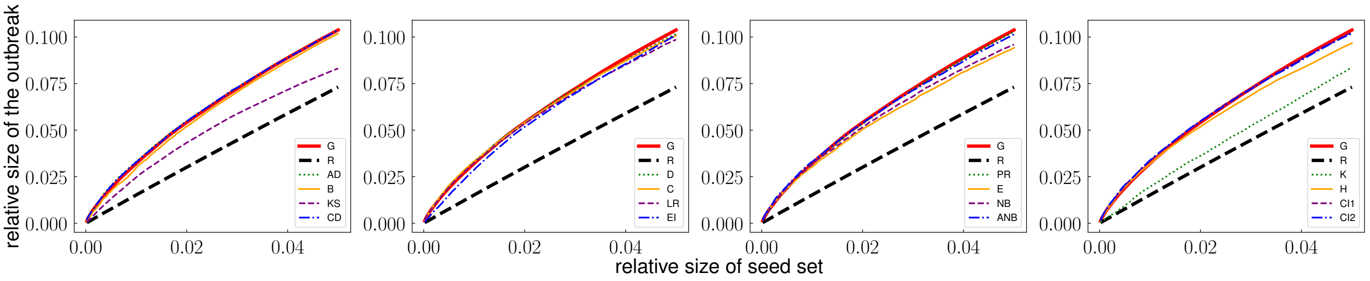

Figure 359: Johns Hopkins - p=0.5p_c

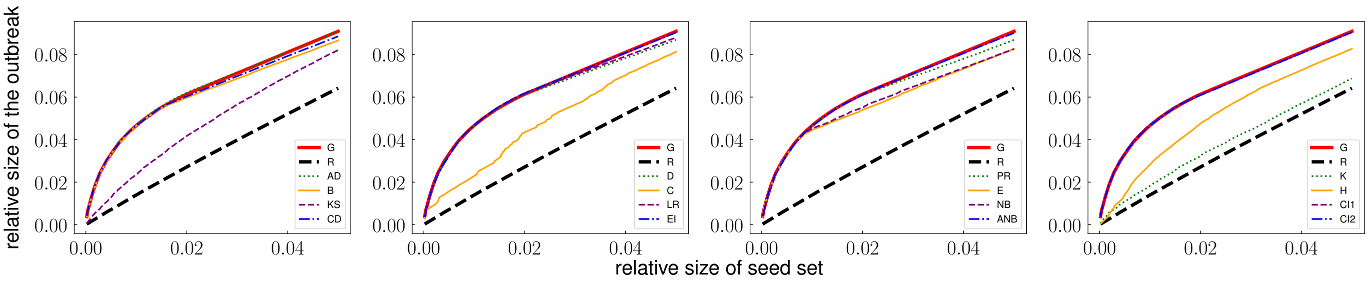

Figure 360: HT09 - p=0.5p_c

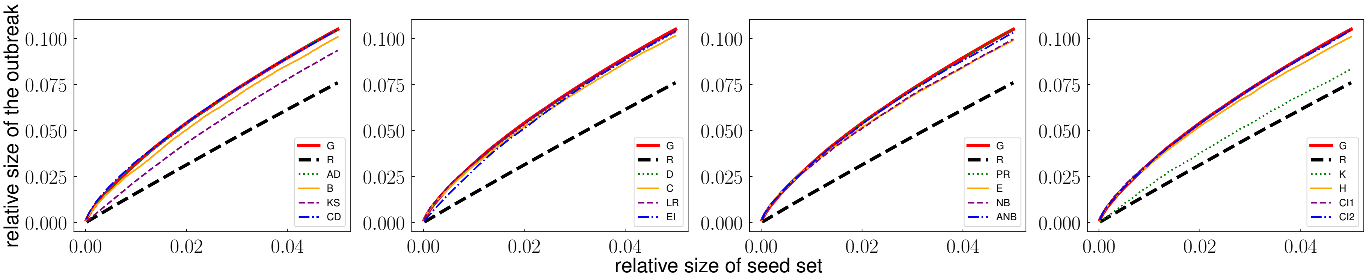

Figure 361: Wake - p=0.5p_c

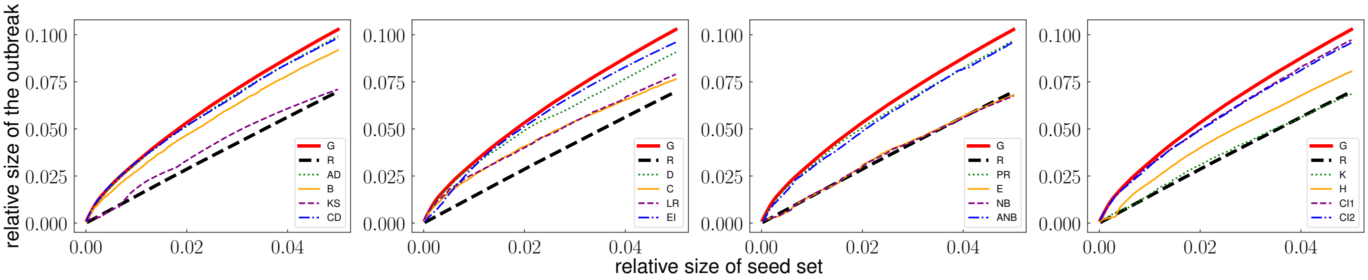

Figure 362: Hep-Th, 1995-1999 - p=0.5p_c

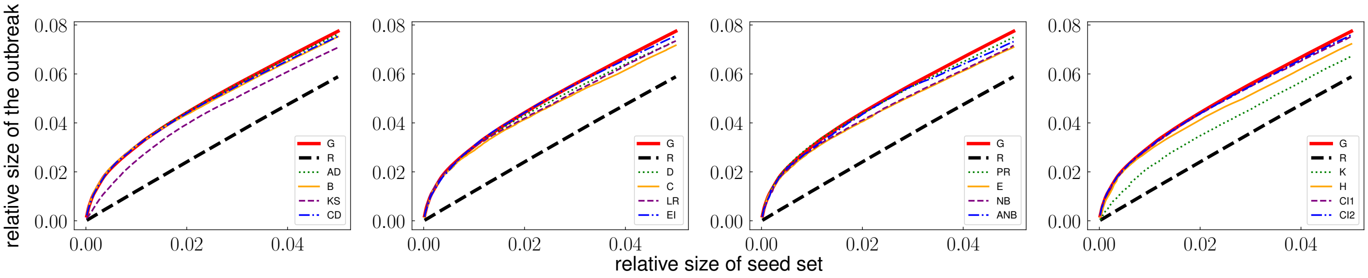

Figure 363: Bitcoin OTC - p=0.5p_c

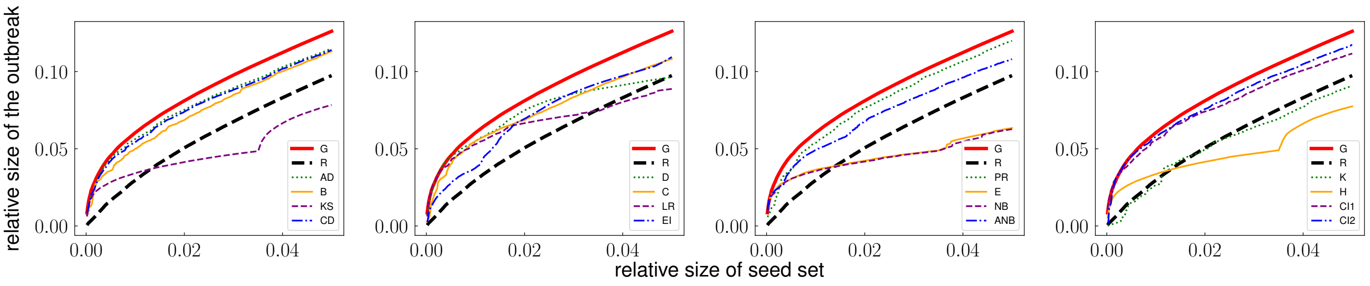

Figure 364: Reactome - p=0.5p_c

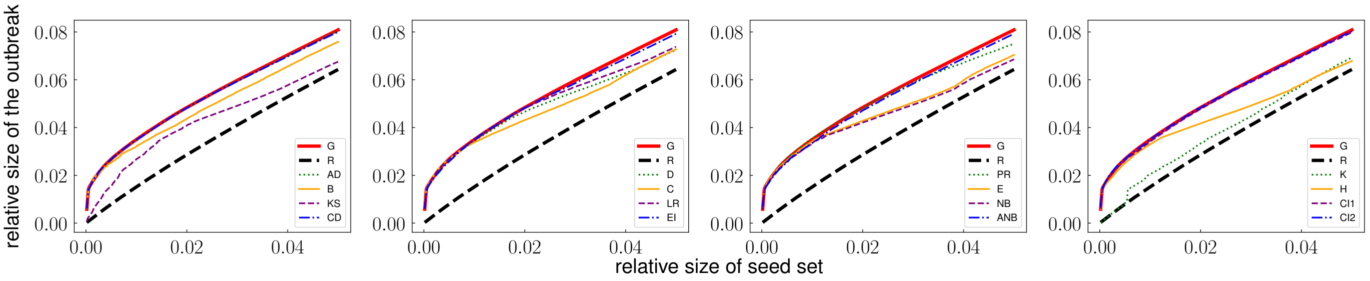

Figure 365: Jung - p=0.5p_c

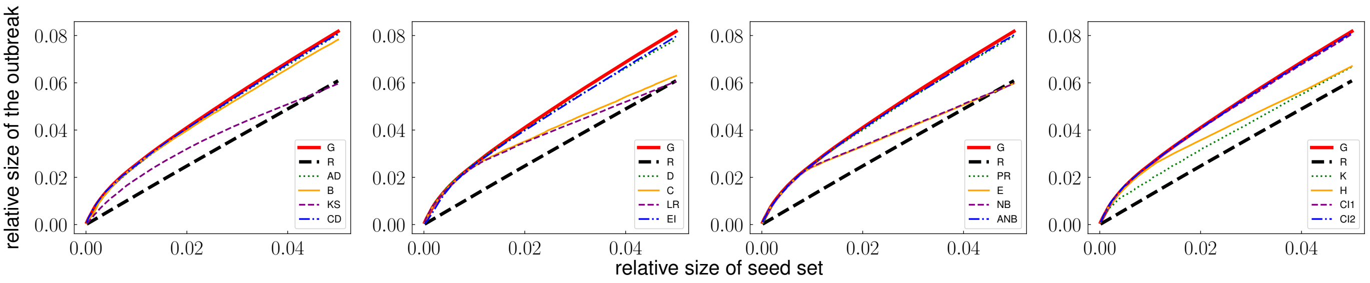

Figure 366: Gnutella, Aug. 8, 2002 - p=0.5p_c

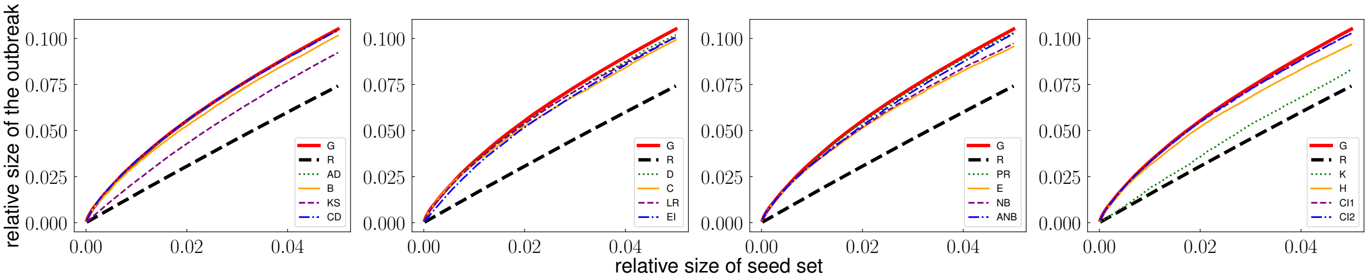

Figure 367: American - p=0.5p_c

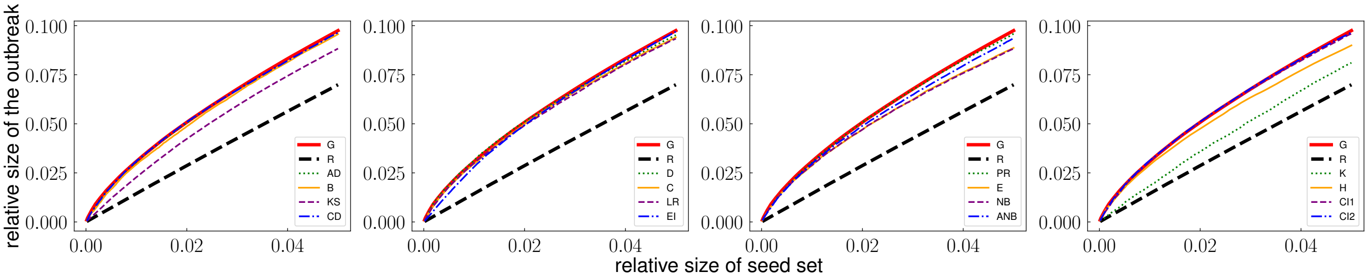

Figure 368: MIT - p=0.5p_c

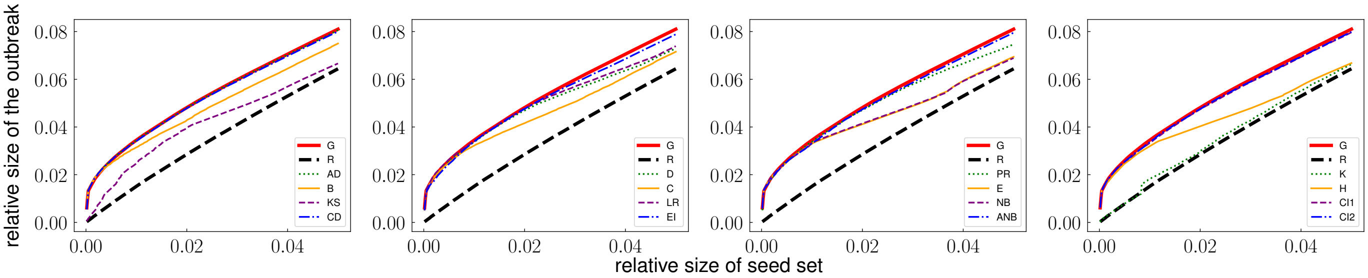

Figure 369: JDK - p=0.5p_c

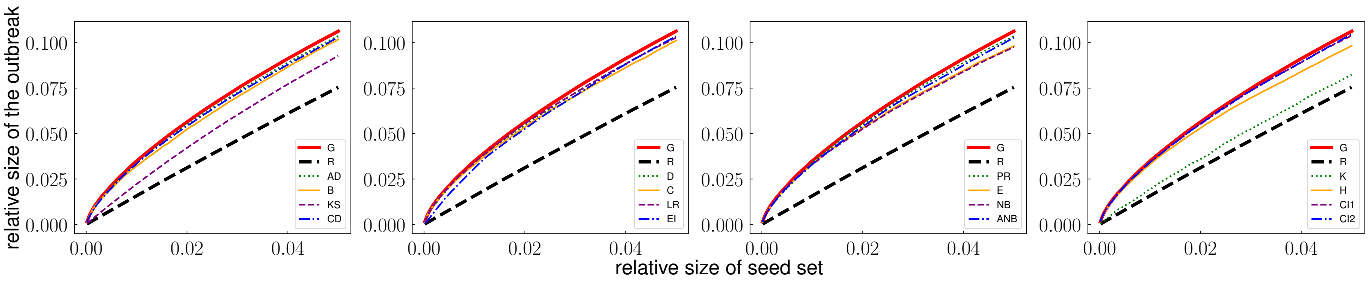

Figure 370: William - p=0.5p_c

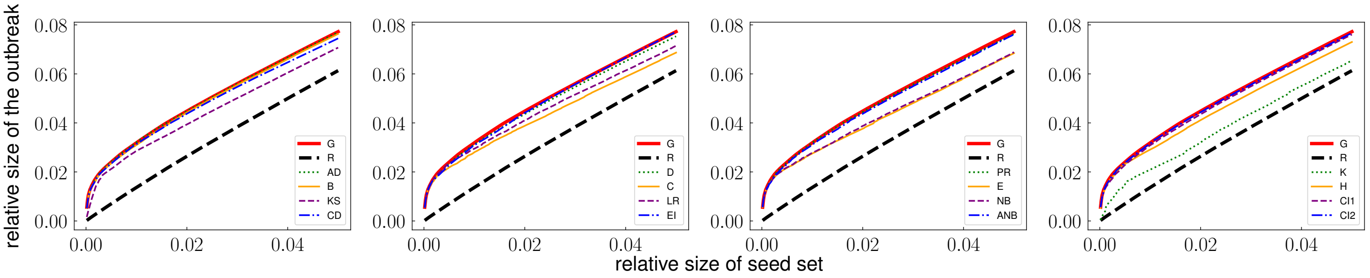

Figure 371: AS Oregon - p=0.5p_c

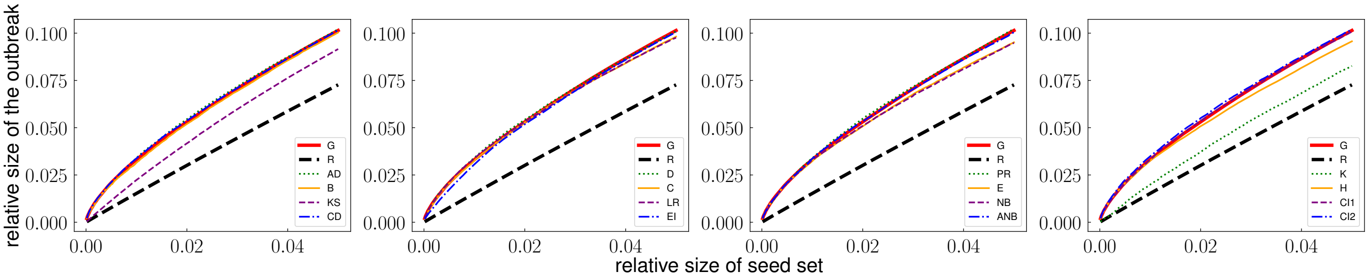

Figure 372: UChicago - p=0.5p_c

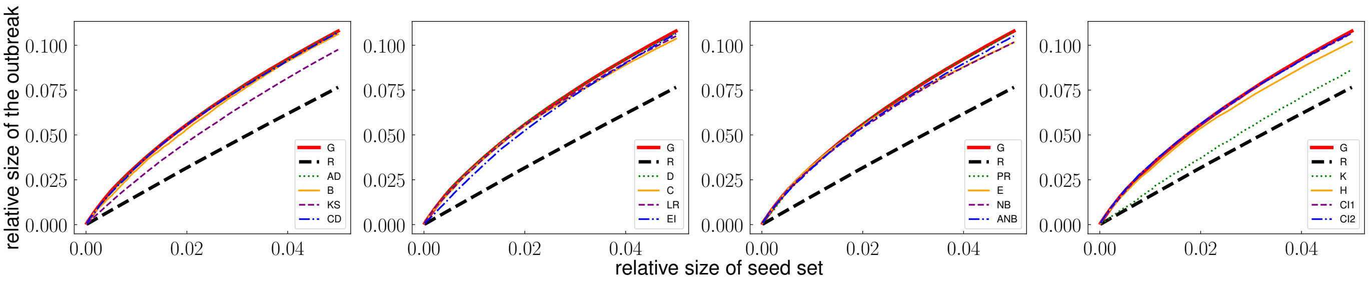

Figure 373: Princeton - p=0.5p_c

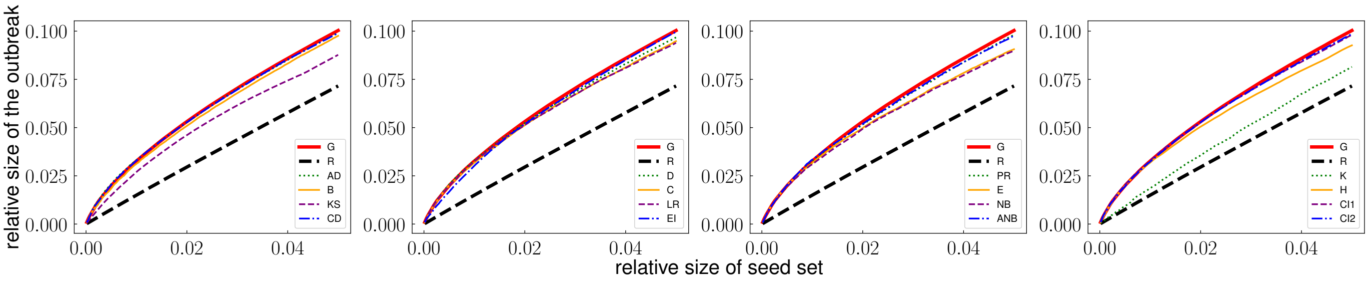

Figure 374: Carnegie - p=0.5p_c

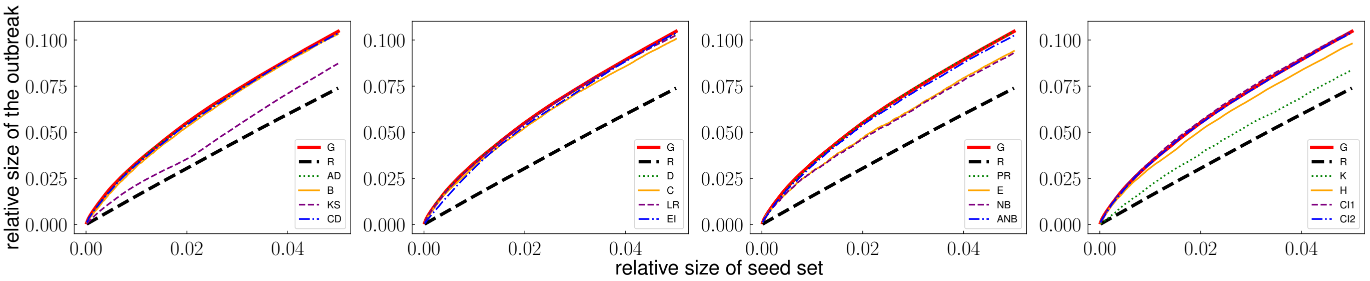

Figure 375: Tufts - p=0.5p_c

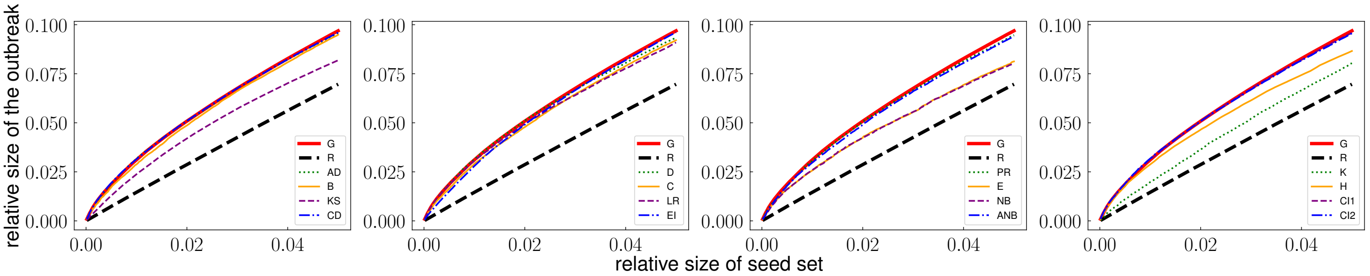

Figure 376: UC - p=0.5p_c

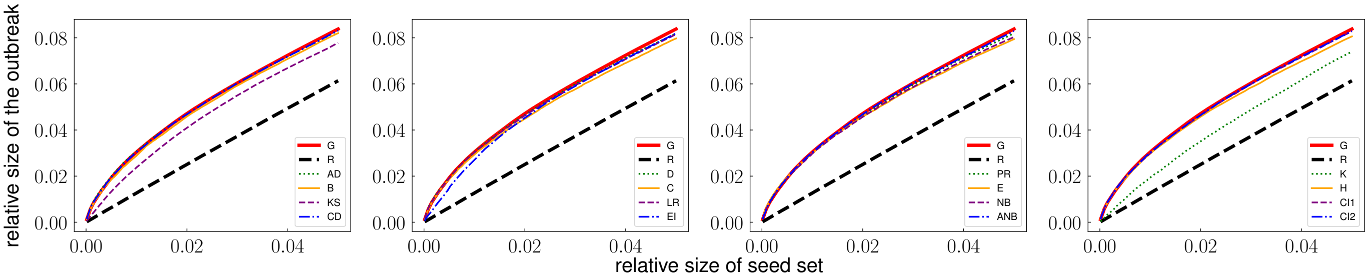

Figure 377: Wikipedia elections - p=0.5p_c

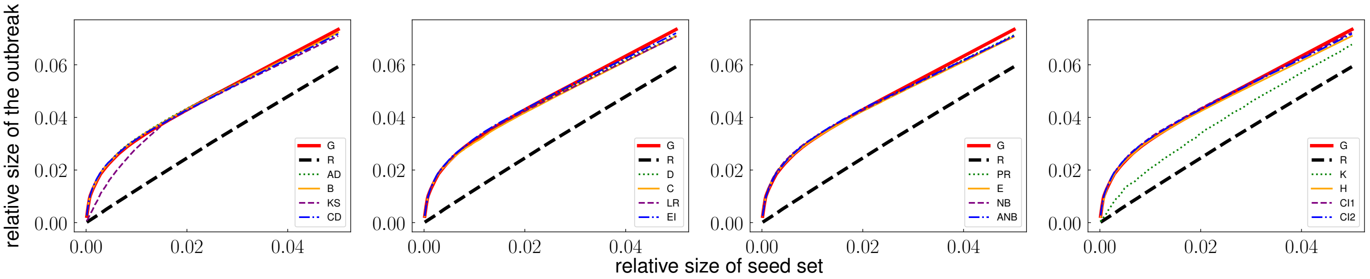

Figure 378: English - p=0.5p_c

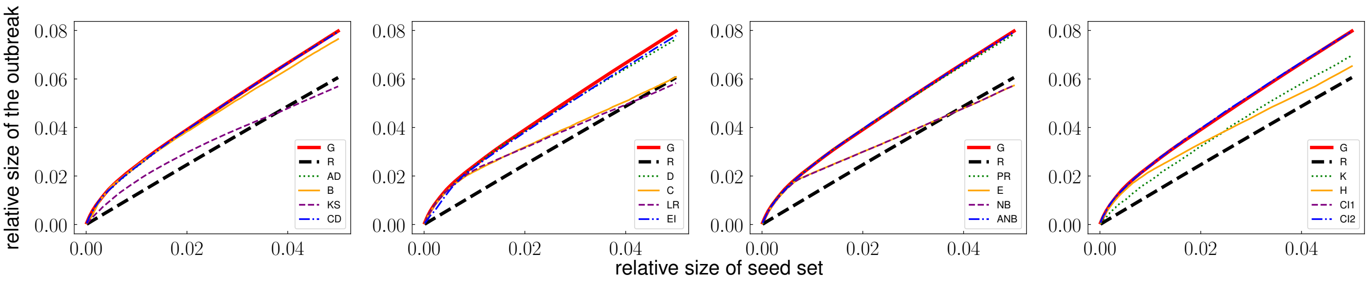

Figure 379: Gnutella, Aug. 9, 2002 - p=0.5p$_c$

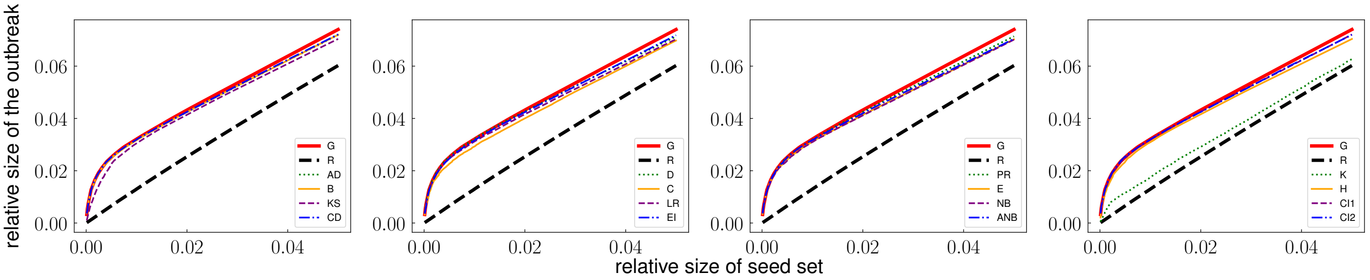

Figure 380: French - p=0.5p$_c$

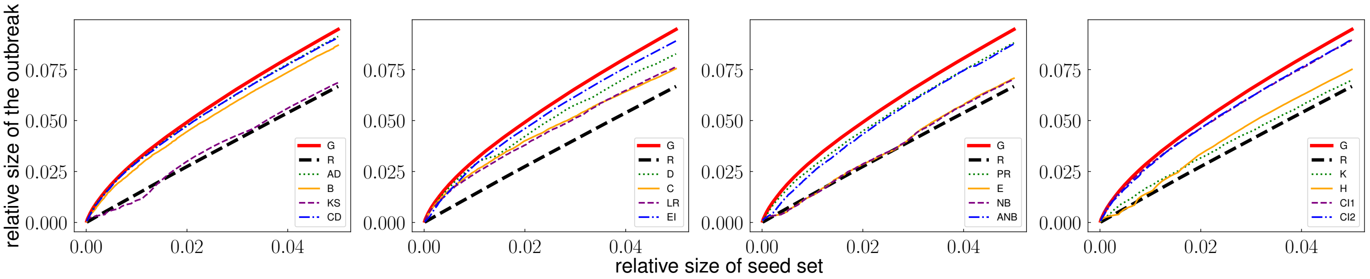

Figure 381: Hep-Th, 1993-2003 - p=0.5p$_c$

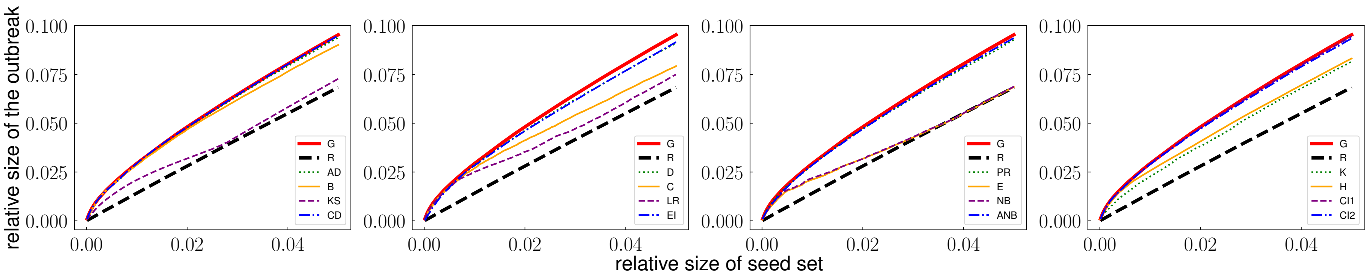

Figure 382: Gnutella, Aug. 6, 2002 - p=0.5p_c

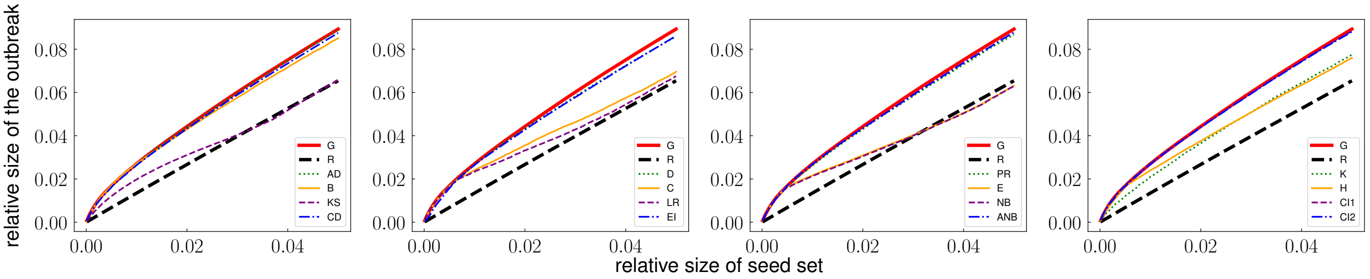

Figure 383: Gnutella, Aug. 5, 2002 - p=0.5p_c

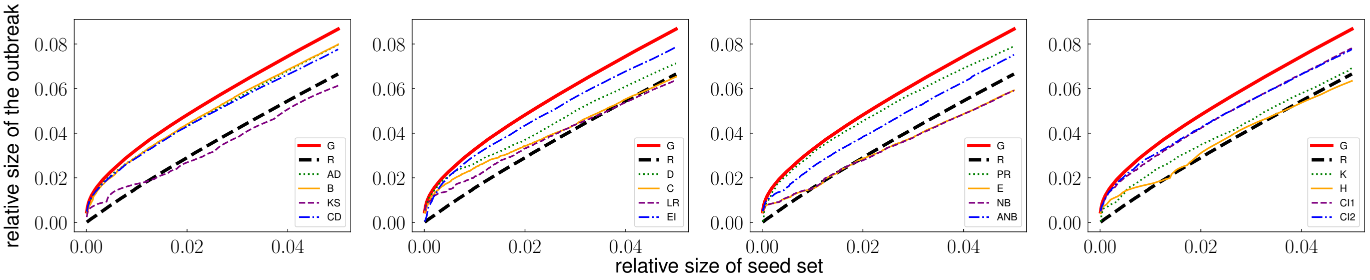

Figure 384: PGP - p=0.5p_c

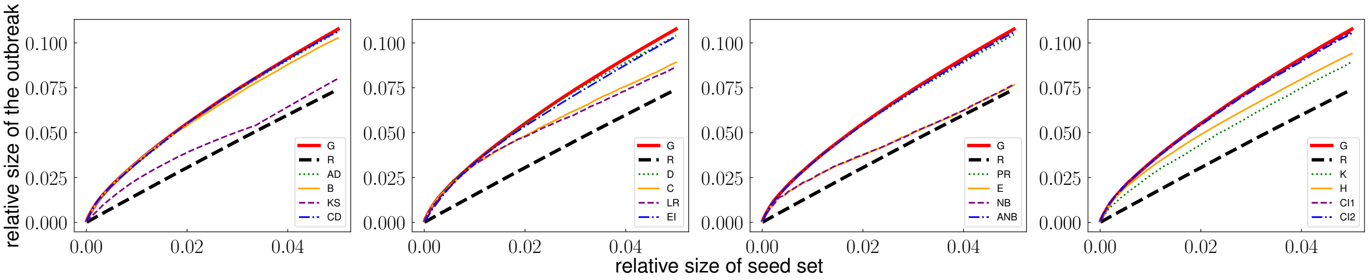

Figure 385: Gnutella, Aug. 4, 2002 - $p=0.5p_c$

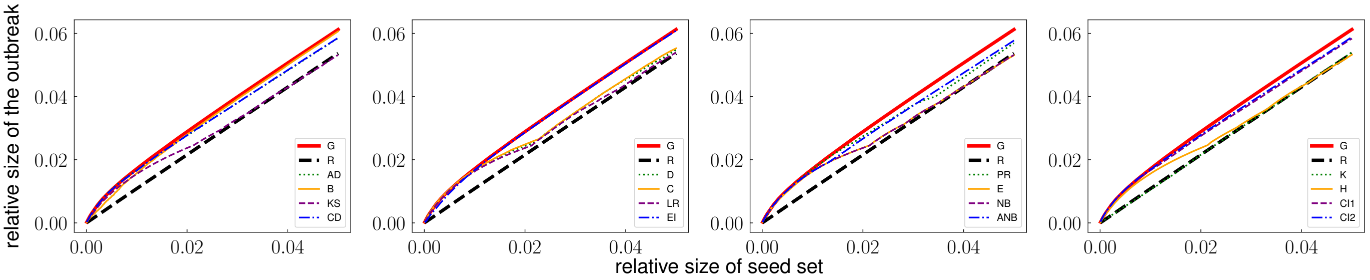

Figure 386: Hep-Ph, 1993-2003 - $p=0.5p_c$

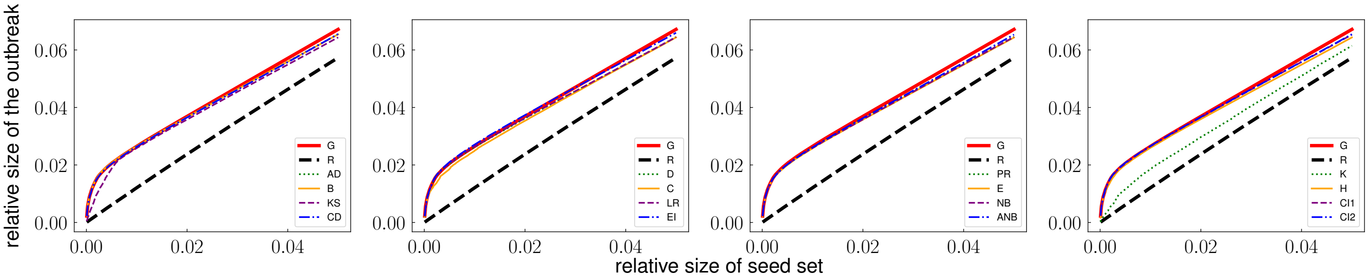

Figure 387: Spanish 1 - $p=0.5p_c$

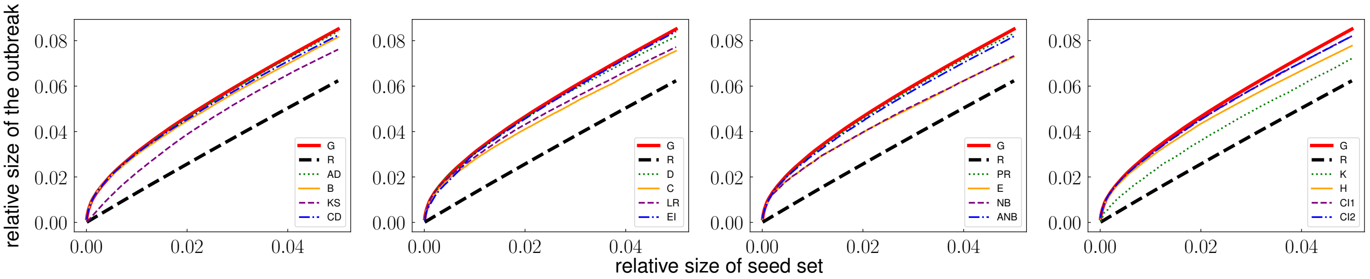

Figure 388: DBLP, citations - p=0.5p_c

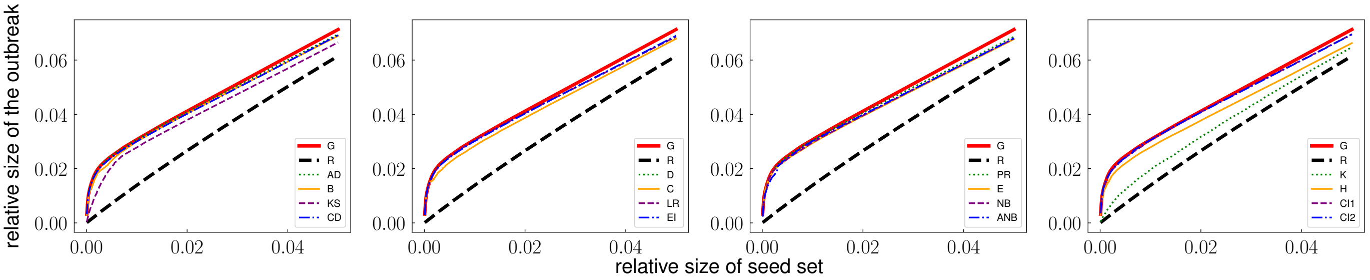

Figure 389: Spanish 2 - p=0.5p_c

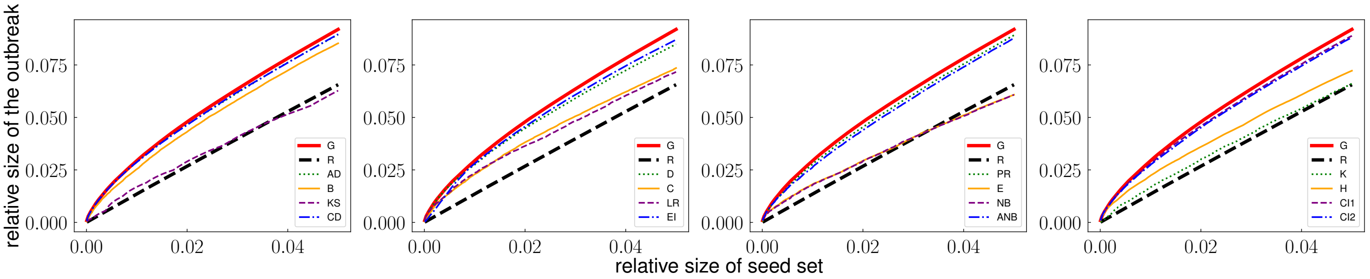

Figure 390: Cond-Mat, 1995-1999 - p=0.5p_c

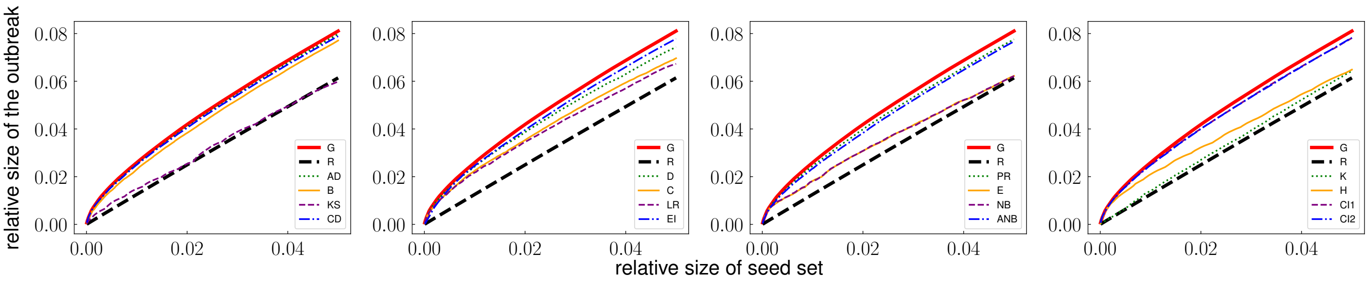

Figure 391: Astrophysics - p=0.5p_c

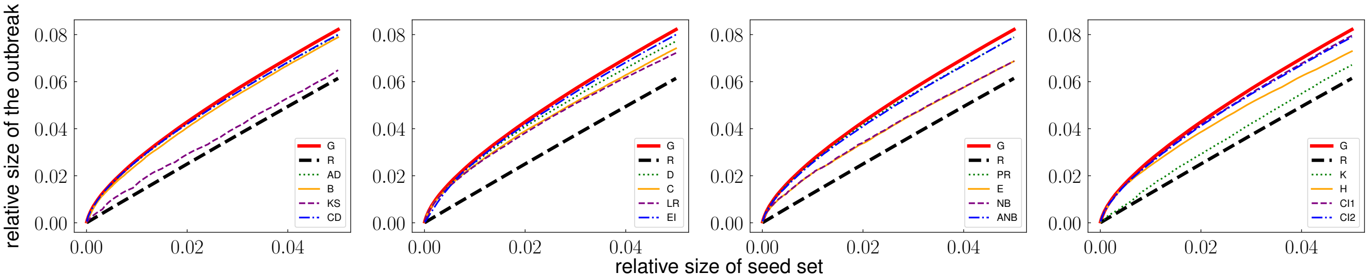

Figure 392: AstroPhys, 1993-2003 - p=0.5p_c

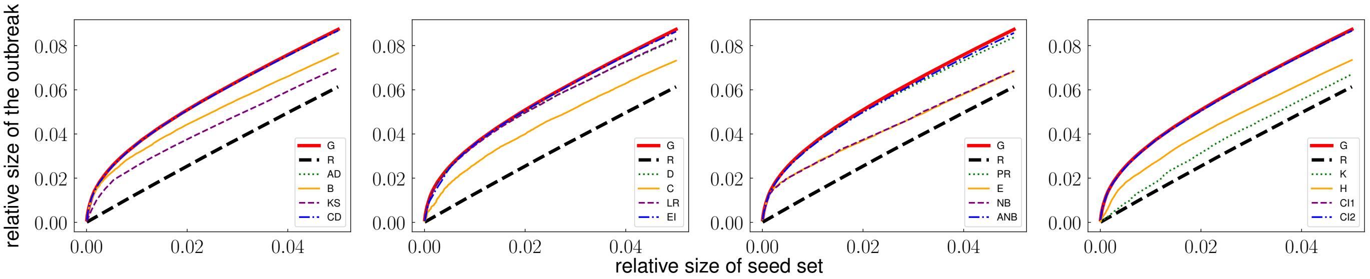

Figure 393: Marvel - p=0.5p_c

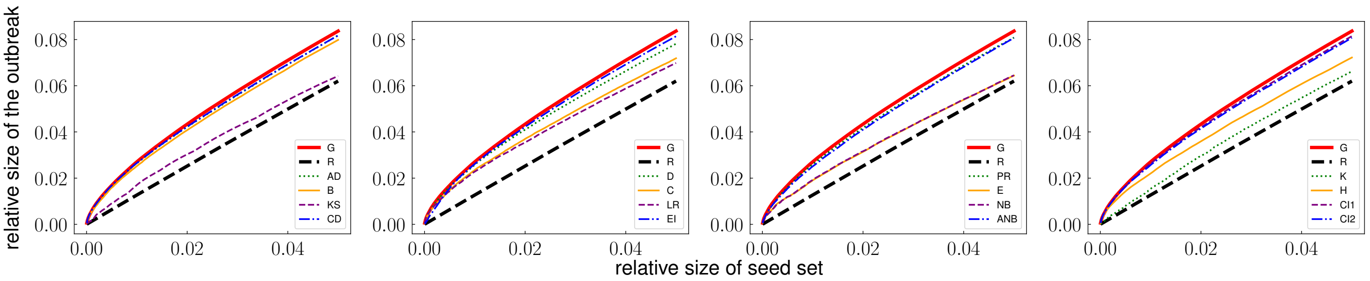

Figure 394: Cond-Mat, 1993-2003 - $p=0.5p_c$

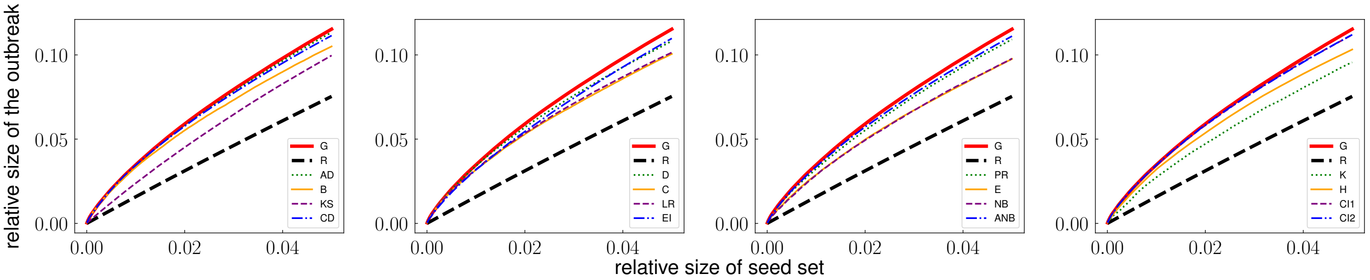

Figure 395: Gnutella, Aug. 25, 2002 - $p=0.5p_c$

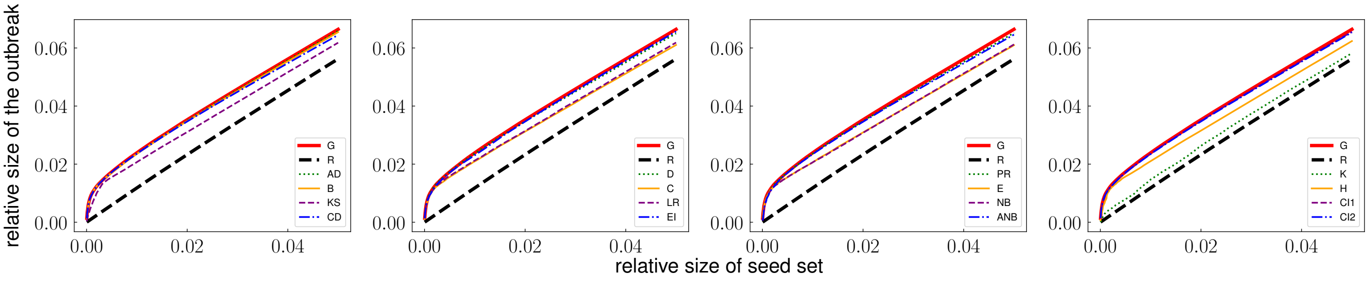

Figure 396: Internet - $p=0.5p_c$

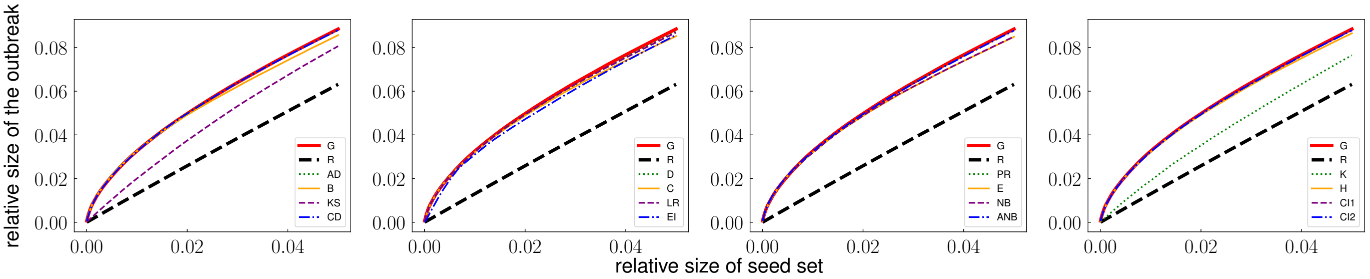

Figure 397: Thesaurus - p=0.5p_c

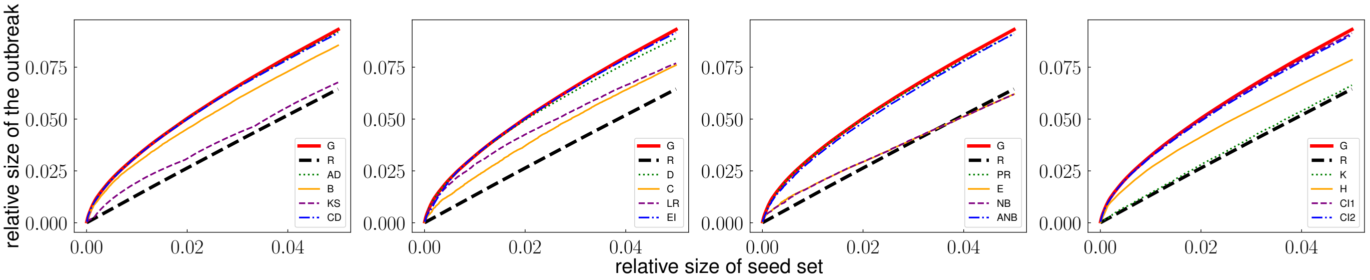

Figure 398: Cora - p=0.5p_c

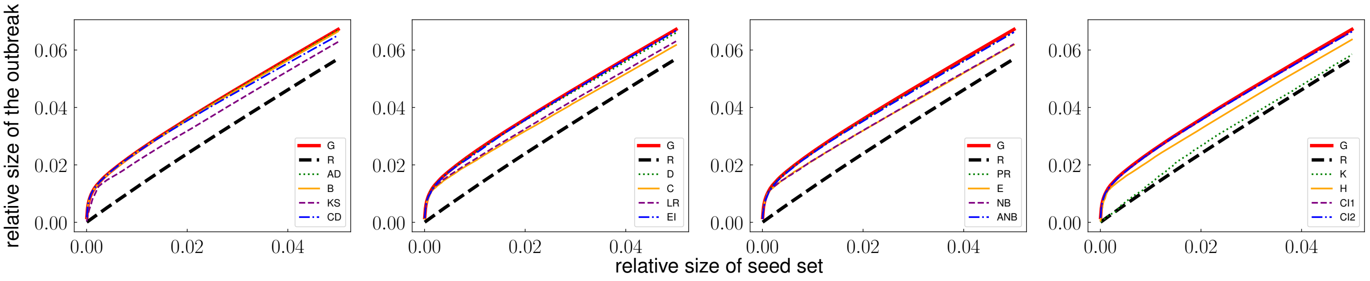

Figure 399: AS Caida - p=0.5p_c

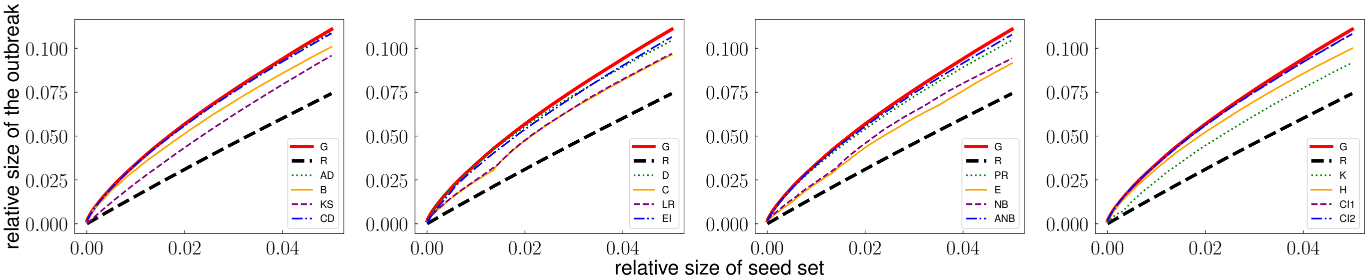

Figure 400: Gnutella, Aug. 24, 2002 - p=0.5p_c

3.1.2 Critical Regime

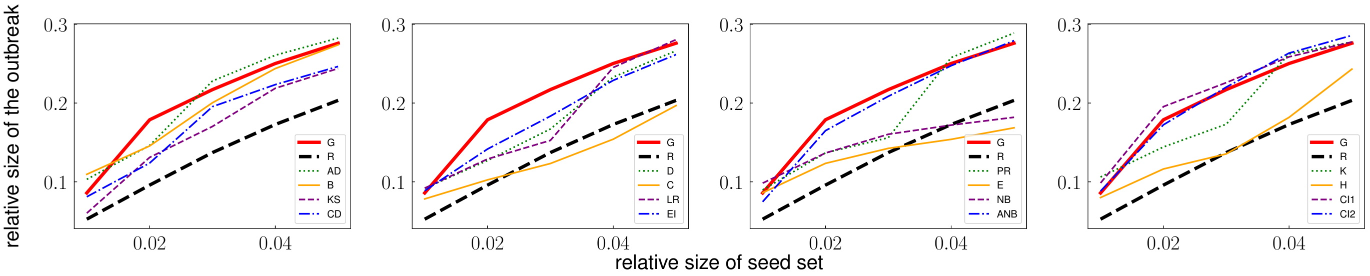

Figure 401: Political books - p=1.0p_c

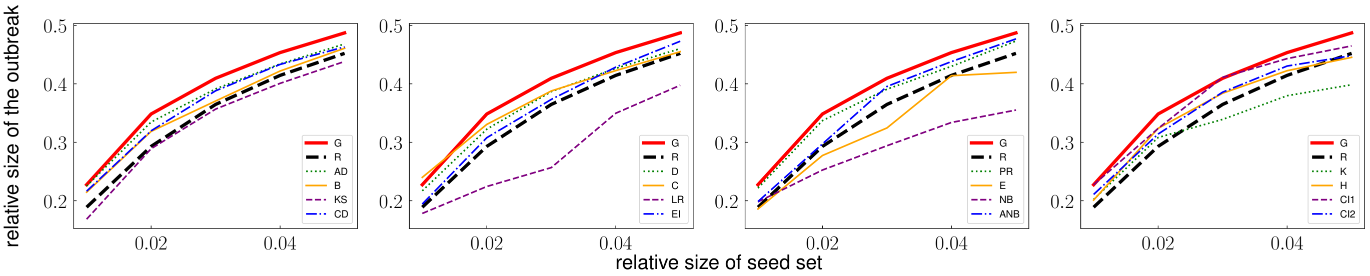

Figure 402: College football - p=1.0p_c

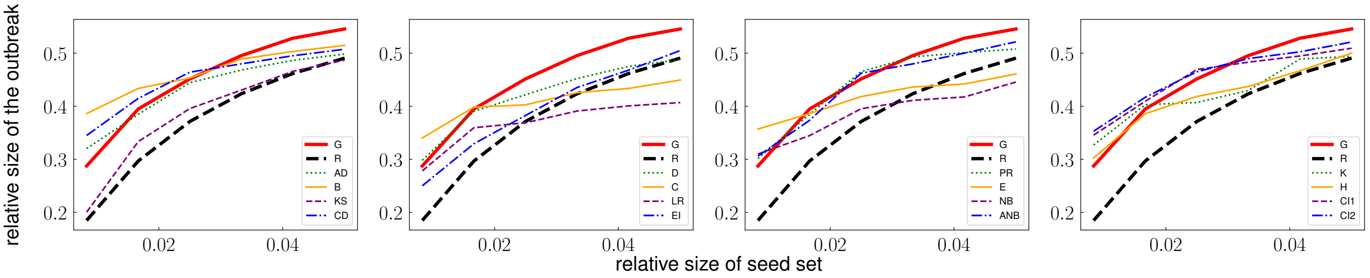

Figure 403: S208 - p=1.0p_c

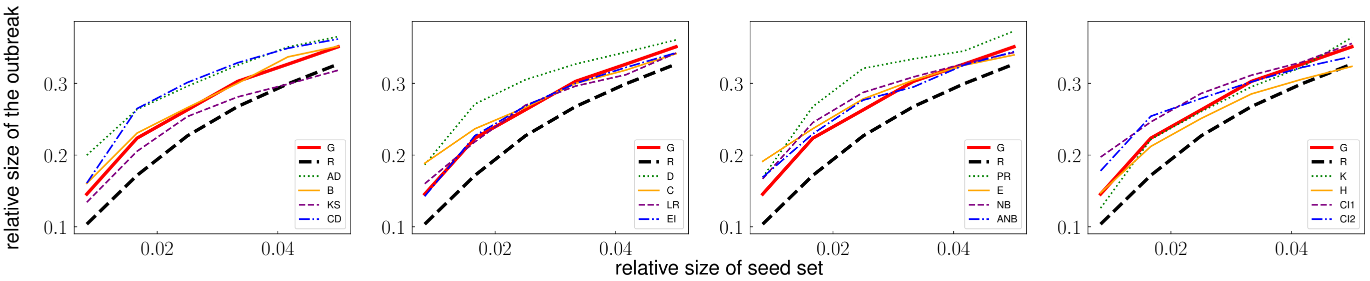

Figure 404: High school, 2011 - $p{=}1.0p_c$

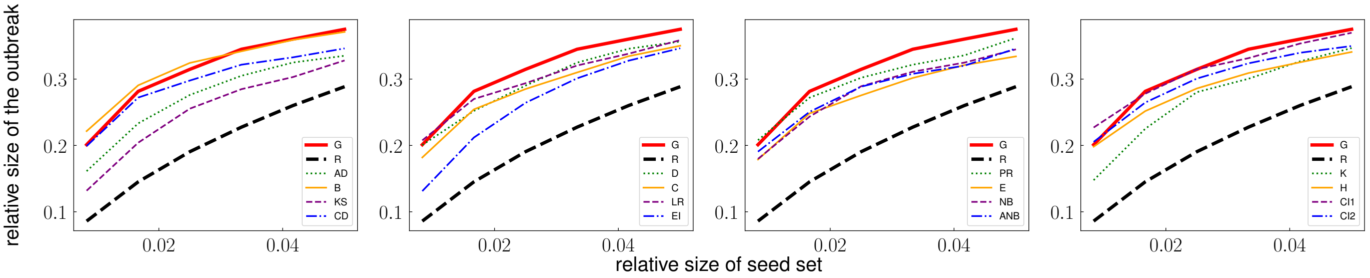

Figure 405: Bay Dry - $p{=}1.0p_c$

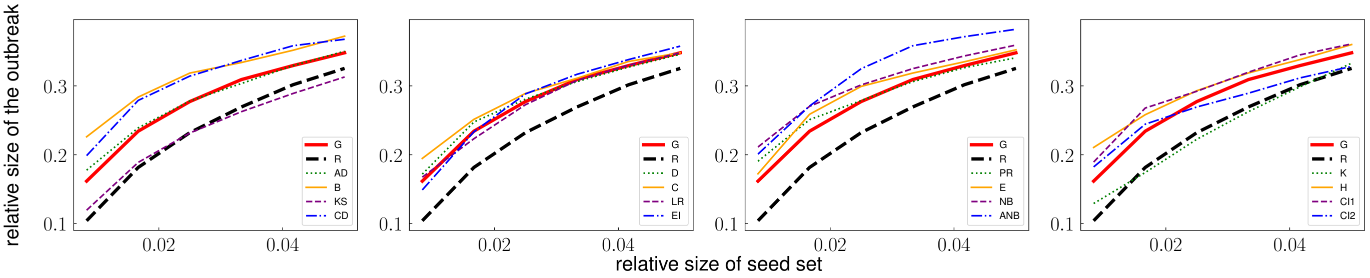

Figure 406: Bay Wet - $p{=}1.0p_c$

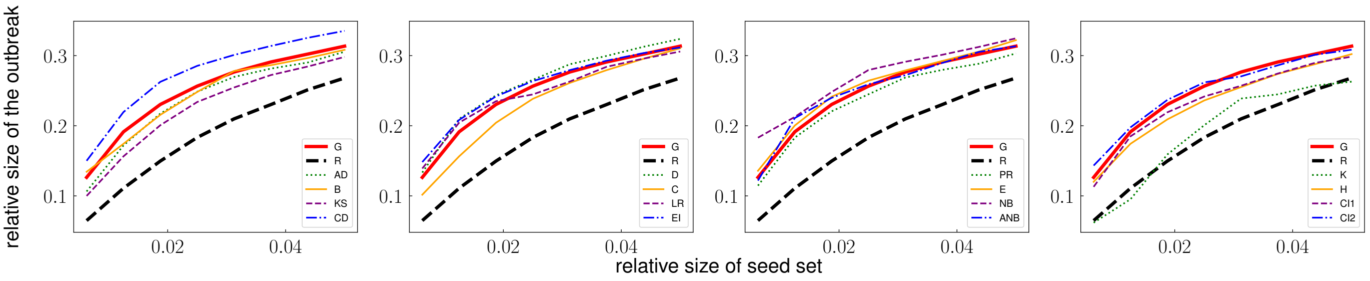

Figure 407: Radoslaw Email - p=1.0p_c

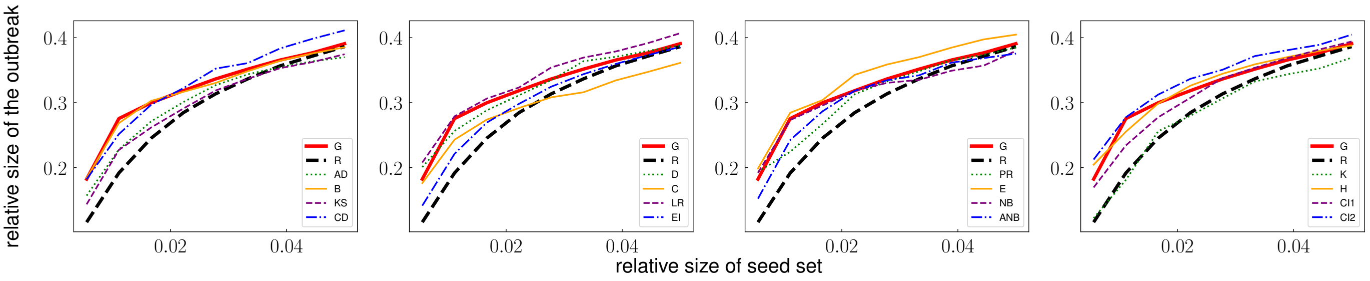

Figure 408: High school, 2012 - p=1.0p_c

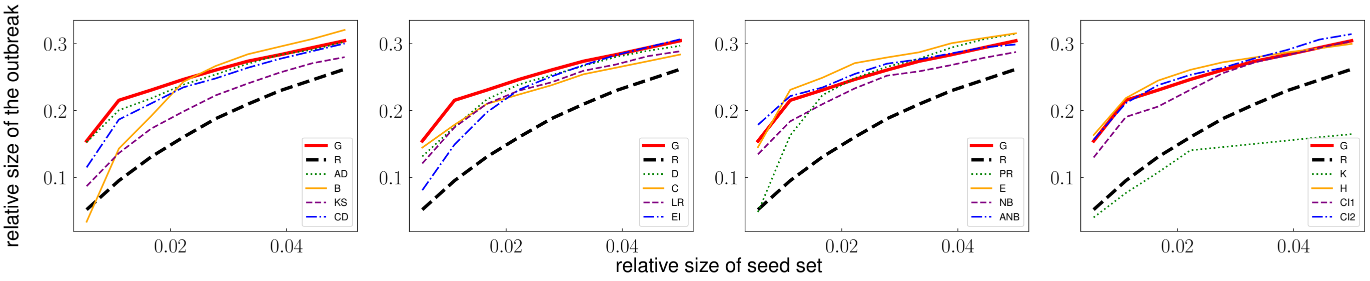

Figure 409: Little Rock Lake - p=1.0p_c

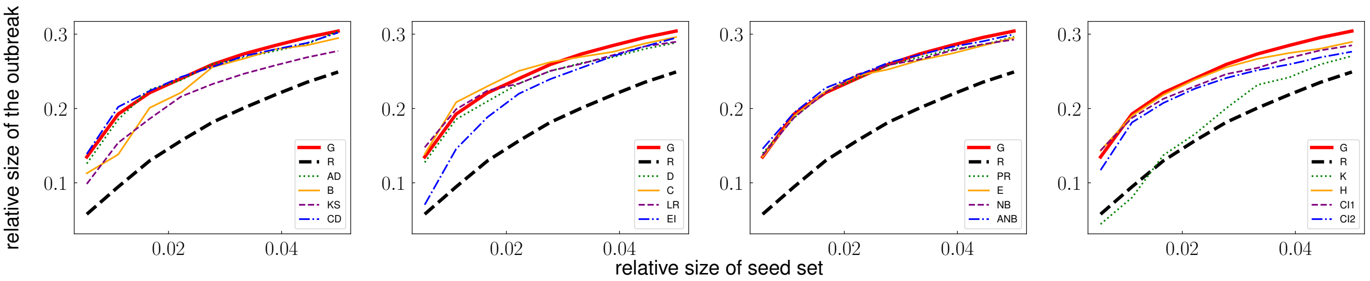

Figure 410: Jazz - p=1.0p_c

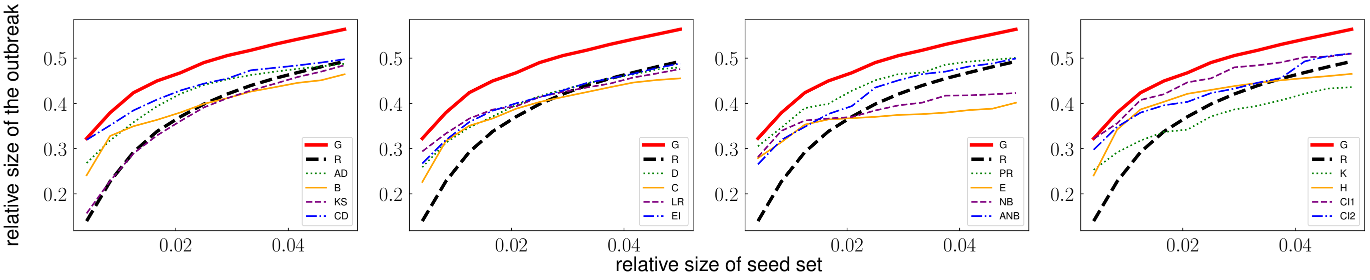

Figure 411: S420 - p=1.0p_c

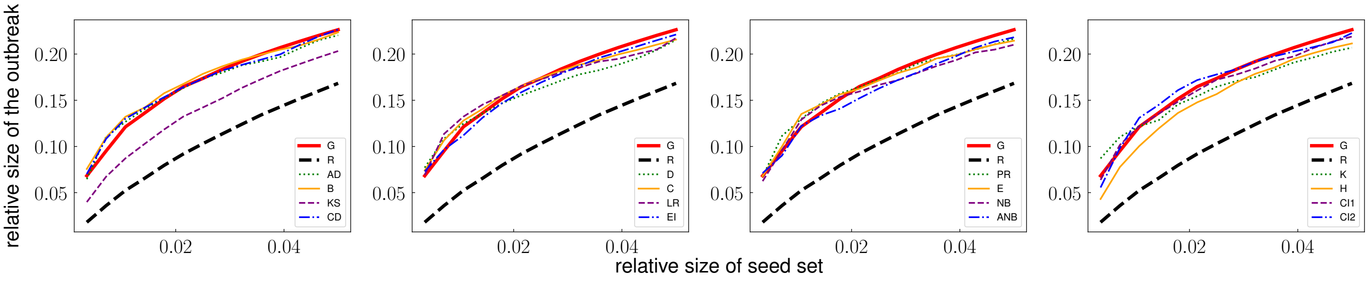

Figure 412: C. Elegans, neural - p=1.0p_c

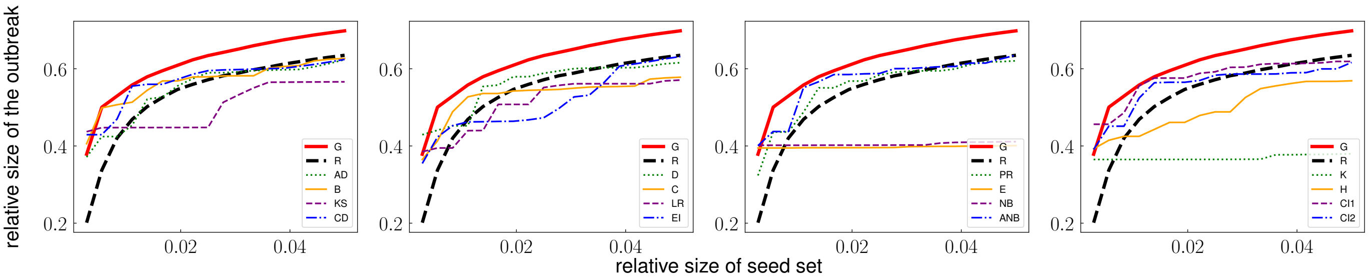

Figure 413: Network Science - p=1.0p_c

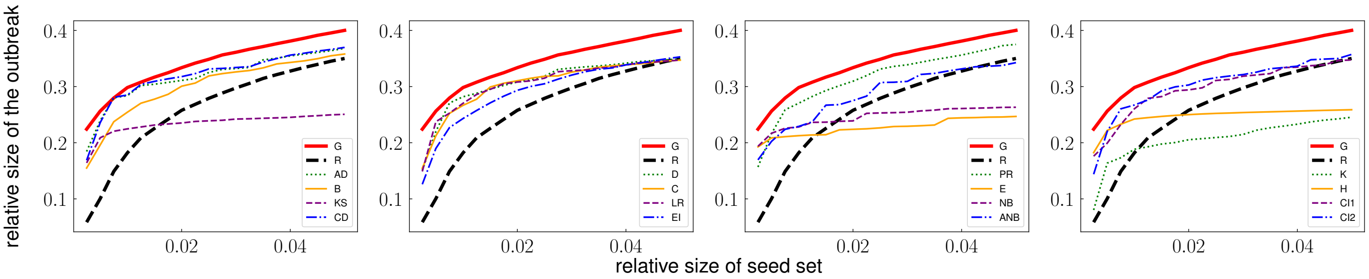

Figure 414: Dublin - p=1.0p_c

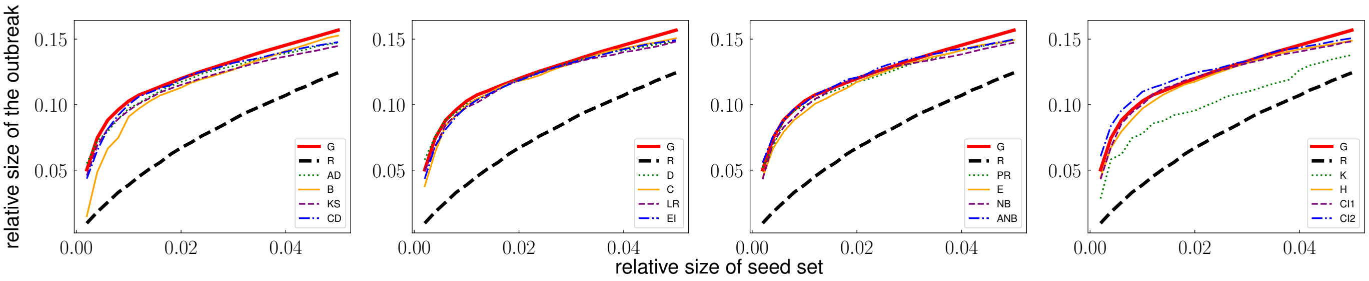

Figure 415: US Air Trasportation - p=1.0p_c

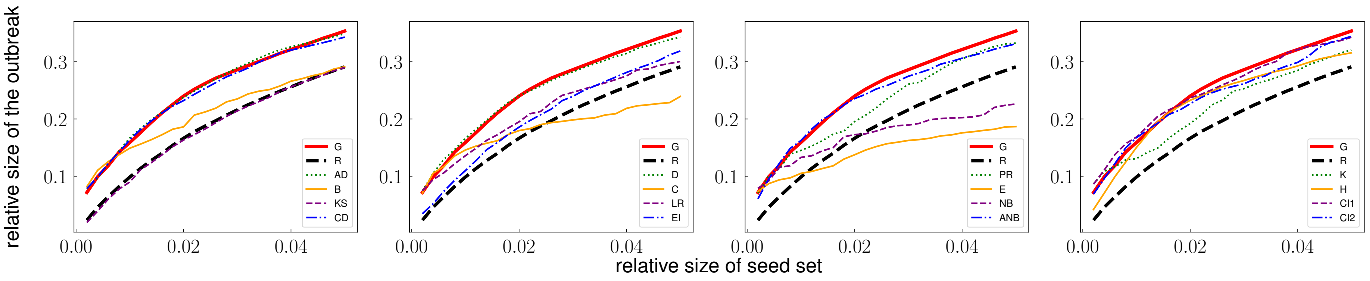

Figure 416: S838 - p=1.0p_c

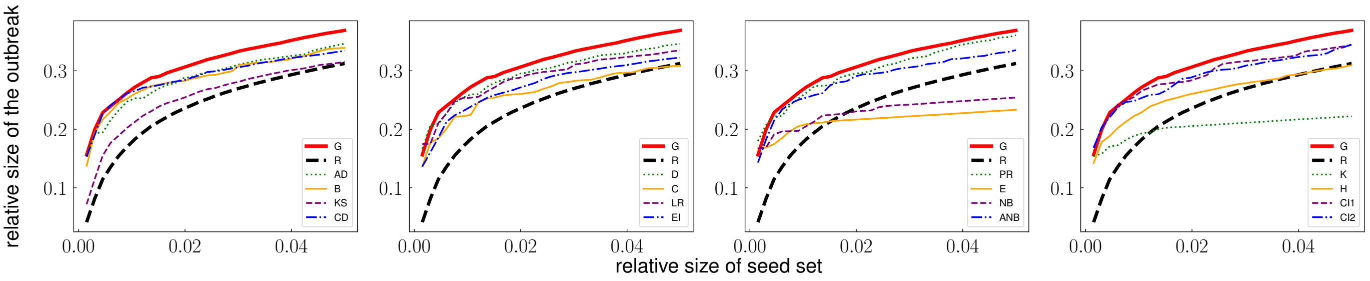

Figure 417: Yeast, transcription - p=1.0p_c

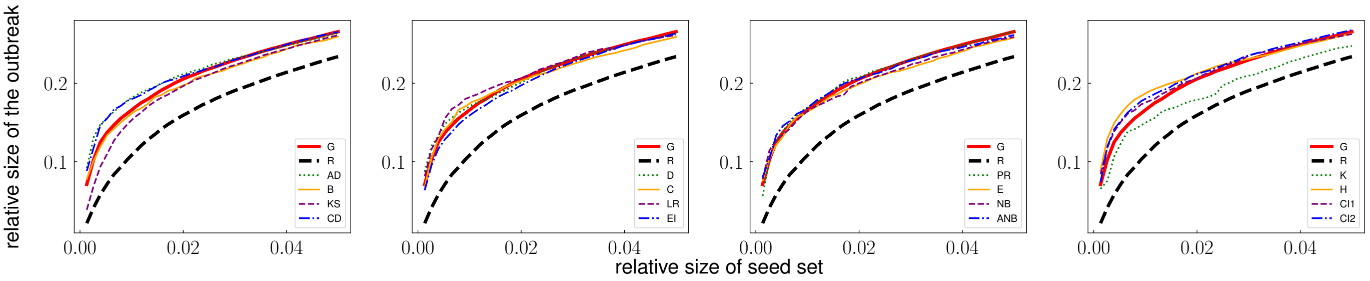

Figure 418: Caltech - p=1.0p_c

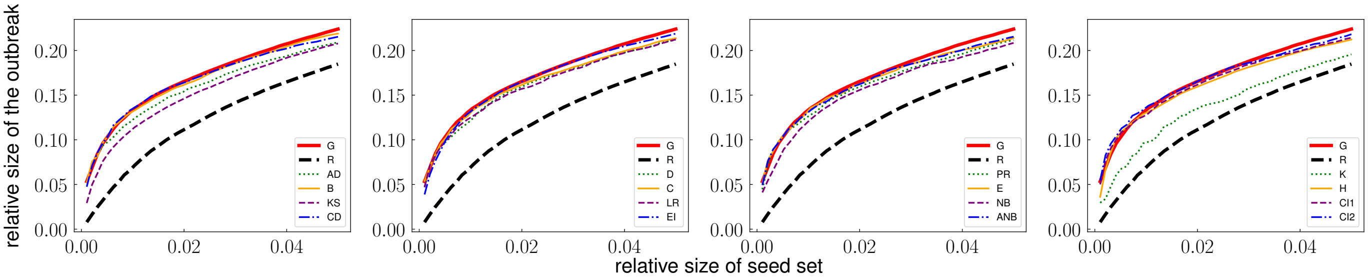

Figure 419: Reed - p=1.0p_c

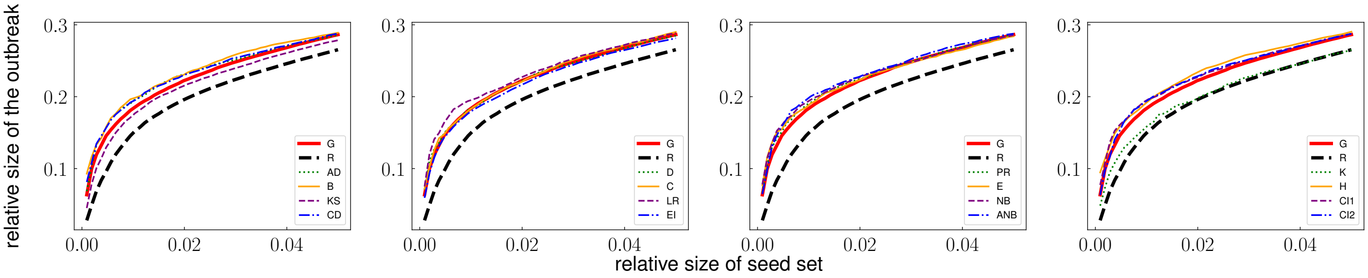

Figure 420: Mouse retina - p=1.0p_c

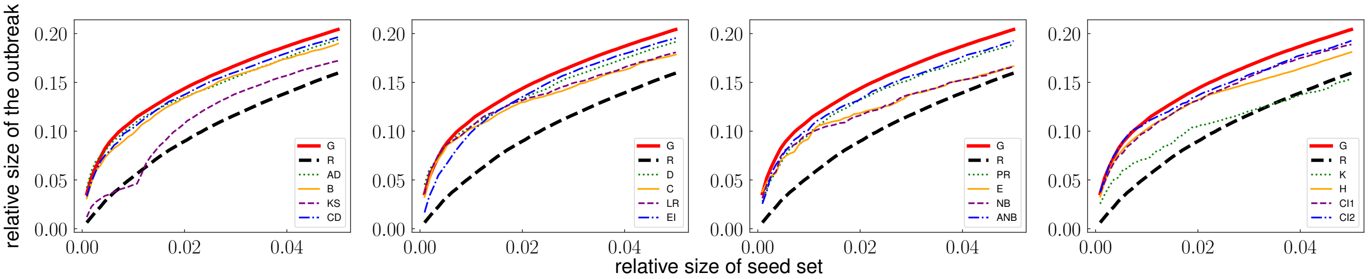

Figure 421: URV email - p=1.0p_c

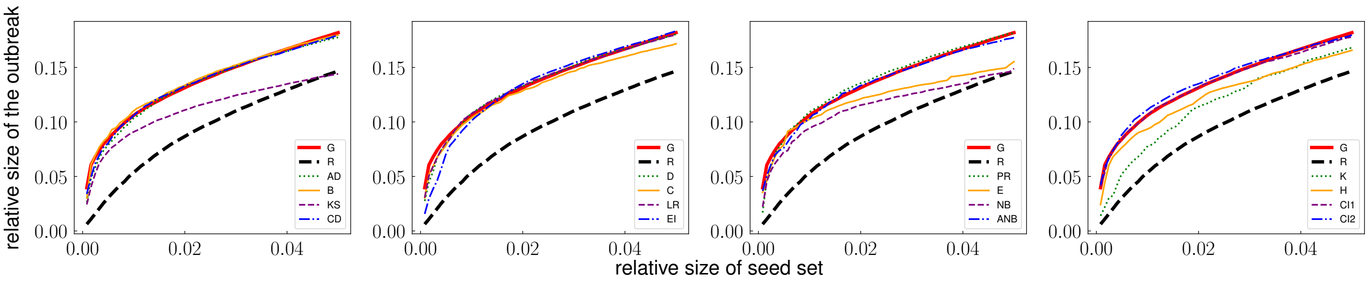

Figure 422: Political blogs - p=1.0p$_c$

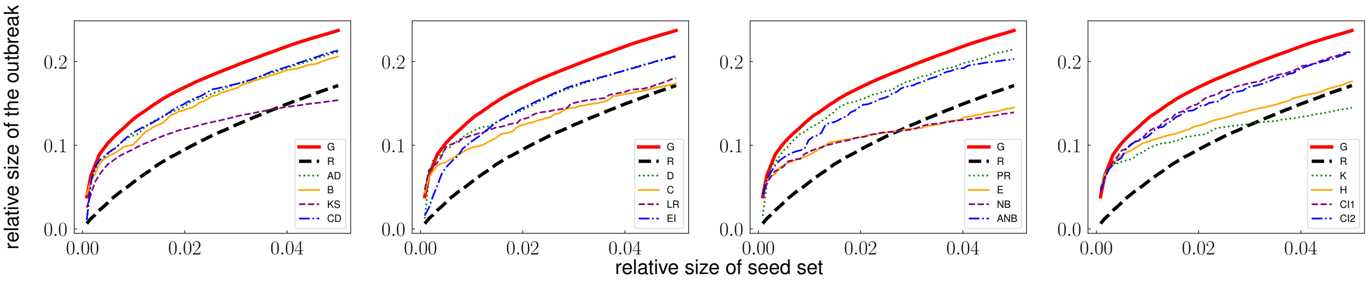

Figure 423: Air traffic - p=1.0p$_c$

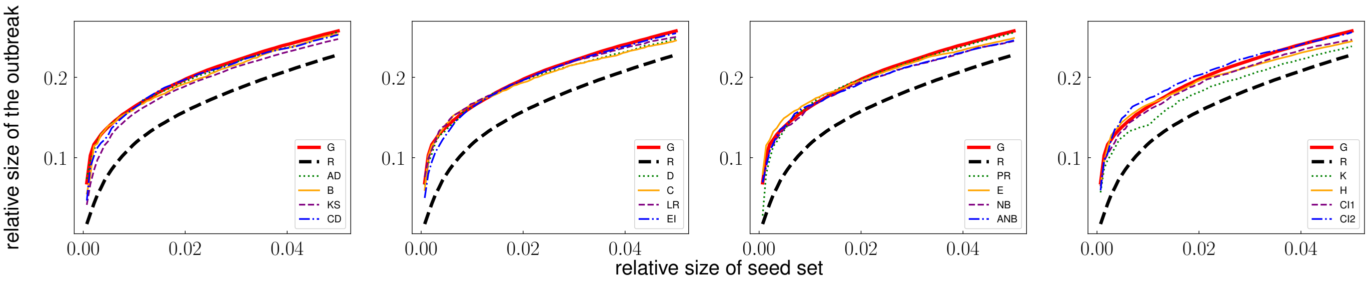

Figure 424: Haverford - p=1.0p$_c$

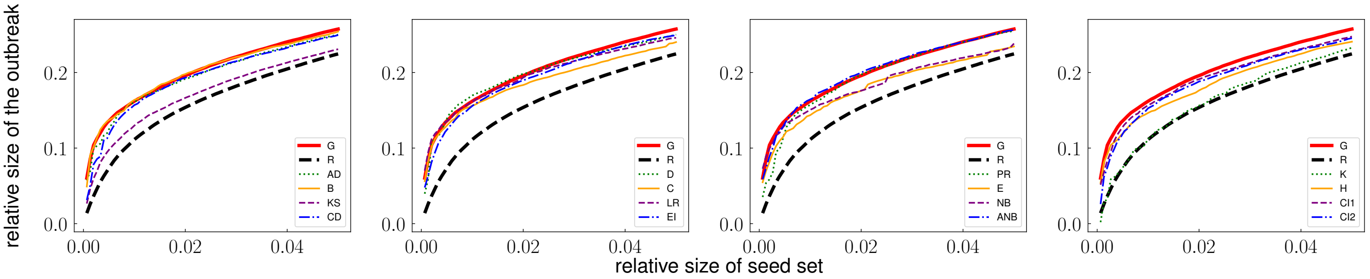

Figure 425: Simmons - p=1.0p$_c$

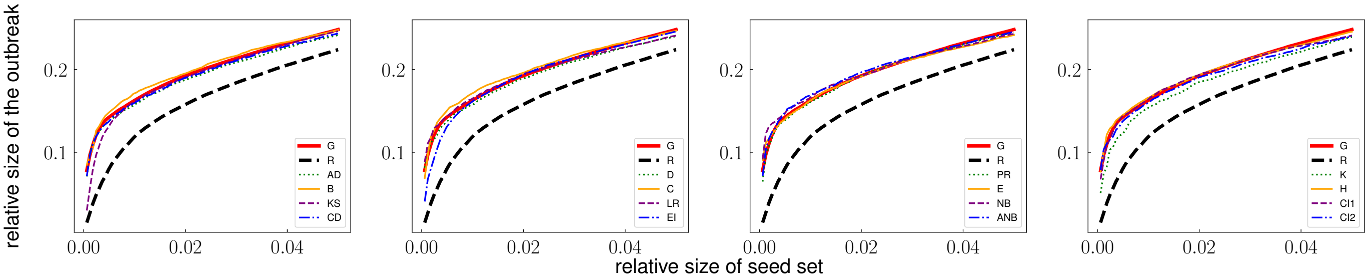

Figure 426: Swarthmore - p=1.0p$_c$

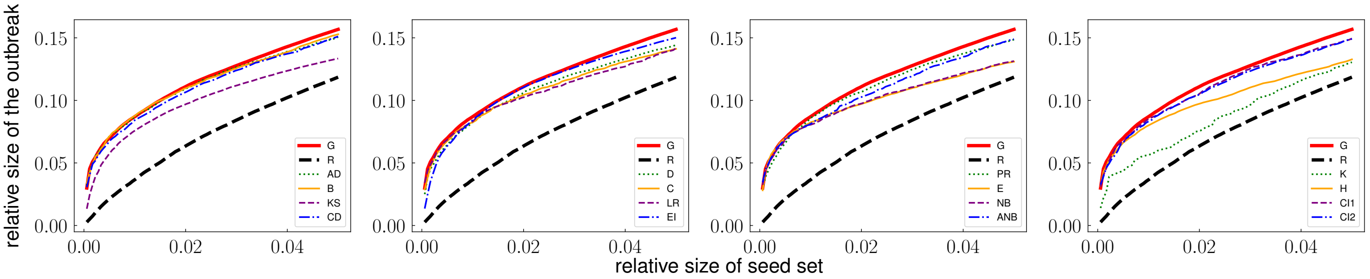

Figure 427: Petster, hamster - p=1.0p$_c$

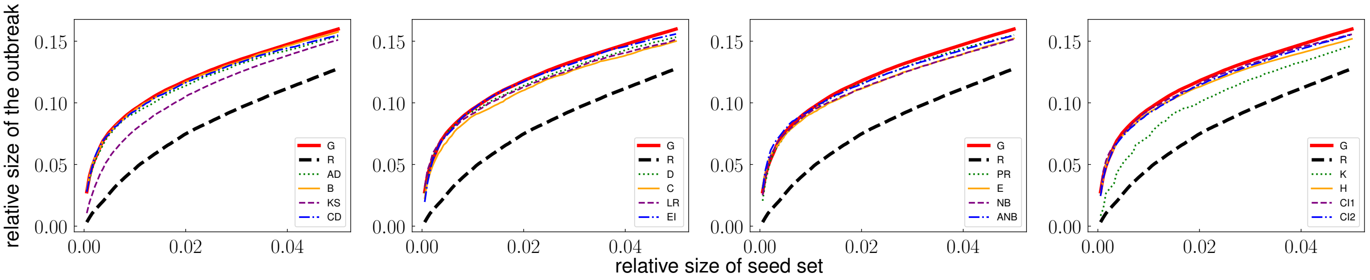

Figure 428: UC Irvine - p=1.0p_c

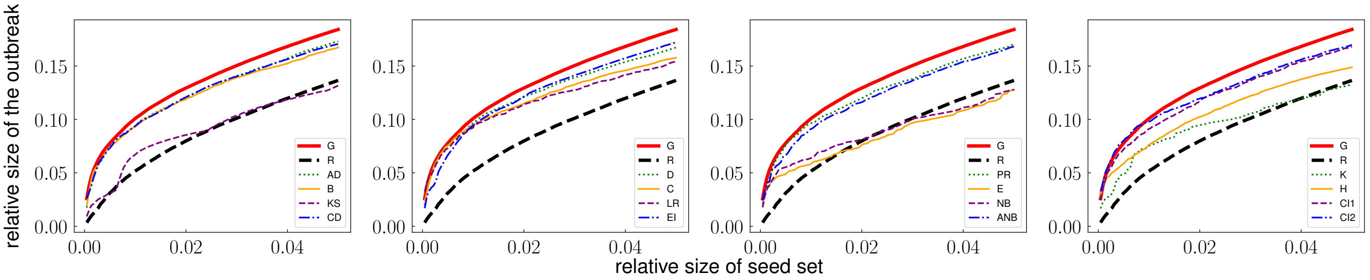

Figure 429: Yeast, protein - p=1.0p_c

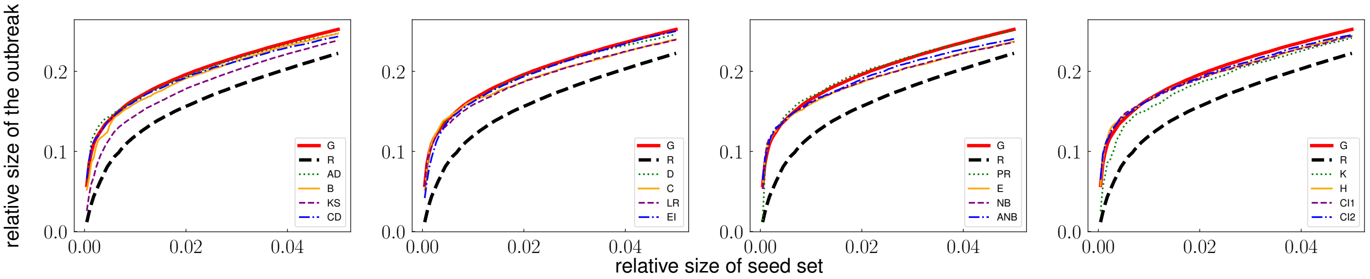

Figure 430: Amherst - p=1.0p_c

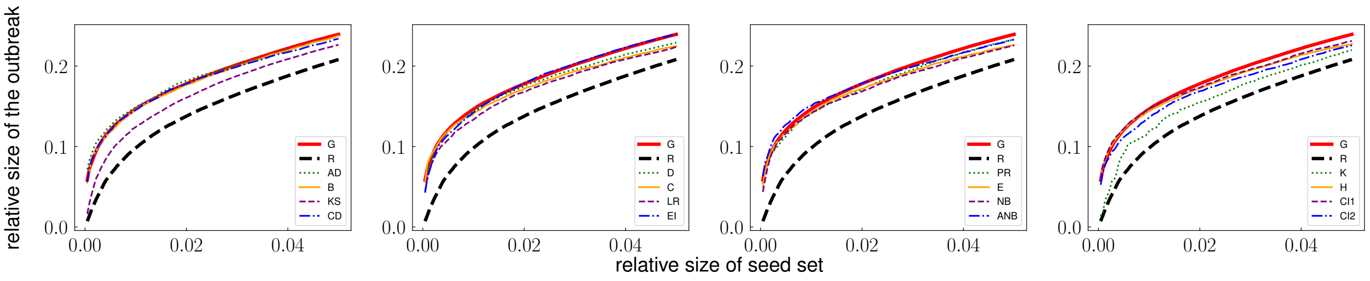

Figure 431: Bowdoin - p=1.0p_c

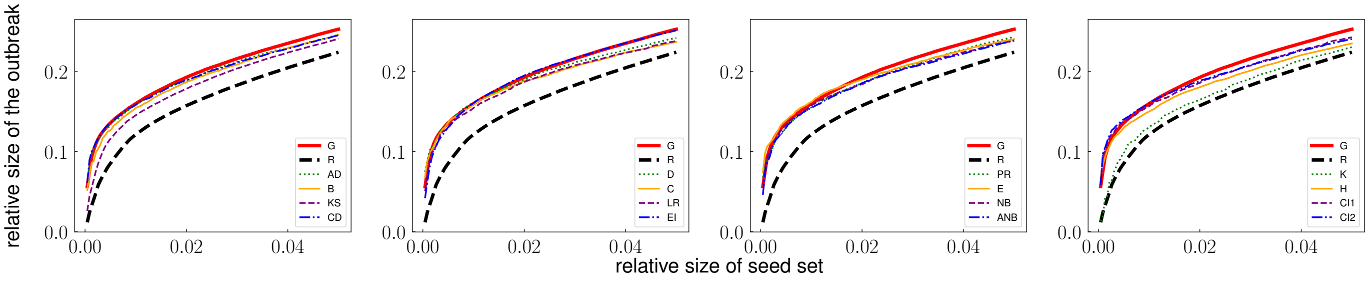

Figure 432: Hamilton - p=1.0p_c

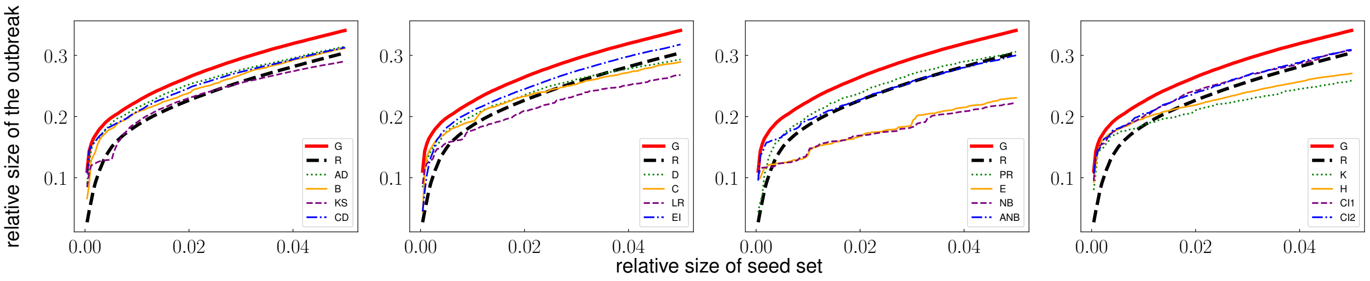

Figure 433: Adolescent health - p=1.0p_c

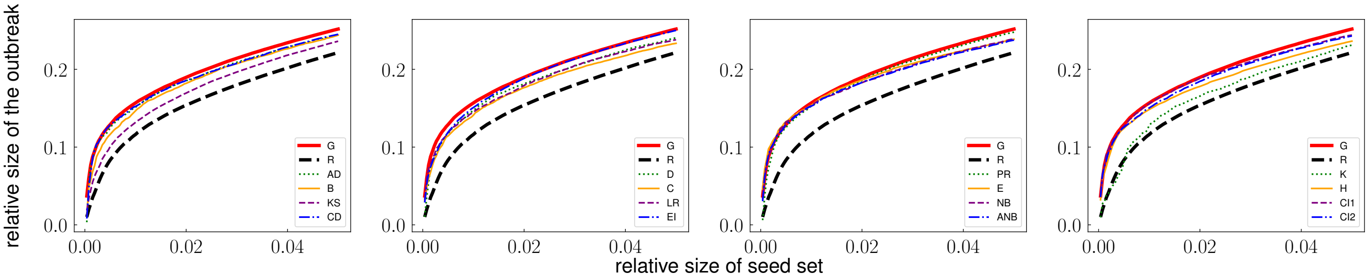

Figure 434: Trinity - p=1.0p_c

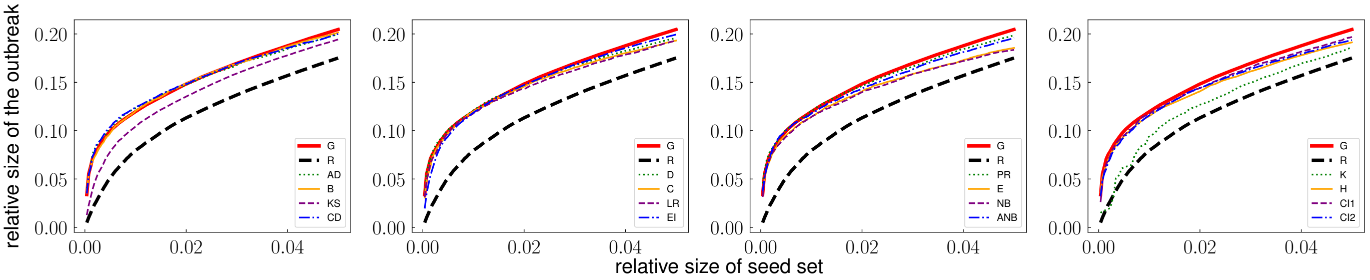

Figure 435: USFCA - p=1.0p_c

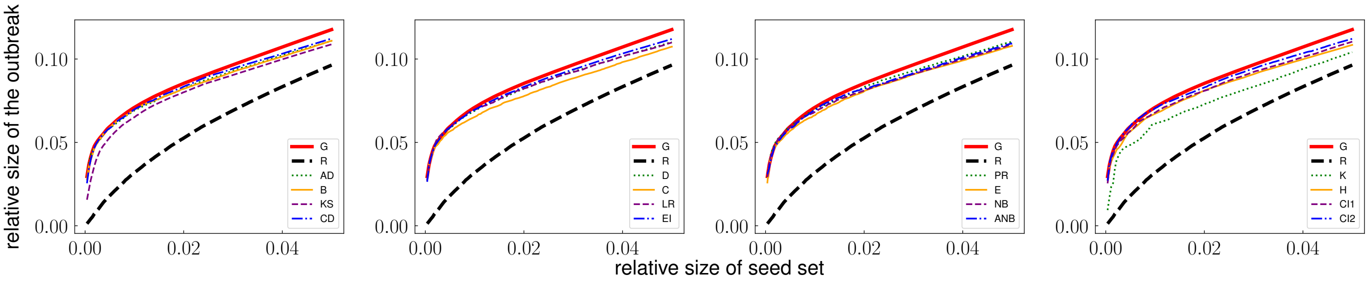

Figure 436: Japanese - p=1.0p_c

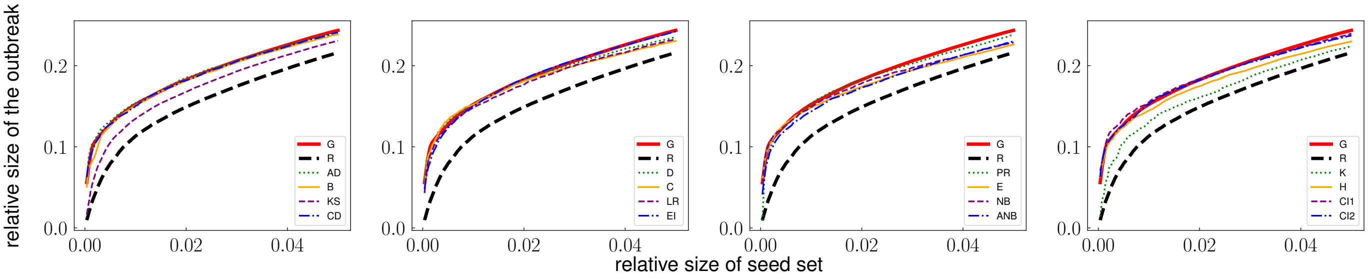

Figure 437: Williams - p=1.0p_c

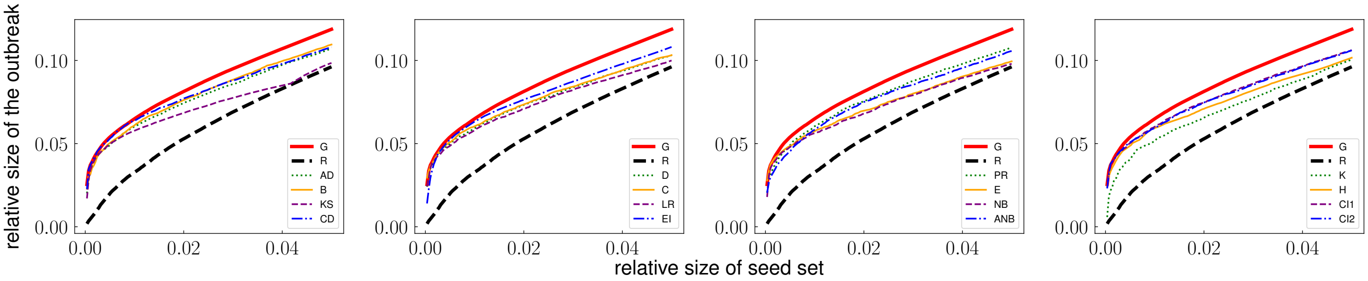

Figure 438: Open flights - p=1.0p_c

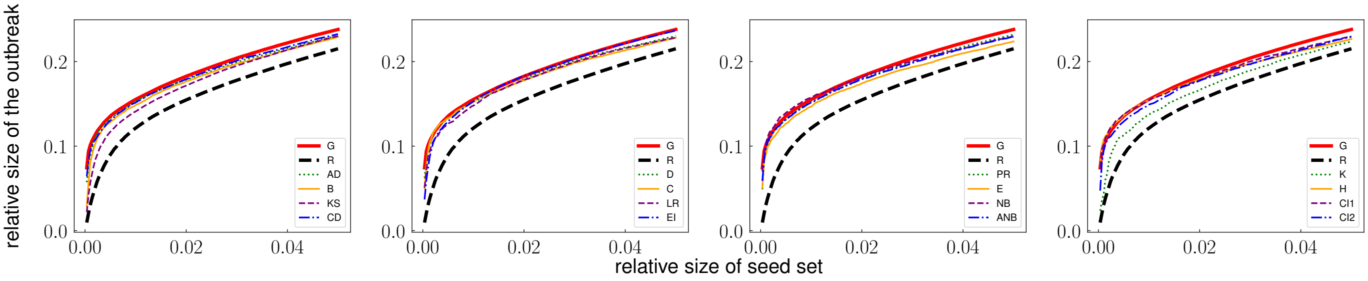

Figure 439: Oberlin - p=1.0p_c

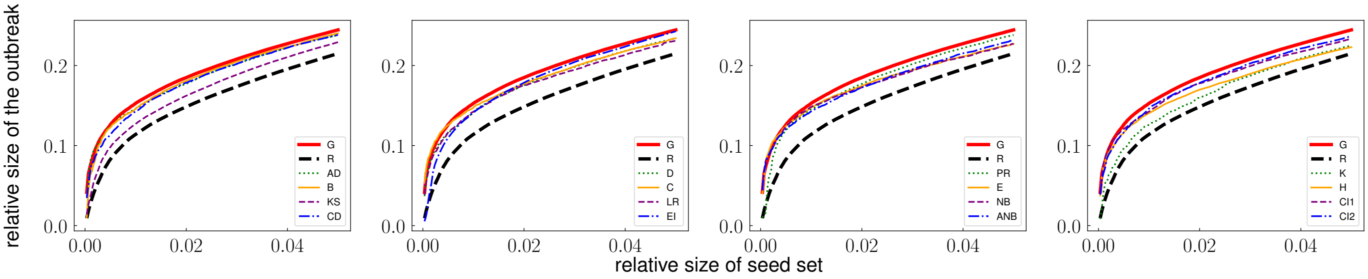

Figure 440: Smith - p=1.0p_c

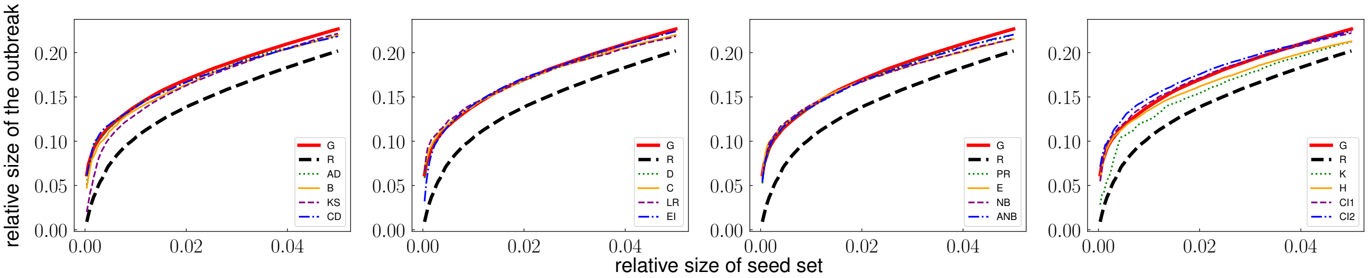

Figure 441: Wellesley - p=1.0p_c

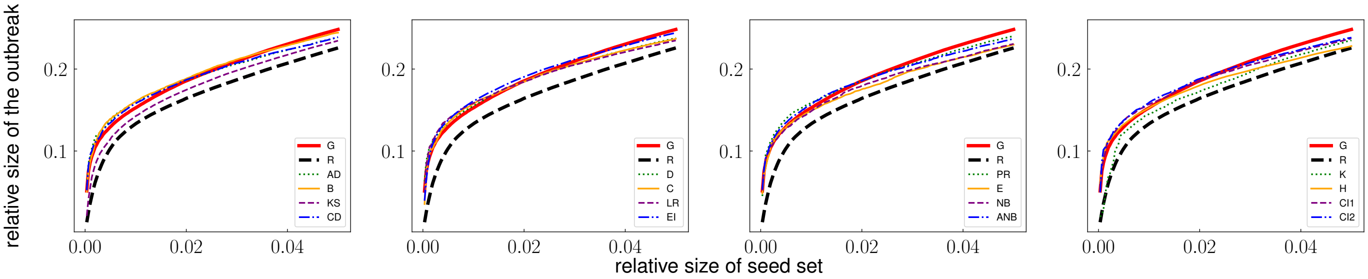

Figure 442: Vassar - p=1.0p_c

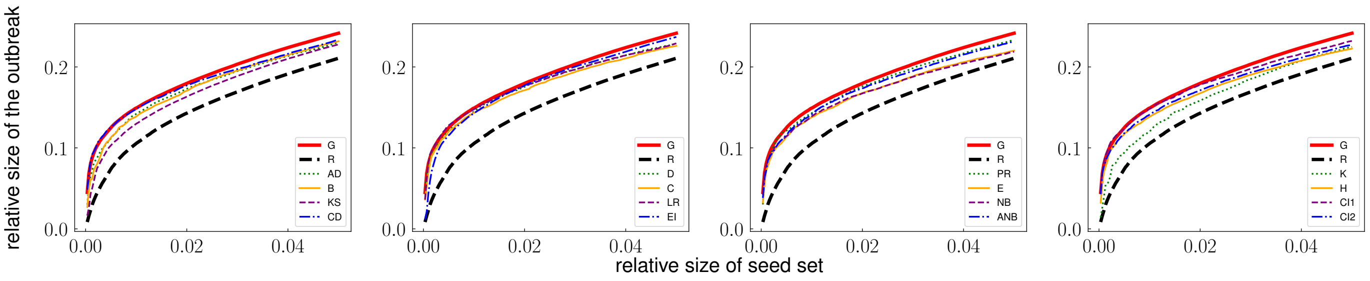

Figure 443: Middlebury - p=1.0p_c

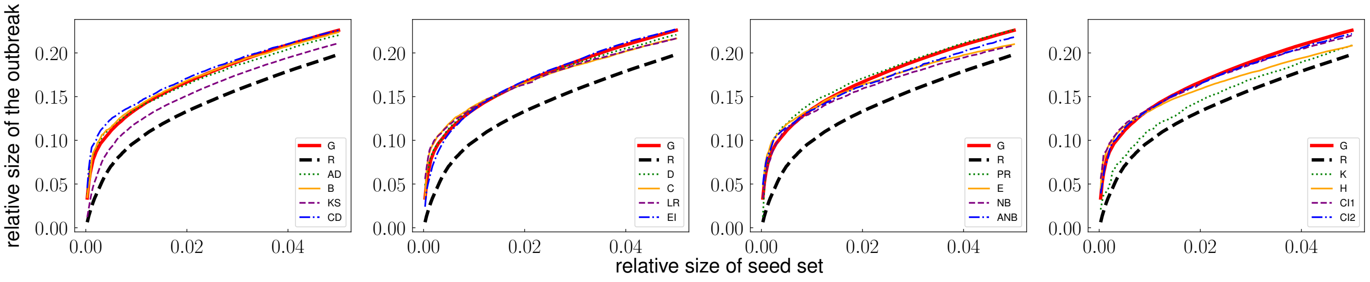

Figure 444: Pepperdine - p=1.0p_c

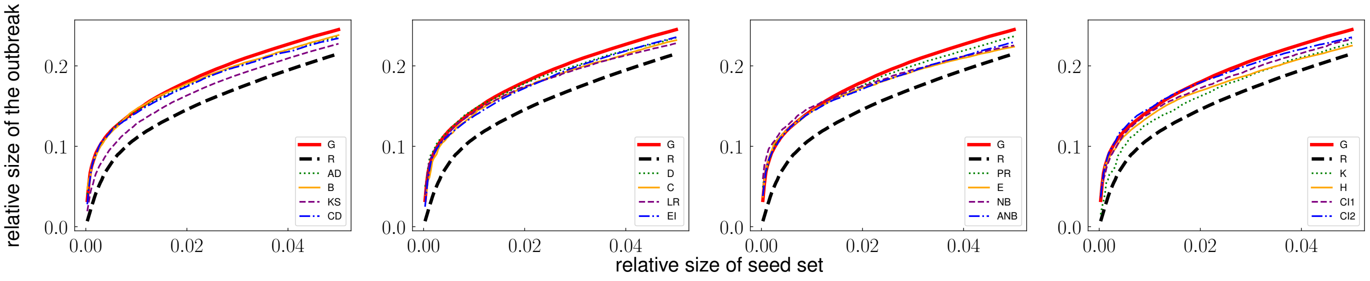

Figure 445: Colgate - p=1.0p_c

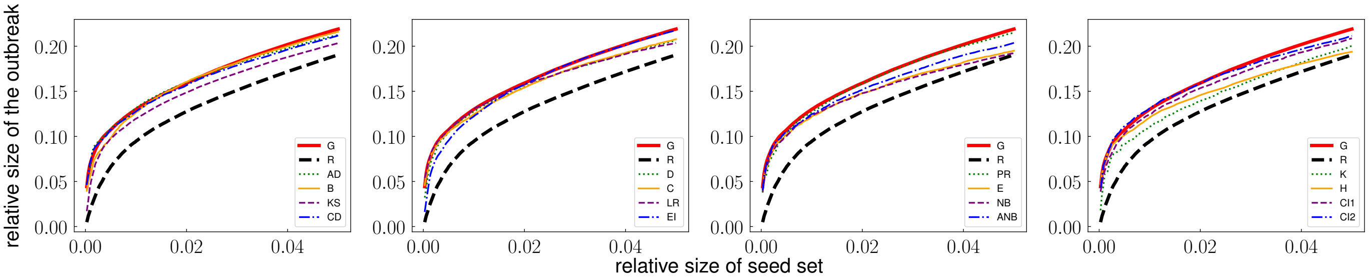

Figure 446: Santa - p=1.0p_c

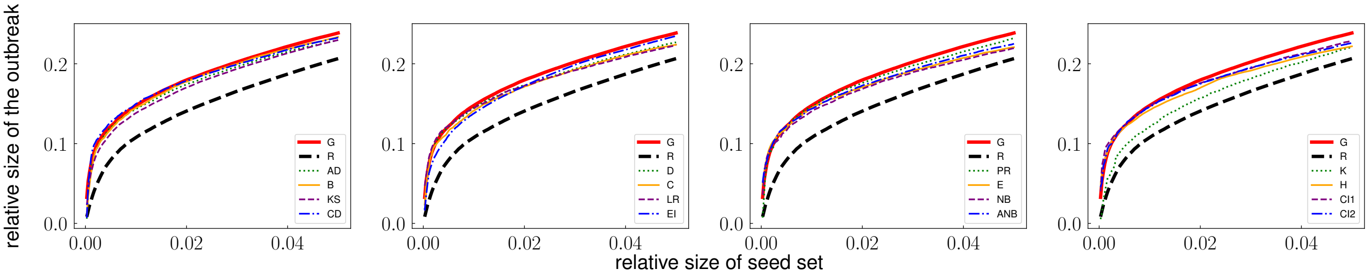

Figure 447: Wesleyan - p=1.0p_c

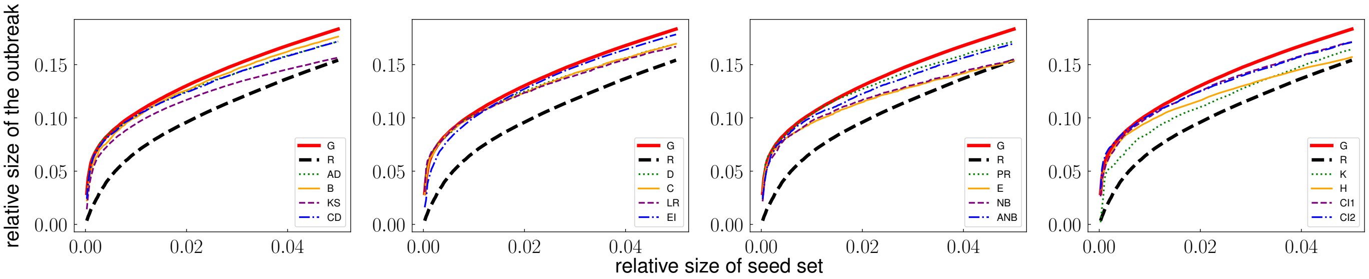

Figure 448: Mich - p=1.0p_c

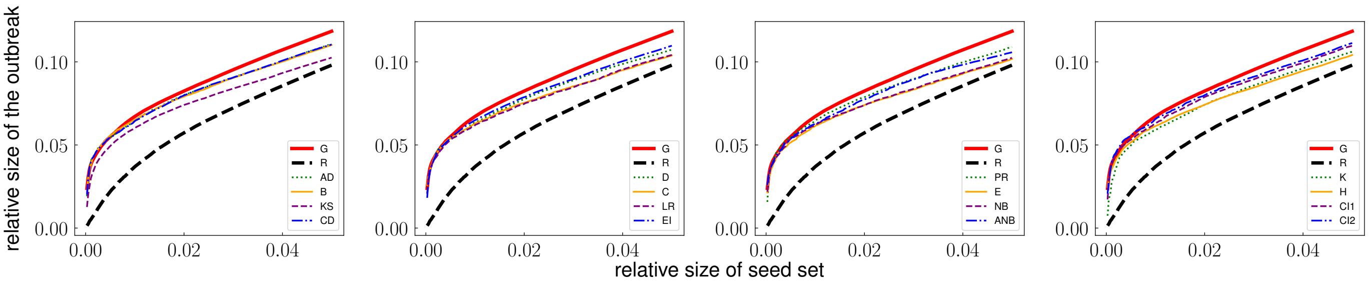

Figure 449: Bitcoin Alpha - p=1.0p_c

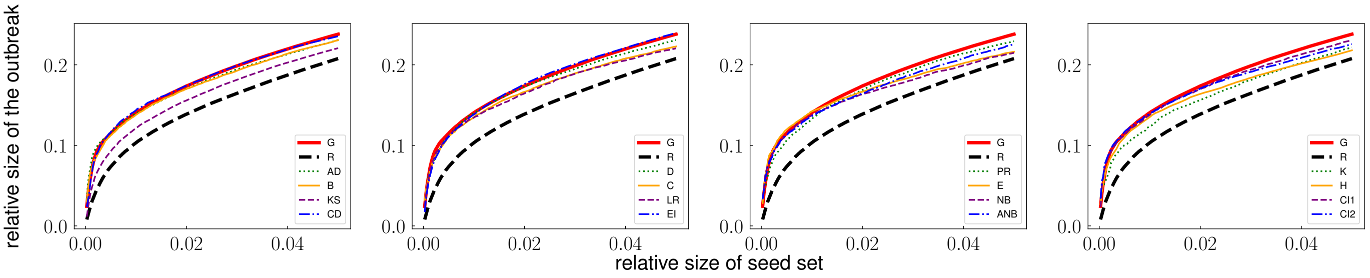

Figure 450: Bucknell - p=1.0p_c

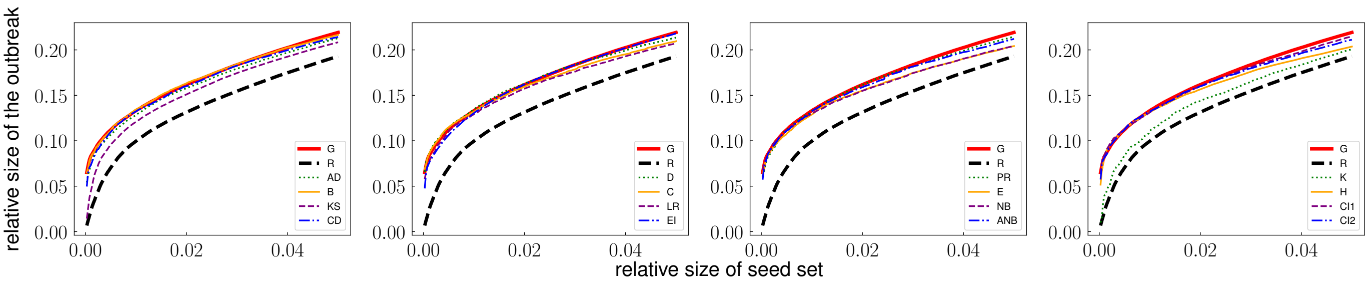

Figure 451: Brandeis - p=1.0p_c

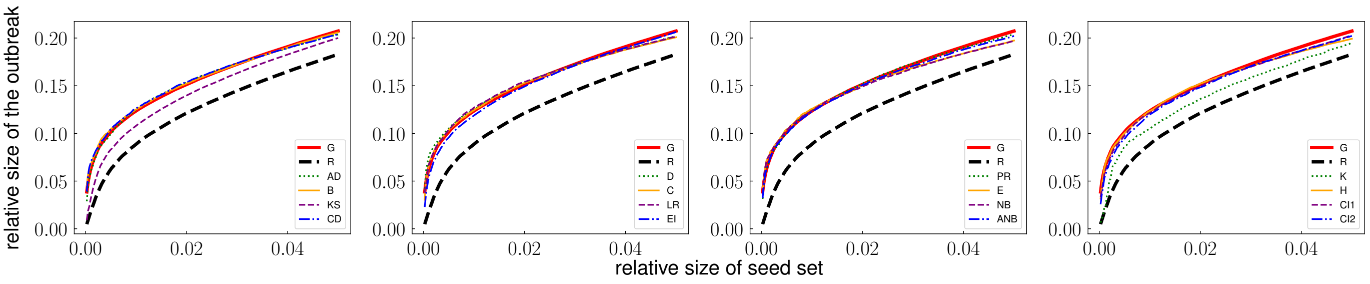

Figure 452: Howard - p=1.0p$_c$

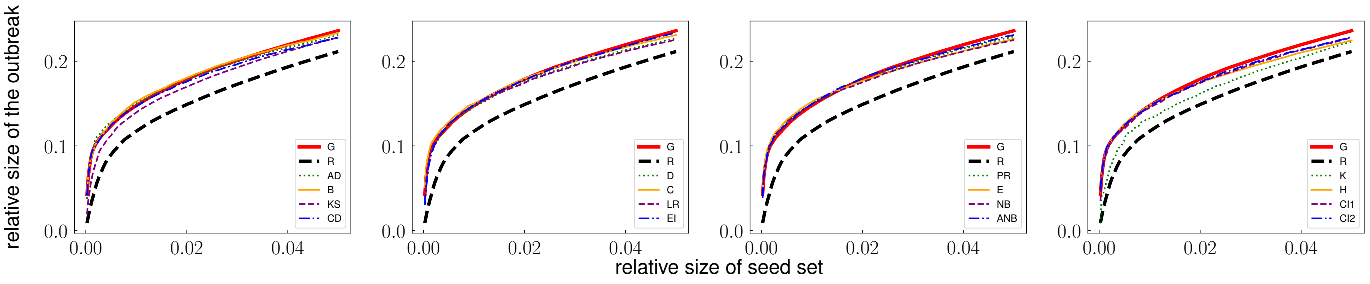

Figure 453: Rice - p=1.0p$_c$

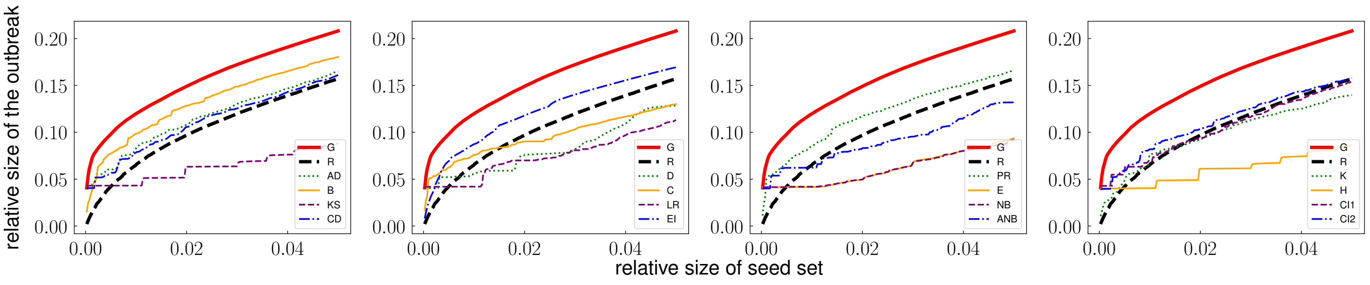

Figure 454: GR-QC, 1993-2003 - p=1.0p$_c$

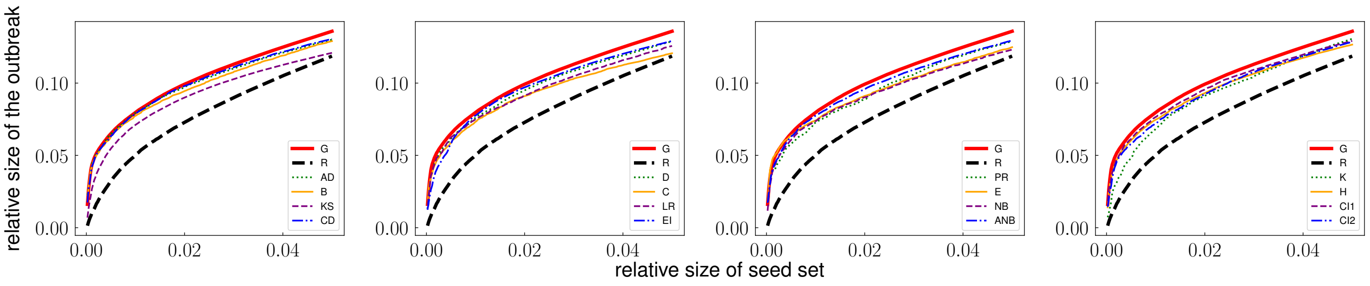

Figure 455: Tennis - p=1.0p_c

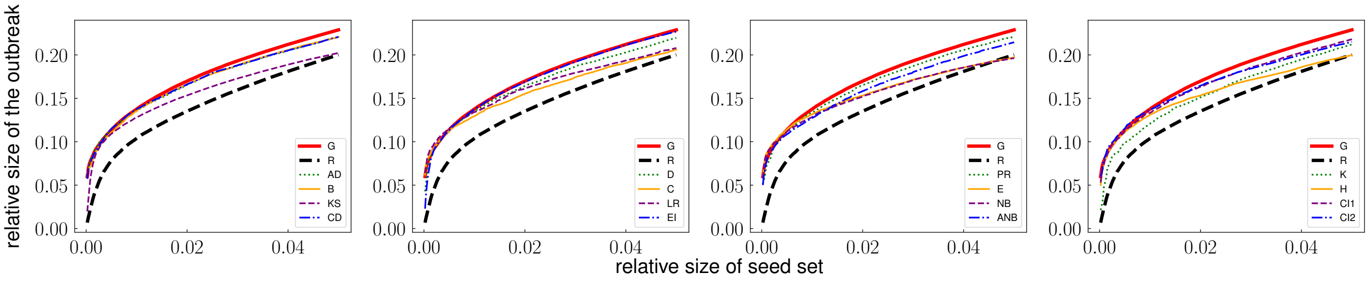

Figure 456: Rochester - p=1.0p_c

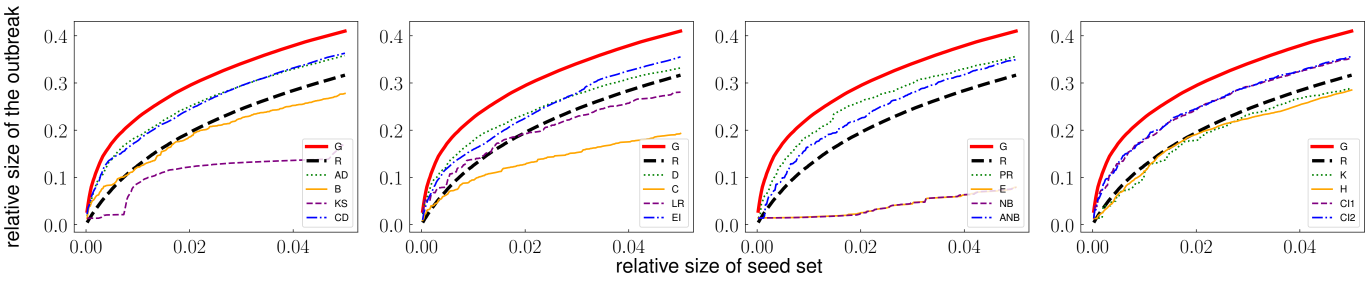

Figure 457: US Power grid - p=1.0p_c

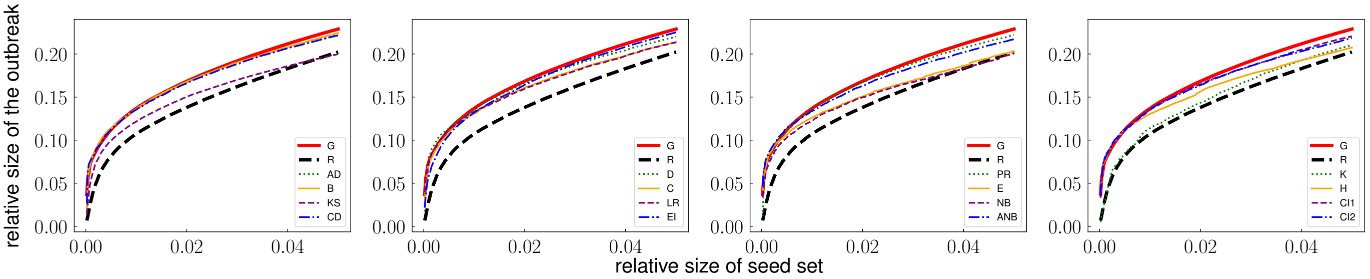

Figure 458: Lehigh - p=1.0p_c

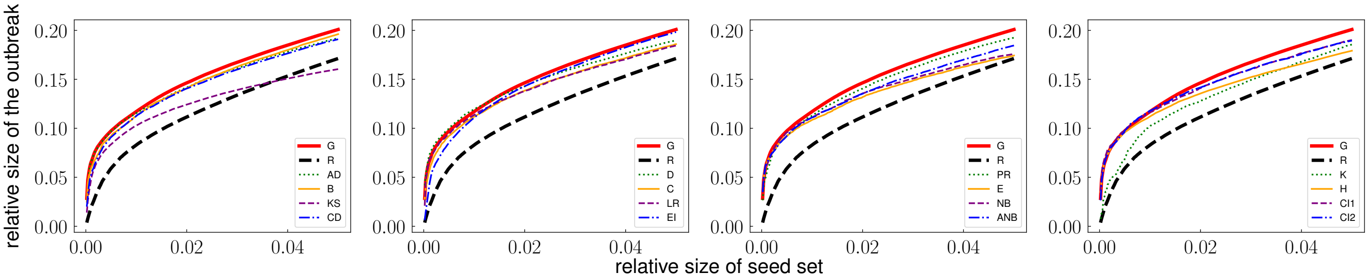

Figure 459: Johns Hopkins - p=1.0p_c

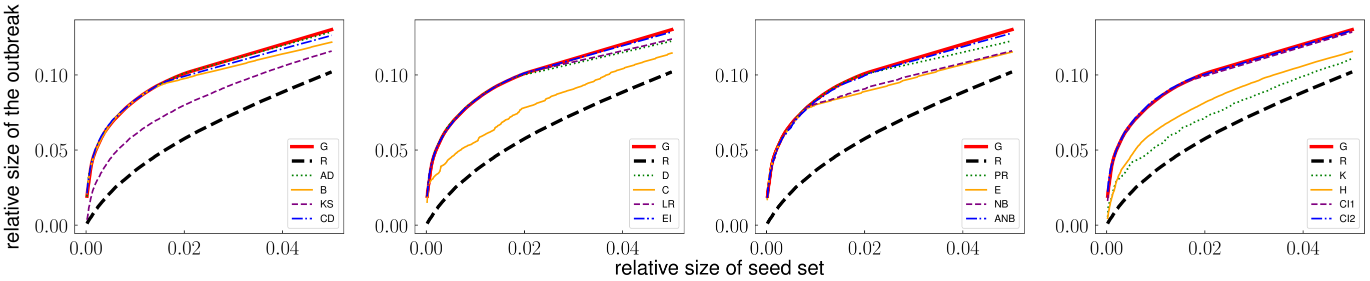

Figure 460: HT09 - p=1.0p_c

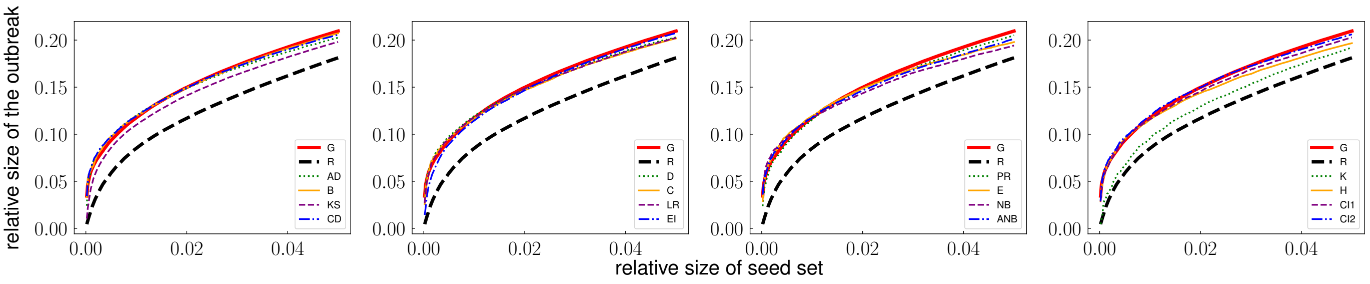

Figure 461: Wake - p=1.0p_c

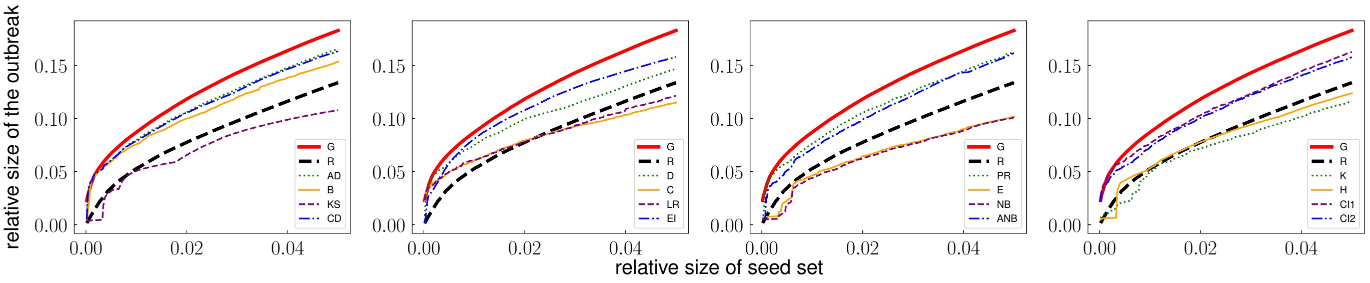

Figure 462: Hep-Th, 1995-1999 - p=1.0p_c

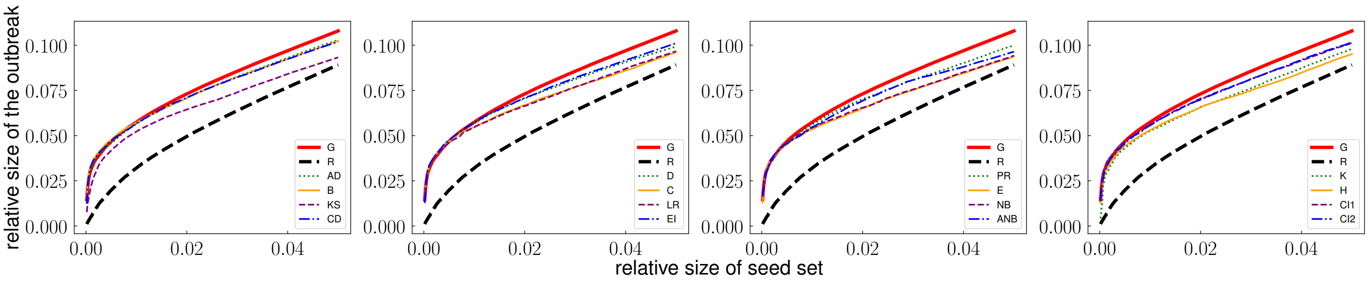

Figure 463: Bitcoin OTC - p=1.0p_c

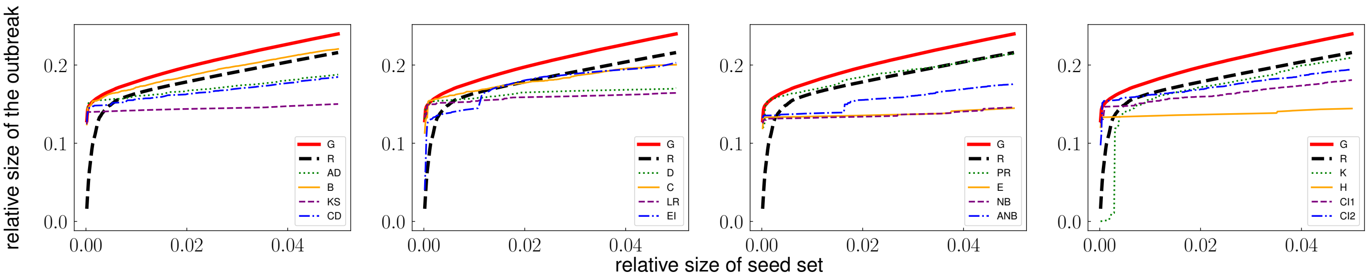

Figure 464: Reactome - p=1.0p_c

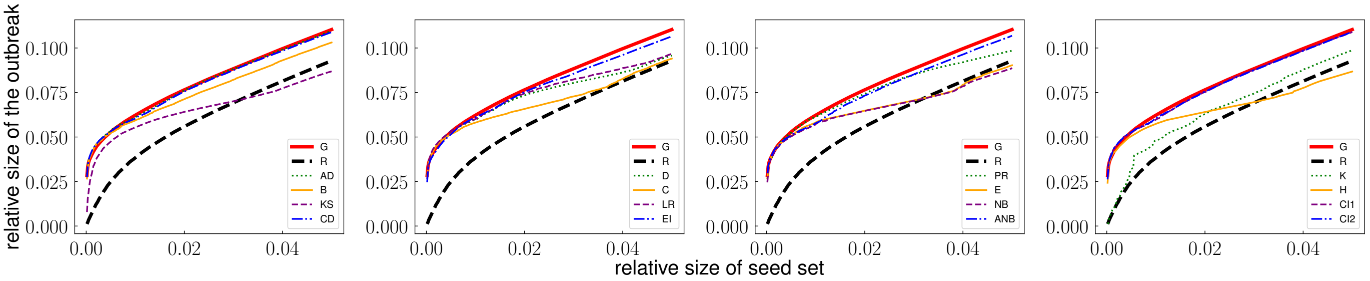

Figure 465: Jung - p=1.0p_c

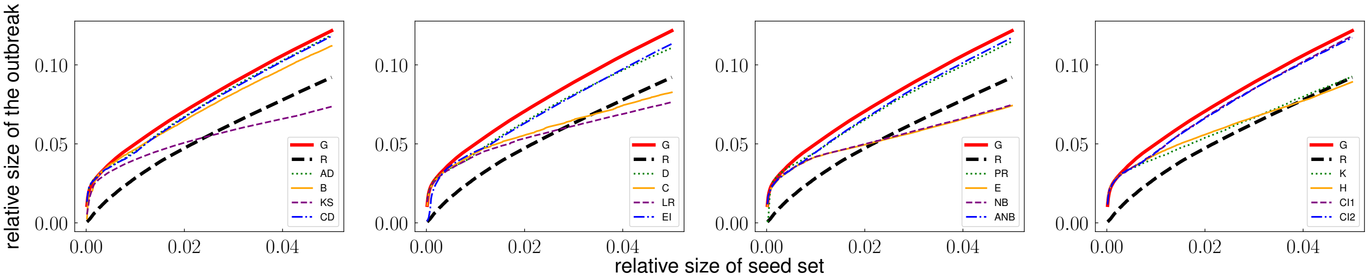

Figure 466: Gnutella, Aug. 8, 2002 - p=1.0p_c

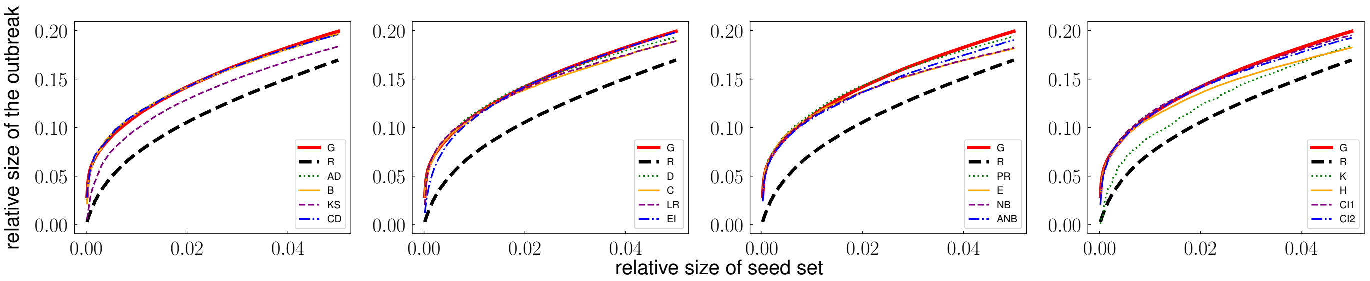

Figure 467: American - p=1.0p_c

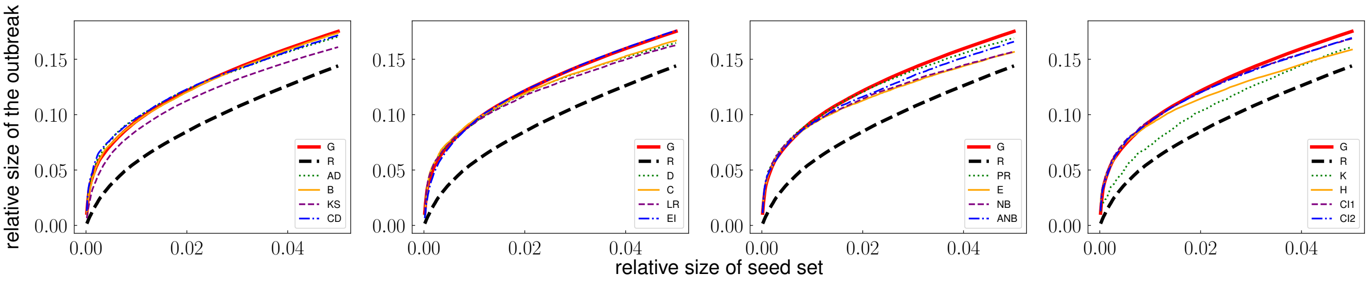

Figure 468: MIT - p=1.0p_c

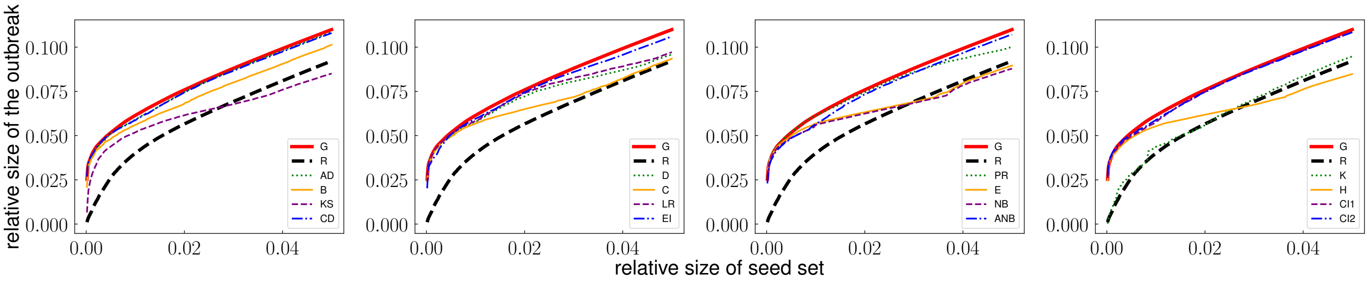

Figure 469: JDK - p=1.0p_c

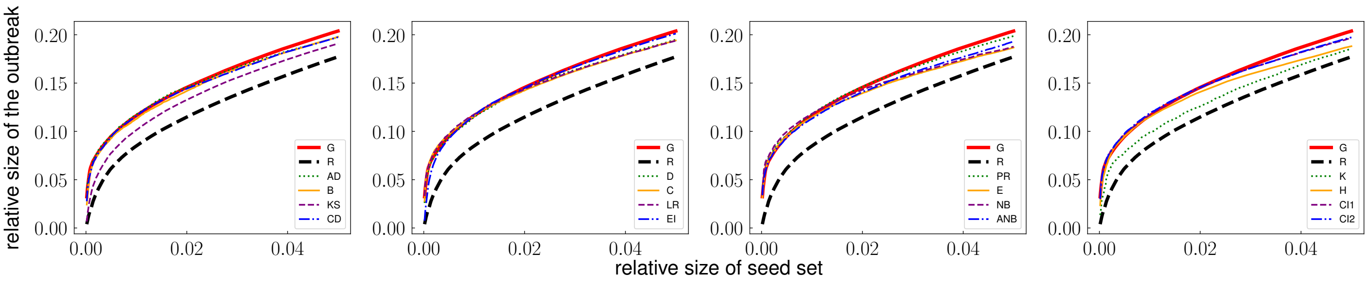

Figure 470: William - p=1.0p$_c$

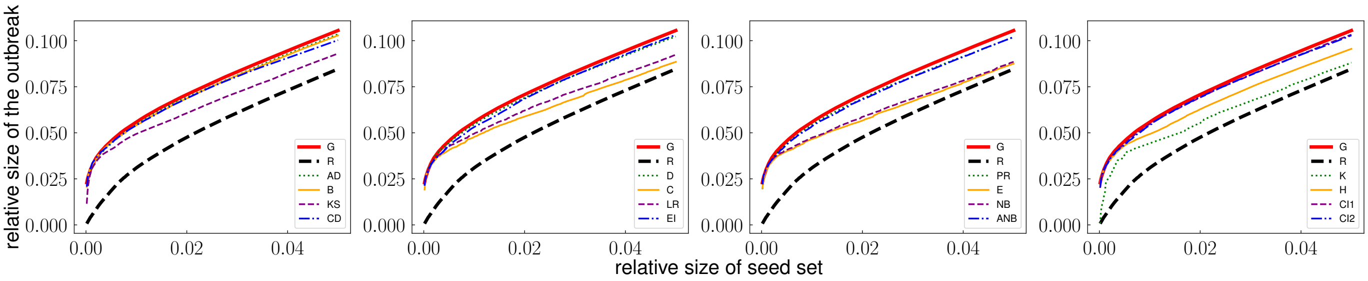

Figure 471: AS Oregon - p=1.0p$_c$

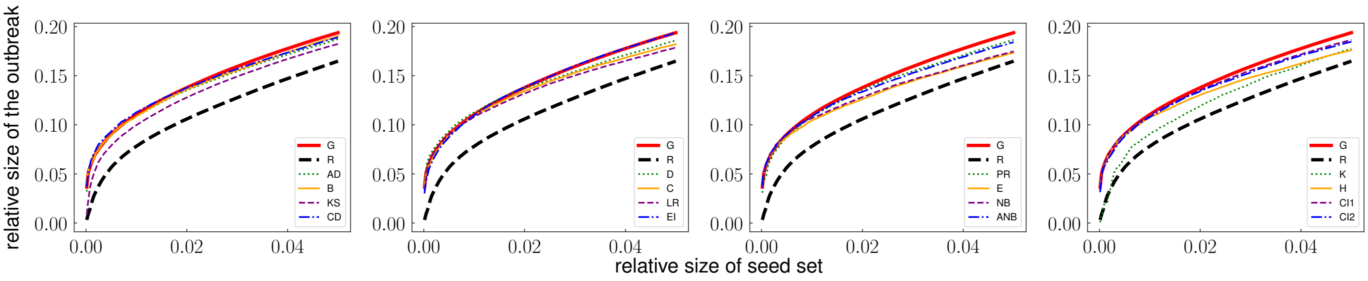

Figure 472: UChicago - p=1.0p$_c$

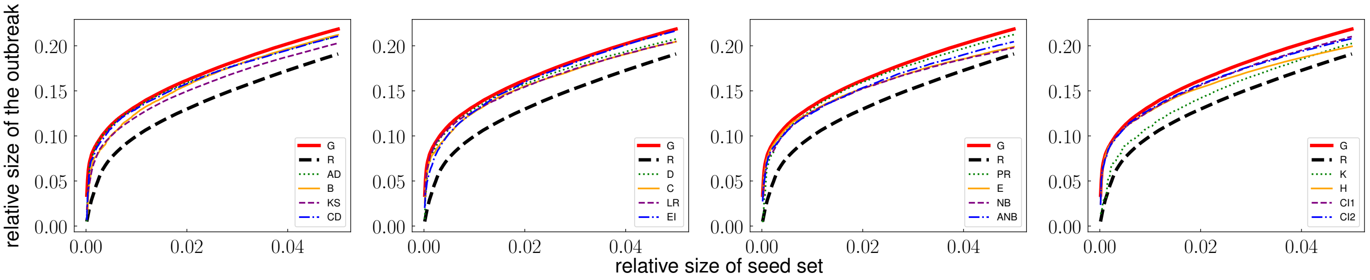

Figure 473: Princeton - $p=1.0p_c$

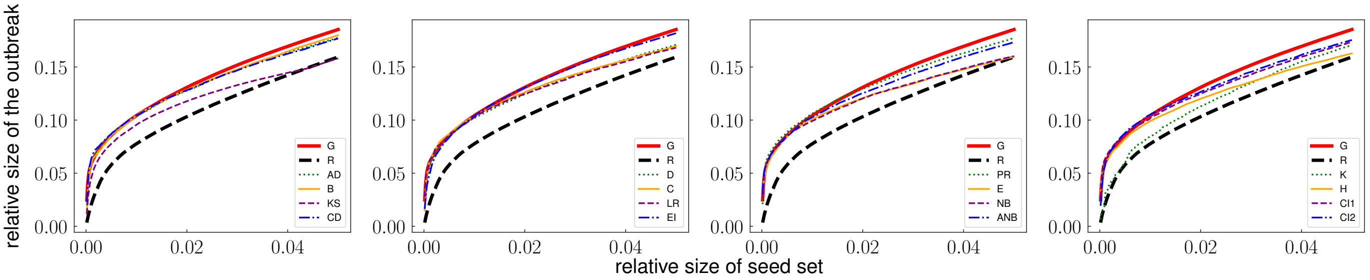

Figure 474: Carnegie - $p=1.0p_c$

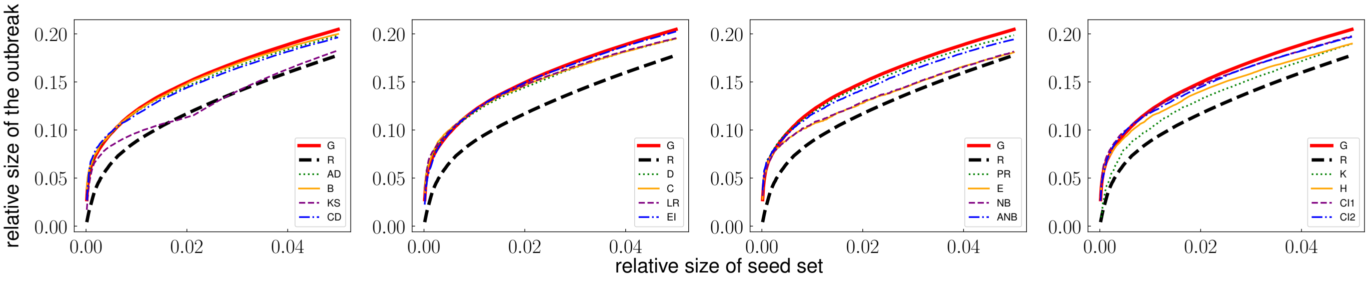

Figure 475: Tufts - $p=1.0p_c$

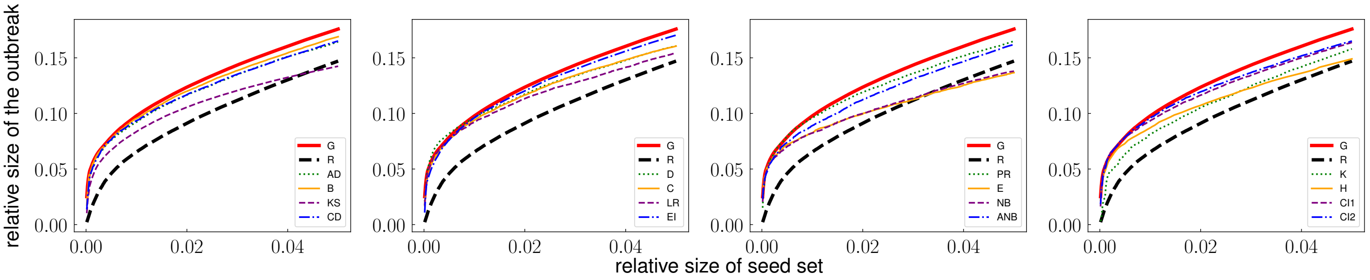

Figure 476: UC - p=1.0p_c

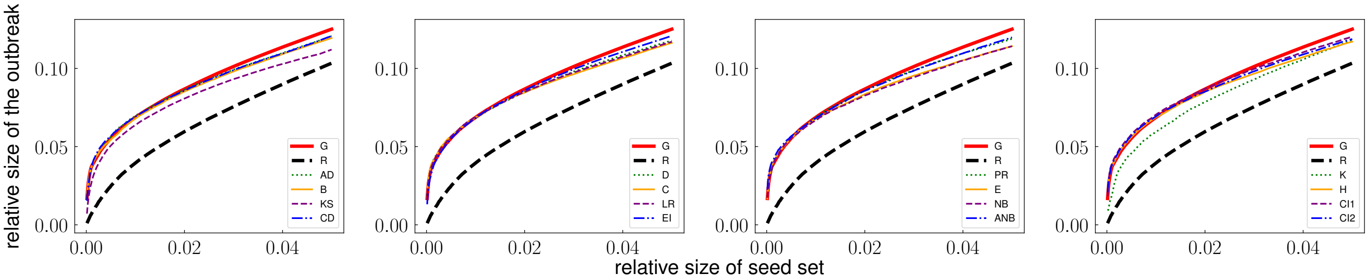

Figure 477: Wikipedia elections - p=1.0p_c

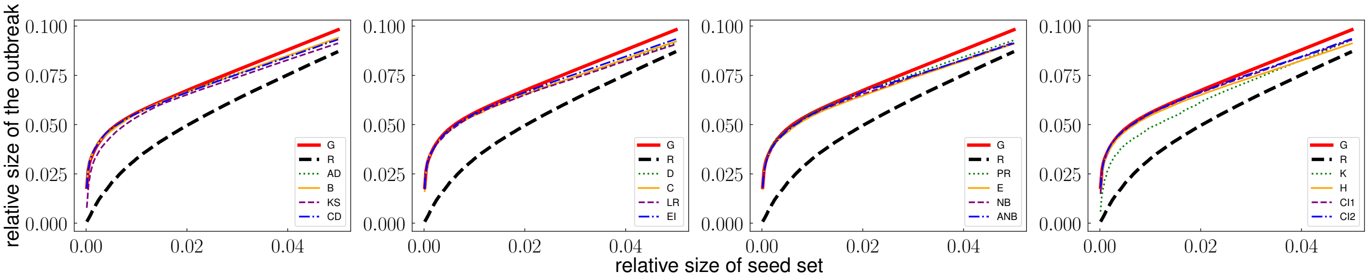

Figure 478: English - p=1.0p_c

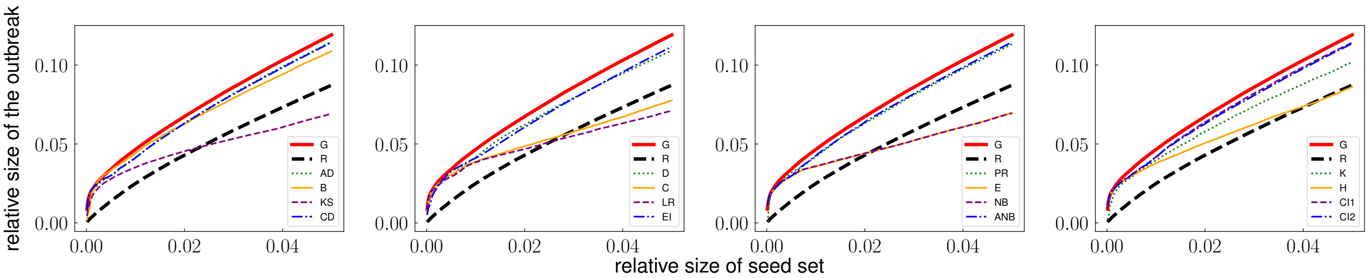

Figure 479: Gnutella, Aug. 9, 2002 - p=1.0p_c

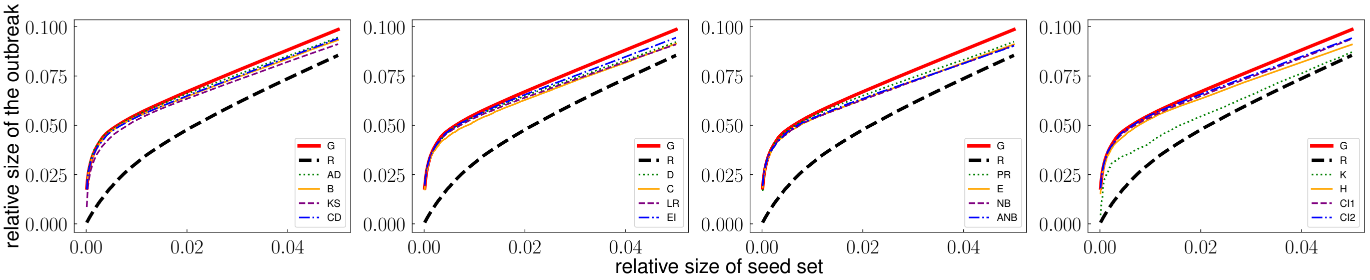

Figure 480: French - p=1.0p_c

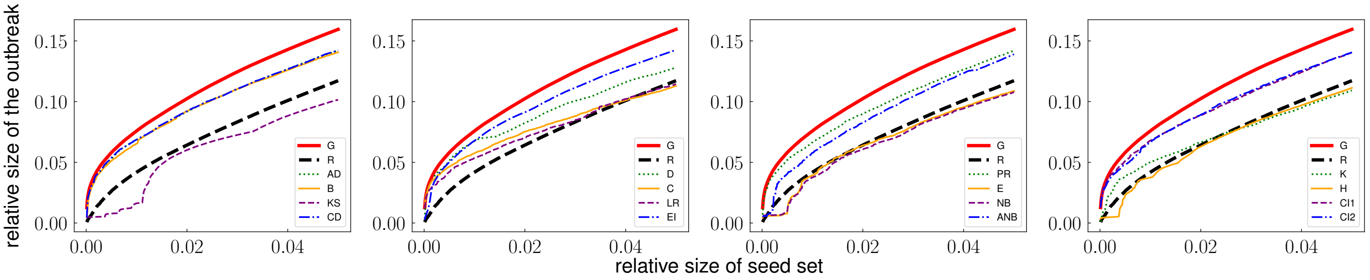

Figure 481: Hep-Th, 1993-2003 - p=1.0p_c

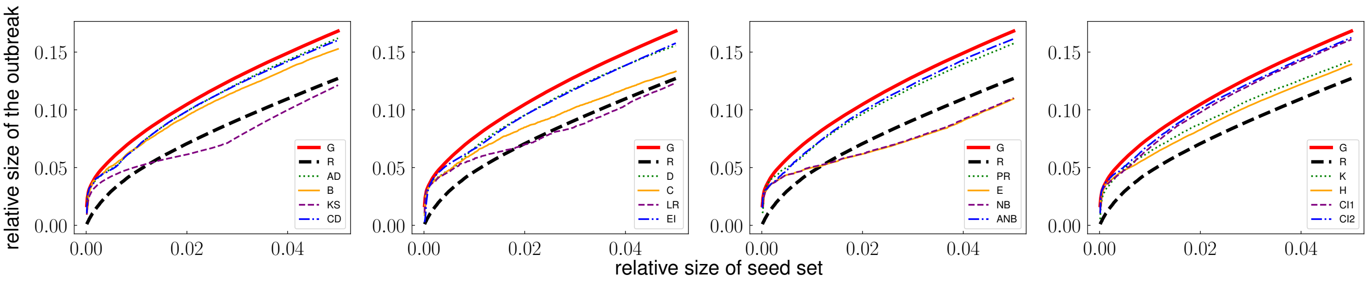

Figure 482: Gnutella, Aug. 6, 2002 - p=1.0p$_c$

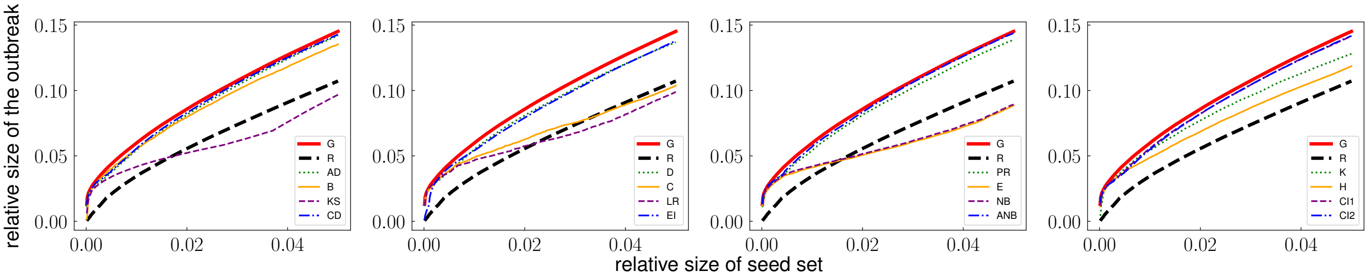

Figure 483: Gnutella, Aug. 5, 2002 - p=1.0p$_c$

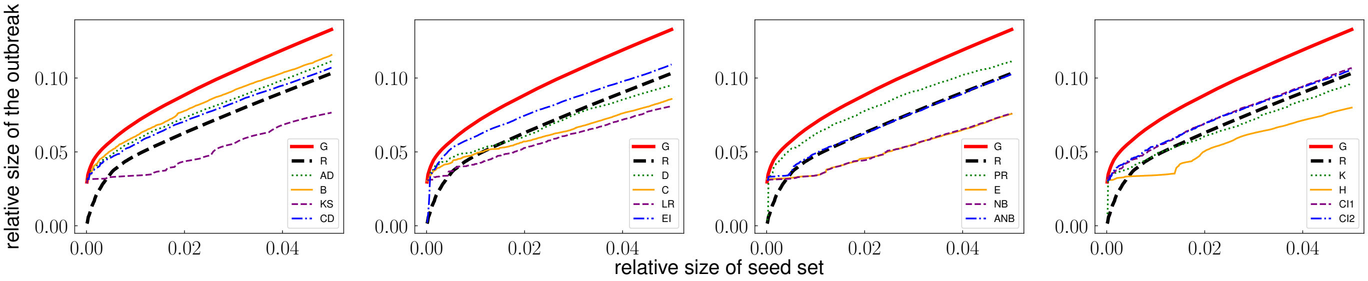

Figure 484: PGP - p=1.0p$_c$

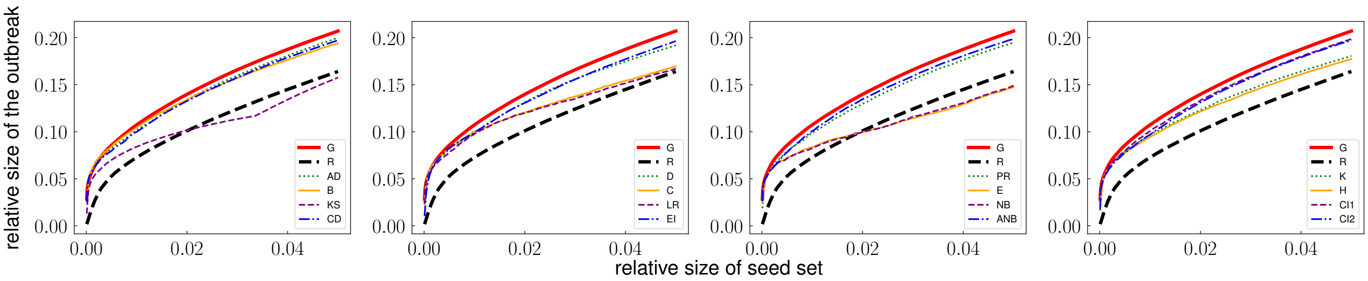

Figure 485: Gnutella, Aug. 4, 2002 - p=1.0p_c

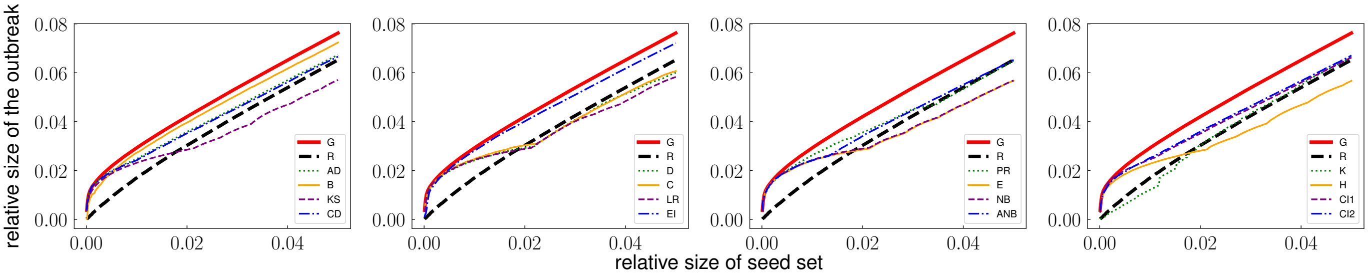

Figure 486: Hep-Ph, 1993-2003 - p=1.0p_c

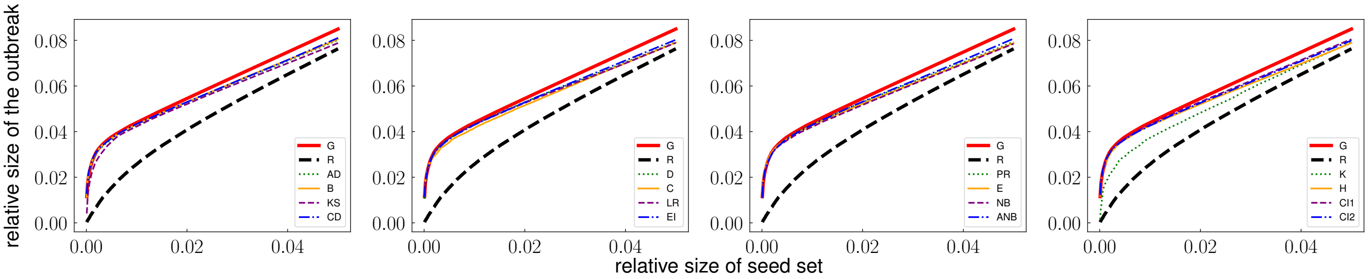

Figure 487: Spanish 1 - p=1.0p_c

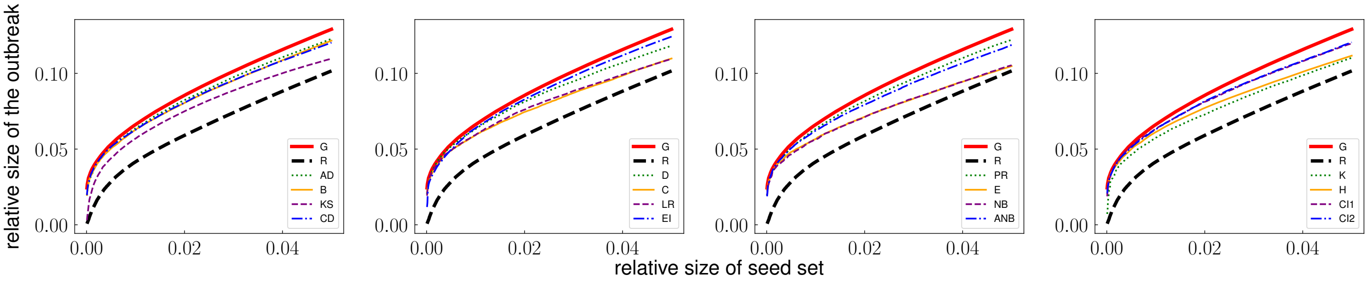

Figure 488: DBLP, citations - p=1.0p_c

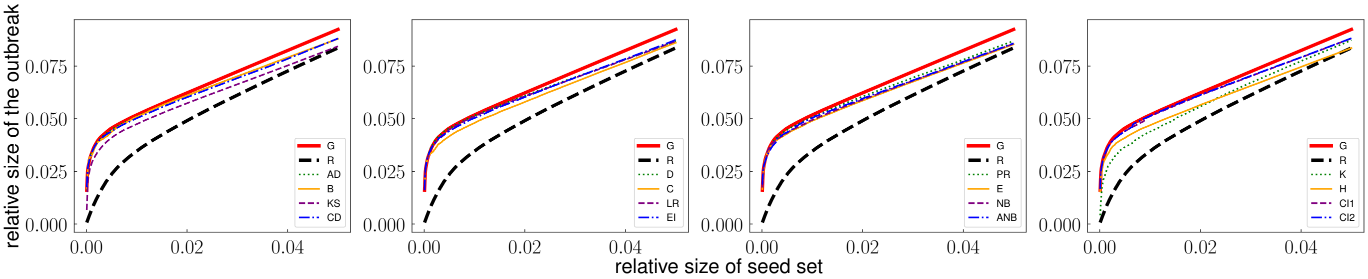

Figure 489: Spanish 2 - p=1.0p_c

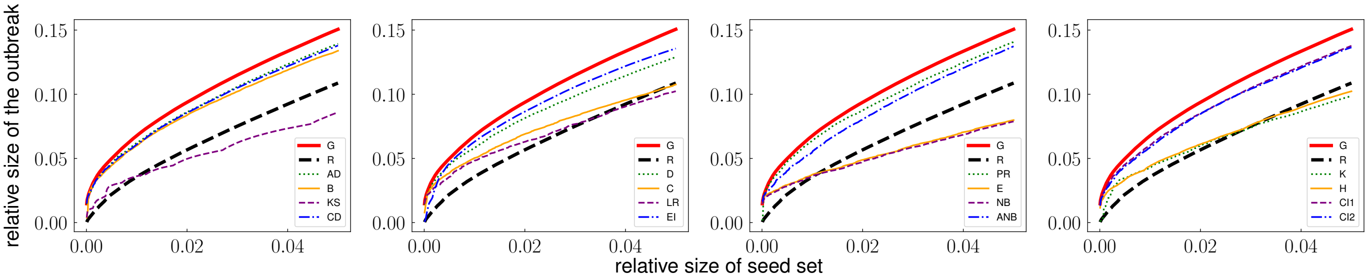

Figure 490: Cond-Mat, 1995-1999 - p=1.0p_c

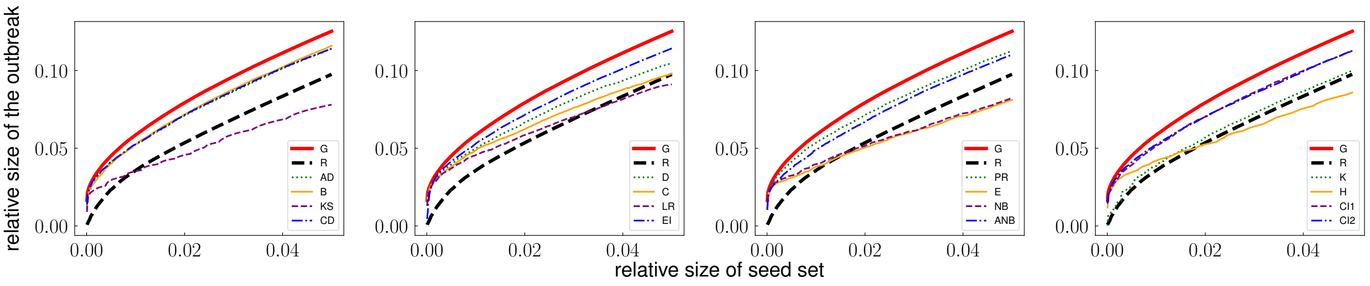

Figure 491: Astrophysics - p=1.0p_c

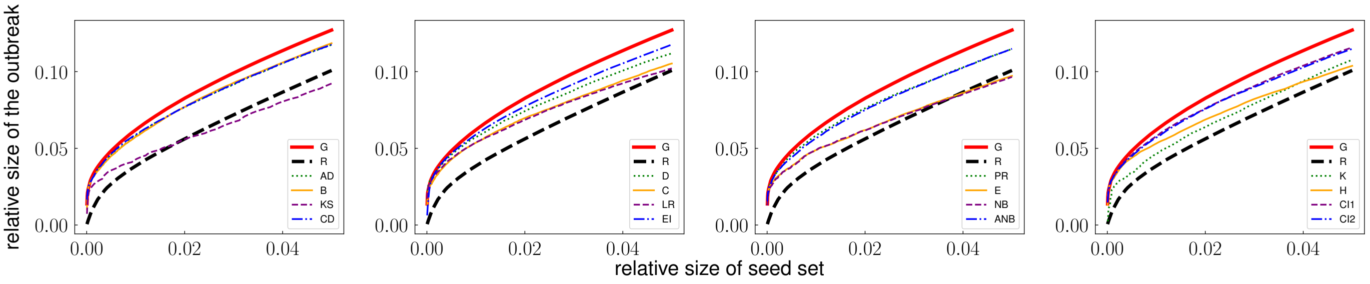

Figure 492: AstroPhys, 1993-2003 - p=1.0p_c

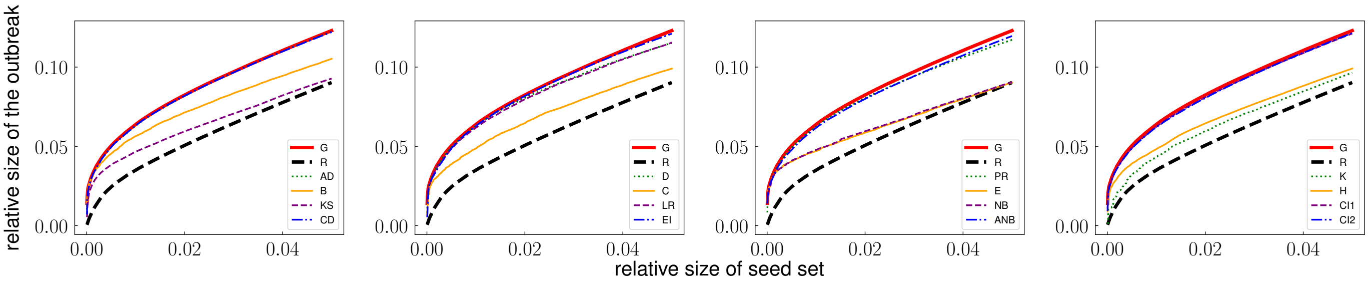

Figure 493: Marvel - p=1.0p_c

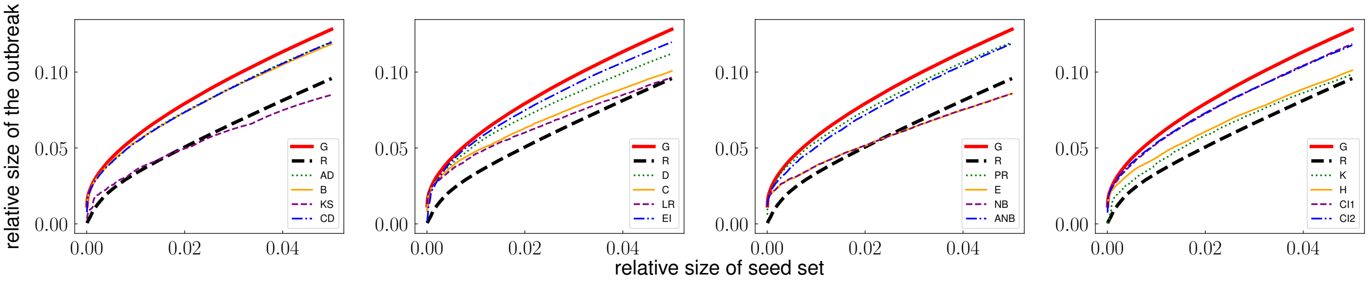

Figure 494: Cond-Mat, 1993-2003 - p=1.0p_c

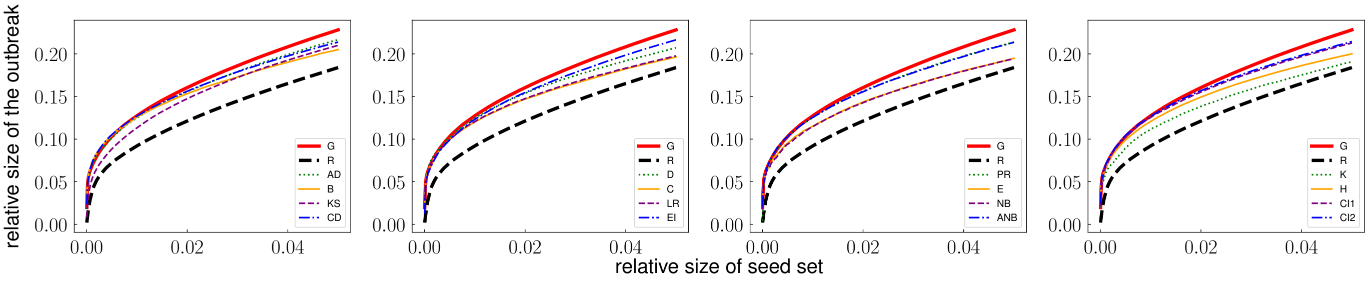

Figure 495: Gnutella, Aug. 25, 2002 - p=1.0p_c

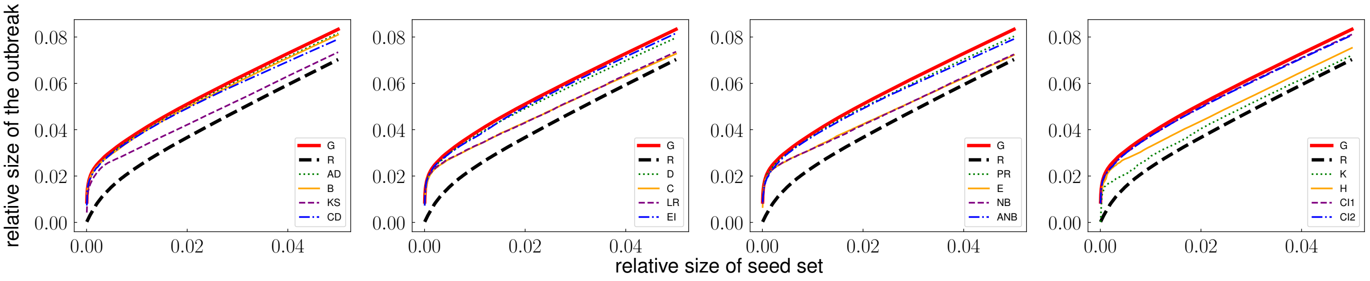

Figure 496: Internet - p=1.0p_c

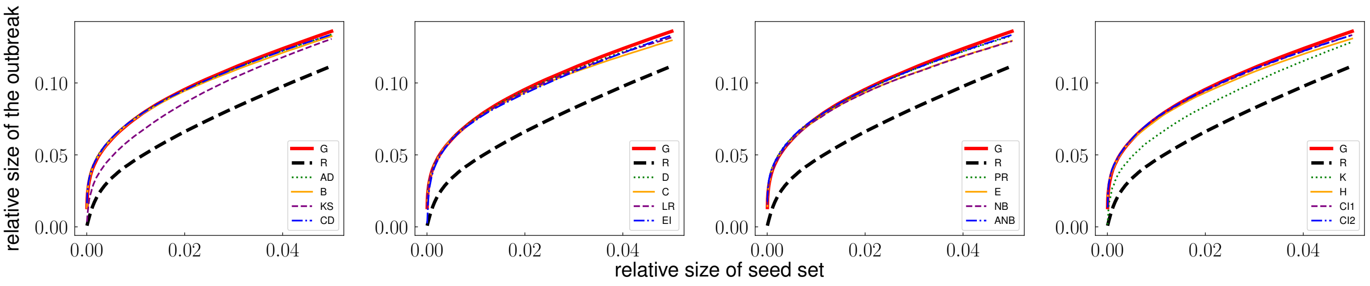

Figure 497: Thesaurus - p=1.0p_c

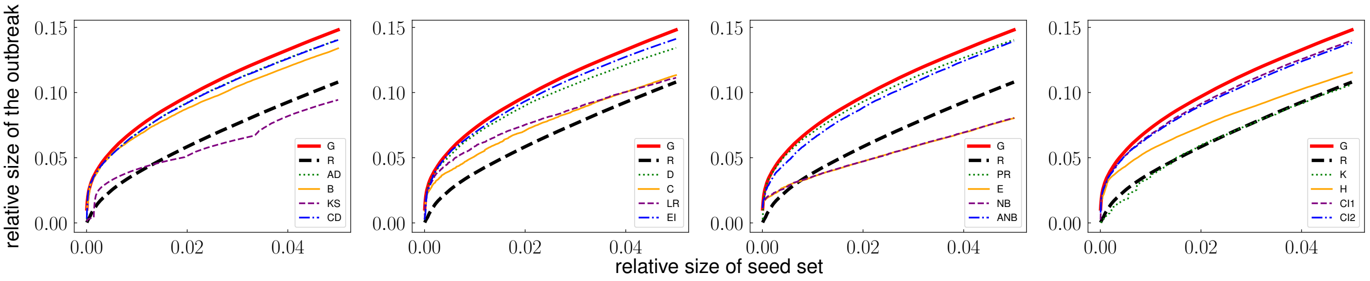

Figure 498: Cora - p=1.0p_c

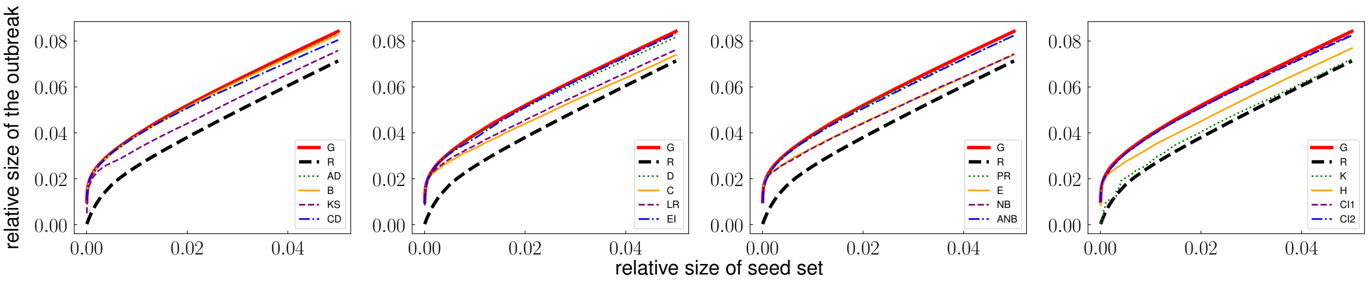

Figure 499: AS Caida - p=1.0p_c

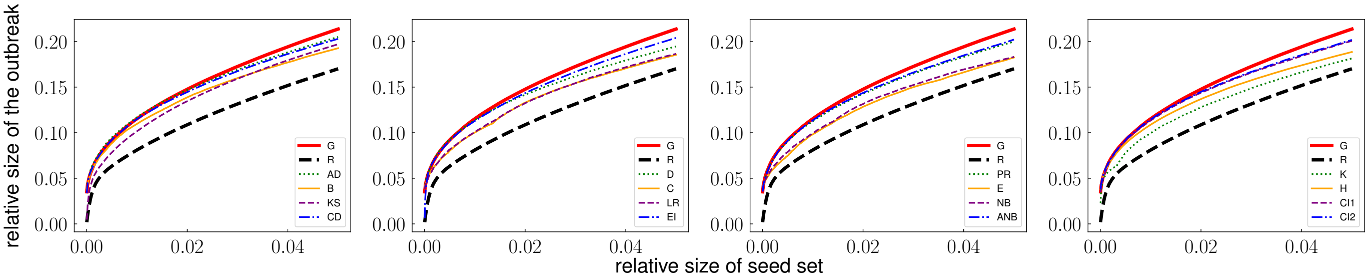

Figure 500: Gnutella, Aug. 24, 2002 - p=1.0p_c

3.1.3 Supercritical Regime

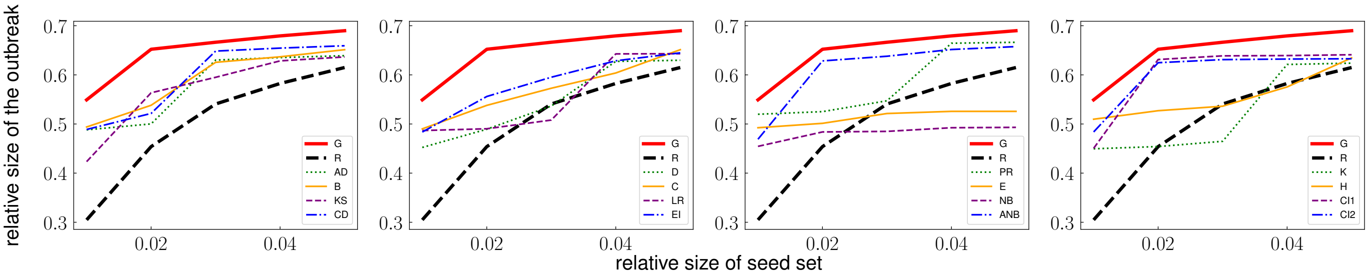

Figure 501: Political books - p=2.0p_c

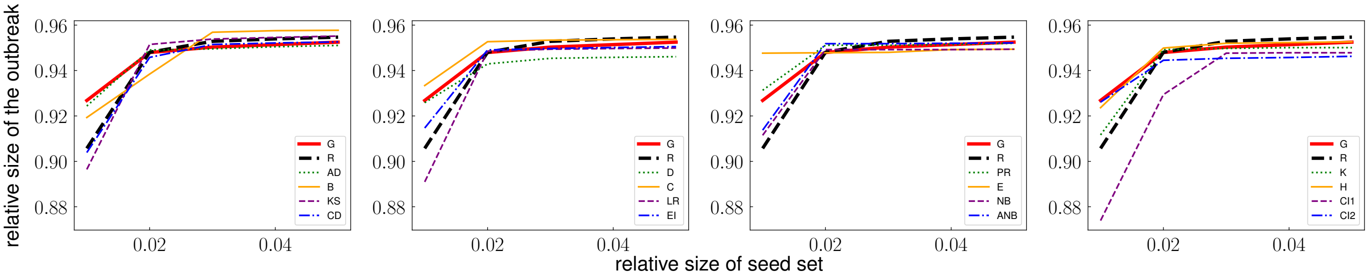

Figure 502: College football - p=2.0p_c

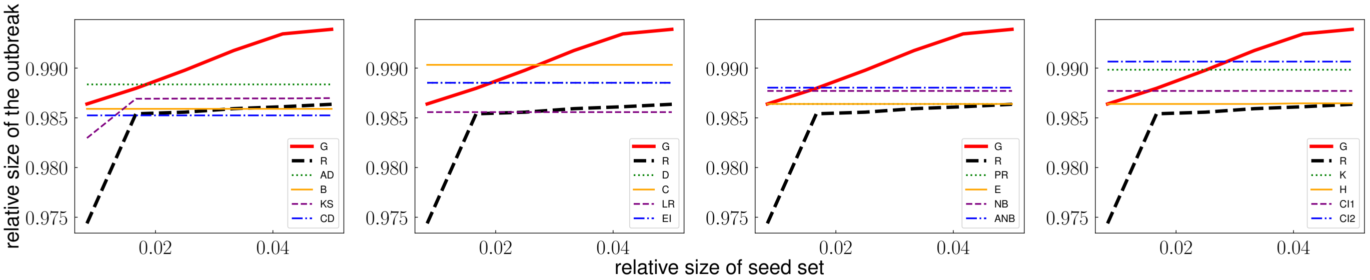

Figure 503: S208 - p=2.0p_c

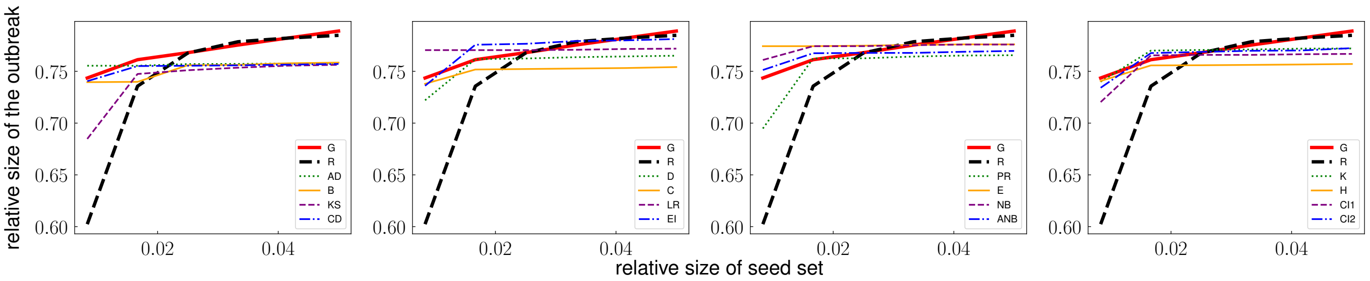

Figure 504: High school, 2011 - p=2.0p_c

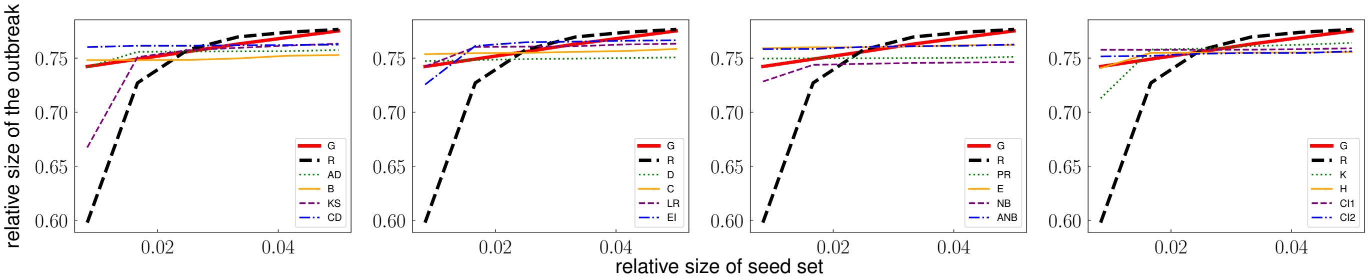

Figure 505: Bay Dry - p=2.0p_c

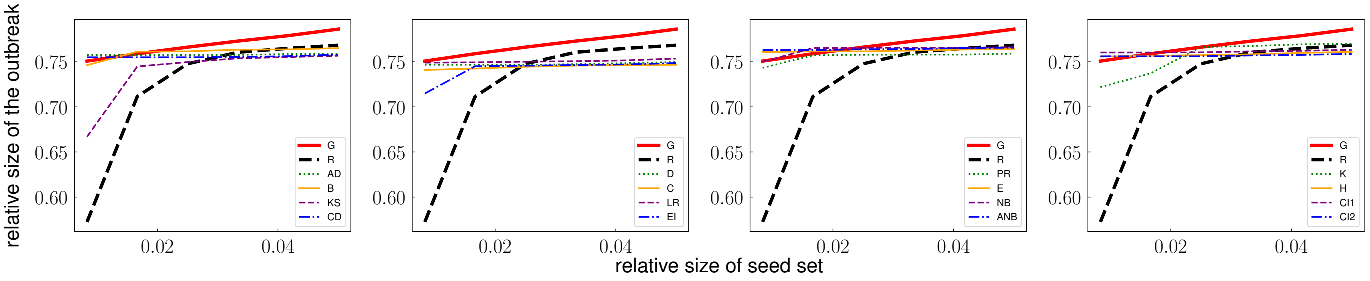

Figure 506: Bay Wet - p=2.0p_c

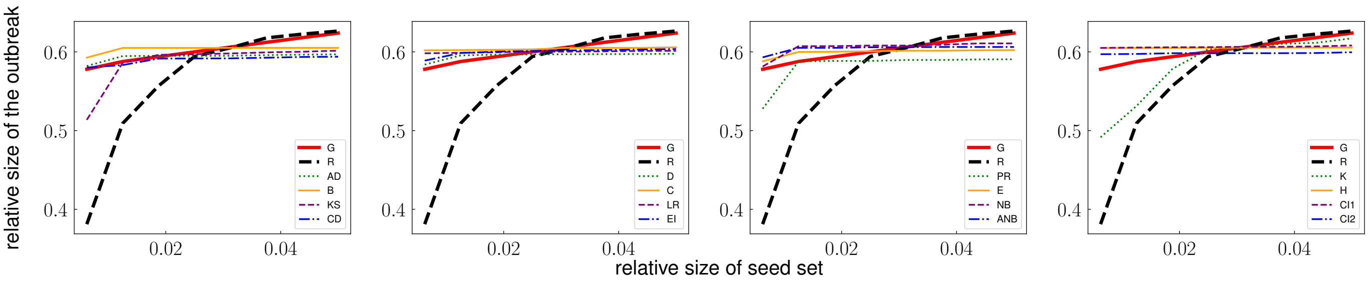

Figure 507: Radoslaw Email - p=2.0p_c

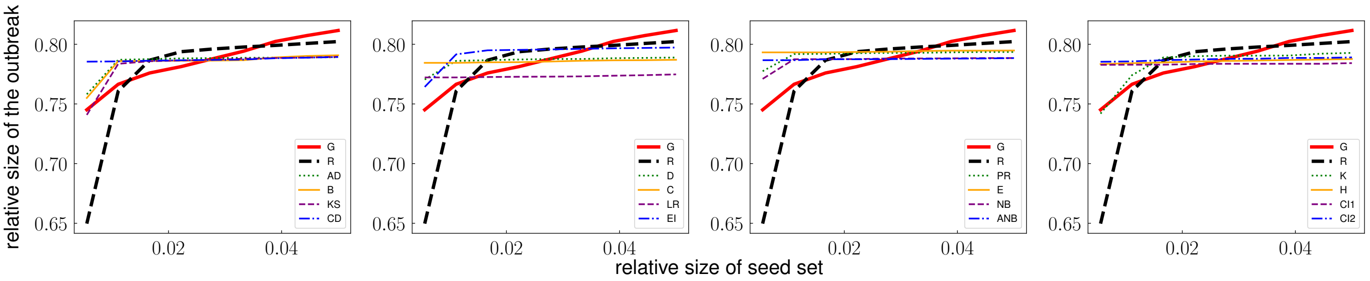

Figure 508: High school, 2012 - p=2.0p_c

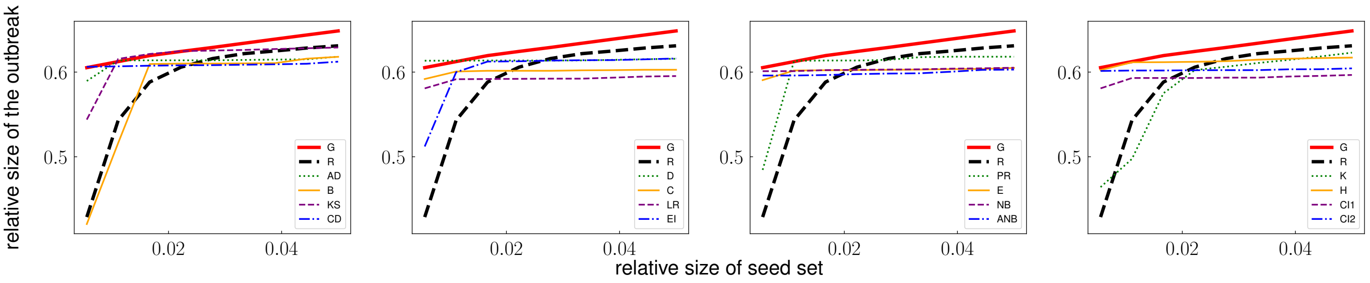

Figure 509: Little Rock Lake - p=2.0p_c

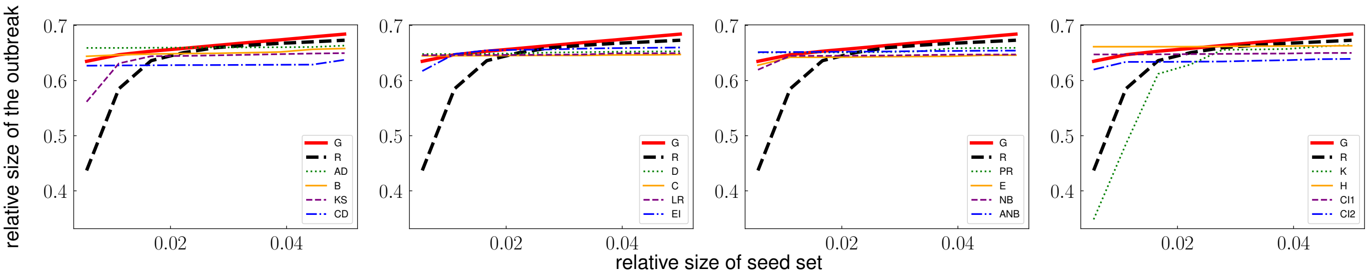

Figure 510: Jazz - p=2.0p_c

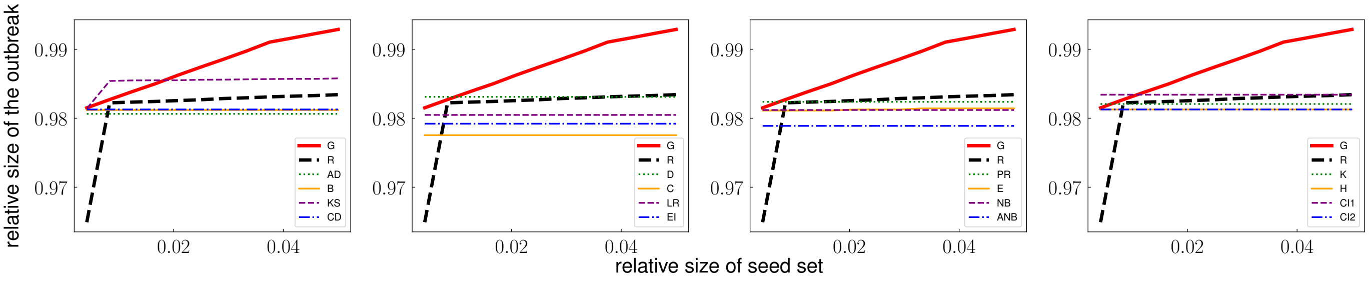

Figure 511: S420 - p=2.0p_c

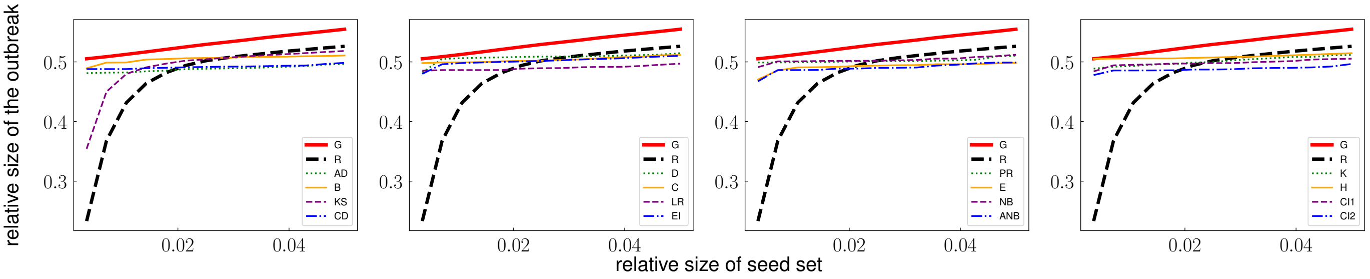

Figure 512: C. Elegans, neural - p=2.0p_c

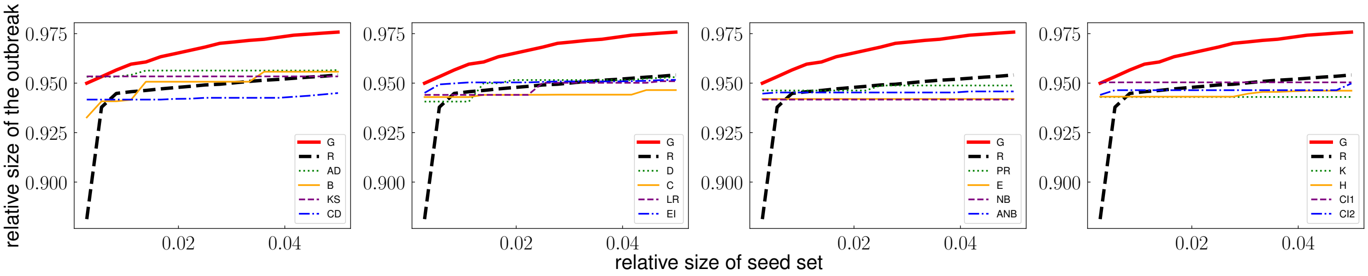

Figure 513: Network Science - p=2.0p_c

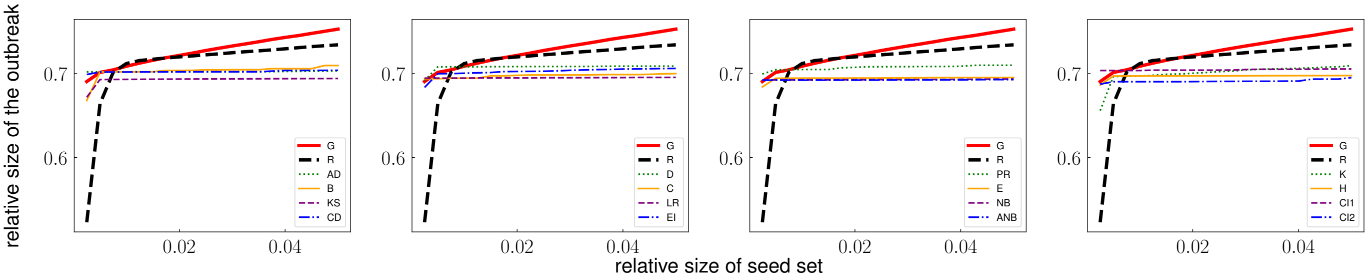

Figure 514: Dublin - p=2.0p_c

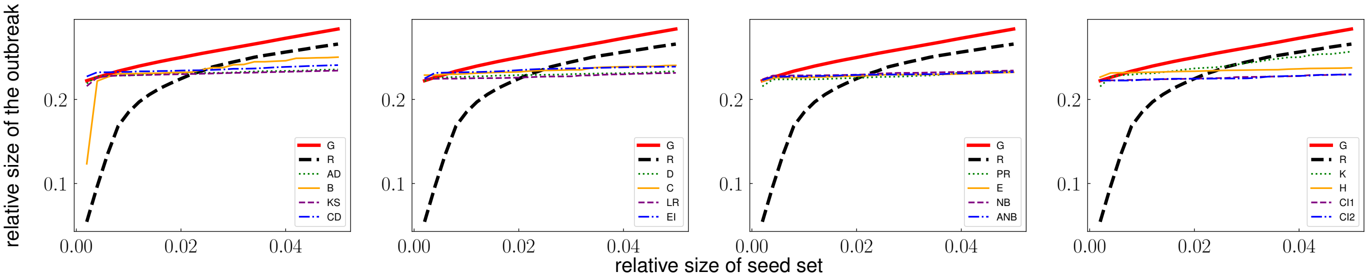

Figure 515: US Air Trasportation - p=2.0p_c

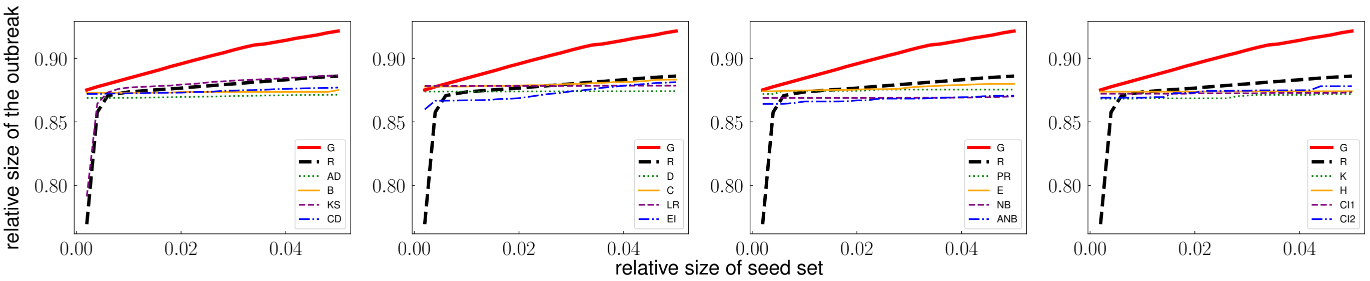

Figure 516: S838 - p=2.0p_c

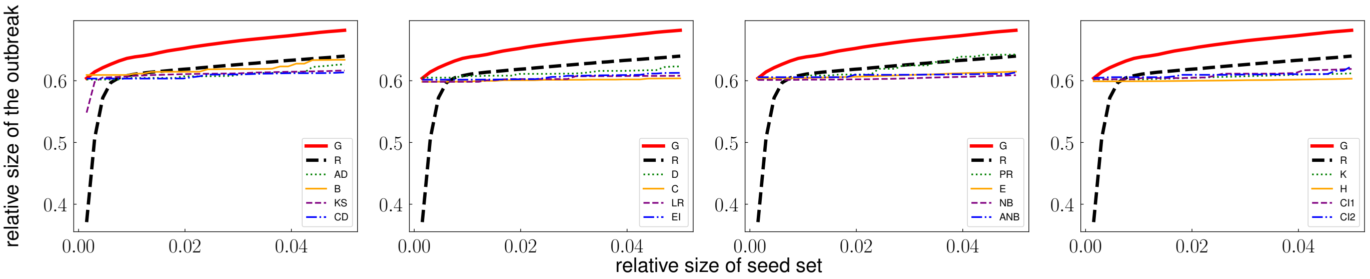

Figure 517: Yeast, transcription - p=2.0p_c

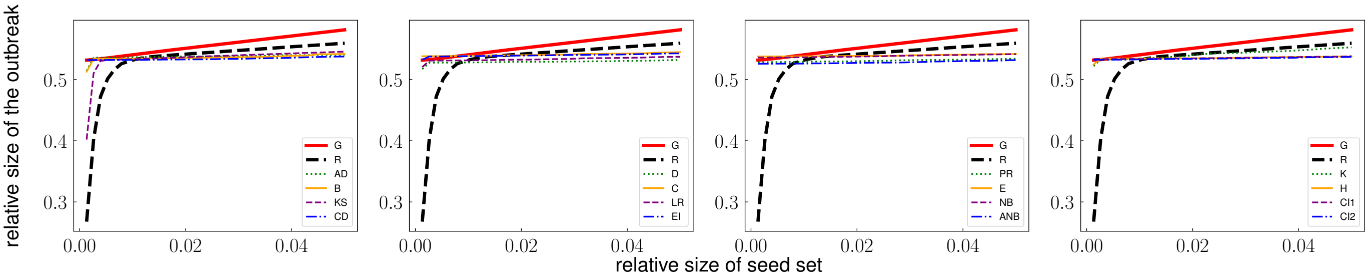

Figure 518: Caltech - p=2.0p_c

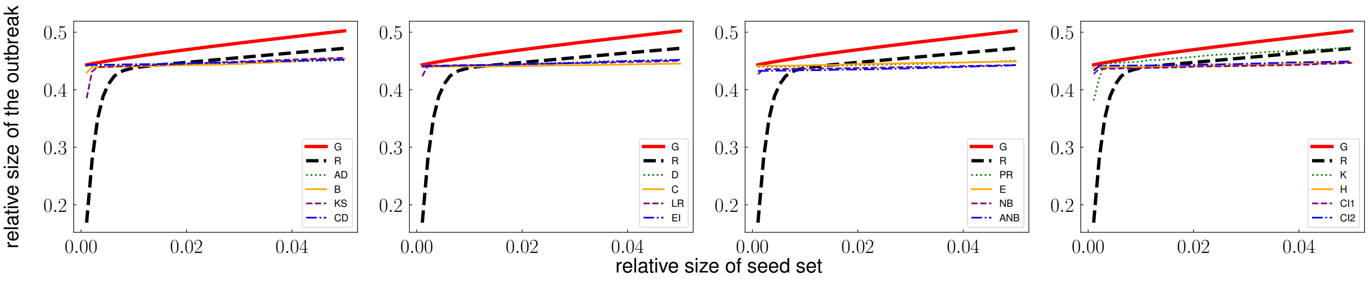

Figure 519: Reed - p=2.0p_c

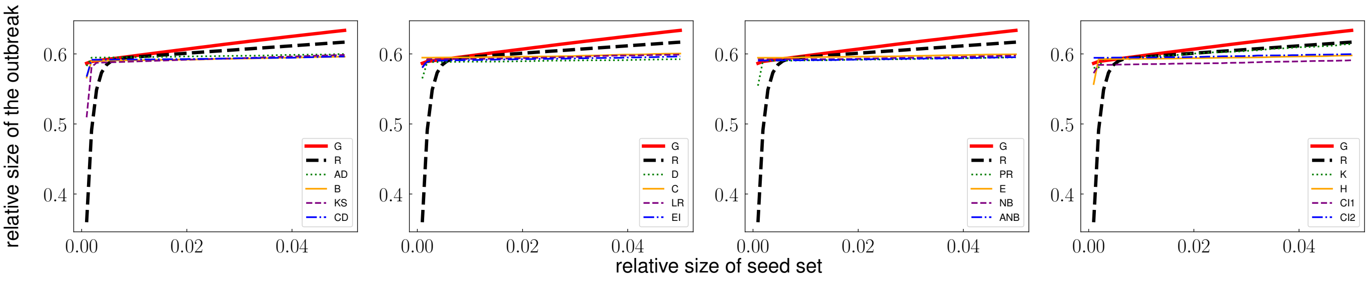

Figure 520: Mouse retina - p=2.0p_c

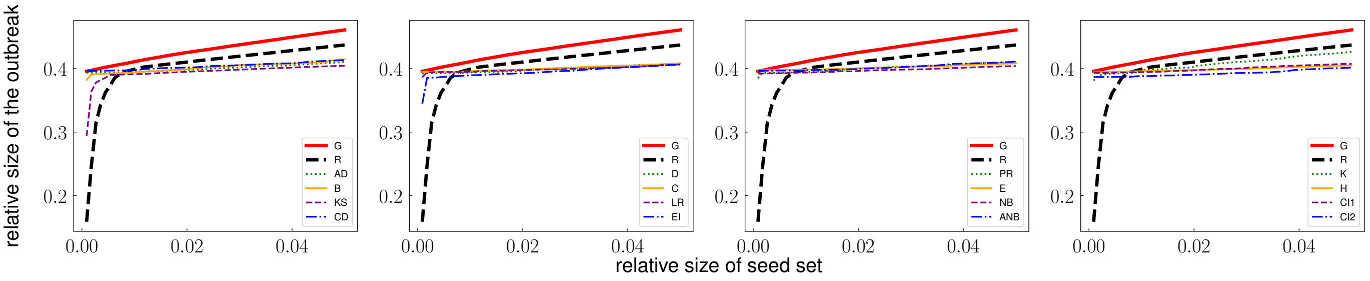

Figure 521: URV email - p=2.0p_c

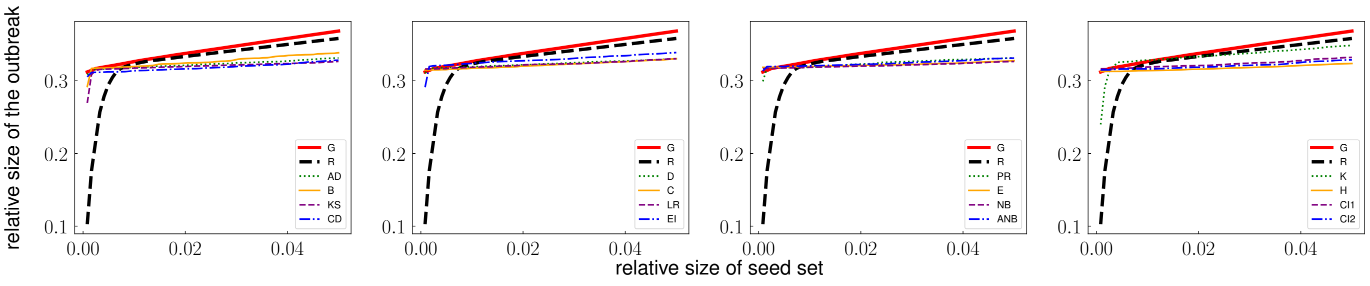

Figure 522: Political blogs - p=2.0p_c

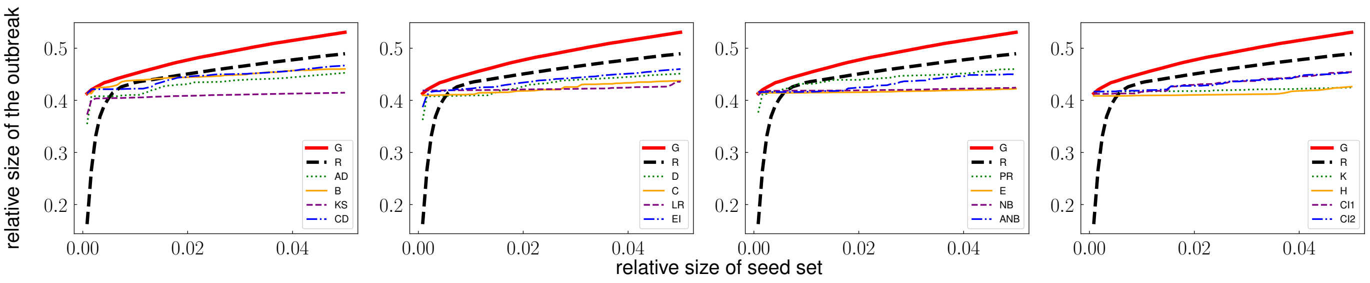

Figure 523: Air traffic - p=2.0p_c

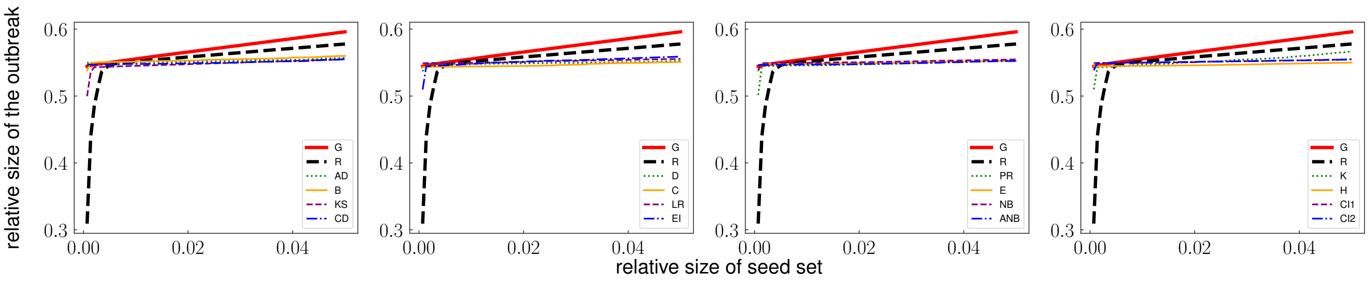

Figure 524: Haverford - p=2.0p_c

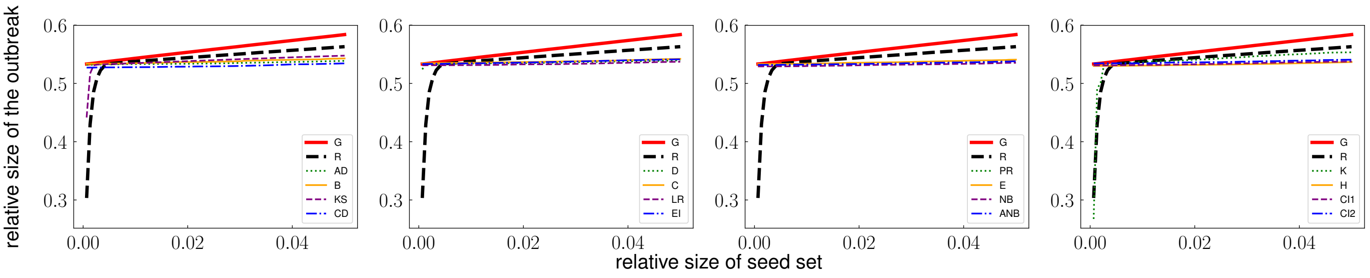

Figure 525: Simmons - p=2.0p_c

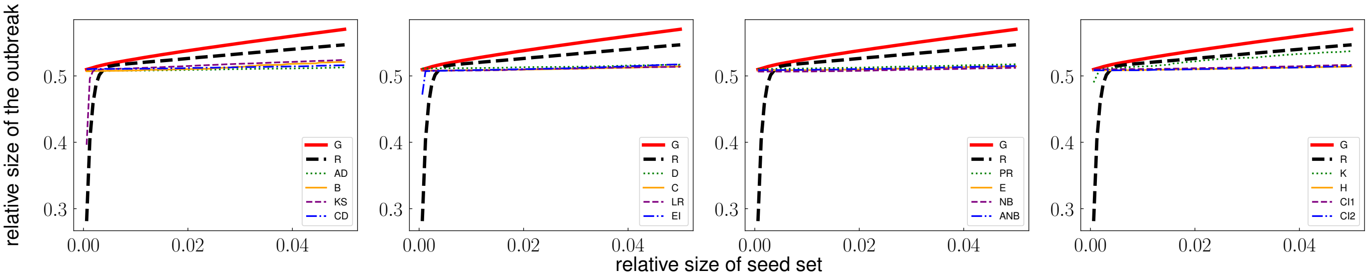

Figure 526: Swarthmore - p=2.0p_c

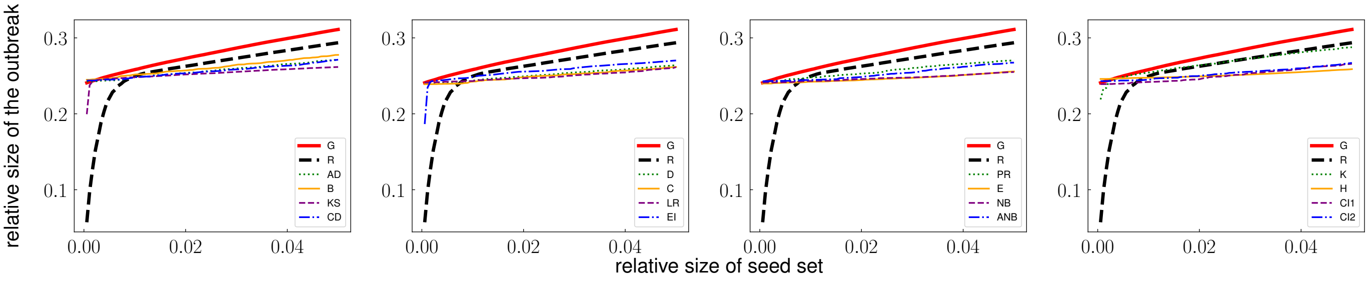

Figure 527: Petster, hamster - p=2.0p_c

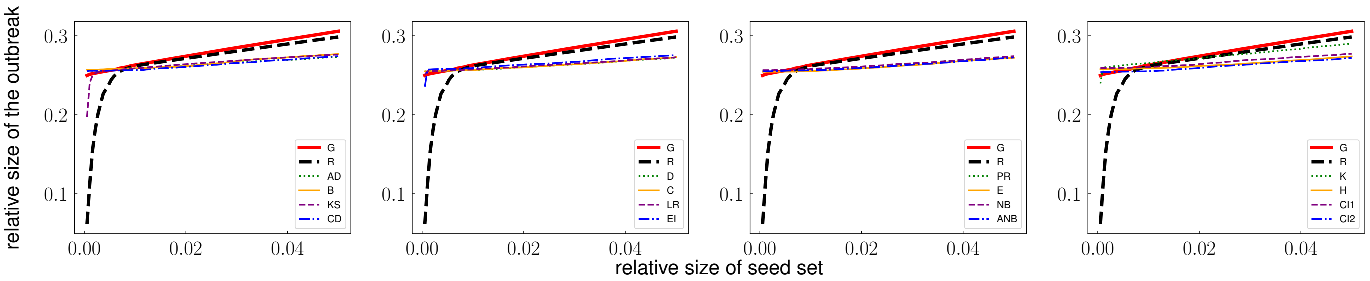

Figure 528: UC Irvine - p=2.0p_c

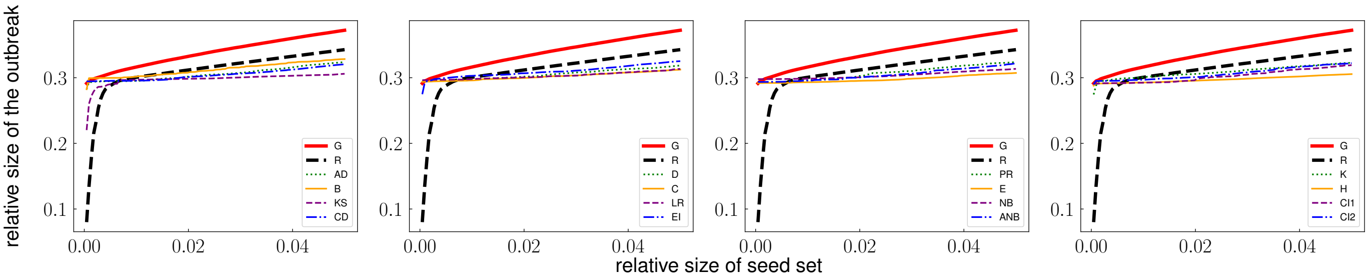

Figure 529: Yeast, protein - p=2.0p_c

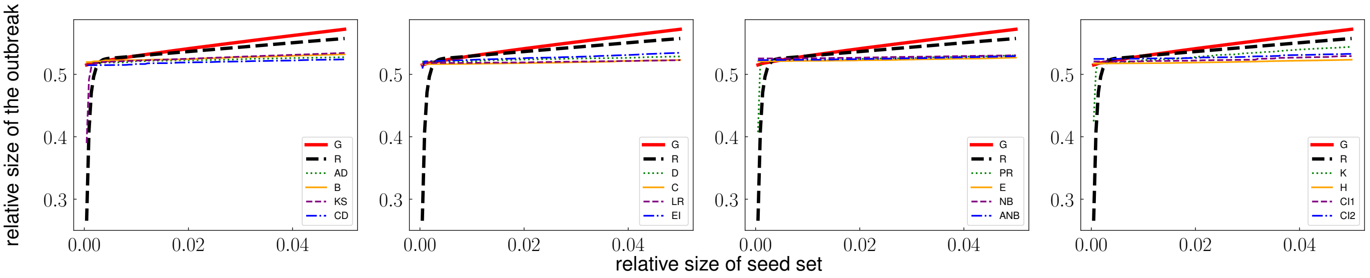

Figure 530: Amherst - p=2.0p_c

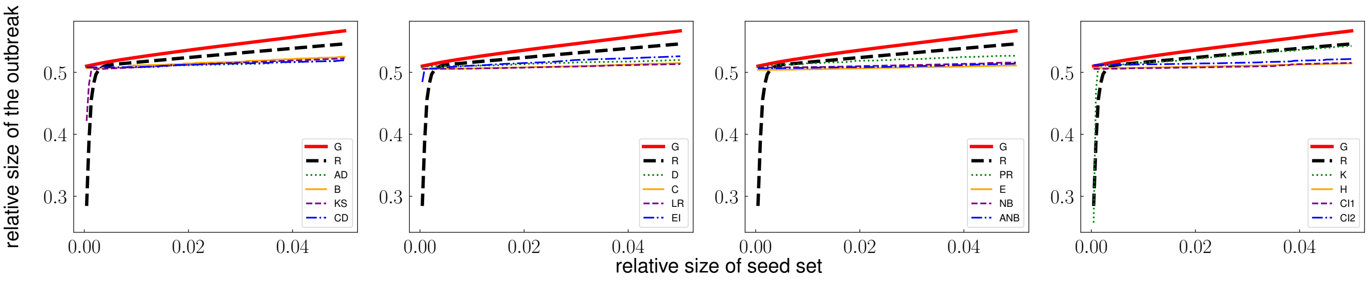

Figure 531: Bowdoin - p=2.0p_c

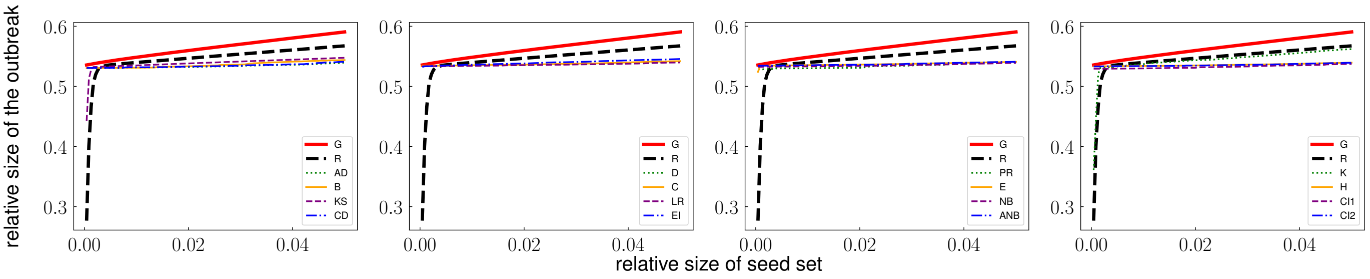

Figure 532: Hamilton - p=2.0p_c

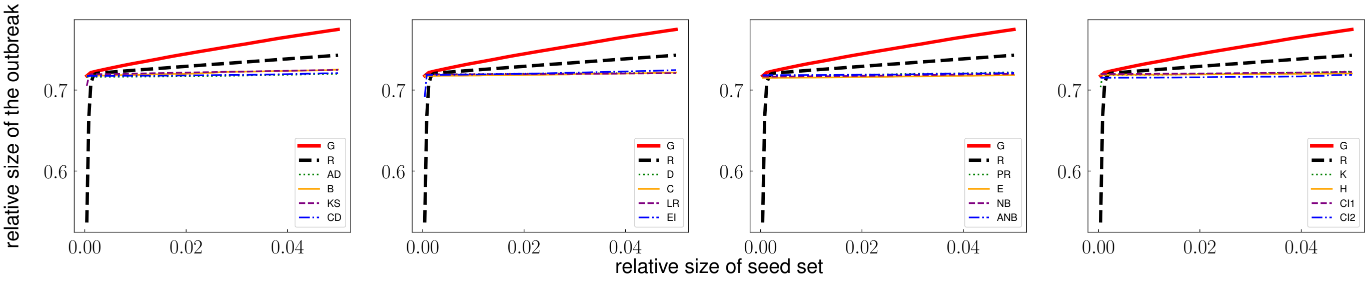

Figure 533: Adolescent health - p=2.0p_c

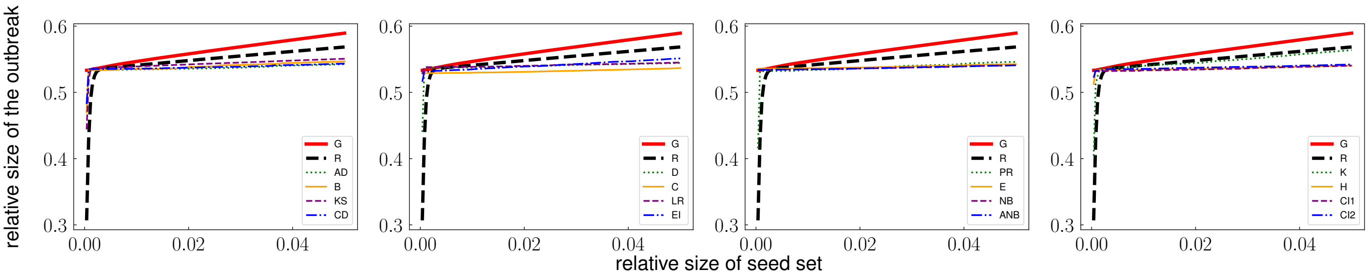

Figure 534: Trinity - p=2.0p_c

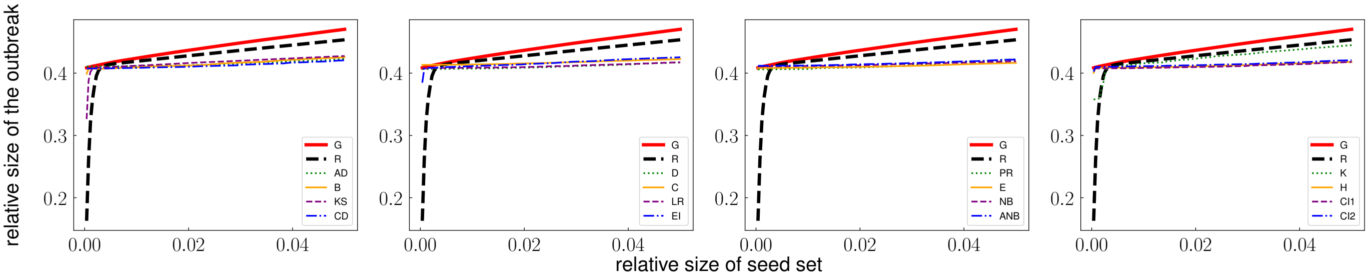

Figure 535: USFCA - p=2.0p_c

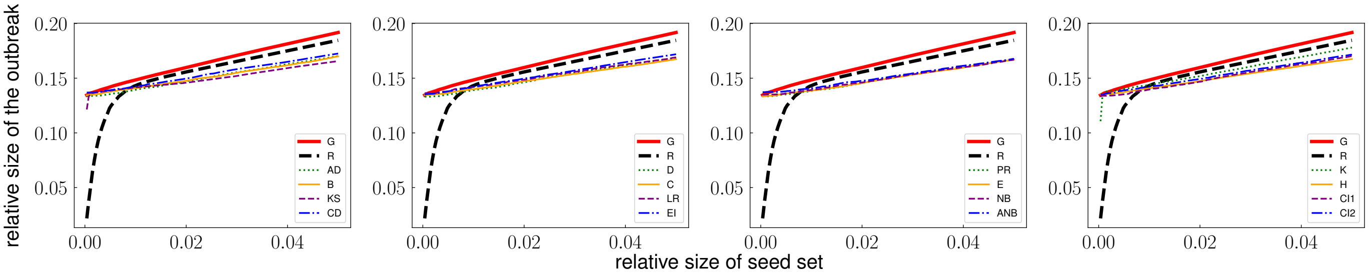

Figure 536: Japanese - p=2.0p_c

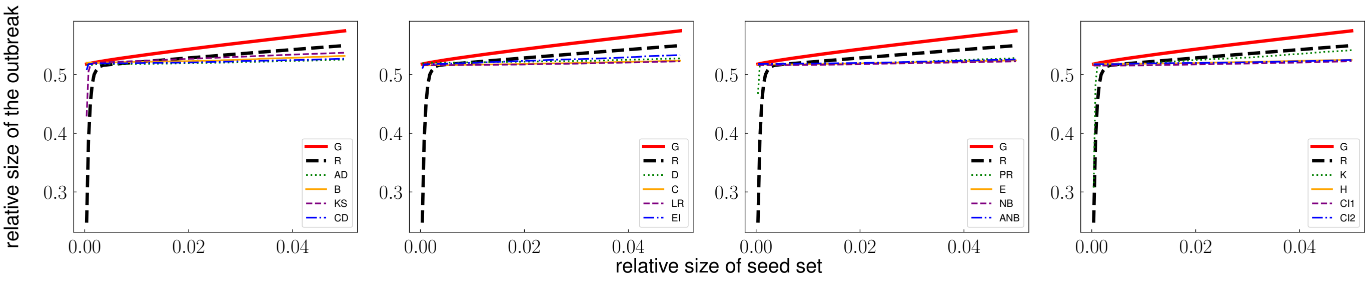

Figure 537: Williams - p=2.0p_c

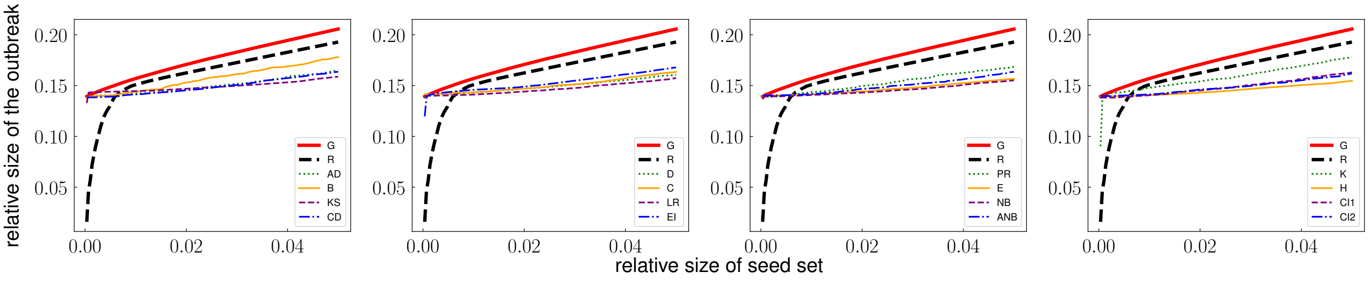

Figure 538: Open flights - p=2.0p_c

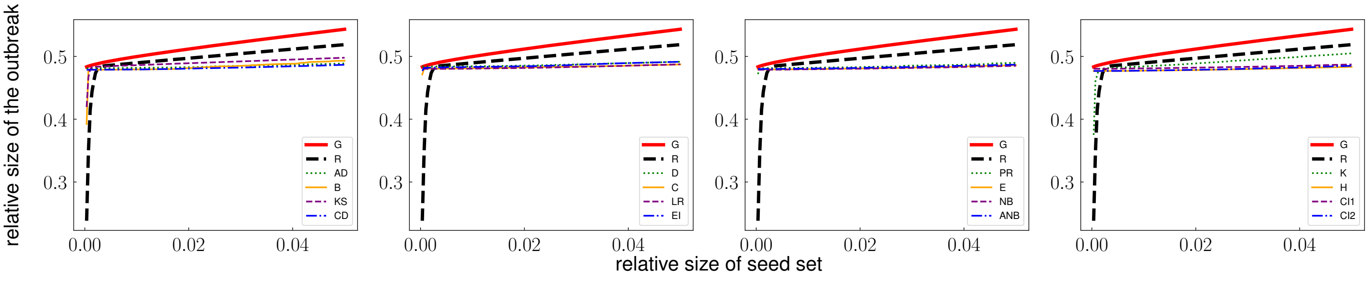

Figure 539: Oberlin - p=2.0p_c

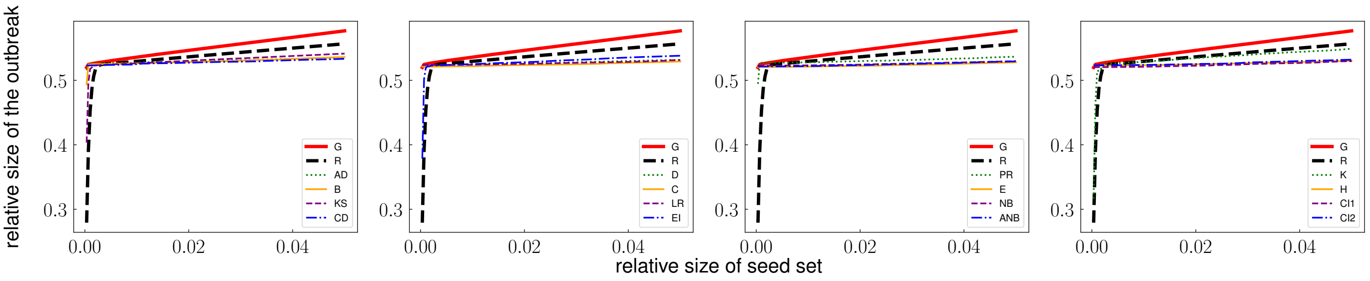

Figure 540: Smith - p=2.0p_c

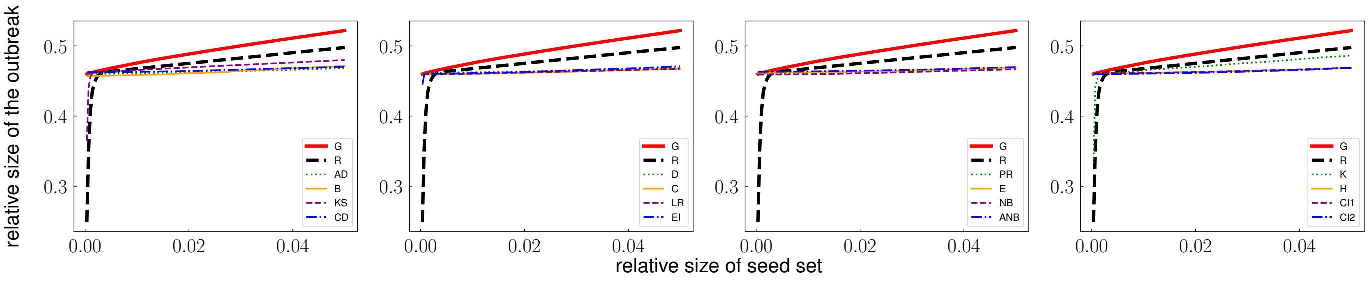

Figure 541: Wellesley - p=2.0p_c

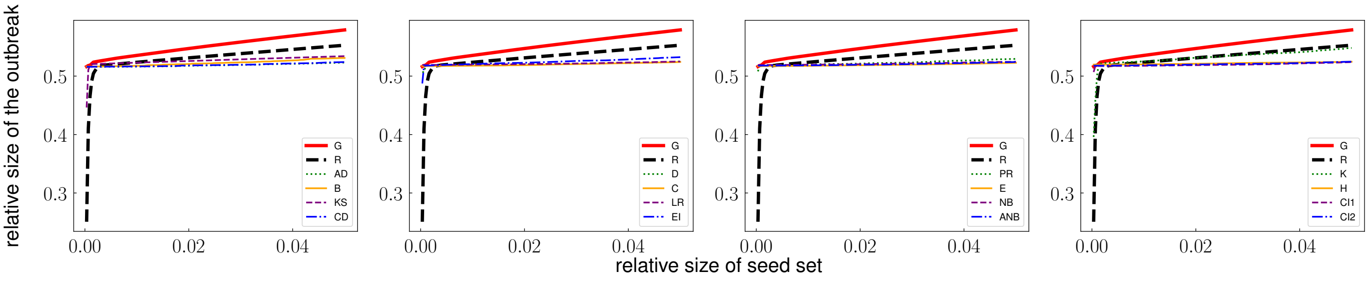

Figure 542: Vassar - p=2.0p_c

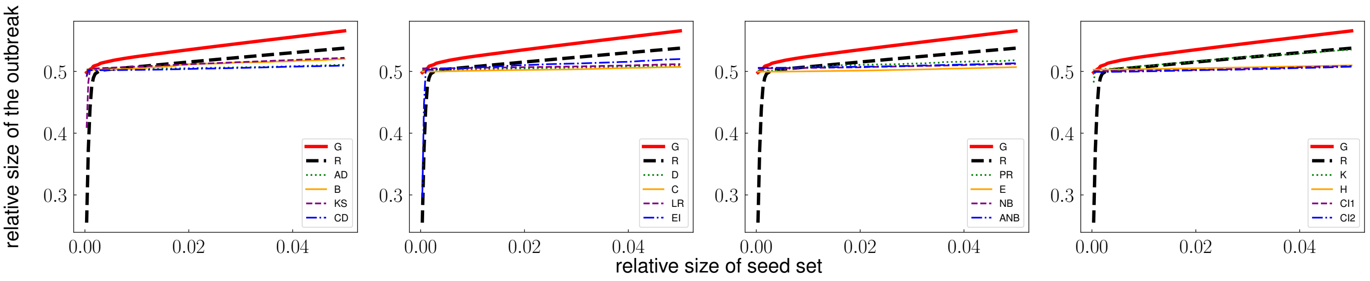

Figure 543: Middlebury - p=2.0p$_c$

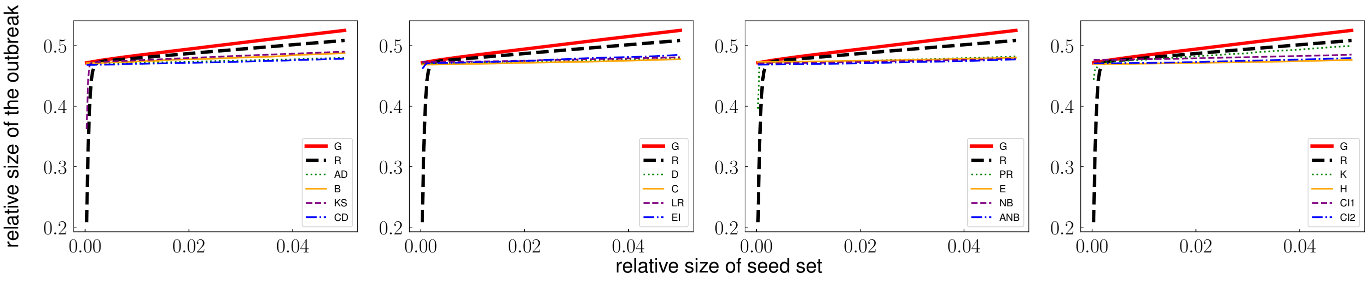

Figure 544: Pepperdine - p=2.0p$_c$

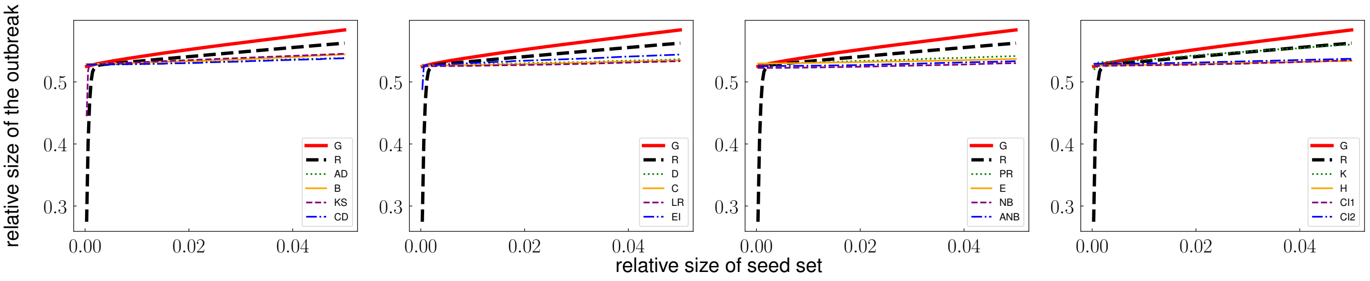

Figure 545: Colgate - p=2.0p$_c$

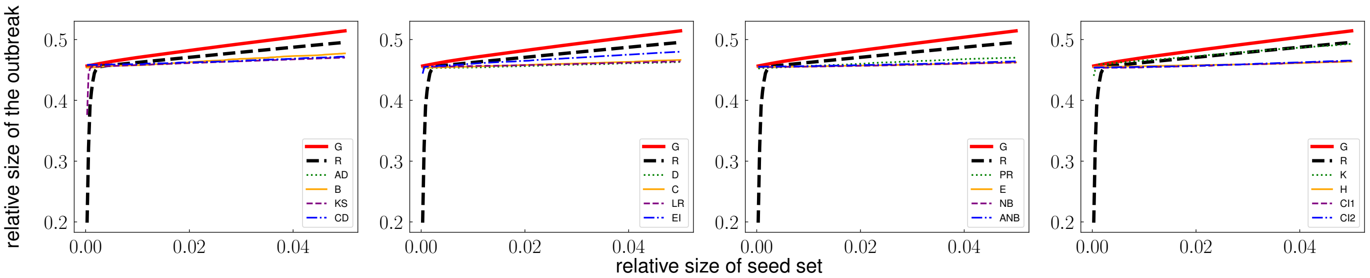

Figure 546: Santa - p=2.0p_c

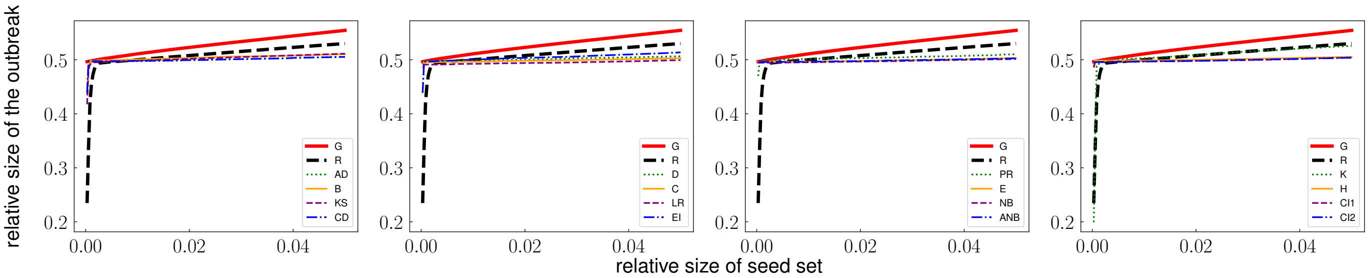

Figure 547: Wesleyan - p=2.0p_c

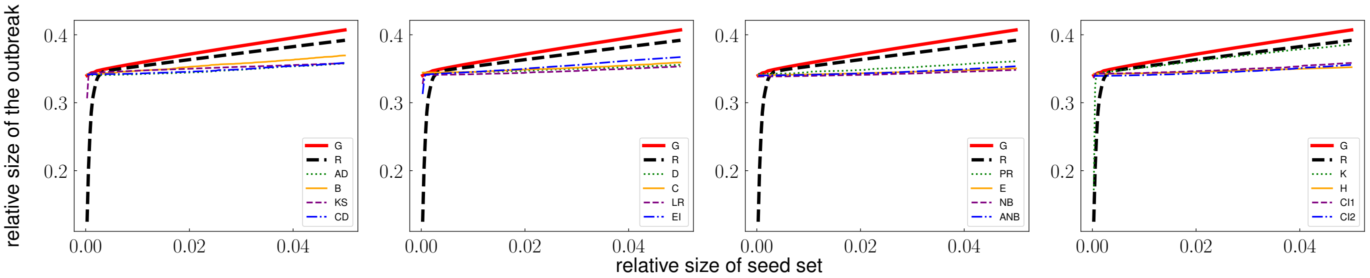

Figure 548: Mich - p=2.0p_c

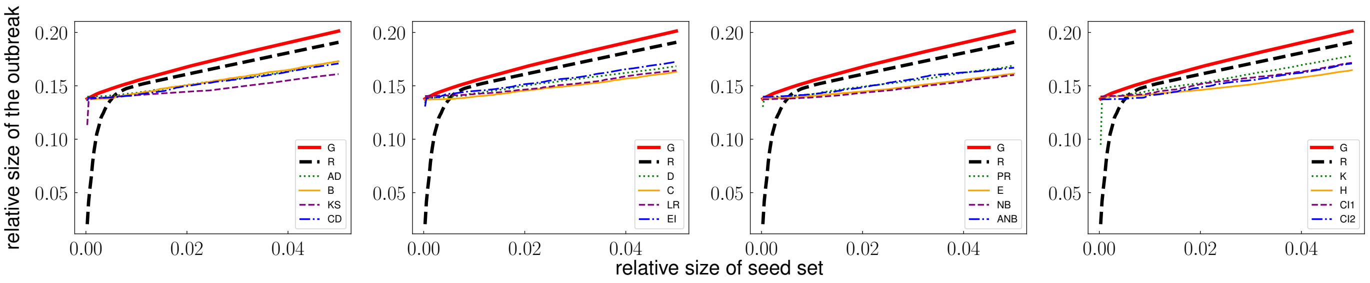

Figure 549: Bitcoin Alpha - p=2.0p$_c$

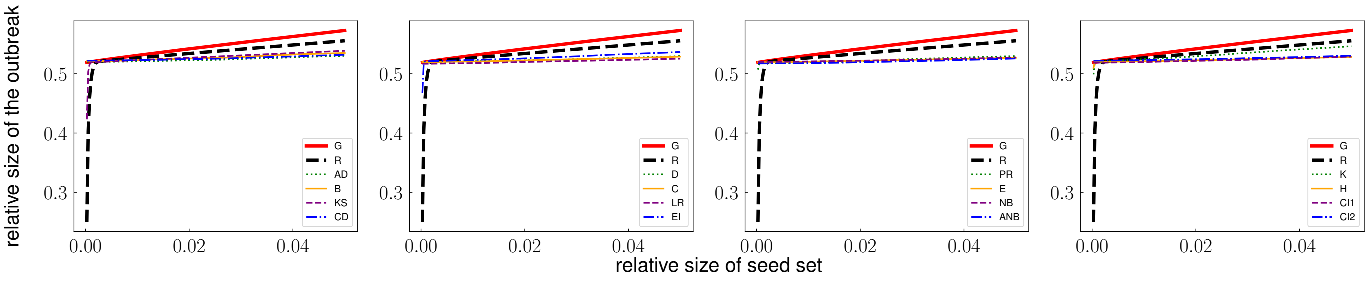

Figure 550: Bucknell - p=2.0p$_c$

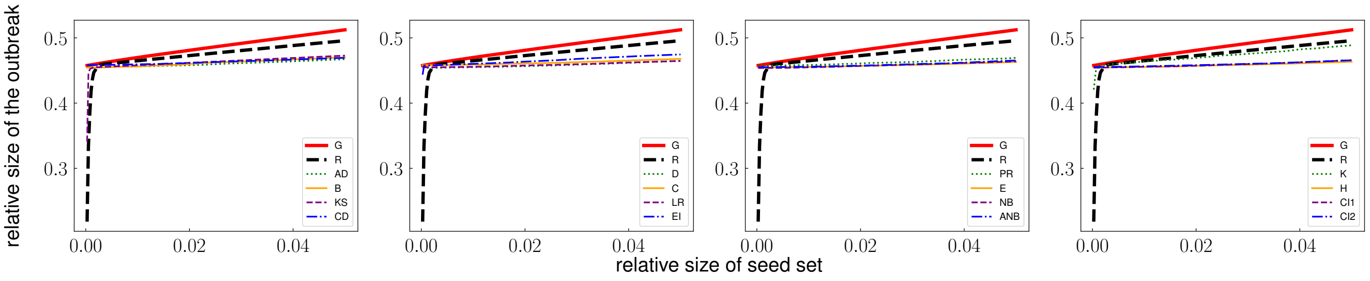

Figure 551: Brandeis - p=2.0p$_c$

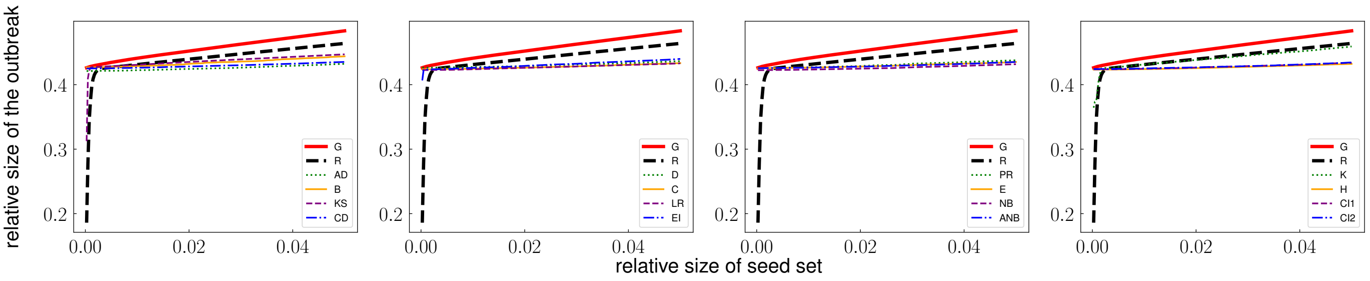

Figure 552: Howard - p=2.0p_c

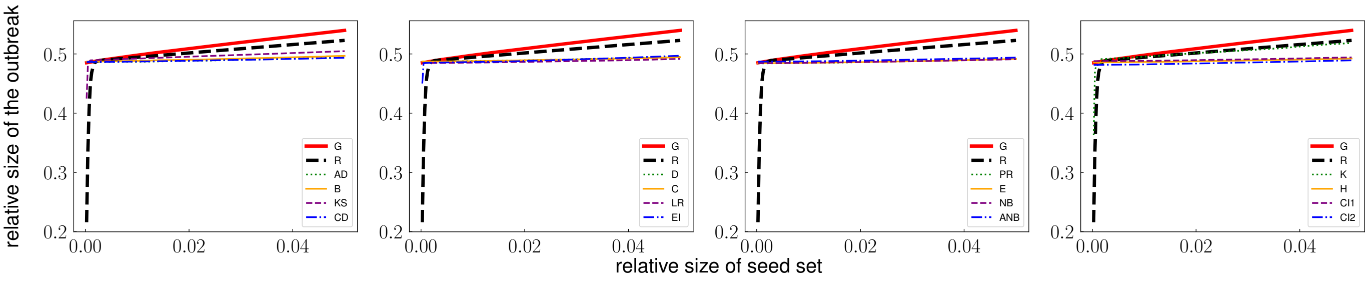

Figure 553: Rice - p=2.0p_c

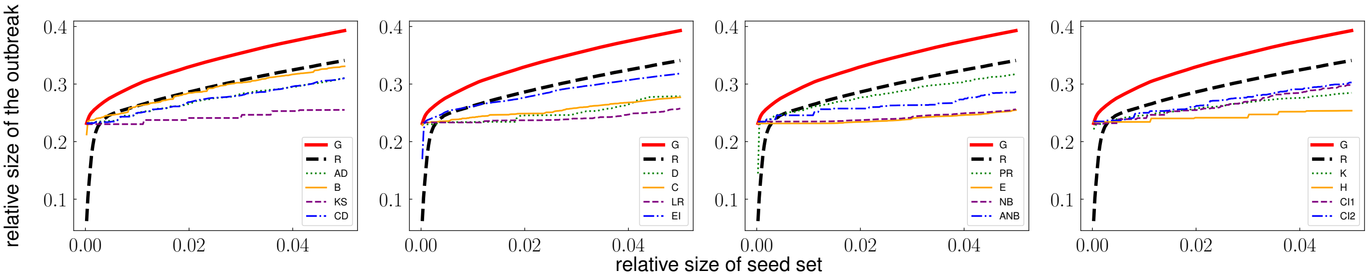

Figure 554: GR-QC, 1993-2003 - p=2.0p_c

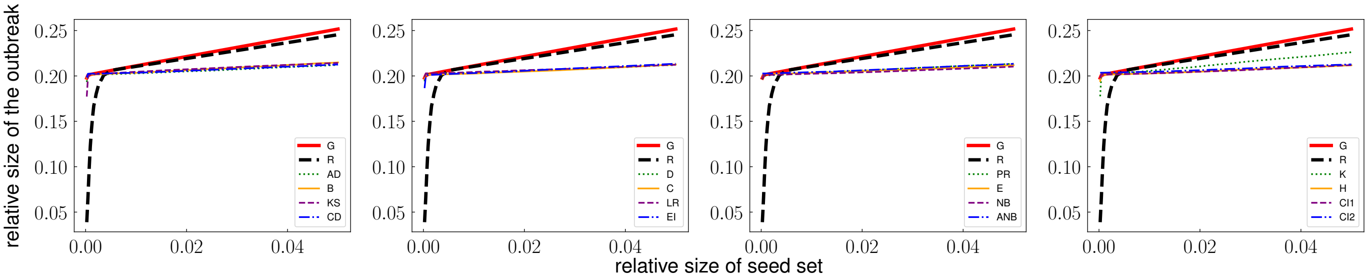

Figure 555: Tennis - p=2.0p_c

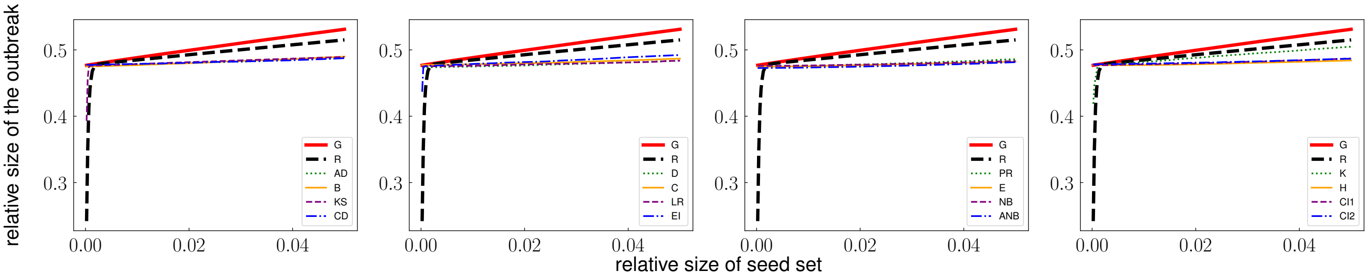

Figure 556: Rochester - p=2.0p_c

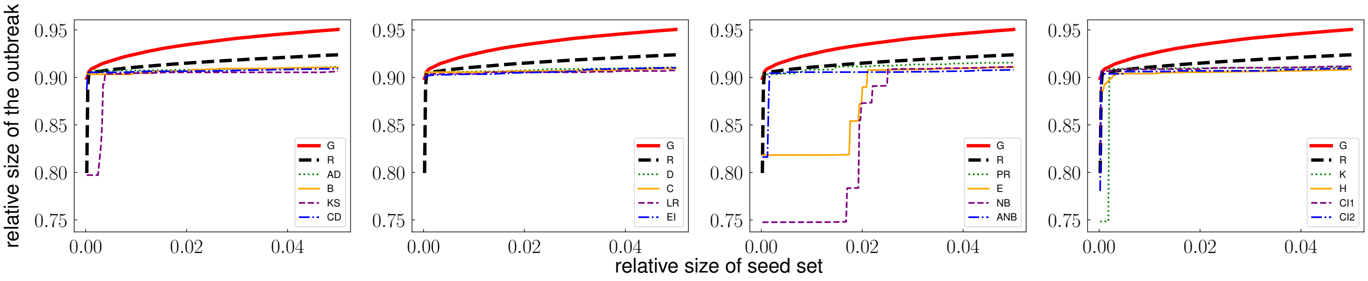

Figure 557: US Power grid - p=2.0p_c

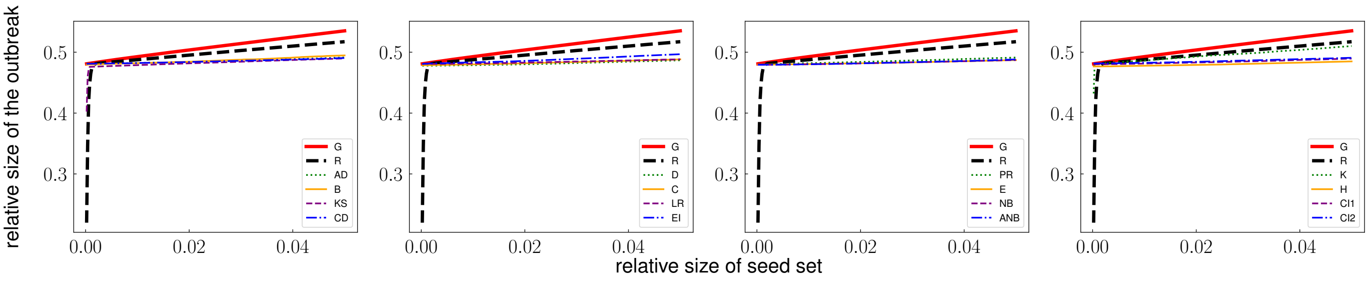

Figure 558: Lehigh - p=2.0p_c

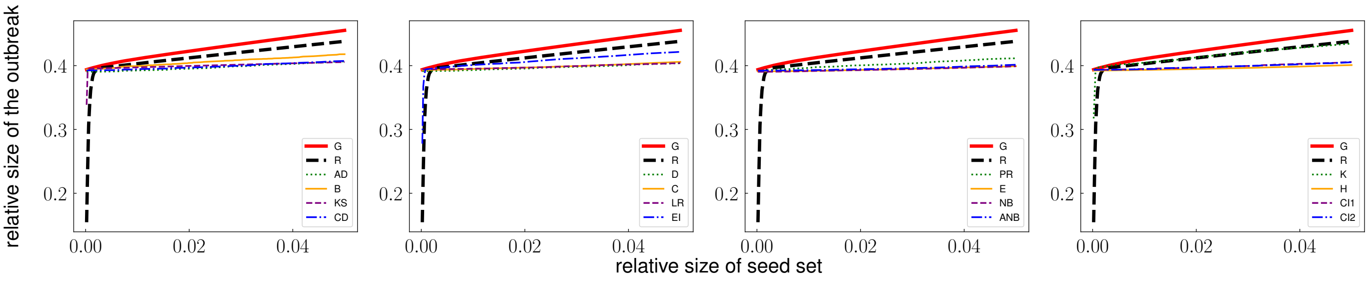

Figure 559: Johns Hopkins - p=2.0p_c

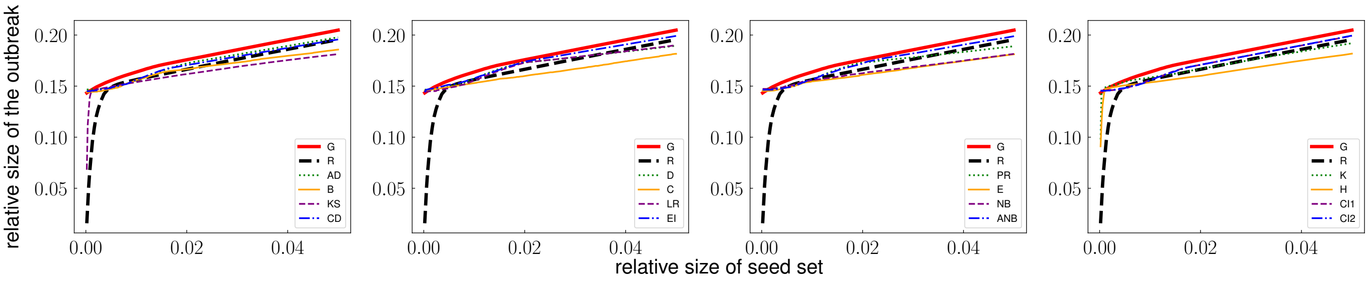

Figure 560: HT09 - p=2.0p_c

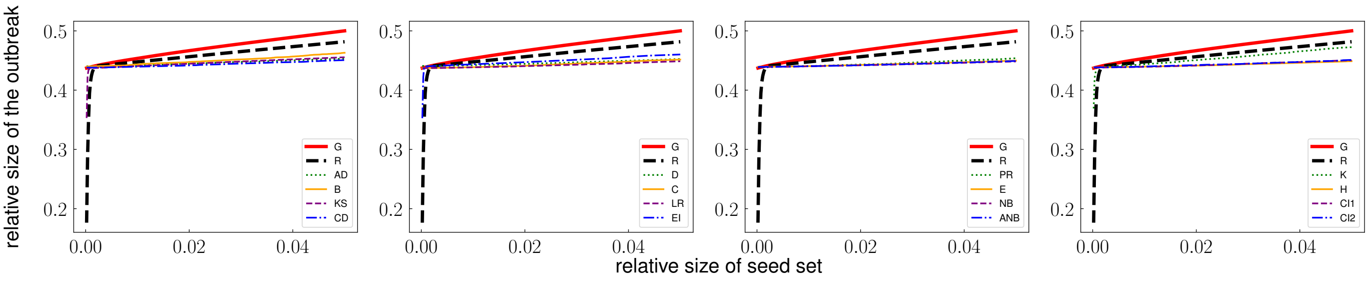

Figure 561: Wake - p=2.0p_c

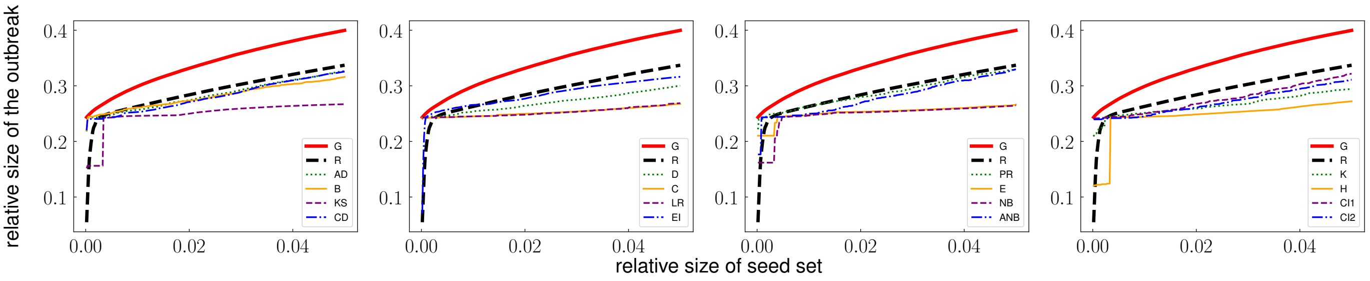

Figure 562: Hep-Th, 1995-1999 - p=2.0p_c

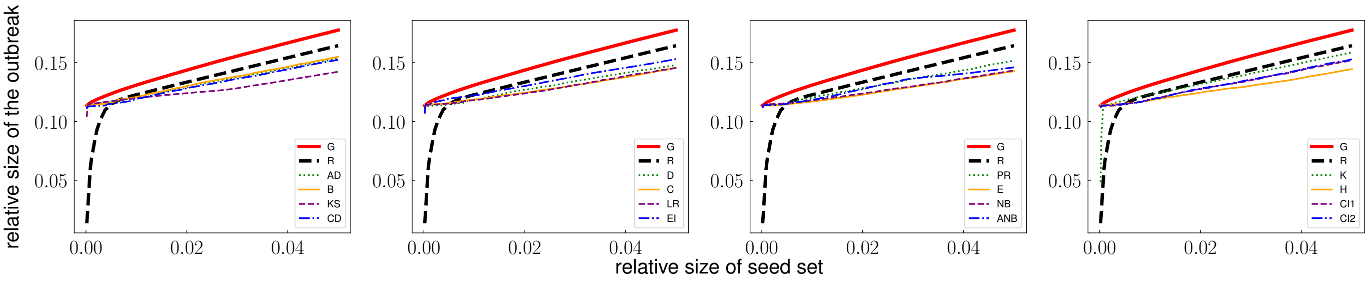

Figure 563: Bitcoin OTC - p=2.0p_c

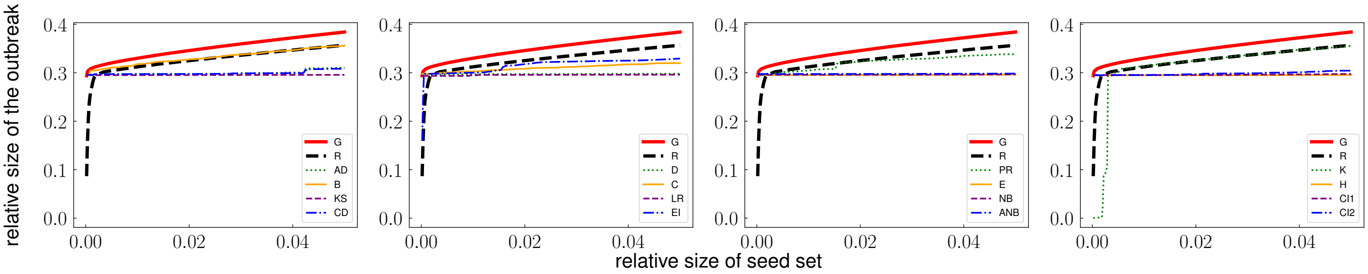

Figure 564: Reactome - p=2.0p$_c$

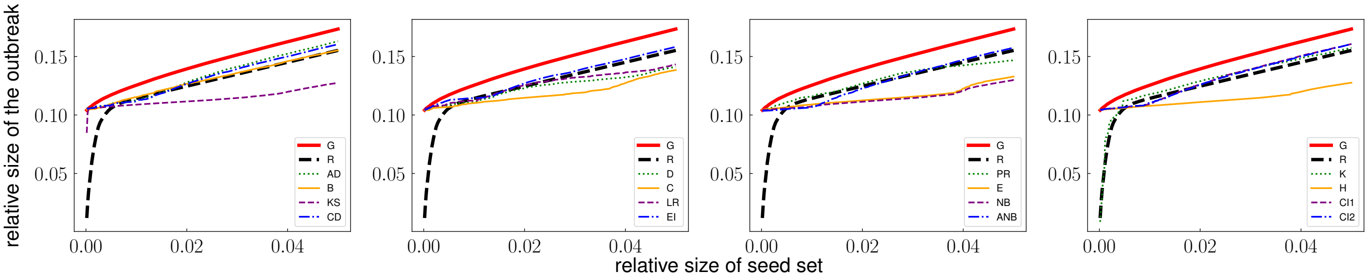

Figure 565: Jung - p=2.0p$_c$

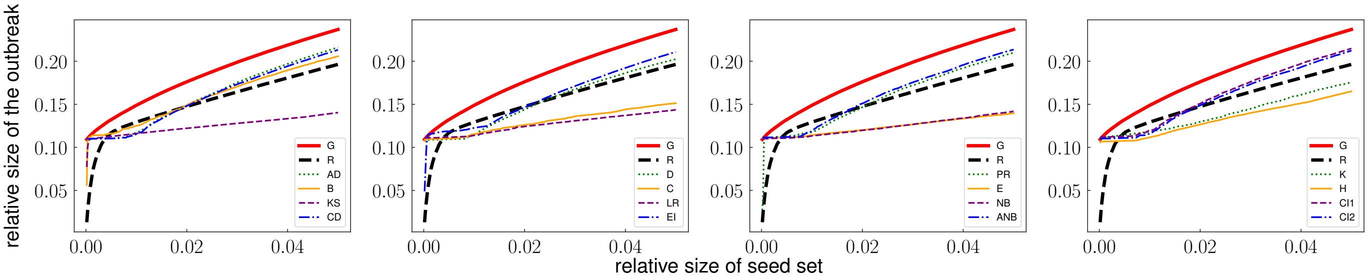

Figure 566: Gnutella, Aug. 8, 2002 - p=2.0p$_c$

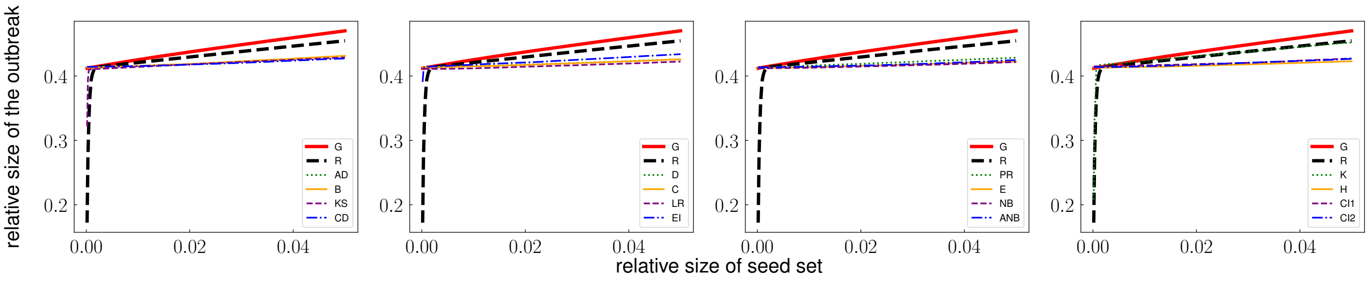

Figure 567: American - p=2.0p_c

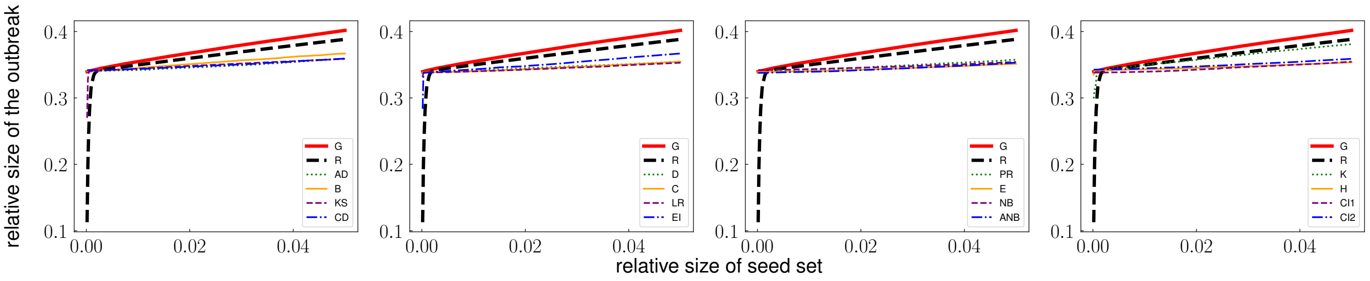

Figure 568: MIT - p=2.0p_c

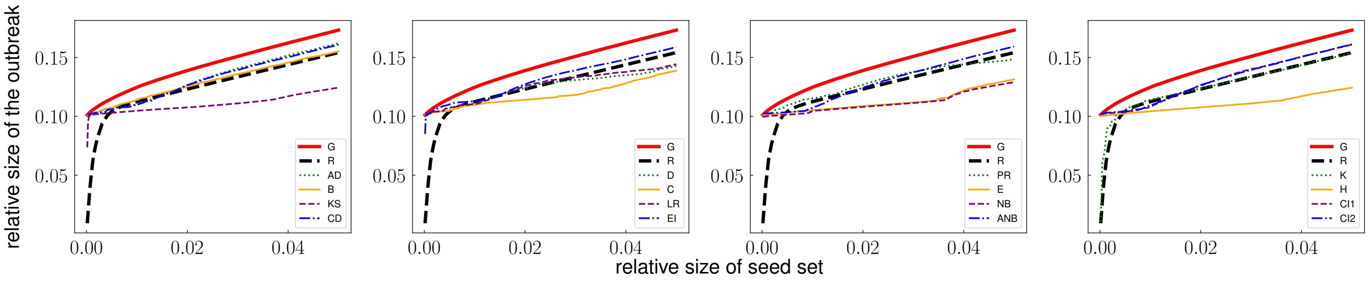

Figure 569: JDK - p=2.0p_c

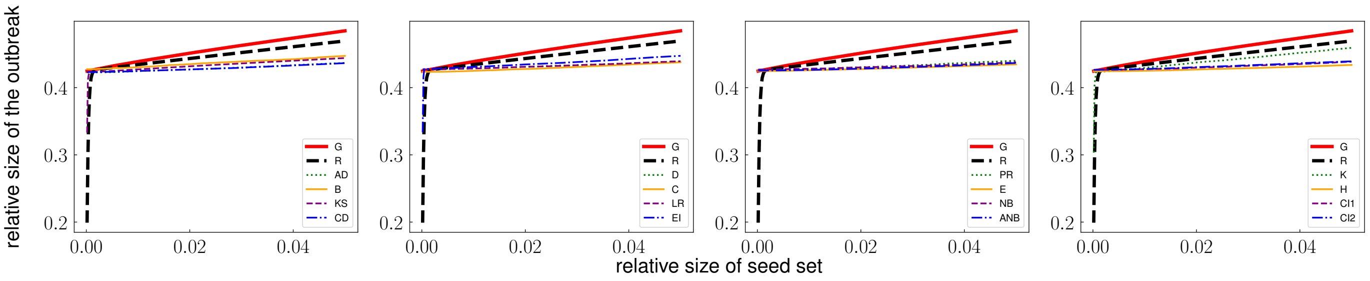

Figure 570: William - p=2.0p_c

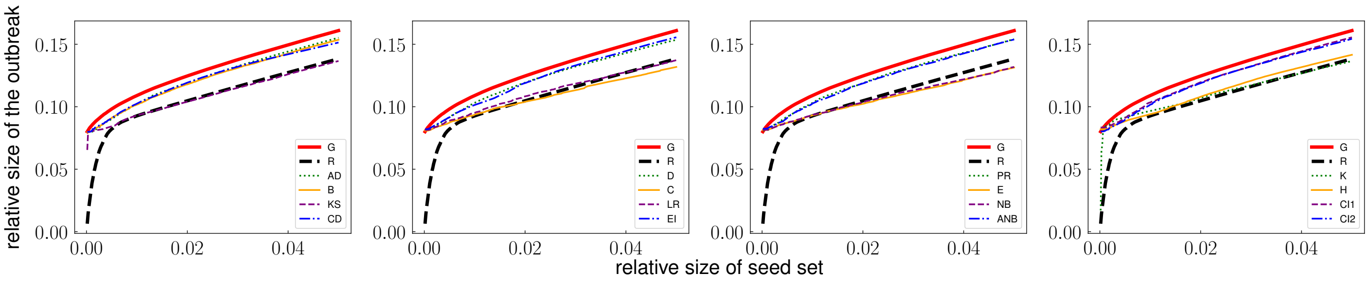

Figure 571: AS Oregon - p=2.0p_c

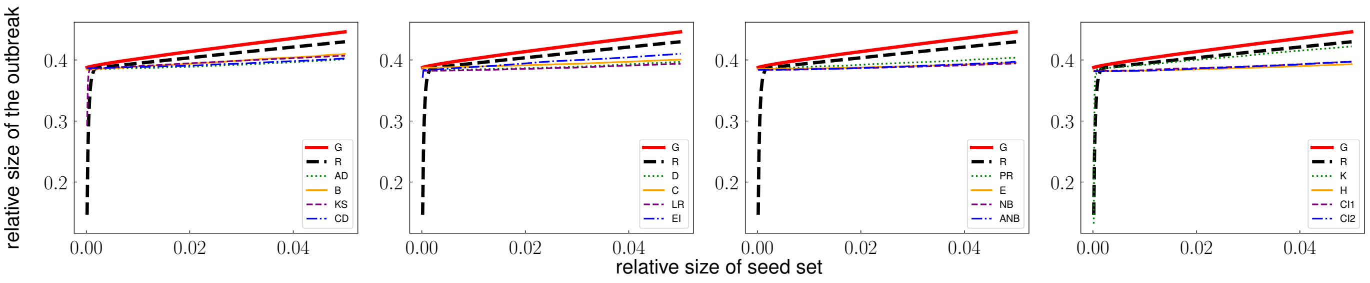

Figure 572: UChicago - p=2.0p_c

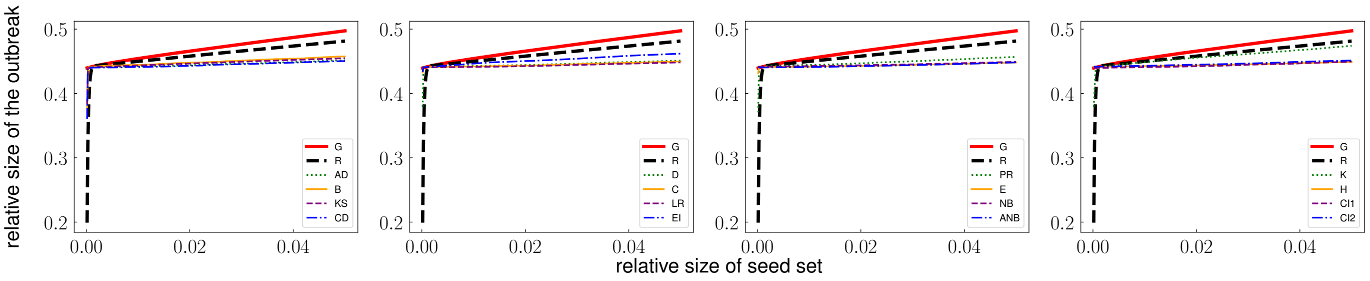

Figure 573: Princeton - p=2.0p_c

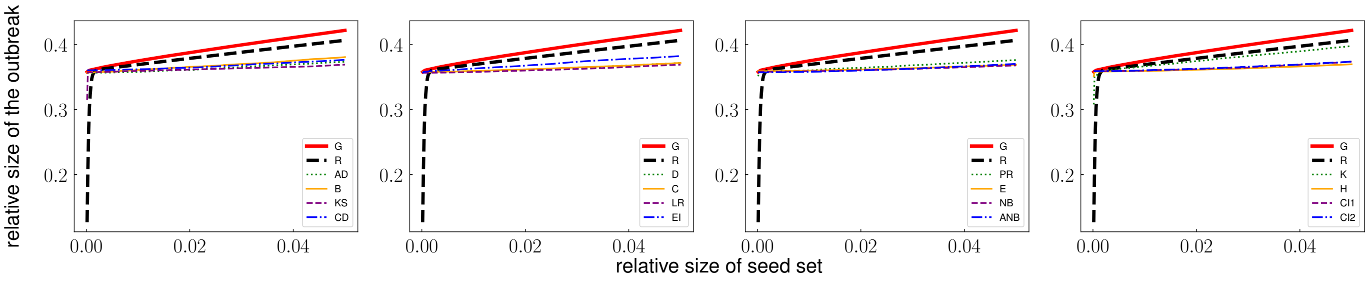

Figure 574: Carnegie - p=2.0p_c

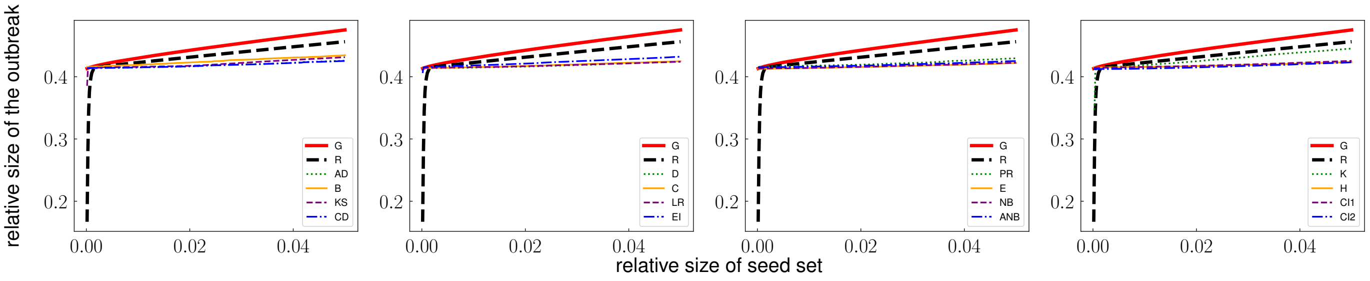

Figure 575: Tufts - p=2.0p_c

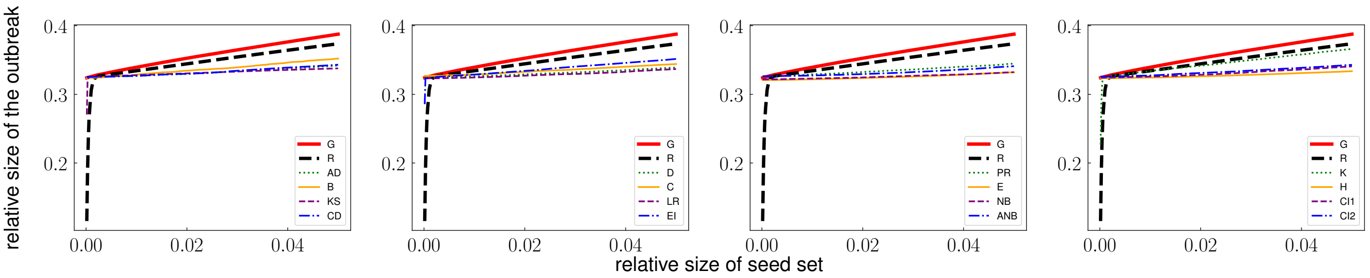

Figure 576: UC - p=2.0p_c

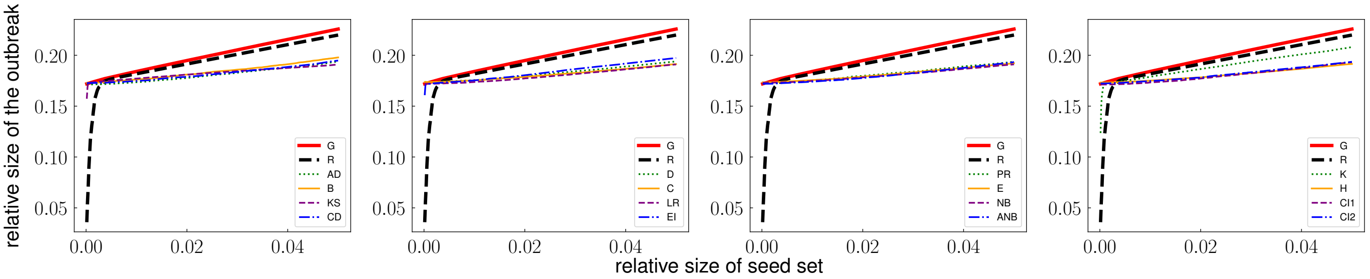

Figure 577: Wikipedia elections - p=2.0p_c

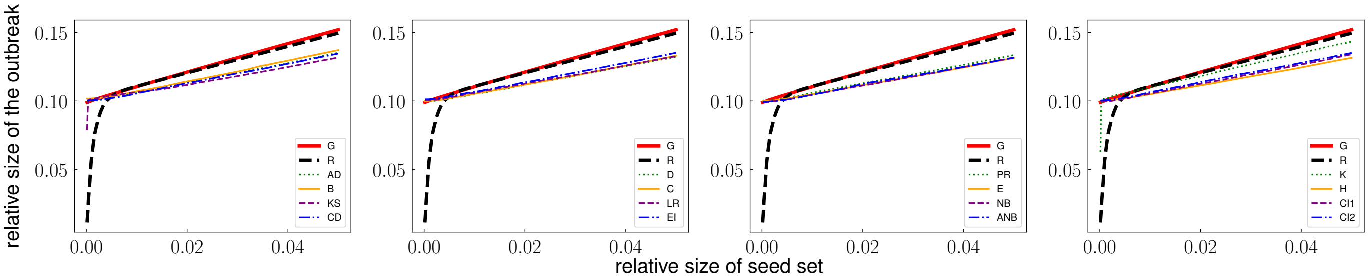

Figure 578: English - p=2.0p_c

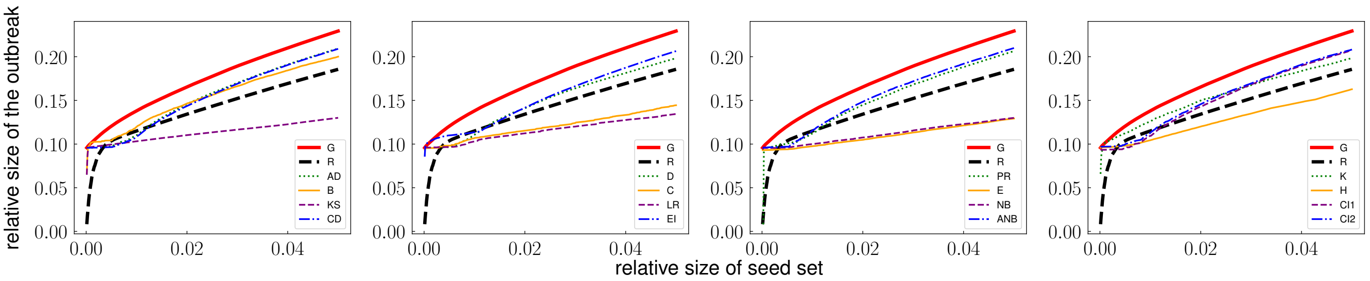

Figure 579: Gnutella, Aug. 9, 2002 - p=2.0p$_c$

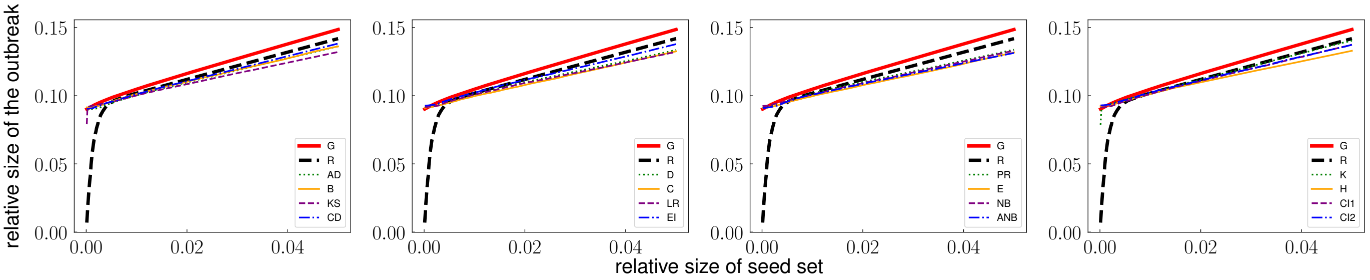

Figure 580: French - p=2.0p$_c$

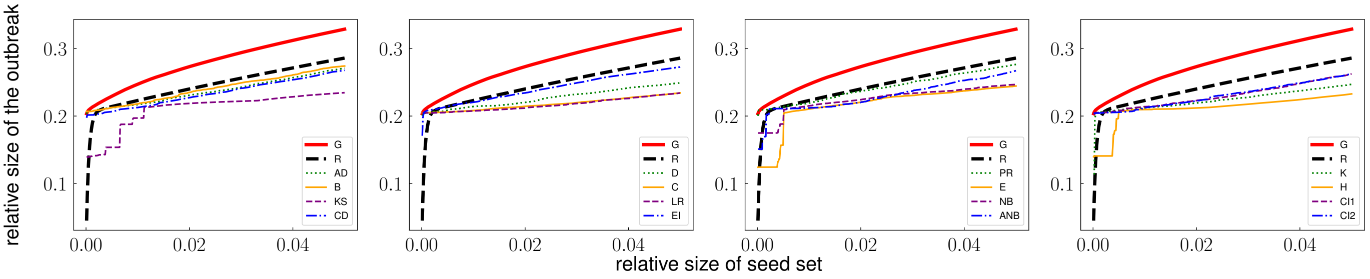

Figure 581: Hep-Th, 1993-2003 - p=2.0p$_c$

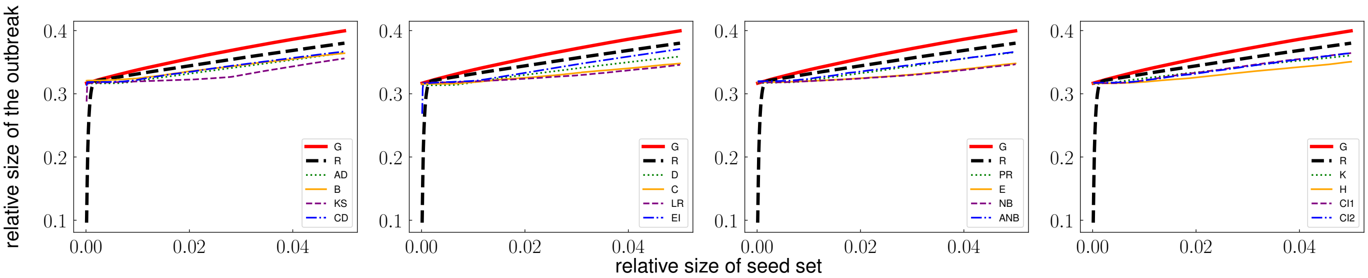

Figure 582: Gnutella, Aug. 6, 2002 - p=2.0p_c

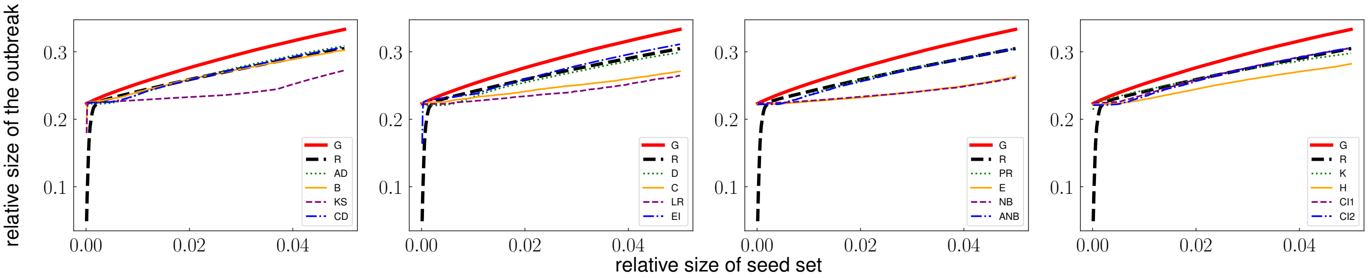

Figure 583: Gnutella, Aug. 5, 2002 - p=2.0p_c

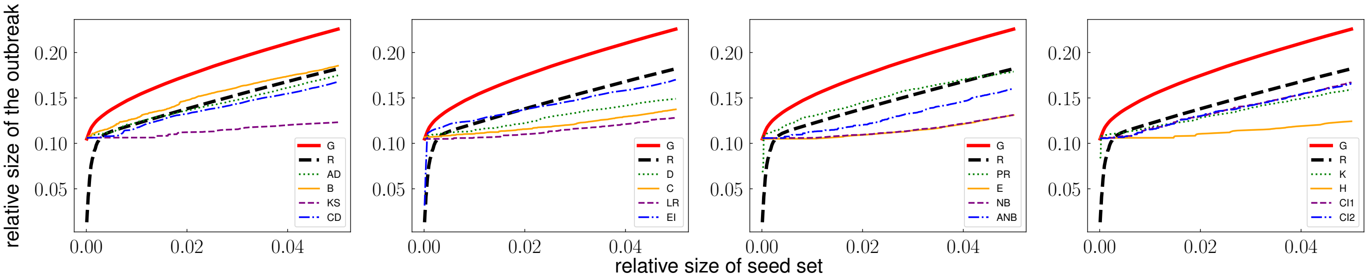

Figure 584: PGP - p=2.0p_c

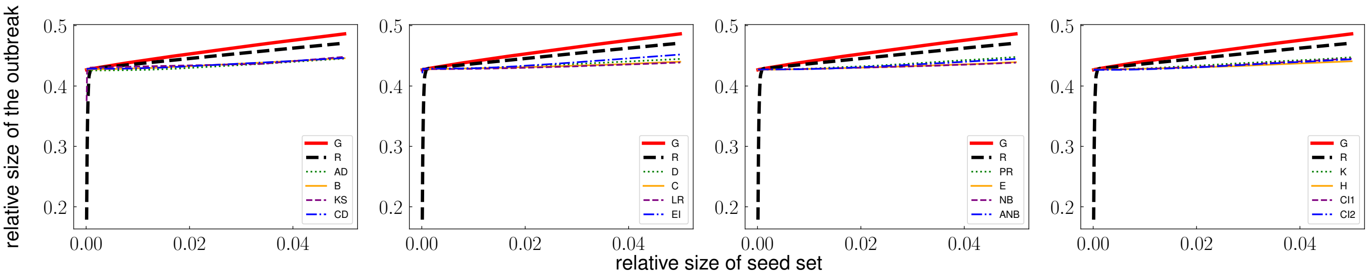

Figure 585: Gnutella, Aug. 4, 2002 - p=2.0p_c

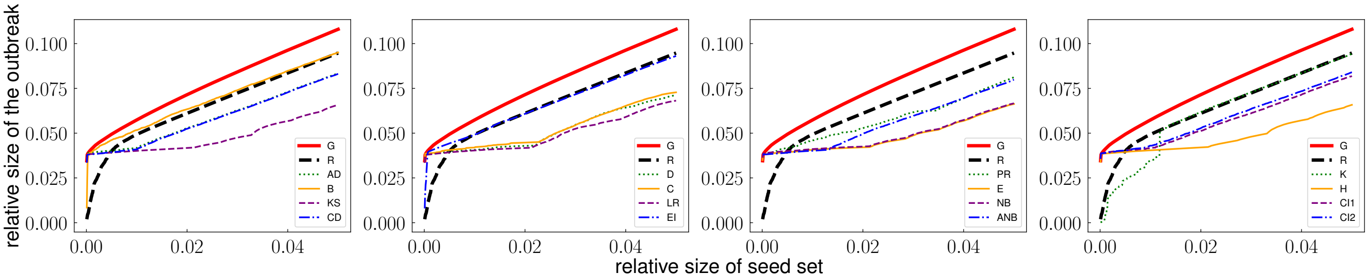

Figure 586: Hep-Ph, 1993-2003 - p=2.0p_c

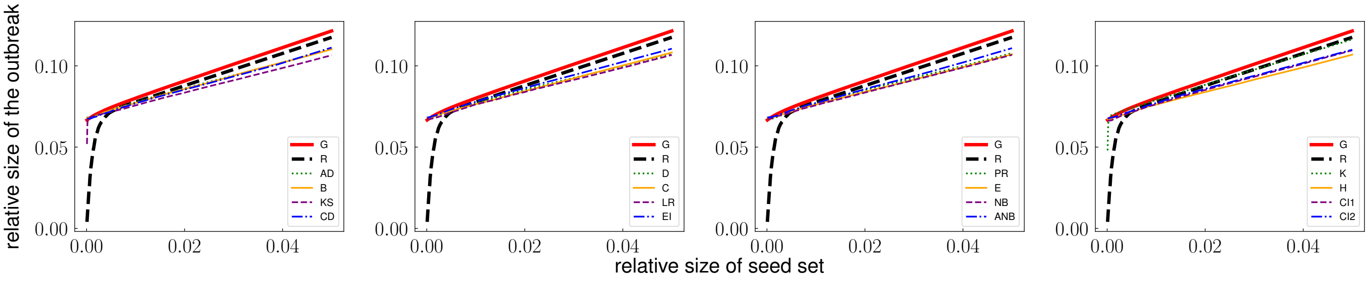

Figure 587: Spanish 1 - p=2.0p_c

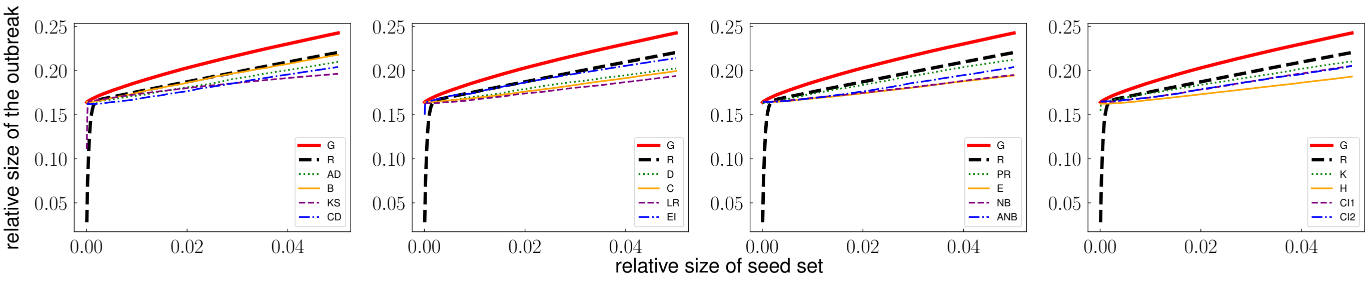

Figure 588: DBLP, citations - p=2.0p_c

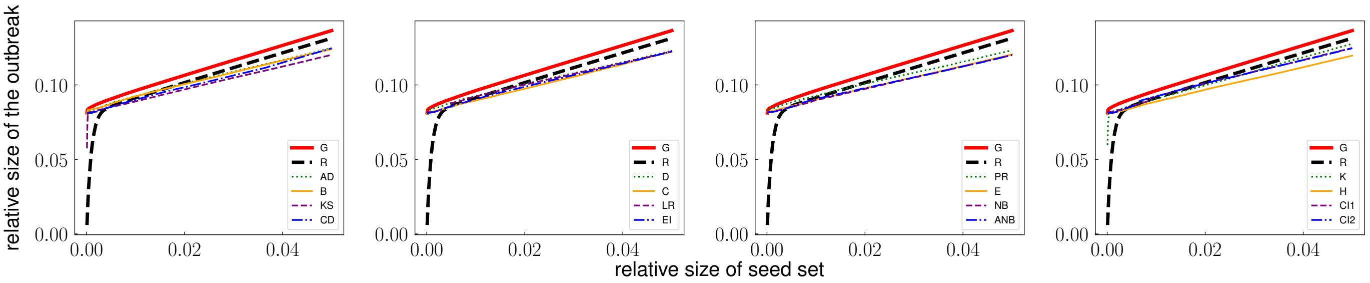

Figure 589: Spanish 2 - p=2.0p_c

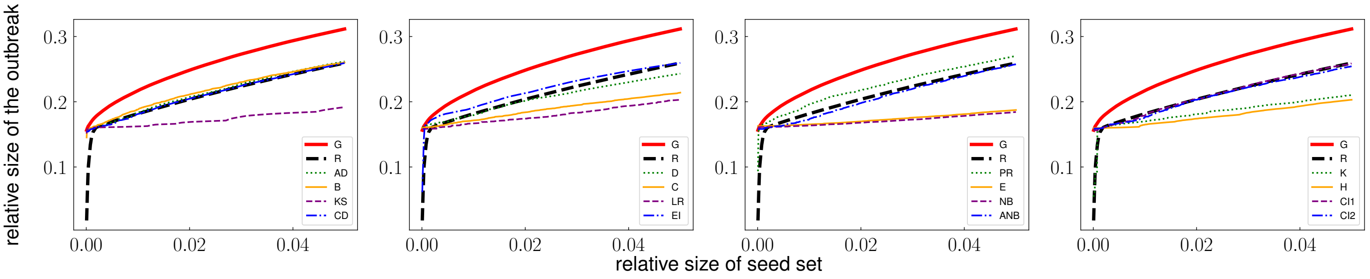

Figure 590: Cond-Mat, 1995-1999 - p=2.0p_c

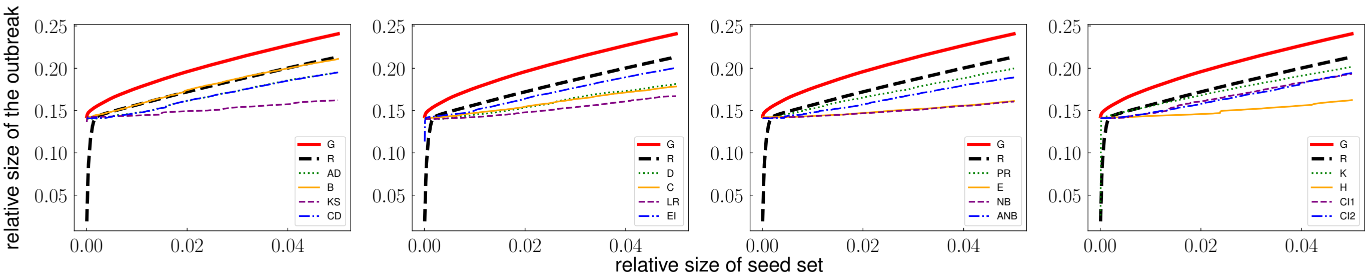

Figure 591: Astrophysics - p=2.0p_c

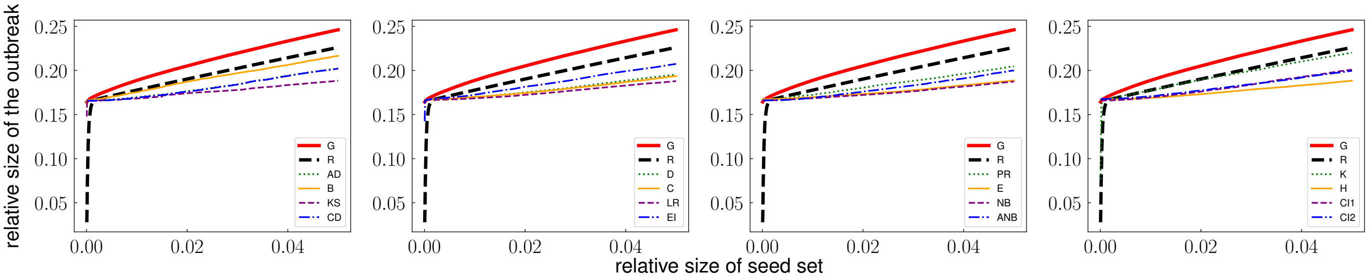

Figure 592: AstroPhys, 1993-2003 - p=2.0p_c

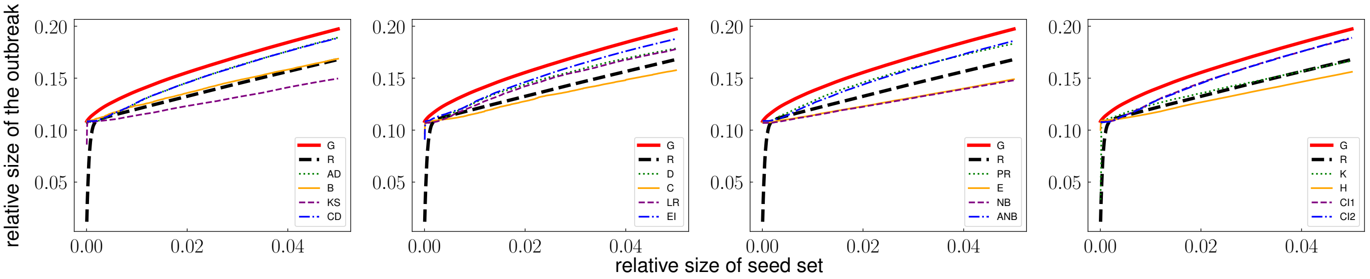

Figure 593: Marvel - p=2.0p_c

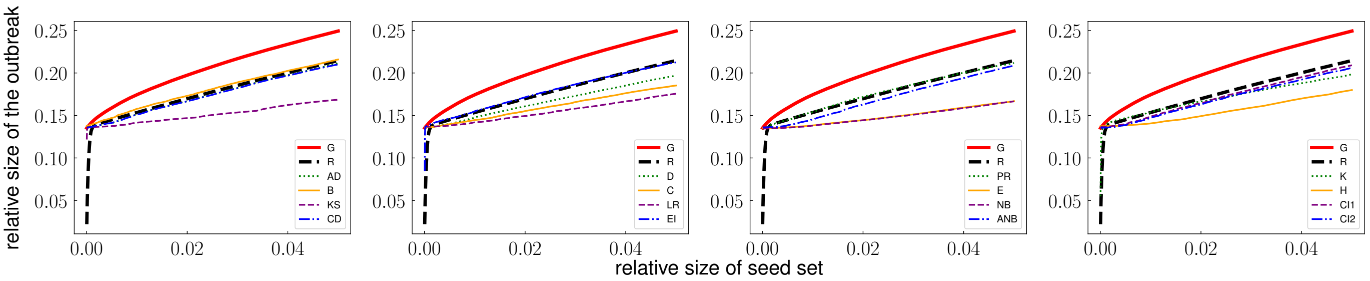

Figure 594: Cond-Mat, 1993-2003 - p=2.0p$_c$

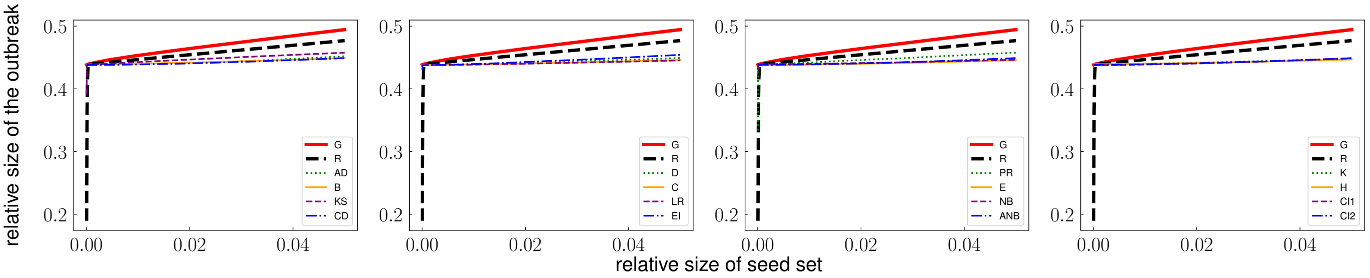

Figure 595: Gnutella, Aug. 25, 2002 - p=2.0p$_c$

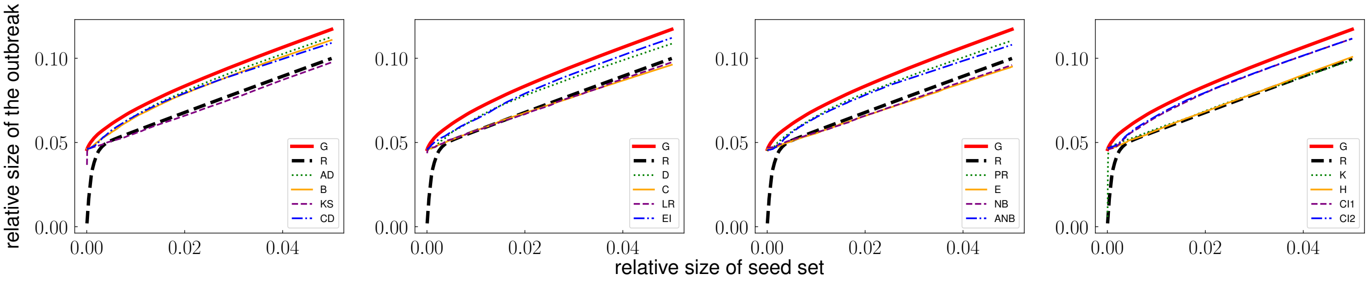

Figure 596: Internet - p=2.0p$_c$

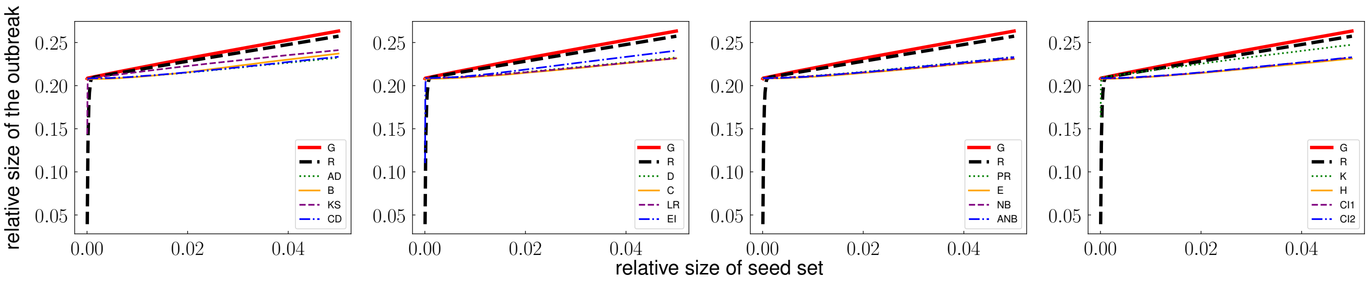

Figure 597: Thesaurus - p=2.0p_c

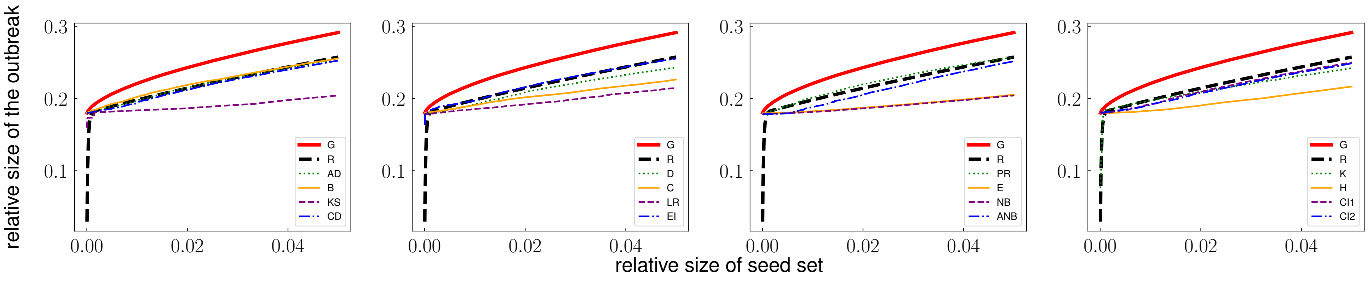

Figure 598: Cora - p=2.0p_c

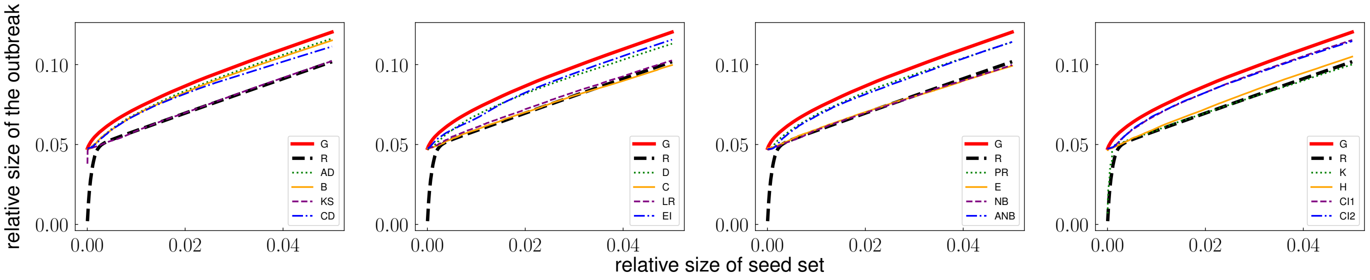

Figure 599: AS Caida - p=2.0p_c

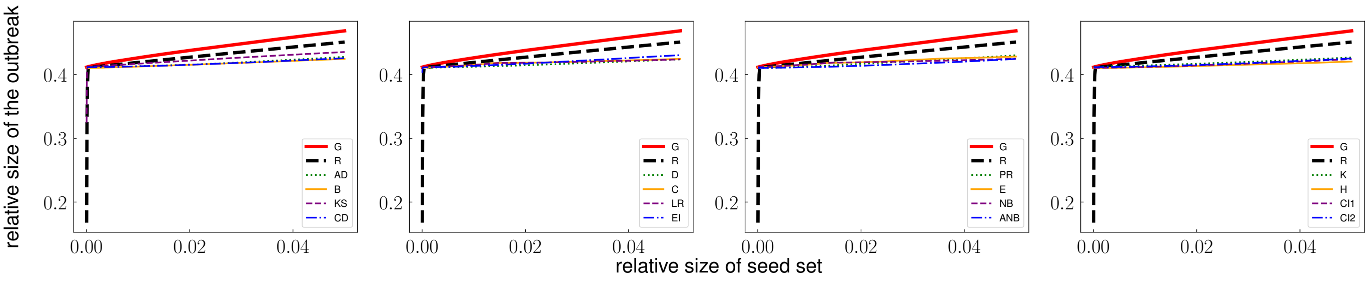

Figure 600: Gnutella, Aug. 24, 2002 - p=2.0p_c

References

[1] L. A. Adamic and N. Glance, "The political blogosphere and the 2004 us election: divided they blog," in *Proceedings of the 3rd international workshop on Link discovery*, pp. 36–43, ACM, 2005.

[2] M. Girvan and M. E. Newman, "Community structure in social and biological networks," *Proceedings of the National Academy of Sciences*, vol. 99, no. 12, pp. 7821–7826, 2002.

[3] R. Milo, S. Itzkovitz, N. Kashtan, R. Levitt, S. Shen-Orr, I. Ayzenshtat, M. Sheffer, and U. Alon, "Superfamilies of evolved and designed networks," *Science*, vol. 303, no. 5663, pp. 1538–1542, 2004.

[4] J. Fournet and A. Barrat, "Contact patterns among high school students," *PloS one*, vol. 9, no. 9, p. e107878, 2014.

[5] R. Ulanowicz, C. Bondavalli, and M. Egnotovich, "Network analysis of trophic dynamics in south florida ecosystem, fy 97: The florida bay ecosystem," *Annual Report to the United States Geological Service Biological Resources Division Ref. No.[UMCES] CBL*, pp. 98–123, 1998.

[6] J. Kunegis, "KONECT – The Koblenz Network Collection," in *Proc. Int. Conf. on World Wide Web Companion*, pp. 1343–1350, 2013.

[7] R. Michalski, S. Palus, and P. Kazienko, "Matching organizational structure and social network extracted from email communication," in *Lecture Notes in Business Information Processing*, vol. 87, pp. 197–206, Springer Berlin Heidelberg, 2011.

[8] N. D. Martinez, "Artifacts or attributes? effects of resolution on the little rock lake food web," *Ecological Monographs*, pp. 367–392, 1991.

[9] P. M. Gleiser and L. Danon, "Community structure in jazz," *Advances in complex systems*, vol. 6, no. 04, pp. 565–573, 2003.

[10] D. J. Watts and S. H. Strogatz, "Collective dynamics of small-worldnetworks," *nature*, vol. 393, no. 6684, pp. 440–442, 1998.

[11] M. E. Newman, "Finding community structure in networks using the eigenvectors of matrices," *Physical review E*, vol. 74, no. 3, p. 036104, 2006.

[12] L. Isella, J. Stehlé, A. Barrat, C. Cattuto, J.-F. Pinton, and W. Van den Broeck, "What's in a crowd? analysis of face-to-face behavioral networks," *Journal of theoretical biology*, vol. 271, no. 1, pp. 166–180, 2011.

[13] V. Colizza, R. Pastor-Satorras, and A. Vespignani, "Reaction–diffusion processes and metapopulation models in heterogeneous networks," *Nature Physics*, vol. 3, no. 4, pp. 276–282, 2007.

[14] R. Milo, S. Shen-Orr, S. Itzkovitz, N. Kashtan, D. Chklovskii, and U. Alon, “Network motifs: simple building blocks of complex networks,” *Science*, vol. 298, no. 5594, pp. 824–827, 2002.

[15] A. L. Traud, P. J. Mucha, and M. A. Porter, “Social structure of Facebook networks,” *Phys. A*, vol. 391, pp. 4165–4180, Aug 2012.

[16] A. L. Traud, E. D. Kelsic, P. J. Mucha, and M. A. Porter, “Comparing community structure to characteristics in online collegiate social networks,” *SIAM Rev.*, vol. 53, no. 3, pp. 526–543, 2011.

[17] R. A. Rossi and N. K. Ahmed, “The network data repository with interactive graph analytics and visualization,” in *Proceedings of the Twenty-Ninth AAAI Conference on Artificial Intelligence*, 2015.

[18] M. Helmstaedter, K. L. Briggman, S. C. Turaga, V. Jain, H. S. Seung, and W. Denk, “Connectomic reconstruction of the inner plexiform layer in the mouse retina,” *Nature*, vol. 500, no. 7461, p. 168, 2013.

[19] A. Clauset, E. Tucker, and M. Sainz, “The colorado index of complex networks,” 2016.

[20] R. Guimera, L. Danon, A. Diaz-Guilera, F. Giralt, and A. Arenas, “Self-similar community structure in a network of human interactions,” *Physical review E*, vol. 68, no. 6, p. 065103, 2003.

[21] T. Opsahl and P. Panzarasa, “Clustering in weighted networks,” *Social networks*, vol. 31, no. 2, pp. 155–163, 2009.

[22] D. Bu, Y. Zhao, L. Cai, H. Xue, X. Zhu, H. Lu, J. Zhang, S. Sun, L. Ling, N. Zhang, *et al.*, “Topological structure analysis of the protein–protein interaction network in budding yeast,” *Nucleic acids research*, vol. 31, no. 9, pp. 2443–2450, 2003.

[23] J. Moody, “Peer influence groups: identifying dense clusters in large networks,” *Social Networks*, vol. 23, no. 4, pp. 261–283, 2001.

[24] T. Opsahl, F. Agneessens, and J. Skvoretz, “Node centrality in weighted networks: Generalizing degree and shortest paths,” *Social Networks*, vol. 32, no. 3, pp. 245–251, 2010.

[25] S. Kumar, F. Spezzano, V. Subrahmanian, and C. Faloutsos, “Edge weight prediction in weighted signed networks,” in *Data Mining (ICDM), 2016 IEEE 16th International Conference on*, pp. 221–230, IEEE, 2016.

[26] S. Kumar, B. Hooi, D. Makhija, M. Kumar, C. Faloutsos, and V. Subrahmanian, “Rev2: Fraudulent user prediction in rating platforms,” in *Proceedings of the Eleventh ACM International Conference on Web Search and Data Mining*, pp. 333–341, ACM, 2018.

[27] J. Leskovec and A. Krevl, "SNAP Datasets: Stanford large network dataset collection." `http://snap.stanford.edu/data`, June 2014.

[28] J. Leskovec, J. Kleinberg, and C. Faloutsos, "Graph evolution: Densification and shrinking diameters," *ACM Transactions on Knowledge Discovery from Data (TKDD)*, vol. 1, no. 1, p. 2, 2007.

[29] F. Radicchi, "Who is the best player ever? a complex network analysis of the history of professional tennis," *PloS one*, vol. 6, no. 2, p. e17249, 2011.

[30] M. E. Newman, "The structure of scientific collaboration networks," *Proceedings of the National Academy of Sciences*, vol. 98, no. 2, pp. 404–409, 2001.

[31] G. Joshi-Tope, M. Gillespie, I. Vastrik, P. D'Eustachio, E. Schmidt, B. de Bono, B. Jassal, G. Gopinath, G. Wu, L. Matthews, *et al.*, "Reactome: a knowledgebase of biological pathways," *Nucleic acids research*, vol. 33, no. suppl 1, pp. D428–D432, 2005.

[32] L. Šubelj and M. Bajec, "Software systems through complex networks science: Review, analysis and applications," in *Proceedings of the First International Workshop on Software Mining*, pp. 9–16, ACM, 2012.

[33] M. Ripeanu, I. Foster, and A. Iamnitchi, "Mapping the gnutella network: Properties of large-scale peer-to-peer systems and implications for system design," *arXiv preprint cs/0209028*, 2002.

[34] J. Leskovec, J. Kleinberg, and C. Faloutsos, "Graphs over time: densification laws, shrinking diameters and possible explanations," in *Proceedings of the eleventh ACM SIGKDD international conference on Knowledge discovery in data mining*, pp. 177–187, ACM, 2005.

[35] J. Leskovec, D. Huttenlocher, and J. Kleinberg, "Signed networks in social media," in *Proceedings of the SIGCHI conference on human factors in computing systems*, pp. 1361–1370, ACM, 2010.

[36] J. Leskovec, D. Huttenlocher, and J. Kleinberg, "Predicting positive and negative links in online social networks," in *Proceedings of the 19th international conference on World wide web*, pp. 641–650, ACM, 2010.

[37] M. Boguñá, R. Pastor-Satorras, A. Díaz-Guilera, and A. Arenas, "Models of social networks based on social distance attachment," *Physical Review E*, vol. 70, no. 5, p. 056122, 2004.

[38] M. Ley, "The dblp computer science bibliography: Evolution, research issues, perspectives," in *String Processing and Information Retrieval*, pp. 1–10, Springer, 2002.

[39] R. Alberich, J. Miro-Julia, and F. Rosselló, "Marvel universe looks almost like a real social network," *arXiv preprint cond-mat/0202174*, 2002.

[40] G. R. Kiss, C. Armstrong, R. Milroy, and J. Piper, "An associative thesaurus of english and its computer analysis," *The computer and literary studies*, pp. 153–165, 1973.

[41] L. Šubelj and M. Bajec, "Model of complex networks based on citation dynamics," in *Proceedings of the 22nd international conference on World Wide Web companion*, pp. 527–530, International World Wide Web Conferences Steering Committee, 2013.
