# Supplementary material for: Systematic comparison between methods for the detection of influential spreaders in complex networks
Source: Sci Rep. 2019 Oct 22;9:15095. doi: 10.1038/s41598-019-51209-6 (PMC6805897; doi:10.1038/s41598-019-51209-6)
Supplement: Supplementary file 2 — SM2 [file 41598_2019_51209_MOESM2_ESM.pdf]

1 T=0.05

1.1 Performance relative to greedy vs cumulative distribution

1.1.1 Subcritical Regime

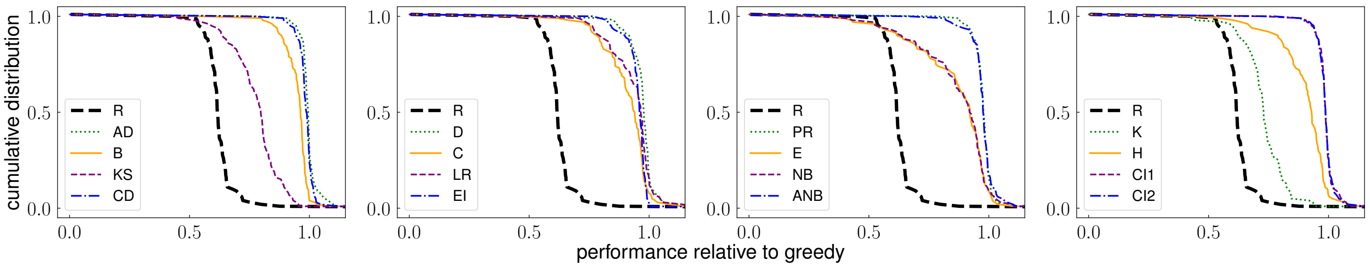

Figure 1: $p = 0.5p_c$

1.1.2 Critical Regime

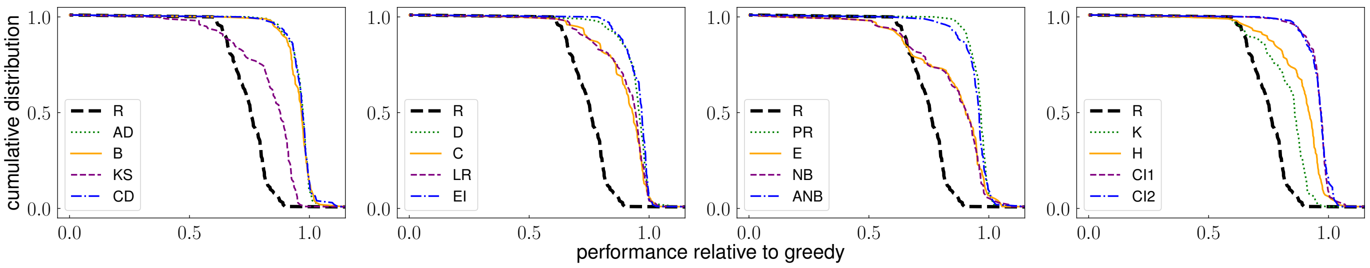

Figure 2: $p = p_c$

1.1.3 Supercritical Regime

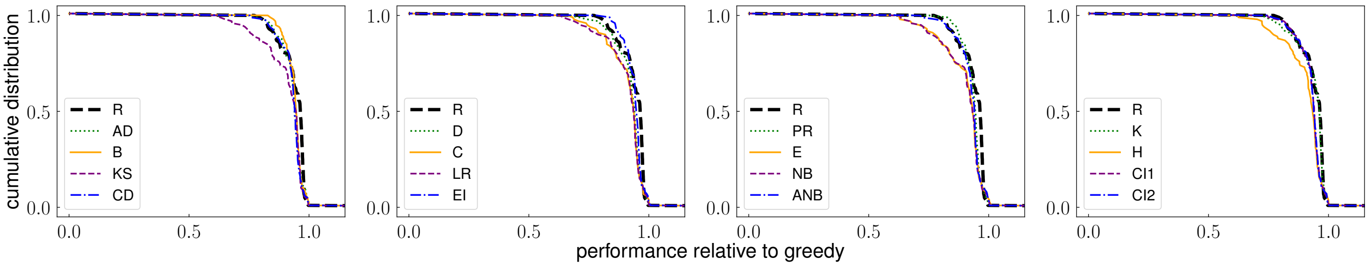

Figure 3: $p = 2p_c$

1.2 Precision relative to greedy vs cumulative distribution

1.2.1 Subcritical Regime

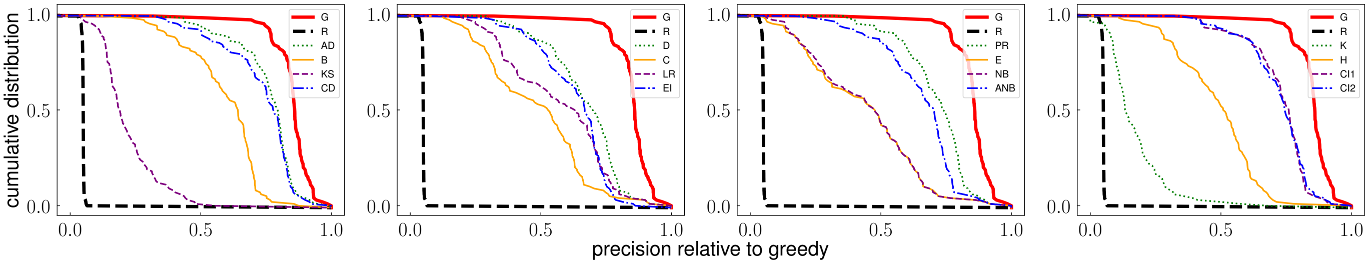

Figure 4: $p = 0.5p_c$

1.2.2 Critical Regime

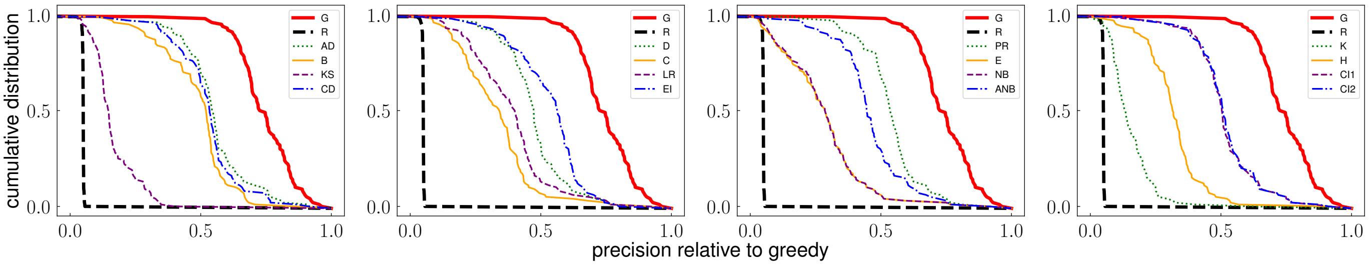

Figure 5: $p = p_c$

1.2.3 Supercritical Regime

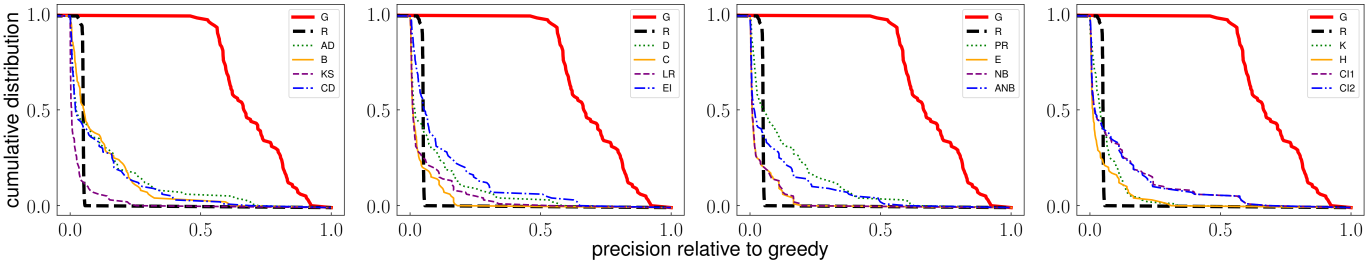

Figure 6: $p = 2p_c$

	Subcritical		Critical		Supercritical	
Method	$\langle g_m \rangle$	$\langle r_m \rangle$	$\langle g_m \rangle$	$\langle r_m \rangle$	$\langle g_m \rangle$	$\langle r_m \rangle$
G	1.00 ± 0.00	0.85 ± 0.01	1.00 ± 0.00	0.74 ± 0.01	1.00 ± 0.00	0.69 ± 0.01
R	0.62 ± 0.01	0.05 ± 0.01	0.75 ± 0.01	0.05 ± 0.01	0.94 ± 0.01	0.05 ± 0.01
D	0.97 ± 0.01	0.67 ± 0.01	0.94 ± 0.01	0.47 ± 0.01	0.92 ± 0.01	0.08 ± 0.01
AD	0.99 ± 0.01	0.75 ± 0.01	0.96 ± 0.01	0.55 ± 0.01	0.93 ± 0.01	0.12 ± 0.02
B	0.95 ± 0.01	0.59 ± 0.01	0.95 ± 0.01	0.48 ± 0.01	0.94 ± 0.01	0.11 ± 0.01
C	0.91 ± 0.01	0.48 ± 0.02	0.90 ± 0.01	0.33 ± 0.01	0.91 ± 0.01	0.03 ± 0.01
E	0.86 ± 0.02	0.44 ± 0.02	0.85 ± 0.02	0.28 ± 0.02	0.90 ± 0.01	0.03 ± 0.01
K	0.73 ± 0.01	0.17 ± 0.01	0.83 ± 0.01	0.15 ± 0.01	0.93 ± 0.01	0.06 ± 0.01
PR	0.97 ± 0.01	0.71 ± 0.01	0.96 ± 0.01	0.53 ± 0.01	0.93 ± 0.01	0.12 ± 0.02
NB	0.86 ± 0.02	0.44 ± 0.02	0.85 ± 0.02	0.28 ± 0.02	0.90 ± 0.01	0.03 ± 0.01
ANB	0.97 ± 0.01	0.65 ± 0.01	0.94 ± 0.01	0.46 ± 0.01	0.92 ± 0.01	0.09 ± 0.01
KS	0.77 ± 0.01	0.22 ± 0.01	0.83 ± 0.01	0.16 ± 0.01	0.90 ± 0.01	0.02 ± 0.01
LR	0.93 ± 0.01	0.57 ± 0.02	0.90 ± 0.01	0.38 ± 0.02	0.91 ± 0.01	0.05 ± 0.01
H	0.90 ± 0.01	0.48 ± 0.02	0.88 ± 0.01	0.32 ± 0.01	0.90 ± 0.01	0.04 ± 0.01
EI	0.94 ± 0.01	0.63 ± 0.01	0.95 ± 0.01	0.53 ± 0.01	0.94 ± 0.01	0.12 ± 0.02
CD	0.98 ± 0.01	0.73 ± 0.01	0.96 ± 0.01	0.52 ± 0.01	0.93 ± 0.01	0.10 ± 0.01
CI1	0.99 ± 0.01	0.72 ± 0.01	0.96 ± 0.01	0.51 ± 0.01	0.93 ± 0.01	0.10 ± 0.02
CI2	0.99 ± 0.01	0.72 ± 0.01	0.96 ± 0.01	0.51 ± 0.01	0.93 ± 0.01	0.10 ± 0.02

Table 1: Performance and precision of methods for the identification of influential spreaders in real networks. Results are based on the systematic analysis of 100 real-world networks. For each network, we first evaluate the critical value of the spreading probability p_c for ICM dynamics. Then, we consider the analysis for three distinct phases of spreading: subcritical $p = p_c/2$, critical $p = p_c$, and supercritical $p = 2p_c$. Each row of the table corresponds to one method. For clarity, methods are identified by the same abbreviations as those defined in Table I of the main paper. Overall performance $\langle g_m \rangle$ is a metric of performance that relies on the size of the outbreak associated with the set of influential spreaders identified by the method compared to the typical outbreak obtained with the greedy algorithm. Overall precision $\langle r_m \rangle$ instead quantifies the overlap between the sets of spreaders identified by a method and those identified by the greedy algorithm. Error estimates correspond to standard errors of the mean associated with the numerical estimates of $\langle g_m \rangle$ and $\langle r_m \rangle$. The numerical values reported here are the same as those used in the visualization of Figure 4 of the main paper.

2 T=0.10

2.1 Performance relative to greedy vs cumulative distribution

2.1.1 Subcritical Regime

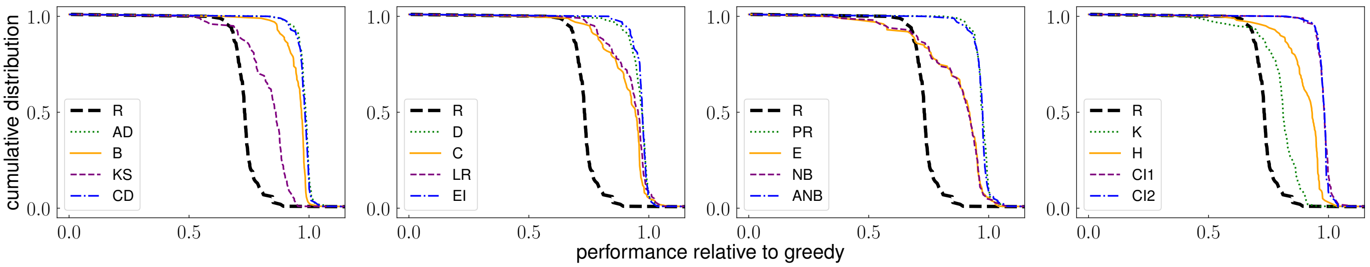

Figure 7: $p = 0.5p_c$

2.1.2 Critical Regime

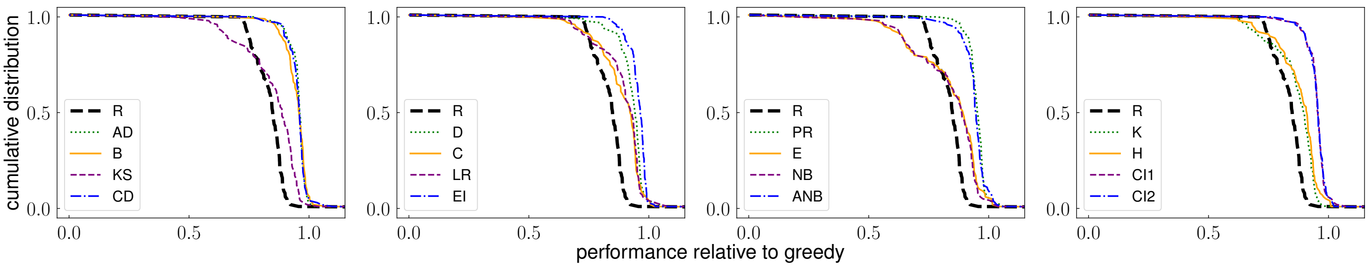

Figure 8: $p = p_c$

2.1.3 Supercritical Regime

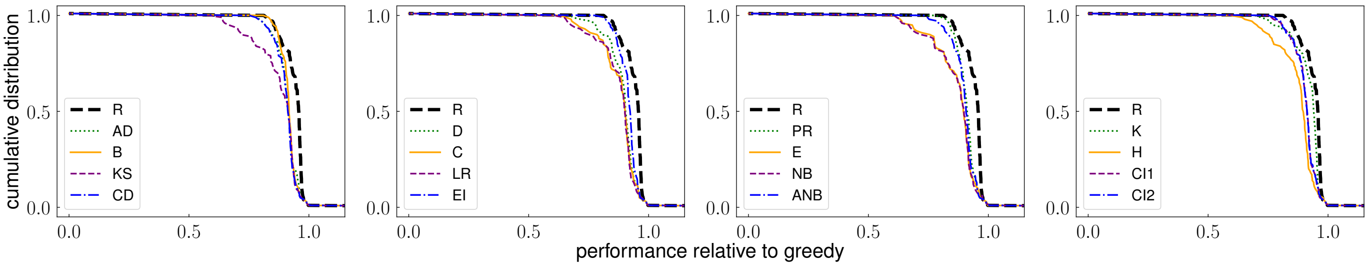

Figure 9: $p = 2p_c$

2.2 Precision relative to greedy vs cumulative distribution

2.2.1 Subcritical Regime

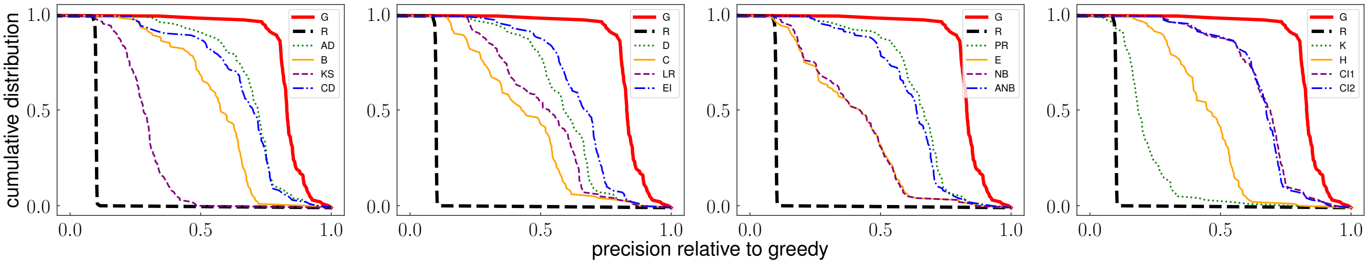

Figure 10: $p = 0.5p_c$

2.2.2 Critical Regime

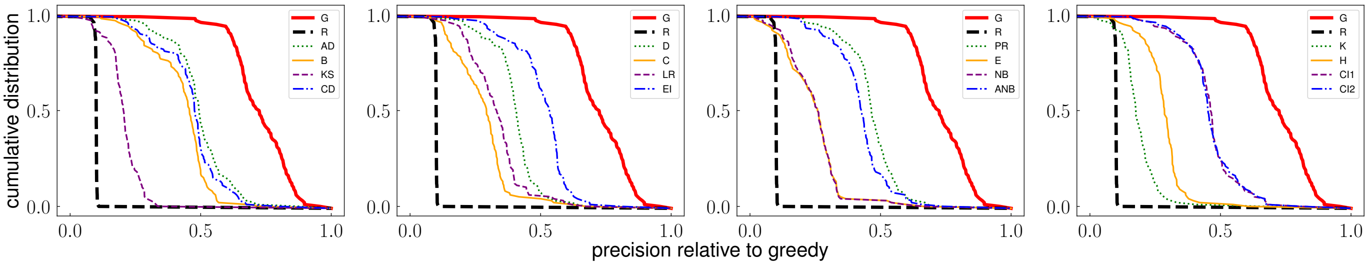

Figure 11: $p = p_c$

2.2.3 Supercritical Regime

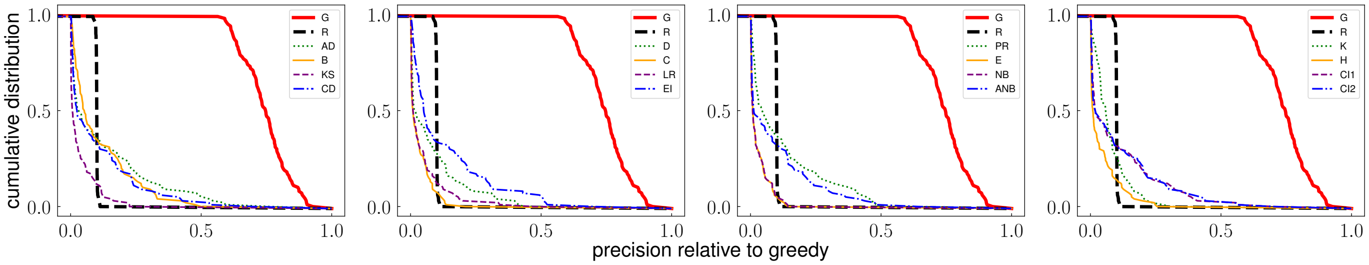

Figure 12: $p = 2p_c$

2.3 Overall performance vs overall precision

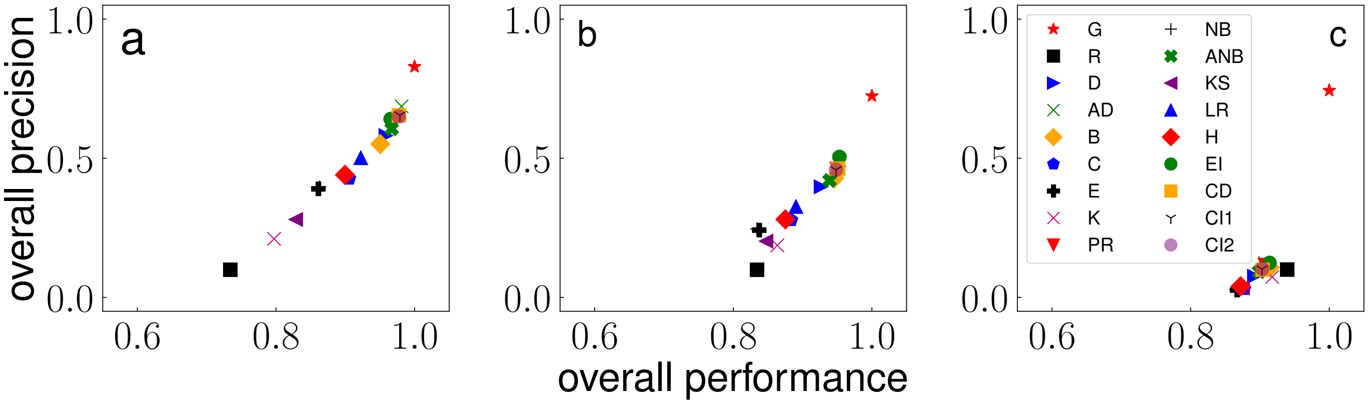

Figure 13: All three regimes for T=0.10

2.4 Pairwise precision values of methods

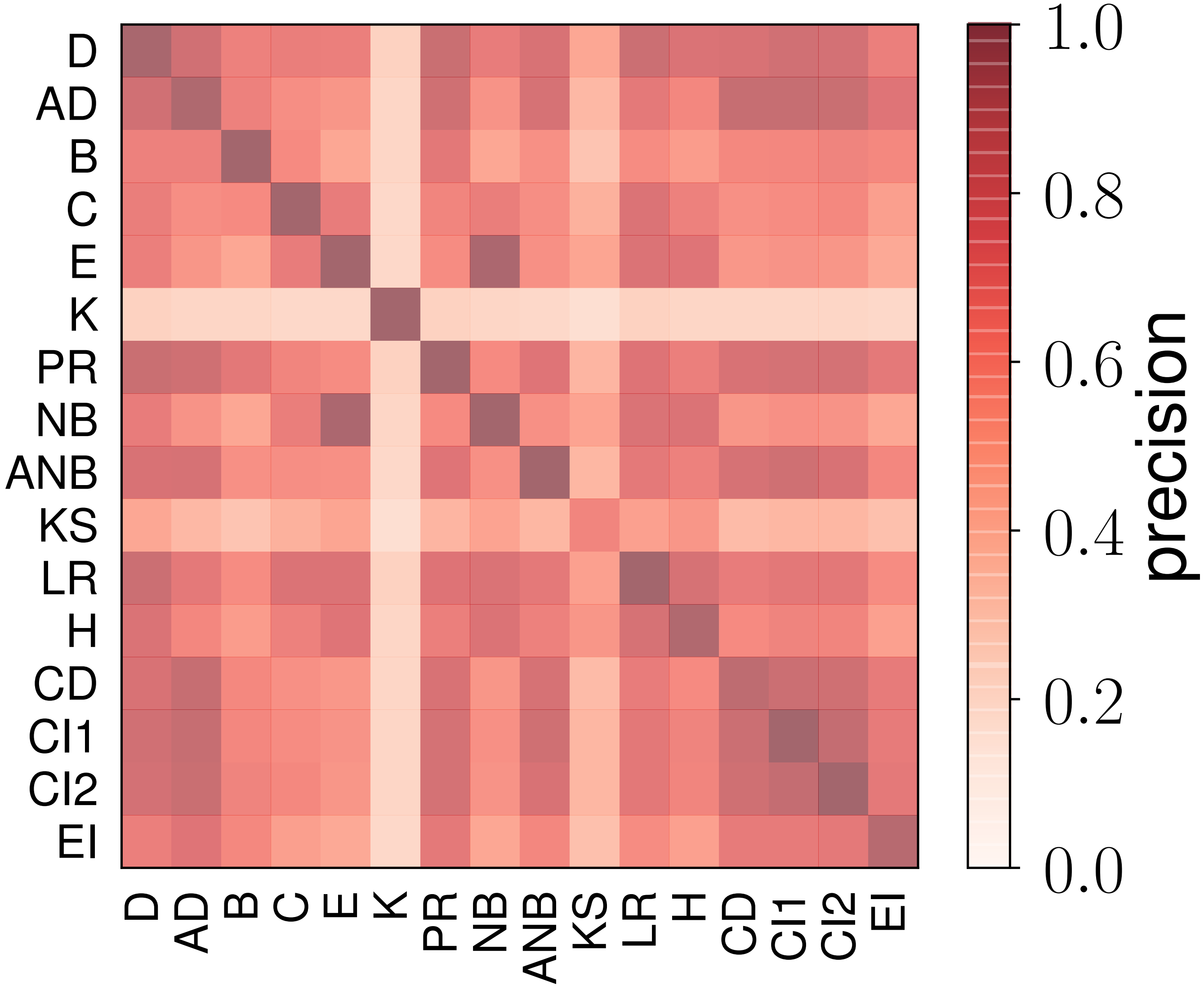

Figure 14: Precision of seed sets for T=0.10

3 Barabasi-Albert networks

In this analysis, we have used the Barabasi-Albert algorithm to generate the networks. The sizes of the networks has been set as 5000, 10000, and 20000, and 10 of each has been used. We observe there are sharp changes in the plots. We believe this is due to the fact that every network is generated with the same dynamics coming from the algorithm. Because of this, the characteristics of the networks are very similar, and their performance and precision values turn out to be very close.

3.1 Performance relative to greedy vs cumulative distribution

3.1.1 Subcritical Regime

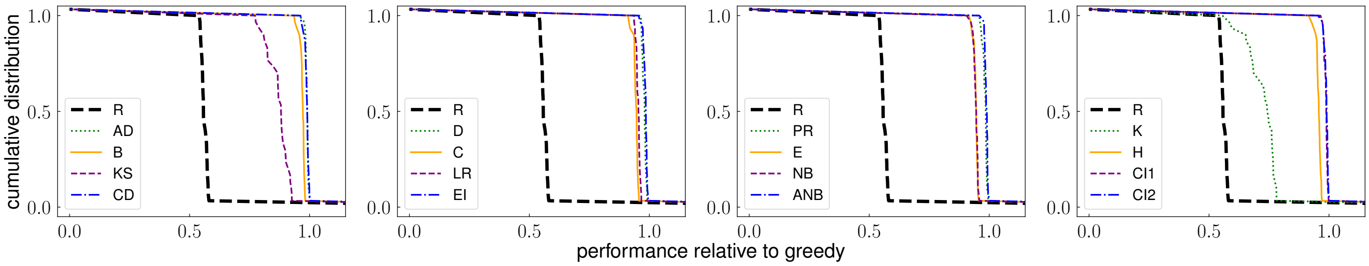

Figure 15: $p = 0.5p_c$

3.1.2 Critical Regime

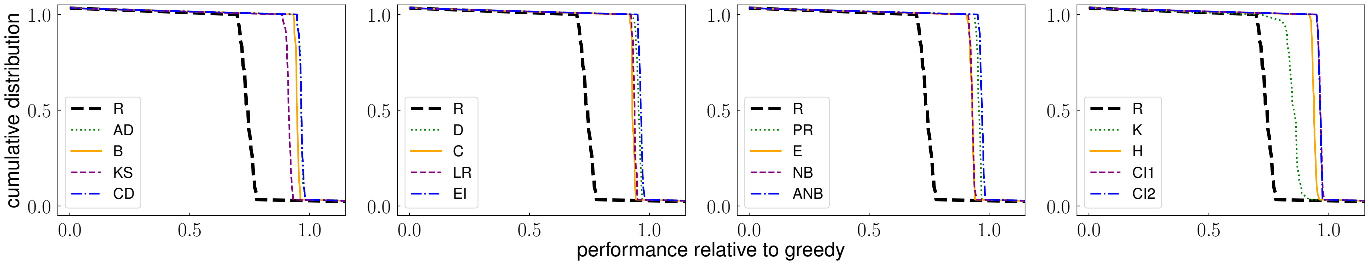

Figure 16: $p = p_c$

3.1.3 Supercritical Regime

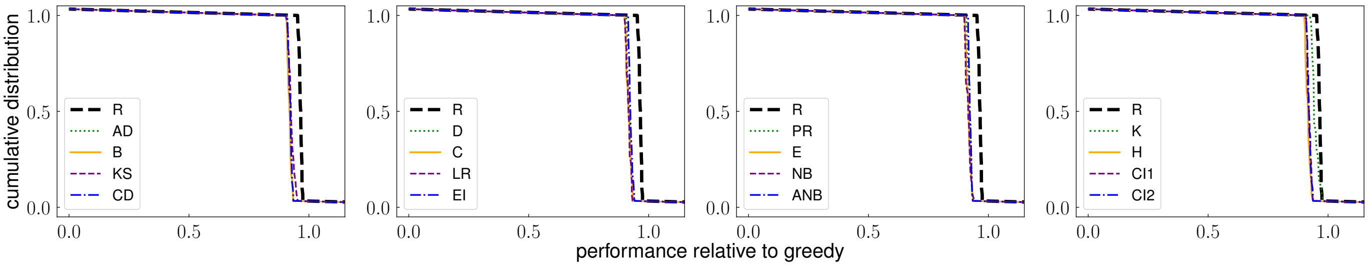

Figure 17: $p = 2p_c$

3.2 Precision relative to greedy vs cumulative distribution

3.2.1 Subcritical Regime

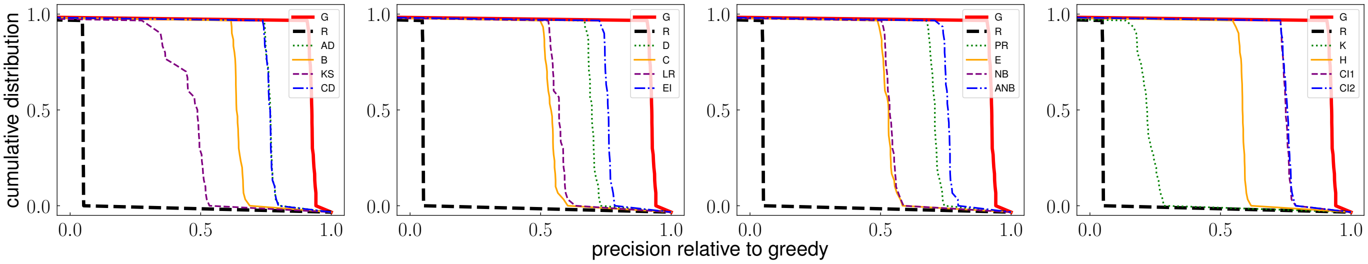

Figure 18: $p = 0.5p_c$

3.2.2 Critical Regime

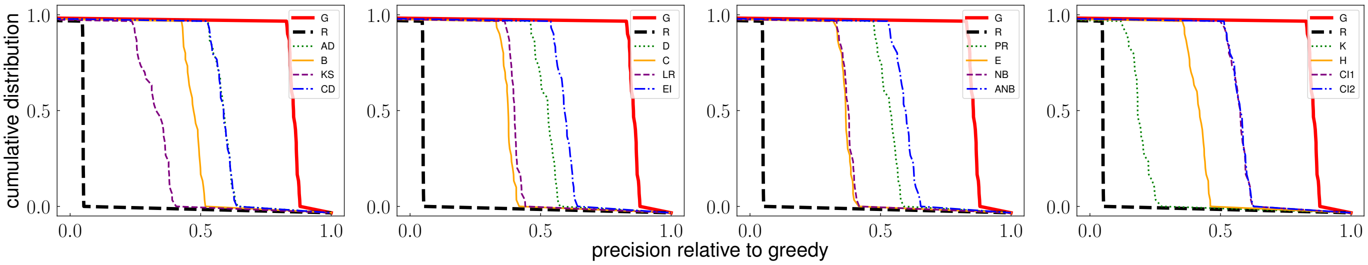

Figure 19: $p = p_c$

3.2.3 Supercritical Regime

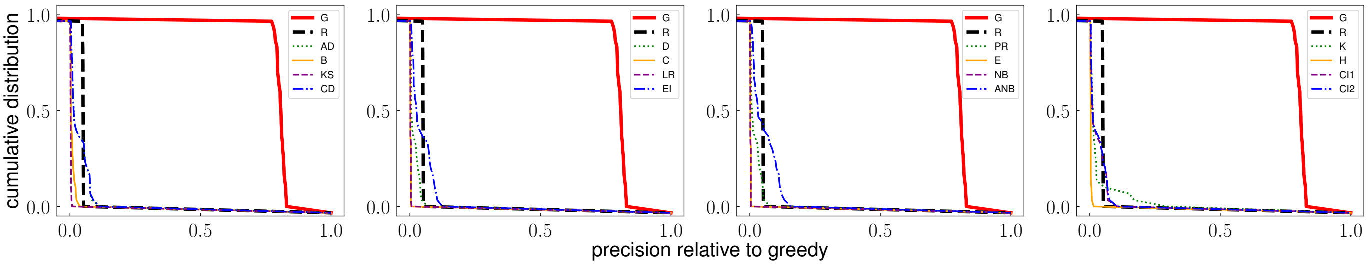

Figure 20: $p = 2p_c$

3.3 Overall performance vs overall precision

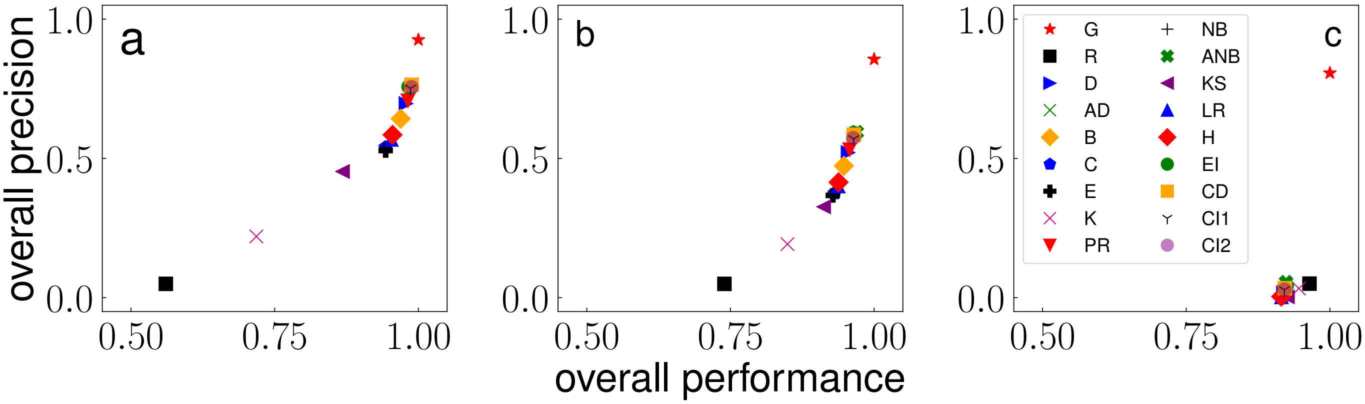

Figure 21: BA networks in all three regimes

3.4 Pairwise precision values of methods

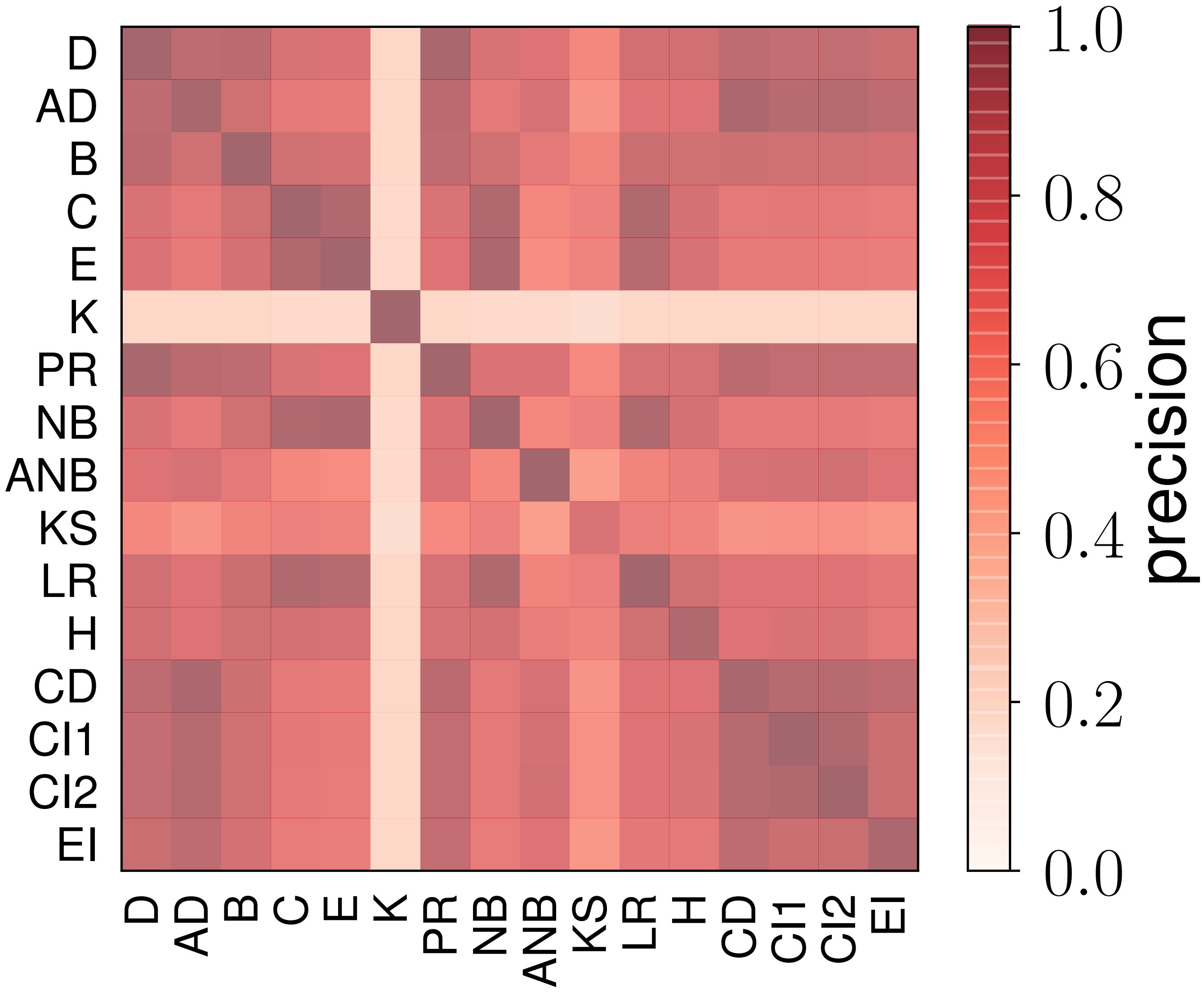

Figure 22: Precision of seed sets for BA networks

4 Analysis for Network Domains - T=0.05

4.1 Overall performance vs overall precision

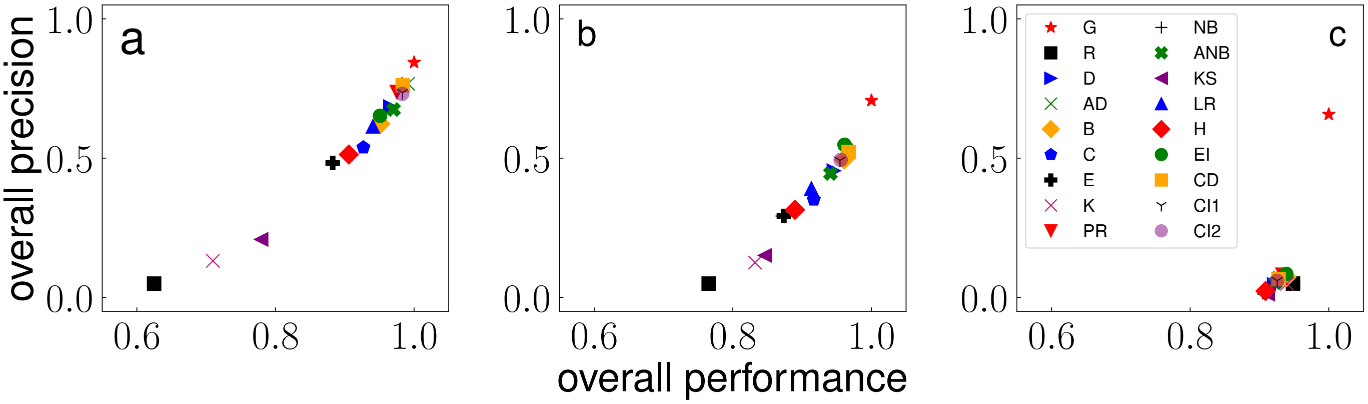

Figure 23: All three regimes for T=0.05 for social networks

	Subcritical		Critical		Supercritical	
Method	$\langle g_m \rangle$	$\langle r_m \rangle$	$\langle g_m \rangle$	$\langle r_m \rangle$	$\langle g_m \rangle$	$\langle r_m \rangle$
G	1.00 ± 0.00	0.84 ± 0.01	1.00 ± 0.00	0.71 ± 0.01	1.00 ± 0.00	0.66 ± 0.01
R	0.62 ± 0.01	0.05 ± 0.01	0.77 ± 0.01	0.05 ± 0.01	0.95 ± 0.01	0.05 ± 0.01
D	0.97 ± 0.01	0.68 ± 0.02	0.95 ± 0.01	0.45 ± 0.01	0.92 ± 0.01	0.05 ± 0.01
AD	0.99 ± 0.01	0.77 ± 0.01	0.96 ± 0.01	0.53 ± 0.01	0.93 ± 0.01	0.07 ± 0.02
B	0.95 ± 0.01	0.62 ± 0.02	0.96 ± 0.01	0.50 ± 0.01	0.94 ± 0.01	0.07 ± 0.01
C	0.93 ± 0.01	0.54 ± 0.02	0.92 ± 0.01	0.35 ± 0.02	0.91 ± 0.01	0.02 ± 0.01
E	0.88 ± 0.02	0.48 ± 0.02	0.87 ± 0.02	0.29 ± 0.02	0.91 ± 0.01	0.02 ± 0.01
K	0.71 ± 0.01	0.13 ± 0.01	0.83 ± 0.01	0.13 ± 0.01	0.94 ± 0.01	0.05 ± 0.01
PR	0.98 ± 0.01	0.74 ± 0.01	0.96 ± 0.01	0.53 ± 0.01	0.93 ± 0.01	0.08 ± 0.01
NB	0.88 ± 0.02	0.48 ± 0.02	0.87 ± 0.02	0.29 ± 0.02	0.91 ± 0.01	0.02 ± 0.01
ANB	0.97 ± 0.01	0.67 ± 0.02	0.94 ± 0.01	0.44 ± 0.01	0.92 ± 0.01	0.05 ± 0.01
KS	0.78 ± 0.01	0.21 ± 0.01	0.85 ± 0.02	0.15 ± 0.01	0.91 ± 0.01	0.01 ± 0.01
LR	0.94 ± 0.01	0.61 ± 0.02	0.91 ± 0.01	0.39 ± 0.02	0.91 ± 0.01	0.03 ± 0.01
H	0.91 ± 0.01	0.51 ± 0.02	0.89 ± 0.01	0.31 ± 0.01	0.91 ± 0.01	0.02 ± 0.01
EI	0.95 ± 0.01	0.65 ± 0.02	0.96 ± 0.01	0.55 ± 0.02	0.94 ± 0.01	0.08 ± 0.01
CD	0.98 ± 0.01	0.76 ± 0.01	0.97 ± 0.01	0.52 ± 0.01	0.93 ± 0.01	0.07 ± 0.01
CI1	0.98 ± 0.01	0.74 ± 0.01	0.95 ± 0.01	0.49 ± 0.01	0.93 ± 0.01	0.06 ± 0.01
CI2	0.98 ± 0.01	0.73 ± 0.01	0.96 ± 0.01	0.49 ± 0.01	0.93 ± 0.01	0.06 ± 0.01

Table 2: Same as in Table 1, but for the subset of social networks.

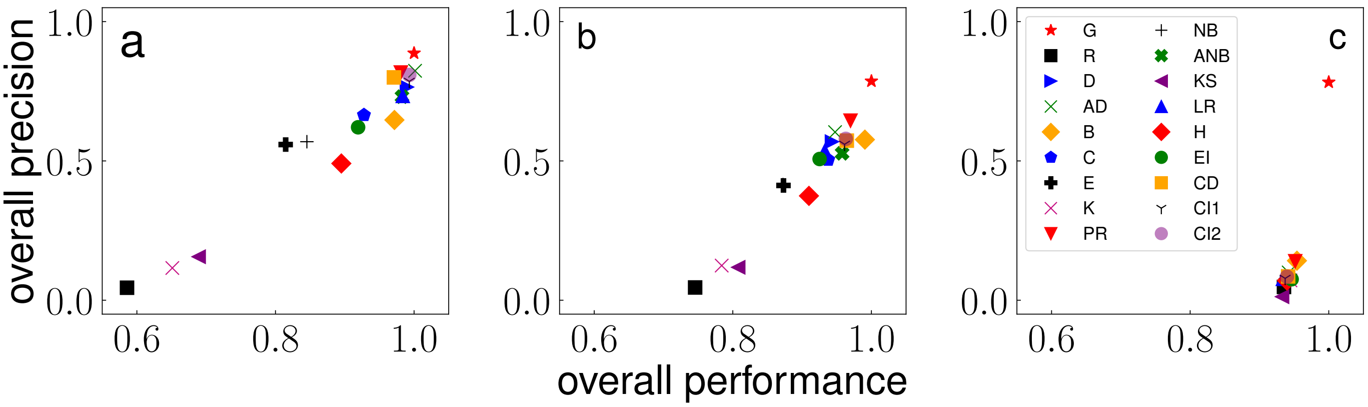

Figure 24: All three regimes for T=0.05 for biological networks

	Subcritical		Critical		Supercritical	
Method	$\langle g_m \rangle$	$\langle r_m \rangle$	$\langle g_m \rangle$	$\langle r_m \rangle$	$\langle g_m \rangle$	$\langle r_m \rangle$
G	1.00 ± 0.00	0.89 ± 0.02	1.00 ± 0.00	0.79 ± 0.03	1.00 ± 0.00	0.78 ± 0.04
R	0.59 ± 0.02	0.04 ± 0.01	0.75 ± 0.04	0.05 ± 0.01	0.94 ± 0.01	0.05 ± 0.01
D	0.99 ± 0.02	0.77 ± 0.07	0.94 ± 0.02	0.57 ± 0.07	0.94 ± 0.02	0.08 ± 0.02
AD	1.00 ± 0.02	0.82 ± 0.05	0.95 ± 0.02	0.60 ± 0.07	0.94 ± 0.02	0.10 ± 0.02
B	0.97 ± 0.02	0.65 ± 0.05	0.99 ± 0.03	0.58 ± 0.05	0.95 ± 0.01	0.14 ± 0.03
C	0.93 ± 0.03	0.66 ± 0.08	0.94 ± 0.02	0.50 ± 0.08	0.94 ± 0.01	0.08 ± 0.02
E	0.81 ± 0.08	0.56 ± 0.12	0.87 ± 0.06	0.41 ± 0.10	0.94 ± 0.02	0.07 ± 0.02
K	0.65 ± 0.04	0.12 ± 0.03	0.78 ± 0.05	0.12 ± 0.02	0.94 ± 0.01	0.07 ± 0.04
PR	0.98 ± 0.01	0.82 ± 0.04	0.97 ± 0.01	0.64 ± 0.05	0.95 ± 0.01	0.14 ± 0.02
NB	0.85 ± 0.08	0.57 ± 0.11	0.87 ± 0.05	0.42 ± 0.10	0.94 ± 0.02	0.07 ± 0.02
ANB	0.98 ± 0.02	0.73 ± 0.07	0.96 ± 0.04	0.53 ± 0.08	0.94 ± 0.02	0.07 ± 0.02
KS	0.69 ± 0.03	0.16 ± 0.02	0.81 ± 0.03	0.12 ± 0.02	0.93 ± 0.02	0.01 ± 0.01
LR	0.98 ± 0.04	0.73 ± 0.08	0.93 ± 0.03	0.54 ± 0.08	0.93 ± 0.02	0.08 ± 0.02
H	0.90 ± 0.07	0.49 ± 0.07	0.91 ± 0.04	0.37 ± 0.07	0.94 ± 0.02	0.07 ± 0.02
EI	0.92 ± 0.02	0.62 ± 0.04	0.93 ± 0.02	0.51 ± 0.04	0.95 ± 0.01	0.08 ± 0.02
CD	0.97 ± 0.02	0.80 ± 0.05	0.96 ± 0.03	0.57 ± 0.07	0.94 ± 0.02	0.09 ± 0.02
CI1	0.99 ± 0.02	0.79 ± 0.05	0.96 ± 0.03	0.56 ± 0.07	0.94 ± 0.02	0.08 ± 0.02
CI2	0.99 ± 0.01	0.81 ± 0.04	0.96 ± 0.02	0.58 ± 0.07	0.94 ± 0.02	0.08 ± 0.02

Table 3: Same as in Table 1, but for the subset of biological networks.

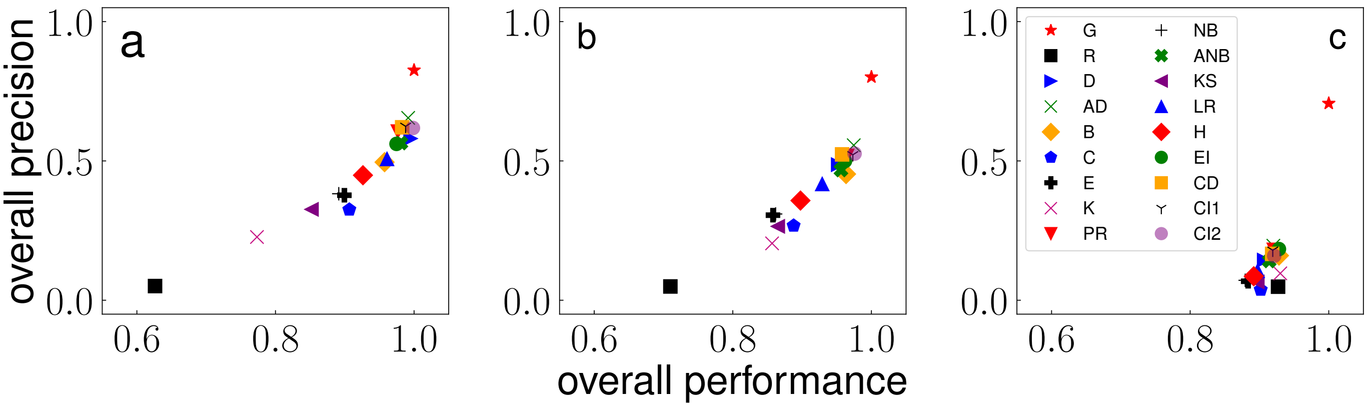

Figure 25: All three regimes for T=0.05 for information networks

	Subcritical		Critical		Supercritical	
Method	$\langle g_m \rangle$	$\langle r_m \rangle$	$\langle g_m \rangle$	$\langle r_m \rangle$	$\langle g_m \rangle$	$\langle r_m \rangle$
G	1.00 ± 0.00	0.83 ± 0.02	1.00 ± 0.00	0.80 ± 0.02	1.00 ± 0.00	0.71 ± 0.03
R	0.63 ± 0.02	0.05 ± 0.01	0.71 ± 0.02	0.05 ± 0.01	0.93 ± 0.02	0.05 ± 0.01
D	1.00 ± 0.01	0.58 ± 0.05	0.95 ± 0.01	0.49 ± 0.04	0.91 ± 0.01	0.14 ± 0.03
AD	0.99 ± 0.01	0.65 ± 0.05	0.97 ± 0.01	0.56 ± 0.04	0.92 ± 0.01	0.20 ± 0.03
B	0.96 ± 0.01	0.50 ± 0.03	0.96 ± 0.01	0.45 ± 0.03	0.93 ± 0.01	0.16 ± 0.03
C	0.91 ± 0.02	0.33 ± 0.04	0.89 ± 0.03	0.27 ± 0.03	0.90 ± 0.01	0.04 ± 0.01
E	0.90 ± 0.04	0.38 ± 0.04	0.86 ± 0.05	0.31 ± 0.03	0.88 ± 0.02	0.07 ± 0.02
K	0.77 ± 0.02	0.23 ± 0.05	0.86 ± 0.03	0.20 ± 0.05	0.93 ± 0.02	0.10 ± 0.03
PR	0.98 ± 0.01	0.61 ± 0.06	0.97 ± 0.01	0.52 ± 0.05	0.92 ± 0.01	0.18 ± 0.03
NB	0.89 ± 0.03	0.38 ± 0.04	0.86 ± 0.04	0.31 ± 0.03	0.88 ± 0.02	0.07 ± 0.02
ANB	0.98 ± 0.01	0.56 ± 0.06	0.96 ± 0.01	0.47 ± 0.05	0.91 ± 0.01	0.14 ± 0.04
KS	0.85 ± 0.03	0.33 ± 0.03	0.86 ± 0.03	0.27 ± 0.02	0.90 ± 0.02	0.07 ± 0.02
LR	0.96 ± 0.02	0.51 ± 0.06	0.93 ± 0.02	0.42 ± 0.05	0.90 ± 0.01	0.11 ± 0.03
H	0.93 ± 0.02	0.45 ± 0.03	0.90 ± 0.02	0.36 ± 0.03	0.89 ± 0.01	0.09 ± 0.02
EI	0.97 ± 0.01	0.56 ± 0.05	0.96 ± 0.01	0.50 ± 0.04	0.93 ± 0.01	0.18 ± 0.03
CD	0.98 ± 0.01	0.62 ± 0.06	0.96 ± 0.01	0.52 ± 0.05	0.92 ± 0.01	0.17 ± 0.03
CI1	0.99 ± 0.01	0.62 ± 0.05	0.97 ± 0.01	0.52 ± 0.04	0.92 ± 0.01	0.18 ± 0.04
CI2	1.00 ± 0.02	0.62 ± 0.05	0.98 ± 0.01	0.53 ± 0.04	0.92 ± 0.01	0.16 ± 0.03

Table 4: Same as in Table 1, but for the subset of information networks.

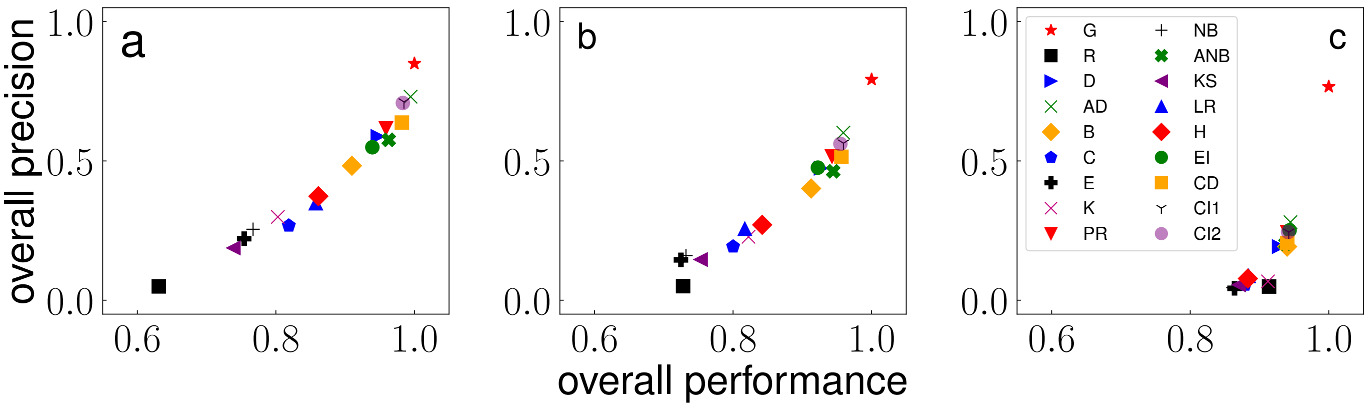

Figure 26: All three regimes for T=0.05 for technological networks

	Subcritical		Critical		Supercritical	
Method	$\langle g_m \rangle$	$\langle r_m \rangle$	$\langle g_m \rangle$	$\langle r_m \rangle$	$\langle g_m \rangle$	$\langle r_m \rangle$
G	1.00 ± 0.00	0.85 ± 0.02	1.00 ± 0.00	0.79 ± 0.03	1.00 ± 0.00	0.77 ± 0.03
R	0.63 ± 0.01	0.05 ± 0.01	0.73 ± 0.01	0.05 ± 0.01	0.91 ± 0.02	0.05 ± 0.01
D	0.95 ± 0.01	0.59 ± 0.02	0.93 ± 0.01	0.47 ± 0.03	0.93 ± 0.01	0.19 ± 0.05
AD	0.99 ± 0.01	0.73 ± 0.02	0.96 ± 0.01	0.60 ± 0.04	0.94 ± 0.01	0.28 ± 0.07
B	0.91 ± 0.03	0.48 ± 0.04	0.91 ± 0.02	0.40 ± 0.04	0.94 ± 0.01	0.19 ± 0.06
C	0.82 ± 0.03	0.27 ± 0.02	0.80 ± 0.03	0.19 ± 0.02	0.88 ± 0.02	0.06 ± 0.02
E	0.75 ± 0.04	0.22 ± 0.03	0.72 ± 0.05	0.14 ± 0.02	0.86 ± 0.03	0.04 ± 0.01
K	0.80 ± 0.02	0.30 ± 0.03	0.82 ± 0.02	0.23 ± 0.02	0.91 ± 0.02	0.07 ± 0.02
PR	0.96 ± 0.01	0.62 ± 0.02	0.94 ± 0.01	0.52 ± 0.03	0.94 ± 0.01	0.24 ± 0.06
NB	0.77 ± 0.04	0.25 ± 0.03	0.73 ± 0.05	0.16 ± 0.02	0.86 ± 0.03	0.05 ± 0.02
ANB	0.96 ± 0.01	0.58 ± 0.04	0.94 ± 0.01	0.46 ± 0.04	0.94 ± 0.01	0.20 ± 0.05
KS	0.74 ± 0.03	0.19 ± 0.02	0.75 ± 0.03	0.15 ± 0.02	0.87 ± 0.03	0.06 ± 0.02
LR	0.86 ± 0.02	0.35 ± 0.02	0.82 ± 0.02	0.26 ± 0.03	0.88 ± 0.02	0.09 ± 0.03
H	0.86 ± 0.02	0.37 ± 0.02	0.84 ± 0.02	0.27 ± 0.02	0.88 ± 0.02	0.08 ± 0.03
EI	0.94 ± 0.01	0.55 ± 0.04	0.92 ± 0.01	0.48 ± 0.04	0.94 ± 0.01	0.25 ± 0.06
CD	0.98 ± 0.01	0.64 ± 0.03	0.96 ± 0.01	0.51 ± 0.03	0.94 ± 0.01	0.21 ± 0.05
CI1	0.98 ± 0.01	0.71 ± 0.02	0.96 ± 0.01	0.56 ± 0.03	0.94 ± 0.01	0.24 ± 0.06
CI2	0.98 ± 0.01	0.71 ± 0.02	0.95 ± 0.01	0.56 ± 0.03	0.94 ± 0.01	0.24 ± 0.06

Table 5: Same as in Table 1, but for the subset of technological networks.

5 Analysis for using $V_m^{(T)}$ as the main metric - T=0.05

5.1 Performance relative to greedy vs cumulative distribution

5.1.1 Subcritical Regime

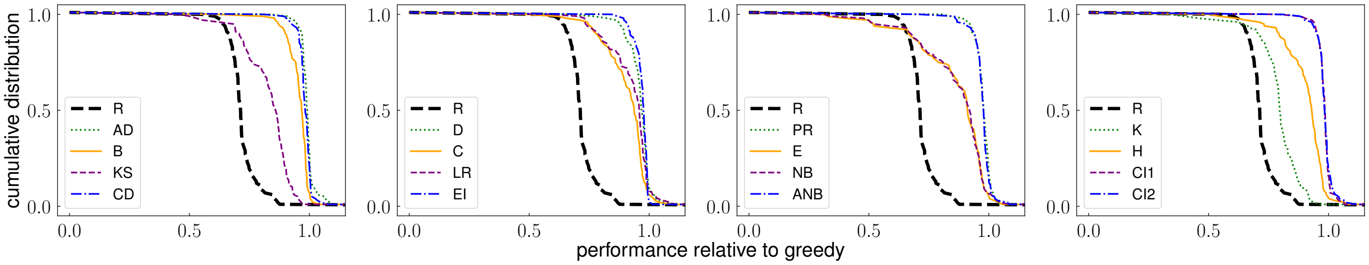

Figure 27: $p = 0.5p_c$

5.1.2 Critical Regime

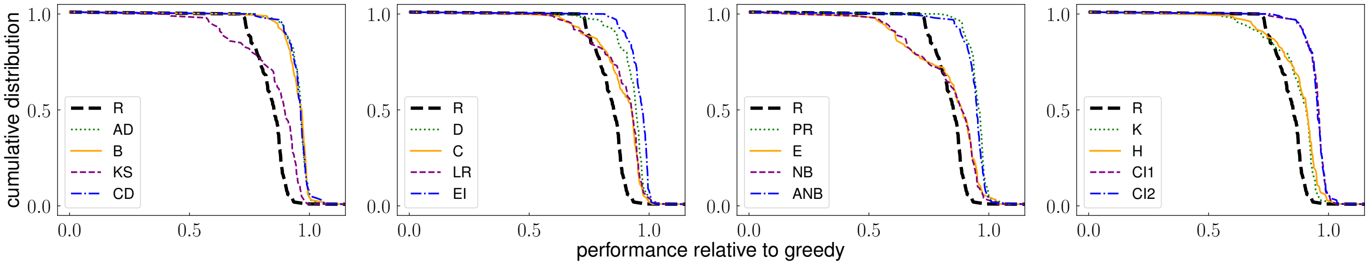

Figure 28: $p = p_c$

5.1.3 Supercritical Regime

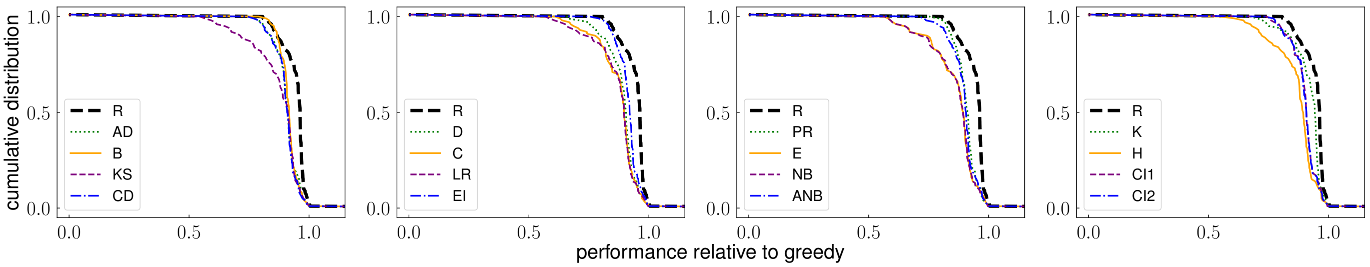

Figure 29: $p = 2p_c$

5.2 Overall performance vs overall precision

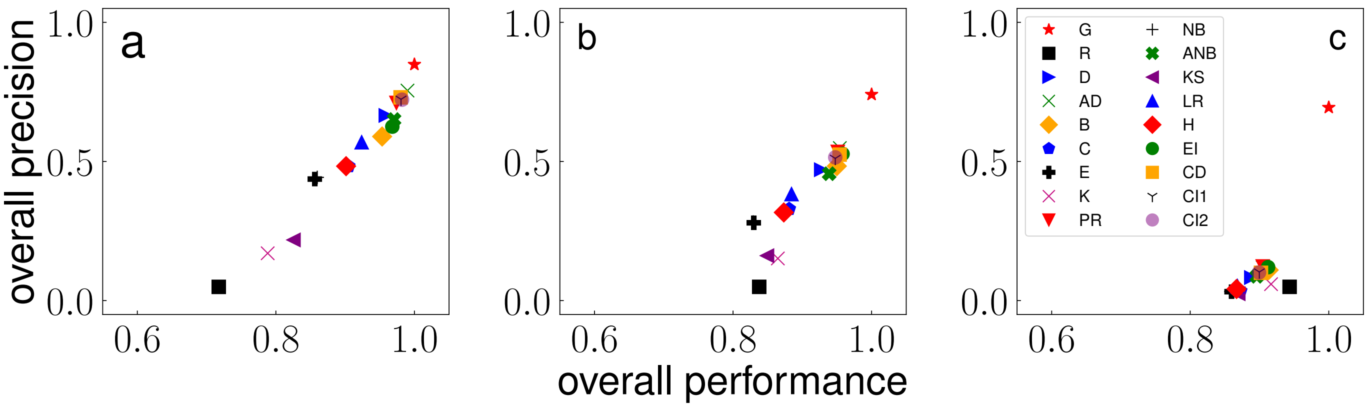

Figure 30: All three regimes for T=0.05

6 Table of results for hybrid methods - T=0.10

Method	Features	**Subcritical**	**Critical**	**Supercritical**
AD	c_{AD}	1.000	1.000	1.000
	$\langle g_m \rangle$	0.981	0.953	0.907
	$\langle r_m \rangle$	0.687	0.493	0.123
CD	c_{CD}	1.000	1.000	1.000
	$\langle g_m \rangle$	0.977	0.951	0.904
	$\langle r_m \rangle$	0.654	0.463	0.100
B	c_B	1.000	1.000	1.000
	$\langle g_m \rangle$	0.951	0.945	0.914
	$\langle r_m \rangle$	0.551	0.427	0.108
AD,B	c_{AD}	0.801	0.606	-0.025
	c_B	-0.146	-0.044	0.058
	$\langle g_m \rangle$	0.976	0.952	0.925
	$\langle r_m \rangle$	0.688	0.494	0.098
AD,PR,LR	c_{AD}	1.126	0.862	0.087
	c_{PR}	-0.024	0.422	0.714
	c_{LR}	-0.508	-0.746	-0.751
	$\langle g_m \rangle$	0.983	0.977	0.963
	$\langle r_m \rangle$	0.763	0.574	0.303
PR,LR,CD	c_{PR}	0.253	0.639	0.746
	c_{LR}	-0.569	-0.788	-0.751
	c_{CD}	0.939	0.709	0.057
	$\langle g_m \rangle$	0.987	0.975	0.963
	$\langle r_m \rangle$	0.711	0.552	0.301
AD,B,LR	c_{AD}	1.215	1.042	0.302
	c_B	-0.128	-0.025	0.073
	c_{LR}	-0.511	-0.537	-0.405
	$\langle g_m \rangle$	0.981	0.963	0.921
	$\langle r_m \rangle$	0.76	0.588	0.283
PR,LR,EI	c_{PR}	0.418	0.653	0.741
	c_{LR}	-0.098	-0.437	-0.721
	c_{EI}	0.336	0.377	0.036
	$\langle g_m \rangle$	0.975	0.961	0.964
	$\langle r_m \rangle$	0.660	0.552	0.299

Table 6: Performance of hybrid methods for the identification of influential spreaders in networks for T=0.10. Statistical errors (not reported) are smaller than 0.001.
